# Automated Synthesis of C1-Functionalized Oligosaccharides

**DOI:** 10.1021/jacs.4c11798

**Published:** 2024-12-31

**Authors:** Georg B. Niggemeyer, José A. Danglad-Flores, Peter H. Seeberger

**Affiliations:** † Department of Biomolecular Systems, Max-Planck-Institute of Colloids and Interfaces, Potsdam 14476, Germany; ‡ Freie Universität Berlin, Institute of Chemistry and Biochemistry, Berlin 14195, Germany

## Abstract

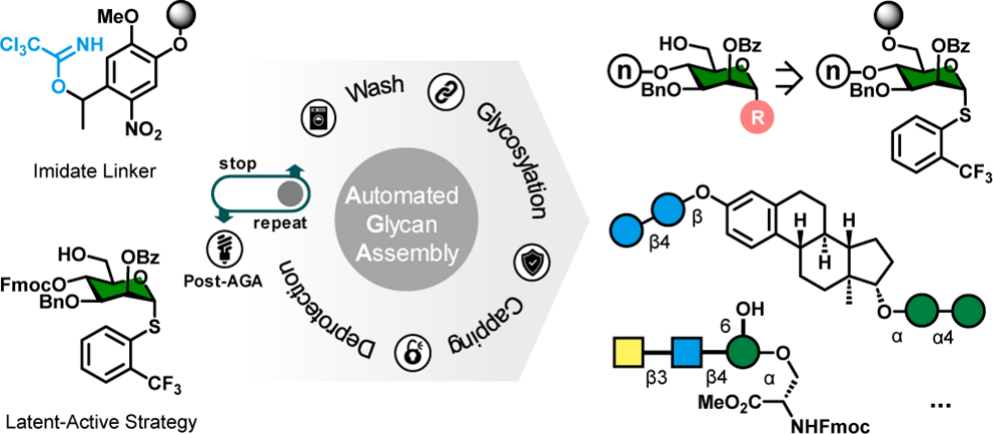

Automated glycan assembly (AGA) streamlines the synthesis of complex oligosaccharides. The reducing end of the oligosaccharide serves as an attachment site to the polymer support to liberate a free reducing end or an aminopentanol for ready conjugation to carrier proteins or surfaces. The facile installation of different aglycons on oligosaccharides has not been possible via AGA until now. Here, we describe a latent-active approach enabled by a traceless photolabile linker that allows for bidirectional AGA and ready introduction of various aglycons. Oligosaccharide thioglycosides, peptidoglycans, prototypical saponins, and click-chemistry-based conjugates are synthesized to illustrate the versatility of the method.

## Introduction

In nature, some carbohydrates, such as cellulose, bear a free reducing end, while others are part of glycoconjugates such as saponins. These amphiphilic molecules are biologically important but constitute a synthetic challenge.^1,2^ Ubiquitous in the plant kingdom, saponins bear a triterpene- or steroid aglycone that is responsible for the namesake detergent character of these glycans.^2,3^ This heterogeneous class of compounds serves in many applications, ranging from food additives^4^ to adjuvants^5^ and cell-permeabilizing nonionic surfactants in molecular biology.^6^ Currently, saponins are prepared either through laborious total synthesis,^7^ complicated extractions from natural matrices such as tree bark, or by tailored heterologous expression systems.^8^

Another class of glycoconjugates are *O*-glycans where oligosaccharides are linked to proteins via the side chains of serine or threonine. Such post-translational modifications serve many functions. In the case of dystroglycan, the glycan is essential for establishing a link between the intracellular actin cytoskeleton and the extracellular matrix. Defects lead to muscular dystrophy.^9,10^

**Figure 1:**
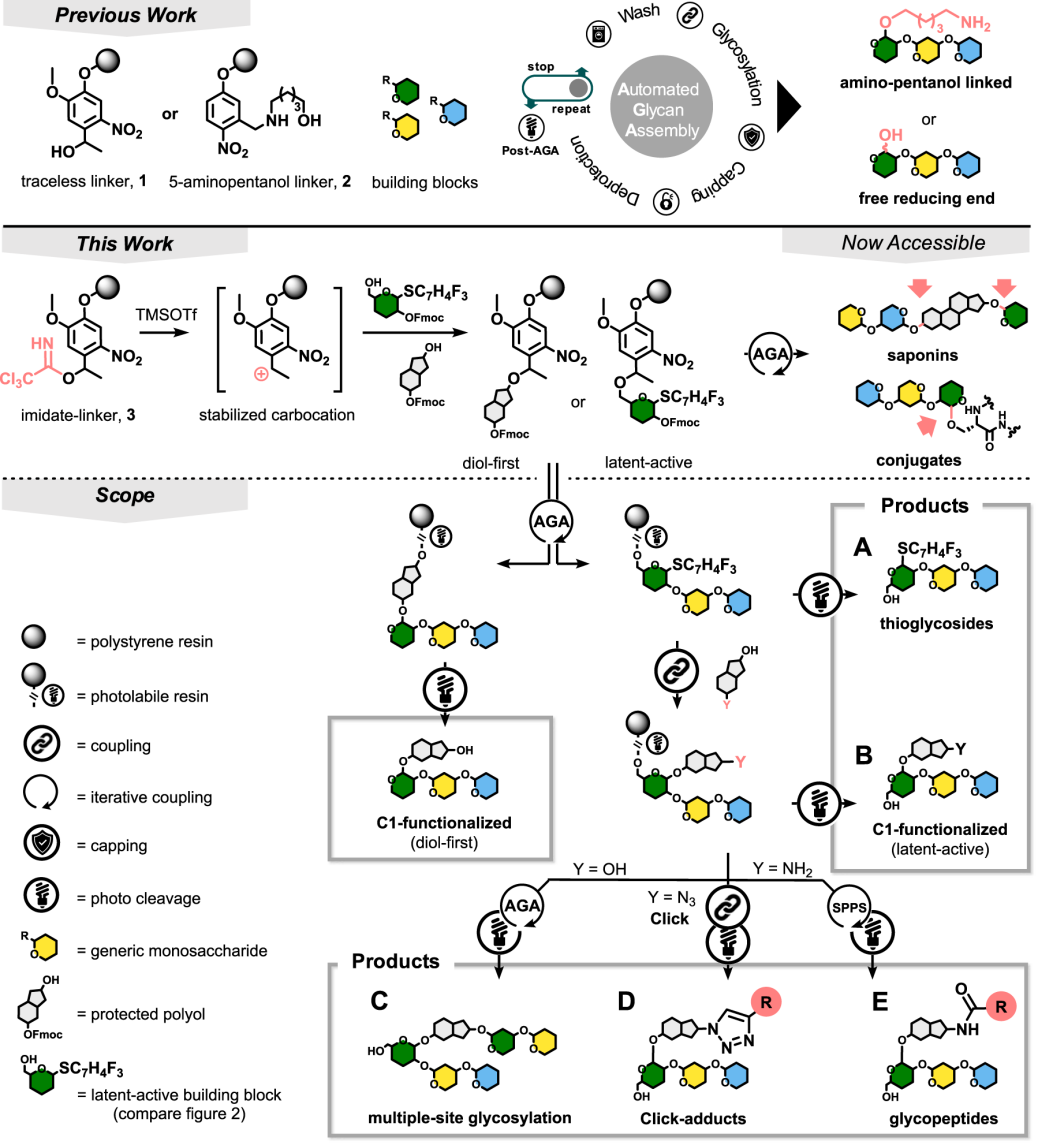
Conventional AGA produces glycans functionalized with an aminopentanol or a free reducing end using current linkers. The trichloroacetimidate linker introduced in this work opens up the C1-position for the variable substituents. The scope is divided in five classes of products, which can matched with Scheme 1: (A) oligosaccharide thioglycosides, (B) C1-functionalized glycans via the diol-first or latent-active approach, (C) multiply glycosylated aglycons from bidirectional AGA, (D) click-adducts, and (D) glycosyl-amides obtained by merging AGA and SPPS.

An efficient synthetic approach to accessing different classes of biologically important glycoconjugates is desirable. Automated glycan assembly (AGA) is a time- and labor-efficient platform to access complex glycans.^11^ Yet, the synthetic logic of assembling glycans from the reducing end to the nonreducing end complicates the introduction of variable aglycons. Here, we describe the development of a fully automated latent-active approach that renders the reducing end of an oligosaccharide ready for the introduction of different aglycons. The first sugar building block (BB) is connected to the solid support via an ether linkage that results from the reaction of a hydroxyl group and a trichloroacetimidate (TCAI) photolabile linker (Figure 1). Utilizing this approach for AGA, we produced thioglycosides that were coupled to various glycosyl acceptors. The functional groups introduced via the aglycone can be utilized in bidirectional AGA, amide coupling, or *Cu*AAC-click conjugation (copper-catalyzed azide-alkyne cycloaddition).

## Results

### Latent-Active AGA

In order to accommodate a latent-active leaving group at the reducing end of the nascent oligosaccharide, the first building block was attached to the polymer resin via its C6 hydroxyl group. A host of chemistries has been applied to fashion similar ether linkages to solid supports.^12−14^ Carbohydrates were indirectly attached via amidation of a succinic acid linker^15^ or Migita-Stille coupling of a 4-iodobenzenesulfonyl linker.^16^ Both approaches are incompatible with AGA and provide little versatility.

**Figure 2:**
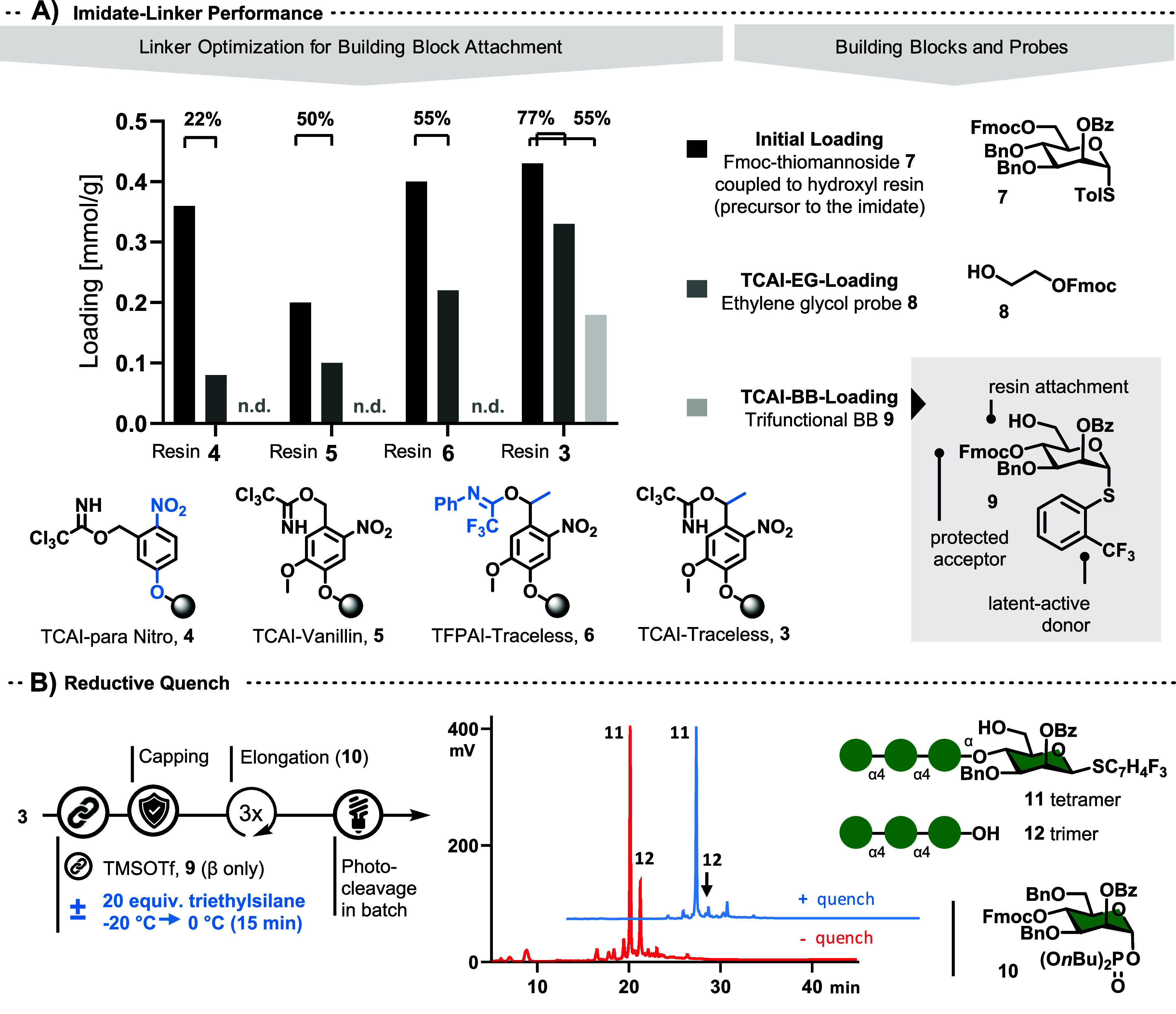
Method development. (A) Optimization of the linker substitution pattern for optimal attachment of hydroxyl building blocks to the solid support. (B) Introduction of a reductive quenching protocol to reduce deletion sequences.

Hanessian and Xie^17^ and Yan and Mayer^18^ utilized a trichloroacetimidate-functionalized Wang resin to attach amino alcohols via a p-alkoxybenzyl carbocation intermediate. We adopted this strategy, utilizing the photolabile ortho-nitrobenzyl linkers routinely used in AGA for facile reaction monitoring during process optimization. Linkers with three different substitution patterns were synthesized and attached to Merrifield resin (SI Chapter 1).^19^ The initial loading was determined by glycosylation of resin (50 mg) with 6-*O*-Fmoc manno-thioglycoside **7** on the automated synthesizer^20^ (Figure 2A), quantifying Fmoc cleavage by UV–vis spectrophotometry.^21^ The hydroxyl resins were transformed into the corresponding imidates in a custom-built bubbling reactor using four cycles of excess trichloroacetonitrile with a catalytic amount of DBU (**3**, **4,** and **5**, Figure 2A; SI Chapter 1.1). Resin **6** was prepared similarly, using *N*-phenyltrifluoroacetimidate chloride, triethylamine, and a catalytic amount of DMAP. Reaction conditions for the alkylation of sugar alcohols were first optimized in solution and then adjusted to the synthesizer (SI Chapter 1.2). Although *BF*_3_·*O*Et_2_ proved to be an excellent activator, TMS*O*Tf was employed as it is also used for the acidic wash during AGA.

The attachment process starts at −40 °C when two equivalents of TMSOTf are added to a suspension of the first building block and the resin. After five minutes, the temperature in the reactor is ramped up to −20 °C (4 °C/min). The reaction is stopped after 35 min of incubation.

The four imidate resins were ranked for their ability to react with Fmoc-ethylene glycol probe **8**. Here, the above-mentioned initial loading with BB **7** served as a point of reference to quantify functionalization. “TCAI-Traceless” resin **3** reached the highest functionalization of 77% (Figure 2A). Building block **9** was attached to the TCAI-Traceless linker resin in 55% yield.

Differentially protected thioglycoside building block **9** was synthesized as part of the latent-active strategy (Figure 1).^22,23^ Similar 2-(*CF*_3_)Ph-thioglycosides exhibited remarkably low reactivity in glycosylation reactions.^24,25^ Here, they serve to prevent aglycon transfer during attachment and elongation of the nascent oligosaccharide. A late exchange of the thioacetal was chosen to enable tailoring of the reactivity of the latent-active building block. Most of the six equivalents of BB **9** that were used in this first coupling step were recovered and reused after purification (81%, *n* = 3).

Glycan elongation was performed using three to six equivalents of dibutyl phosphate donors. This type of donor was chosen as part of the latent-active strategy because glycosyl phosphates can be activated orthogonally to thioglycosides. This type of donor is particularly appealing for two reasons. First, it is accessed readily from commercially available thioglycoside building blocks in one step. Second, it can be activated using TMSOTf, a Lewis acid already integral to AGA. However, nothing speaks against exploring other selective or orthogonal donor/activator pairs.

Photocleavage from the solid support was performed in a batch process on fully protected glycans or after on-resin methanolysis. To this end, a fritted syringe loaded with resin and DMF (5 mL) was irradiated with a 370 nm Kessil lamp. Stirring with an egg-shaped stir bar not only ensured mixing but also crushed the beads, maximizing the irradiated surface area (SI Chapter 1.3).^26^

### Oligosaccharide Synthesis

The assembly of model tetramer **11** revealed deletion sequence **12** as a major byproduct (Figure 2B). Trisaccharide **12**, lacking the first building block (**9**), is the result of an incomplete first coupling step. Here, the activated TCAI-traceless linker partially forms a species that later gives rise to a glycosyl acceptor. Glycan elongation on this extra acceptor via the following three glycosylations furnishes **12**. Acetylation after the resin attachment of **9** failed to suppress the deletion sequence. Delivery of excess scavenger, such as methanol, at the end of the first coupling step reduced the formation of **12**, whereas triethylsilane (TES) deactivated the resin completely (Figure 2B and SI Chapter 1.4). Hydrosilanes are mild hydride donors under acidic conditions and are routinely used as cation scavengers in peptide chemistry.^27^

**Scheme 1:**
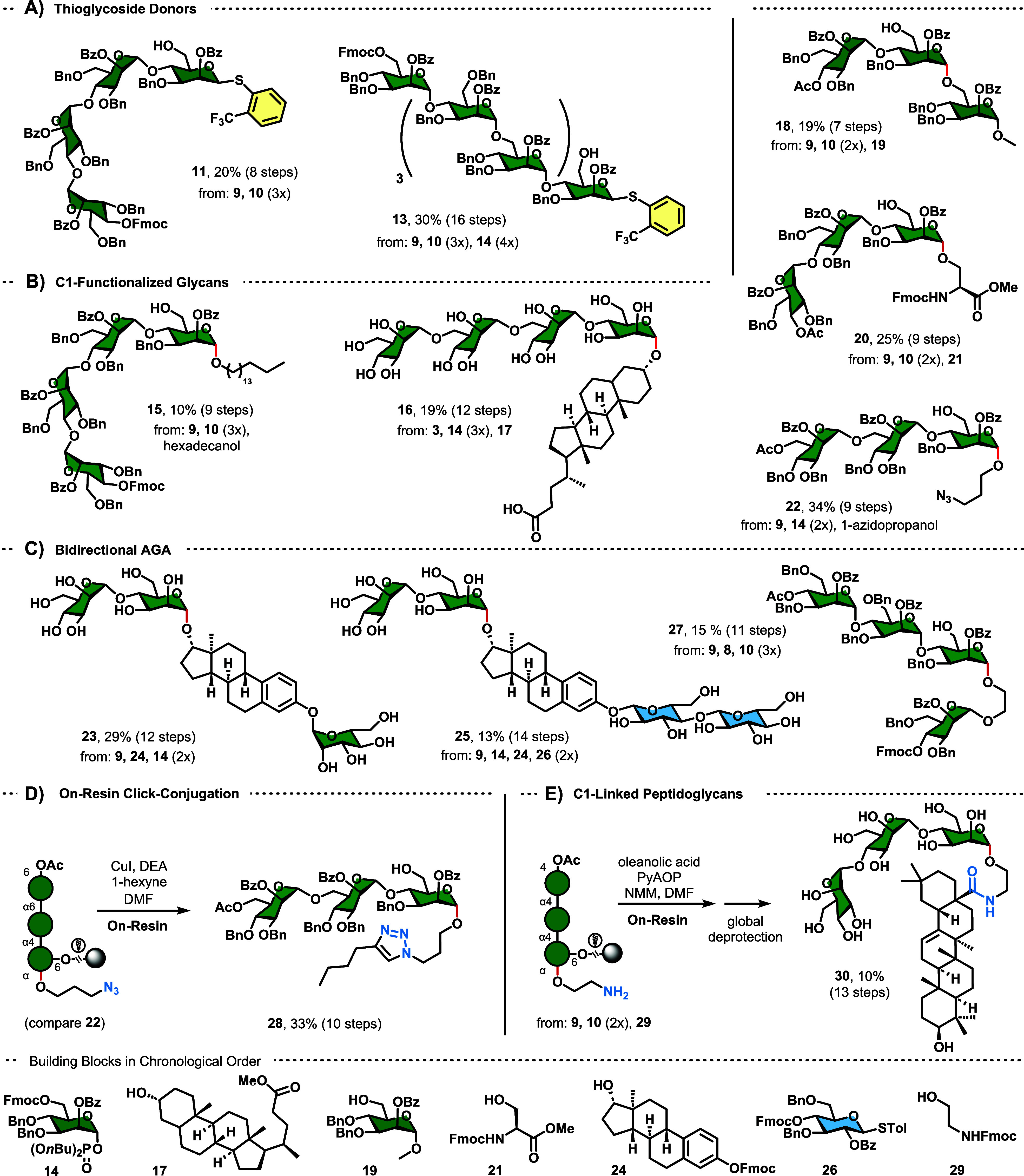
Scope with Implicit Deprotection Steps; (A) Thioglycosides, (B) C1-functionalized Glycans, (C) Multiply Glycosylated Aglycons, (D) Glycan Click-Conjugate **29,** and (E) Amidoglycan **30**

The optimized resin loading provided a robust platform for the automated assembly of oligosaccharide thioglycoside donors (Figure 1 class A; Scheme 1A). Similar on-resin donors functioned as intermediates in the synthesis of subsequent glycosides.

The on-resin glycosylation of 2-(*CF*_3_)Ph-thioglycoside donors proceeded at 30–35 °C with three equivalents of triflic acid, based on 55% resin functionalization. With excess nucleophile, little to no hydrolyzed starting material was observed for any glycoside (Figure 1 class B, Scheme 1B). α/β-Mixtures of BB **9** were used in syntheses that included glycosylation of the reducing end, as both species resulted in α-glycosidic bonds selectively in glycosylations with all shown aglycons.

### Scope

The scope of the method was explored by accessing oligosaccharide thioglycosides, C1-modified glycans, glycosides via bidirectional AGA, and conjugates produced by CuAAC or amide coupling (Figure 1, Scheme 1). Selected glycans were deprotected to illustrate the workflow. Yields were calculated based on the initial loading (IL, Figure 2A) to include TCAI-functionalization of the resin and attachment of the first building block (see above).

α-1,4-Linked tetramannose thioglycoside **11** and alternating α-1,4/1,6-linked octamannose thioglycoside **13** were synthesized by leaving the latent-active donor intact (Scheme 1 class A). An isolated yield of 30% for octamer **13** translates into an efficiency of 94% per synthetic step.

Different 2-(*CF*_3_)Ph-thioglycosides were coupled to various aglycons (Scheme 1 class B) such as hexadecanol (**15**), lithocholic acid methyl ester (**16**), methyl 6-hydroxyl-1-*O*-α-mannopyranoside (**18**), Fmoc-serine(*OH*)-*O*Me (**20**) and 1-azidopropanol (**22**). The 15-step synthesis of amphiphile **16** proceeded with a 19% overall yield. After the attachment of building block **9**, the glycan was extended by three units of building block **14**. Next, the tetramer was reacted with lithocholic methyl ester. Photocleavage, hydrogenolysis, hydrolysis, and one final purification gave **16**. Conceptually, **16** is a synthetic saponin, and glycosylated amino acid **20** can be utilized as a building block for SPPS—with or without prior cleavage from the resin.^28^

Glycosylating the C1-position with Fmoc-protected diols enables bidirectional AGA (Figure 1 class C, Scheme 1C). The latent active thioglycosides are assembled in a forward direction before acetic anhydride-capping of the nonreducing end. Glycosylation with a monoprotected diol (e.g., **8**) then allows for glycan elongation in the “opposite” direction. Glycosylation at more than one site of an aglycon is especially relevant, as many natural saponins possess multiple glycosylated sites. Estradiol-linked **23** was synthesized starting from BB **9**, elongating via glycosylation with phosphate BB **10** and acetylation of the nonreducing end after Fmoc removal. The resultant trimannose thioglycoside donor was coupled to 3-Fmoc estradiol (**24**). Subsequent Fmoc removal, glycosylation with **10,** and global deprotection gave the product (29% yield, 92% per step). Glycans **25** and **28** were synthesized accordingly. In the case of **25**, thioglycoside donor **26** was used in the “backwards” direction, as the latent-active thioglycoside group was no longer present at that stage.

The glycosylation with azidopropanol (see **22**, Scheme 1B) highlights the possibility of tapping into the ever-growing pools of azide and alkyne reagents (Figure 1 class D). Click chemistry is frequently used for the synthesis of compound libraries or biological probes. On-resin *Cu*AAC of azide **22** with 1-hexyne produced triazole **28** quantitatively (Scheme 1D).

Attachment of a protected amino alcohol (e.g., **29**) to the nascent oligo thioglycoside donor allowed for seamless transfer of the solid support from AGA to SPPS (Scheme 1 class E, Scheme 1E). Here, oleanolic acid was coupled to the amine precursor of compound **30**.

**Scheme 2:**
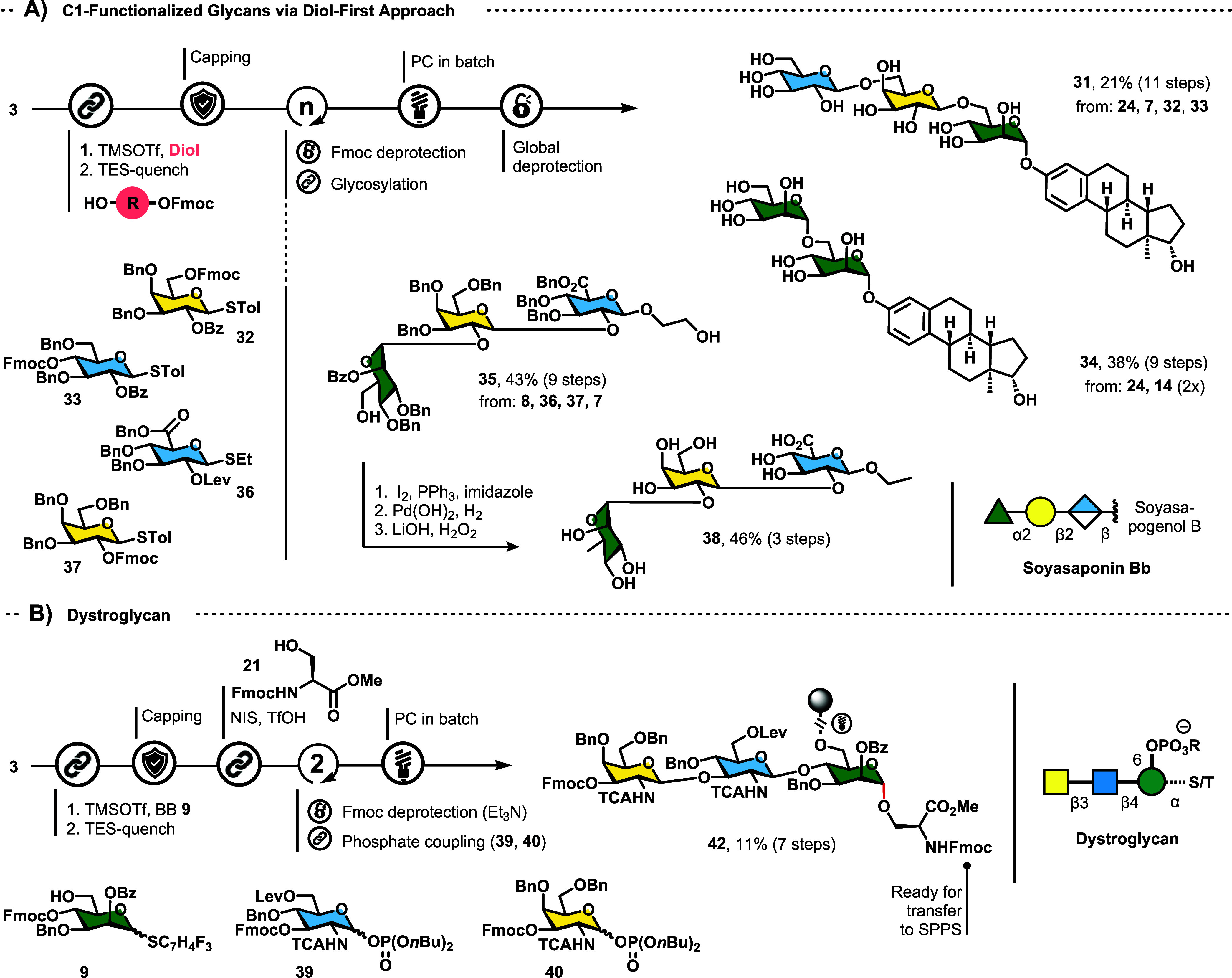
Syntheses Differing from Standard Latent-Active Approach; (A) C1-Functionalized Glycans via the “Diol-First” Approach; (B) Synthesis of Dystroglycan Required the Installation of Serine in the Second Step of the Synthesis

Apart from building block **9**, almost any hydroxyl-containing molecule can be attached to TCAI resin **3** at the beginning of a synthesis.^17,18^ Glycosylation of the resin-bound aglycon can then be performed without any restriction associated with the latent-active approach detailed above. Here, we attached 3-Fmoc-protected estradiol **24** or ethylene glycol probe **8** as the first building block under the same conditions used for trifunctional BB **9** (Figure 1 ”diol first”, Scheme 2A). The yields for this approach were generally higher, hinting at the superior nucleophilicity compared to that of BB **9**. 3-β-Glu-(1 → 6)-β-Gal-(1 → 6)-α-Man estradiol **31** was synthesized from thioglycoside building blocks **7**, **32,** and **33**. 3-Dimannosyl estradiol **34** was synthesized by using phosphate building block **14**. Lastly, an all-D-diastereomer of the soyasaponin Bb glycan (**38**) was synthesized to highlight the ease of transforming the released hydroxyl group of the linker into useful synthetic handles such as iodides.^29^ Fully protected 6-*OH*-α-Man-(1 → 2)-β-Gal-(1 → 2)-β-GluA ethylene glycol was synthesized from building blocks **8**, **36**, **37,** and **7** in 43% yield. Trisaccharide **35** was then treated with iodine, triphenylphosphine, and imidazole to furnish the diiodide that was fully reduced during hydrogenation. Hydrolysis of the remaining benzoyl group afforded **38** in a 20% overall yield.

The mammalian *O*-glycan dystroglycan was synthesized to illustrate the use of bidirectional AGA for natural product synthesis (Scheme 2B). First, a thioglycoside precursor to dystroglycan was assembled in 26% yield using building blocks **9**, **39,** and **40** (**41**, SI Chapter 3.3). Lower concentrations of TMS*O*Tf were used to circumvent the intrinsic acid sensitivity of this amino sugar sequence. Consecutive glycosylation with Fmoc-Ser(*OH*)-*O*Me (compare **20**, Scheme 1B) resulted in the complete decomposition of **41**. However, **42** was obtained by introducing serine **21** as the second building block. Advancing the synthesis analogous to that of **41** produced α-dystroglycan.

## Conclusion

We describe a versatile method for the automated glycan assembly of oligosaccharides containing diverse aglycons. To this end, a photolabile trichloroacetimidate acceptor resin was matched with a latent-active glycosylation approach. This method provides automated access to oligosaccharide thioglycosides that can be transformed into the glycosides of choice. The incorporation of bifunctional linkers enabled bidirectional AGA as well as consecutive SPPS and CuAAC.

This work paves the way for the target-oriented synthesis of saponins, glycoconjugates, and cyclic oligosaccharides. The generation of collections of molecules with varying glycan or aglycon portions can now be significantly streamlined.

## Supporting Information Available

Additional experimental details, materials, and methods, including photographs of the experimental setup and analytical data (PDF)

## Abbreviations

AGA: Automated Glycan Assembly
BB: Building Block
CuAAC: copper-catalyzed azide alkyne cycloaddition
IL: Initial Loading
PC: Photo Cleavage
SPPS: Solid-Phase Peptide Synthesis
TCAI: trichloroacetimidate
TES: triethylsilane
