# Supplementary material for: Automated Synthesis of C1-Functionalized Oligosaccharides
Source: J Am Chem Soc. 2024 Dec 31;147(2):1649–55. doi: 10.1021/jacs.4c11798 (PMC11744757; doi:10.1021/jacs.4c11798)
Supplement: Supplementary file 1 — ja4c11798_si_001.pdf [file ja4c11798_si_001.pdf]

Supporting Information

Automated synthesis of C1-functionalized oligosaccharides

Georg B. Niggemeyer, José A. Danglad-Flores and Peter H. Seeberger*

Table of Contents

General Information....2

1 Method development....3

1.1 Trichloroacetimidation....3

1.2 Optimization of TCAI-based coupling....7

1.3 Optimization of photocleavage conditions....18

1.4 Methanol as quenching agent....20

2 Synthesis....21

2.1 Monosaccharide building blocks....21

2.2 Nucleophiles....59

2.3 Photolabile Resins....73

3 Automated Glycan Assembly....90

3.1 Standard solutions....90

3.2 Automation programs....91

3.3 Products from automated glycan assembly....99

References....204

General Information

All chemicals used were reagent grade and used as supplied (Sigma Aldrich, TCI, Thermo Fisher, Glyco Universe) unless stated otherwise. All automated glycan assemblies were performed on home-built synthesizers developed at the Max Planck Institute of Colloids and Interfaces.[1] Analytical thin-layer chromatography (TLC) was performed on Merck silica gel 60 F254 plates (0.25 mm). Compounds were visualized by UV irradiation or dipping the plate in a staining solution (CAM: 48 g/L ammonium molybdate, 60 g/L ceric ammonium molybdate in 6% H_2SO_4 aqueous solution). Flash column chromatography was carried out by using a forced flow of the indicated solvent on Fluka Kieselgel 60 M (0.04 – 0.063 mm). Analysis and purification by normal and reverse-phase HPLC were performed by using an Agilent 1200 series. Products were lyophilized using a Christ Alpha 2-4 LD plus freeze dryer. ^{1}H, ^{13}C, and HSQC NMR spectra were recorded on a Varian 400-MR (400 MHz), Varian 600-MR (600 MHz), or Bruker Biospin AVANCE700 (700 MHz) spectrometer. Spectra were recorded in deuterated solvents by using the chemical shift of solvent residual peak as the internal standard ($CDCl_3$: 7.26 ppm 1H, 77.16 ppm 13C; D_2O: 4.79 ppm 1H). In cases of D_2O/D_3-acetonitrile mixtures, the D_2O peak was taken as reference. High-resolution mass spectra were acquired using a 6210 ESI-TOF mass spectrometer (Agilent). Optical rotations were measured using a 10 cm glass chamber using a Schmidt + Haensch UniPol L1000 polarimeter. Concentration of the analyte in the given solvent is given as “c” (g / 100 mL).

1 Method development

This section details the development of the synthetic platform presented in the main body of the publication. Reactions performed on solid support were mostly studied in solution before transferring them to AGA.

1.1 Trichloroacetimidation

1.1.1 In solution

To optimize the trichloroacetimidation of photolabile linkers, this reaction was first optimized in solution before moving to solid support. An access of trichloroacetonitrile and the use of DBU a catalytic amount of DBU proved to be optimal (Table 1, entry 6).

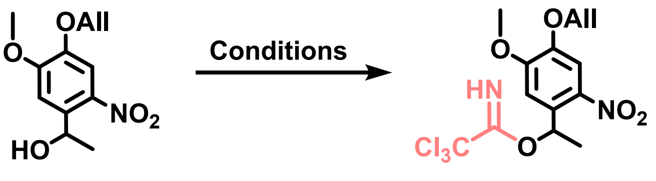

Table 1: In-solution optimization of linker trichloroacetimidation.

**No**	**Cl_3CN** [equiv.]	**Base** [equiv.]	**Time**	**Yield** [%]
1	1 → 5	DABCO (0.1)	16 h	-
2	1 → 5	DMAP (0.1)	16 h	-
3	1 → 5	tetramethyl guanidine (0.1)	16 h	traces
4	1	DBU (0.5)	180 min	64
5	1	DBU (0.1)	60 min	84
6*	5	DBU (0.1)	65 min	98

Reactions were performed in solution at 0.1 mM in CH_2Cl_2 at RT and at scales ranging from 0.2 to 6 mmol. *reaction started at 0 °C removing the ice bath after 5 min.

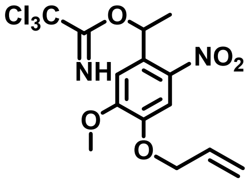

SI2

1-(4-(Allyloxy)-5-methoxy-2-nitrophenyl)ethyl 2,2,2-trichloroacetimidate (**SI2**)

SI1 (300 mg, 1.18 mmol) under nitrogen in CH_2Cl_2 (0.1 M) at 0°C were added trichloroacetonitrile (360 µL, 3.54 mmol, 3 eq.) followed by dropwise addition of DBU (17.6 µL, 118 µmol, 0.1 eq.) in 100 µL CH_2Cl_2. The reaction was stirred for 5 min at 0°C followed by 1 h at 23°C. The reaction was diluted with CH_2Cl_2 and washed with sat. aq. NH_4Cl. The phases were separated and aqueous phase was extracted with CH_2Cl_2 twice. The combined organic phases were dried over Na_2SO_4 and the solvent was removed under reduced pressure. **SI2** was isolated by FCC (7:1 hexane/EtOAc; Rf = 0.36) in 98% yield as a slightly yellow solid (459 mg, 1.15 µmol).

^{1}H NMR (400 MHz, $CDCl_3$) δ 8.28 (s, 1H), 7.61 (d, J = 0.9 Hz, 1H), 7.18 (d, J = 1.0 Hz, 1H), 6.57 (qd, J = 6.4, 1.0 Hz, 1H), 6.17 – 5.89 (m, 1H), 5.46 (dq, J = 17.3, 1.5 Hz, 1H), 5.36 (dq, J = 10.5, 1.3 Hz, 1H), 4.66 (dt, J = 5.5, 1.4 Hz, 2H), 3.93 (d, J = 1.0 Hz, 3H), 1.75 (d, J = 6.4 Hz, 3H).

^{13}C NMR (101 MHz, $CDCl_3$) δ 160.8, 154.2, 147.0, 140.00, 133.2, 132.0, 119.4, 109.2, 107.9, 91.6, 73.3, 70.2, 56.5, 22.0.

HRMS (ESI): $C_{14}H_{15}N_2NaO_5$ $[M+Na]^+$; calculated: 418.9944, found: 418.9942.

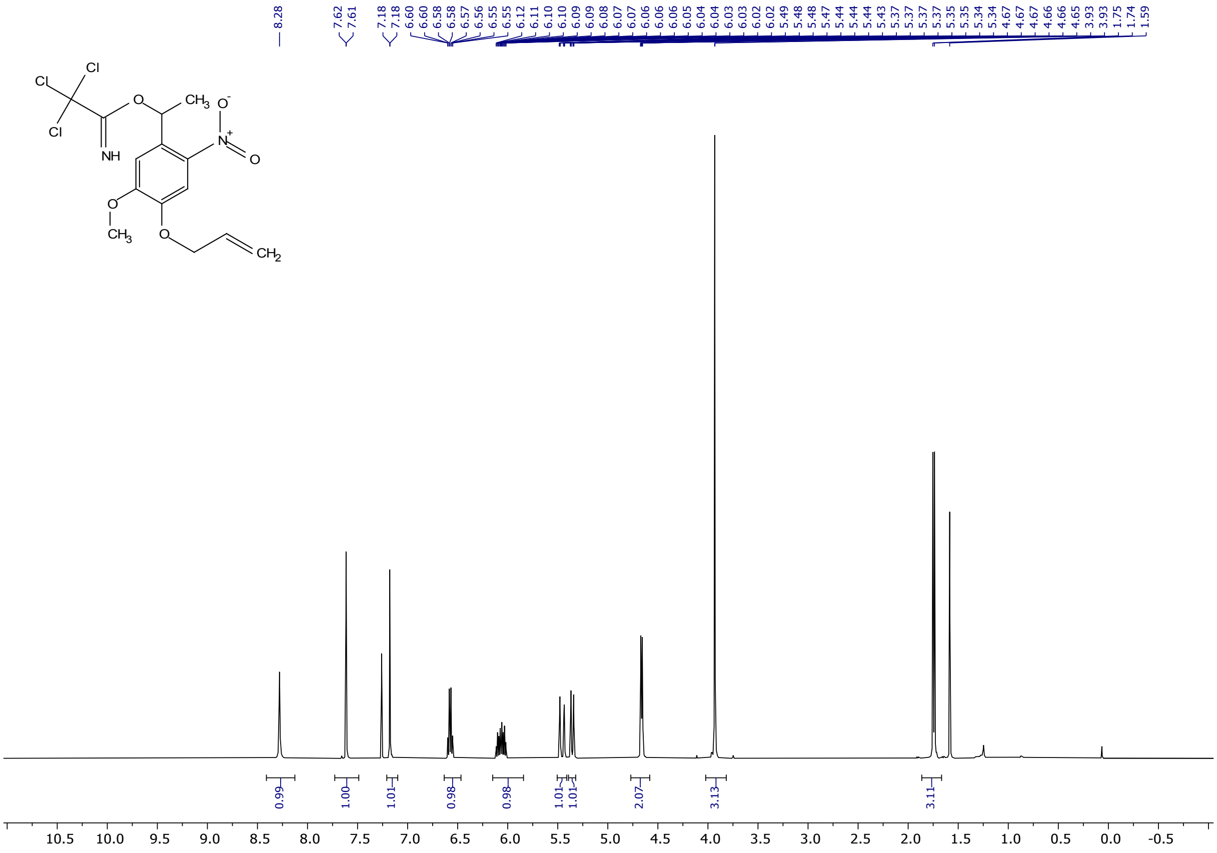
Cl
Cl
Cl
O
CH3
O-
N+
O
NH
O
CH3
O
CH2
8.28
7.62
7.61
7.18
7.18
6.60
6.58
6.58
6.57
6.56
6.55
6.55
6.12
6.11
6.10
6.10
6.09
6.08
6.07
6.06
6.06
6.06
6.05
6.04
6.04
6.03
6.03
6.02
6.02
5.49
5.48
5.48
5.47
5.44
5.44
5.44
5.43
5.37
5.37
5.37
5.37
5.35
5.35
5.34
5.34
4.67
4.67
4.67
4.66
4.66
4.65
3.93
3.93
1.75
1.74
1.59
0.99
1.00
1.01
0.98
0.98
1.01
1.01
2.07
3.13
3.11
10.5 10.0 9.5 9.0 8.5 8.0 7.5 7.0 6.5 6.0 5.5 5.0 4.5 4.0 3.5 3.0 2.5 2.0 1.5 1.0 0.5 0.0 -0.5

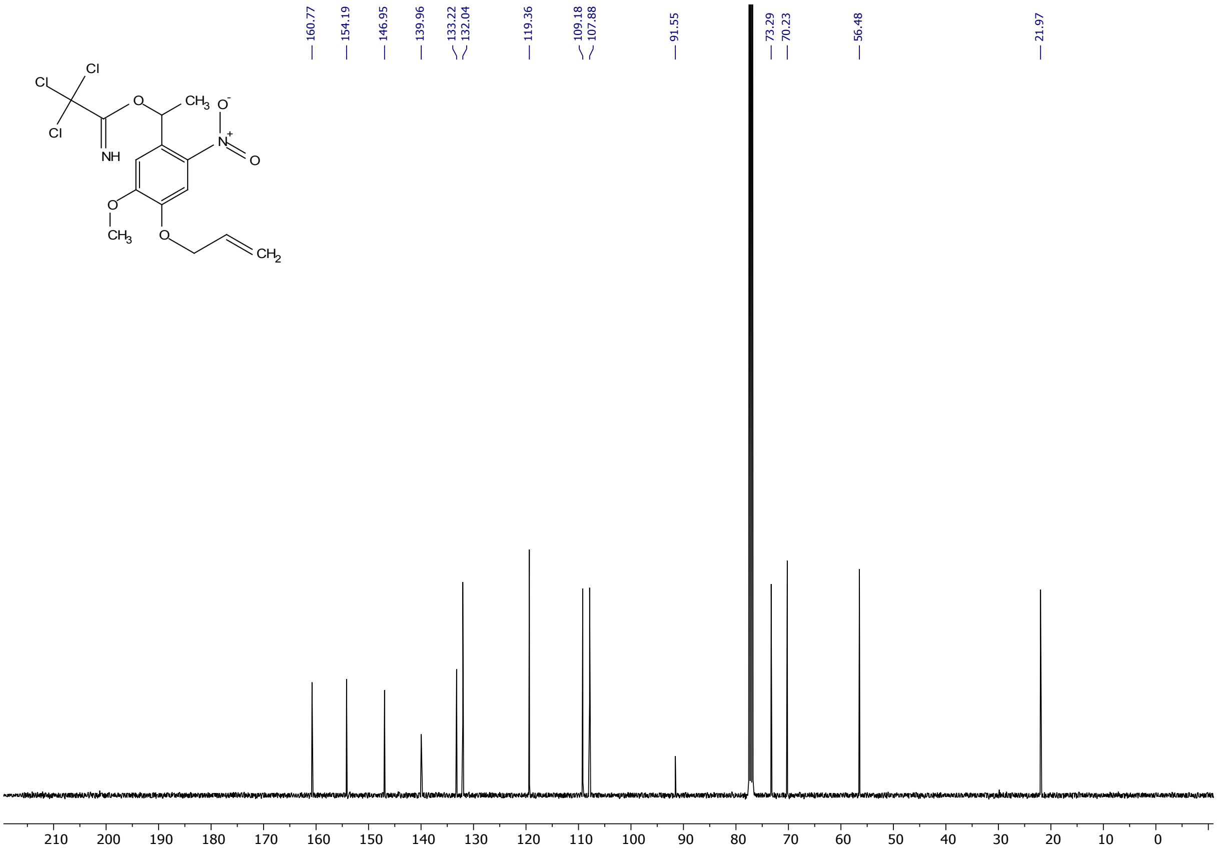
Cl
Cl
Cl
O
CH3
O-
N+
O
NH
O
CH3
O
CH2
160.77
154.19
146.95
139.96
133.22
132.04
119.36
109.18
107.88
91.55
73.29
70.23
56.48
21.97
210 200 190 180 170 160 150 140 130 120 110 100 90 80 70 60 50 40 30 20 10 0

1.1.2 On resin

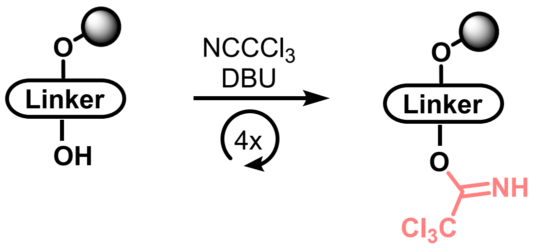

Merrifield resin functionalized with various photolabile linkers (see Section 2.3 Photolabile linkers) and of various initial loadings was placed in a 60 mL fritted syringe and attached to a 1 L screwcap bottle via a 3-way cap. The two other inlets were fitted to a vacuum line and an argon line. The syringe was wrapped in aluminum foil and direct light irradiation was prevented at all stages. The resin was washed with anhydrous CH_2Cl_2 five times. Then the resin was suspended equivalent to a 0.1 M solution in regards to the initial loading in anhydrous CH_2Cl_2. Five equivalents of trichloroacetonitrile were added and bubbling was adjusted to ensure sufficient mixing. Then, 0.1 equivalents of DBU were added dropwise and the reaction was bubbled for 1h. This process was repeated three times, washing with anhydrous CH_2Cl_2 after each coupling step. Finally, the resin was washed with CH_2Cl_2, MeOH, DMF and three times CH_2Cl_2. The solvent was removed under reduced pressure. The resin was stored under vacuum at RT under the exclusion of light. Alternatively, larger quantities were stored at - 20 °C under argon for later use.

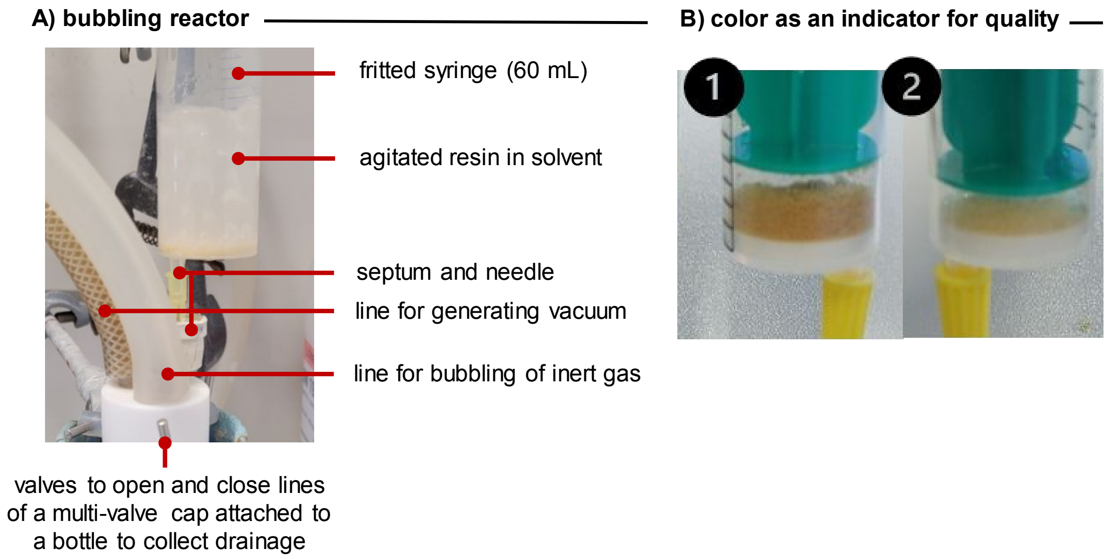

Figure 1: A) Self-built Argon bubbling reactor; B) the use of more than 0.1 eq. DBU or a high concentration of reagents causes lower quality resin. Darker color can be used as a rough indicator for quality ("1" darkened resin, "2" correctly colored TCAI-resin)

1.2 Optimization of TCAI-based coupling

1.2.1 In solution

Allyl protected TCAI traceless linker **SI2** was used for early in-solution development. It was found that TMSOTf at - 41 °C gave quantitative attachment of galactose BB **SI3** (Table 2, entry 7). The same conditions facilitated quantitative aglycon transfer from BB **SI5** to **SI2** (Table 2, entry 8).[2] This underpins the requirement of low-reactivity thioglycoside donors such as 2-(CF_3)Ph-thioglycoside BB **9** for a successful latent-active approach as described in this publication.

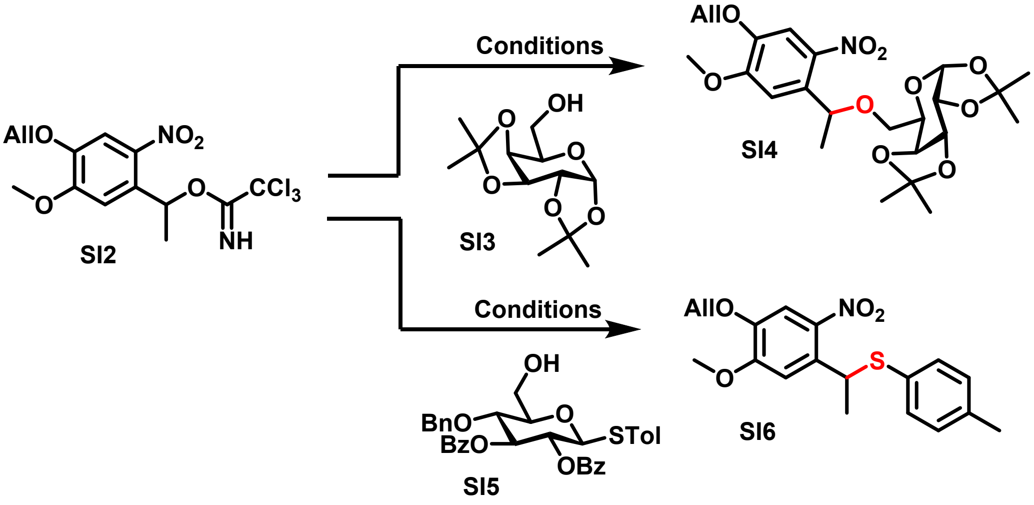

Table 2: In-solution optimization of the coupling of BB to linker.

No	Building Block	Promotor	Temperature / Time	Yield [%]
1	**SI3**	TMSOTf (0.1 eq.)	23 °C / 20 min	38
2	**SI3**	CSA	23 °C / 16 h	-
3	**SI3**	TfOH	- 41 °C / 60 min	35
4	**SI3** in THF	BF_3-Et_2O (0.5 eq.)	23 °C / 90 min	64
5	**SI3**	BF_3-Et_2O (1.5 eq.)	- 41 °C / 30 min	93
6	**SI3**	TMSOTf (0.1 eq.)	- 41 °C / 60 min	43
7	**SI3**	TMSOTf (1 eq.)	- 41 °C / 30 min → 0 °C / 30 min	quantitative
8	**SI5**	TMSOTf (1 eq.)	- 41 °C 30 min → 0 °C 30 min	aglycon transfer (quantitative)

Experiments were performed at a 0.04 to 0.2 mmol scale in 0.1 M dilution in CH_2Cl_2, using an access of three equivalents of alcohol.

Reacting one equiv. of **SI2** with ethylene glycol probe **8** in solution gave 86% yield (Table 3), validating EG-probe **8** for the quantification of TCAI-based resin loading Table 1.

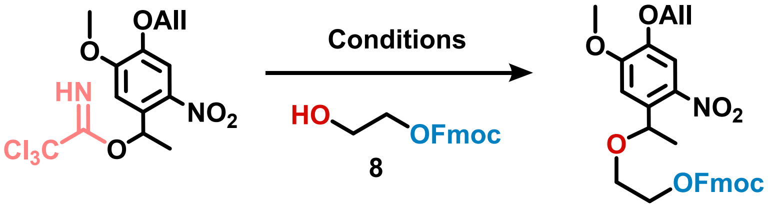

Table 3: In-solution attachment of Fmoc-ethylene glycol linker **2** to linker **SI2**.

No	Promotor	Building Block	T [°C]	Yield [%]
1	TMSOTf (1 eq.)	1 equiv.	- 40°C (30 min) → -20°C (30 min)	86

Experiment was performed at 0.1 M in CH_2Cl_2 at 0.2 mmol scale.

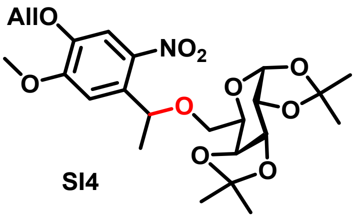

1,2,3,4-Diacetonide-6-((5-methoxy-2-nitro-4-(prop-1-en-1-yl)oxy)phenyl)ethoxy)methyl) galactopyranoside (**SI4**)

^{1}H NMR (400 MHz, $CDCl_3$) δ 7.60 (d, J = 7.0 Hz, 1H), 7.39 – 7.21 (m, 1H), 6.07 (ddt, J = 17.2, 10.6, 5.5 Hz, 1H), 5.53 – 5.41 (m, 2H), 5.35 (dq, J = 10.5, 1.4 Hz, 1H), 5.23 (dq, J = 7.9, 6.2 Hz, 1H), 4.66 (dt, J = 5.6, 1.6 Hz, 2H), 4.60 (ddd, J = 7.8, 6.7, 2.4 Hz, 1H), 4.33 – 4.18 (m, 2H), 3.98 (d, J = 2.6 Hz, 3H), 3.59 – 3.34 (m, 2H), 1.54 (d, J = 9.5 Hz, 3H), 1.50 (dd, J = 6.3, 4.2 Hz, 3H), 1.36 (d, J = 11.1 Hz, 3H), 1.33 (s, 3H), 1.31 (d, J = 5.1 Hz, 3H).

^{13}C NMR (101 MHz, $CDCl_3$) δ 154.5, 154.4, 146.6, 146.5, 140.2, 140.1, 136.2, 135.9, 132.3, 132.2, 119.2, 119.2, 109.5, 109.5, 109.4, 109.4, 109.3, 109.0, 108.7, 96.5, 74.4, 74.0, 71.5, 71.3, 70.8, 70.7, 70.7, 70.6, 70.2, 70.2, 68.4, 67.8, 67.4, 66.6, 56.5, 56.5, 26.2, 26.2, 26.1, 26.1, 25.1, 25.0, 24.7, 24.5, 23.8, 23.7.

HRMS (ESI): $C_{24}H_{33}NO_{10}Na$ $[M+Na]^+$; calculated: 518.2002, found: 518.2000.

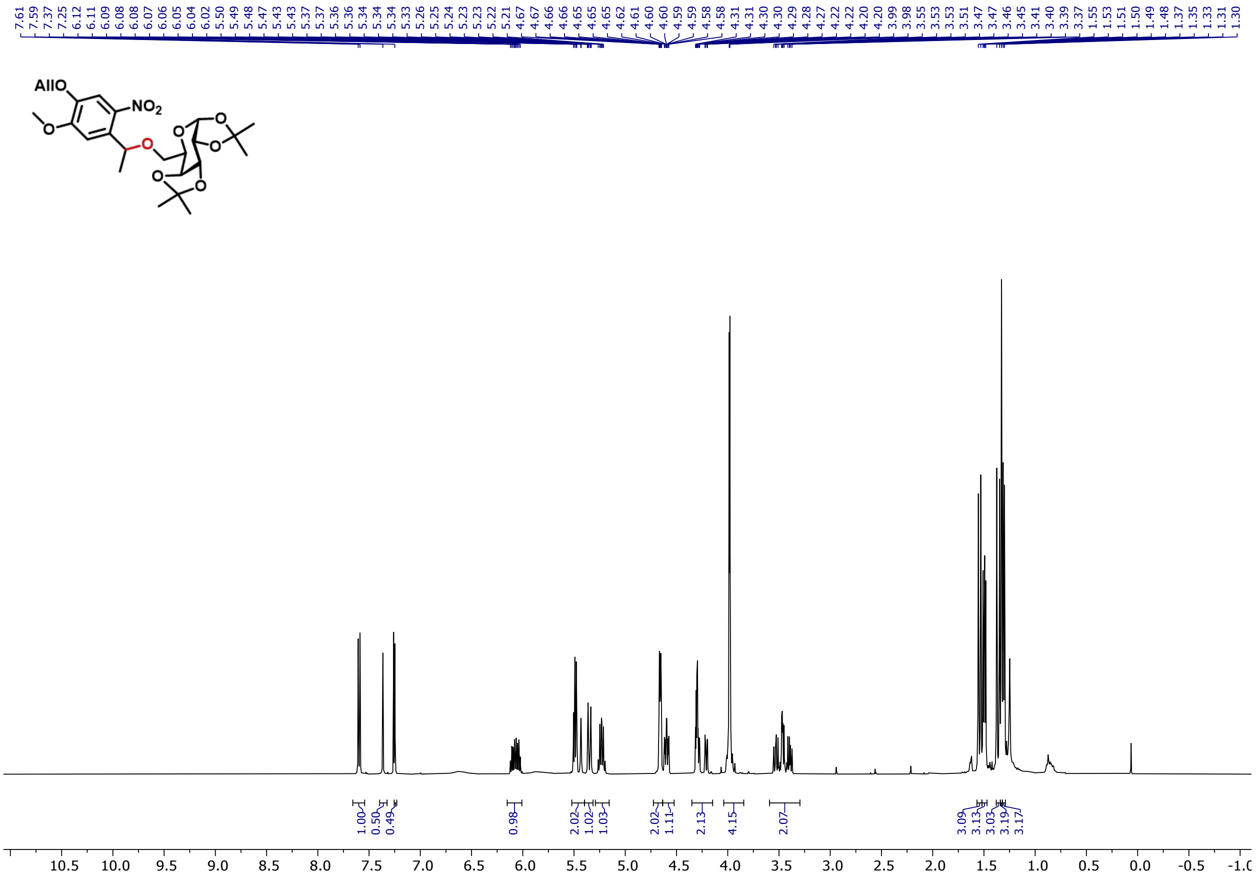
7.61 7.59 7.37 7.25 6.12 6.11 6.09 6.08 6.08 6.07 6.06 6.05 6.04 6.02 5.50 5.49 5.48 5.47 5.43 5.43 5.37 5.37 5.36 5.36 5.34 5.34 5.34 5.33 5.26 5.25 5.24 5.23 5.23 5.22 5.21 4.67 4.67 4.66 4.65 4.65 4.62 4.61 4.60 4.60 4.59 4.59 4.58 4.58 4.31 4.31 4.30 4.29 4.28 4.27 4.22 4.22 4.20 4.20 3.99 3.98 3.55 3.53 3.53 3.51 3.47 3.47 3.46 3.45 3.41 3.40 3.39 3.37 1.55 1.53 1.51 1.50 1.49 1.48 1.37 1.35 1.33 1.31 1.30
AllO
NO2
1.00 0.50 0.49 0.98 2.02 1.02 1.03 2.02 1.11 2.13 4.15 2.07 3.09 3.13 3.03 3.19 3.17
10.5 10.0 9.5 9.0 8.5 8.0 7.5 7.0 6.5 6.0 5.5 5.0 4.5 4.0 3.5 3.0 2.5 2.0 1.5 1.0 0.5 0.0 -0.5

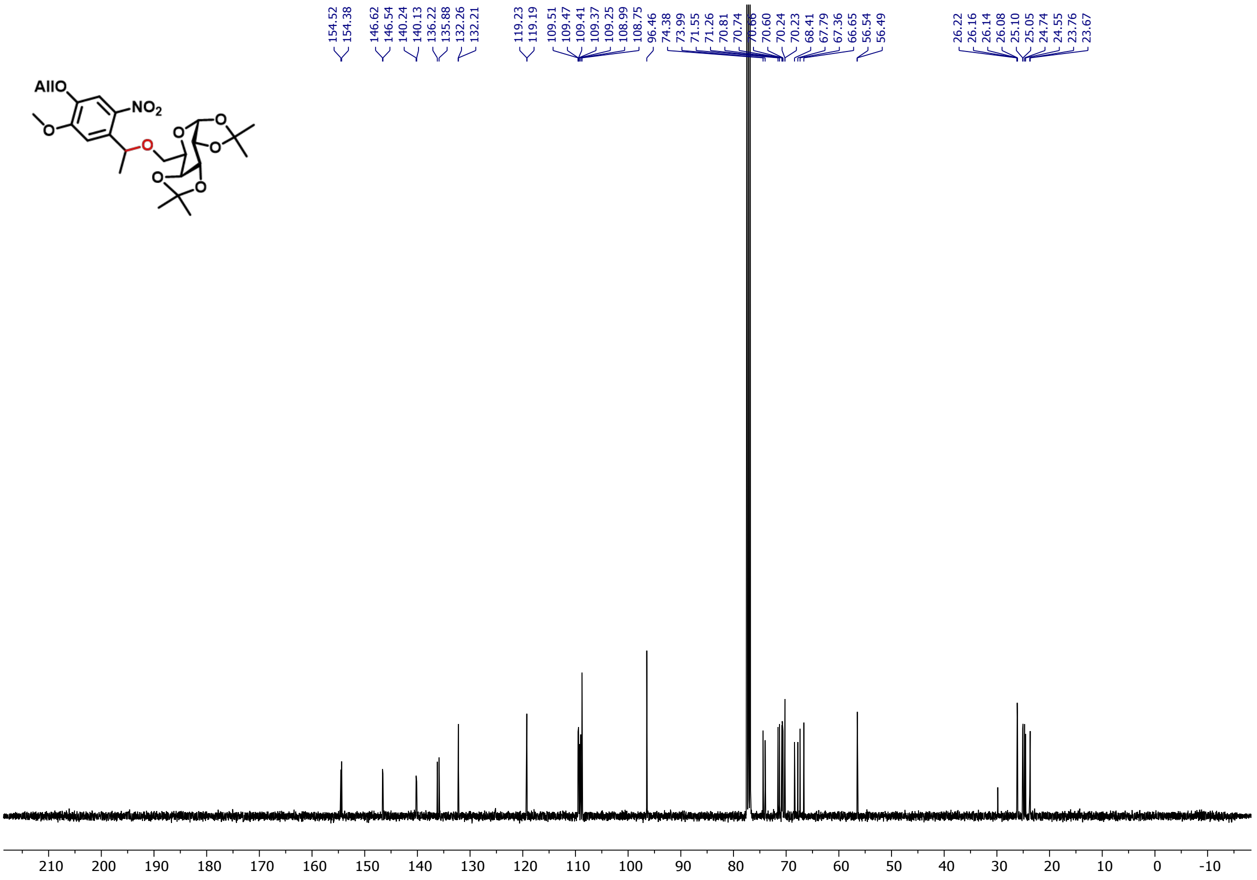
154.52 154.38 146.62 146.54 140.24 140.13 136.22 135.88 132.26 132.21 119.23 119.19 109.51 109.47 109.41 109.37 109.25 108.99 108.75 96.46 74.38 73.99 71.55 71.26 70.81 70.74 70.66 70.60 70.24 70.23 68.41 67.79 67.36 66.65 56.54 56.49 26.22 26.16 26.14 26.08 25.10 25.05 24.74 24.55 23.76 23.67
AllO
NO2
210 200 190 180 170 160 150 140 130 120 110 100 90 80 70 60 50 40 30 20 10 0 -10

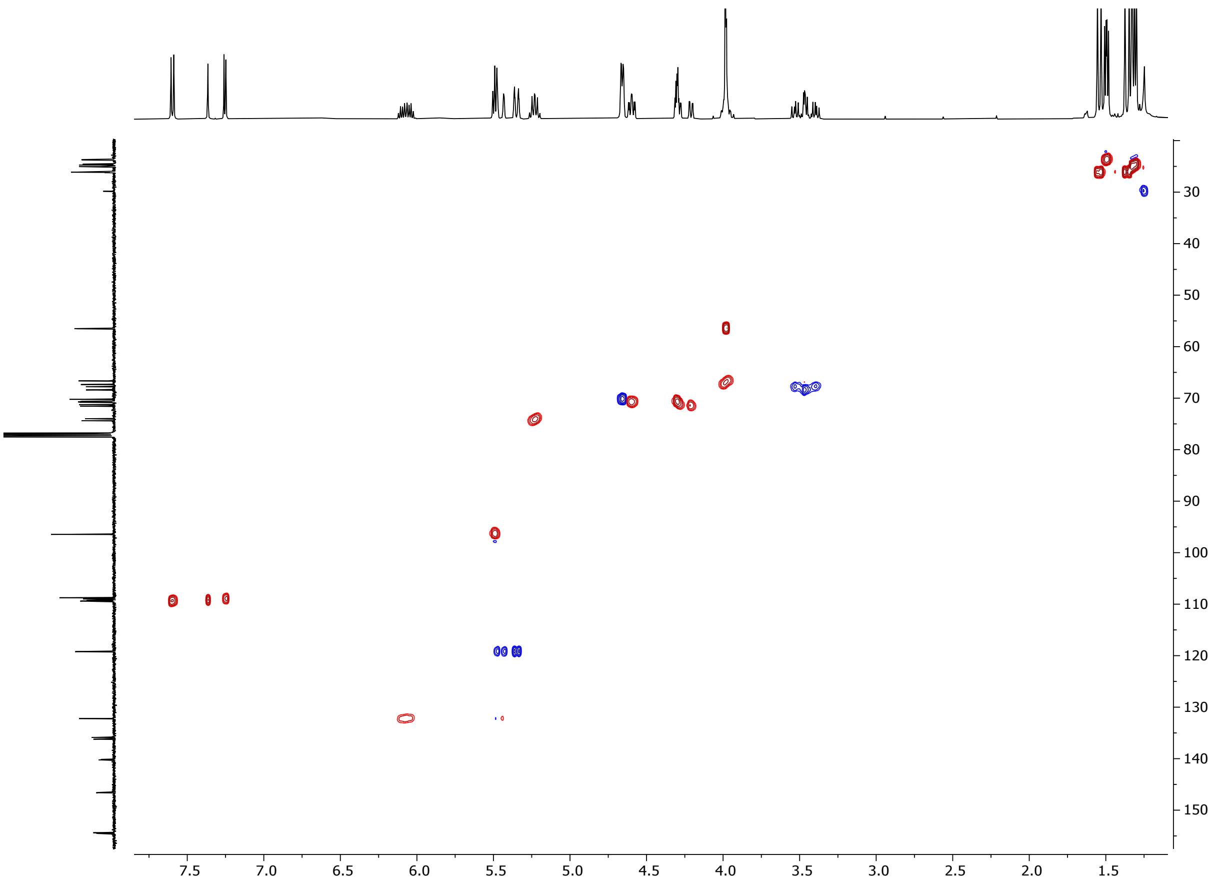

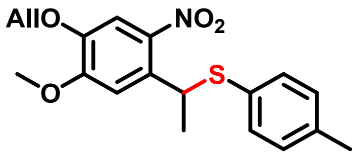

(1-(5-Methoxy-2-nitro-4-(prop-1-en-1-yloxy)phenyl)ethyl)(p-tolyl)sulfane (**SI6**)

^{1}H NMR (400 MHz, $CDCl_3$) *δ* 7.41 (s, 1H), 7.17 (s, 1H), 7.16 – 7.10 (m, 2H), 7.04 – 6.98 (m, 2H), 6.05 (ddt, *J* = 17.2, 10.7, 5.5 Hz, 1H), 5.47 – 5.39 (m, 1H), 5.34 (dt, *J* = 10.5, 1.2 Hz, 1H), 5.26 (q, *J* = 6.9 Hz, 1H), 4.68 – 4.55 (m, 2H), 3.92 (s, 3H), 2.27 (s, 3H), 1.63 (d, *J* = 6.9 Hz, 3H).

^{13}C NMR (101 MHz, $CDCl_3$) δ 153.5, 146.4, 141.0, 137.9, 133.8, 133.0, 132.1, 130.3, 129.8, 119.2, 110.7, 109.3, 70.2, 56.4, 42.6, 22.5, 21.2.

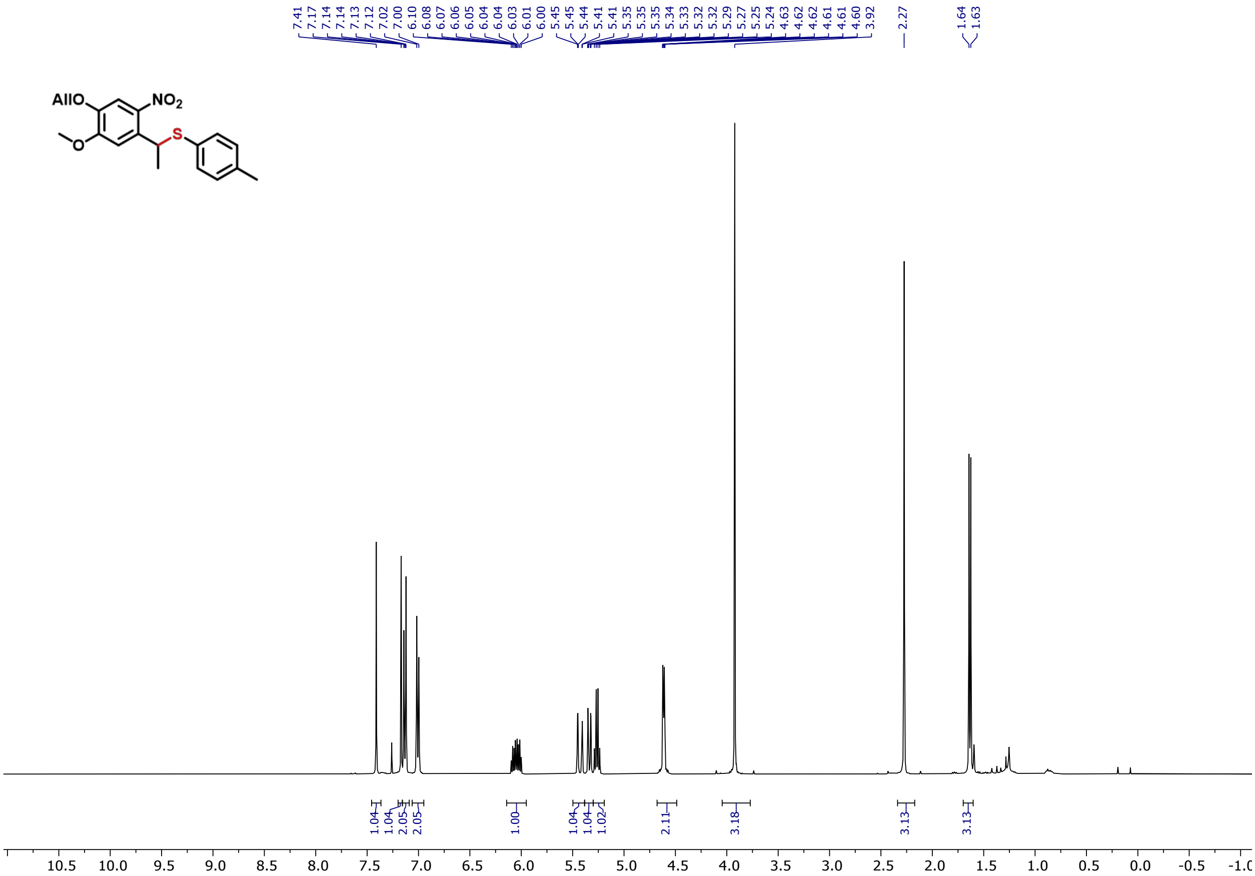
7.41 7.17 7.14 7.14 7.13 7.12 7.02 7.00 6.10 6.08 6.07 6.06 6.05 6.04 6.04 6.03 6.01 6.00 5.45 5.45 5.44 5.41 5.41 5.35 5.35 5.35 5.34 5.33 5.32 5.32 5.29 5.27 5.25 5.24 4.63 4.62 4.62 4.61 4.61 4.60 3.92
2.27
1.64 1.63
AllO
NO2
O
S
1.04 1.04 2.05 2.05 1.00 1.04 1.04 1.02 2.11 3.18 3.13 3.13
10.5 10.0 9.5 9.0 8.5 8.0 7.5 7.0 6.5 6.0 5.5 5.0 4.5 4.0 3.5 3.0 2.5 2.0 1.5 1.0 0.5 0.0 -0.5

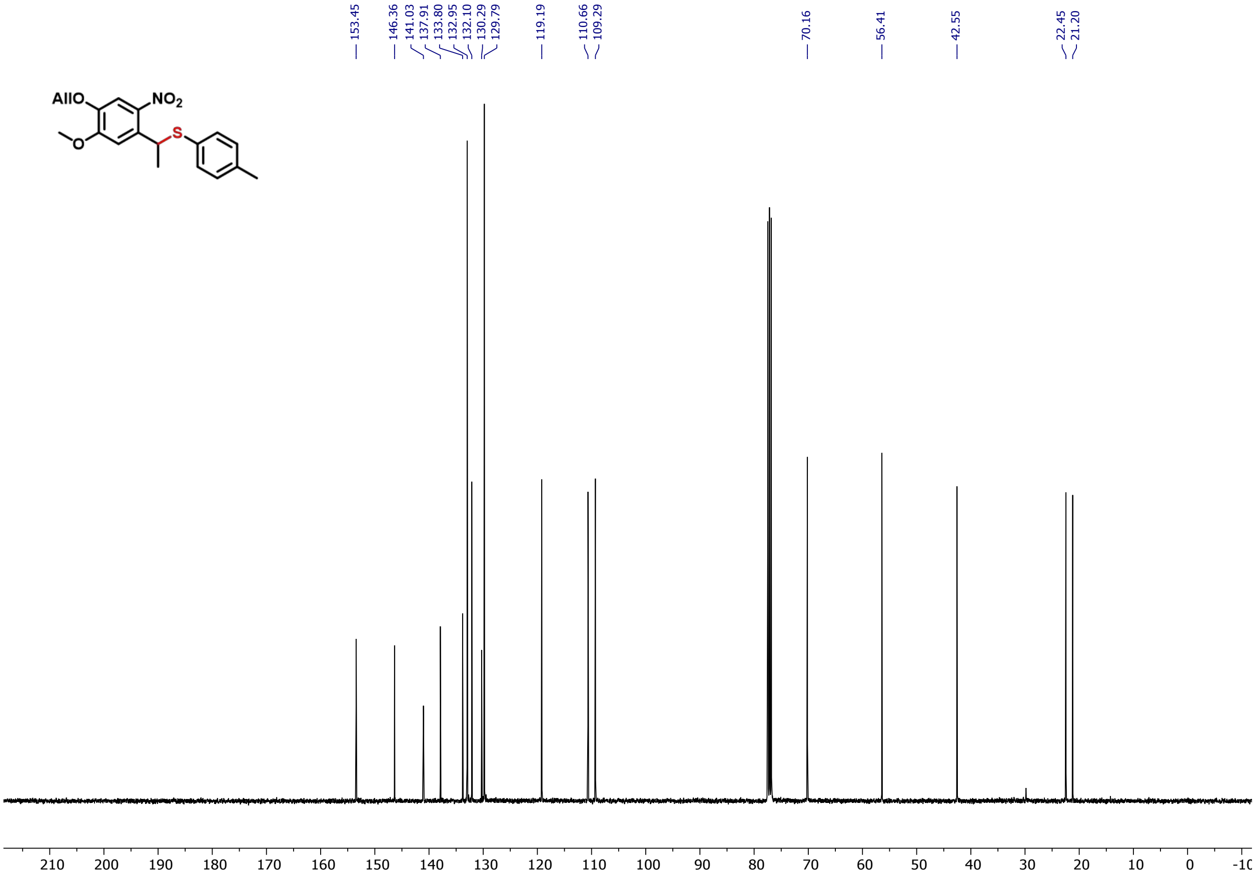
153.45
146.36 141.03 137.91 133.80 132.95 132.10 130.29 129.79
119.19
110.66 109.29
70.16
56.41
42.55
22.45 21.20
AllO
NO2
O
S
210 200 190 180 170 160 150 140 130 120 110 100 90 80 70 60 50 40 30 20 10 0

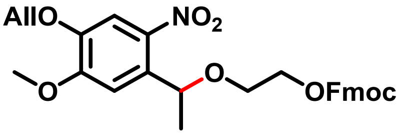

(9H-Fluoren-9-yl)methyl (2-(1-(5-methoxy-2-nitro-4-(prop-1-en-1-yloxy)phenyl)ethoxy)ethyl) carbonate (**SI7**)

^{1}H NMR (400 MHz, $CDCl_3$) δ 7.65 (d, J = 7.5 Hz, 2H), 7.54 – 7.44 (m, 3H), 7.32 – 7.26 (m, 2H), 7.24 – 7.11 (m, 3H), 5.95 (ddt, J = 17.3, 10.7, 5.5 Hz, 1H), 5.34 (dq, J = 17.2, 1.5 Hz, 1H), 5.23 (dq, J = 10.5, 1.3 Hz, 1H), 5.10 (q, J = 6.2 Hz, 1H), 4.53 (dt, J = 5.5, 1.5 Hz, 2H), 4.39 – 4.27 (m, 2H), 4.23 (ddd, J = 11.9, 7.6, 2.8 Hz, 1H), 4.18 – 4.07 (m, 2H), 3.77 (s, 3H), 3.47 (ddd, J = 11.1, 4.7, 2.9 Hz, 1H), 3.39 (ddd, J = 10.9, 7.6, 2.9 Hz, 1H), 1.41 (d, J = 6.3 Hz, 3H).

^{13}C NMR (101 MHz, $CDCl_3$) δ 155.3, 154.6, 146.7, 143.4, 143.3, 141.4, 140.1, 135.5, 132.2, 128.0, 127.3, 127.3, 125.1, 125.1, 120.2, 119.2, 109.4, 108.6, 74.1, 70.2, 69.8, 67.3, 66.8, 56.5, 46.9, 23.7.

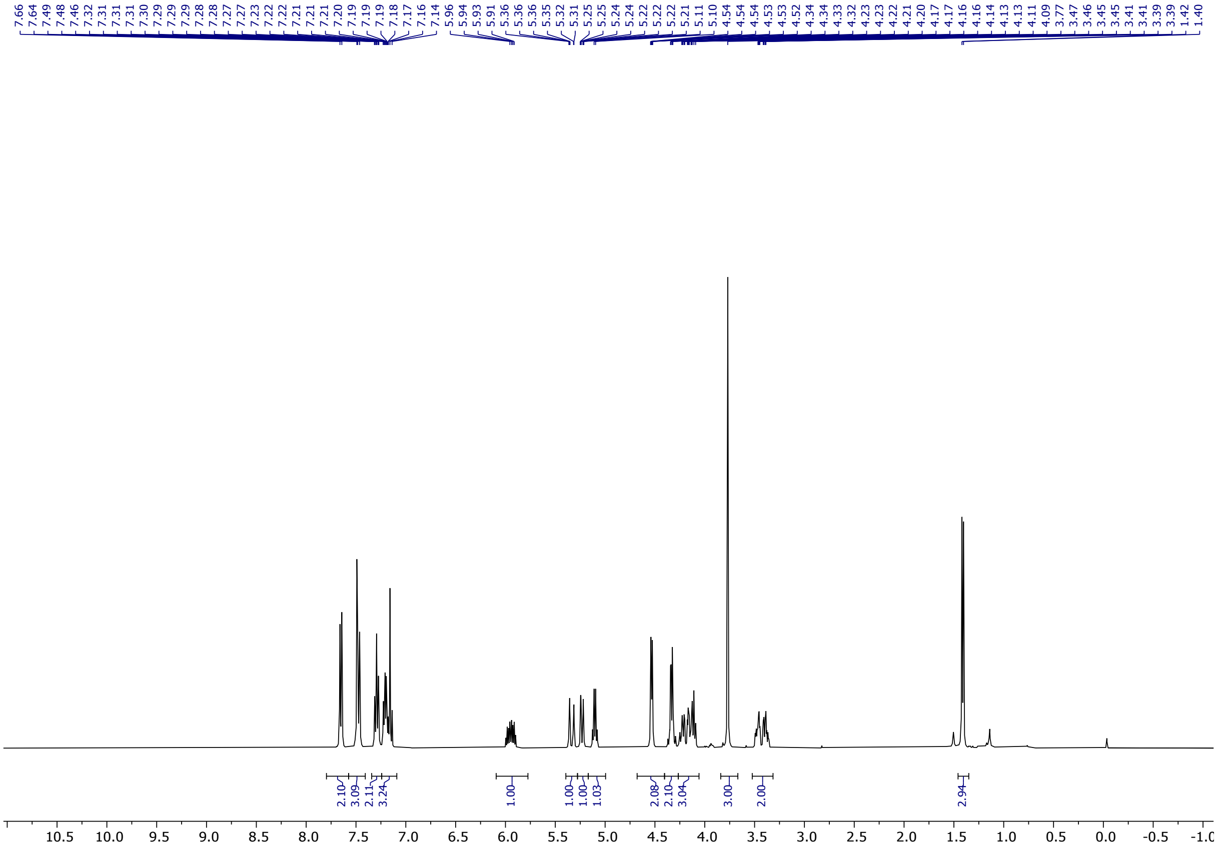

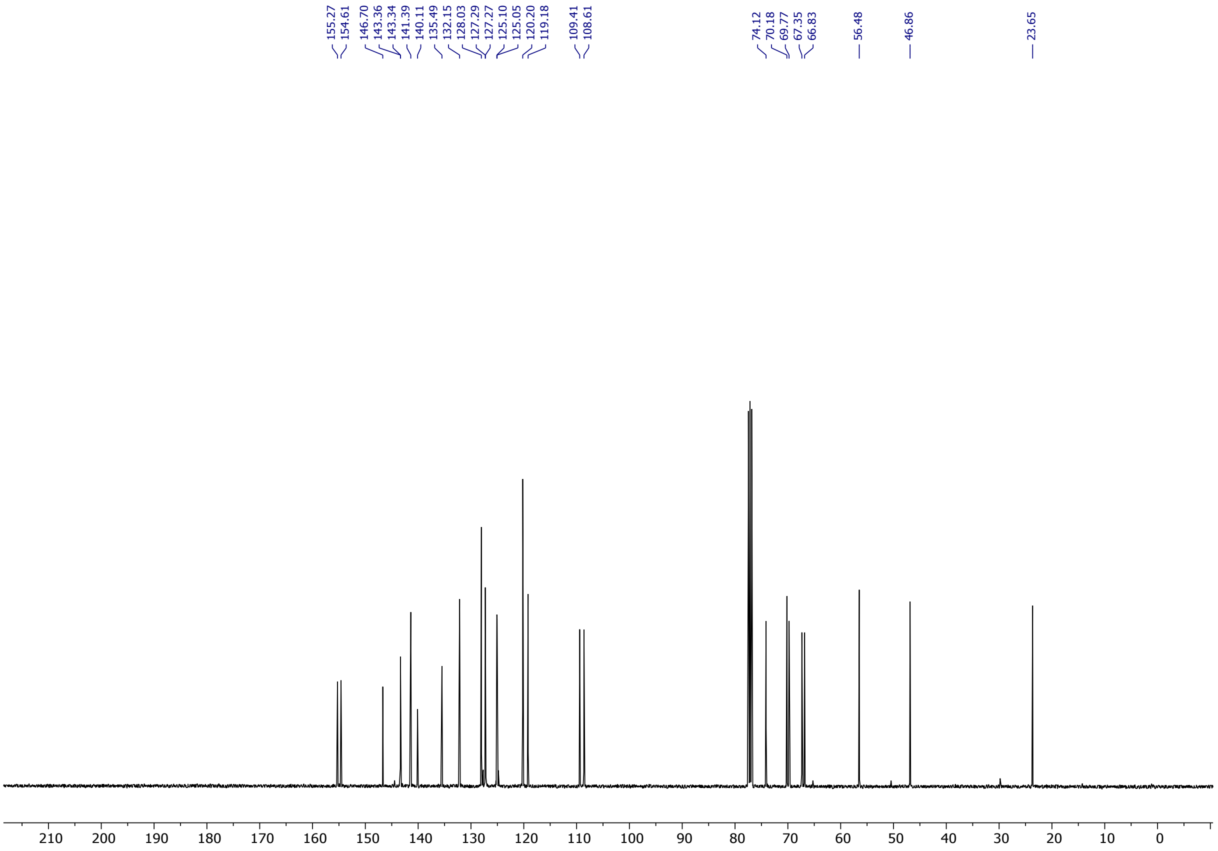
155.27
154.61
146.70
143.36
143.34
141.39
140.11
135.49
132.15
128.03
127.29
127.27
125.10
125.05
120.20
119.18
109.41
108.61
74.12
70.18
69.77
67.35
66.83
56.48
46.86
23.65

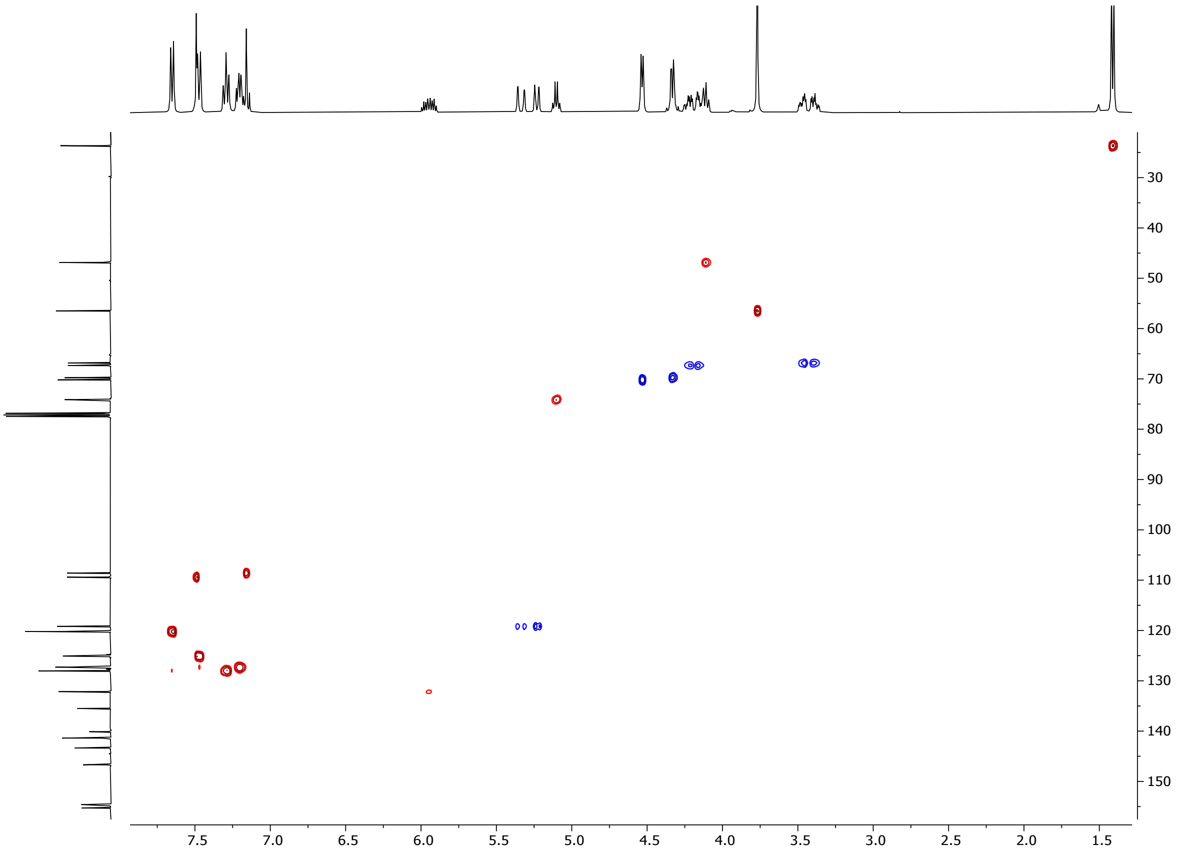

1.2.2 On resin

Optimization of promotor, coupling time, building block and temperature

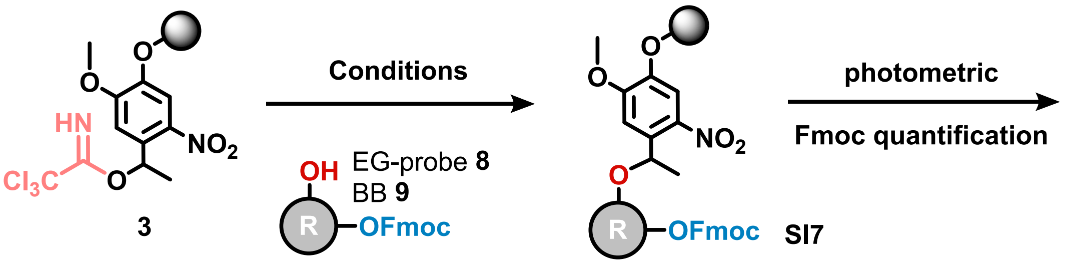

Table 4: Activator screen for automated attachment of **6** to TCAI-Traceless resin **4**.

No	Promotor	T1 → T2 [°C]	Loading [mmol/g]
1	TMSOTf (1 eq.)	-40 (5 min) → -40 (35 min)	0.179
2	TMSOTf (1 eq.)	-40 (5 min) → -20 (35 min)	0.280
3	TMSOTf (1 eq.)	-40 (5 min) → -0 (35 min)	0.251
4	TMSOTf (0.5 eq.)	-40 (5 min) → -20 (35 min)	0.178
5	TMSOTf (2 eq.)	-40 (5 min) → -20 (35 min)	0.334 (77%)
6	TMSOTf (4.5 eq.)	-40 (5 min) → -40 (35 min)	0.237
7	BF_3-Et_2O (4 eq.)	-40 (5 min) → -20 (35 min)	0.359 (83%)

Both reactions were performed on a resin with an initial loading of 0.43 mmol/g using 10 eq. Fmoc-EG probe in 1 mL CH_2Cl_2.

As BF_3-OEt_2 and TMSOTf tested in solution (Table 2) and on resin (Table 4) had similar yields, TMSOTf was prioritized because it is already a standard reagent in AGA.

Optimization of trifunctional BB 9 attachment – temperature regime and BB concentration/equivalents

Table 5: Automated on-resin optimization of attachment of BB **9** to TCAI-Traceless resin.

**No**	**Building Block**	**T** [°C] / **Time**[min]	**Loading** [mmol/g]
1	5 eq. in 2 mL CH_2Cl_2	- 41 (30, in flask)	0.098
2	5 eq. in 2 mL CH_2Cl_2	- 40°C (20 min) → -20°C (10)	0.1
3	6 eq. in 1 mL CH_2Cl_2	- 40°C (5 min) → 0°C (20)	0.124
4	12 eq. in 2 mL CH_2Cl_2	- 40°C (5 min) → -20°C (20)	0.231
5	6 eq. in 1 mL CH_2Cl_2	- 40°C (5 min) → -20°C (35)	0.221

All reactions were performed on a resin with an initial loading of 0.4 mmol/g and using 2 eq. of TMSOTf in 1 mL CH_2Cl_2 as the activator and trifunctional BB **9** (mixture of anomers) as coupling partner.

Entry 5 was set as the default as less building block was needed.

Table 6: Loading control via photometric Fmoc-quantification of imidate resins using Fmoc ethylene glycol probe **8**.

**No**	**Resin**	**Initial Loading** [mmol/g]	**EG-Probe loading** [mmol/g]
1	TFPAI-Traceless (**6**)	0.40	0.22
2	TCAI-Para Nitro (**4**)	0.36	0.08
3	TCAI-Vanillin (**5**)	0.20	0.10
4	TCAI-Traceless (**3**)	0.43	0.33

All reactions were performed using a temperature protocol (- 40°C (5 min) → - 20°C (35 min)), 2 eq. of TMSOTf in 1 mL CH_2Cl_2 as the activator and 10 equiv. EG-probe **8** as coupling partner.

α- and *β*-anomer attached equally well to the TCAI-traceless resin and reacted equally in further reactions. Using a 1:1 mixture of anomers of BB **9** led to a 1:1 mixture of thioglycoside anomers on model trimer **SI8**. Using this type of “mixed” donors in glycosylations of the reducing end resulted in the exclusive formation of *α*-glycosides.

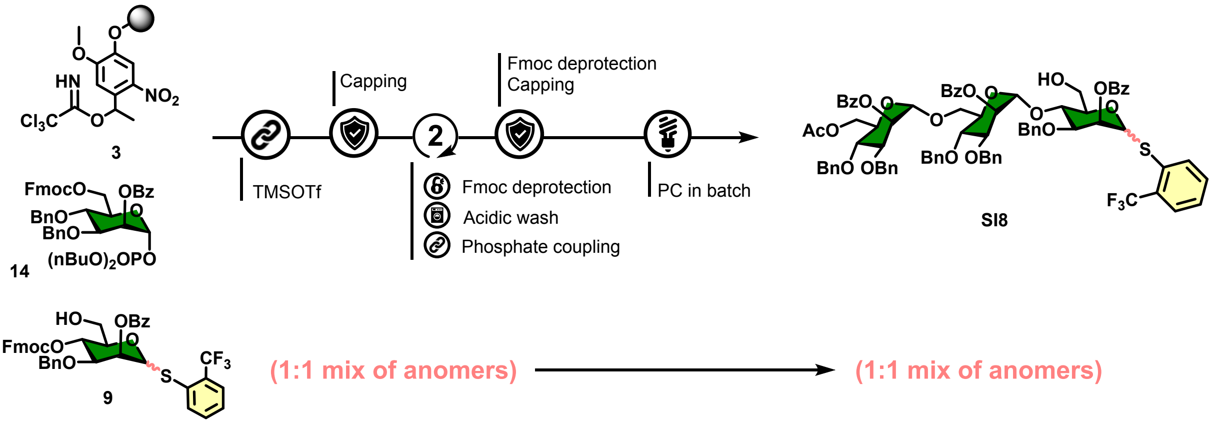

Figure 2: A synthesis from a 1:1 mixture of anomers of BB **9** results in a 1:1 mixture of anomers of model trimer **SI8**.

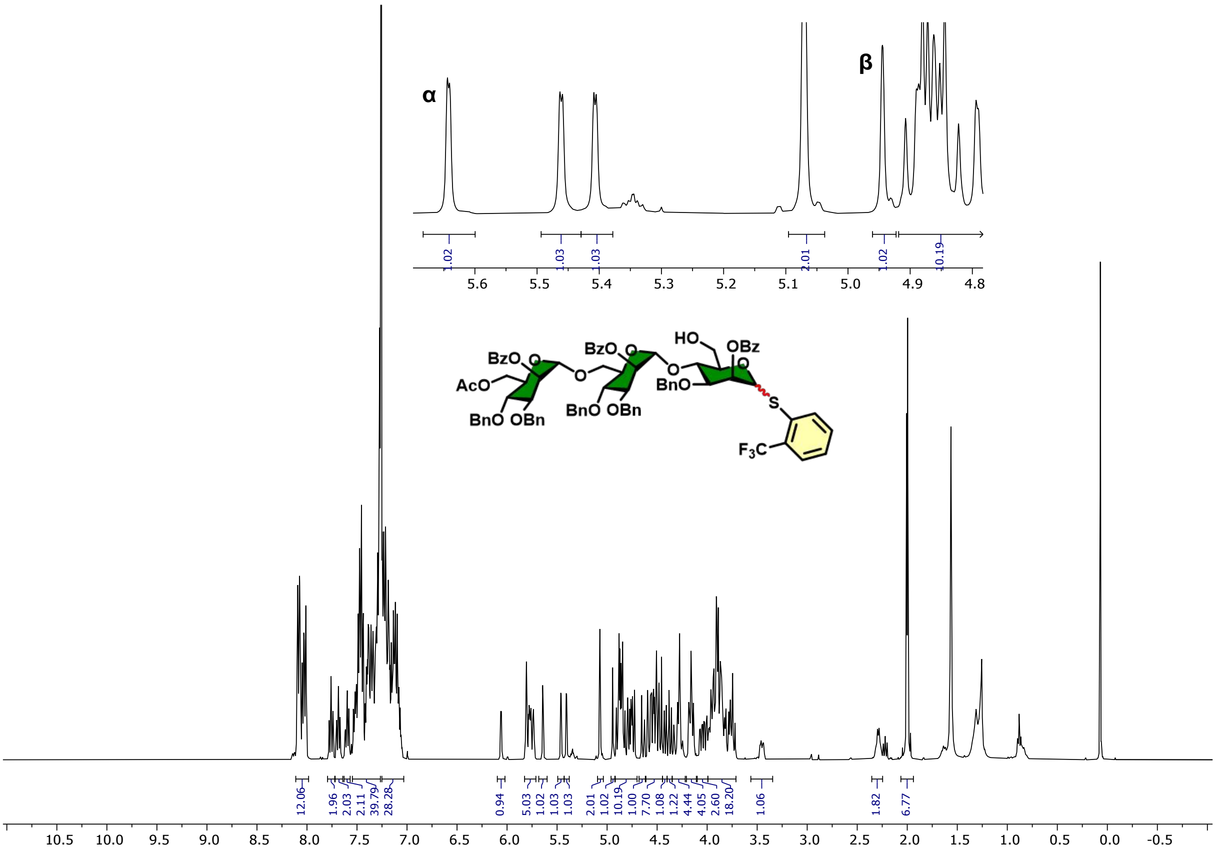

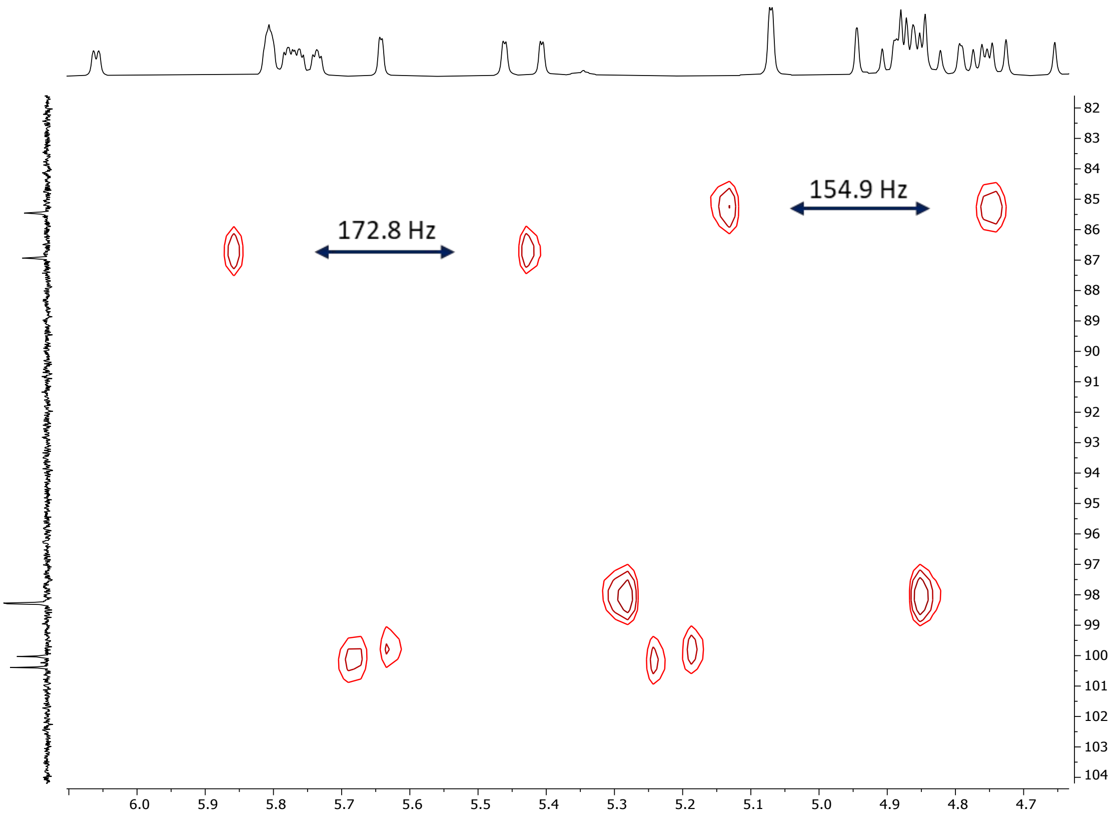

172.8 Hz
154.9 Hz

1.3 Optimization of photocleavage conditions

1.3.1 In solution

To achieve optimal cleavage of the ether bond of the glycan to the stationary phase, the reaction was first tested on **SI4**. Initial tests (Table 7) were performed using a previously described flowreactor.[3]

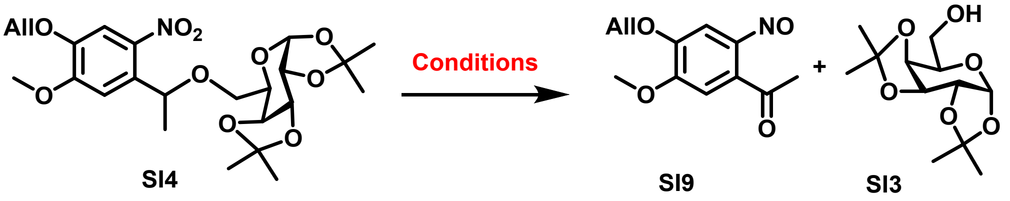

Table 7: In-solution optimization of photocleavage of galactose BB **SI3** from linker adduct **SI4** using a flowreactor setup.

**No**	**Solvent**	**Flowrate** [mL/min]	**Yield** [%]
1*	CH_2Cl_2/EtOH (2 mL, 1:1)	Batch, 16 h	38
2	CH_2Cl_2 (3 mL)	0.4	48
3	CH_2Cl_2 (3 mL)	0.8	71
4	MeCN (3 mL)	0.4	39
5	EtOH (3 mL)	0.4	69
6	CH_2Cl_2 (1 mL)	0.8	60
7	CH_2Cl_2 (3 mL)	1.5	53
8	CH_2Cl_2 (1 mL)	1.5	54
9	CH_2Cl_2/EtOH+H_2O (3 mL, 1:1)	0.8	53

All experiments were performed at 0.1 ± 0.02 mmol scale at 20 °C in a flow reactor [3] and an irradiation at 365 nm. *Performed at 366 nm under TCL-benchtop lamp in a sealed tube stirred at 400 rpm in 0.01 M dilution reaching temperatures of up to 40 °C.

1.3.2 On resin

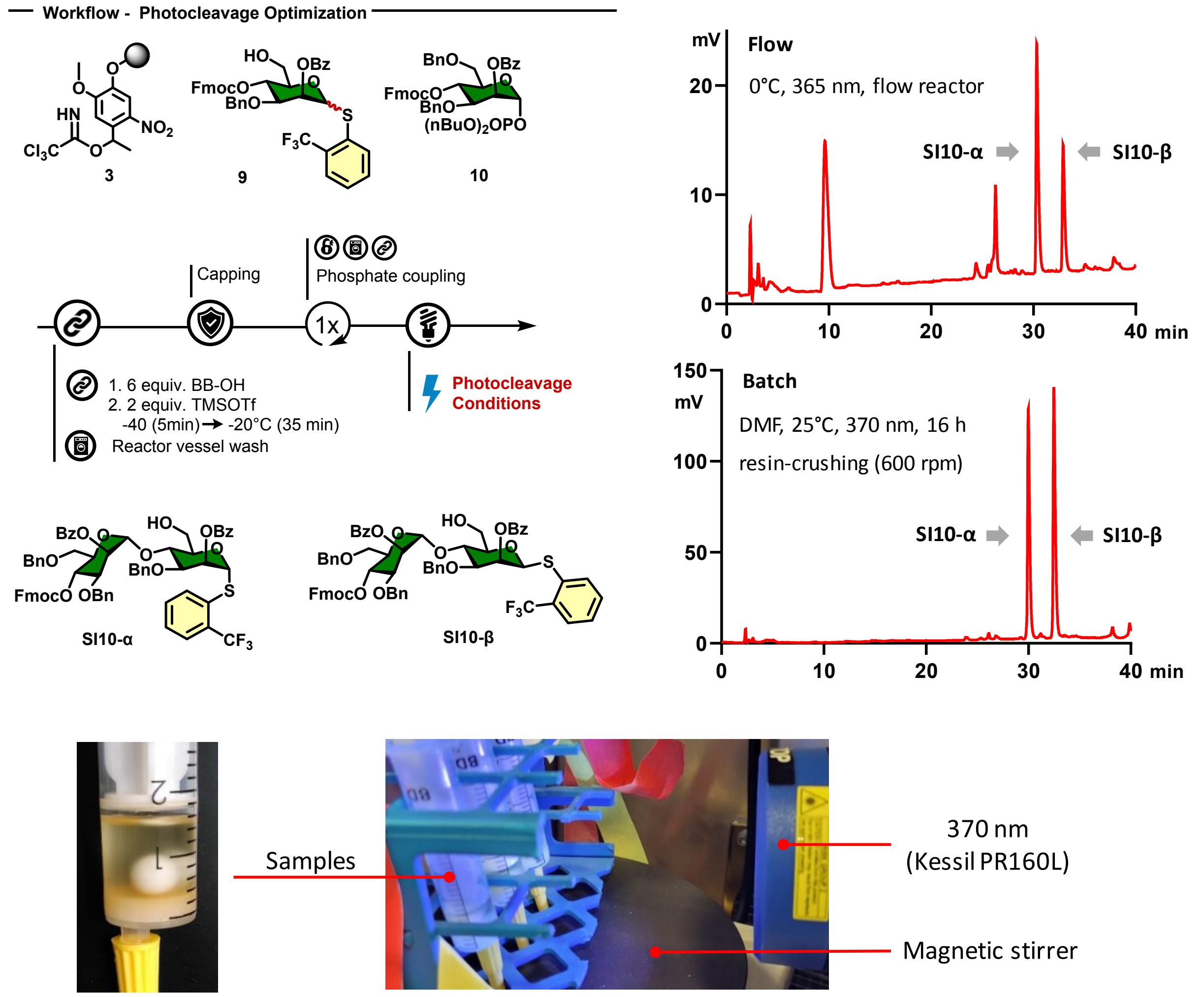

Figure 3: Photocleavage was optimized for purity and yield. Top left: experimental workflow; bottom left: experimental setup of batch photocleavage; right side: representative analytical HPLC traces of crude products after cleavage.

The flow reactor-based method, preferred for carbamate or acetal cleavage, proved to be unsatisfactory in regards to crude yield and purity (Figure 3, top right graph).[3–5] Thus, a batch protocol was developed. The setup can be seen on the bottom of Figure 3. This setup not only allowed for elongated irradiation, but also for resin crushing via a stirring bar. Resin crushing has been shown to boost cleavage yields[6] and is especially beneficial when the solid support became darkened during on-resin glycosylations. Exact conditions for batch photocleavage, can be found in supporting information chapter 3.2 Photocleavage.

1.4 Methanol as quenching agent

MeOH was selected for a nucleophilic quench of putative leftover reactive species after the initial coupling of a building block to the TCAI-traceless resin. This resulted in dramatically decreased levels of deletion sequence **12** (Figure 4). However, no complete disappearance like in case of triethylsilane was achieved.

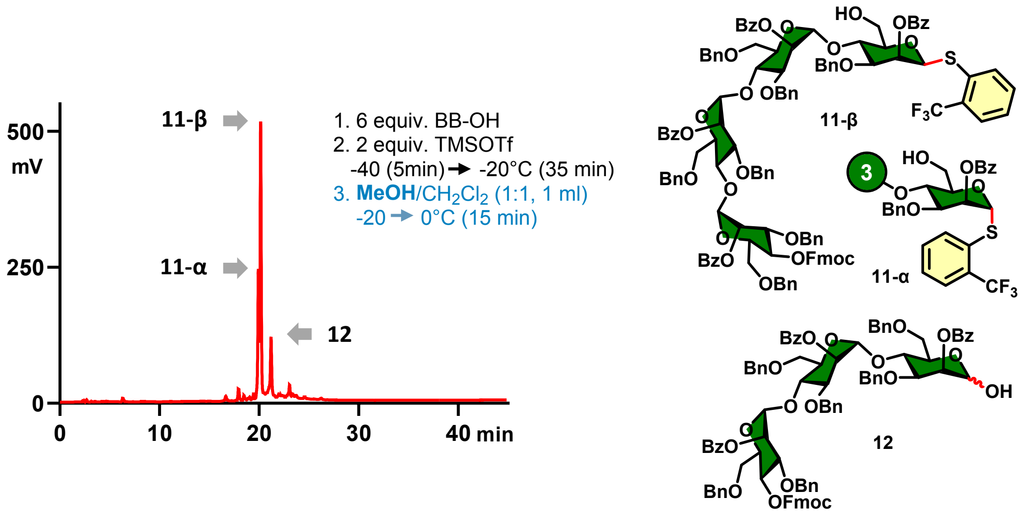

Figure 4: HPLC-trace of methanol as a quenching reagent. Building block **9** was employed as a mixture of roughly 1:2 of *α*/*β*-anomers. The peak corresponding to deletion sequence **12** was lower than without a quench but higher than under TES-quenching conditions (Figure 1D main text).

2 Synthesis

2.1 Monosaccharide building blocks

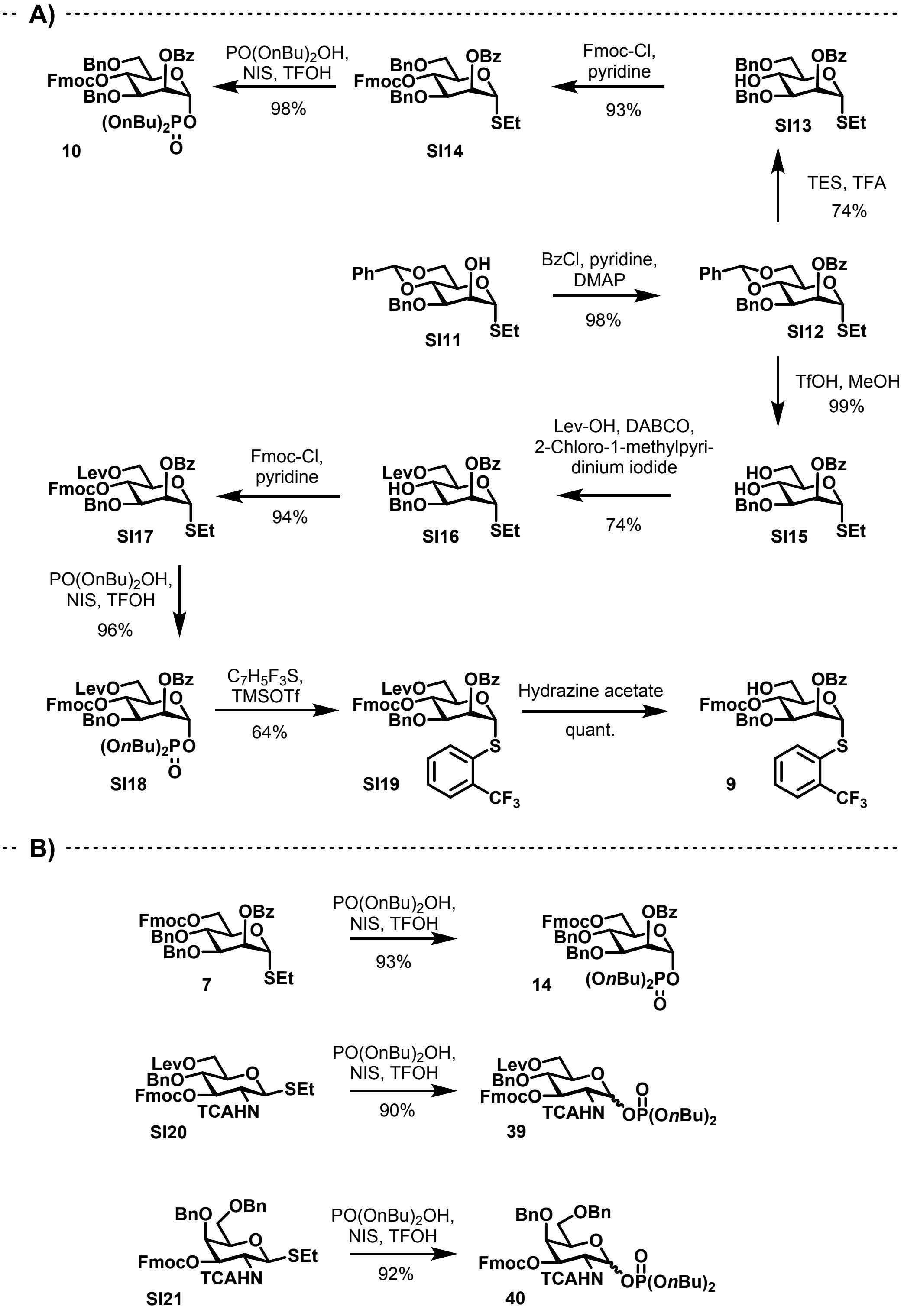

Scheme 1: Overview of the synthesis of monosaccharide building blocks. A) 4-Fmoc-Mannose BBs; B) Phosphate BBs from commercial precursors.

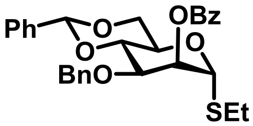

Ethyl 2-*O*-benzoyl-3-*O*-benzyl-4,6-*O*-benzylidene-1-thio-*α*-D-mannopyranoside (S12)

DMAP (2.09 g, 17.1 mmol, 0.3 eq.) was added to ethyl 3-O-benzyl-4,6-O-benzylidene-1-thio-*α*-D-mannopyranoside **SI11** (23 g, 57.1 mmol, 1 eq.) in 120 mL anhydrous pyridine under nitrogen. BzCl (19.9 mL, 171 mmol, 3 eq.) was added at 0 °C and stirred at that temperature for additional 30 min. The reaction was stirred warmed to 23 °C for 60 min. Access BzCl was destroyed by the addition of *i*PrOH (50 mL). The solvent was evaporated under reduced pressure, CH_2Cl_2 (500 mL) and H_2O (100 mL) were added and HCl was added until pH 5-6. The phases were separated and the aqueous phase was extracted with CH_2Cl_2 (100 mL). The combined organic phases were dried over Na_2SO_4 and the solvent was removed under reduced pressure. **8** was isolated by FCC (4:1 hexanes/EtOAc; Rf = 0.38) in 98% yield as a colorless resin (28.4 g, 57.1 mmol).

^{1}H NMR (400 MHz, $CDCl_3$) δ 8.08 – 7.93 (m, 2H), 7.53 – 7.46 (m, 1H), 7.44 – 7.35 (m, 4H), 7.32 – 7.25 (m, 3H), 7.24 – 7.20 (m, 2H), 7.18 – 7.12 (m, 4H), 5.59 (s, 1H), 5.56 (dd, *J* = 3.4, 1.4 Hz, 1H), 5.31 (d, *J* = 1.3 Hz, 1H), 4.73 – 4.55 (m, 2H), 4.24 – 4.15 (m, 2H), 4.12 (t, *J* = 9.5 Hz, 1H), 3.97 (dd, *J* = 9.7, 3.4 Hz, 1H), 3.86 – 3.78 (m, 1H), 2.55 (qq, *J* = 13.0, 7.4 Hz, 2H), 1.20 (t, *J* = 7.4 Hz, 3H).

^{13}C NMR (101 MHz, $CDCl_3$) δ 165.9, 137.9, 137.6, 133.5, 130.1, 1299, 129.1, 128.6, 128.4, 128.3, 127.8, 127.8, 126.3, 101.8, 83.7, 79.1, 74.4, 72.3, 72.2, 68.8, 64.7, 25.8, 15.1.

HRMS (ESI): $C_{29}H_{30}NaO_6S$ $[M+Na]^+$; calculated: 529.1661., found: 529.1658.

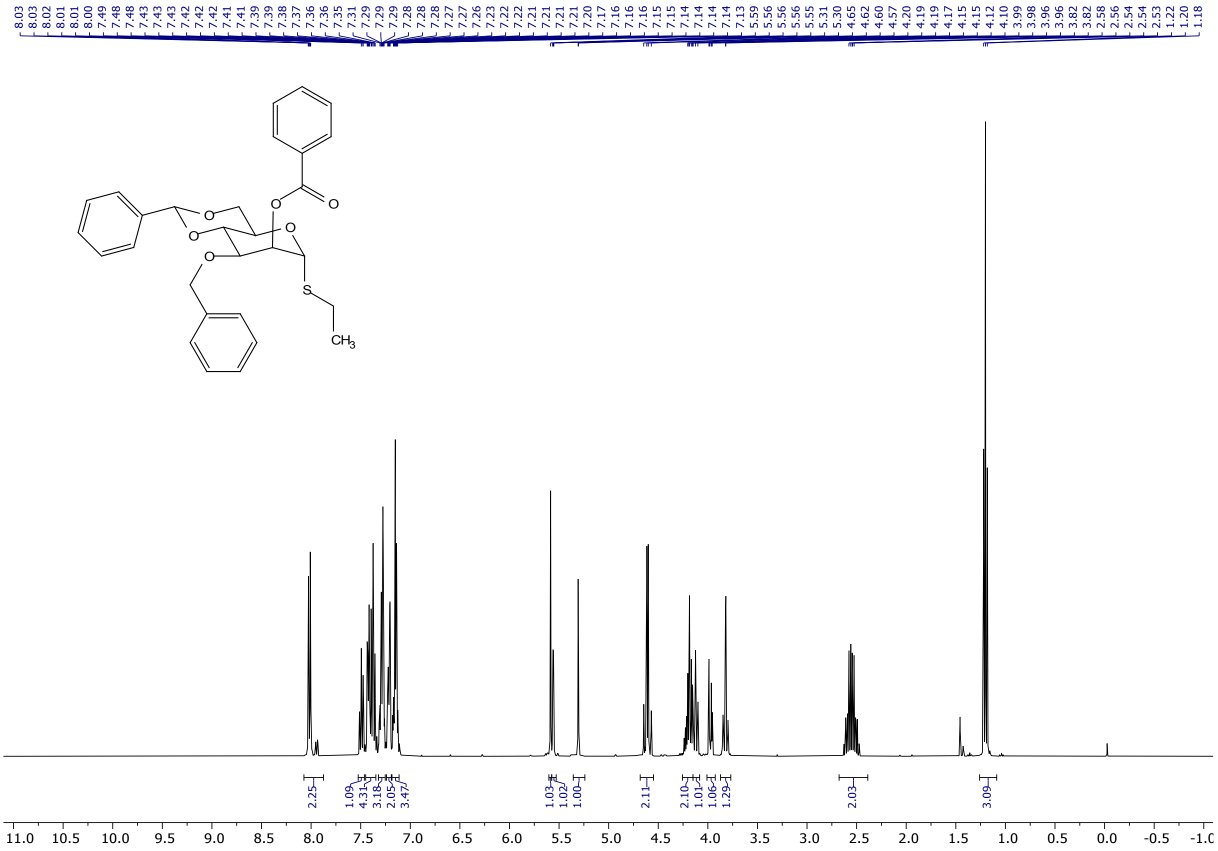

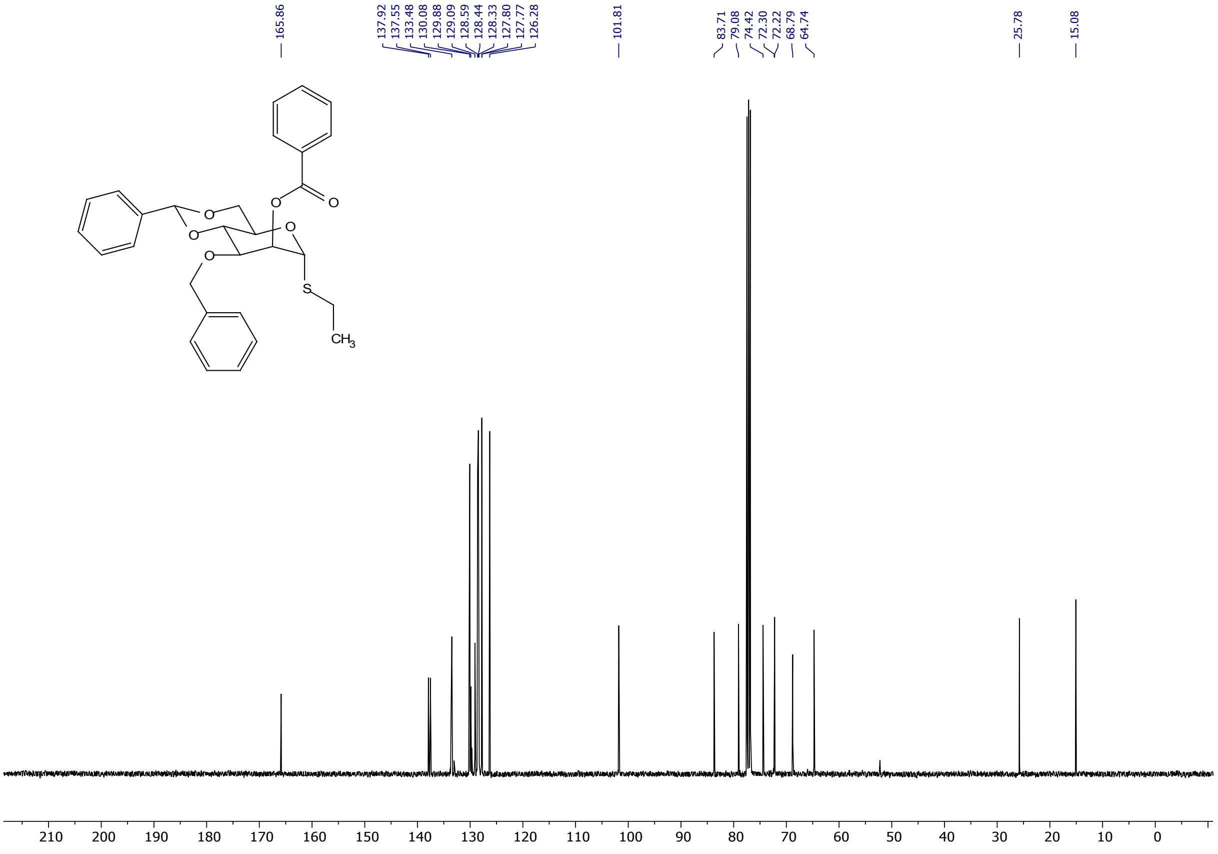

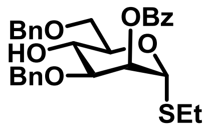

Ethyl 2-*O*-benzoyl-3,6-di-*O*-benzyl-1-thio-*α*-D-mannopyranoside (SI13)

Triethyl silane (13.1 mL, 82.2 mmol, 3 eq.) was added to benzylidene **S12** (13.9 g, 27.4 mmol, 1 eq.) and activated MS (4 Å) in anhydrous CH_2Cl_2 (500 mL) at - 78 °C under nitrogen. After 15 min, TFA (8.2 mL, 110 mmol, 4 eq.) was added slowly. The reaction was stirred for 1 h and then placed into an ice bath and stirred for another 1 h. Then, triethylamine was added (8 mL) and the reaction was washed with sat. aq. $NaHCO_3$. The aqueous phase was extracted with CH_2Cl_2, the combined organic phases were dried over Na_2SO_4 and the solvent was removed under reduced pressure. **SI13** was isolated by FCC (2:1:7 EtOAc/CH_2Cl_2/hexanes; Rf = 0.38) in 74% yield as a colorless resin (10.3 g, 20.1 mmol).

^{1}H NMR (400 MHz, $CDCl_3$) δ 8.11 – 8.02 (m, 2H), 7.62 – 7.52 (m, 1H), 7.45 – 7.22 (m, 12H), 5.69 (dd, J = 3.1, 1.7 Hz, 1H), 5.47 (d, J = 1.6 Hz, 1H), 4.78 (d, J = 11.3 Hz, 1H), 4.71 (d, J = 11.9 Hz, 1H), 4.60 (d, J = 12.0 Hz, 1H), 4.50 (d, J = 11.3 Hz, 1H), 4.28 – 4.16 (m, 2H), 3.95 – 3.79 (m, 3H), 2.77 – 2.60 (m, 2H), 2.57 (d, J = 1.9 Hz, 1H), 1.32 (t, J = 7.4 Hz, 3H).

^{13}C NMR (101 MHz, $CDCl_3$) δ 165.8, 138.4, 137.5, 133.3, 130.0, 129.8, 128.6, 128.5, 128.4, 128.3, 128.1, 127.6, 127.5, 8.7, 78.2, 73.7, 71.9, 71.6, 70.3, 69.8, 67.5, 25.7, 15.0.

HRMS (ESI): $C_{29}H_{32}NaO_6S$ $[M+Na]^+$; calculated: 531.1817, found: 531.1816.

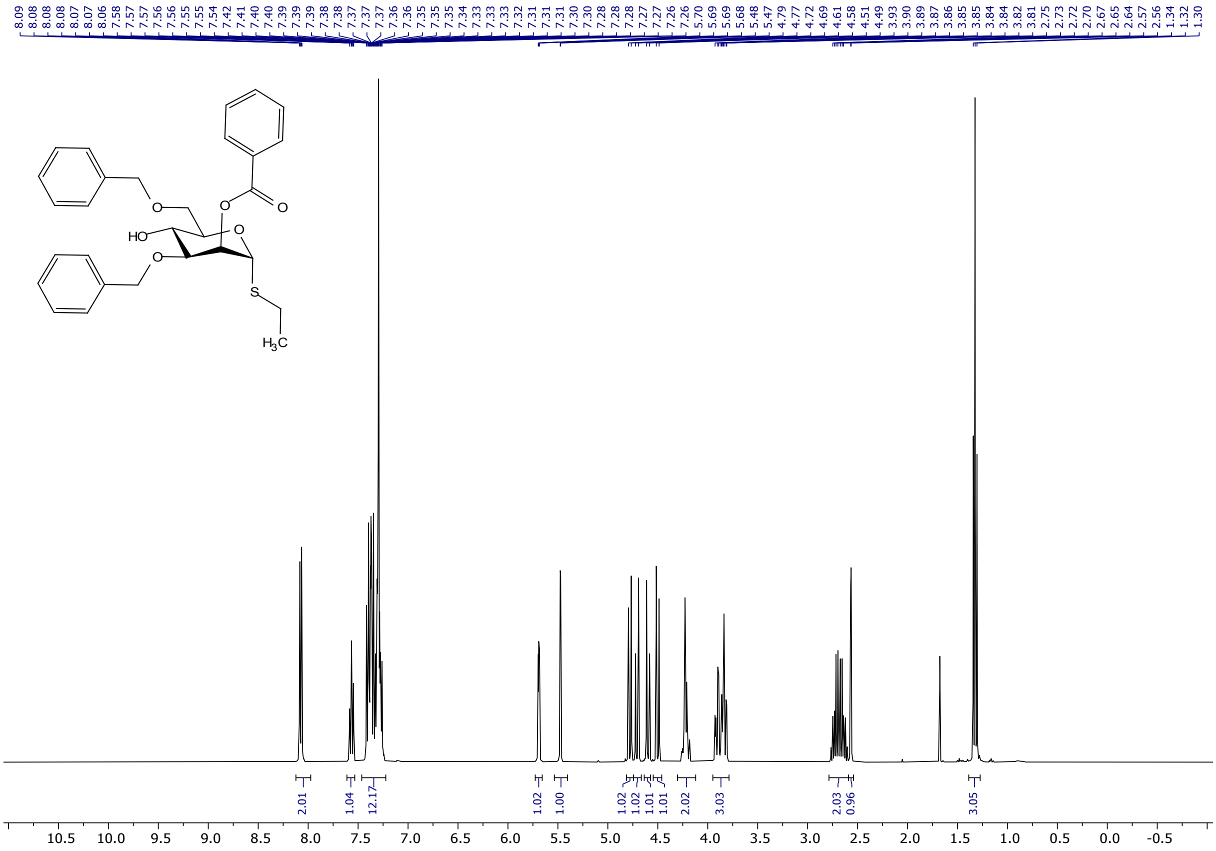
8.09 8.08 8.08 8.08 8.07 8.07 8.06 7.58 7.57 7.57 7.56 7.56 7.55 7.55 7.54 7.42 7.41 7.40 7.40 7.39 7.39 7.39 7.38 7.38 7.37 7.37 7.37 7.36 7.36 7.35 7.35 7.35 7.34 7.33 7.33 7.33 7.32 7.31 7.31 7.31 7.30 7.30 7.28 7.28 7.27 7.27 7.26 5.70 5.69 5.69 5.68 5.48 5.47 4.79 4.77 4.72 4.69 4.61 4.58 4.51 4.49 3.93 3.90 3.89 3.87 3.86 3.85 3.85 3.84 3.84 3.82 3.81 2.75 2.73 2.73 2.72 2.70 2.67 2.65 2.64 2.57 2.56 1.34 1.32 1.30
O
HO
O
O
O
S
H3C
2.01 1.04 12.17 1.02 1.00 1.02 1.02 1.01 1.01 2.02 3.03 2.03 0.96 3.05
10.5 10.0 9.5 9.0 8.5 8.0 7.5 7.0 6.5 6.0 5.5 5.0 4.5 4.0 3.5 3.0 2.5 2.0 1.5 1.0 0.5 0.0 -0.5

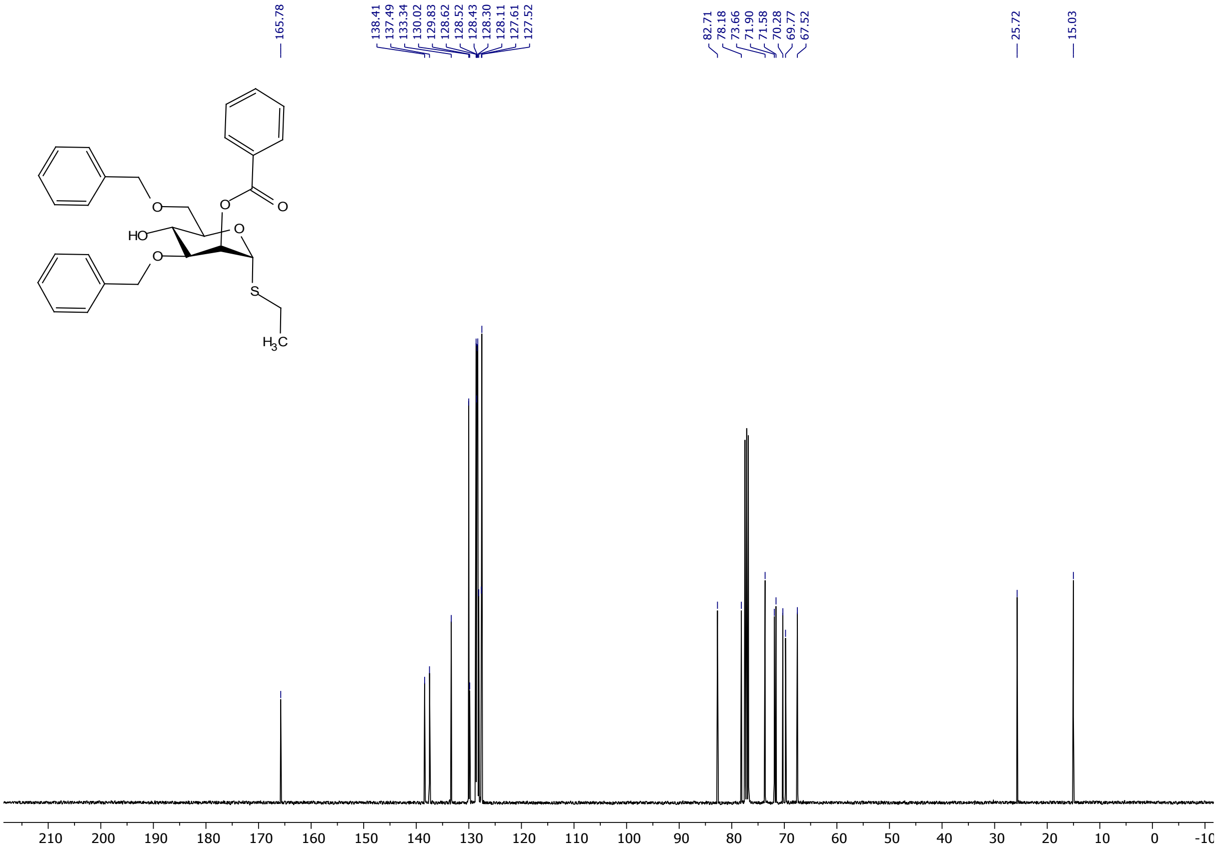
165.78
138.41 137.49 133.34 130.02 129.83 128.62 128.52 128.43 128.30 128.11 127.61 127.52
82.71 78.18 73.66 71.90 71.58 70.28 69.77 67.52
25.72
15.03
O
HO
O
O
O
S
H3C
210 200 190 180 170 160 150 140 130 120 110 100 90 80 70 60 50 40 30 20 10 0 -10

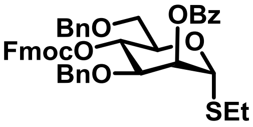

Ethyl 2-*O*-benzoyl-3,6-di-*O*-benzyl-4-O-(9-fluorenylmethoxycarbonyl)-1-thio-*α*-D-mannopyranoside (SI14)

FmocCl (7.6 g, 29.2 mmol, 1.5 eq.) and pyridine (7.9 mL, 97.5 mmol, 5 eq.) were added to alcohol **SI13** (9.92 g, 19.5 mmol, 1 eq.) in anhydrous CH_2Cl_2 (100 mL) at 0 °C under nitrogen. The reaction was allowed to warm to 23 °C and stirred for 16 h. The reaction was diluted with CH_2Cl_2 and washed with aq. citric acid (10% w/v). The phases were separated and the aqueous phase was extracted with CH_2Cl_2 (100 mL). The combined organic phases were dried over Na_2SO_4 and the solvent was removed under reduced pressure. **SI14** was isolated by FCC (2:1:7 EtOAc/CH_2Cl_2/hexanes; Rf = 0.44) in 93% yield as a colorless foam (13.2 g, 18.1 mmol).

^{1}H NMR (400 MHz, $CDCl_3$) δ 8.10 – 7.99 (m, 2H), 7.78 (dd, J = 7.7, 1.1 Hz, 2H), 7.62 – 7.57 (m, 2H), 7.56 – 7.51 (m, 1H), 7.44 – 7.33 (m, 6H), 7.32 – 7.21 (m, 7H), 7.21 – 7.17 (m, 3H), 5.67 (dt, J = 3.1, 1.4 Hz, 1H), 5.49 – 5.37 (m, 2H), 4.71 (d, J = 12.0 Hz, 1H), 4.63 (d, J = 11.7 Hz, 1H), 4.57 – 4.50 (m, 2H), 4.44 – 4.31 (m, 3H), 4.18 (t, J = 7.3 Hz, 1H), 4.07 – 4.01 (m, 1H), 3.74 (d, J = 3.7 Hz, 2H), 2.79 – 2.57 (m, 2H), 1.31 (t, J = 7.4 Hz, 3H).

^{13}C NMR (101 MHz, $CDCl_3$) δ 165.8, 154.7, 143.5, 143.4, 141.4, 138.2, 137.4, 133.4, 130.1, 129.7, 128.6, 128.5, 128.4, 128.0, 128.0, 127.9, 127.75, 127.6, 127.3, 125.3, 125.3, 120.2, 82.5, 75.4, 73.8, 72.9, 71.6, 70.7, 70.3, 70.2, 69.2, 46.9, 25.8, 15.0.

HRMS (ESI): $C_{44}H_{42}NaO_8S$ $[M+Na]^+$; calculated: 753.2498, found: 753.2532.

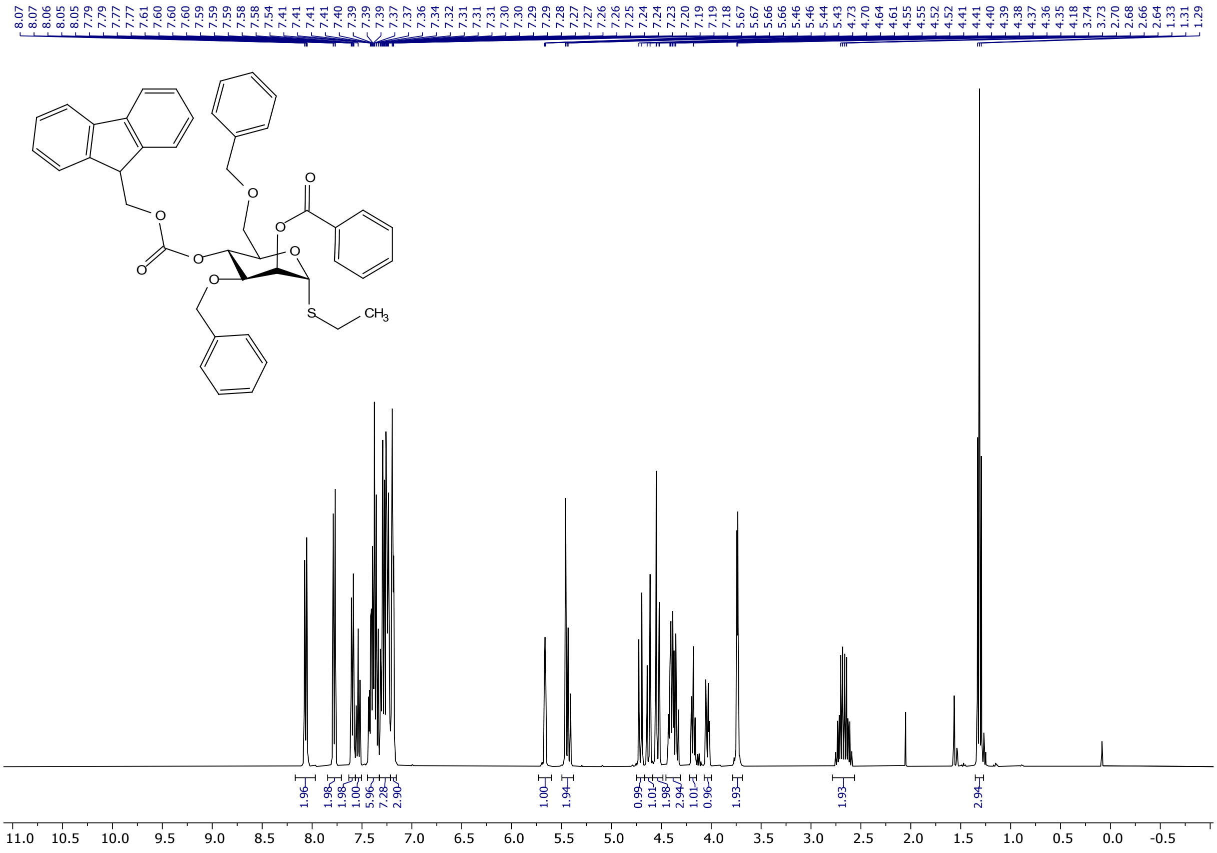
8.07 8.07 8.06 8.05 7.79 7.79 7.77 7.77 7.61 7.60 7.60 7.59 7.59 7.59 7.58 7.54 7.41 7.41 7.41 7.41 7.40 7.39 7.39 7.39 7.37 7.37 7.36 7.34 7.32 7.31 7.31 7.30 7.30 7.29 7.29 7.28 7.27 7.27 7.26 7.26 7.25 7.24 7.24 7.23 7.20 7.19 7.19 7.18 5.67 5.67 5.66 5.66 5.46 5.46 5.44 5.43 4.73 4.70 4.64 4.61 4.55 4.55 4.52 4.52 4.41 4.41 4.40 4.39 4.38 4.37 4.36 4.35 4.18 3.74 3.73 2.70 2.68 2.66 2.64 1.33 1.31 1.29
O
O
O
O
O
O
O
O
S
CH3
1.96 1.98 1.98 1.00 5.96 7.28 2.90 1.00 1.94 0.99 1.01 1.98 2.94 1.01 0.96 1.93 1.93 2.94
11.0 10.5 10.0 9.5 9.0 8.5 8.0 7.5 7.0 6.5 6.0 5.5 5.0 4.5 4.0 3.5 3.0 2.5 2.0 1.5 1.0 0.5 0.0 -0.5

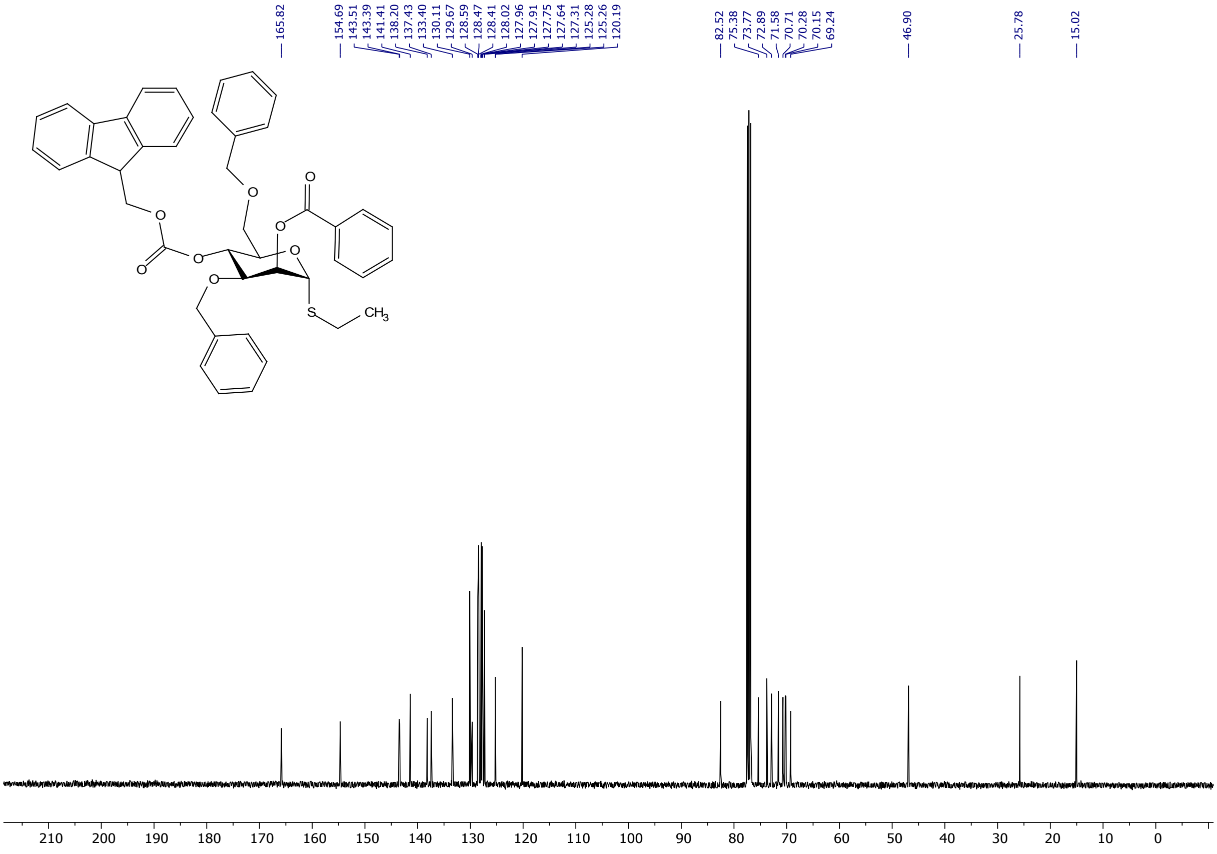
165.82
154.69 143.51 143.39 141.41 138.20 137.43 133.40 130.11 129.67 128.59 128.47 128.41 128.02 127.96 127.91 127.75 127.64 127.31 125.28 125.26 120.19
82.52 75.38 73.77 72.89 71.58 70.71 70.28 70.15 69.24
46.90
25.78
15.02
O
O
O
O
O
O
O
O
S
CH3
210 200 190 180 170 160 150 140 130 120 110 100 90 80 70 60 50 40 30 20 10 0

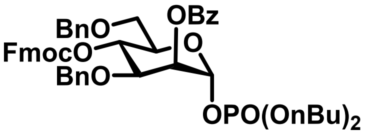

Dibutyl 2-*O*-benzoyl-3,6-di-*O*-benzyl-4-O-(9-fluorenylmethoxycarbonyl)-1-phosphate-*α*-D-mannopyranoside (10)

NIS (364 mg, 1.62 mmol, 1.2 eq.) was added to thioglycoside **SI14** (987 mg, 1.35 mmol, 1 eq.) and dibutyl phosphate (670 mL, 3.4 mmol, 2.5 eq.) and MS (3 Å) in anhydrous CH_2Cl_2 (0.1 M) at 0 °C under nitrogen. The reaction was stirred for 15 min before TfOH (36 µL, 405 µmol, 0.3 eq.) was added dropwise and stirred for 1 h. The reaction was stopped by addition of sat. aq. $NaHCO_3$, the phases were separated and the aqueous phase was extracted with CH_2Cl_2 (10 mL, three times). The combined organic phases were dried over Na_2SO_4 and the solvent was removed under reduced pressure. **10** was isolated by FCC (2:3 EtOAc/hexanes; Rf = 0.47) in 98% yield as a colorless oil (1.32 g, 1.16 mmol).

^{1}H NMR (400 MHz, $CDCl_3$) *δ* 8.06 – 8.00 (m, 2H), 7.82 – 7.72 (m, 2H), 7.62 – 7.50 (m, 3H), 7.44 – 7.32 (m, 6H), 7.32 – 7.20 (m, 7H), 7.20 – 7.13 (m, 3H), 5.78 (dd, *J* = 6.6, 2.1 Hz, 1H), 5.64 (t, *J* = 2.7 Hz, 1H), 5.44 (t, *J* = 10.0 Hz, 1H), 4.73 (d, *J* = 12.0 Hz, 1H), 4.61 (d, *J* = 11.7 Hz, 1H), 4.54 (t, *J* = 12.1 Hz, 2H), 4.43 – 4.31 (m, 2H), 4.23 (dt, *J* = 10.2, 3.6 Hz, 1H), 4.19 – 4.12 (m, 2H), 4.12 – 4.00 (m, 4H), 3.73 (d, *J* = 3.6 Hz, 2H), 1.70 – 1.59 (m, 4H), 1.46 – 1.33 (m, 4H), 0.93 (dt, *J* = 7.3, 7.3 Hz, 6H).

^{13}C NMR (101 MHz, $CDCl_3$) 165.5, 154.6, 143.4, 143.4, 141.4, 138.1, 137.4, 133.6, 130.1, 129.3, 128.6, 128.5, 128.4, 128.0, 127.9, 127.9, 127.8, 127.7, 127.3, 125.3, 120.2, 95.4, 95.3, 74.1, 73.8, 72.1, 71.8, 71.7, 70.2, 69.0, 68.4, 68.3, 68.3, 68.2, 68.2, 68.2, 46.9, 32.4, 32.3, 18.8, 18.8, 13.7.

^{31}P NMR (162 MHz, $CDCl_3$) *δ* -3.09.

HRMS (ESI): $C_{50}H_{55}NaO_{12}P$ $[M+Na]^+$; calculated: 901.3329, found: 901.3364.

Optical rotation: $[\alpha]_D^{25}$ = -13.7° (c = 1.0, $CHCl_3$)

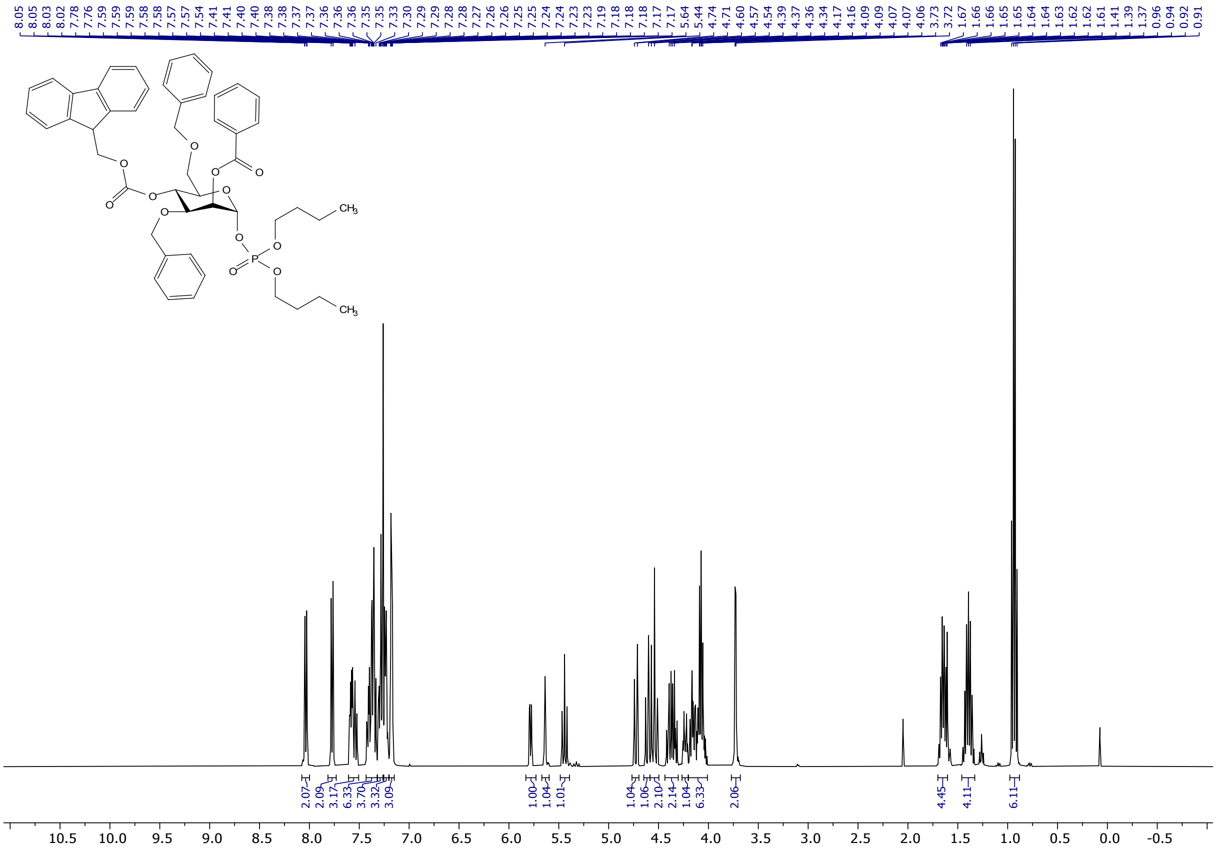
8.05 8.05 8.03 8.02 7.78 7.76 7.59 7.59 7.59 7.58 7.58 7.57 7.57 7.54 7.41 7.41 7.40 7.40 7.38 7.38 7.37 7.37 7.36 7.36 7.35 7.35 7.33 7.30 7.29 7.29 7.28 7.28 7.27 7.26 7.26 7.25 7.25 7.24 7.24 7.23 7.23 7.19 7.18 7.18 7.17 7.17 5.64 5.44 4.74 4.71 4.60 4.57 4.54 4.39 4.37 4.36 4.34 4.17 4.16 4.09 4.09 4.07 4.07 4.06 3.73 3.72 1.67 1.66 1.66 1.65 1.65 1.64 1.64 1.63 1.62 1.62 1.61 1.41 1.39 1.37 0.96 0.94 0.92 0.91
CH3
CH3
2.07 2.09 3.17 6.33 3.70 3.32 3.09 1.00 1.04 1.01 1.04 1.06 2.10 2.14 1.04 6.33 2.06 4.45 4.11 6.11
10.5 10.0 9.5 9.0 8.5 8.0 7.5 7.0 6.5 6.0 5.5 5.0 4.5 4.0 3.5 3.0 2.5 2.0 1.5 1.0 0.5 0.0 -0.5

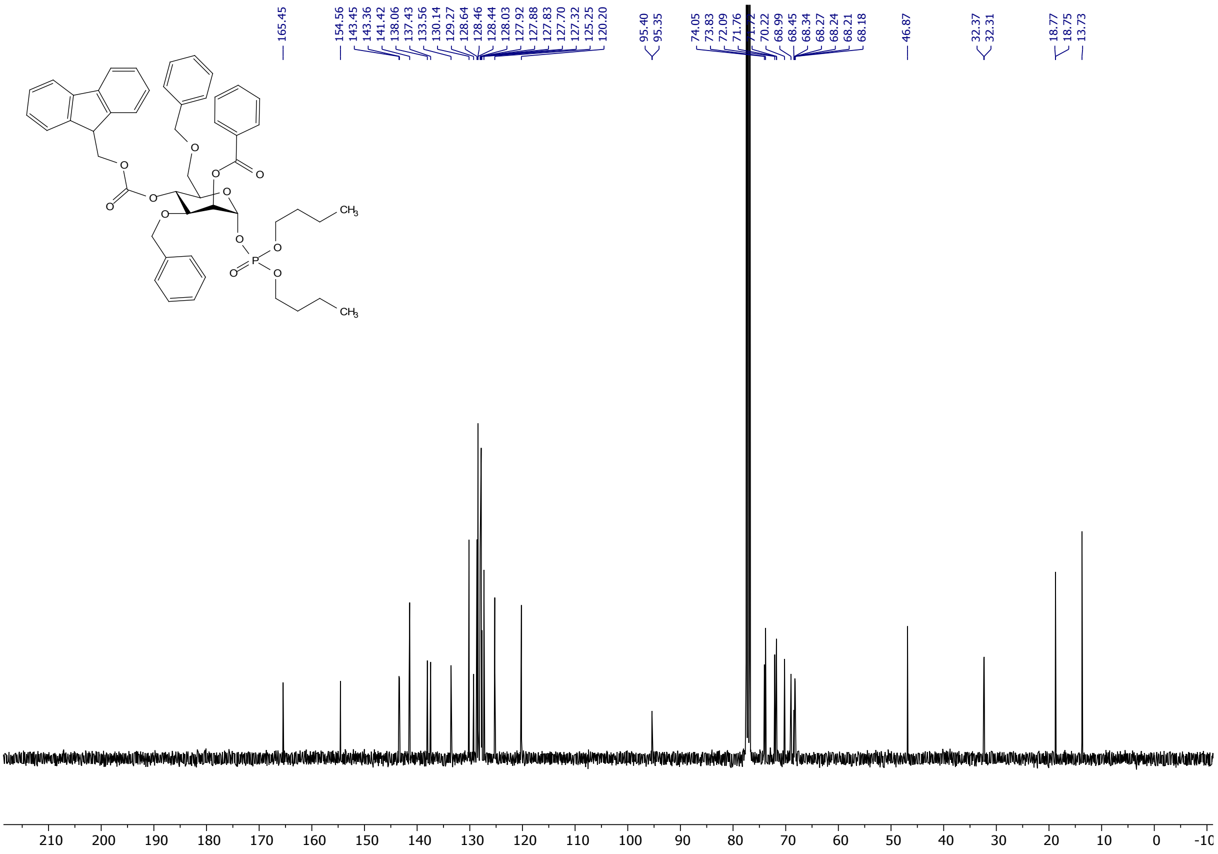
165.45 154.56 143.45 143.36 141.42 138.06 137.43 133.56 130.14 129.27 128.64 128.46 128.44 128.03 127.92 127.88 127.83 127.70 127.32 125.25 120.20 95.40 95.35 74.05 73.83 72.09 71.76 71.72 70.22 68.99 68.45 68.34 68.27 68.24 68.21 68.18 46.87 32.37 32.31 18.77 18.75 13.73
CH3
CH3
210 200 190 180 170 160 150 140 130 120 110 100 90 80 70 60 50 40 30 20 10 0 -10

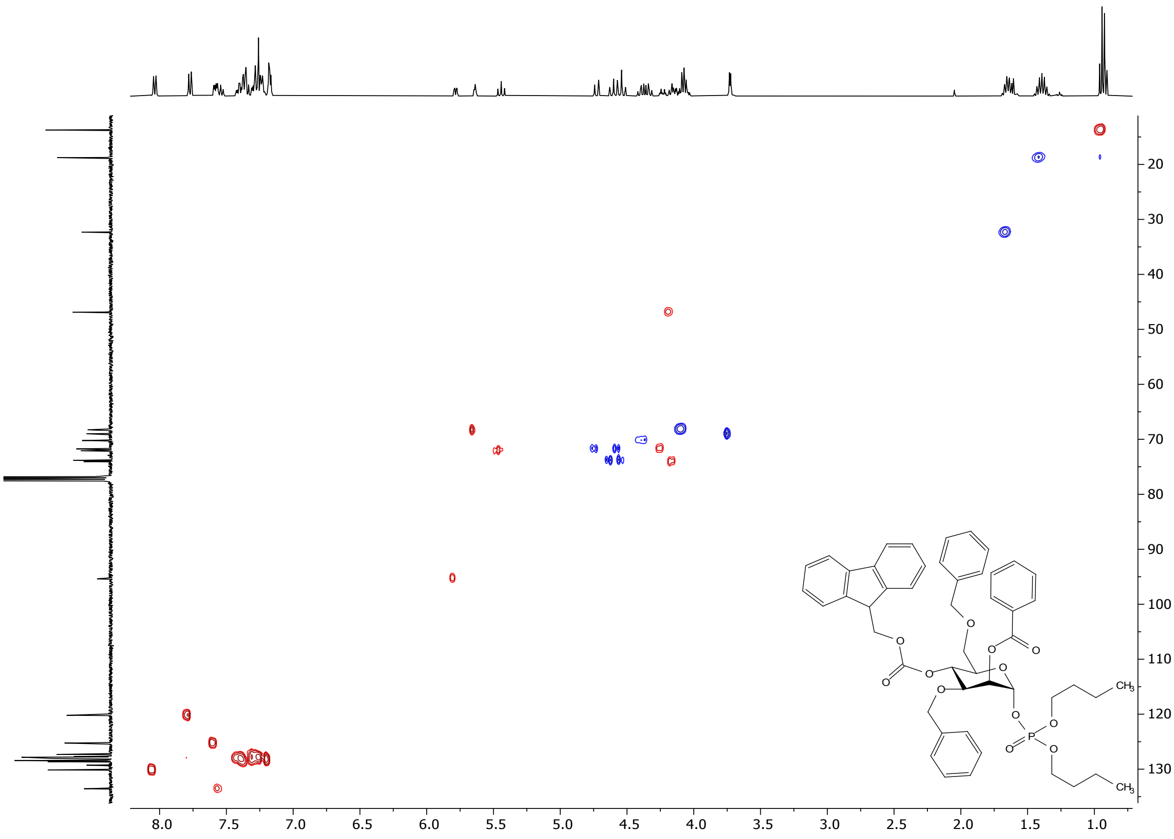
8.0
7.5
7.0
6.5
6.0
5.5
5.0
4.5
4.0
3.5
3.0
2.5
2.0
1.5
1.0
20
30
40
50
60
70
80
90
100
110
120
130
CH3
CH3

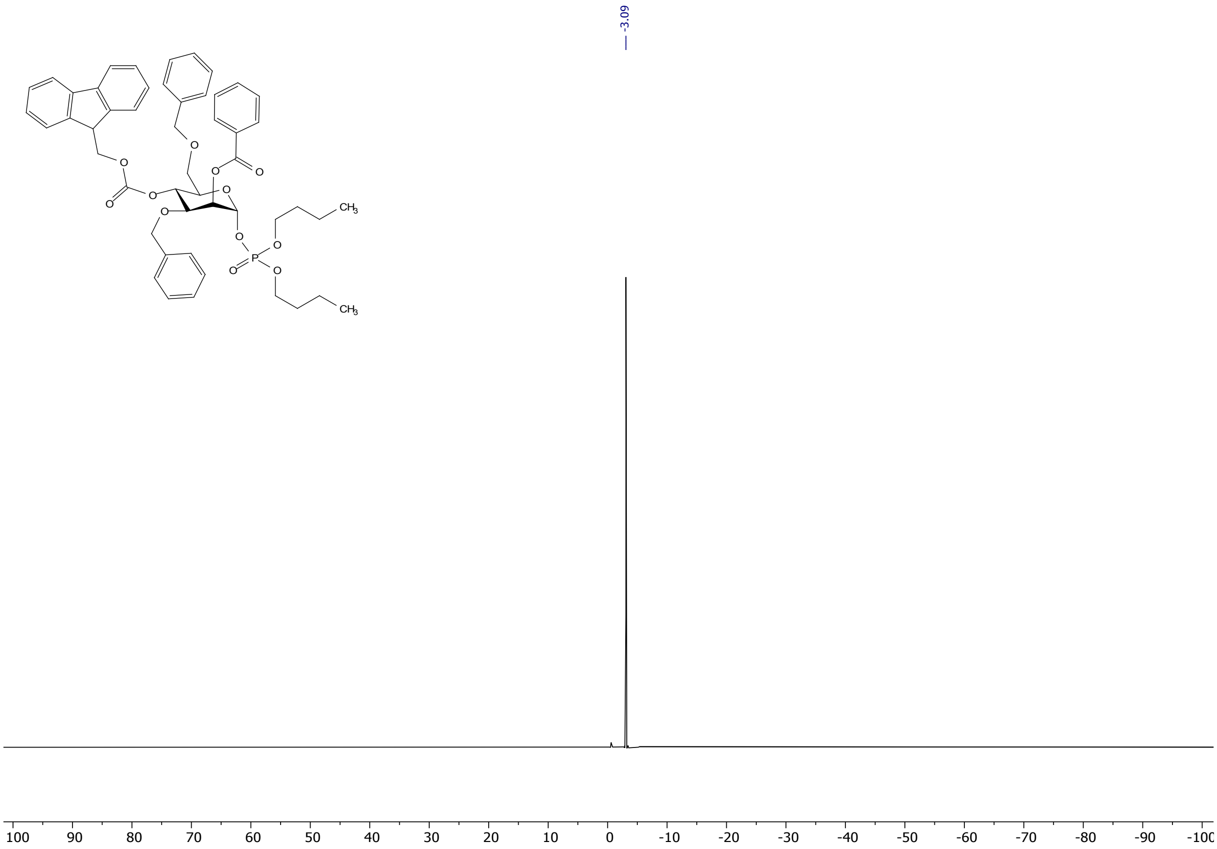
-3.09
CH3
CH3
100
90
80
70
60
50
40
30
20
10
0
-10
-20
-30
-40
-50
-60
-70
-80
-90
-100

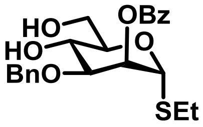

Ethyl 2-*O*-benzoyl-3-*O*-benzyl-1-thio-*α*-D-mannopyranoside (SI15)

TfOH (1.33 mL, 14.8 mmol, 0.5 eq.) was added to benzylidene **SI12** (15 g, 29.6 mmol, 1 eq.) in anhydrous MeOH (0.1 M) at 0 °C under nitrogen. The reaction was stirred for 1 h and at 23 °C and for another 1 h. The reaction was stopped by dropwise addition of 4 mL Et_3N and the solvent was removed under reduced pressure. **SI15** was isolated by FCC (6:1:3 EtOAc/CH_2Cl_2/hexanes; Rf = 0.31) in 99% yield as a colorless oil (12.2 g, 29.2 mmol).

^{1}H NMR (400 MHz, $CDCl_3$) δ 8.14 – 7.96 (m, 2H), 7.64 – 7.53 (m, 1H), 7.50 – 7.41 (m, 2H), 7.31 – 7.26 (m, 5H), 5.66 (dd, *J* = 3.1, 1.6 Hz, 1H), 5.42 (d, *J* = 1.6 Hz, 1H), 4.76 (d, *J* = 11.3 Hz, 1H), 4.47 (d, *J* = 11.3 Hz, 1H), 4.16 – 4.04 (m, 2H), 3.96 – 3.87 (m, 2H), 3.86 – 3.76 (m, 1H), 2.79 – 2.56 (m, 2H), 2.44 (d, *J* = 1.7 Hz, 1H), 2.02 (dd, *J* = 7.1, 5.9 Hz, 1H), 1.59 (s, 1H), 1.31 (t, *J* = 7.4 Hz, 3H).

^{13}C NMR (101 MHz, $CDCl_3$) δ 165.8, 137.4, 133.5, 130.0, 129.8, 128.7, 128.7, 128.3, 128.2, 82.8, 78.2, 72.4, 71.6, 70.3, 67.5, 62.7, 25.8, 15.0.

HRMS (ESI): $C_{22}H_{26}NaO_6S$ $[M+Na]^+$; calculated: 441.1348, found: 441.1339.

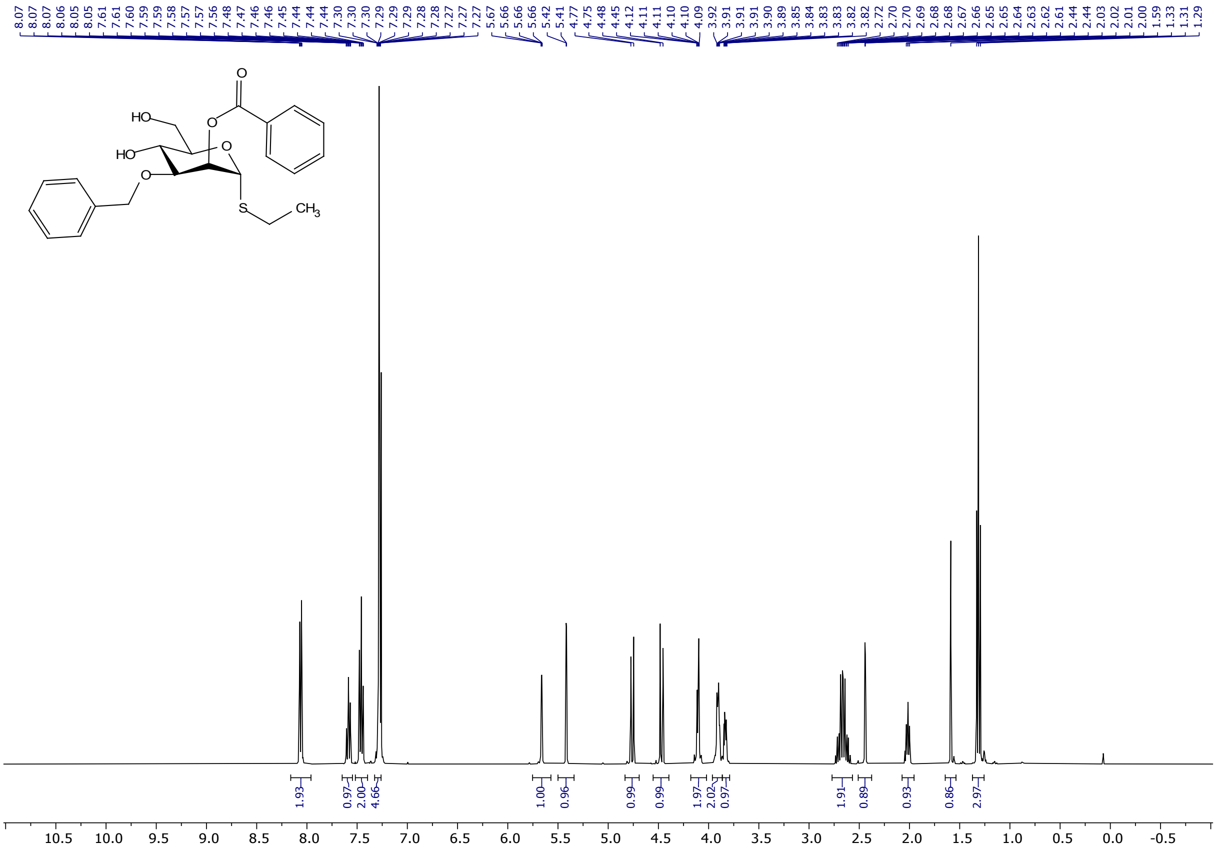

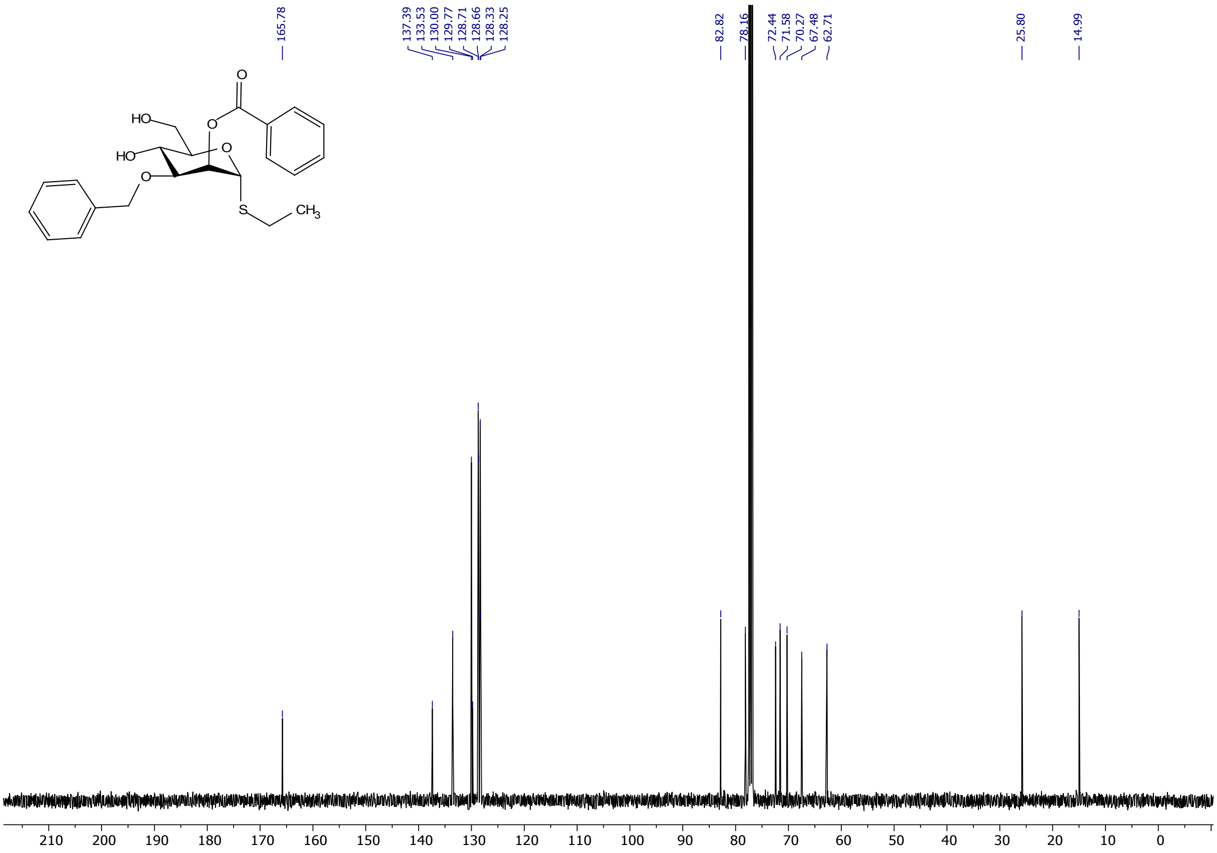

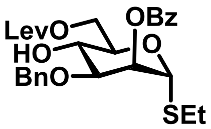

Ethyl 2-*O*-benzoyl-3-*O*-benzyl-6-*O*-levolinoyl-1-thio-*α*-D-mannopyranoside (SI16)

2-chloro-1-methylpyridinium iodide (14.7 g, 57.4 mmol, 1 eq.) was added to diol **SI15** (12 g, 28.7 mmol, 1 eq.) in anhydrous CH_2Cl_2 (0.1 M) at 0 °C under nitrogen. Then levulinic acid (3.24 mL, 31.6 mmol, 1.1 eq.) was added and the reaction was cooled to – 18 °C and stirred for 15 min. DABCO (9.7 g, 86.1 mmol, 3 eq.) was added and the reaction was stirred for 3 h. Sat. aq. $NaHCO_3$ was added and the aqueous phase was extracted with CH_2Cl_2, the combined organic phases were dried over Na_2SO_4 and the solvent was removed under reduced pressure. **SI16** was isolated by FCC running a gradient from 4:1:5 to 6:1:3 EtOAc/CH_2Cl_2/hexanes (Rf in 6:1:3 EtOAc/CH_2Cl_2/hexanes = 0.42) in 91% yield as a colorless oil (13.5 g, 26.1 mmol).

^{1}H NMR (400 MHz, $CDCl_3$) δ 8.12 – 8.03 (m, 2H), 7.62 – 7.54 (m, 1H), 7.49 – 7.42 (m, 2H), 7.31 – 7.26 (m, 5H), 5.65 (dd, *J* = 3.1, 1.6 Hz, 1H), 5.42 (d, *J* = 1.5 Hz, 1H), 4.76 (d, *J* = 11.3 Hz, 1H), 4.57 – 4.45 (m, 2H), 4.36 (dd, *J* = 12.0, 2.2 Hz, 1H), 4.25 (ddd, *J* = 9.8, 5.0, 2.1 Hz, 1H), 4.00 (td, *J* = 9.6, 2.3 Hz, 1H), 3.82 (dd, *J* = 9.4, 3.1 Hz, 1H), 2.81 – 2.57 (m, 7H), 2.16 (s, 3H), 1.32 (t, *J* = 7.4 Hz, 3H).

^{13}C NMR (101 MHz, $CDCl_3$) δ 206.6, 173.0, 165.7, 137.4, 133.5, 130.0, 129.8, 128.6, 128.6, 128.3, 128.2, 82.8, 77.9, 71.7, 70.9, 70.3, 67.0, 63.8, 38.0, 30.0, 27.9, 25.8, 15.0.

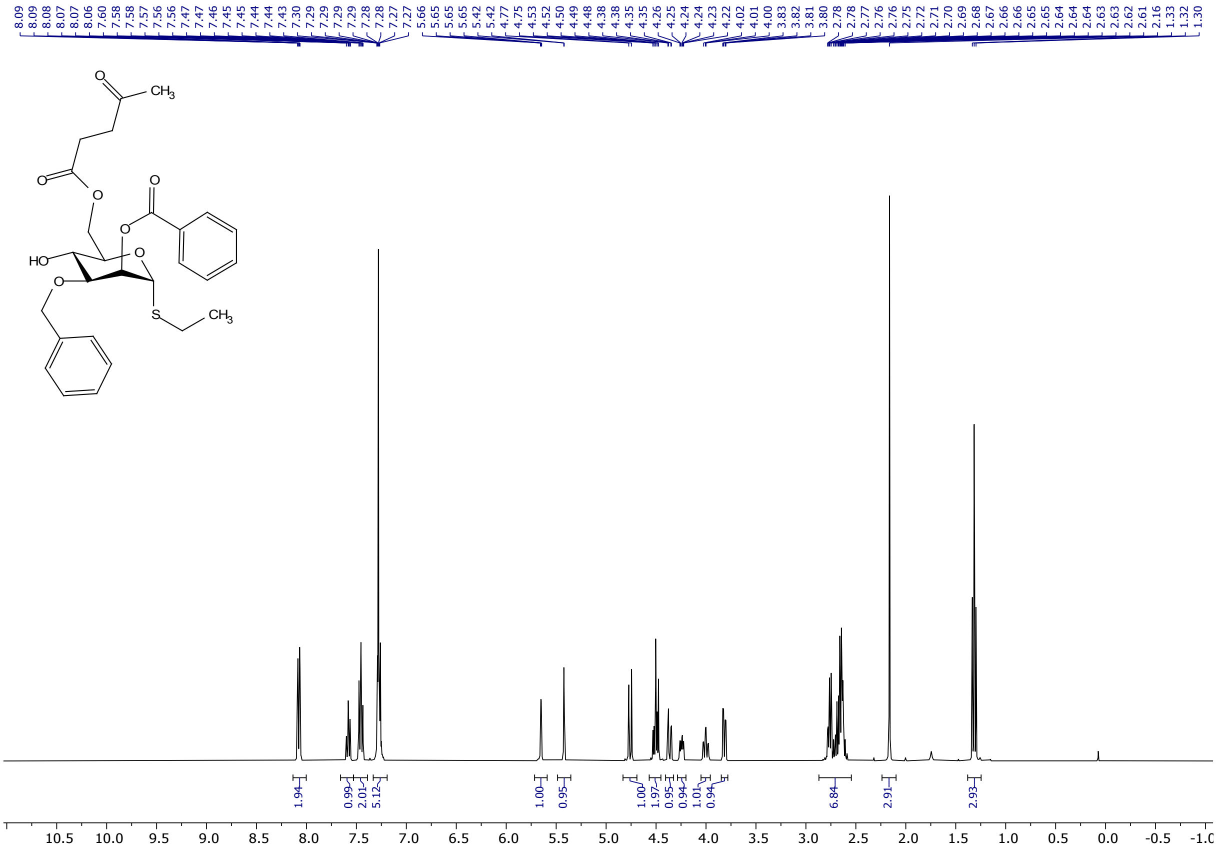
8.09 8.09 8.08 8.07 8.07 8.06 7.60 7.58 7.58 7.57 7.56 7.56 7.47 7.47 7.46 7.45 7.45 7.44 7.44 7.43 7.30 7.29 7.29 7.29 7.29 7.28 7.28 7.27 7.27 5.66 5.65 5.65 5.42 5.42 4.77 4.75 4.53 4.52 4.50 4.49 4.48 4.38 4.38 4.35 4.35 4.26 4.25 4.24 4.24 4.23 4.22 4.02 4.01 4.00 3.83 3.82 3.81 3.80 2.78 2.78 2.77 2.76 2.76 2.75 2.72 2.71 2.70 2.69 2.68 2.67 2.66 2.66 2.65 2.65 2.64 2.64 2.63 2.63 2.62 2.61 2.16 1.33 1.32 1.30
O
CH3
O
O
O
O
HO
O
O
S
CH3
1.94
0.99
2.01
5.12
1.00
0.95
1.00
1.97
0.95
0.94
1.01
0.94
6.84
2.91
2.93
10.5 10.0 9.5 9.0 8.5 8.0 7.5 7.0 6.5 6.0 5.5 5.0 4.5 4.0 3.5 3.0 2.5 2.0 1.5 1.0 0.5 0.0 -0.5 -1.0

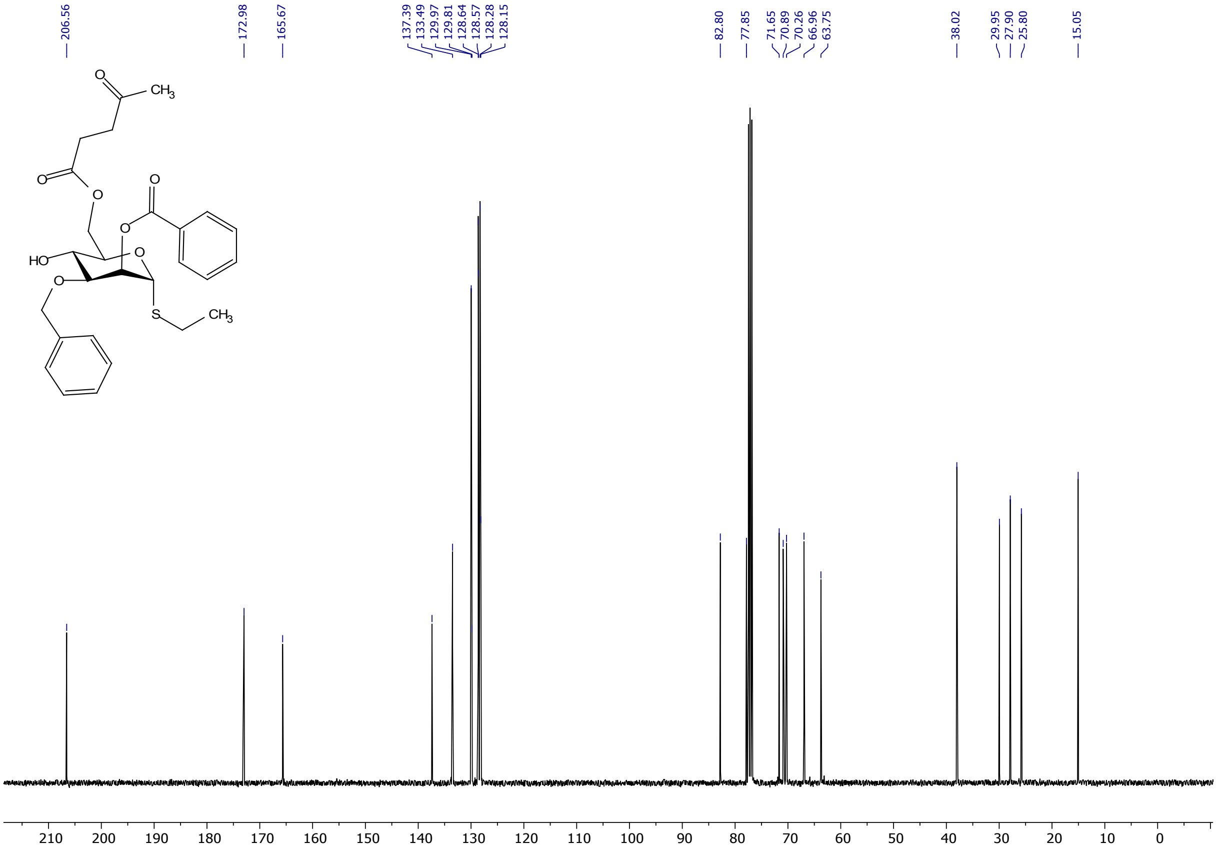
206.56
172.98
165.67
137.39 133.49 129.97 129.81 128.64 128.57 128.28 128.15
82.80
77.85
71.65 70.89 70.26 66.96 63.75
38.02
29.95 27.90 25.80
15.05
O
CH3
O
O
O
O
HO
O
O
S
CH3
210 200 190 180 170 160 150 140 130 120 110 100 90 80 70 60 50 40 30 20 10 0

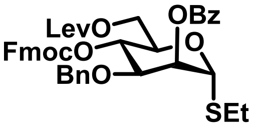

Ethyl 2-*O*-benzoyl-3-*O*-benzyl-4-O-(9-fluorenylmethoxycarbonyl)-6-*O*-levolinoyl-1-thio-*α*-D-mannopyranoside (SI17)

FmocCl (7.88 g, 30.5 mmol, 1.5 eq.) and pyridine (8.2 mL, 102 mmol, 5 eq.) were added to alcohol **SI16** (10.5 g, 20.3 mmol, 1 eq.) in anhydrous CH_2Cl_2 (0.1 M) at 0 °C under nitrogen. The reaction was allowed to warm to 23 °C and stirred for 16 h. The reaction was diluted with CH_2Cl_2 and washed with aq. citric acid (10% w/v). The phases were separated and the aqueous phase was extracted with CH_2Cl_2 (100 mL). The combined organic phases were dried over Na_2SO_4 and the solvent was removed under reduced pressure. **SI17** was isolated by FCC (2:1:7 EtOAc/CH_2Cl_2/hexanes; Rf = 0.38) in 94% yield as a colorless foam (14.1 g, 19.1 mmol).

^{1}H NMR (400 MHz, $CDCl_3$) *δ* 8.14 – 8.05 (m, 2H), 7.81 – 7.74 (m, 2H), 7.64 – 7.57 (m, 3H), 7.52 – 7.44 (m, 2H), 7.43 – 7.37 (m, 2H), 7.30 (q, *J* = 7.7 Hz, 2H), 7.24 – 7.16 (m, 5H), 5.67 – 5.61 (m, 1H), 5.45 – 5.40 (m, 1H), 5.29 (t, *J* = 9.7 Hz, 1H), 4.69 (d, *J* = 12.0 Hz, 1H), 4.52 (d, *J* = 12.1 Hz, 1H), 4.46 – 4.35 (m, 4H), 4.27 – 4.20 (m, 2H), 4.01 (dd, *J* = 9.7, 3.1 Hz, 1H), 2.84 – 2.57 (m, 6H), 2.15 (s, 3H), 1.32 (t, *J* = 7.4 Hz, 3H).

^{13}C NMR (101 MHz, $CDCl_3$) δ 206.5, 172.5, 165.7, 154.7, 143.4, 141.4, 141.4, 137.3, 133.6, 130.0, 129.7, 128.7, 128.5, 128.0, 128.0, 127.9, 127.3, 125.3, 125.2, 120.2, 120.2, 82.6, 75.1, 72.3, 71.6, 70.7, 70.3, 69.0, 62.9, 46.9, 38.0, 30.0, 27.9, 25.9, 15.0.

HRMS (ESI): $C_{42}H_{42}NaO_{10}S$ $[M+Na]^+$; calculated: 761.2396, found: 761.2421.

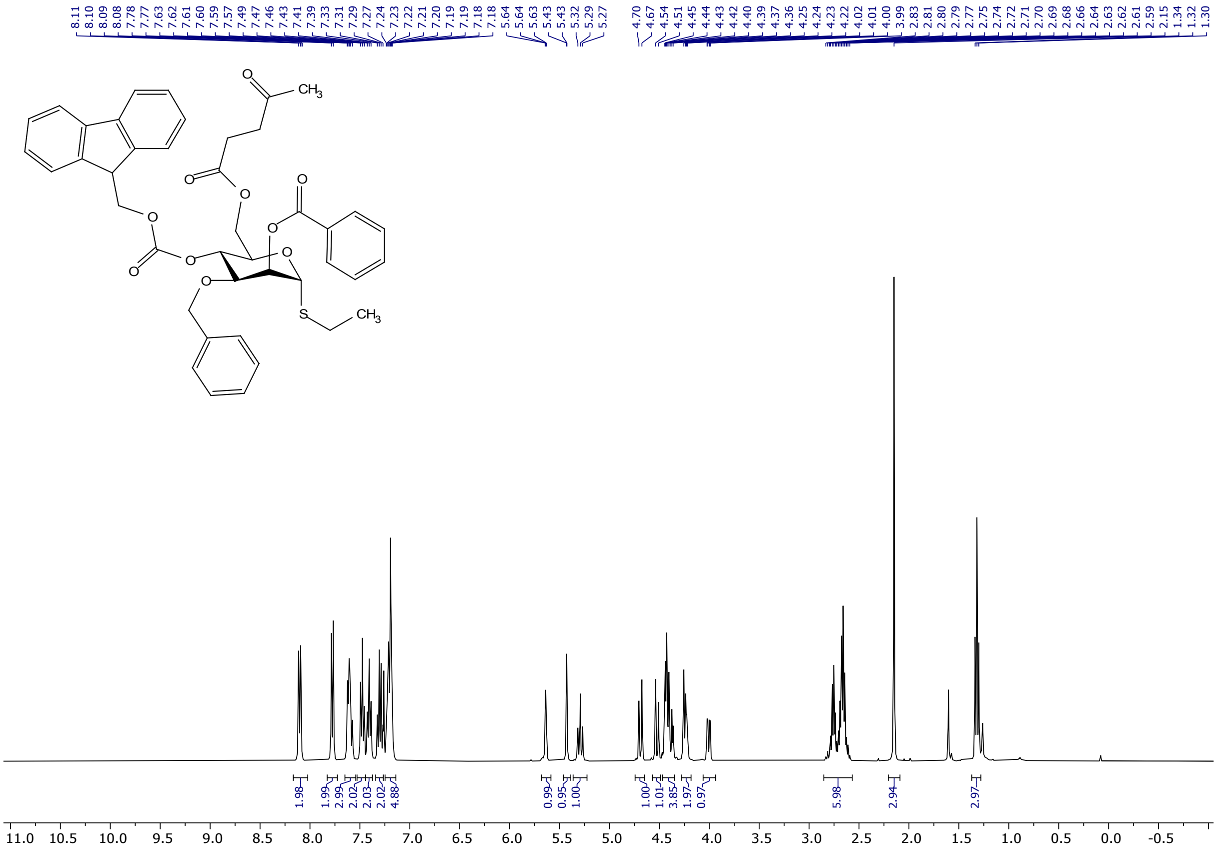
8.11 8.10 8.09 8.08 7.78 7.77 7.63 7.62 7.61 7.60 7.59 7.57 7.49 7.47 7.46 7.43 7.41 7.39 7.33 7.31 7.29 7.27 7.24 7.23 7.22 7.21 7.20 7.19 7.19 7.18 7.18 5.64 5.64 5.63 5.43 5.32 5.29 5.27 4.70 4.67 4.54 4.51 4.45 4.44 4.43 4.42 4.40 4.39 4.37 4.36 4.25 4.24 4.23 4.22 4.02 4.01 4.00 3.99 2.83 2.81 2.80 2.79 2.77 2.75 2.74 2.72 2.71 2.70 2.69 2.68 2.66 2.64 2.63 2.62 2.61 2.59 2.15 1.34 1.32 1.30
CH3
O
S
1.98 1.99 2.99 2.02 2.03 2.02 4.88 0.99 0.95 1.00 1.00 1.01 3.85 1.97 0.97 5.98 2.94 2.97
11.0 10.5 10.0 9.5 9.0 8.5 8.0 7.5 7.0 6.5 6.0 5.5 5.0 4.5 4.0 3.5 3.0 2.5 2.0 1.5 1.0 0.5 0.0 -0.5

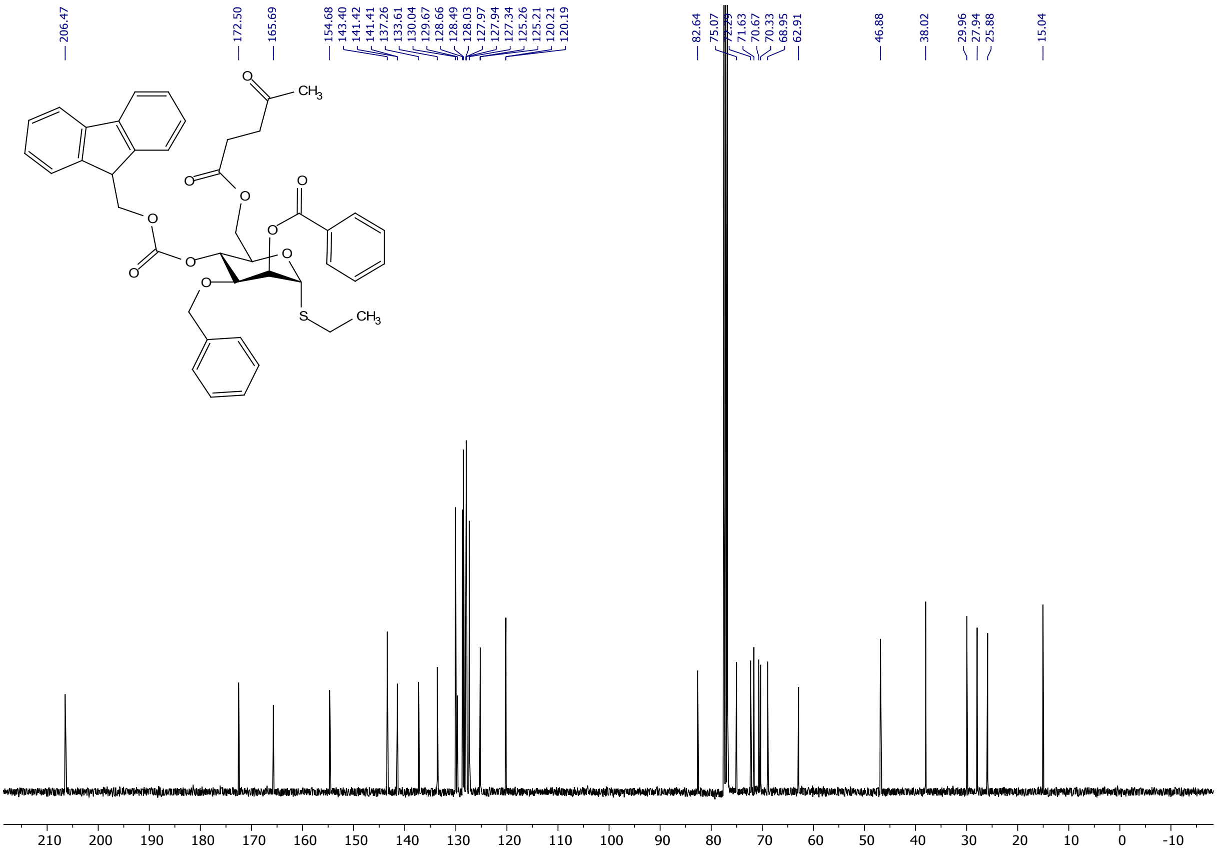
206.47 172.50 165.69 154.68 143.40 141.42 141.41 137.26 133.61 130.04 129.67 128.66 128.49 128.03 127.97 127.94 127.34 125.26 125.21 120.21 120.19 82.64 75.07 72.29 71.63 70.67 70.33 68.95 62.91 46.88 38.02 29.96 27.94 25.88 15.04
CH3
O
S
210 200 190 180 170 160 150 140 130 120 110 100 90 80 70 60 50 40 30 20 10 0 -10

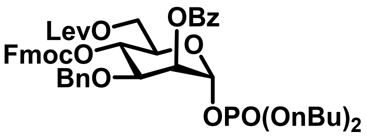

Dibutyl 2-*O*-benzoyl-3-*O*-benzyl-4-O-(9-fluorenylmethoxycarbonyl)-6-*O*-levolinoyl-1-phosphate-*α*-D-mannopyranoside (17)

NIS (6.7 g, 29.8 mmol, 1.2 eq.) was added to thioglycoside **SI16** (18.3 g, 24.8 mmol, 1 eq.) and dibutyl phosphate (12.3 mL, 62 mmol, 2.5 eq.) and activated MS (3 Å) in anhydrous CH_2Cl_2 (0.1 M) at 0 °C under nitrogen. The reaction was stirred for 15 min before TfOH (670 µL, 7.44 mmol, 0.3 eq.) was added dropwise and stirred for 1 h. The reaction was stopped by addition of sat. aq. $NaHCO_3$, the phases were separated and the aqueous phase was extracted with CH_2Cl_2 (10 mL, three times). The combined organic phases were washed with sat. aq. $NaHSO_3$, dried over Na_2SO_4 and the solvent was removed under reduced pressure. **17** was isolated by FCC (2:1:2 EtOAc/CH_2Cl_2/hexanes; Rf = 0.3) in 93% yield as a colorless oil (20.3 g, 22.9 mmol).

^{1}H NMR (400 MHz, $CDCl_3$) *δ* 8.14 – 8.04 (m, 2H), 7.77 (d, *J* = 7.6 Hz, 2H), 7.64 – 7.57 (m, 3H), 7.51 – 7.45 (m, 2H), 7.44 – 7.38 (m, 2H), 7.33 – 7.26 (m, 2H), 7.25 – 7.14 (m, 5H), 5.75 (dd, *J* = 6.4, 2.1 Hz, 1H), 5.62 (t, *J* = 2.6 Hz, 1H), 5.33 (t, *J* = 9.8 Hz, 1H), 4.71 (d, *J* = 12.0 Hz, 1H), 4.55 (d, *J* = 12.1 Hz, 1H), 4.49 – 4.33 (m, 3H), 4.28 – 4.19 (m, 3H), 4.16 – 4.02 (m, 5H), 2.85 – 2.62 (m, 4H), 2.14 (s, 3H), 1.73 – 1.62 (m, 4H), 1.48 – 1.37 (m, 4H), 0.96 (dt, *J* = 7.4, 1.1 Hz, 6H).

^{13}C NMR (101 MHz, $CDCl_3$) *δ* 206.4, 172.4, 165.3, 154.5, 143.4, 143.3, 141.4, 141.4, 137.3, 133.8, 130.1, 129.3, 128.7, 128.5, 128.0, 128.0, 127.9, 127.3, 125.2, 125.2, 120.2, 120.2, 95.3, 95.2, 73.8, 71.8, 71.3, 70.4, 70.2, 68.4, 68.4, 68.3, 68.2, 62.4, 46.9, 38.0, 32.4, 32.4, 32.3, 32.3, 29.9, 27.9, 18.8, 13.7.

HRMS (ESI): $C_{48}H_{55}NaO_{14}P$ $[M+Na]^+$; calculated: 909.3227, found: 909.3271.

Optical rotation: $[\alpha]_D^{25}$ = +18.1° (c = 1.0, $CHCl_3$)

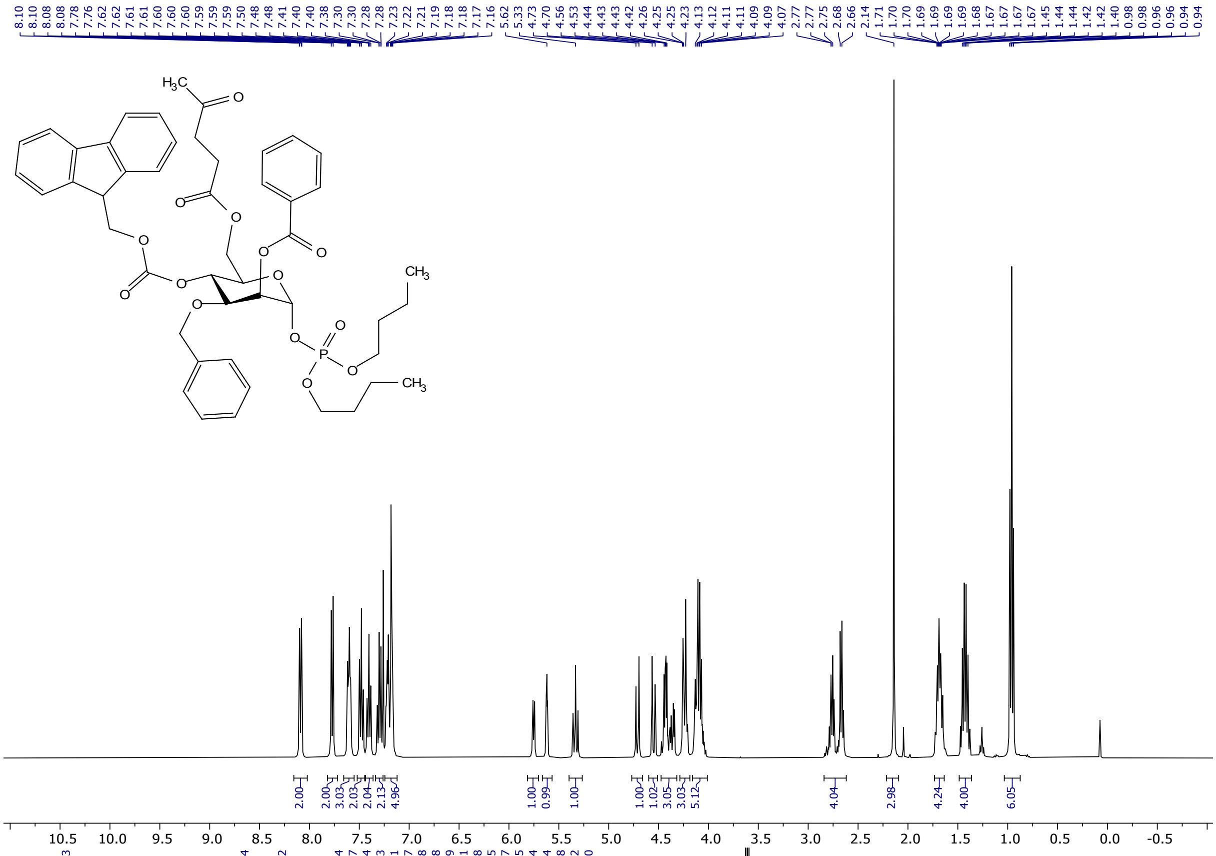
8.10 8.10 8.08 8.08 7.78 7.76 7.62 7.62 7.61 7.61 7.60 7.60 7.59 7.59 7.59 7.50 7.48 7.48 7.41 7.40 7.40 7.38 7.30 7.30 7.28 7.28 7.23 7.22 7.21 7.19 7.18 7.18 7.17 7.16 5.62 5.33 4.73 4.70 4.56 4.53 4.44 4.43 4.43 4.42 4.26 4.25 4.25 4.23 4.13 4.12 4.11 4.11 4.09 4.09 4.07 2.77 2.75 2.68 2.66 2.14 1.71 1.70 1.69 1.69 1.69 1.68 1.67 1.67 1.67 1.67 1.45 1.44 1.44 1.42 1.42 1.40 0.98 0.98 0.96 0.96 0.94 0.94
H_3C
O
CH_3
P
2.00 2.00 3.03 2.03 2.04 2.13 4.96 1.00 0.99 1.00 1.00 1.02 3.05 3.03 5.12 4.04 2.98 4.24 4.00 6.05
10.5 10.0 9.5 9.0 8.5 8.0 7.5 7.0 6.5 6.0 5.5 5.0 4.5 4.0 3.5 3.0 2.5 2.0 1.5 1.0 0.5 0.0 -0.5

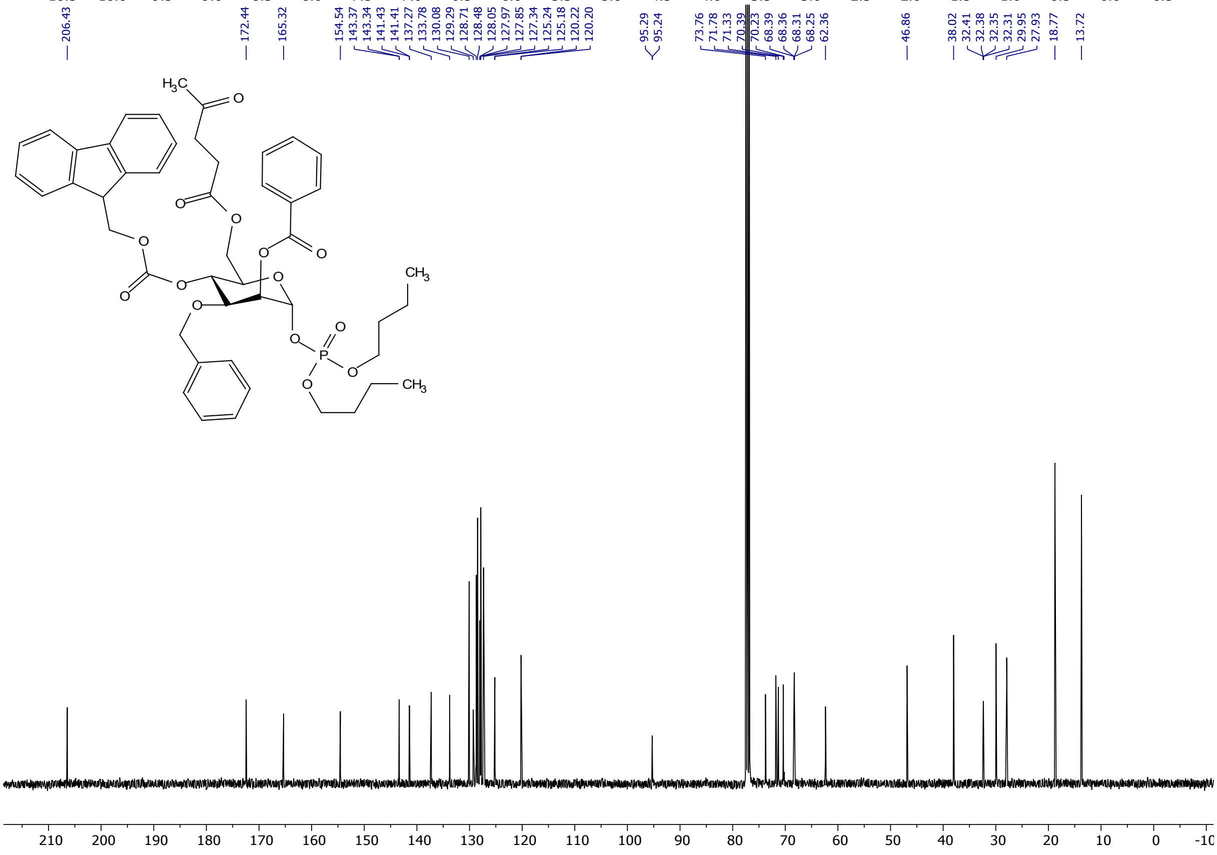
206.43 172.44 165.32 154.54 143.37 143.34 141.43 141.41 137.27 133.78 130.08 129.29 128.71 128.48 128.05 127.97 127.85 127.34 125.24 125.18 120.22 120.20 95.29 95.24 73.76 71.78 71.33 70.39 70.23 68.39 68.36 68.31 68.25 62.36 46.86 38.02 32.41 32.38 32.35 32.31 29.95 27.93 18.77 13.72
H_3C
O
CH_3
P
210 200 190 180 170 160 150 140 130 120 110 100 90 80 70 60 50 40 30 20 10 0 -10

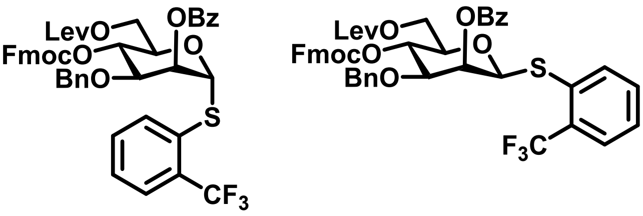

(Trifluoromethyl)phenyl 2-*O*-benzoyl-3-*O*-benzyl-4-*O*-(9-fluorenylmethoxycarbonyl)-6-*O*-levolinoyl-1-thio-*α*-D-mannopyranoside and (trifluoromethyl)phenyl 2-*O*-benzoyl-3-*O*-benzyl-4-*O*-(9-fluorenylmethoxycarbonyl)-6-*O*-levolinoyl-1-thio-*β*-D-mannopyranoside (SI18)

TMSOTf (1.55 mL, 8.56 mmol, 1.1 eq.) was added dropwise to a mixture of phosphoester **SI17** (6.9 g, 7.78 mmol, 1 eq.), 2-(trifluoromethyl)benzenethiol (2.1 mL, 15.6 mmol, 2 eq.) and activated MS (3 Å) in anhydrous CH_2Cl_2 (0.1 M) at 0 °C under nitrogen. The reaction was stopped after 2 h by addition of sat. aq. $NaHCO_3$, the phases were separated and the aqueous phase was extracted three times with CH_2Cl_2 (100 mL). The combined organic phases were dried over Na_2SO_4 and the solvent was removed under reduced pressure. **SI18** was isolated by FCC (7:3 Et_2O/Hexanes; Rf = 0.25) in 64% yield as a colorless foam (4.25 g, 4.97 mmol; 2.3 g alpha as first fraction, 0.8 g mixed alpha/beta, 1.15 g beta as the second fraction; approximate *α*/*β*-ratio 1.75:1).

***α* - ^{1}H NMR** (400 MHz, $CDCl_3$) *δ* 8.02 – 7.98 (m, 2H), 7.72 – 7.67 (m, 3H), 7.62 (dd, *J* = 8.0, 1.5 Hz, 1H), 7.55 (ddd, *J* = 7.5, 3.9, 1.0 Hz, 2H), 7.52 – 7.48 (m, 1H), 7.44 (td, *J* = 7.7, 1.5 Hz, 1H), 7.38 (dd, *J* = 8.3, 7.1 Hz, 2H), 7.33 (m, *J* = 7.7, 4.6, 2.0, 1.1 Hz, 3H), 7.23 (qd, *J* = 7.5, 1.2 Hz, 2H), 7.19 – 7.16 (m, 2H), 7.14 – 7.10 (m, 3H), 5.73 (dd, *J* = 3.1, 1.8 Hz, 1H), 5.57 (d, *J* = 1.8 Hz, 1H), 5.27 (t, *J* = 9.9 Hz, 1H), 4.67 (d, *J* = 11.9 Hz, 1H), 4.51 (d, *J* = 11.9 Hz, 1H), 4.46 (ddd, *J* = 10.1, 5.2, 2.4 Hz, 1H), 4.37 (dd, *J* = 7.2, 2.3 Hz, 2H), 4.32 (dd, *J* = 12.4, 4.9 Hz, 1H), 4.20 – 4.12 (m, 2H), 3.97 (dd, *J* = 9.7, 3.0 Hz, 1H), 2.66 (td, *J* = 6.9, 3.1 Hz, 2H), 2.55 (dd, *J* = 7.0, 5.9 Hz, 2H), 2.06 (s, 3H).

***β* - ^{1}H NMR** (400 MHz, $CDCl_3$) *δ* 8.10 – 8.04 (m, 2H), 7.80 – 7.75 (m, 1H), 7.68 (dd, *J* = 7.6, 1.0 Hz, 2H), 7.60 (dd, *J* = 7.9, 1.5 Hz, 1H), 7.56 – 7.49 (m, 3H), 7.46 – 7.38 (m, 3H), 7.35 – 7.28 (m, 3H), 7.24 – 7.17 (m, 2H), 7.17 – 7.13 (m, 2H), 7.12 – 7.08 (m, 3H), 5.94 (dd, *J* = 3.4, 1.1 Hz, 1H), 5.07 (t, *J* = 9.8 Hz, 1H), 4.83 (d, *J* = 1.1 Hz, 1H), 4.74 (d, *J* = 12.1 Hz, 1H), 4.46 (d, *J* = 12.1 Hz, 1H), 4.35 (dd, *J* = 7.1, 2.0 Hz, 2H), 4.27

(d, J = 4.4 Hz, 2H), 4.13 (t, J = 7.1 Hz, 1H), 3.77 – 3.58 (m, 2H), 2.83 – 2.46 (m, 4H), 2.09 (s, 3H).

α **- ^{13}C NMR** (101 MHz, $CDCl_3$) *δ* 206.5, 172.4, 165.5, 154.6, 143.3, 141.4, 141.4, 137.0, 134.5, 133.7, 132.6, 132.0, 131.7, 131.4, 131.1, 130.0, 129.4, 128.7, 128.5, 128.2, 128.1, 128.1, 128.0, 127.4, 127.1, 127.1, 127.0, 126.9, 125.2, 125.2, 120.2, 120.2, 86.8, 75.1, 71.9, 70.6, 70.4, 70.0, 62.7, 46.8, 38.0, 30.0, 27.9.

β **- ^{13}C NMR** (101 MHz, $CDCl_3$) *δ* 206.6, 172.5, 165.5, 154.6, 143.3, 143.3, 141.4, 137.1, 134.7, 133.6, 133.0, 132.3, 131.5, 131.2, 130.9, 130.3, 129.4, 128.6, 128.5, 128.1, 128.1, 128.0, 127.4, 127.3, 126.9, 126.9, 125.2, 125.2, 120.2, 120.2, 85.4, 77.5, 76.1, 71.9, 71.5, 70.4, 70.1, 63.3, 46.8, 38.0, 30.0, 27.9.

HRMS (ESI): $C_{47}H_{41}F_3NaO_{10}S$ $[M+Na]^+$; calculated: 877.2270, found: 877.2333.

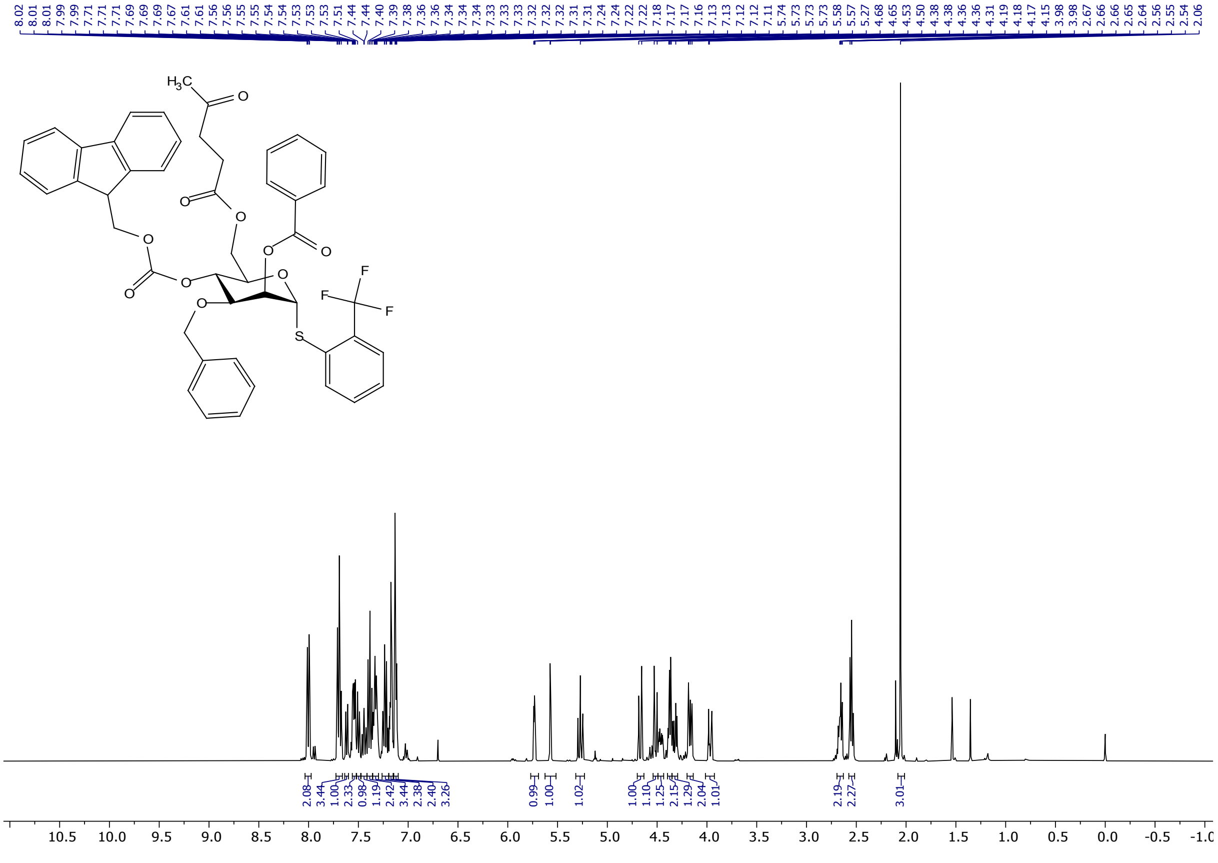

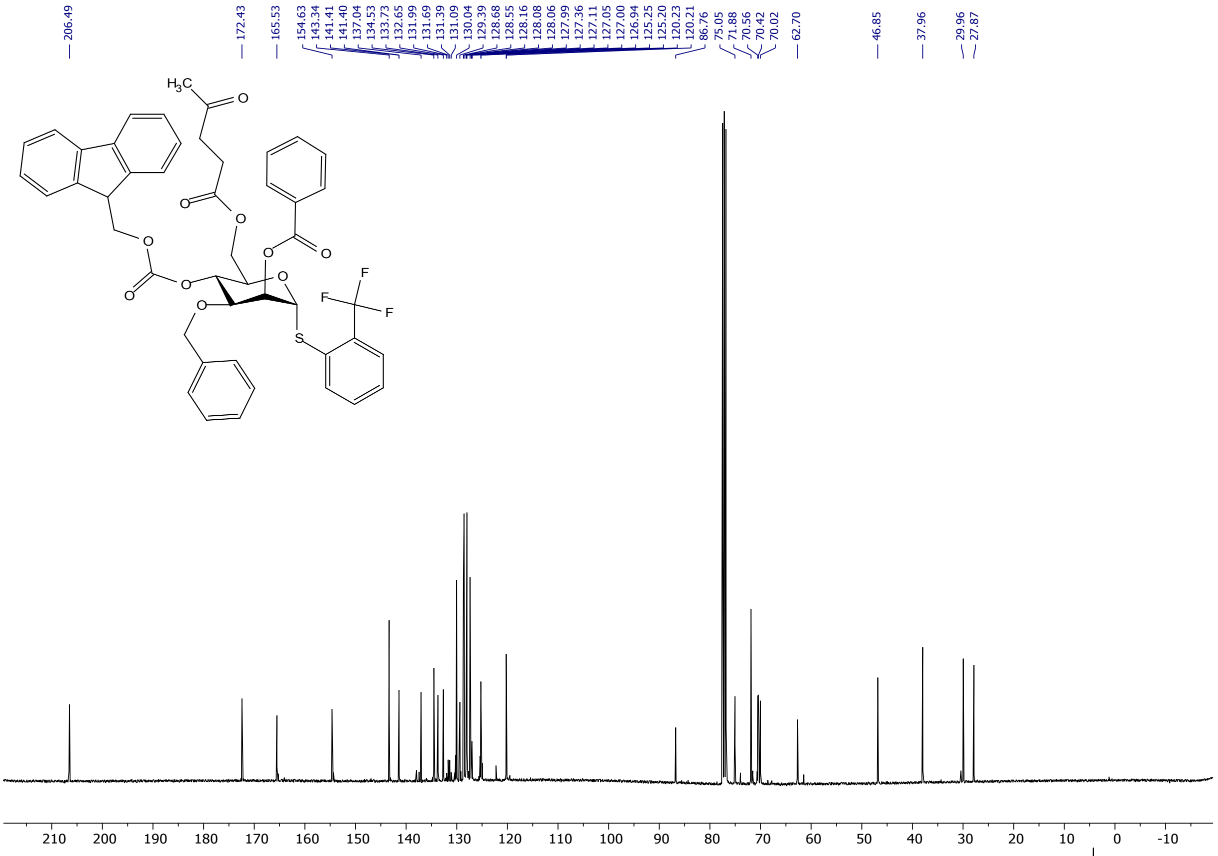
206.49
172.43
165.53
154.63
143.34
141.41
141.40
137.04
134.53
133.73
132.65
131.99
131.69
131.39
131.09
130.04
129.39
128.68
128.55
128.16
128.08
128.06
127.99
127.36
127.11
127.05
127.00
126.94
125.25
125.20
120.23
120.21
86.76
75.05
71.88
70.56
70.42
70.02
62.70
46.85
37.96
29.96
27.87

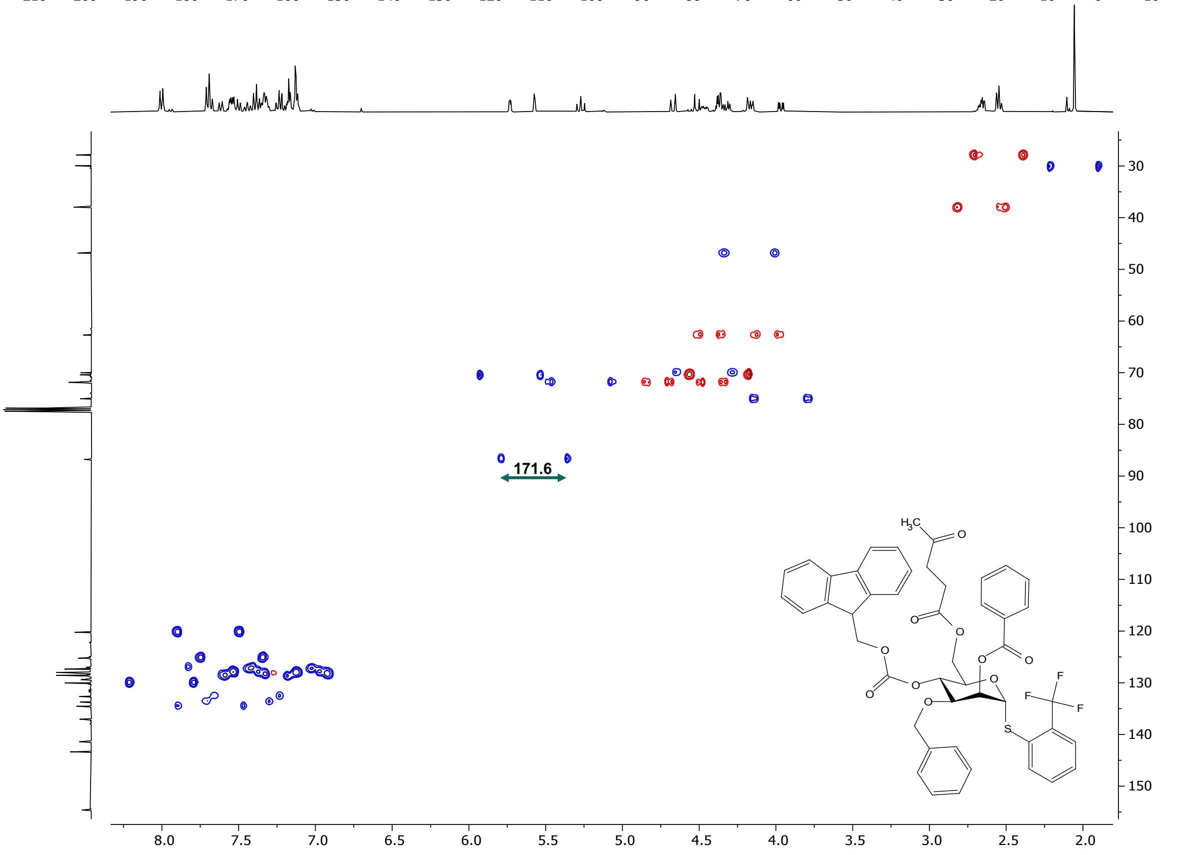
171.6

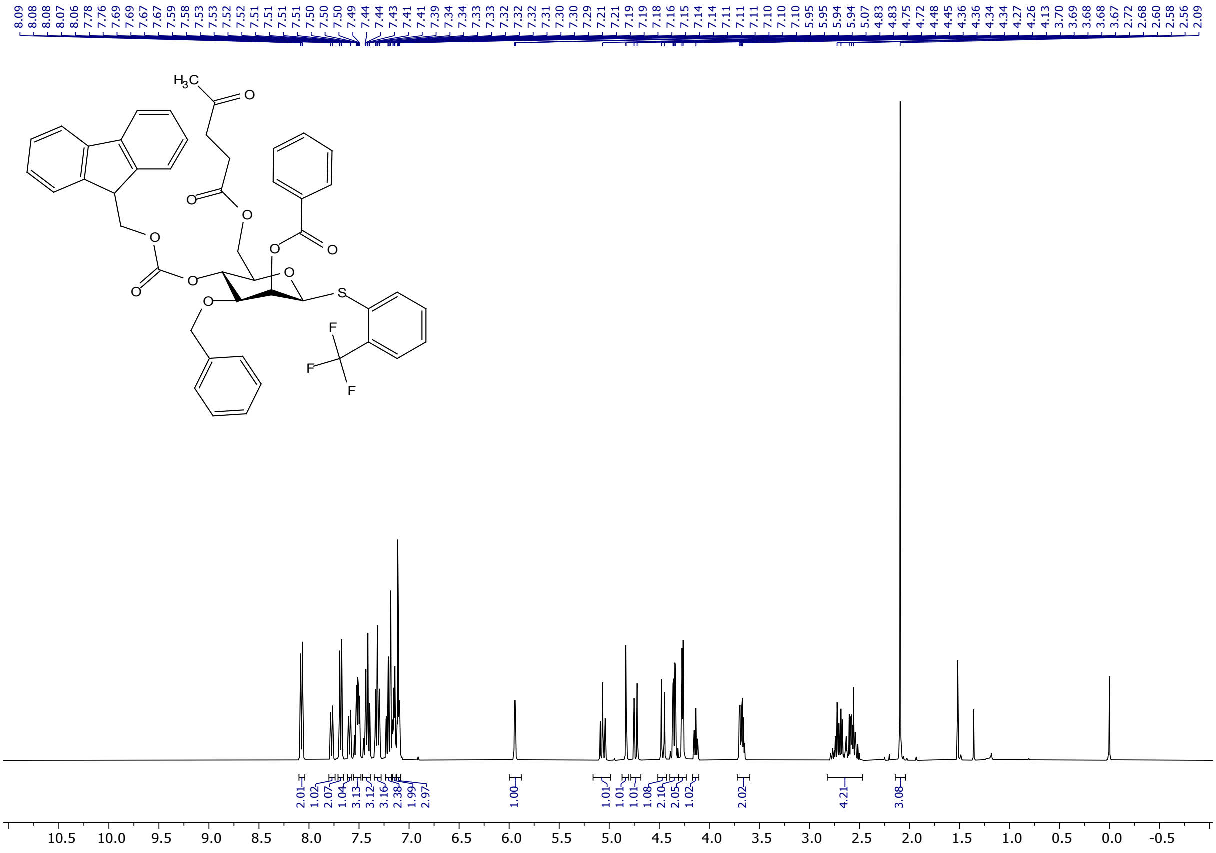
8.09 8.08 8.08 8.07 8.06 7.78 7.76 7.69 7.69 7.67 7.67 7.59 7.58 7.53 7.53 7.52 7.52 7.51 7.51 7.51 7.51 7.50 7.50 7.50 7.49 7.44 7.44 7.43 7.41 7.41 7.39 7.34 7.34 7.33 7.33 7.32 7.32 7.32 7.31 7.30 7.30 7.29 7.21 7.21 7.19 7.19 7.18 7.16 7.15 7.14 7.14 7.11 7.11 7.11 7.10 7.10 7.10 5.95 5.95 5.94 5.94 5.07 4.83 4.83 4.75 4.72 4.48 4.45 4.36 4.36 4.34 4.34 4.27 4.26 4.13 3.70 3.69 3.68 3.67 2.72 2.68 2.60 2.58 2.56 2.09
H3C
O
S
F
2.01 1.02 2.07 1.04 3.13 3.12 3.16 2.38 1.99 2.97 1.00 1.01 1.01 1.01 1.08 2.10 2.05 1.02 2.02 4.21 3.08
10.5 10.0 9.5 9.0 8.5 8.0 7.5 7.0 6.5 6.0 5.5 5.0 4.5 4.0 3.5 3.0 2.5 2.0 1.5 1.0 0.5 0.0 -0.5

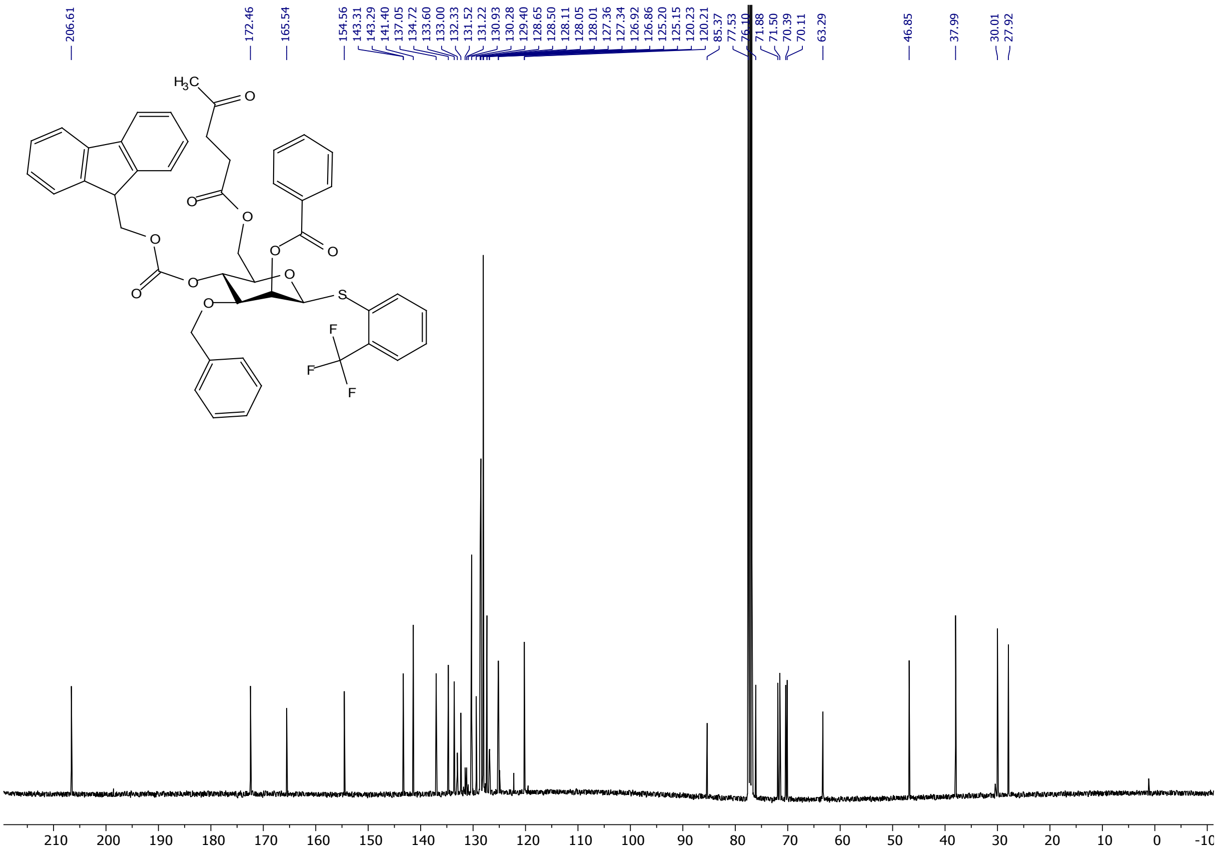
206.61 172.46 165.54 154.56 143.31 143.29 141.40 137.05 134.72 133.60 133.00 132.33 131.52 131.22 130.93 130.28 129.40 128.65 128.50 128.11 128.05 128.01 127.36 127.34 126.92 126.86 125.20 125.15 120.23 120.21 85.37 77.53 76.10 71.88 71.50 70.39 70.11 63.29 46.85 37.99 30.01 27.92
H3C
O
S
F
210 200 190 180 170 160 150 140 130 120 110 100 90 80 70 60 50 40 30 20 10 0 -10

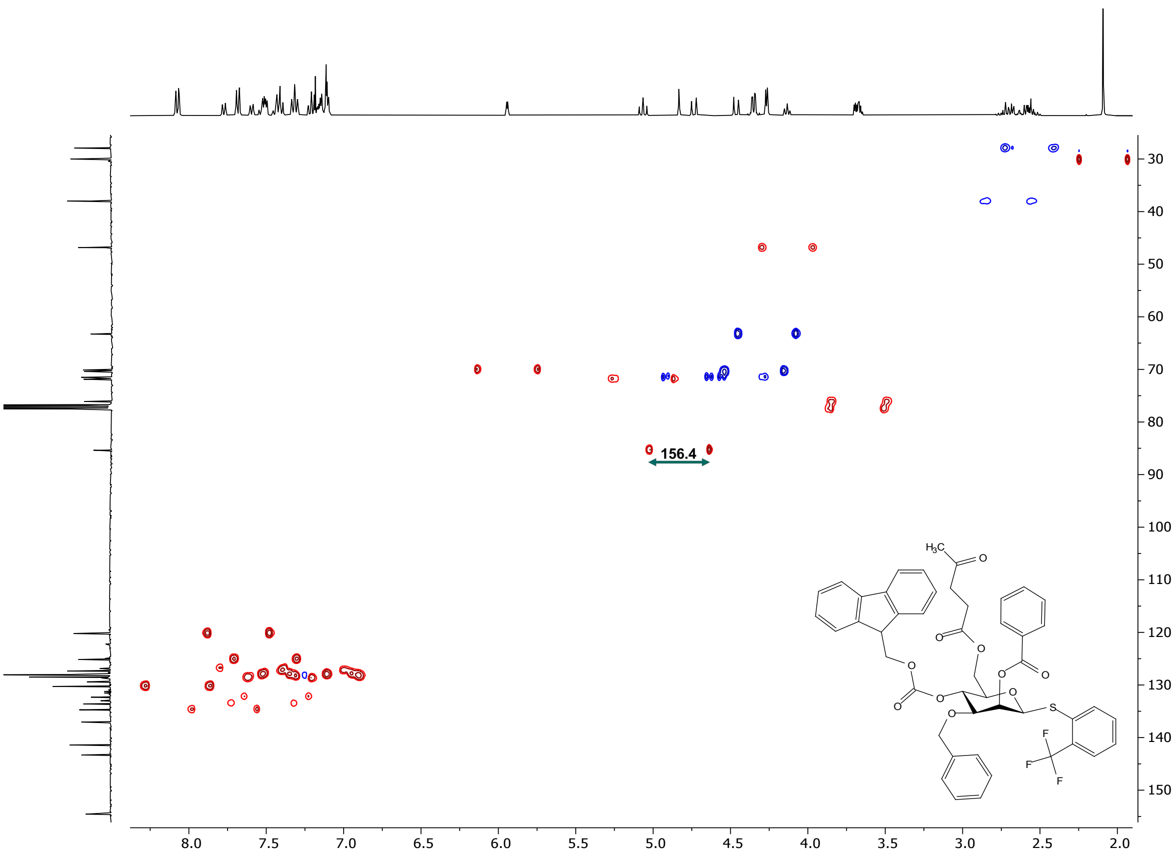

156.4
H3C
O
F
S
8.0
7.5
7.0
6.5
6.0
5.5
5.0
4.5
4.0
3.5
3.0
2.5
2.0
30
40
50
60
70
80
90
100
110
120
130
140
150

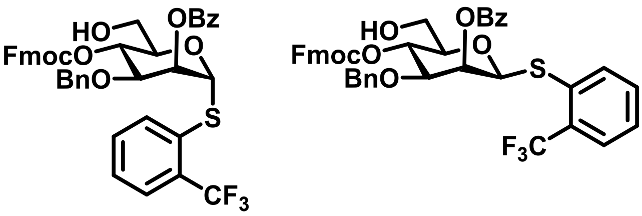

(Trifluoromethyl)phenyl 2-*O*-benzoyl-3-*O*-benzyl-4-*O*-(9-fluorenylmethoxycarbonyl)-1-thio-*α*-D-mannopyranoside and (trifluoromethyl)phenyl 2-*O*-benzoyl-3-*O*-benzyl-4-*O*-(9-fluorenylmethoxycarbonyl)-1-thio-*β*-D-mannopyranoside (9)

Hydrazine acetate (89 mg, 965 µmol, 1.5 eq.) was added levolinyl ester **10** (550 mg, 643 µmol, 1 eq.) in anhydrous THF/MeOH (0.1 M) under nitrogen. The reaction was stirred for 1h at 23 °C and 2 h at 35 °C. The reaction was diluted with CH_2Cl_2 and washed with water and aq. citric acid (10% w/v). The phases were separated and the aqueous phases was extracted two times with CH_2Cl_2 (20 mL). The combined organic phases were dried over Na_2SO_4 and the solvent was removed under reduced pressure. **9** was isolated by FCC (2:3 EtOAc/hexanes; Rf = 0.4) in quantitative yield as a colorless foam (486 mg, 643 µmol). Diastereomers could be separated best in Et_2O/Toluene.

***α* - ^{1}H NMR** (400 MHz, $CDCl_3$) *δ* 8.09 (dt, *J* = 8.0, 1.1 Hz, 2H), 7.80 (d, *J* = 7.5 Hz, 2H), 7.76 (d, *J* = 7.8 Hz, 1H), 7.71 (dd, *J* = 7.9, 1.5 Hz, 1H), 7.64 (dt, *J* = 7.5, 0.9 Hz, 2H), 7.61 – 7.55 (m, 1H), 7.51 (td, *J* = 7.8, 1.6 Hz, 1H), 7.48 – 7.39 (m, 5H), 7.38 – 7.27 (m, 5H), 7.26 – 7.24 (m, 2H), 5.85 (dt, *J* = 3.0, 1.5 Hz, 1H), 5.71 (d, *J* = 1.7 Hz, 1H), 5.37 (td, *J* = 9.9, 1.2 Hz, 1H), 4.78 (d, *J* = 11.9 Hz, 1H), 4.64 (d, *J* = 11.9 Hz, 1H), 4.52 (d, *J* = 7.0 Hz, 2H), 4.38 – 4.20 (m, 2H), 4.11 (dd, *J* = 9.6, 3.3 Hz, 1H), 3.85 – 3.67 (m, 2H), 2.41 (t, *J* = 7.0 Hz, 1H).

***β* - ^{1}H NMR** (400 MHz, $CDCl_3$) *δ* 8.07 (dt, *J* = 7.6, 0.9 Hz, 2H), 7.69 (t, *J* = 7.4 Hz, 3H), 7.60 (dd, *J* = 7.9, 1.5 Hz, 1H), 7.55 – 7.47 (m, 3H), 7.45 – 7.37 (m, 3H), 7.34 – 7.29 (m, 3H), 7.24 – 7.15 (m, 5H), 7.15 – 7.10 (m, 3H), 5.96 (d, *J* = 3.3 Hz, 1H), 5.07 (t, *J* = 9.8 Hz, 1H), 4.85 (d, *J* = 1.1 Hz, 1H), 4.74 (d, *J* = 12.1 Hz, 1H), 4.47 (d, *J* = 12.1 Hz, 1H), 4.39 (d, *J* = 7.0 Hz, 2H), 4.13 (t, *J* = 6.9 Hz, 1H), 3.77 – 3.62 (m, 3H), 3.46 (ddd, *J* = 10.0, 5.0, 2.6 Hz, 1H), 2.26 (ddt, *J* = 8.3, 5.8, 2.4 Hz, 1H).

***α* - ^{13}C NMR** (101 MHz, $CDCl_3$) *δ* 166.1, 155.9, 143.7, 143.5, 141.9, 141.9, 137.6, 134.8, 134.1, 133.2, 133.2, 133.2, 133.0, 132.3, 132.0, 131.7, 131.4, 130.5, 129.8,

129.1, 129.1, 129.0, 128.5, 128.5, 128.5, 128.4, 127.8, 127.8, 127.5, 127.5, 127.4, 127.4, 125.6, 125.6, 120.7, 120.7, 87.3, 75.3, 72.8, 72.4, 72.4, 71.2, 70.8, 61.6, 47.3.***β* - ^{13}C NMR** (101 MHz, $CDCl_3$) *δ* 165.6, 155.0, 143.3, 143.1, 141.4, 141.4, 137.1, 134.8, 133.6, 132.8, 132.4, 132.0, 131.7, 131.4, 131.1, 130.3, 129.3, 128.7, 128.5, 128.3, 128.1, 128.0, 128.0, 127.3, 127.1, 127.0, 127.0, 125.1, 125.1, 120.3, 120.2, 85.6, 78.6, 71.8, 71.5, 70.3, 70.2, 61.9, 46.9.

HRMS (ESI): $C_{42}H_{35}F_3NaO_8S$ $[M+Na]^+$; calculated: 779.1902, found: 779.1924.

Optical rotation: *α-anomer:* $[\alpha]_D^{25}$ = +39.1° (c = 1.0, $CHCl_3$); *β-anomer:* $[\alpha]_D^{25}$ = -87.8° (c = 1.0, $CHCl_3$)

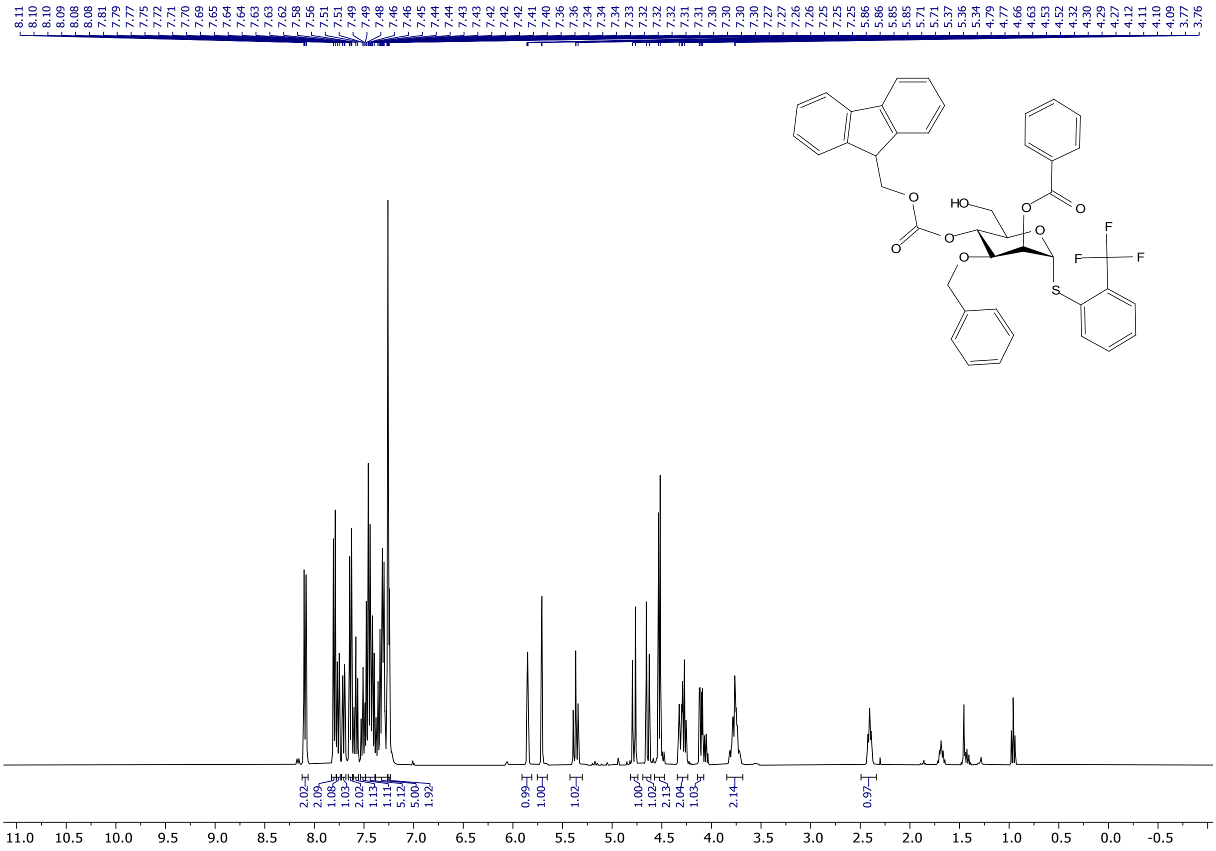

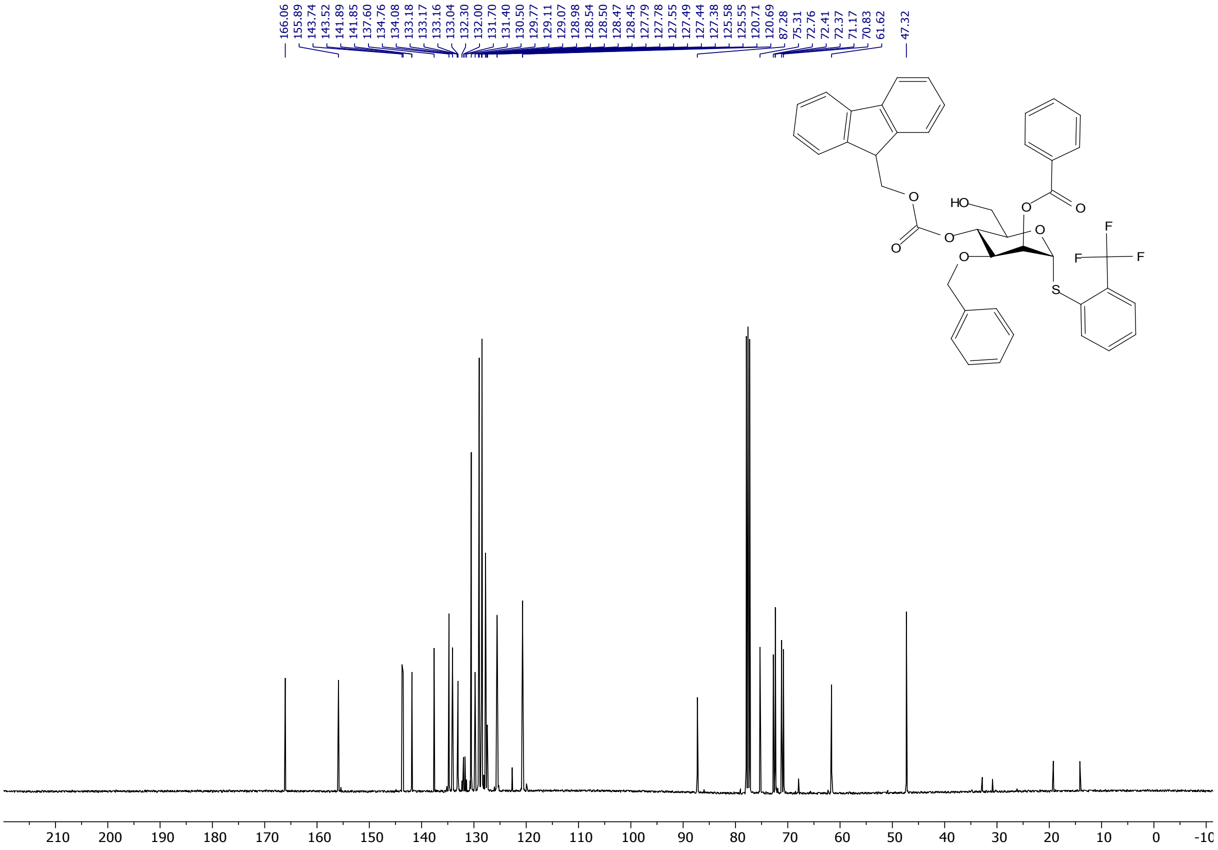
166.06
155.89
143.74
143.52
141.89
141.85
137.60
134.76
134.08
133.18
133.17
133.16
133.04
132.30
132.00
131.70
131.40
130.50
129.77
129.11
129.07
128.98
128.54
128.50
128.47
128.45
127.79
127.78
127.55
127.49
127.44
127.38
125.58
125.55
120.71
120.69
87.28
75.31
72.76
72.41
72.37
71.17
70.83
61.62
47.32
HO
O
F
F
F
S

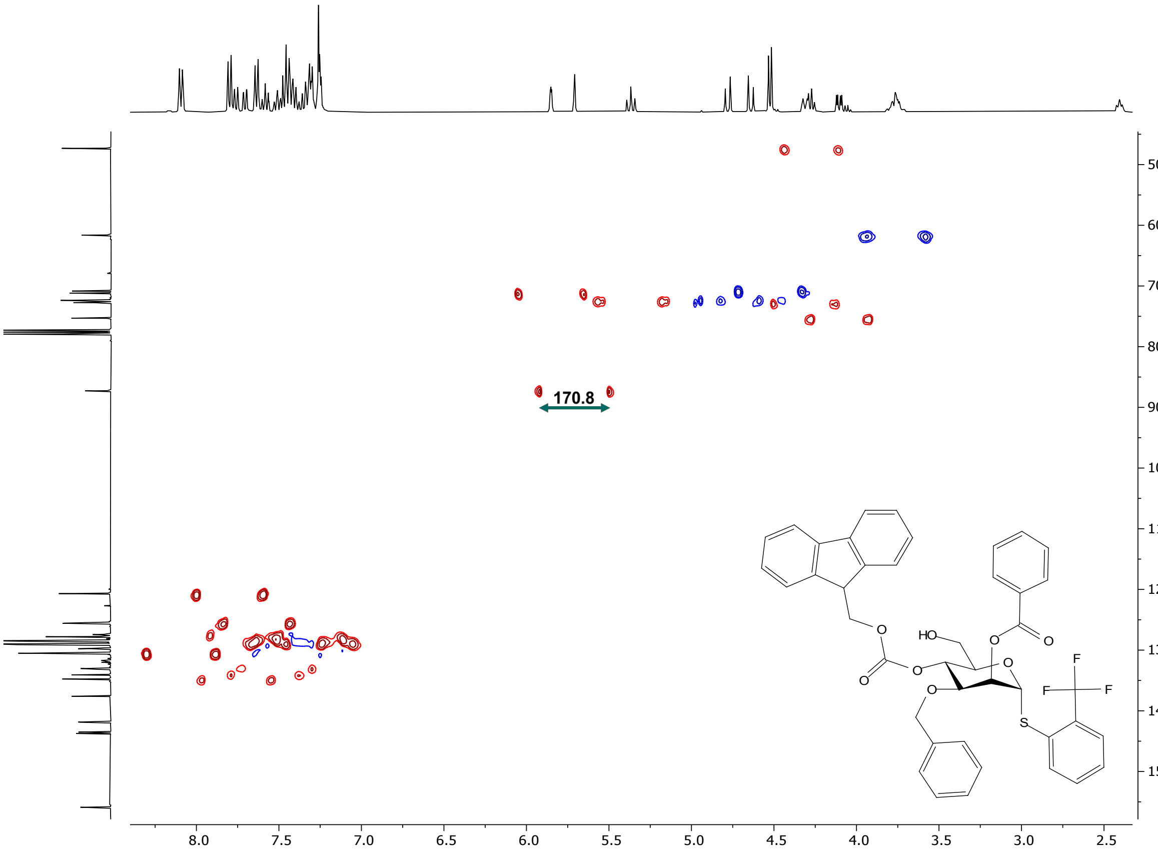
170.8
HO
O
F
F
F
S

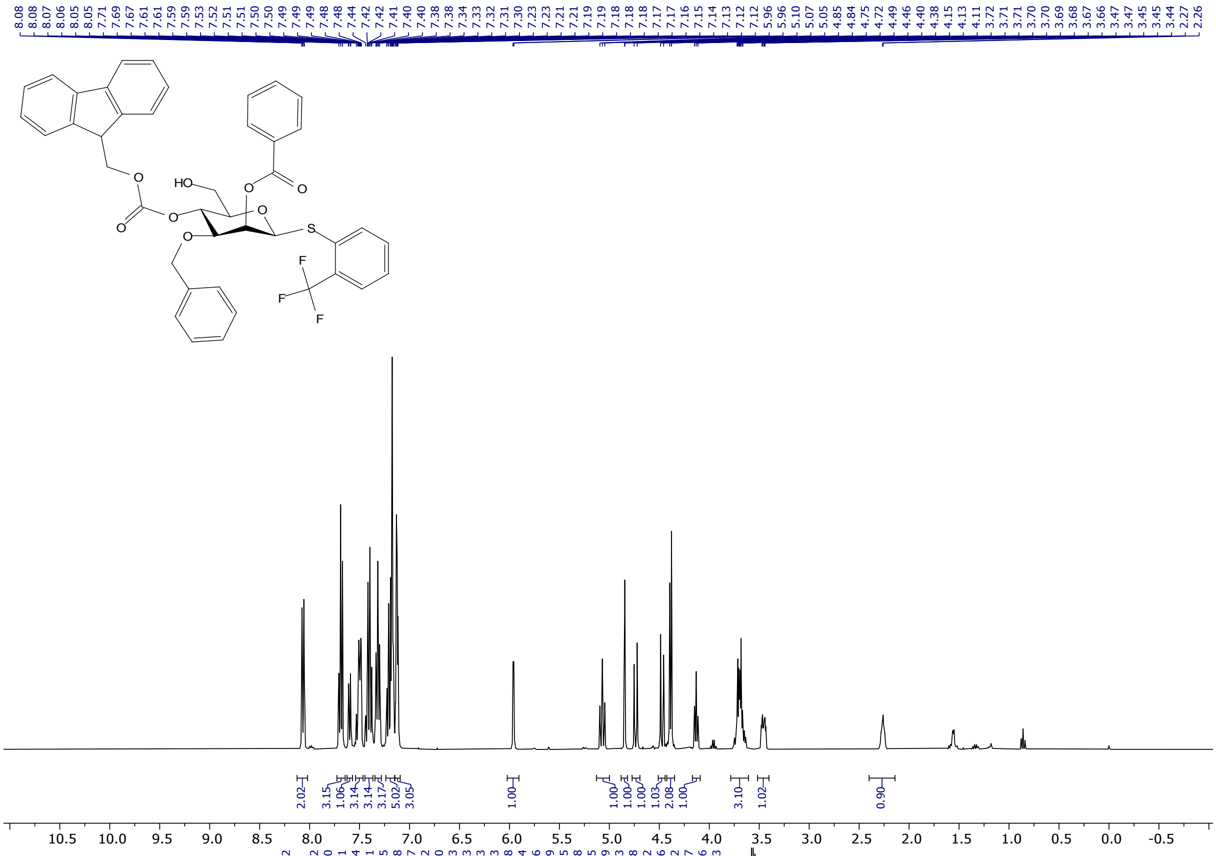

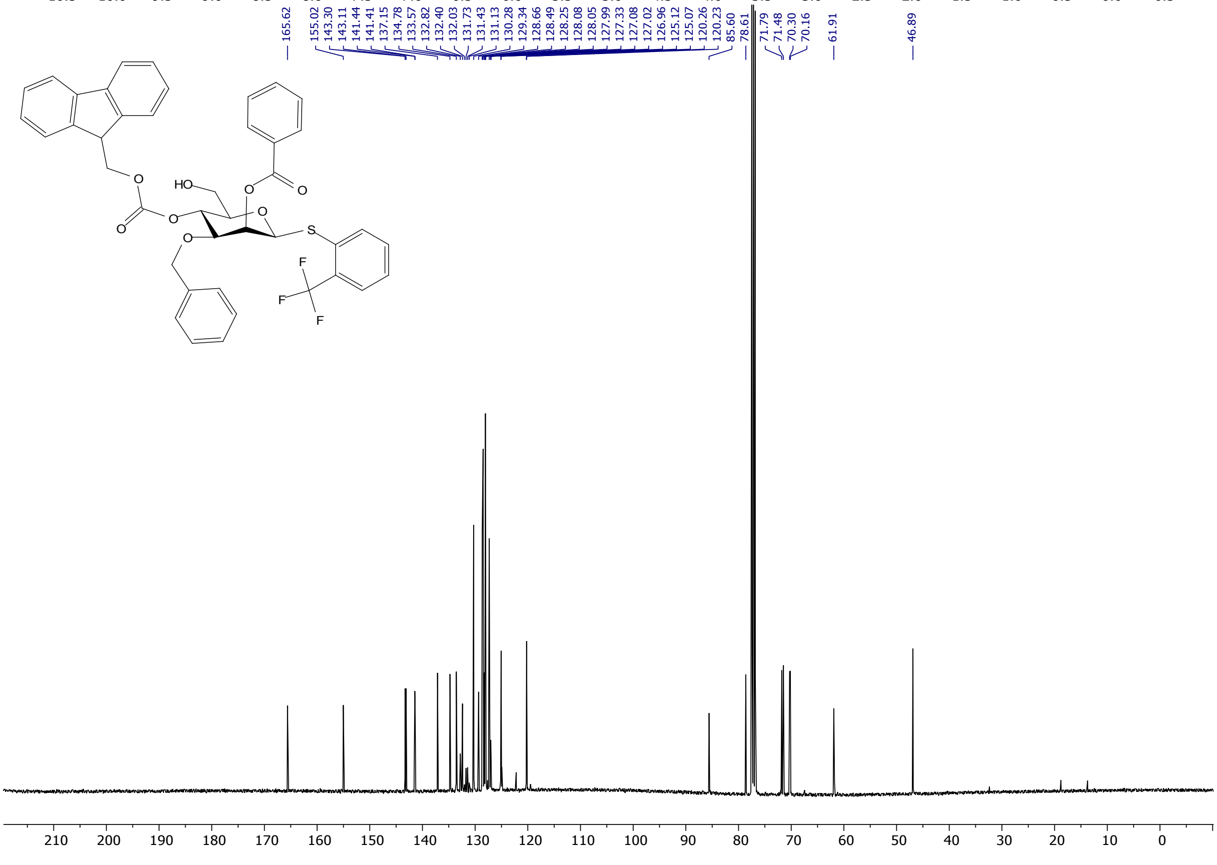

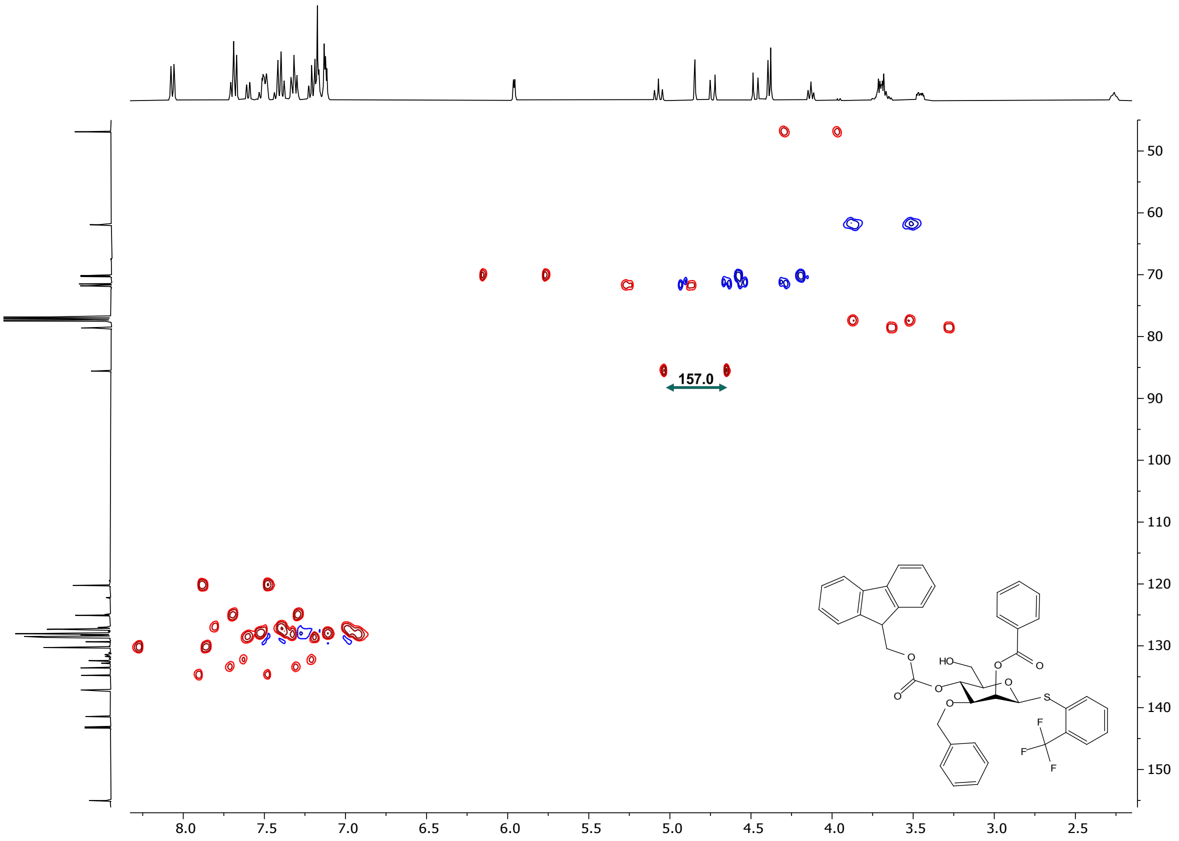
157.0
HO
O
O
O
O
O
O
O
S
F
F
F
8.0
7.5
7.0
6.5
6.0
5.5
5.0
4.5
4.0
3.5
3.0
2.5
50
60
70
80
90
100
110
120
130
140
150

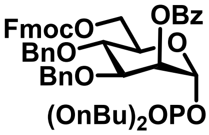

Dibutly 2-*O*-benzoyl-3,4-di-*O*-benzyl-6-*O*-(9-fluorenylmethoxycarbonyl)-1-phosphate-*α*-D-mannopyranoside (14)

NIS (3.35 g, 14.9 mmol, 1.2 eq.) was added to a mixture of thioglycoside **7** (9.83 g, 12.4 mmol, 1 eq.), dibutyl phosphate (6.2 mL, 31 mmol, 2.5 eq.) and activated MS (3 Å) in anhydrous CH_2Cl_2 (0.1 M) at 0 °C under nitrogen. The reaction was stirred for 15 min before TfOH (330 µL, 3.72 mmol, 0.3 eq.) was added dropwise and stirred for 1 h. The reaction was stopped by addition of sat. aq. $NaHCO_3$, the phases were separated and the aqueous phase was extracted three times with CH_2Cl_2 (100 mL). The combined organic phases were washed with sat. aq. $NaHSO_3$, dried over Na_2SO_4 and the solvent was removed under reduced pressure. **14** was isolated by FCC (3:2:5 EtOAc/CH_2Cl_2/hexanes; Rf = 0.29) in 93% yield as a colorless oil (10.1 g, 11.5 mmol).

^{1}H NMR (400 MHz, $CDCl_3$) *δ* 8.15 – 8.10 (m, 2H), 7.80 – 7.75 (m, 2H), 7.66 – 7.59 (m, 2H), 7.58 – 7.50 (m, 1H), 7.41 (td, *J* = 7.6, 6.0 Hz, 4H), 7.36 – 7.26 (m, 12H), 5.80 (dd, *J* = 6.5, 2.2 Hz, 1H), 5.72 (t, *J* = 2.6 Hz, 1H), 4.94 (d, *J* = 10.9 Hz, 1H), 4.84 (d, *J* = 11.2 Hz, 1H), 4.62 (dd, *J* = 11.0, 8.8 Hz, 2H), 4.50 – 4.36 (m, 4H), 4.26 (t, *J* = 7.5 Hz, 1H), 4.19 (dd, *J* = 9.4, 2.9 Hz, 1H), 4.16 – 3.99 (m, 6H), 1.72 – 1.62 (m, 4H), 1.42 (hd, *J* = 7.4, 5.7 Hz, 4H), 0.94 (q, *J* = 7.3 Hz, 6H).

^{13}C NMR (101 MHz, $CDCl_3$) *δ* 165.3, 155.2, 143.5, 143.3, 141.4, 137.8, 137.6, 133.6, 130.1, 129.5, 128.7, 128.6, 128.5, 128.3, 128.3, 128.0, 128.0, 127.3, 127.3, 125.3, 125.2, 120.2, 95.5, 95.4, 75.5, 73.1, 71.9, 71.6, 70.2, 68.3, 68.2, 68.2, 68.1, 66.4, 46.8, 32.4, 32.3, 32.3, 32.3, 18.7, 13.7.

^{31}P NMR (162 MHz, $CDCl_3$) *δ* -2.9.

HRMS (ESI): $C_{50}H_{55}NaO_{12}P$ $[M+Na]^+$; calculated: 901.3329, found: 901.3386.

Optical rotation: $[\alpha]_D^{25}$ = +7.5° (c = 1.0, $CHCl_3$)

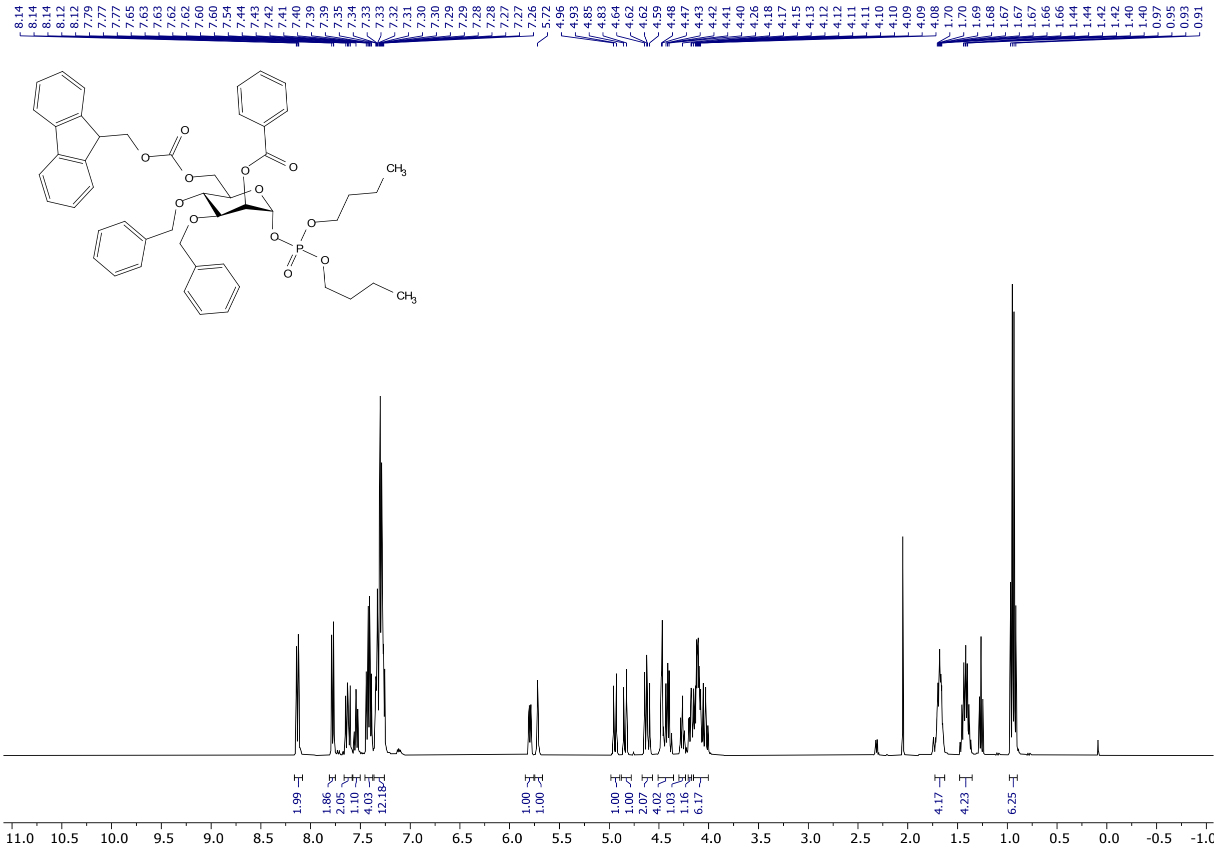
8.14 8.14 8.14 8.12 7.79 7.77 7.77 7.65 7.63 7.63 7.62 7.62 7.60 7.60 7.54 7.44 7.43 7.42 7.41 7.40 7.39 7.39 7.35 7.34 7.33 7.33 7.32 7.31 7.30 7.30 7.29 7.29 7.28 7.28 7.27 7.27 7.26 5.72 4.96 4.93 4.85 4.83 4.64 4.62 4.62 4.59 4.48 4.47 4.43 4.42 4.41 4.40 4.26 4.18 4.17 4.15 4.13 4.12 4.12 4.11 4.11 4.10 4.10 4.09 4.09 4.08 1.70 1.70 1.69 1.68 1.67 1.67 1.67 1.66 1.66 1.44 1.44 1.42 1.42 1.40 1.40 0.97 0.95 0.93 0.91
CH3
CH3
P
1.99 1.86 2.05 1.10 4.03 12.18 1.00 1.00 1.00 1.00 2.07 4.02 1.03 1.16 6.17 4.17 4.23 6.25
11.0 10.5 10.0 9.5 9.0 8.5 8.0 7.5 7.0 6.5 6.0 5.5 5.0 4.5 4.0 3.5 3.0 2.5 2.0 1.5 1.0 0.5 0.0 -0.5 -1.0

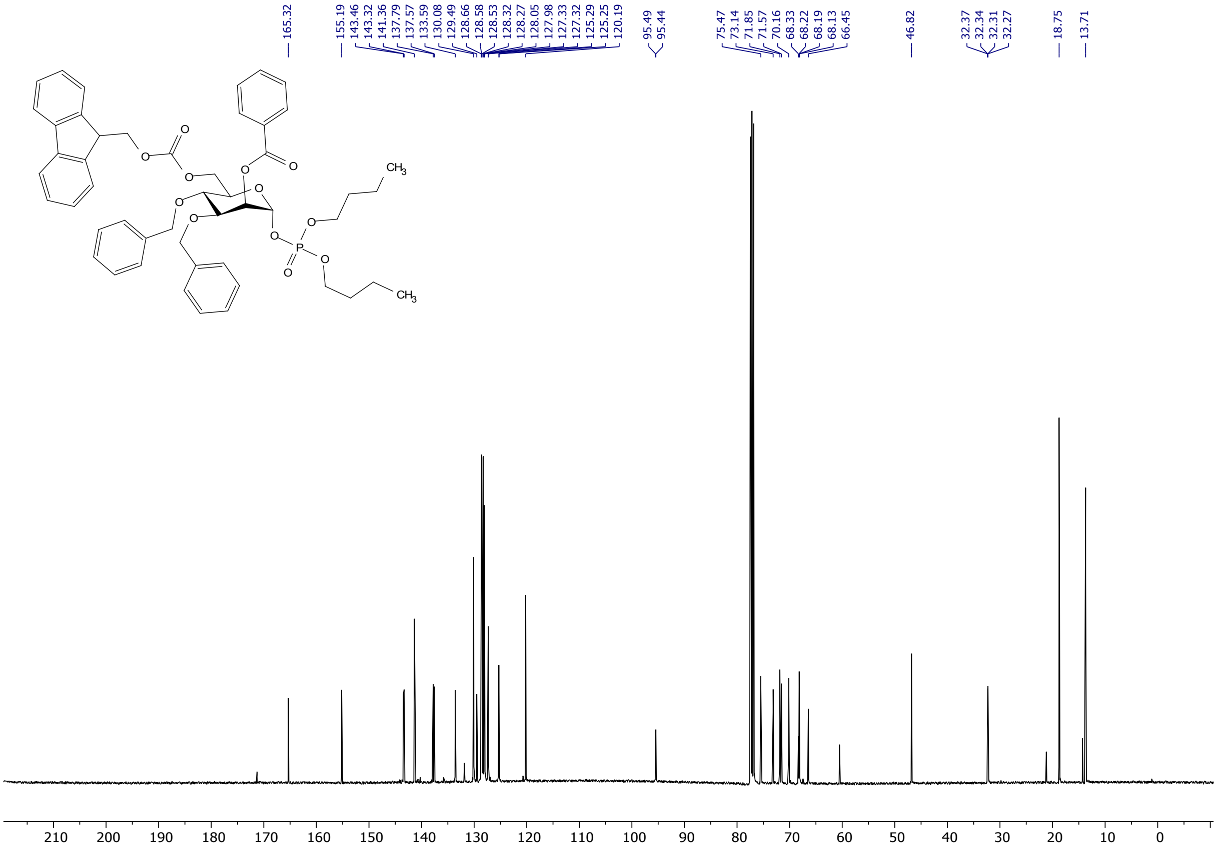
165.32 155.19 143.46 143.32 141.36 137.79 137.57 133.59 130.08 129.49 128.66 128.58 128.53 128.32 128.27 128.05 127.98 127.33 127.32 125.29 125.25 120.19 95.49 95.44 75.47 73.14 71.85 71.57 70.16 68.33 68.22 68.19 68.13 66.45 46.82 32.37 32.34 32.31 32.27 18.75 13.71
CH3
CH3
P
210 200 190 180 170 160 150 140 130 120 110 100 90 80 70 60 50 40 30 20 10 0

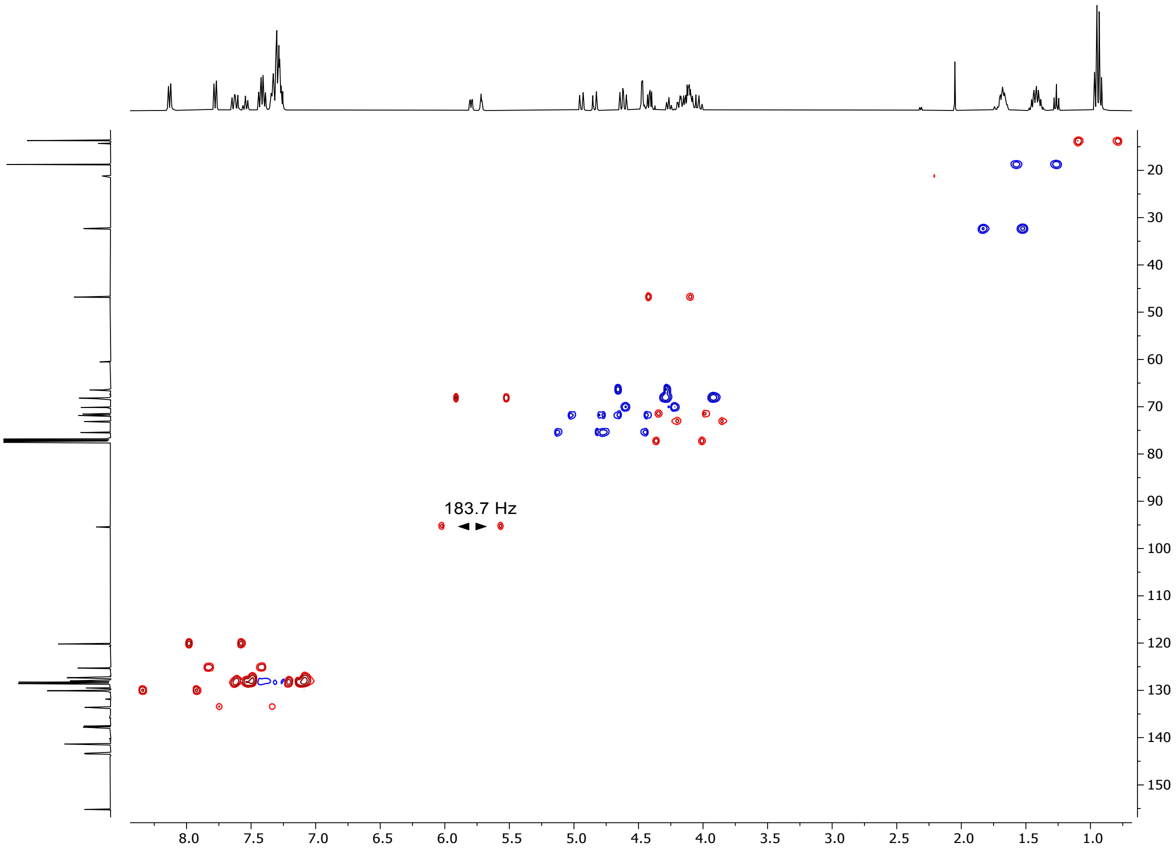
183.7 Hz

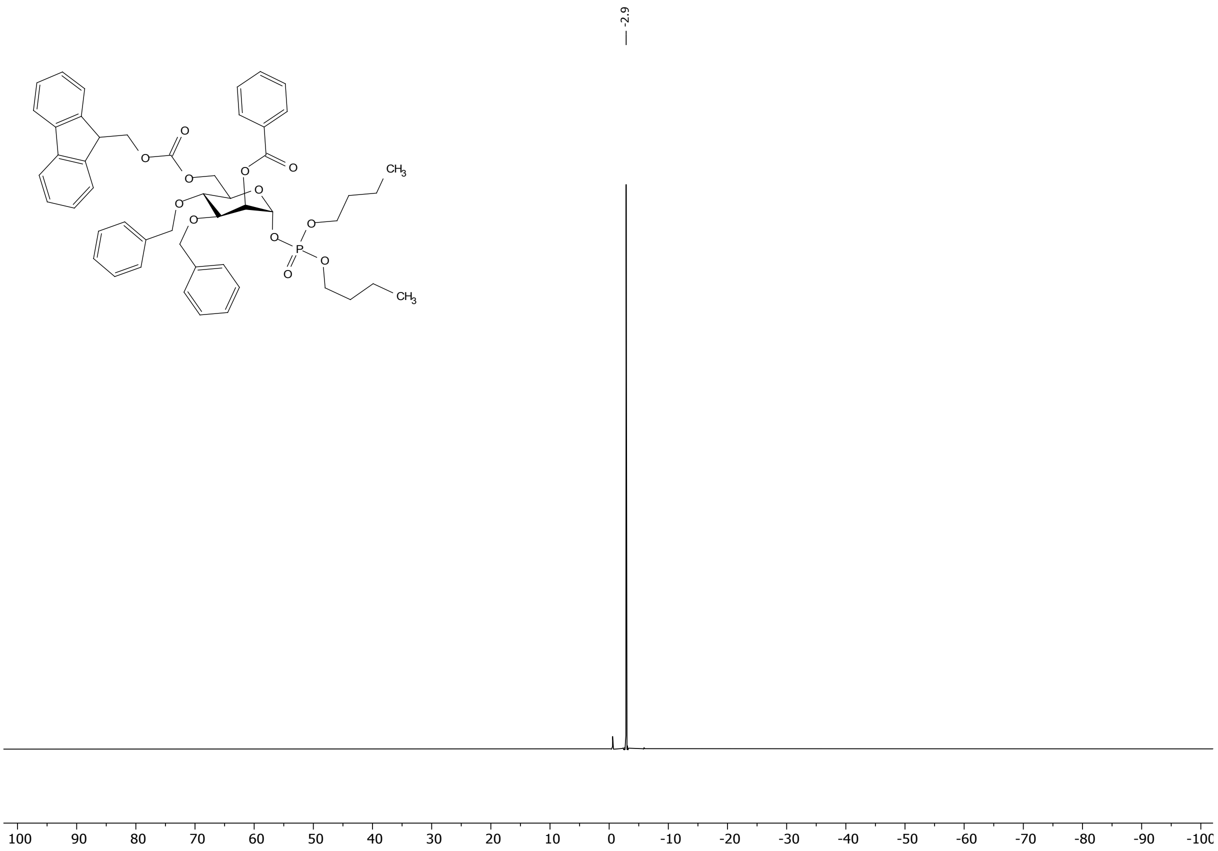
-2.9
CH3
O
P

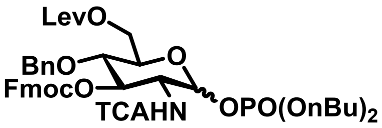

Dibutyl 4-O-benzyl-2-deoxy-2-[(2,2,2,-trichloroacetyl)amino]-6-O-levulinoyl-3-O-(9-fluorenylmethoxycarbonyl)-1-phosphate-*α,β*-D-glucopyranoside (39)

NIS (694 mg, 3.08 mmol, 1.2 eq.) was added to thioglycoside **SI20** (2.00 g, 2.57 mmol, 1 eq.), dibutyl phosphate (1.28 mL, 6.42 mmol, 2.5 eq.) and activated MS (3 Å) in anhydrous CH_2Cl_2 (0.1 M) at 0 °C under nitrogen. The reaction was stirred for 15 min before TfOH (70 µL, 0.77 mmol, 0.3 eq.) was added dropwise and stirred for 1 h. The reaction was stopped by addition of sat. aq. $NaHCO_3$, the phases were separated and the aqueous phase was extracted three times with CH_2Cl_2 (100 mL). The combined organic phases were washed with sat. aq. NaH_SO_3, dried over Na_2SO_4 and the solvent was removed under reduced pressure. **39** was isolated by FCC (5:1:4 EtOAc/CH_2Cl_2/hexanes; Rf = 0.16) in 90% yield as a mixture of anomers and a colorless resin (2.14 g, 2.31 mmol).

^{1}H NMR (600 MHz, $CDCl_3$) *δ* 7.77 – 7.68 (m, 5H), 7.56 (dddt, *J* = 12.9, 6.4, 4.5, 1.0 Hz, 4H), 7.41 – 7.26 (m, 14H), 7.26 – 7.20 (m, 5H), 5.76 (dd, *J* = 6.0, 3.3 Hz, 1H), 5.43 (t, *J* = 7.9 Hz, 1H), 5.23 (ddd, *J* = 26.7, 10.8, 9.0 Hz, 2H), 4.77 (d, *J* = 11.2 Hz, 1H), 4.66 (d, *J* = 11.0 Hz, 1H), 4.63 (d, *J* = 11.2 Hz, 1H), 4.53 (d, *J* = 11.0 Hz, 1H), 4.44 (dd, *J* = 10.5, 7.4 Hz, 1H), 4.41 – 4.16 (m, 12H), 4.15 – 4.02 (m, 8H), 4.02 – 3.92 (m, 2H), 3.85 (dd, *J* = 10.1, 9.2 Hz, 1H), 3.74 (t, *J* = 9.4 Hz, 1H), 3.62 (ddd, *J* = 9.9, 4.0, 2.3 Hz, 1H), 2.83 – 2.71 (m, 2H), 2.70 – 2.61 (m, 2H), 2.61 – 2.56 (m, 2H), 2.55 – 2.50 (m, 2H), 2.21 (s, 3H), 2.18 (s, 3H), 1.70 – 1.54 (m, 8H), 1.44 – 1.29 (m, 8H), 0.96 – 0.87 (m, 12H).

^{13}C NMR (101 MHz, $CDCl_3$) δ 206.5, 206.3, 172.4, 172.4, 162.5, 162.3, 155.7, 155.3, 143.1, 143.1, 143.0, 141.4, 141.4, 141.3, 141.3, 137.1, 136.9, 128.7, 128.7, 128.5, 128.5, 128.3, 128.3, 128.3, 128.2, 128.2, 128.1, 127.4, 127.4, 127.4, 127.3, 125.3, 125.2, 125.2, 120.3, 120.2, 120.2, 120.1, 96.4, 96.4, 95.0, 95.0, 92.4, 91.9, 78.7, 78.7, 76.8, 75.3, 75.1, 74.8, 74.2, 73.7, 71.0, 71.0, 70.9, 68.5, 68.5, 68.4, 68.4, 68.4, 68.3, 68.3, 62.3, 62.0, 56.0, 55.9, 54.7, 54.6, 46.6, 46.6, 37.9, 37.8, 32.4, 32.4, 32.3, 32.3, 32.2, 32.1, 30.0, 30.0, 27.9, 27.8, 18.7, 18.7, 18.7, 13.7, 13.7.

^{31}P NMR (162 MHz, $CDCl_3$) *δ* -2.4, -3.3.

HRMS (ESI): $C_{43}H_{51}Cl_3NNaO_{13}P$ [M+Na]$^+$; calculated: 948.2061, found: 948.2122.

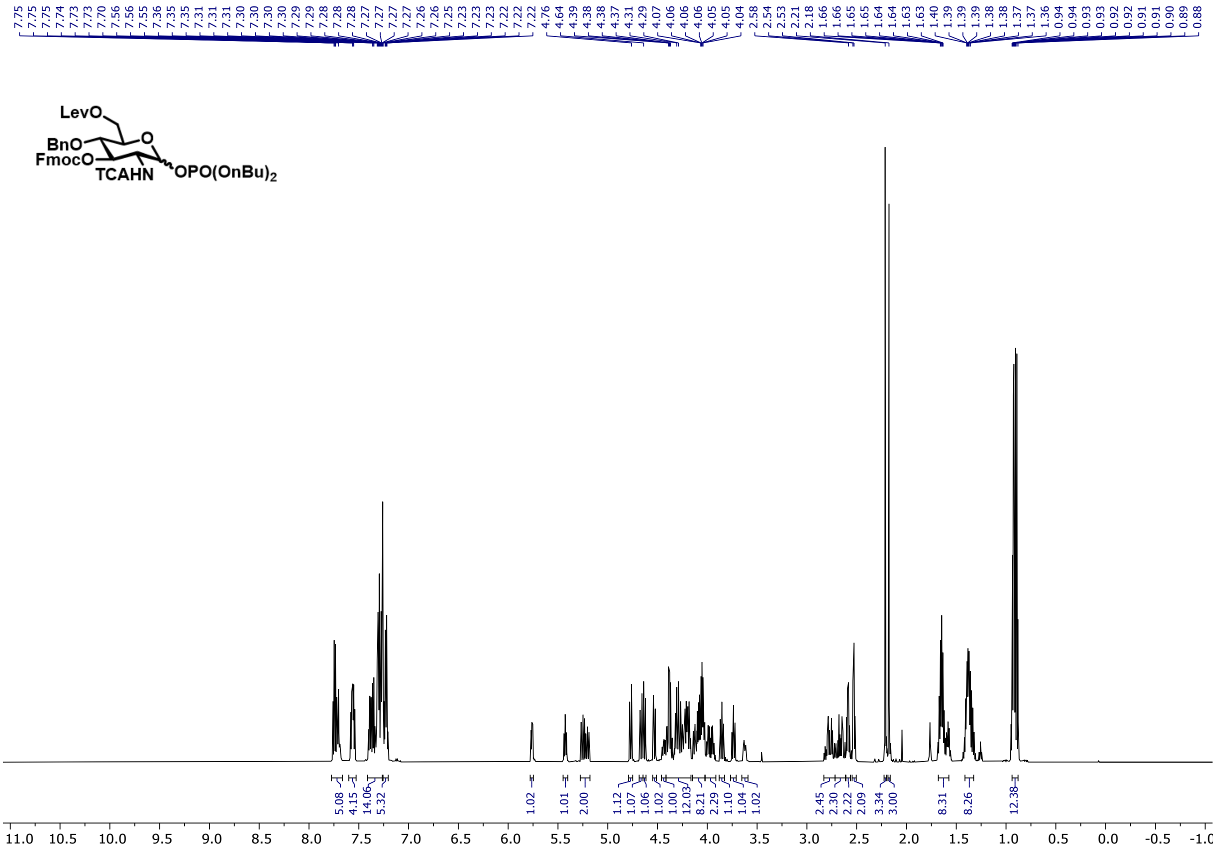

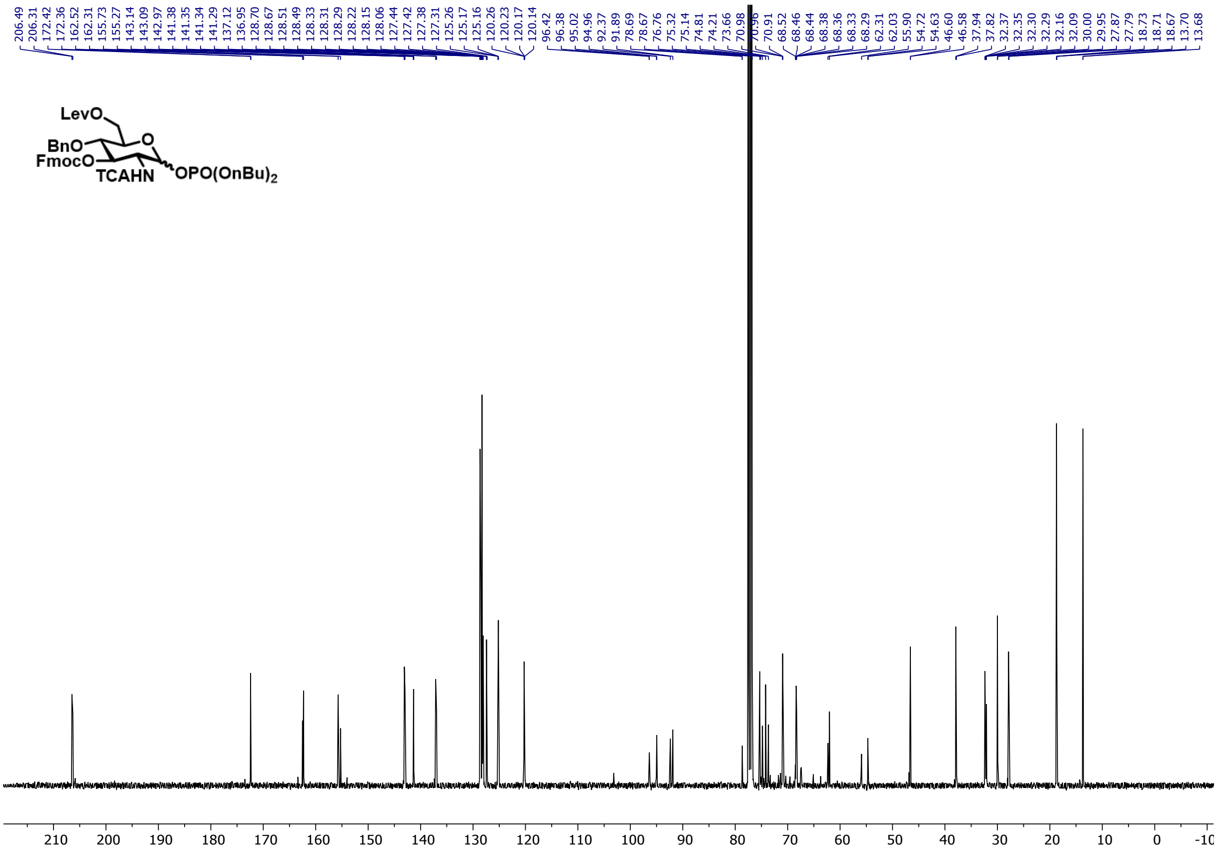

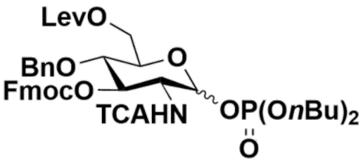
LevO
BnO
FmocO
TCAHN
O
OP(OnBu)2
O

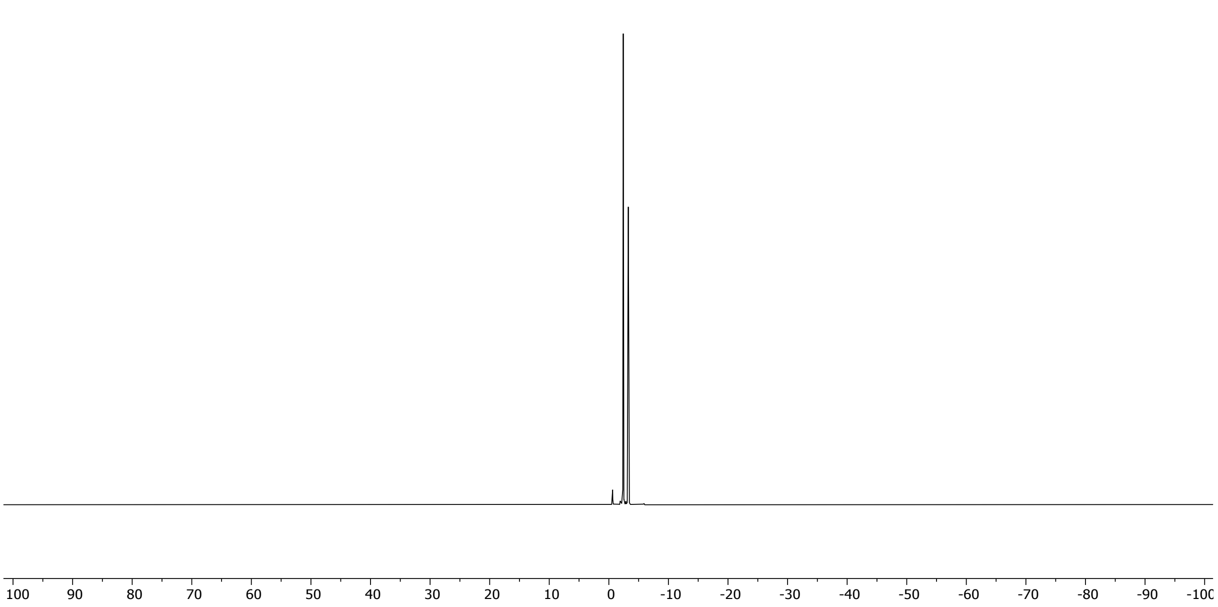
-2.42
-3.27
100
90
80
70
60
50
40
30
20
10
0
-10
-20
-30
-40
-50
-60
-70
-80
-90
-100

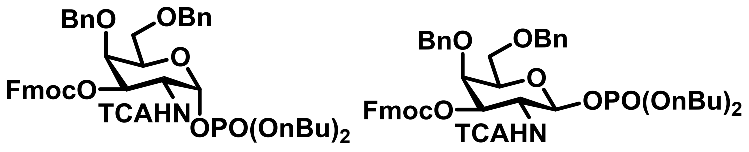

Dibutyl 4,6-di-O-benzyl-2-deoxy-2-[(2,2,2,-trichloroacetyl)amino]-3-O-(9-fluorenylmethoxycarbonyl)-1-phosphate-*α*,*β*-D-galactopyranoside (40)

NIS (700 mg, 3.11 mmol, 1.2 eq.) was added to a mixture of thioglycoside **SI21** (2.00 g, 2.59 mmol, 1 eq.), dibutyl phosphate (1.29 mL, 6.48 mmol, 2.5 eq.) and activated MS (3 Å) in anhydrous CH_2Cl_2 (0.1 M) at 0 °C under nitrogen. The reaction was stirred for 15 min before TfOH (70 µL, 0.77 mmol, 0.3 eq.) was added dropwise and stirred for 1 h. The reaction was stopped by addition of sat. aq. $NaHCO_3$, the phases were separated and the aqueous phase was extracted with CH_2Cl_2 (100 mL). The combined organic phases were washed with sat. aq. NaH_SO_3, dried over Na_2SO_4 and the solvent was removed under reduced pressure. **40** was isolated by FCC (5:1:4 EtOAc/CH_2Cl_2/hexanes; Rf = 0.2(*α*), 0.4 (*β*)) in 92% yield as a colorless foam (2.20 g, 2.39 mmol).

***α* - ^{1}H NMR** (400 MHz, $CDCl_3$) *δ* 7.75 (t, *J* = 6.8 Hz, 2H), 7.58 (d, *J* = 7.5 Hz, 2H), 7.40 (td, *J* = 7.5, 4.9 Hz, 2H), 7.38 – 7.34 (m, 6H), 7.34 – 7.27 (m, 6H), 7.07 (d, *J* = 9.0 Hz, 1H), 5.80 (dd, *J* = 5.7, 3.4 Hz, 1H), 5.18 (dd, *J* = 11.2, 2.7 Hz, 1H), 4.89 – 4.80 (m, 2H), 4.57 (d, *J* = 11.2 Hz, 1H), 4.50 (d, *J* = 11.7 Hz, 1H), 4.45 (d, *J* = 11.7 Hz, 1H), 4.44 – 4.36 (m, 2H), 4.29 (dd, *J* = 7.6, 5.7 Hz, 1H), 4.24 (t, *J* = 7.4 Hz, 1H), 4.14 (dd, *J* = 2.8, 1.3 Hz, 1H), 4.13 – 4.00 (m, 4H), 3.65 (dd, *J* = 9.1, 7.6 Hz, 1H), 3.58 (dd, *J* = 9.1, 5.7 Hz, 1H), 1.62 (dqd, *J* = 10.1, 6.6, 3.6 Hz, 4H), 1.36 (hd, *J* = 7.4, 2.2 Hz, 4H), 0.92 (td, *J* = 7.4, 2.9 Hz, 6H).

***α* - ^{13}C NMR** (101 MHz, $CDCl_3$) *δ* 162.2, 155.4, 143.1, 143.0, 141.4, 141.4, 137.7, 137.6, 128.6, 128.5, 128.1, 128.1, 128.0, 128.0, 127.4, 127.3, 125.3, 125.2, 120.3, 120.2, 96.1, 96.0, 92.2, 75.4, 74.5, 73.6, 73.5, 71.4, 70.8, 68.3, 68.2, 68.2, 68.2, 67.8, 50.9, 50.8, 46.6, 32.3, 32.3, 32.3, 32.2, 18.7, 13.7.

***β* - ¹H NMR** (400 MHz, $CDCl_3$) *δ* 7.73 (ddd, *J* = 7.6, 5.5, 1.1 Hz, 2H), 7.56 (ddd, *J* = 7.6, 4.1, 1.0 Hz, 2H), 7.42 – 7.22 (m, 14H), 5.48 – 5.33 (m, 1H), 5.12 – 4.99 (m, 1H), 4.81 – 4.72 (m, 1H), 4.57 (dd, *J* = 11.9, 8.0 Hz, 1H), 4.53 – 4.43 (m, 2H), 4.36 (qdd, *J* = 10.3, 7.2, 2.9 Hz, 3H), 4.21 (t, *J* = 7.3 Hz, 1H), 4.11 – 3.91 (m, 3H), 3.86 – 3.78 (m, 1H), 3.66 – 3.52 (m, 2H), 1.63 – 1.54 (m, 4H), 1.40 – 1.29 (m, 4H), 0.92 – 0.83 (m, 6H).

***β* - ¹³C NMR** (101 MHz, $CDCl_3$) *δ* 162.3, 154.8, 143.1, 143.0, 141.3, 141.3, 137.6, 128.5, 128.4, 128.4, 128.0, 128.0, 127.9, 127.7, 127.3, 127.2, 125.2, 125.2, 120.1, 120.1, 96.7, 75.2, 74.1, 73.5, 72.8, 70.5, 68.4, 68.3, 68.2, 68.1, 46.6, 32.1, 32.1, 32.0, 32.0, 18.6, 18.6, 13.6.

HRMS (ESI): $C_{45}H_{51}Cl_3NNaO_{11}P$ $[M+Na]^+$; calculated: 940.2163, found: 940.2242.

***α* - ³¹P NMR** (162 MHz, $CDCl_3$) *δ* -2.3.

Optical rotation: *α*-anomer $[\alpha]_D^{25}$ = +44.6° (c = 1.0, $CHCl_3$)

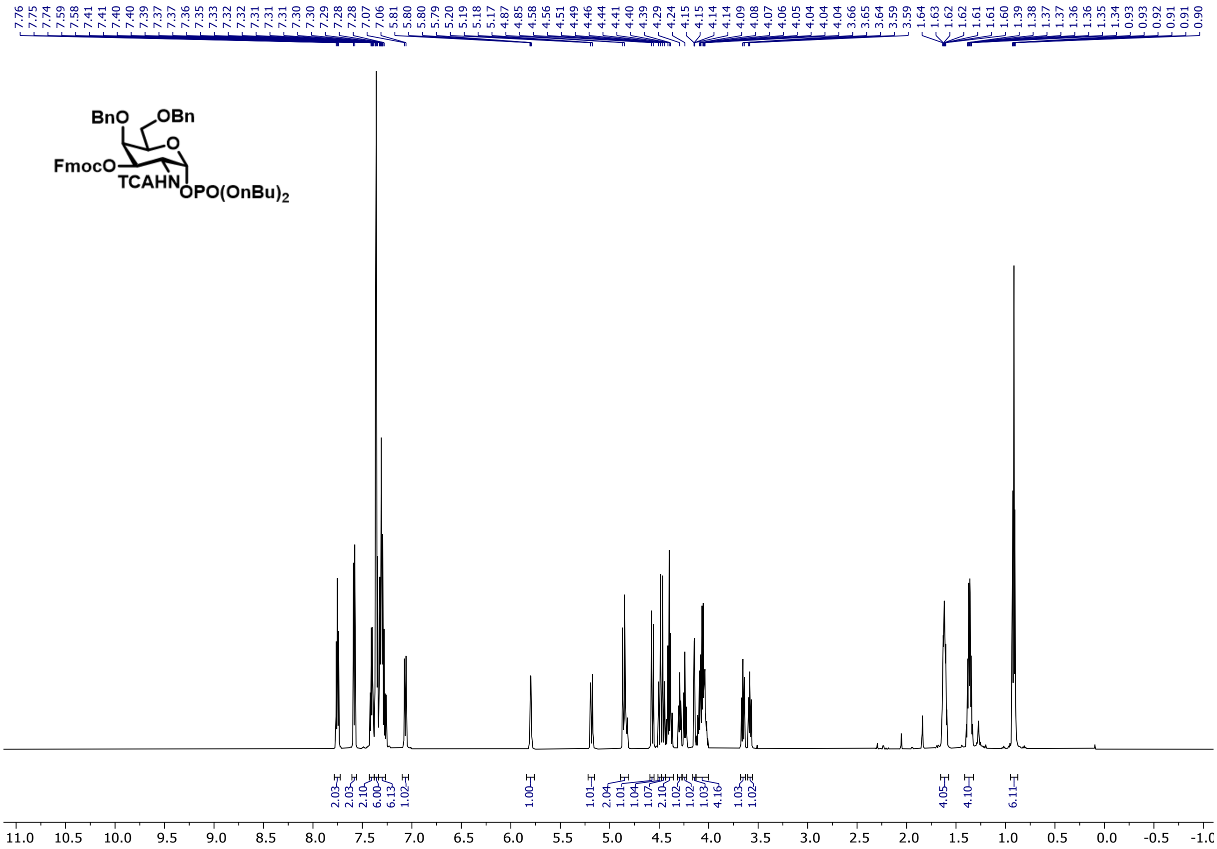

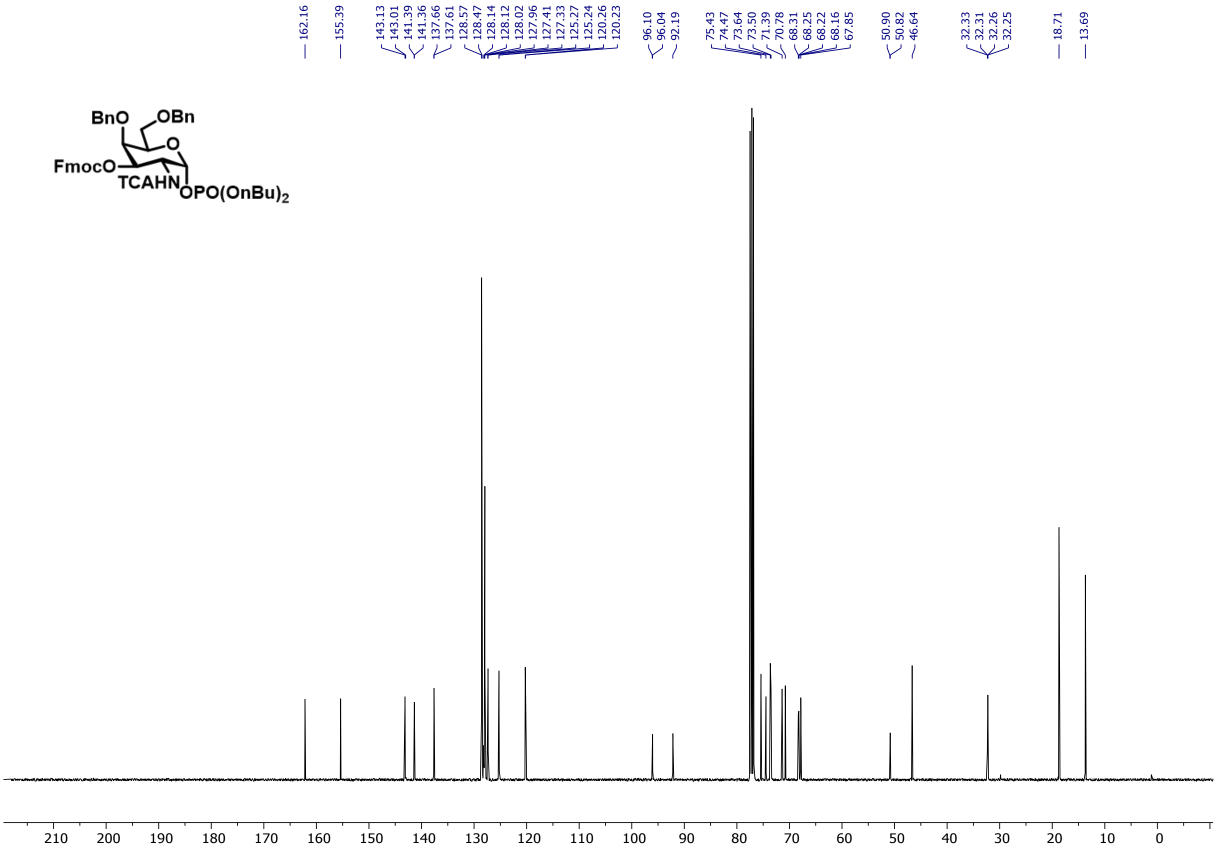
BnO OBn
O
FmocO
TCAHN
OPO(OnBu)2
162.16
155.39
143.13
143.01
141.39
141.36
137.66
137.61
128.57
128.47
128.14
128.12
128.02
127.96
127.41
127.33
125.27
125.24
120.26
120.23
96.10
96.04
92.19
75.43
74.47
73.64
73.50
71.39
70.78
68.31
68.25
68.22
68.16
67.85
50.90
50.82
46.64
32.33
32.31
32.26
32.25
18.71
13.69
210 200 190 180 170 160 150 140 130 120 110 100 90 80 70 60 50 40 30 20 10 0

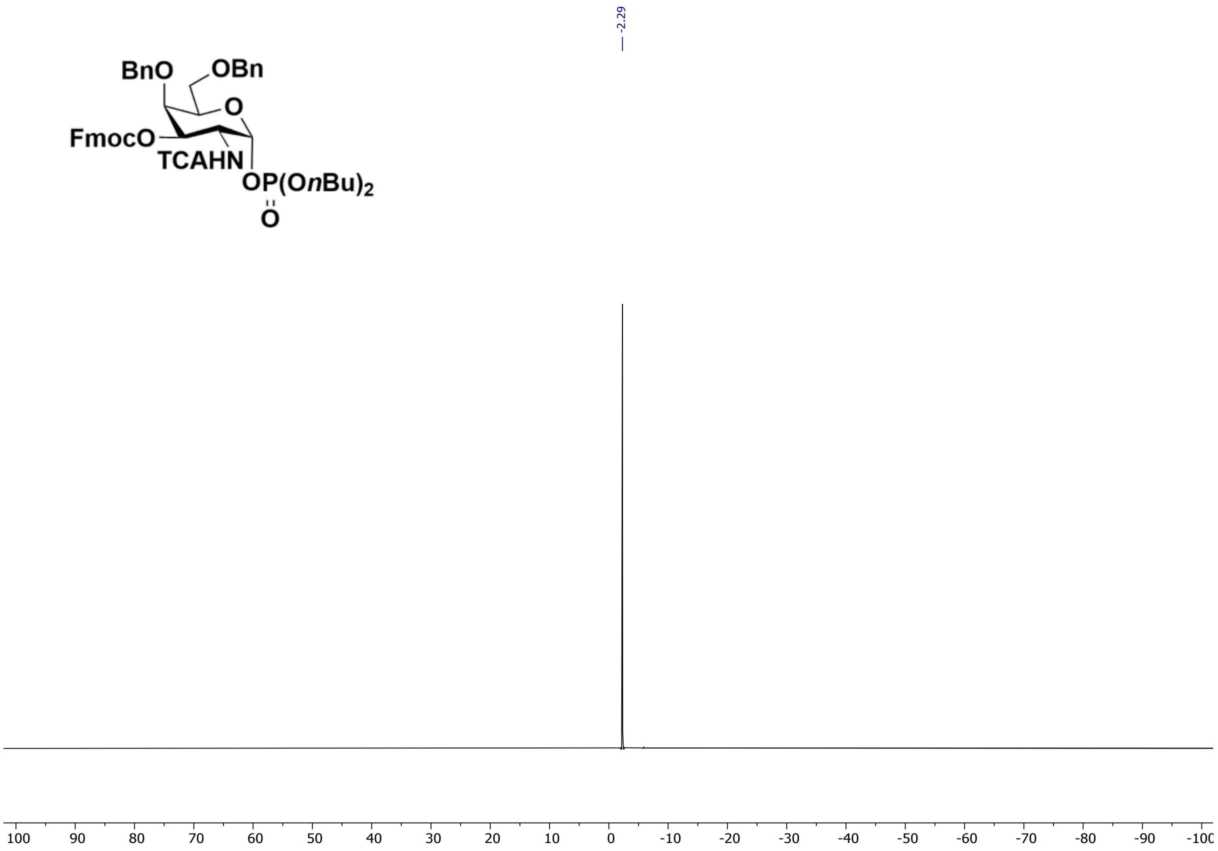
BnO OBn
O
FmocO
TCAHN
OP(OnBu)2
O
-2.29
100 90 80 70 60 50 40 30 20 10 0 -10 -20 -30 -40 -50 -60 -70 -80 -90 -100

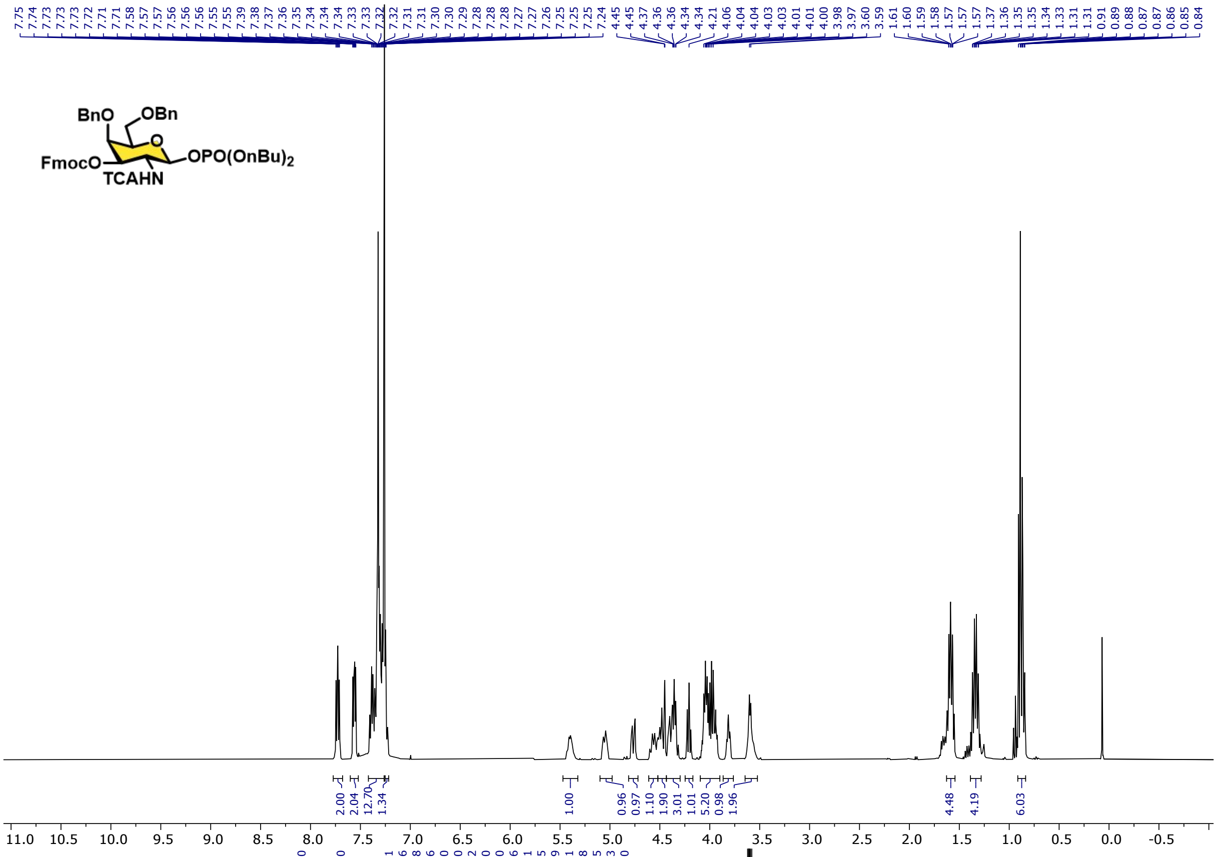

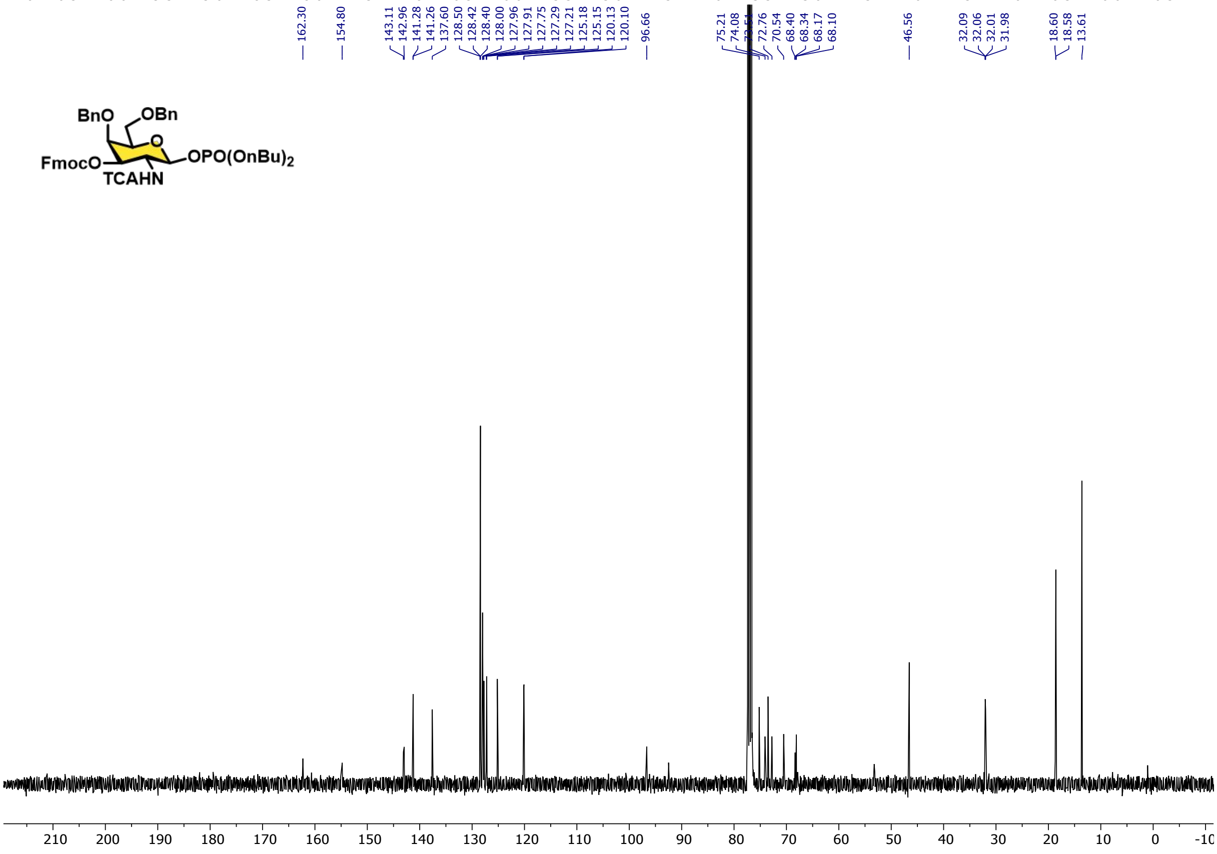

2.2 Nucleophiles

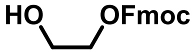

(9H-Fluoren-9-yl)methyl (2-hydroxyethyl) carbonate (8)

FmocCl (4.00 g, 15.2 mmol, 1 eq.) in anhydrous CH_2Cl_2 (0.1 M) under nitrogen was added to ethylene glycol (15 mL, 268 mmol, 17.3 eq.) followed by dropwise addition of and pyridine (6.3 mL, 77.5 mmol, 5 eq.). The reaction was stirred for 1 h at 23 °C and then warmed to 40 °C and stirred another 30 min. The reaction was diluted with CH_2Cl_2 and washed with sat. aq. NH_4Cl. The phases were separated and the aqueous phase was extracted three times with CH_2Cl_2 (100 mL). The combined organic phases were dried over Na_2SO_4 and the solvent was removed under reduced pressure. **8** was isolated by FCC (1:1 EtOAc/ hexanes; Rf = 0.3) in 98% yield as a colorless foam (4.31 g, 15.2 mmol).

^{1}H NMR (400 MHz, $CDCl_3$) δ 7.78 (d, J = 7.5 Hz, 2H), 7.68 – 7.55 (m, 2H), 7.45 – 7.39 (m, 2H), 7.37 – 7.30 (m, 2H), 4.45 (d, J = 7.3 Hz, 2H), 4.35 – 4.21 (m, 3H), 3.94 – 3.78 (m, 2H), 1.97 (t, J = 6.1 Hz, 1H).

^{13}C NMR (101 MHz, $CDCl_3$) δ 155.5, 143.4, 141.4, 128.1, 127.3, 125.3, 120.2, 70.1, 69.6, 61.2, 46.9.

HRMS (ESI): $C_{17}H_{16}NaO_4$ $[M+Na]^+$; calculated: 307.0946, found: 307.0945.

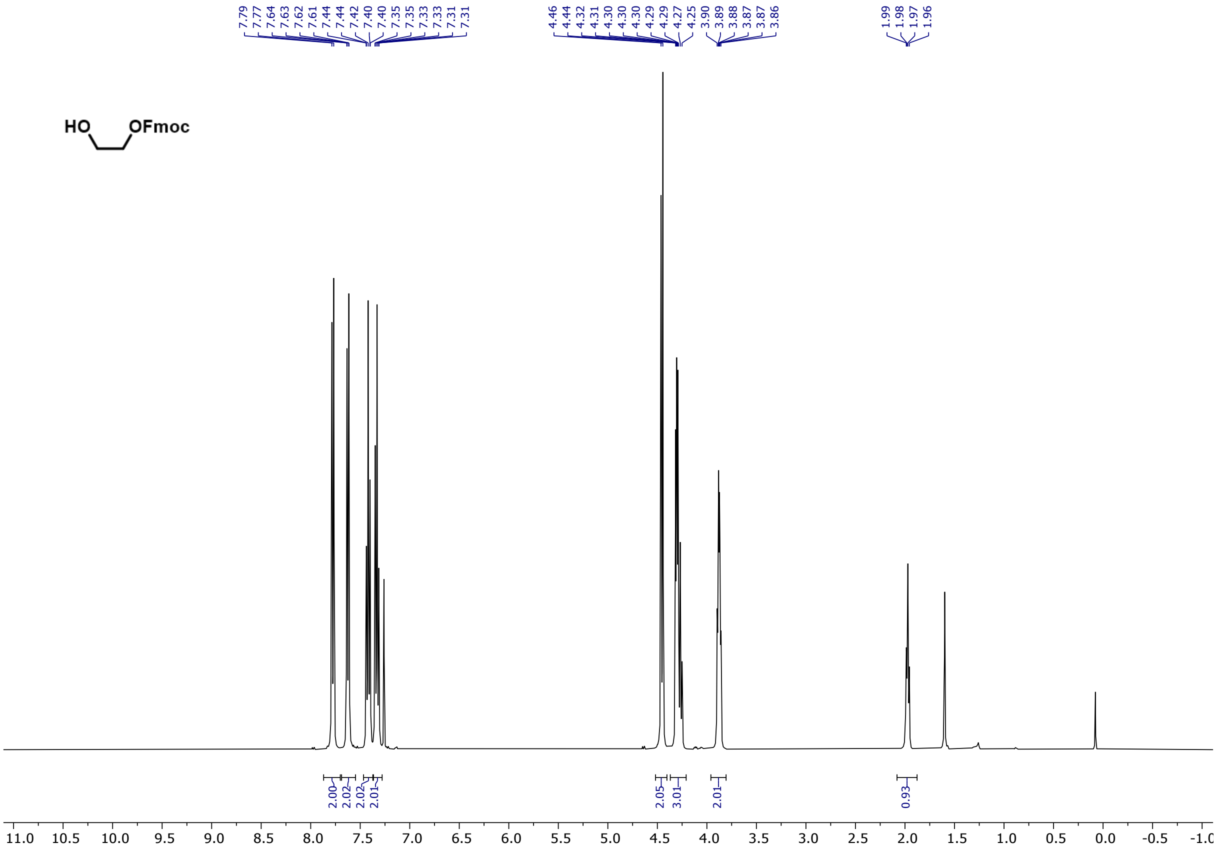

HO OFmoc
7.79 7.77 7.64 7.63 7.62 7.61 7.44 7.44 7.42 7.40 7.40 7.35 7.35 7.33 7.33 7.31 7.31
4.46 4.44 4.32 4.31 4.30 4.30 4.30 4.29 4.29 4.27 4.25 3.90 3.89 3.88 3.87 3.87 3.86
1.99 1.98 1.97 1.96
2.00 2.02 2.02 2.01 2.05 3.01 2.01 0.93
11.0 10.5 10.0 9.5 9.0 8.5 8.0 7.5 7.0 6.5 6.0 5.5 5.0 4.5 4.0 3.5 3.0 2.5 2.0 1.5 1.0 0.5 0.0 -0.5 -1.0

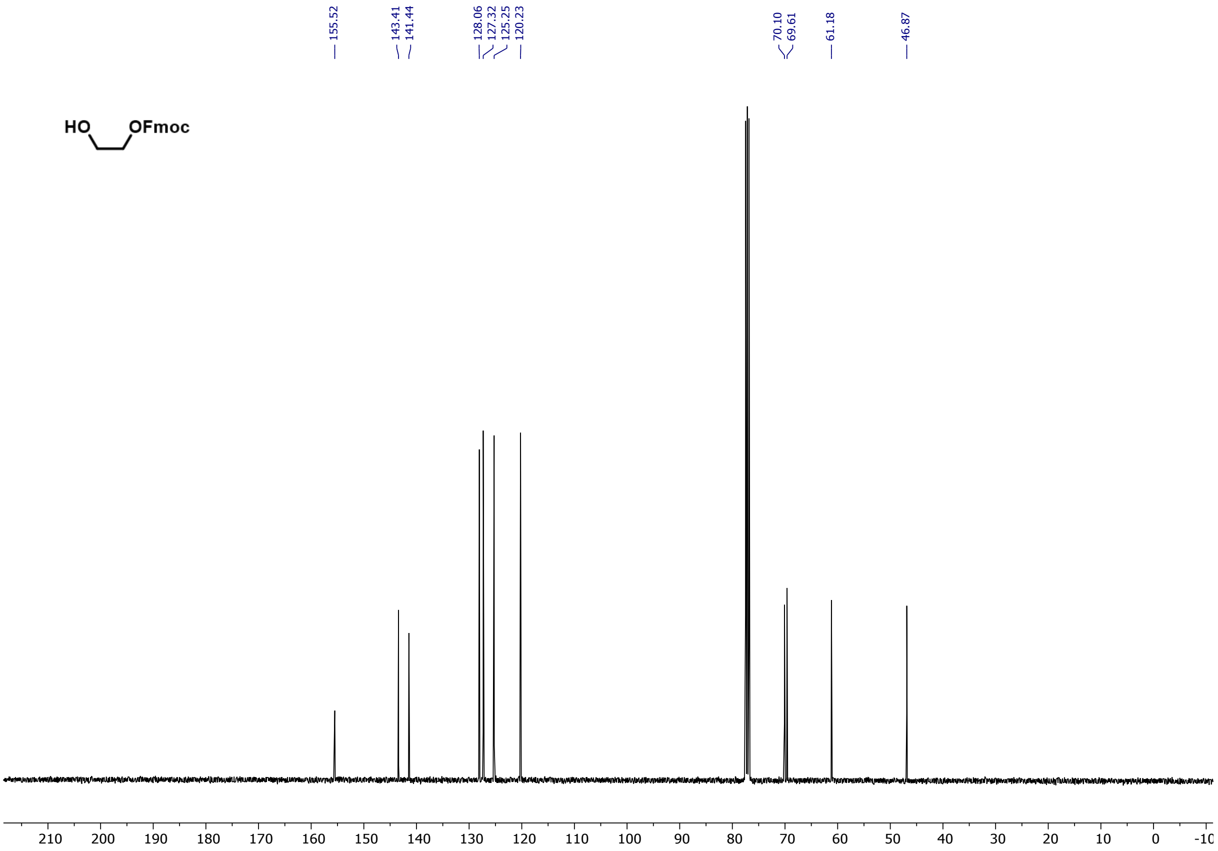

HO OFmoc
155.52
143.41 141.44
128.06 127.32 125.25 120.23
70.10 69.61
61.18
46.87
210 200 190 180 170 160 150 140 130 120 110 100 90 80 70 60 50 40 30 20 10 0 -10

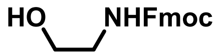

(9H-Fluoren-9-yl)methyl (2-hydroxyethyl)carbamate (29)

FmocCl (4.5 g, 17.4 mmol, 1 eq.) in CH_2Cl_2 was added to ethanolamine (1.15 mL, 19.1 mmol, 1.1 eq.) in anhydrous CH_2Cl_2 (0.1 M) and pyridine (10 mL) under nitrogen at -18 °C. The reaction was allowed to warm to 23 °C and stirred for 1 h. Then the solvent was removed under reduced pressure. **29** was isolated by FCC (3:2 EtOAc/CH_2Cl_2; Rf = 0.3) in 99% yield as a colorless foam (4.5 g, 15.9 mmol).

^{1}H NMR (400 MHz, $CDCl_3$) δ 7.77 (d, J = 7.6 Hz, 2H), 7.67 – 7.50 (m, 2H), 7.40 (tt, J = 7.5, 0.9 Hz, 2H), 7.32 (td, J = 7.5, 1.2 Hz, 2H), 5.19 (t, J = 6.0 Hz, 1H), 4.43 (d, J = 6.8 Hz, 2H), 4.21 (t, J = 6.8 Hz, 1H), 3.71 (q, J = 4.7 Hz, 2H), 3.35 (q, J = 5.3 Hz, 2H), 2.17 (s, 1H).

^{13}C NMR (101 MHz, $CDCl_3$) δ 157.3, 144.0, 141.5, 127.8, 127.2, 125.1, 120.1, 66.9, 62.5, 47.3, 43.6.

HRMS (ESI): $C_{17}H_{17}NNaO_3$ $[M+Na]^+$; calculated: 306.1106, found: 306.1100.

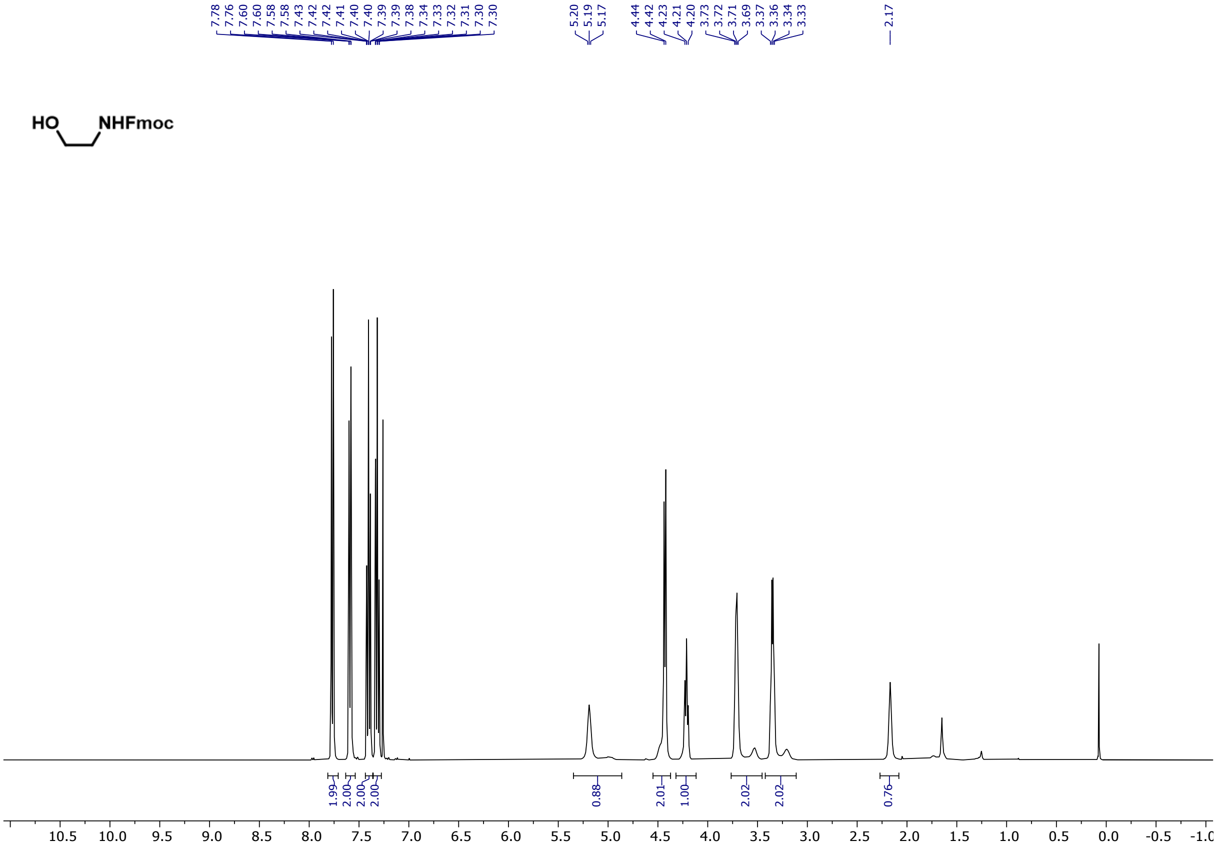
HO NHFmoc
7.78 7.76 7.60 7.58 7.43 7.42 7.42 7.41 7.40 7.39 7.39 7.38 7.34 7.33 7.32 7.31 7.30 7.30
5.20 5.19 5.17
4.44 4.42 4.23 4.21 4.20 3.73 3.72 3.71 3.69 3.37 3.36 3.34 3.33
2.17
1.99 2.00 2.00 2.00 0.88 2.01 1.00 2.02 2.02 0.76
10.5 10.0 9.5 9.0 8.5 8.0 7.5 7.0 6.5 6.0 5.5 5.0 4.5 4.0 3.5 3.0 2.5 2.0 1.5 1.0 0.5 0.0 -0.5 -1.0

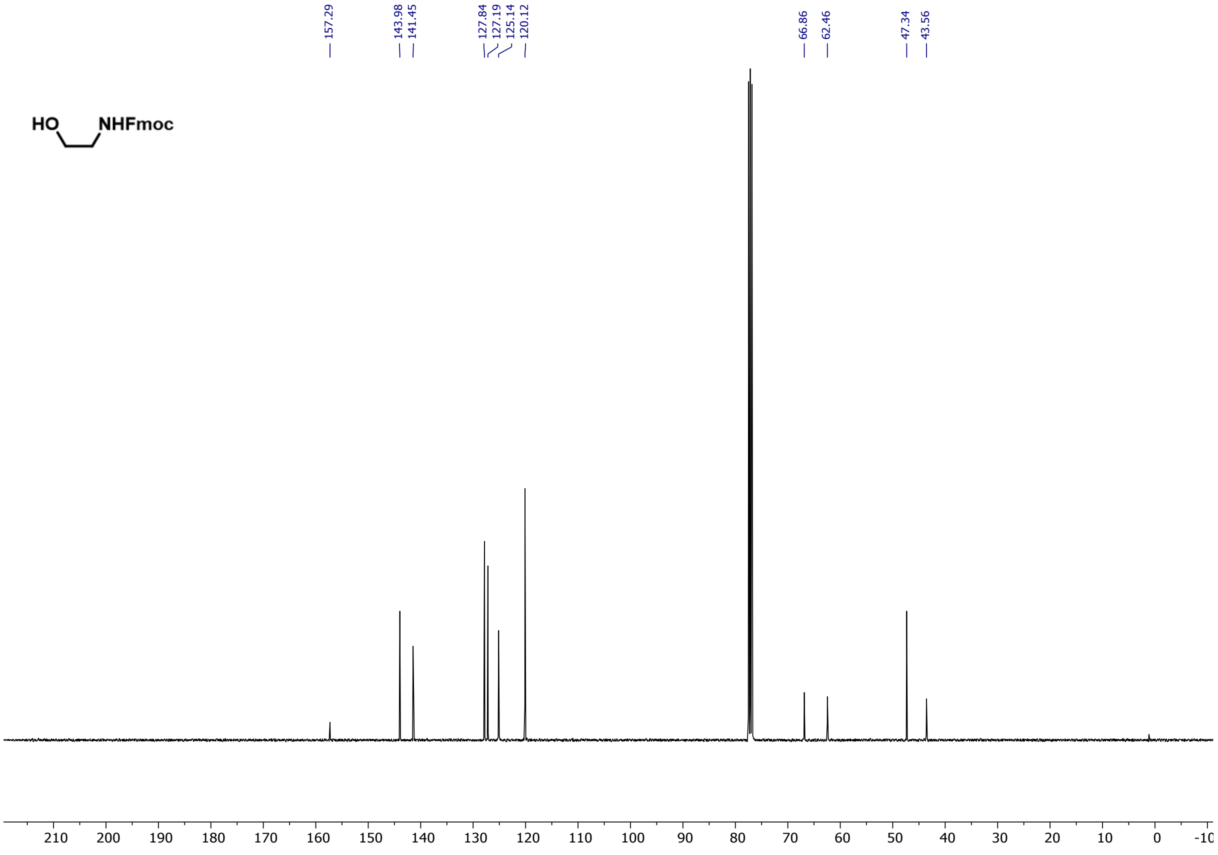
157.29
143.98 141.45
127.84 127.19 125.14 120.12
66.86 62.46
47.34 43.56
HO NHFmoc
210 200 190 180 170 160 150 140 130 120 110 100 90 80 70 60 50 40 30 20 10 0 -10

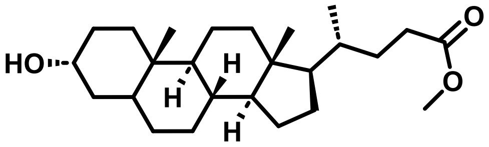

Lithocholic acid methyl ester (17)

Lithocholic acid (2.5 g, 6.64 mmol, 1 eq.) in anhydrous MeCN/DMF (1:1, 0.05 M) was stirred with activated MS 3 Å for 30 min. K_2CO_3 (1.10 g, 7.97 mmol, 1.2 eq.) was added and the mixture was stirred for another 5 min before iodomethane (420 µl, 6.70 mmol, 1 eq.) was added dropwise. The reaction was heated to 50 °C and stirred for 1 h. The resulting suspension was filtered, the solvent of the filtrate was removed under reduced pressure. The residue was dissolved in CH_2Cl_2, washed with $NaHCO_3$ and dried over Na_2SO_4. The solvent was removed under reduced pressure and **17** was isolated by FCC (3:7 EtOAc/hexanes; Rf = 0.29) in 84% yield as a white solid (2.19 g, 5.61 mmol).

^{1}H NMR (400 MHz, $CDCl_3$) *δ* 3.64 (s, 3H), 3.59 (dt, *J* = 11.0, 4.7 Hz, 1H), 2.34 (ddd, *J* = 15.3, 10.2, 5.1 Hz, 1H), 2.20 (ddd, *J* = 15.6, 9.7, 6.5 Hz, 1H), 1.93 (dt, *J* = 12.3, 3.0 Hz, 1H), 1.88 – 1.68 (m, 6H), 1.64 (dq, *J* = 12.4, 2.5 Hz, 1H), 1.59 – 1.45 (m, 2H), 1.42 – 1.00 (m, 16H), 0.96 (dd, *J* = 14.2, 3.4 Hz, 1H), 0.89 (d, *J* = 6.3 Hz, 6H), 0.62 (s, 3H).

^{13}C NMR (101 MHz, $CDCl_3$) *δ* 174.9, 71.9, 56.6, 56.0, 51.6, 42.8, 42.2, 40.5, 40.2, 36.5, 35.9, 35.5, 35.4, 34.7, 31.1, 31.1, 30.6, 28.3, 27.3, 26.5, 24.3, 23.5, 20.9, 18.4, 12.1.

HRMS (ESI): $C_{25}H_{42}NaO_3$ $[M+Na]^+$; calculated: 413.3032, found: 413.2661.

Optical rotation: $[\alpha]_D^{25}$ = +28.3° (c = 1.0, $CHCl_3$)

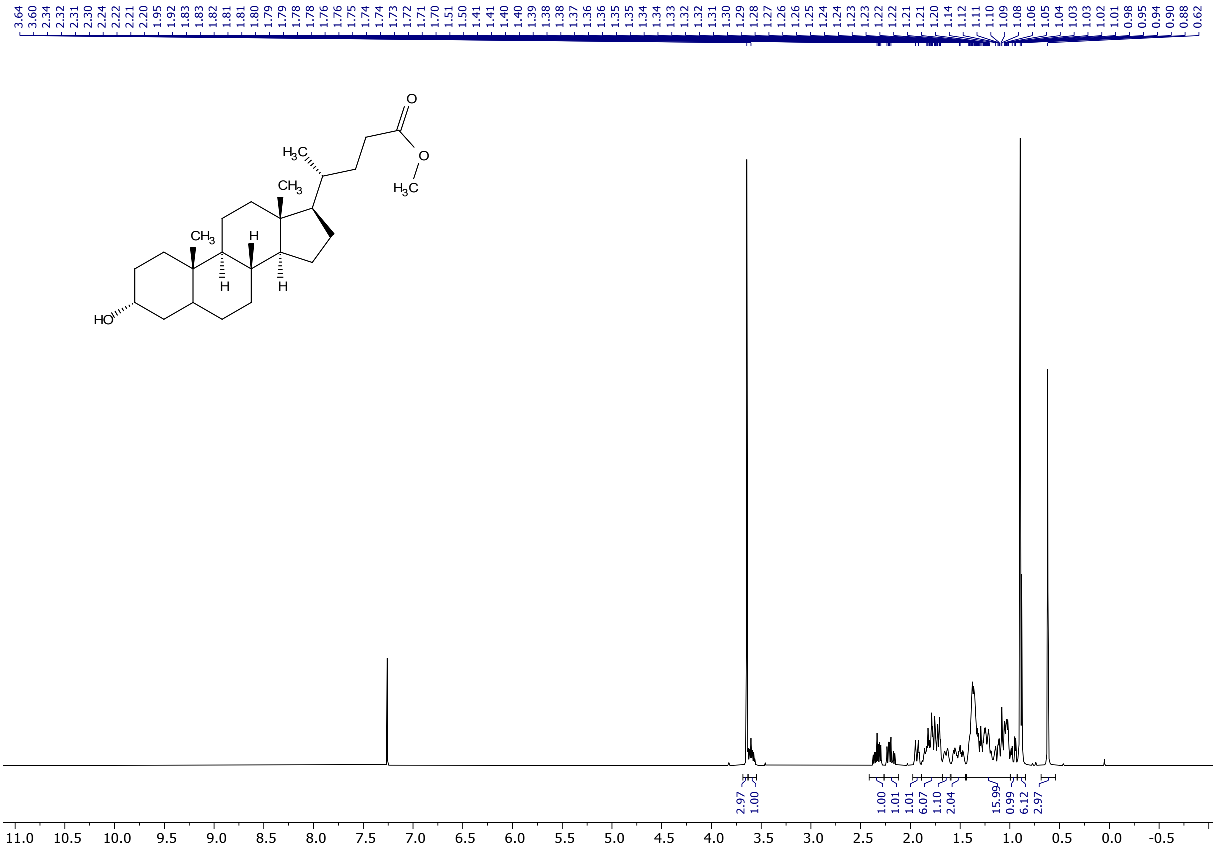
3.64 3.60 2.34 2.32 2.31 2.30 2.24 2.22 2.21 2.20 1.95 1.92 1.83 1.83 1.82 1.81 1.81 1.80 1.79 1.79 1.78 1.78 1.76 1.75 1.74 1.74 1.73 1.72 1.71 1.70 1.51 1.50 1.41 1.41 1.40 1.40 1.39 1.38 1.38 1.37 1.36 1.36 1.35 1.35 1.34 1.34 1.33 1.32 1.32 1.31 1.30 1.29 1.28 1.27 1.26 1.26 1.25 1.24 1.24 1.23 1.23 1.22 1.22 1.21 1.21 1.20 1.14 1.12 1.11 1.10 1.09 1.08 1.06 1.05 1.04 1.03 1.03 1.02 1.01 0.98 0.95 0.94 0.90 0.88 0.62
H3C
CH3
CH3
H
H
H
O
O
H3C
HO
2.97 1.00 1.00 1.01 1.01 6.07 1.10 2.04 15.99 0.99 6.12 2.97
11.0 10.5 10.0 9.5 9.0 8.5 8.0 7.5 7.0 6.5 6.0 5.5 5.0 4.5 4.0 3.5 3.0 2.5 2.0 1.5 1.0 0.5 0.0 -0.5

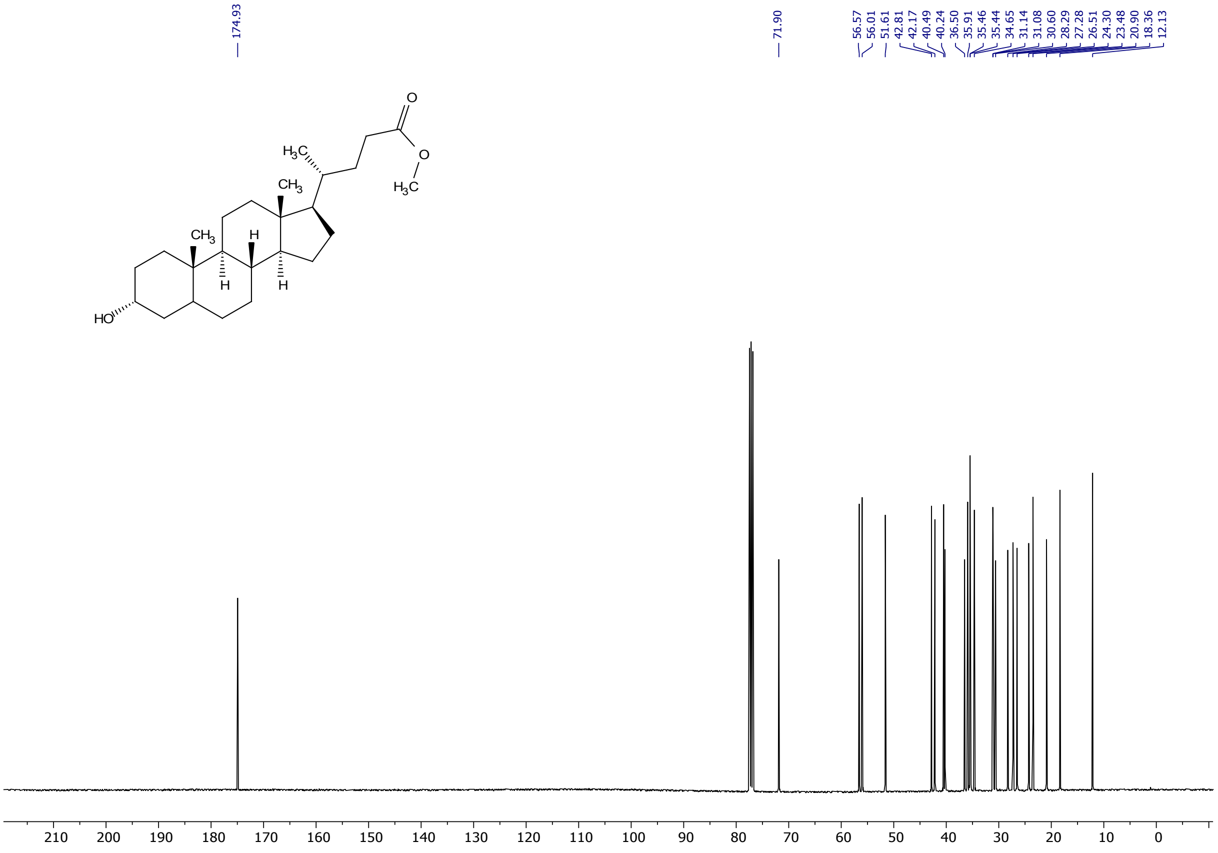
174.93
71.90
56.57 56.01 51.61 42.81 42.17 40.49 40.24 36.50 35.91 35.46 35.44 34.65 31.14 31.08 30.60 28.29 27.28 26.51 24.30 23.48 20.90 18.36 12.13
H3C
CH3
CH3
H
H
H
O
O
H3C
HO
210 200 190 180 170 160 150 140 130 120 110 100 90 80 70 60 50 40 30 20 10 0

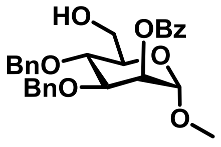

Methyl 2-*O*-benzoyl-3,4-di-*O*-benzyl-6-*O*-(9-fluorenylmethoxycarbonyl)-1-*O*-*α*-D-mannopyranoside (19)

NIS (608 mg, 3.02 mmol, 1.2 eq.) was added to a mixture of thioglycoside **7** (2.00 g, 2.52 mmol, 1 eq.), anhydrous methanol (410 µl, 10.1 mmol, 4 eq.) and activated MS (3 Å) in anhydrous CH_2Cl_2 (0.1 M) at 0 °C under nitrogen. The reaction was stirred for 15 min before TfOH (70 µL, 756 µmol, 0.3 eq.) was added dropwise and stirred for 1 h. The reaction was stopped by addition of sat. aq. $NaHCO_3$, the phases were separated and the aqueous phase was extracted three times with CH_2Cl_2 (100 mL). The combined organic phases were washed with sat. aq. $NaHSO_3$, dried over Na_2SO_4 and the solvent was removed under reduced pressure.

The crude material was dissolved in anhydrous CH_2Cl_2 (0.1 M), cooled to 0 °C and piperidine (1.25 mL, 12.6 mmol, 5 eq.) added dropwise to this mixture. After 1 h the solvent was removed under reduced pressure.

19 was isolated by FCC (2:3 EtOAc/hexanes; Rf = 0.2) in 93% yield as a colorless oil (1.12 g, 2.34 mmol).

^{1}H NMR (400 MHz, $CDCl_3$) δ 8.03 – 7.97 (m, 2H), 7.55 – 7.48 (m, 1H), 7.39 (t, *J* = 7.7 Hz, 2H), 7.30 – 7.19 (m, 7H), 7.19 – 7.14 (m, 3H), 5.52 (dd, *J* = 3.2, 1.9 Hz, 1H), 4.85 (d, *J* = 11.0 Hz, 1H), 4.74 (d, *J* = 1.8 Hz, 1H), 4.69 (d, *J* = 11.4 Hz, 1H), 4.58 (d, *J* = 10.9 Hz, 1H), 4.49 (d, *J* = 11.5 Hz, 1H), 4.02 (dd, *J* = 9.3, 3.2 Hz, 1H), 3.90 (t, *J* = 9.5 Hz, 1H), 3.84 – 3.70 (m, 2H), 3.65 (dt, *J* = 9.7, 3.4 Hz, 1H), 3.29 (s, 3H), 1.93 (dd, *J* = 8.0, 5.2 Hz, 1H).

^{13}C NMR (101 MHz, $CDCl_3$) δ 165.8, 138.3, 138.1, 133.4, 130.0, 129.9, 128.6, 128.5, 128.4, 128.2, 128.1, 127.9, 127.8, 99.0, 78.2, 75.4, 74.1, 71.8, 71.6, 69.1, 62.2, 55.2.

HRMS (ESI): $C_{28}H_{30}NaO_7$ $[M+Na]^+$; calculated: 501.1889, found: 501.1885.

Optical rotation: $[\alpha]_D^{25}$ = -12.3° (c = 1.0, $CHCl_3$)

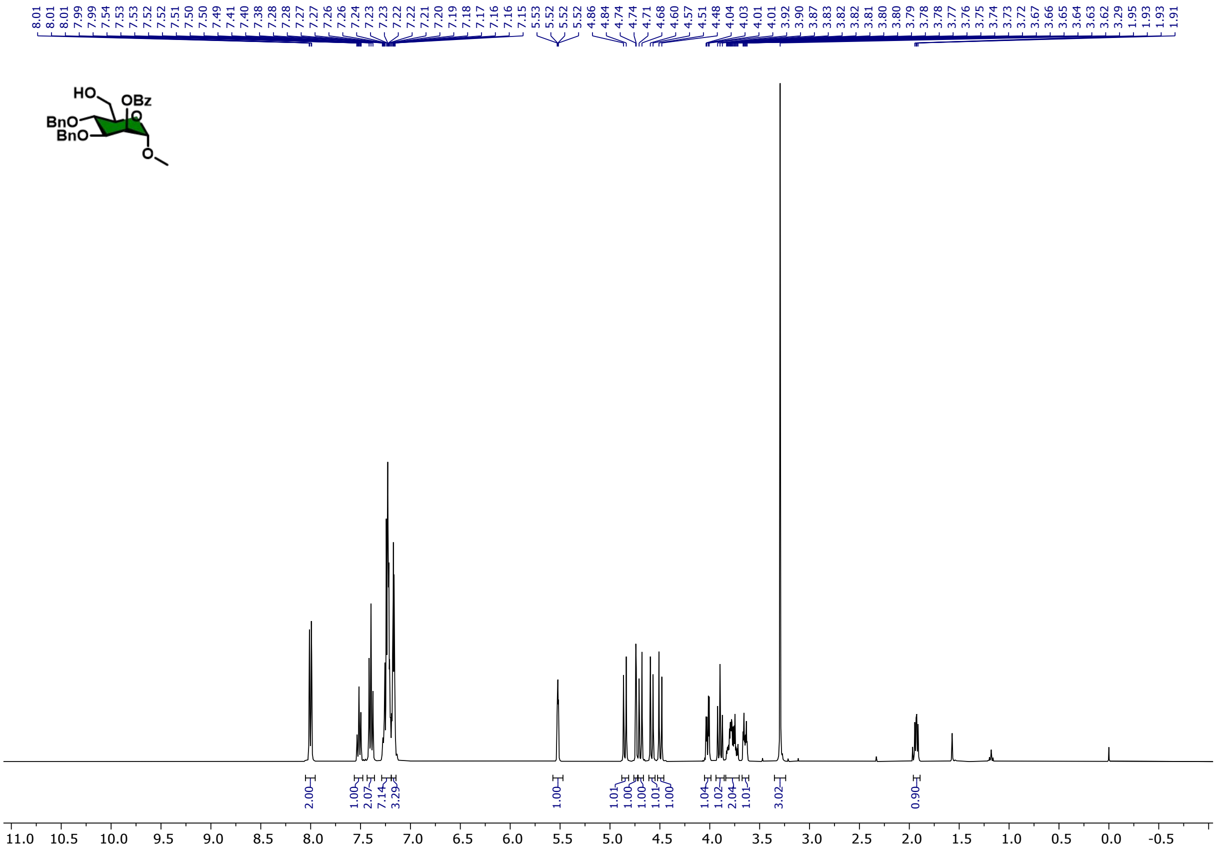
8.01 8.01 8.01 7.99 7.99 7.54 7.53 7.53 7.52 7.52 7.51 7.50 7.50 7.49 7.41 7.40 7.38 7.28 7.28 7.27 7.27 7.26 7.26 7.24 7.23 7.23 7.22 7.22 7.21 7.20 7.19 7.18 7.17 7.16 7.16 7.15 5.53 5.52 5.52 5.52 4.86 4.84 4.74 4.74 4.71 4.68 4.60 4.57 4.51 4.48 4.04 4.03 4.01 4.01 3.92 3.90 3.87 3.83 3.82 3.82 3.81 3.80 3.80 3.79 3.78 3.78 3.77 3.76 3.75 3.74 3.73 3.72 3.67 3.66 3.65 3.64 3.63 3.62 3.29 1.95 1.93 1.93 1.91
HO
OBz
BnO
BnO
O
2.00 1.00 2.07 7.14 3.29 1.00 1.01 1.00 1.00 1.00 1.01 1.00 1.04 1.02 2.04 1.01 3.02 0.90
11.0 10.5 10.0 9.5 9.0 8.5 8.0 7.5 7.0 6.5 6.0 5.5 5.0 4.5 4.0 3.5 3.0 2.5 2.0 1.5 1.0 0.5 0.0 -0.5

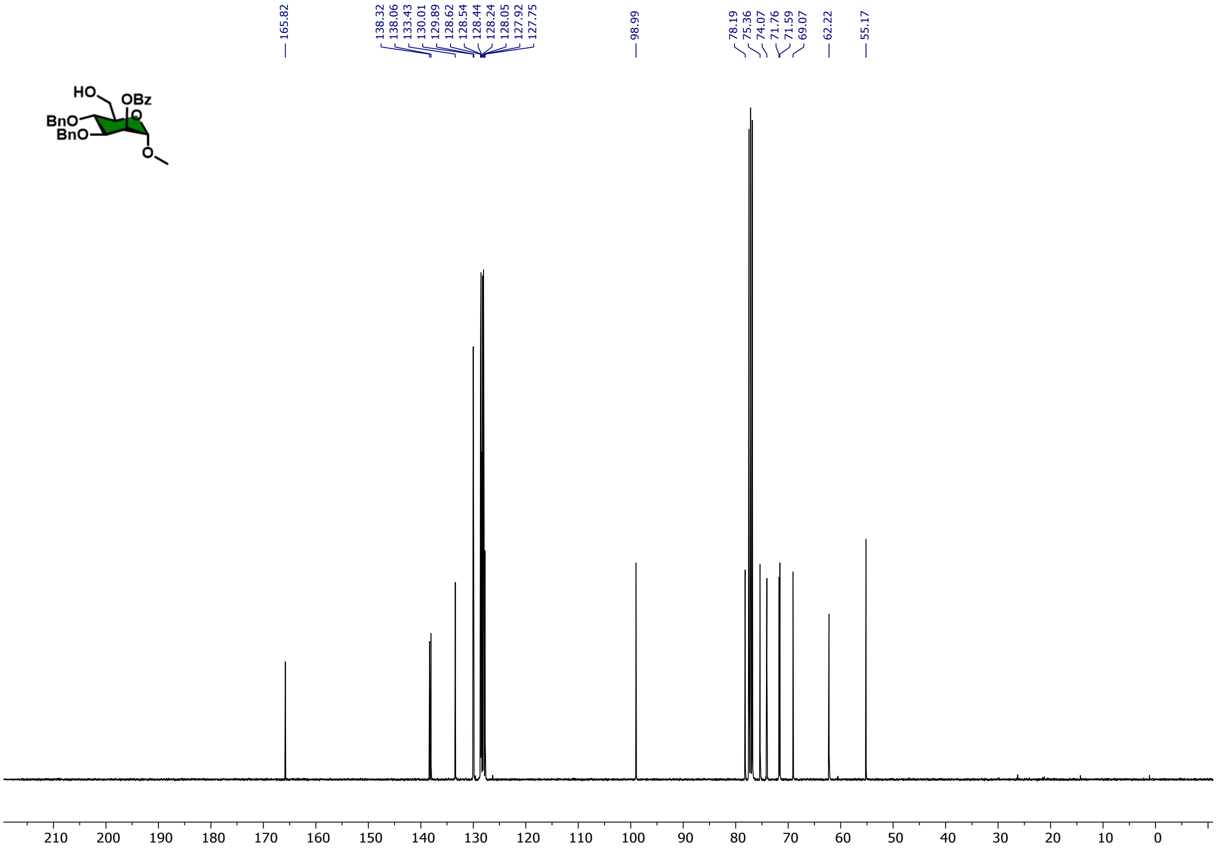
165.82
138.32 138.06 133.43 130.01 129.89 128.62 128.54 128.44 128.24 128.05 127.92 127.75
98.99
78.19 75.36 74.07 71.76 71.59 69.07
62.22
55.17
HO
OBz
BnO
BnO
O
210 200 190 180 170 160 150 140 130 120 110 100 90 80 70 60 50 40 30 20 10 0

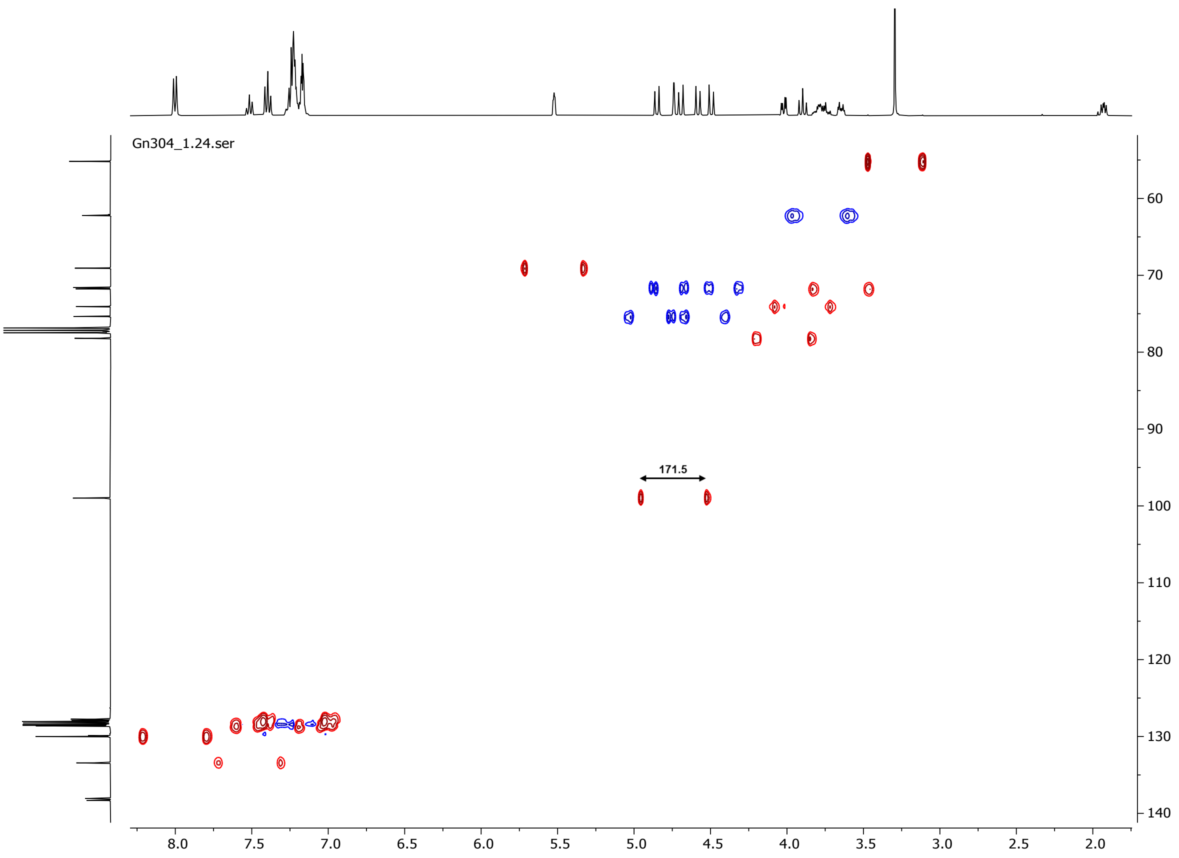
Gn304_1.24.ser
171.5
8.0 7.5 7.0 6.5 6.0 5.5 5.0 4.5 4.0 3.5 3.0 2.5 2.0
60 70 80 90 100 110 120 130 140

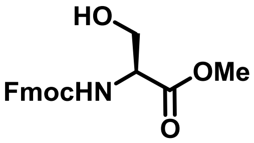

Methyl (((9H-fluoren-9-yl)methoxy)carbonyl)-L-serinate (21)

Pyridine (20 mL) was added to L-Serine methyl ester hydrochloride (2.5 g, 16.1 mmol, 1 eq.) in anhydrous CH_2Cl_2 (0.1 M) under nitrogen at - 18 °C. Then FmocCl (4.37 g, 16.9 mmol, 1.05 eq.) in CH_2Cl_2 was added dropwise. The reaction was allowed to warm to 23 °C and stirred for 3 h before the solvent was removed under reduced pressure. The resulting solid was dissolved in CH_2Cl_2 and washed with aq. citric acid (10% w/v). The aqueous phase was extracted three times with CH_2Cl_2 (50 mL). The combined organic phases were dried over Na_2SO_4 and the solvent was removed under reduced pressure. **21** was isolated by FCC (3:2 EtOAc/CH_2Cl_2; Rf = 0.2) in 78% yield as a colorless foam (4.3 g, 12.6 mmol).

^{1}H NMR (400 MHz, $CDCl_3$) δ 7.76 (d, J = 7.5 Hz, 2H), 7.60 (dd, J = 7.7, 4.1 Hz, 2H), 7.40 (t, J = 7.4 Hz, 2H), 7.34 – 7.27 (m, 2H), 5.83 (d, J = 7.8 Hz, 1H), 4.57 – 4.36 (m, 3H), 4.22 (t, J = 6.9 Hz, 1H), 4.06 – 3.86 (m, 2H), 3.78 (s, 3H), 2.49 (t, J = 6.1 Hz, 1H).

^{13}C NMR (101 MHz, $CDCl_3$) δ 171.2, 156.4, 143.9, 143.8, 141.4, 141.4, 127.9, 127.2, 127.2, 125.2, 120.1, 120.1, 67.3, 63.3, 56.1, 52.9, 47.2.

HRMS (ESI): $C_{19}H_{19}NNaO_5$ $[M+Na]^+$; calculated: 364.1161, found: 364.1155.

Optical rotation: $[\alpha]_D^{25}$ = +2.0° (c = 1.0, $CHCl_3$)

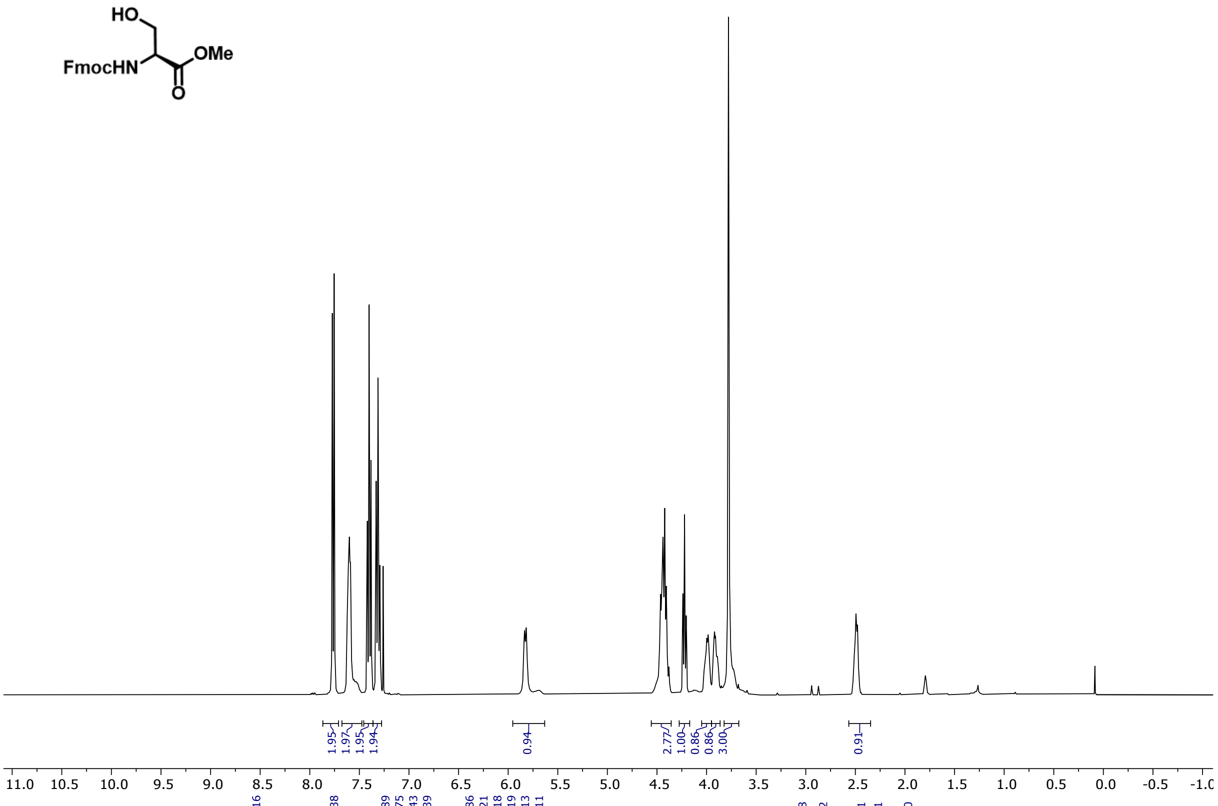
HO
OMe
FmocHN
O
1.95
1.97
1.95
1.94
0.94
2.77
1.00
0.86
0.86
3.00
0.91
11.0 10.5 10.0 9.5 9.0 8.5 8.0 7.5 7.0 6.5 6.0 5.5 5.0 4.5 4.0 3.5 3.0 2.5 2.0 1.5 1.0 0.5 0.0 -0.5 -1.0

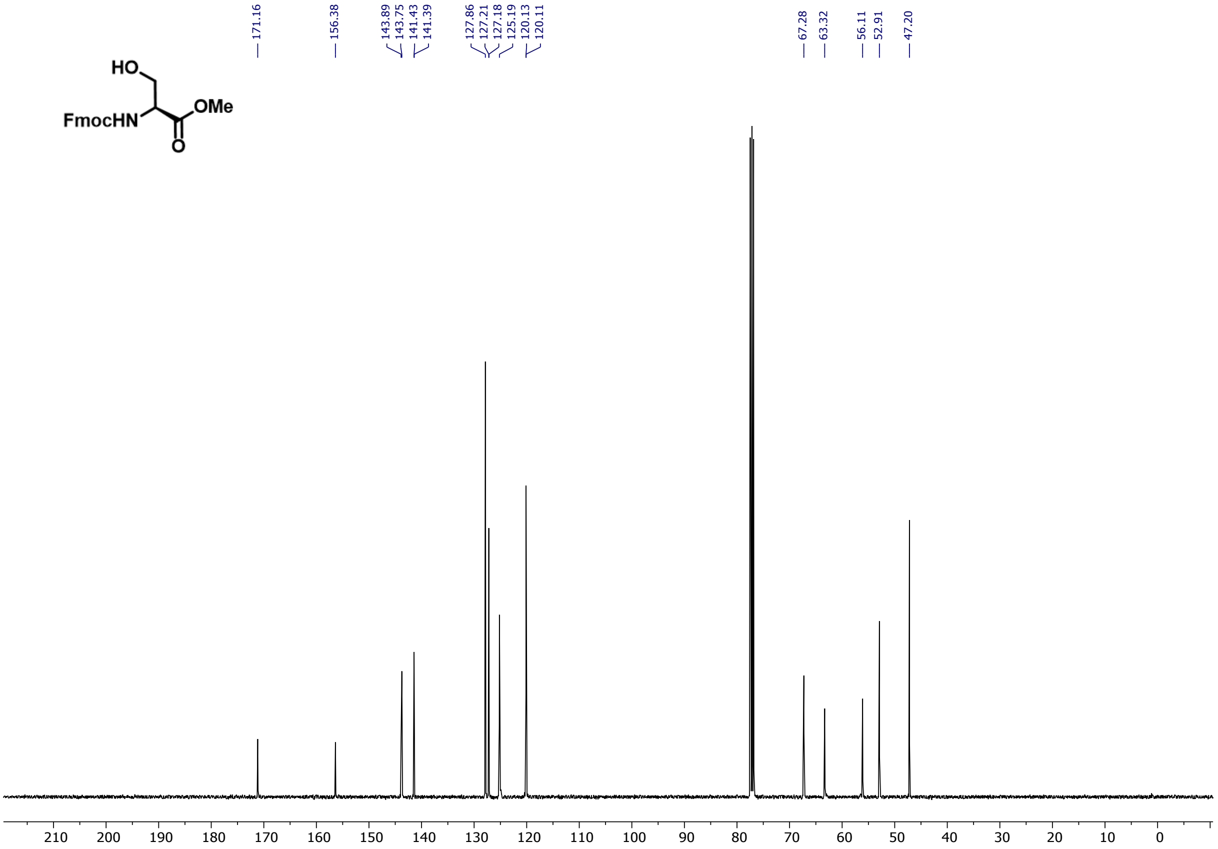
171.16
156.38
143.89
143.75
141.43
141.39
127.86
127.21
127.18
125.19
120.13
120.11
67.28
63.32
56.11
52.91
47.20
HO
OMe
FmocHN
O
210 200 190 180 170 160 150 140 130 120 110 100 90 80 70 60 50 40 30 20 10 0

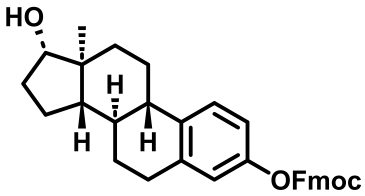

Estradiol-3-OFmoc (24)

FmocCl (476 mg, 1.84 mmol, 1 eq.) and DMAP (270 mL, 2.21 mmol, 1.2 eq) were added to estradiol (500 mg, 1.84 mmol, 1 eq.) in anhydrous THF/CH_2Cl_2 (1:1, 0.1 M) at -18 °C under nitrogen. The reaction was then allowed to warmed to 5 °C over 3 h. The reaction was diluted with CH_2Cl_2 and washed with aq. citric acid (10% w/v). The phases were separated and the aqueous phase was extracted with CH_2Cl_2 (three times 10 mL). The combined organic phases were dried over Na_2SO_4 and the solvent was removed under reduced pressure. **24** was isolated by FCC (1:2:2 EtOAc/CH_2Cl_2/hexanes; Rf = 0.36) in 95% yield as a colorless foam (866 mg, 1.75 mmol).

^{1}H NMR (400 MHz, $CDCl_3$) δ 7.79 (dt, J = 7.6, 1.0 Hz, 2H), 7.65 (ddt, J = 7.5, 1.8, 0.9 Hz, 2H), 7.43 (tt, J = 7.6, 0.9 Hz, 2H), 7.35 (td, J = 7.4, 1.2 Hz, 2H), 7.31 (dd, J = 8.6, 1.1 Hz, 1H), 6.95 (dd, J = 8.5, 2.7 Hz, 1H), 6.90 (d, J = 2.6 Hz, 1H), 4.52 (d, J = 7.4 Hz, 2H), 4.33 (t, J = 7.3 Hz, 1H), 3.74 (t, J = 8.5 Hz, 1H), 2.88 (dt, J = 8.5, 4.1 Hz, 2H), 2.39 – 2.29 (m, 1H), 2.23 (td, J = 11.1, 4.0 Hz, 1H), 2.13 (dtd, J = 13.0, 9.2, 5.4 Hz, 1H), 1.96 (ddd, J = 12.6, 3.9, 2.7 Hz, 1H), 1.93 – 1.84 (m, 1H), 1.71 (dddd, J = 12.4, 9.8, 6.9, 3.0 Hz, 1H), 1.62 – 1.25 (m, 7H), 1.25 – 1.13 (m, 1H), 0.79 (s, 3H).

^{13}C NMR (101 MHz, $CDCl_3$) δ 154.1, 149.0, 143.3, 141.5, 138.5, 138.5, 128.1, 127.3, 126.6, 125.3, 121.1, 120.3, 118.2, 82.0, 70.5, 50.2, 46.8, 44.2, 43.3, 38.6, 36.8, 30.7, 29.7, 27.1, 26.3, 23.2, 11.2.

HRMS (ESI): $C_{44}H_{42}NaO_8$ $[M+Na]^+$; calculated: 517.2359, found: 517.2355.

Optical rotation: $[\alpha]_D^{25}$ = +41.7° (c = 1.0, $CHCl_3$)

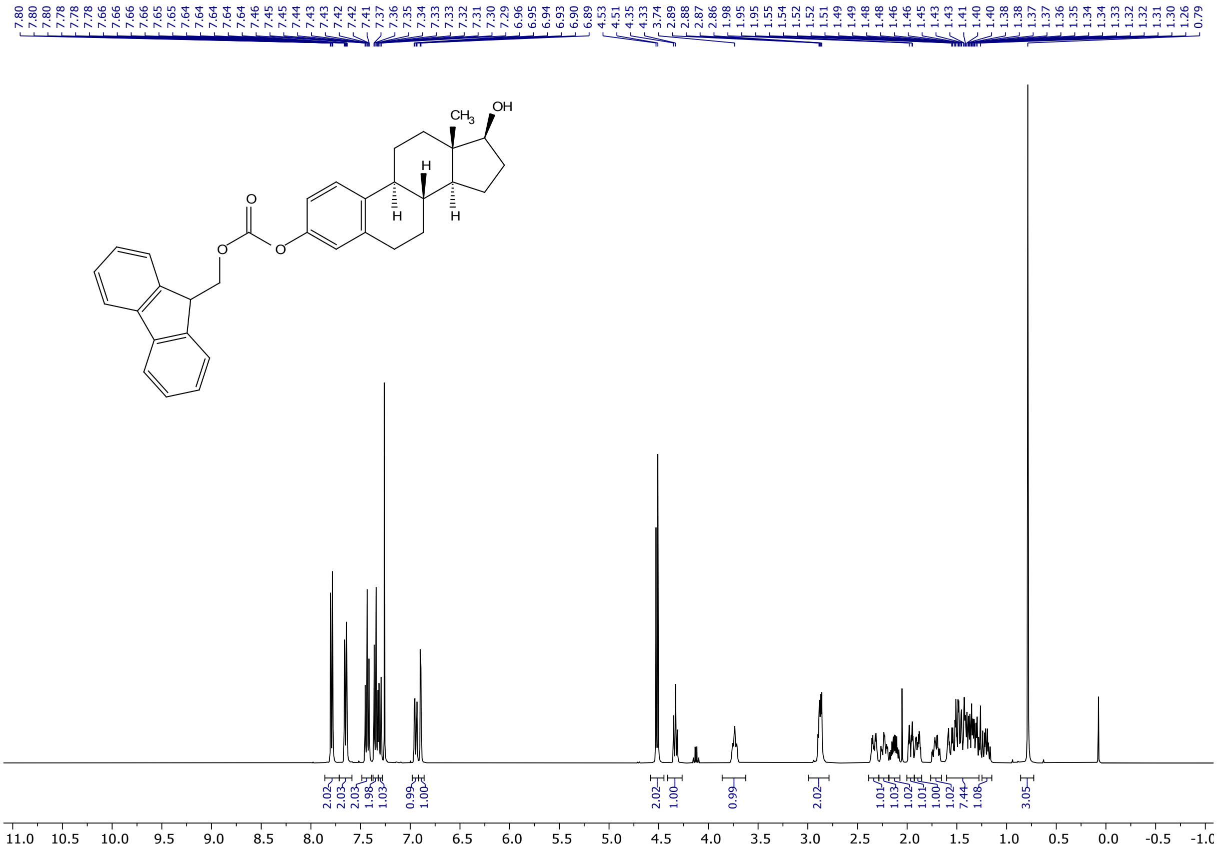
7.80 7.80 7.80 7.78 7.78 7.66 7.66 7.66 7.66 7.65 7.65 7.64 7.64 7.64 7.64 7.64 7.46 7.45 7.45 7.44 7.43 7.43 7.42 7.42 7.41 7.37 7.36 7.35 7.34 7.33 7.33 7.32 7.31 7.30 7.29 6.96 6.95 6.94 6.93 6.90 6.89 4.53 4.51 4.35 4.33 3.74 2.89 2.88 2.87 2.86 1.98 1.95 1.95 1.55 1.54 1.52 1.52 1.51 1.49 1.49 1.48 1.46 1.46 1.45 1.43 1.41 1.40 1.38 1.38 1.37 1.37 1.36 1.35 1.34 1.34 1.33 1.32 1.31 1.30 1.26 0.79
CH3
OH
H
H
H
O
O
O
2.02 2.03 2.03 1.98 1.03 0.99 1.00 2.02 1.00 0.99 2.02 1.01 1.03 1.02 1.01 1.00 1.02 7.44 1.08 3.05
11.0 10.5 10.0 9.5 9.0 8.5 8.0 7.5 7.0 6.5 6.0 5.5 5.0 4.5 4.0 3.5 3.0 2.5 2.0 1.5 1.0 0.5 0.0 -0.5 -1.0

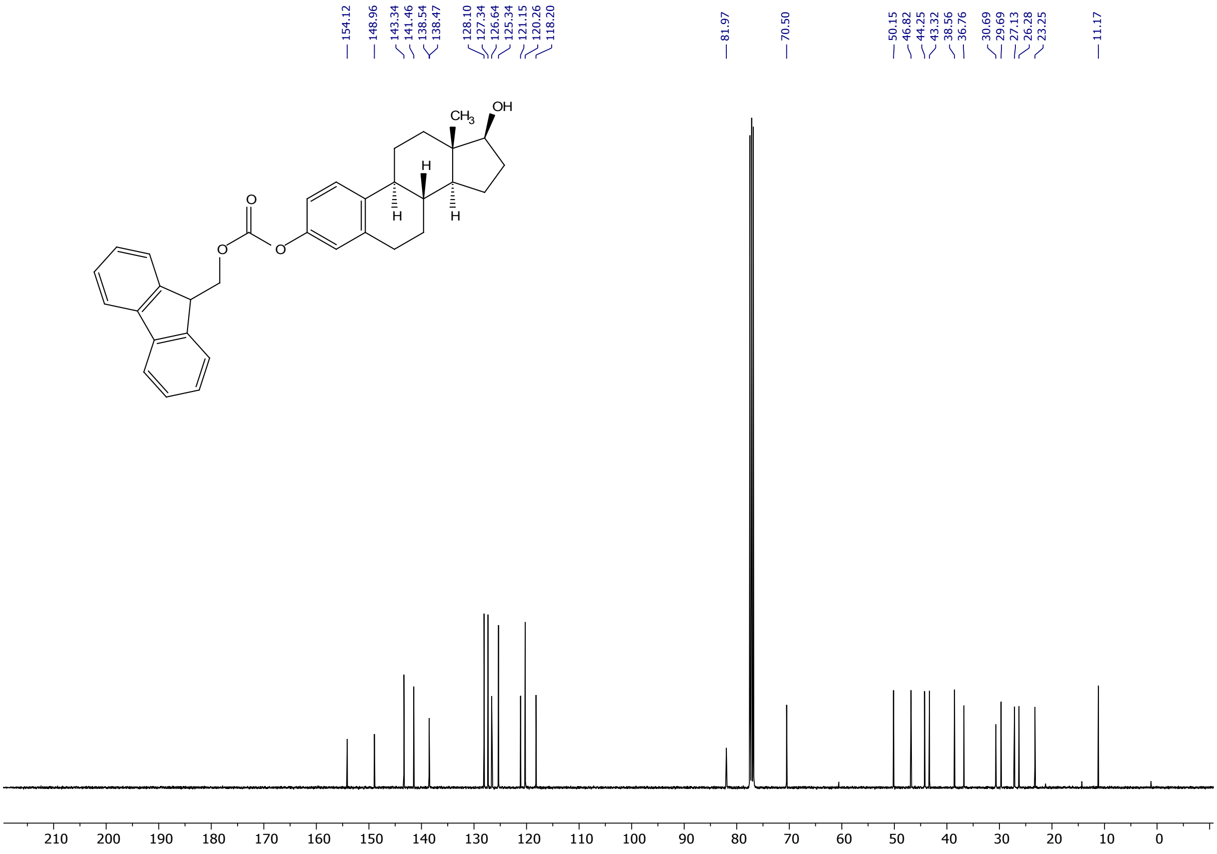
154.12 148.96 143.34 141.46 138.54 138.47 128.10 127.34 126.64 125.34 121.15 120.26 118.20 81.97 70.50 50.15 46.82 44.25 43.32 38.56 36.76 30.69 29.69 27.13 26.28 23.25 11.17
CH3
OH
H
H
H
O
O
O
210 200 190 180 170 160 150 140 130 120 110 100 90 80 70 60 50 40 30 20 10 0

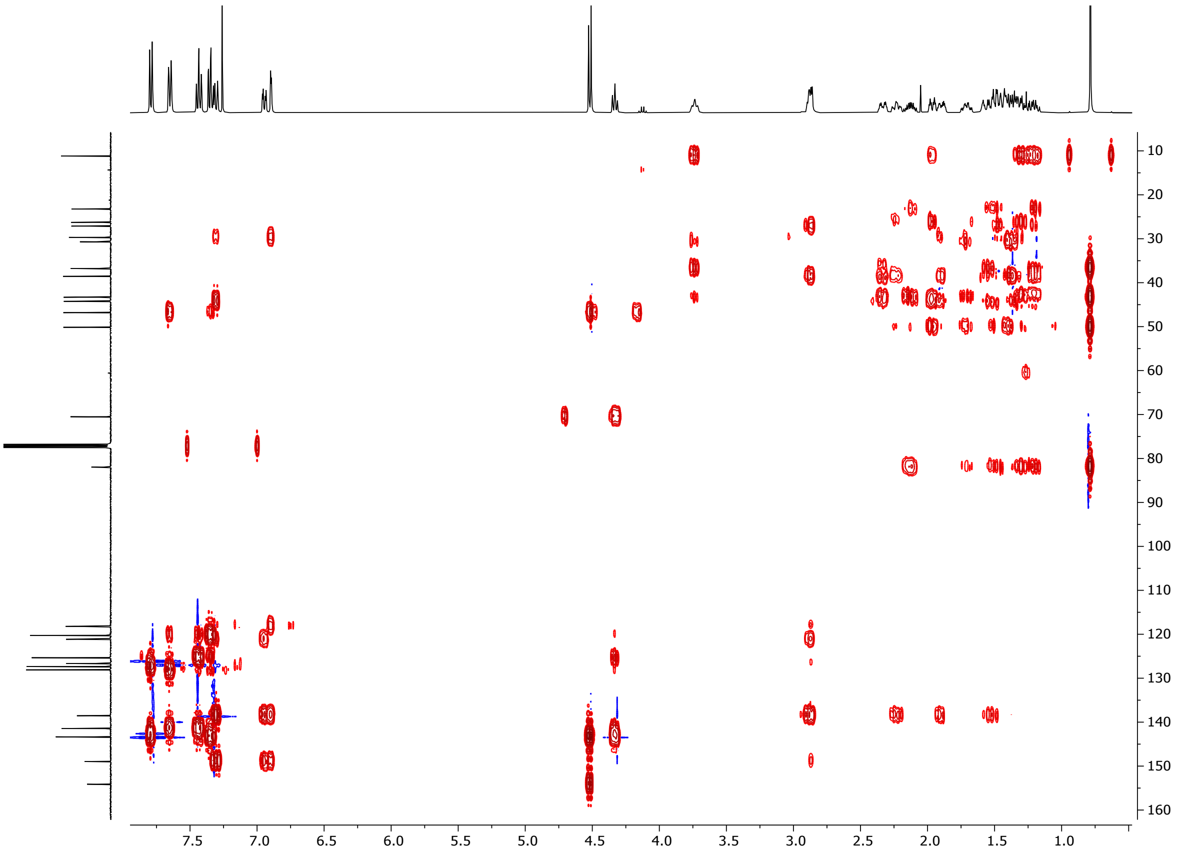

7.5
7.0
6.5
6.0
5.5
5.0
4.5
4.0
3.5
3.0
2.5
2.0
1.5
1.0
10
20
30
40
50
60
70
80
90
100
110
120
130
140
150
160

2.3 Photolabile Resins

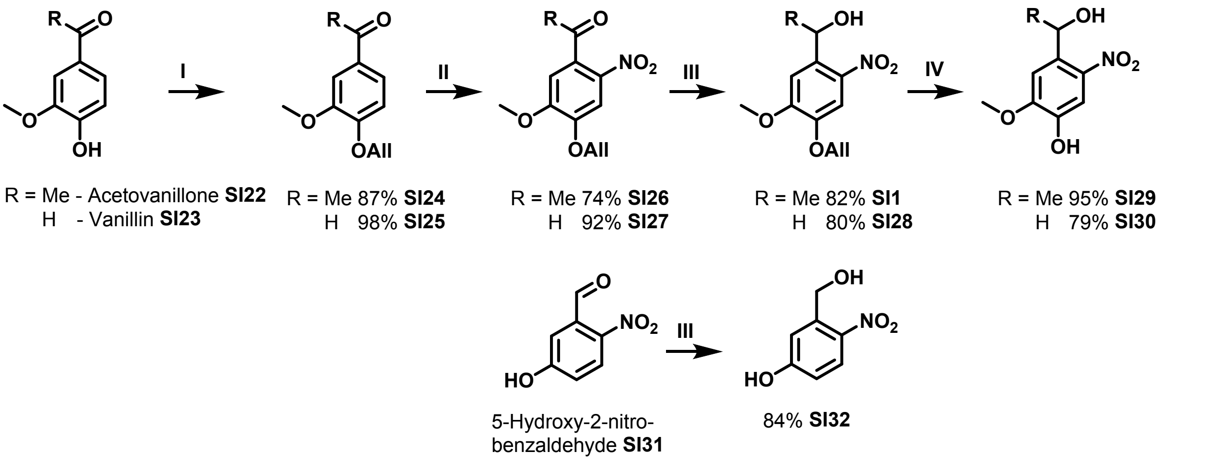

Scheme 2: Synthesis of ortho nitro benzyl linkers **SI29**, **SI30** and **SI32**. I) K_2CO_3, allyl bromide, MeCN, 50 °C, 16 h; II) KNO_3, TFA, - 10 °C to 60 °C, 4h; III) $NaBH_4$, EtOH/CH_2Cl_2 (3:1), 0 to 25 °C, 4 h; IV) $Pd(PPh_3)_4$, K_2CO_3, MeOH, 16 h.

The attachment of linkers **SI29**, **SI30** and **SI32** to Merrifield resin was performed according to literature[4]:

Merrifield resin (100-200 mesh, initial loading 0.67 or 1.02 mmol/g, Novabiochem® or Rapp Polymere GmbH, CAS: 70024-51-0) was washed two times with anhydrous CH_2Cl_2 and three times with in anhydrous DMF. The resin was then transferred to a brown glass flask and anhydrous DMF was added under an atmosphere of argon (twenty times the mmol of resin in mL). The respective nitrophenol linker (2 equiv.), Cs_2CO_3 (5 equiv.) were added. TBAI (1 equiv.) in a minimal amount of DMF was added dropwise while shaking the flask. The suspension was then shaken on a rotavap at 60 °C for 24 hours at 600 mbar and 60 rpm. The mixture was filtered and the resin was washed successively with THF, H_2O, THF, DMF, MeOH, CH_2Cl_2. The resin was dried under vacuum before being resuspended in anhydrous DMF (20x mmol of resin in mL). CsOAc (5 equiv.) was added and the suspension was shaken on a rotavap at 60 °C for 24 hours at 600 mbar and 60 rpm. The mixture was filtered and the resin was washed successively with THF, H_2O, THF, DMF, MeOH, CH_2Cl_2. The resin was dried under vacuum overnight and stored at -20°C under the exclusion of light.

Table 8: Merrifield resin functionalization with photolabile linkers.

No	**Chlorine loading** [mmol/g]	**Linker** [eq.]	**Linker loading** [mmol/g]
1	1.02	**SI32** (0.5)	0.36
2	1.02	**SI29** (0.5)	0.20
3	0.67	**SI30** (2)	0.43

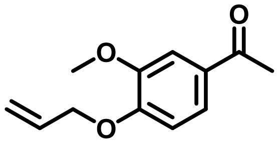

1-(4-(Allyloxy)-3-methoxyphenyl)ethan-1-one (SI24)

K_2CO_3 (4.61 g, 33.1 mmol, 1.1 eq.) was added to a solution of acetovanillone (5 g, 30.1 mmol) under nitrogen in anhydrous MeCN (0.1 M). Then allyl bromide (4 mL, 33.1 mmol, 1.1 eq.) was added dropwise and the reaction was heated for 16 h at 50 °C. The suspension was filtered and the solvent was removed under reduced pressure. **SI24** was isolated by FCC (3:2 hexane:EA; Rf = 0.3) in 87% yield as yellow oil (5.4 g, 26.2 mmol).

^{1}H NMR (400 MHz, $CDCl_3$) δ 7.56 – 7.47 (m, 2H), 6.89 – 6.83 (m, 1H), 6.13 – 5.99 (m, 1H), 5.41 (dq, J = 17.3, 1.5 Hz, 1H), 5.30 (dq, J = 10.5, 1.4 Hz, 1H), 4.66 (dt, J = 5.4, 1.6 Hz, 2H), 3.91 (s, 3H), 2.54 (s, 3H).

^{13}C NMR (101 MHz, $CDCl_3$) δ 196.9, 152.3, 149.3, 132.5, 130.6, 123.2, 118.7, 111.6, 110.4, 69.8, 6.1, 26.3.

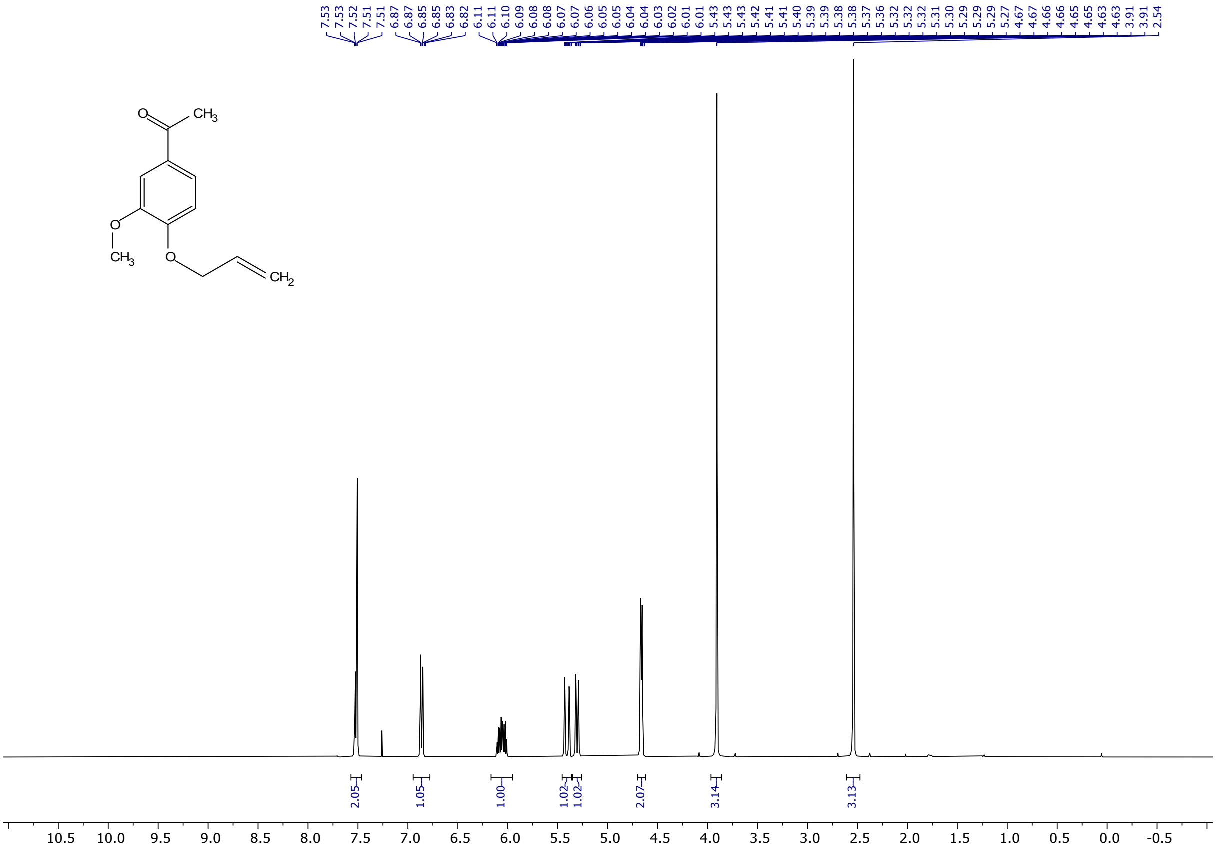

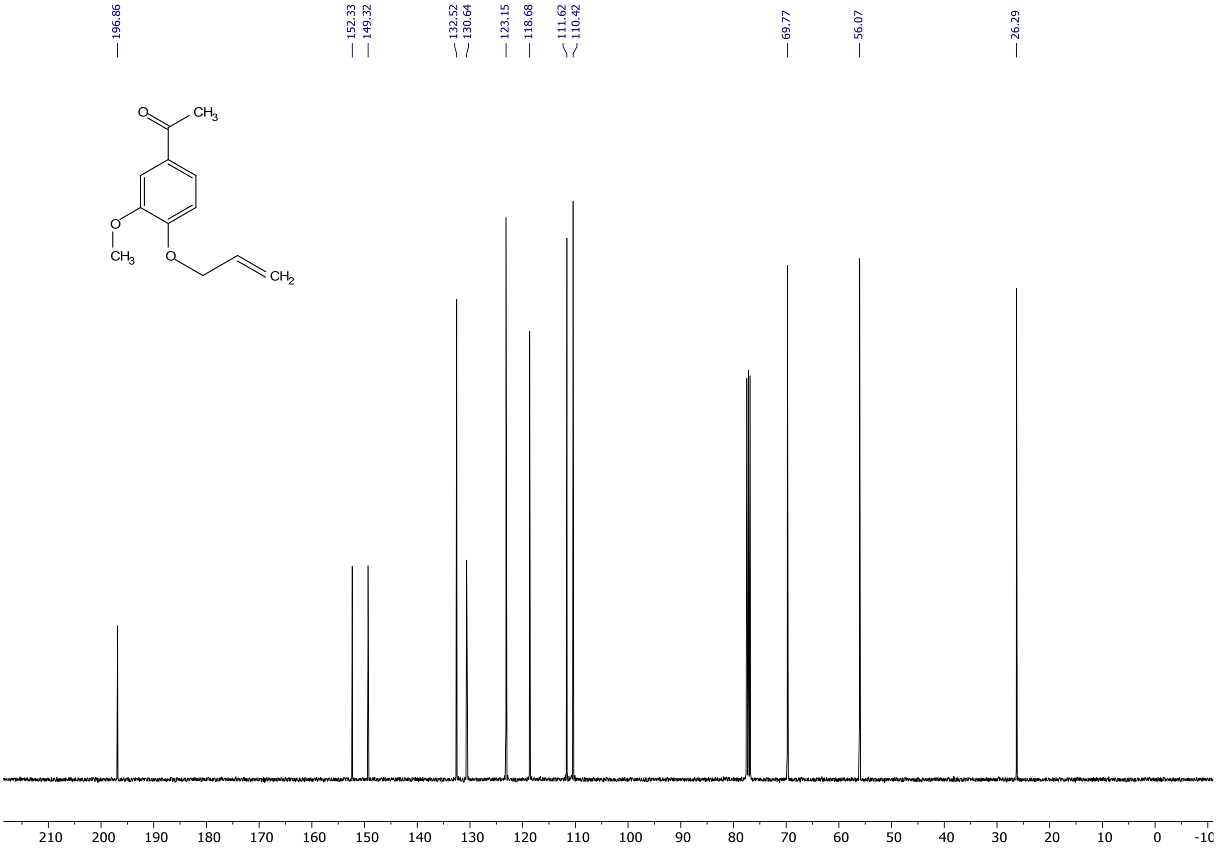

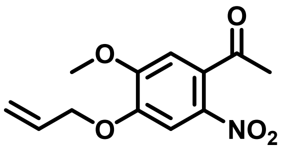

1-(4-(Allyloxy)-5-methoxy-2-nitrophenyl)ethan-1-one (SI26)

KNO_3 (54.9 g, 1.1 eq.) was added portion-wise to ketone **SI24** (10.3 g, 49.9 mmol) under nitrogen in TFA (1 M) at - 10 °C. The resultant dark solution was warmed to 60 °C and stirred for 4 h. CH_2Cl_2 (200 mL) was added at RT followed by water (200 mL). The phases were separated and the aqueous phase was extracted three times with CH_2Cl_2. The combined organic phases were washed with dilute $NaHCO_3$ solution, dried over Na_2SO_4 and the solvent was removed under reduced pressure. **SI26** was isolated by FCC (2:1 hexanes/EA; Rf = 0.39) in 74% yield as a yellow solid (9.24 g, 36.8 mmol).

^{1}H NMR (400 MHz, $CDCl_3$) δ 7.61 (s, 1H), 6.76 (s, 1H), 6.06 (ddt, J = 17.2, 10.7, 5.4 Hz, 1H), 5.46 (dq, J = 17.2, 1.5 Hz, 1H), 5.37 (dq, J = 10.5, 1.3 Hz, 1H), 4.70 (dt, J = 5.4, 1.5 Hz, 2H), 3.97 (s, 3H), 2.49 (s, 3H).

^{13}C NMR (101 MHz, $CDCl_3$) δ 200.3, 154.4, 148.5, 138.3, 133.1, 131.6, 119.6, 108.8, 108.4, 70.4, 56.8, 30.6.

HRMS (ESI): $C_{12}H_{14}NO_5$ $[M+H]^+$; calculated: 252.0872, found: 252.0849.

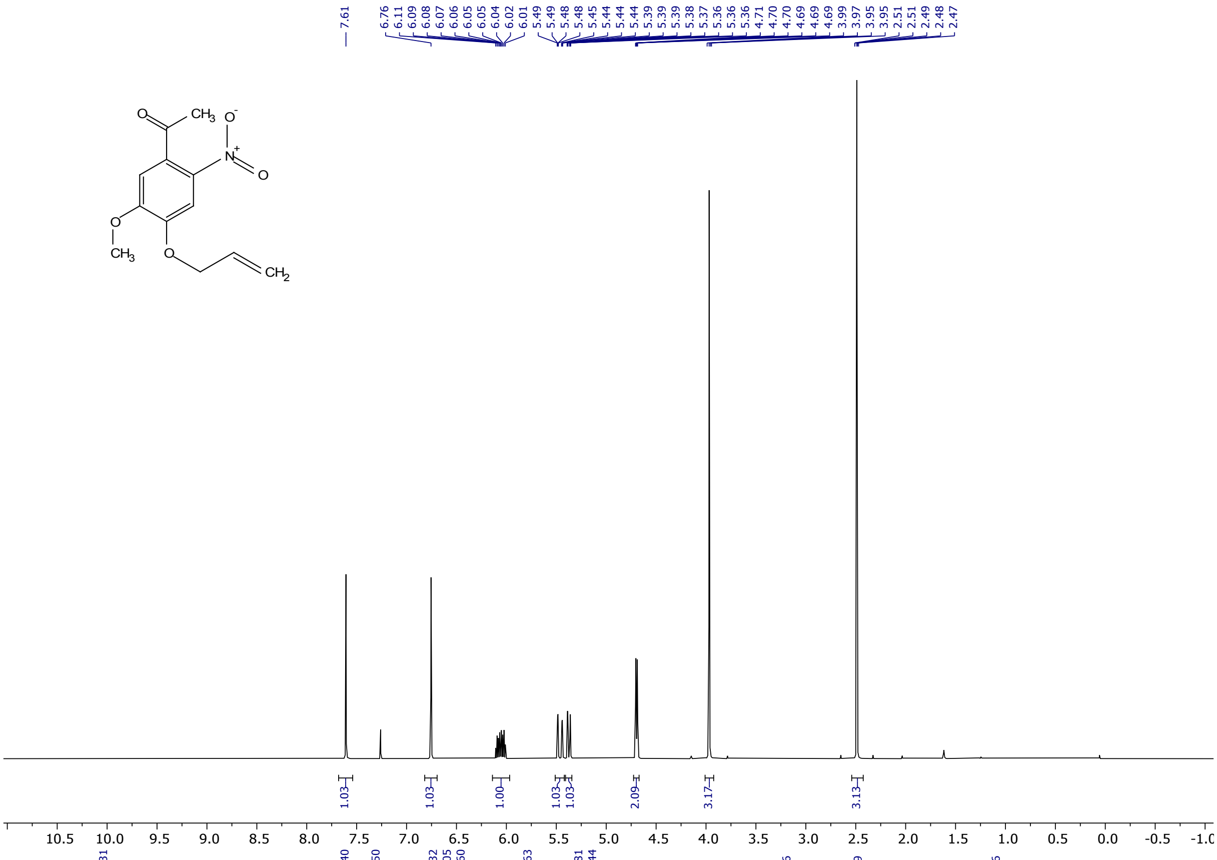
7.61
6.76 6.11 6.09 6.08 6.07 6.06 6.05 6.05 6.04 6.02 6.01 5.49 5.49 5.48 5.48 5.45 5.44 5.44 5.44 5.39 5.39 5.39 5.38 5.37 5.36 5.36 5.36 4.71 4.70 4.70 4.69 4.69 4.69 3.99 3.97 3.95 3.95 2.51 2.51 2.49 2.48 2.47
O CH3 O- N+ O O CH3 O CH2
1.03 1.03 1.00 1.03 1.03 2.09 3.17 3.13
10.5 10.0 9.5 9.0 8.5 8.0 7.5 7.0 6.5 6.0 5.5 5.0 4.5 4.0 3.5 3.0 2.5 2.0 1.5 1.0 0.5 0.0 -0.5 -1.0

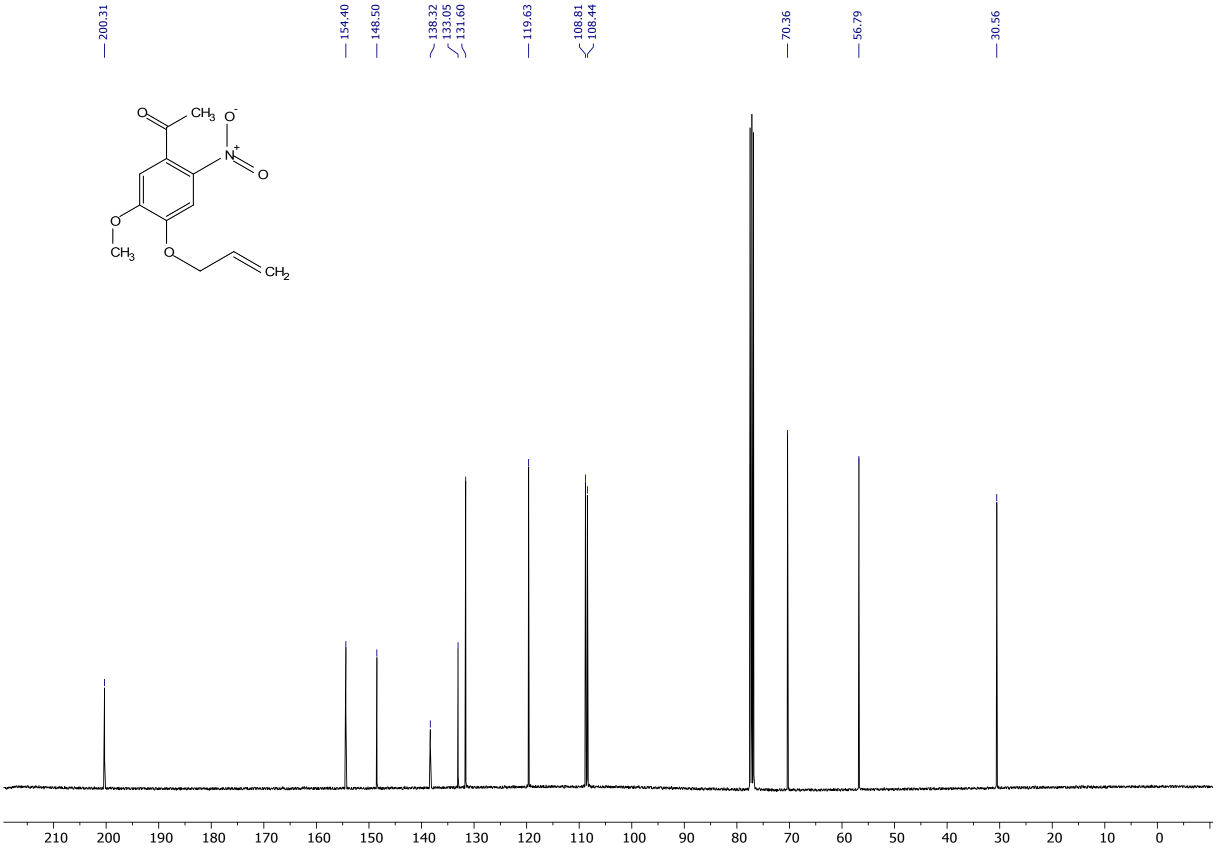
200.31
154.40
148.50
138.32 133.05 131.60
119.63
108.81 108.44
70.36
56.79
30.56
O CH3 O- N+ O O CH3 O CH2
210 200 190 180 170 160 150 140 130 120 110 100 90 80 70 60 50 40 30 20 10 0

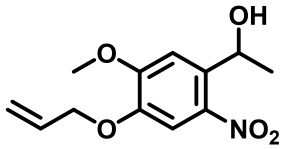

1-(4-(Allyloxy)-5-methoxy-2-nitrophenyl)ethan-1-ol (SI1)

$NaBH_4$ (2.74 g, 72.4 mmol, 2 eq.) was added in portions ketone **SI26** (9.1 g, 36.2 mmol) under nitrogen in EtOH/CH_2Cl_2 (3:1, 0.2 M) at 0 °C. The reaction was slowly warmed to 25 °C and kept at that temperature for another 4 h. The reaction cooled to 0 °C and stopped by the addition of acetone. The solvent was removed under reduced pressure, the residue was triturated in CH_2Cl_2 (100 mL) and washed with citric acid solution (100 mL, 10% w/w) The aqueous phase was extracted with CH_2Cl_2. The combined organic phases were dried over Na_2SO_4 and the solvent was removed under reduced pressure. **SI1** was isolated by FCC (2:1 hexane/EtOAc; Rf = 0.23) in 82% yield as a yellow solid (7.53 g, 29.7 µmol).

^{1}H NMR (400 MHz, $CDCl_3$) *δ* 7.57 (s, 1H), 7.30 (s, 1H), 6.07 (ddt, *J* = 17.3, 10.7, 5.5 Hz, 1H), 5.56 (qd, *J* = 6.4, 2.6 Hz, 1H), 5.45 (dq, *J* = 17.2, 1.5 Hz, 1H), 5.35 (dq, *J* = 10.5, 1.3 Hz, 1H), 4.66 (dt, *J* = 5.5, 1.5 Hz, 2H), 3.99 (s, 3H), 2.37 – 2.26 (m, 1H), 1.55 (d, *J* = 6.3 Hz, 3H)..

^{13}C NMR (101 MHz, $CDCl_3$) *δ* 154.2, 146.7, 139.6, 137.1, 132.1, 119.3, 109.5, 108.8, 70. 3, 65.9, 56.5, 24.4.

HRMS (ESI): $C_{12}H_{16}NO_5$ $[M+H]^+$; calculated: 254.1029, found: 254.2464.

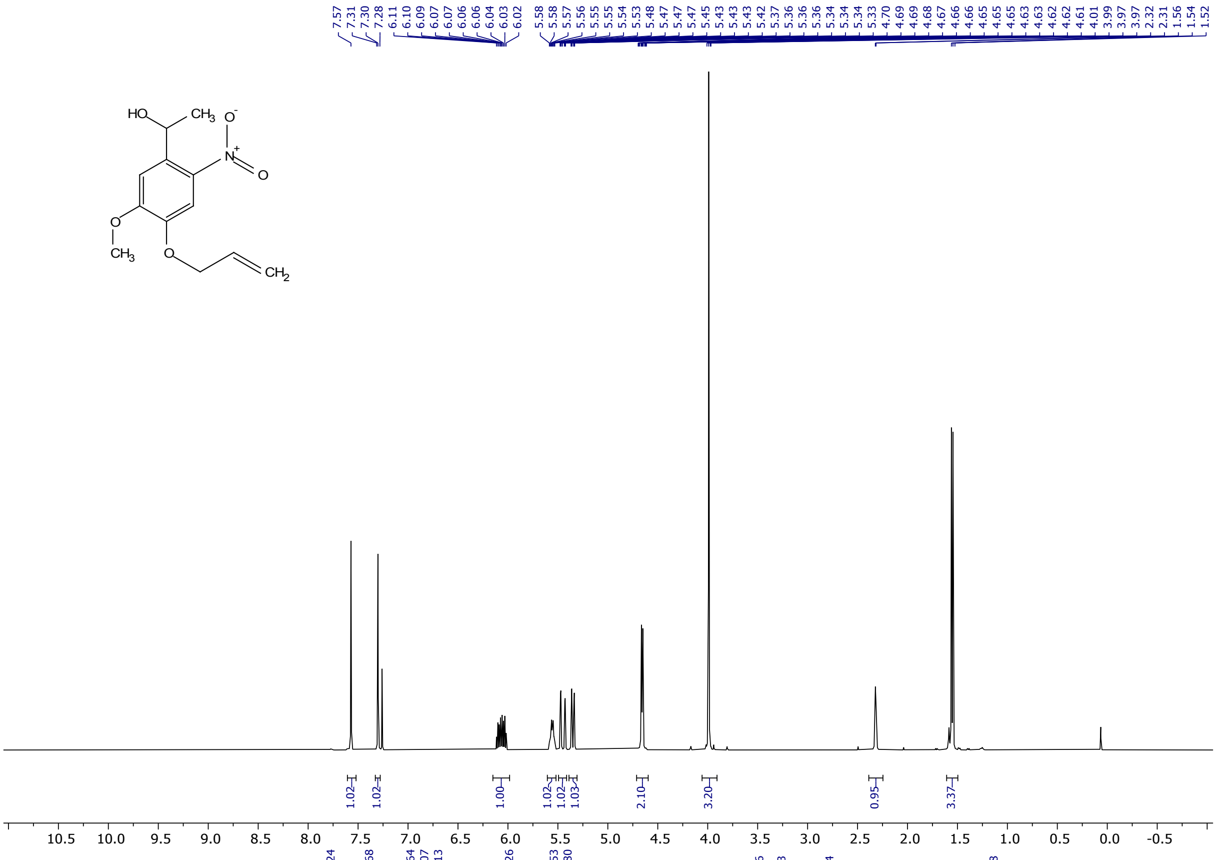
HO
CH3
O-
N+
O
O
CH3
O
CH2
1.02
1.02
1.00
1.02
1.02
1.03
2.10
3.20
0.95
3.37

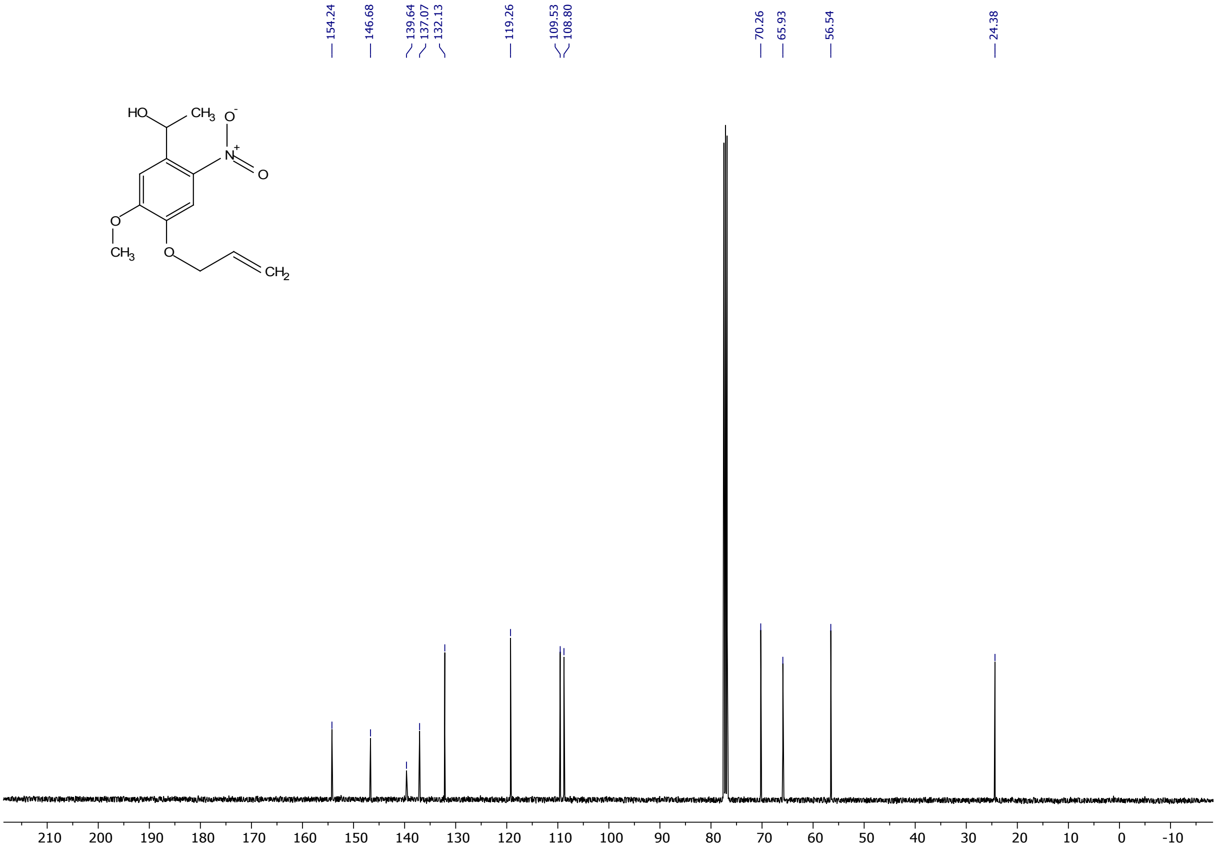
154.24
146.68
139.64
137.07
132.13
119.26
109.53
108.80
70.26
65.93
56.54
24.38
HO
CH3
O-
N+
O
O
CH3
O
CH2

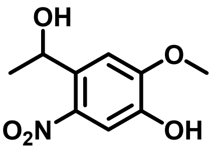

4-(1-Hydroxyethyl)-2-methoxy-5-nitrophenol (SI29)

$Pd(PPh_3)_4$ (365 mg, 0.316 mmol, 0.02 eq.) and K_2CO_3 (6.55 g, 47.4 mmol, 3 eq) were added to alcohol **SI1** (4 g, 15.8 mol) in MeOH (0.1 M) under nitrogen. The reaction was stirred for 16 h, diluted with CH_2Cl_2 and treated with citric acid (10% w/v aq., 300 mL). The aqueous phase was extracted with CH_2Cl_2 three times and the combined organic phases were dried over Na_2SO_4. The solvent was removed under reduced pressure and **SI39** was isolated by FCC (CH_2Cl_2/EtOAc 2:1; Rf = 0.28) in 95% yield as a beige solid (3.28 gg, 15.0 mmol).

^{1}H NMR (400 MHz, DMSO-d6) *δ* 9.92 (br. s, 1H), 7.41 (s, 1H), 7.32 (s, 1H), 5.41 (br. s, 1H), 5.24 (q, *J* = 6.4 Hz, 1H), 3.89 (s, 3H), 1.35 (d, *J* = 6.3 Hz, 3H).

^{13}C NMR (101 MHz, DMSO-d6) *δ* 152.6, 145.1, 138.9, 136.5, 110.7, 109.3, 63.9, 56.0, 25.3.

HRMS (ESI): $C_9H_{11}NNaO_5$ $[M+Na]^+$; calculated: 236.0535, found: 236.0897.

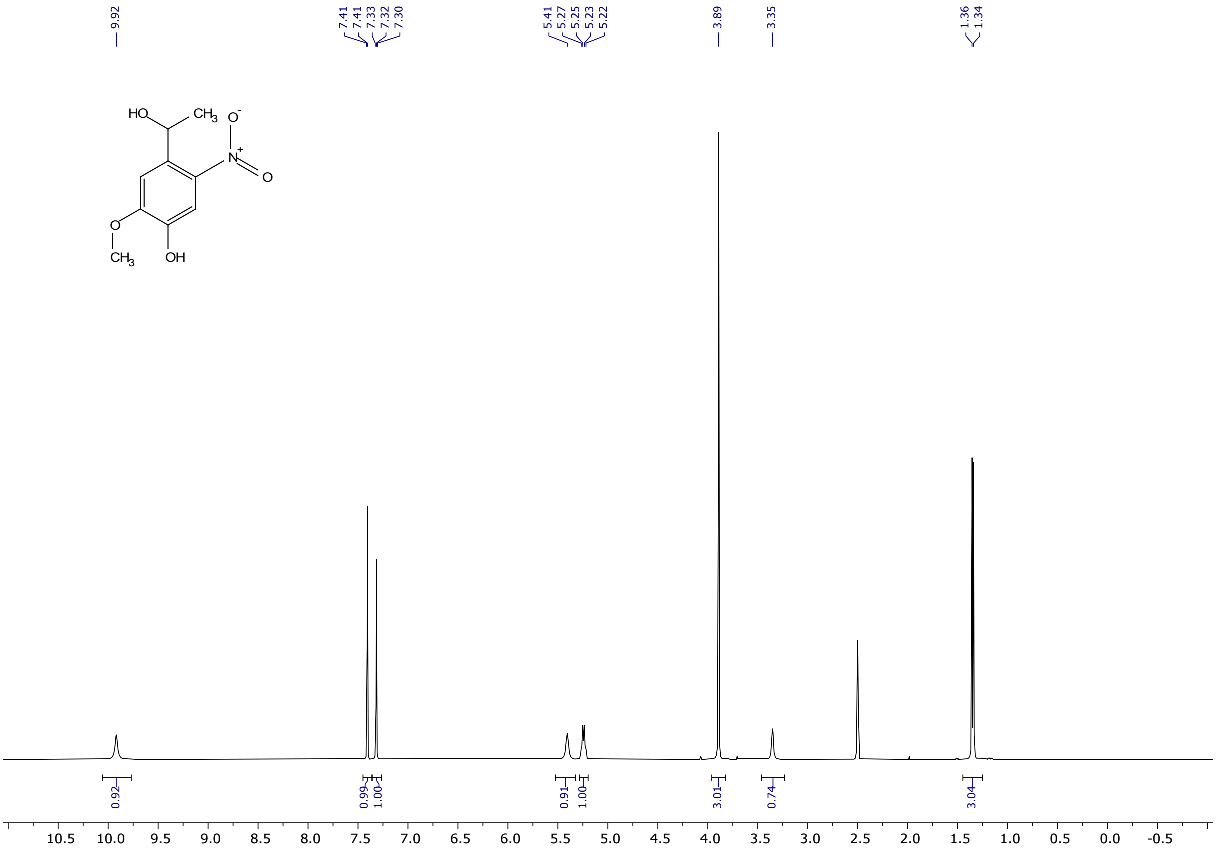

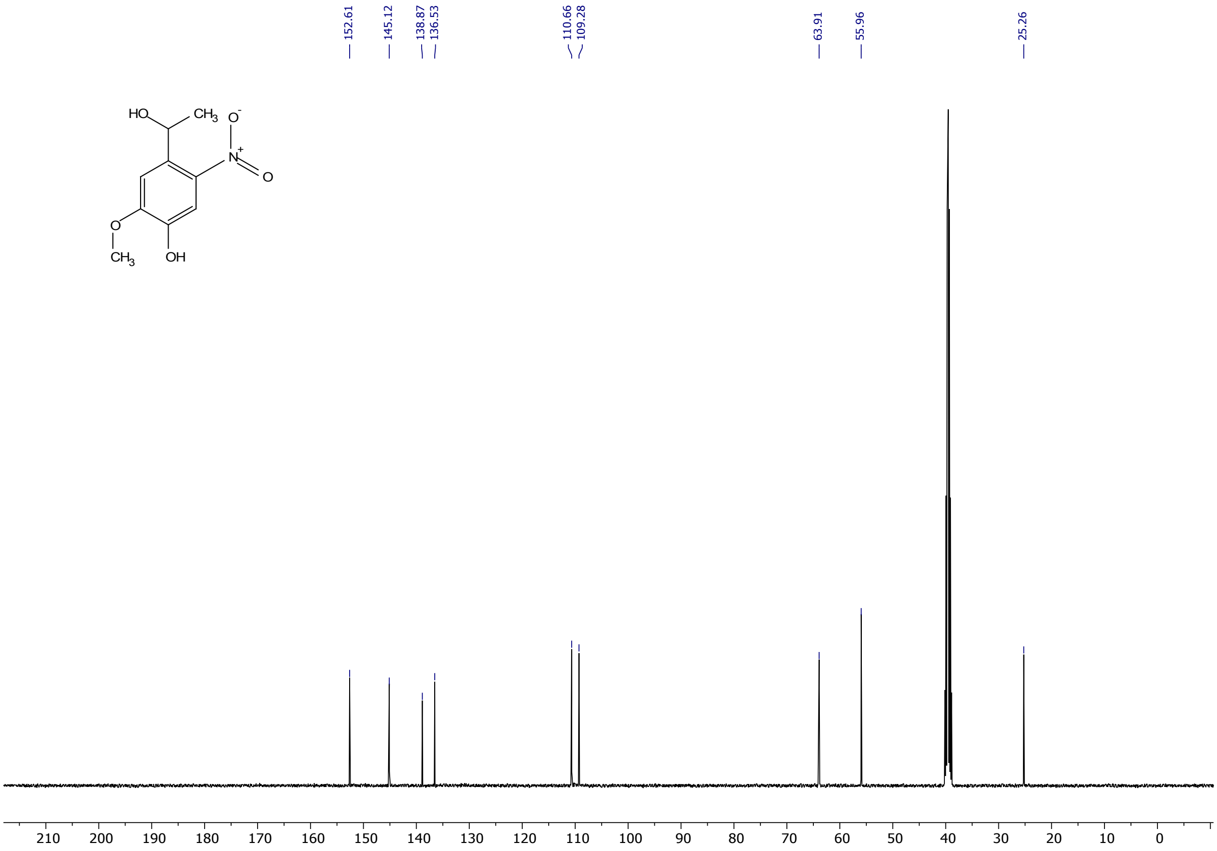

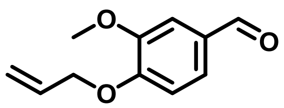

4-(allyloxy)-3-methoxybenzaldehyde (SI25)

K_2CO_3 (8.35 g, 60 mmol, 1.2 eq.) was added to vanillin (7.6 g, 50 mmol) in anhydrous acetonitrile/DMF (9:1, 200 mL) under nitrogen. Then, allyl bromide (4.8 mL, 55 mmol, 1.1 eq.) was added dropwise and the reaction was stirred for 3 h at 25 °C before being heated to 50 °C for another 2 h. The suspension was filtered and the solvent was evaporated. **SI25** was isolated by FCC (1:1:3 CH_2Cl_2/EtOAc/hexane; Rf = 0.45) in 98% yield (9.44 g, 49.1 mmol).

^{1}H NMR (400 MHz, $CDCl_3$) δ 9.82 (s, 1H), 7.46 – 7.36 (m, 2H), 6.95 (d, J = 8.2 Hz, 1H), 6.06 (ddd, J = 22.1, 10.6, 5.3 Hz, 1H), 5.42 (d, J = 17.4 Hz, 1H), 5.32 (d, J = 10.5 Hz, 1H), 4.68 (dd, J = 5.2, 1.4 Hz, 2H), 3.91 (s, 3H).

^{13}C NMR (101 MHz, $CDCl_3$) δ 191.0, 153.6, 150.0, 132.3, 130.3, 126.7, 118.9, 112.0, 109.6, 69.9, 56.1.

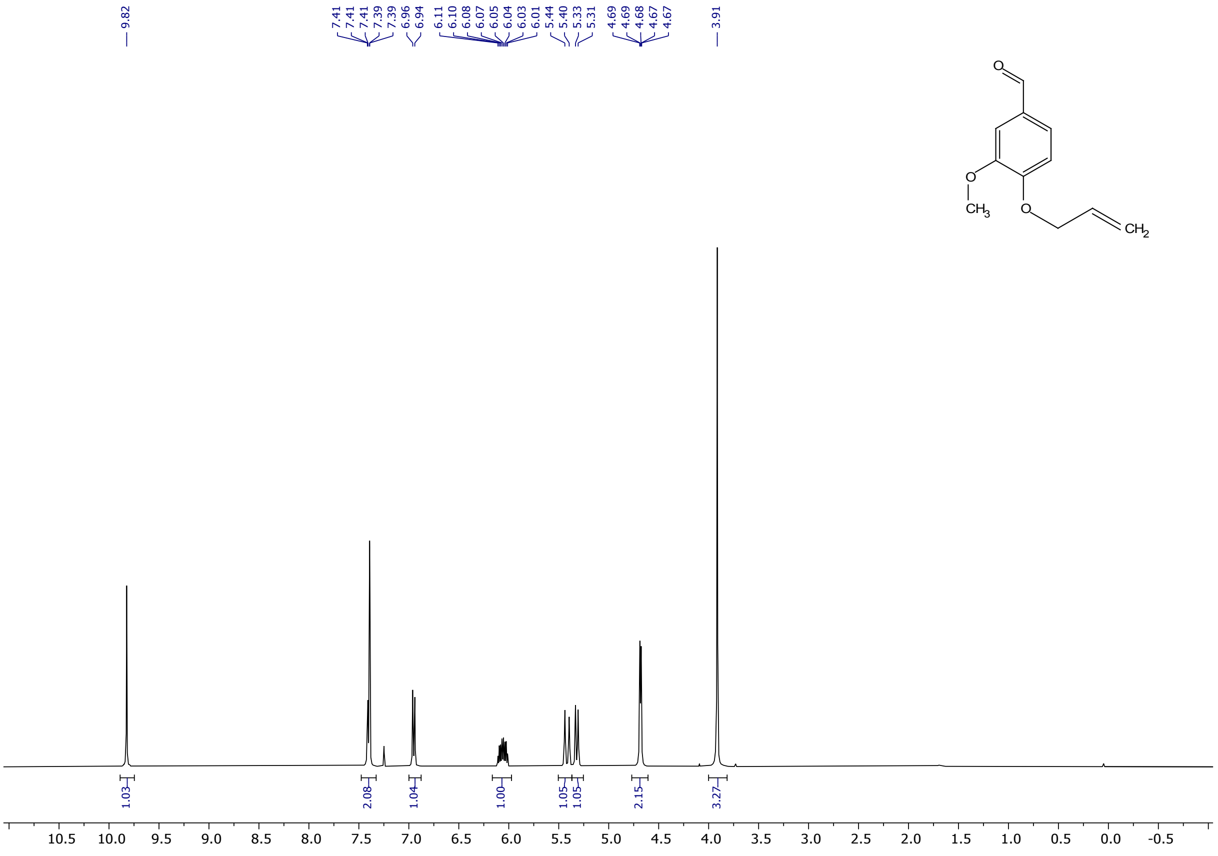

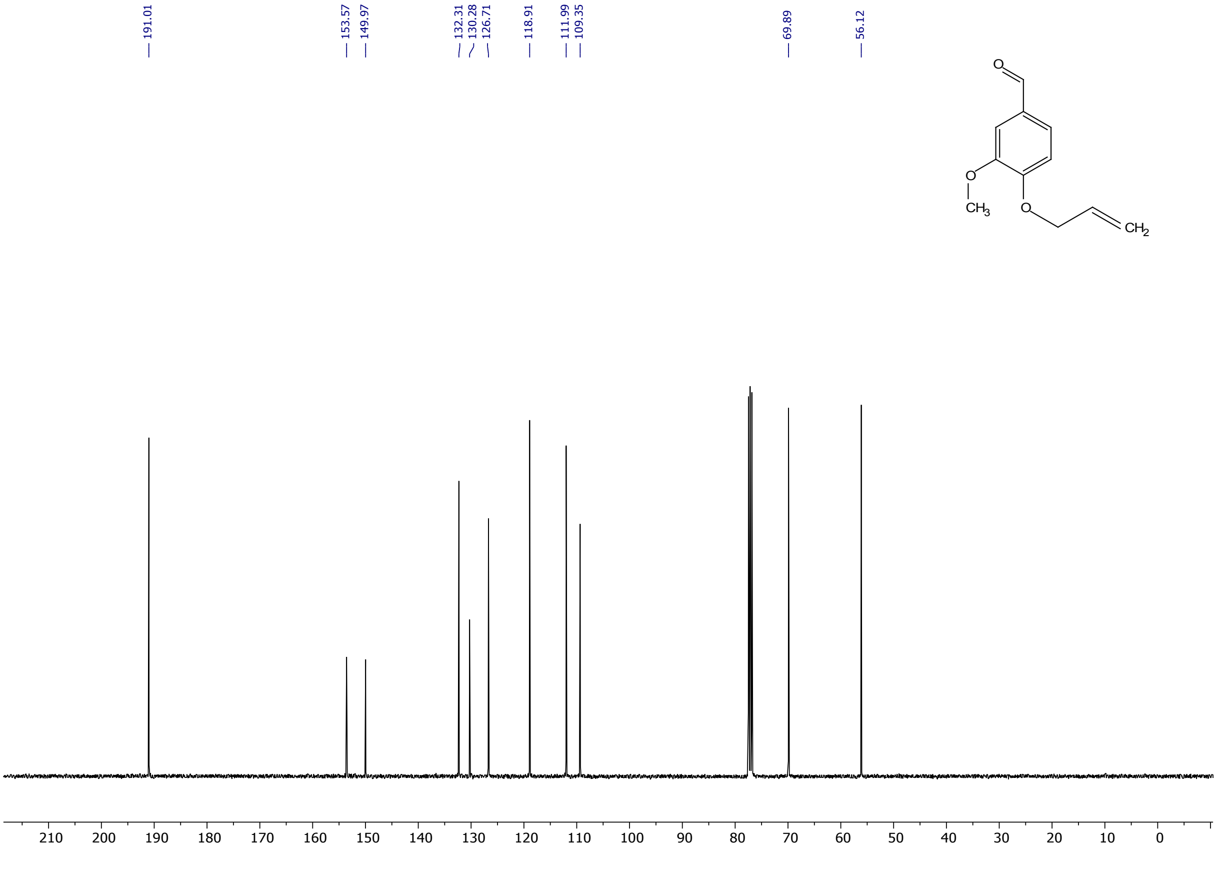

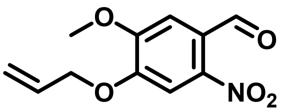

4-(Allyloxy)-5-methoxy-2-nitrobenzaldehyde (SI27)

KNO_3 (53.2 g, 1.1 eq.) was added portion-wise to **SI25** (9.26 g, 48.2 mmol) in TFA (1 M) under nitrogen at - 10 °C. The resultant dark solution was warmed to 60 °C and stirred for 4 h. CH_2Cl_2 (200 mL) was added at RT followed by water (200 mL). The phases were separated and the aqueous phase was extracted three times with CH_2Cl_2. The combined organic phases were washed with dilute $NaHCO_3$ solution, dried over Na_2SO_4 and the solvent was removed under reduced pressure. **SI27** was isolated by FCC (3:1:1 hexanes/EA/ CH_2Cl_2; Rf = 0.5) in 92% yield as a yellow solid (10.5 g, 44.3 mmol).

^{1}H NMR (400 MHz, DMSO-d6) δ 10.19 (s, 1H), 7.71 (s, 1H), 7.38 (s, 1H), 6.06 (ddt, J = 17.3, 10.6, 5.3 Hz, 1H), 5.44 (dq, J = 17.3, 1.6 Hz, 1H), 5.33 (dq, J = 10.5, 1.4 Hz, 1H), 4.80 (dt, J = 5.4, 1.5 Hz, 2H), 3.96 (s, 3H).

^{13}C NMR (101 MHz, DMSO-d6) δ 188.7, 152.8, 150.7, 143.5, 132.5, 124.9, 118.8, 110.2, 108.7, 69.7, 56.5.

HRMS (ESI): $C_{11}H_{14}NO_6$ $[M+H_2O+H]^+$; calculated: 256.08211, found: 256.0976.

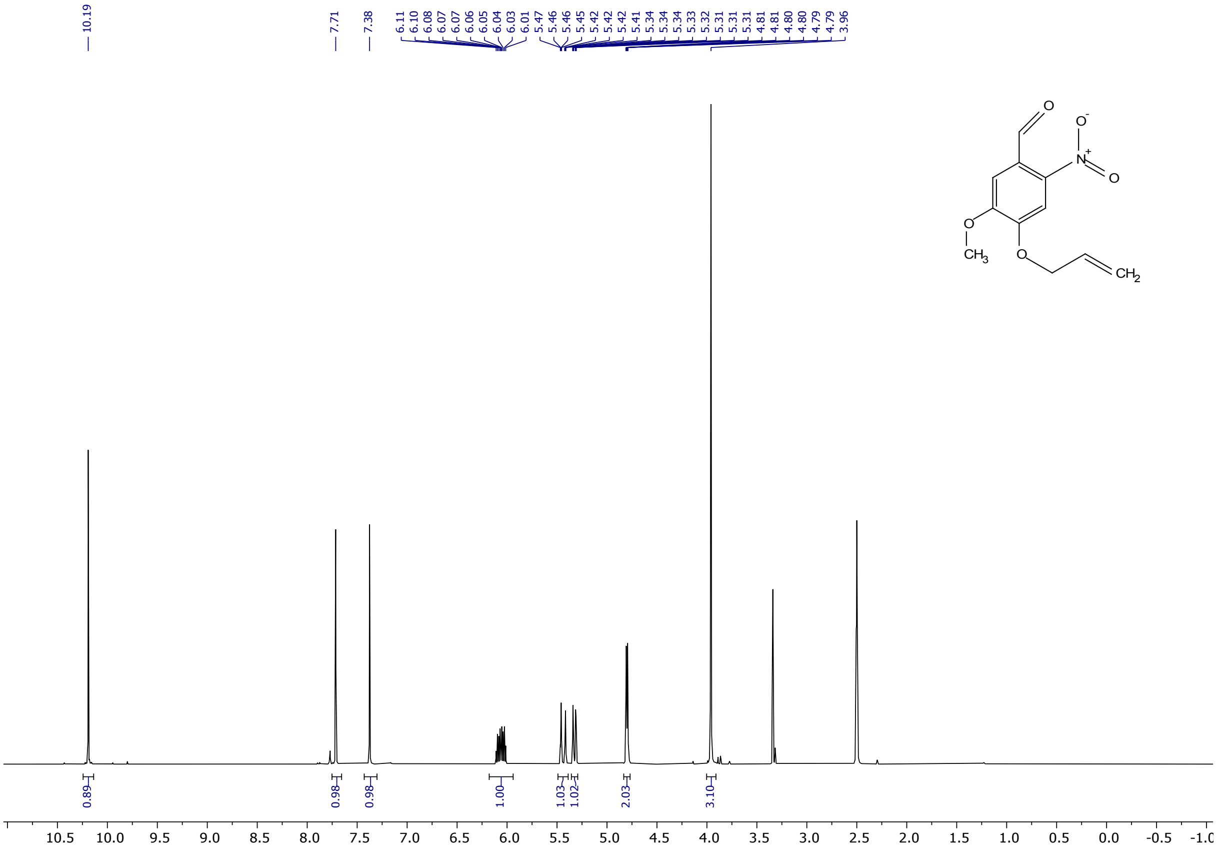
10.19
7.71
7.38
6.11 6.10 6.08 6.07 6.07 6.06 6.05 6.04 6.03 6.01
5.47 5.46 5.46 5.45 5.42 5.42 5.42 5.41 5.34 5.34 5.34 5.33 5.32 5.31 5.31 5.31
4.81 4.81 4.80 4.80 4.79 4.79
3.96
0.89
0.98
0.98
1.00
1.03
1.02
2.03
3.10
O
O-
N+
O
O
CH3
O
CH2
10.5 10.0 9.5 9.0 8.5 8.0 7.5 7.0 6.5 6.0 5.5 5.0 4.5 4.0 3.5 3.0 2.5 2.0 1.5 1.0 0.5 0.0 -0.5 -1.0

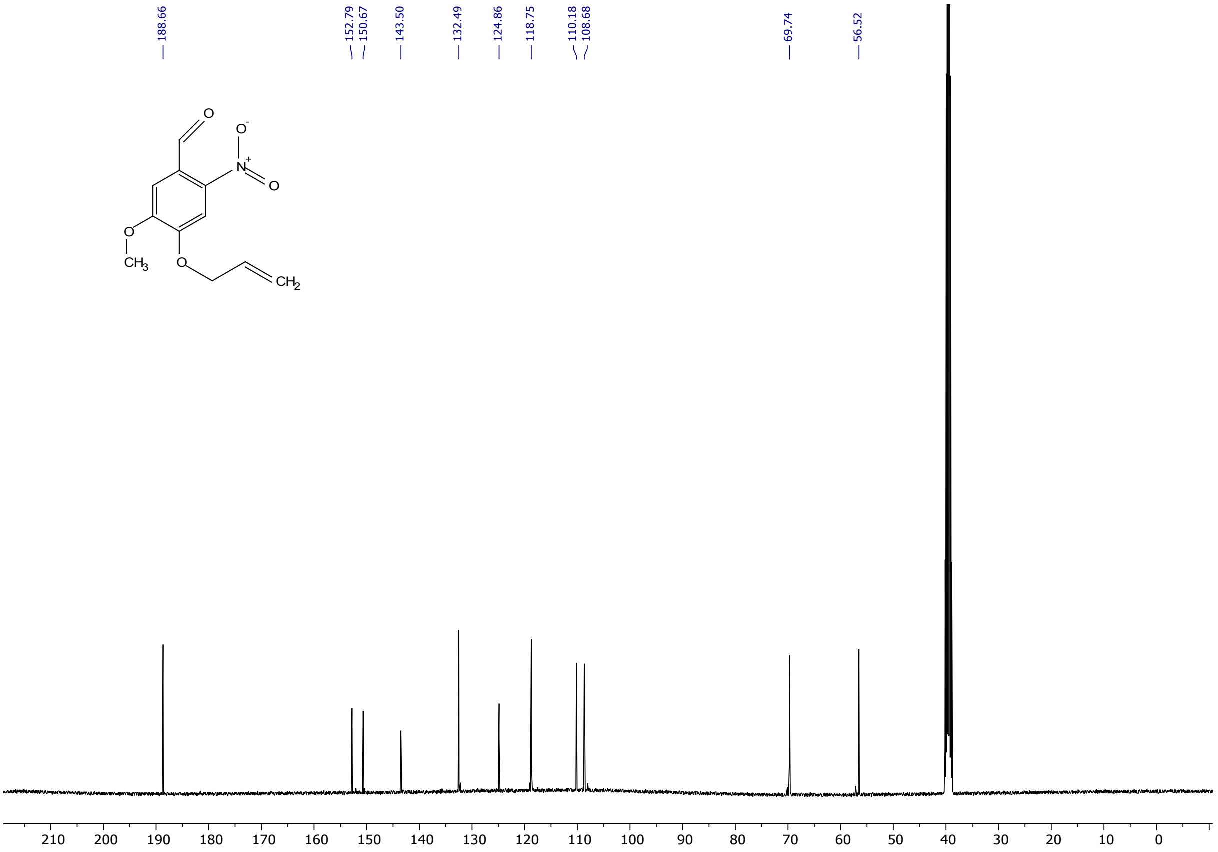
188.66
152.79
150.67
143.50
132.49
124.86
118.75
110.18
108.68
69.74
56.52
O
O-
N+
O
O
CH3
O
CH2
210 200 190 180 170 160 150 140 130 120 110 100 90 80 70 60 50 40 30 20 10 0

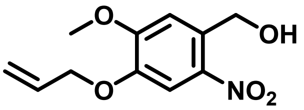

(4-(Allyloxy)-5-methoxy-2-nitrophenyl)methanol (SI28)

$NaBH_4$ (2.13 g, 56.4 mmol, 2 eq.) was added in portions to ketone **SI27** (6.7 g, 28.2 mol) in EtOH/iPrOH/CH_2Cl_2 (2:2:1, 0.1 M) under nitrogen at 0 °C. The reaction was slowly warmed to 25 °C and kept at that temperature for another 2 h. The reaction cooled to 0 °C and stopped by the addition of acetone. The solvent was removed under reduced pressure, the residue was triturated in CH_2Cl_2 (100 mL) and washed with citric acid solution (100 mL, 10% w/w). The aqueous phase was extracted with CH_2Cl_2. The combined organic phases were dried over Na_2SO_4 and the solvent was removed under reduced pressure. **SI28** was isolated by FCC (4:5:1 hexane/EtOAc/CH_2Cl_2; Rf = 0.34) in 80% yield as a yellow solid (5.43 g, 22.7 µmol).

^{1}H NMR (400 MHz, DMSO-d6) *δ* 7.68 (s, 1H), 7.39 (s, 1H), 6.05 (ddt, *J* = 17.3, 10.5, 5.3 Hz, 1H), 5.59 (t, *J* = 5.4 Hz, 1H), 5.41 (dq, *J* = 17.3, 1.7 Hz, 1H), 5.29 (dq, *J* = 10.5, 1.5 Hz, 1H), 4.82 (dd, *J* = 5.5, 0.8 Hz, 2H), 4.66 (dd, *J* = 5.3, 1.5 Hz, 2H), 3.91 (s, 3H).

^{13}C NMR (101 MHz, DMSO-d6) *δ* 153.8, 145.6, 138.3, 134.4, 133.1, 118.2, 109.7, 109.3, 69.3, 60.2, 56.1.

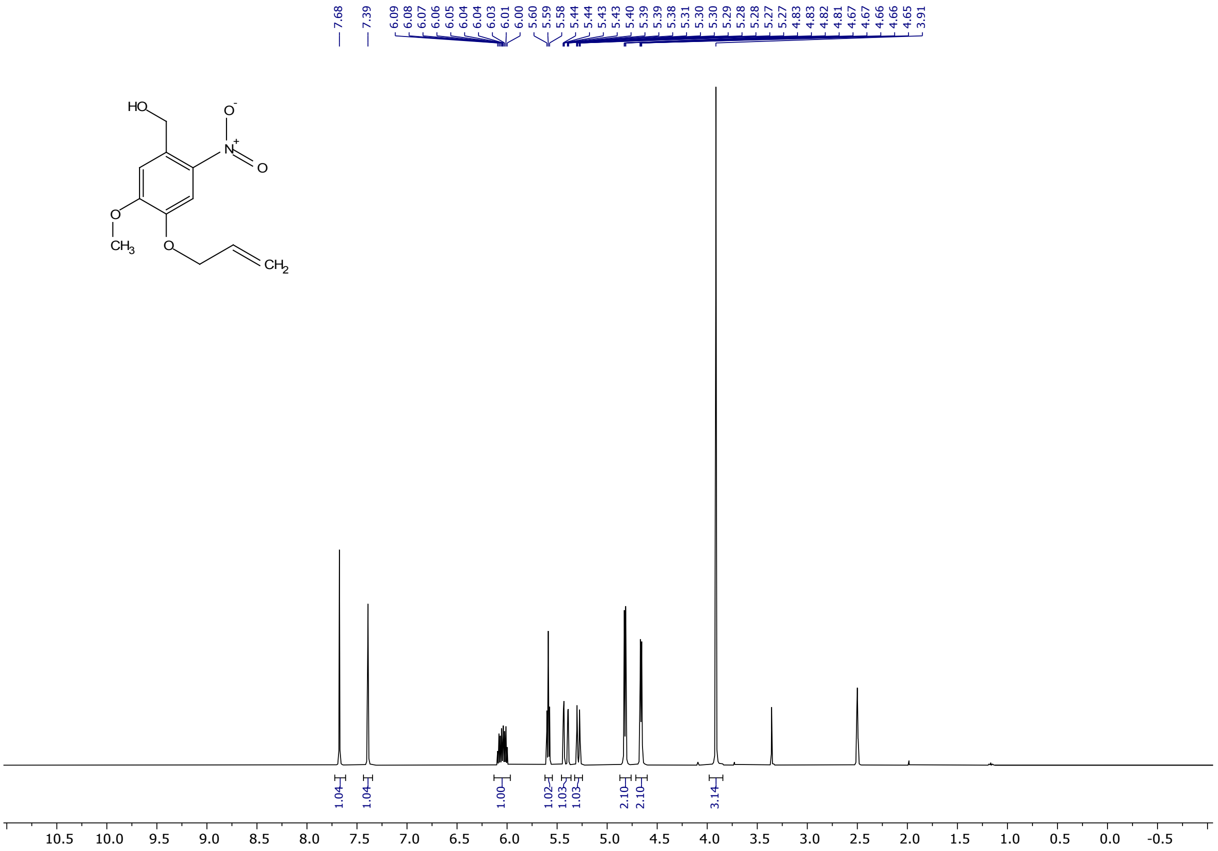
HO
O
N
O
O
CH3
O
CH2
7.68
7.39
6.09
6.08
6.07
6.06
6.05
6.04
6.04
6.03
6.01
6.00
5.60
5.59
5.58
5.44
5.44
5.43
5.43
5.40
5.39
5.39
5.38
5.31
5.30
5.30
5.29
5.28
5.28
5.27
5.27
4.83
4.83
4.82
4.81
4.67
4.67
4.66
4.65
3.91
1.04
1.04
1.00
1.02
1.03
1.03
2.10
2.10
3.14
10.5 10.0 9.5 9.0 8.5 8.0 7.5 7.0 6.5 6.0 5.5 5.0 4.5 4.0 3.5 3.0 2.5 2.0 1.5 1.0 0.5 0.0 -0.5

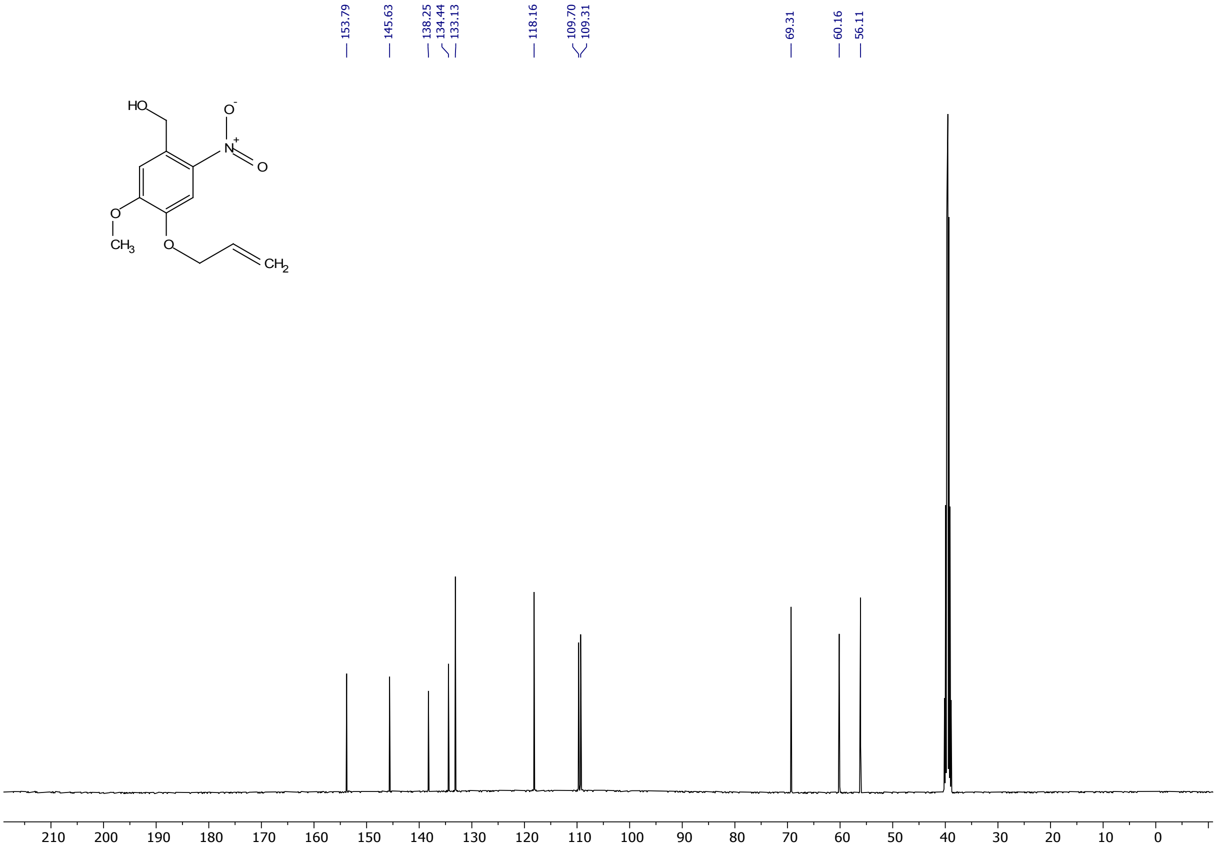
HO
O
N
O
O
CH3
O
CH2
153.79
145.63
138.25
134.44
133.13
118.16
109.70
109.31
69.31
60.16
56.11
210 200 190 180 170 160 150 140 130 120 110 100 90 80 70 60 50 40 30 20 10 0

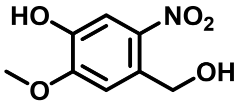

4-(Hydroxymethyl)-2-methoxy-5-nitrophenol (SI30)

$Pd(PPh_3)_4$ (496 mg, 426 µmol, 0.02 eq.) and K_2CO_3 (8.83 g, 63.9 mmol, 3 eq) were added to alcohol **SI28** (5.1 g, 21.3 mol) in MeOH (0.1 M) under nitrogen. The reaction was stirred for 16 h, diluted with CH_2Cl_2 and treated with citric acid (10% w/v aq., 300 mL). The aqueous phase was extracted with CH_2Cl_2 three times and the combined organic phases were dried over Na_2SO_4. The solvent was removed under reduced pressure and **SI30** was isolated by FCC (CH_2Cl_2/EtOAc 1:1; Rf = 0.5) in 79% yield as a yellow solid (3.35 g, 16.8 mmol).

^{1}H NMR (400 MHz, DMSO-d6) δ 9.92 (s, 1H), 7.55 (s, 1H), 7.33 (s, 1H), 5.49 (t, J = 5.4 Hz, 1H), 4.79 (d, J = 5.0 Hz, 2H), 3.90 (s, 3H).

^{13}C NMR (101 MHz, DMSO-d6) δ 162.8, 155.0, 148.4, 142.4, 121.3, 120.0, 70.2, 66.0.

HRMS (ESI): $C_8H_9NaNO_5$ $[M+Na]^+$; calculated: 222.0378, found: 222.0744.

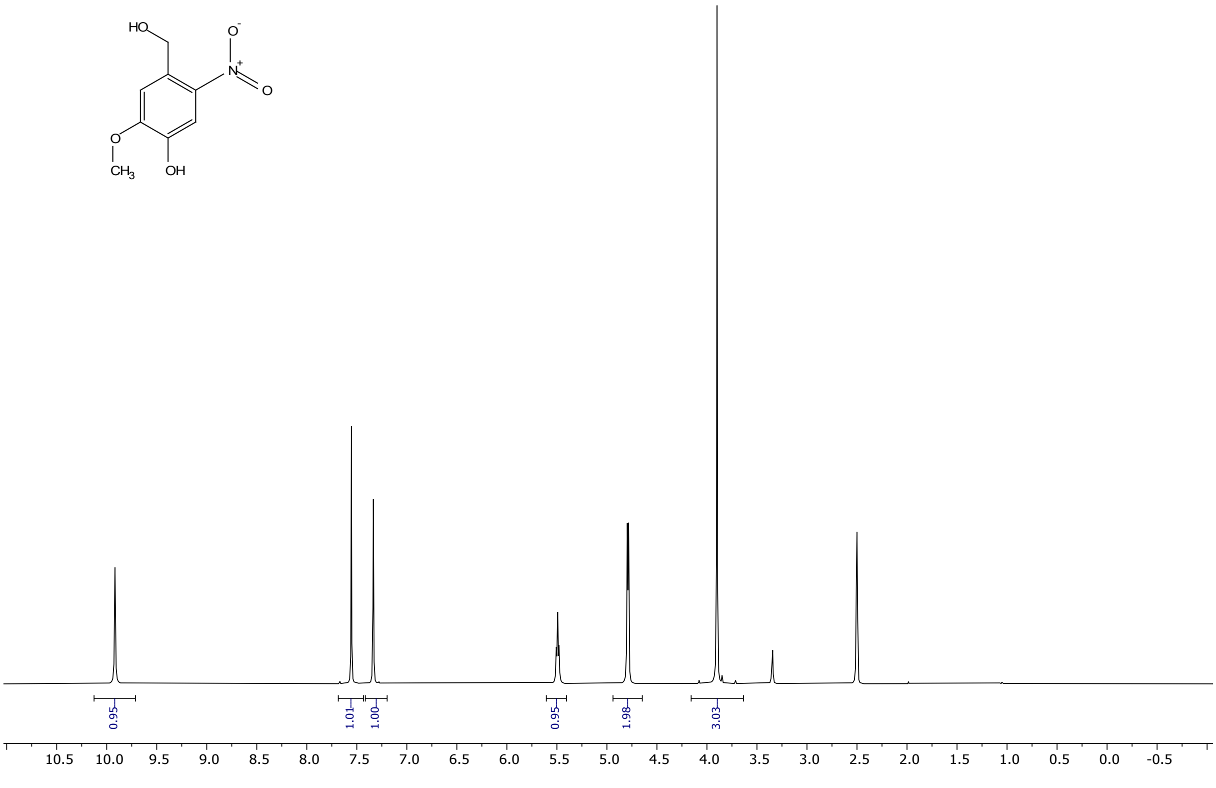

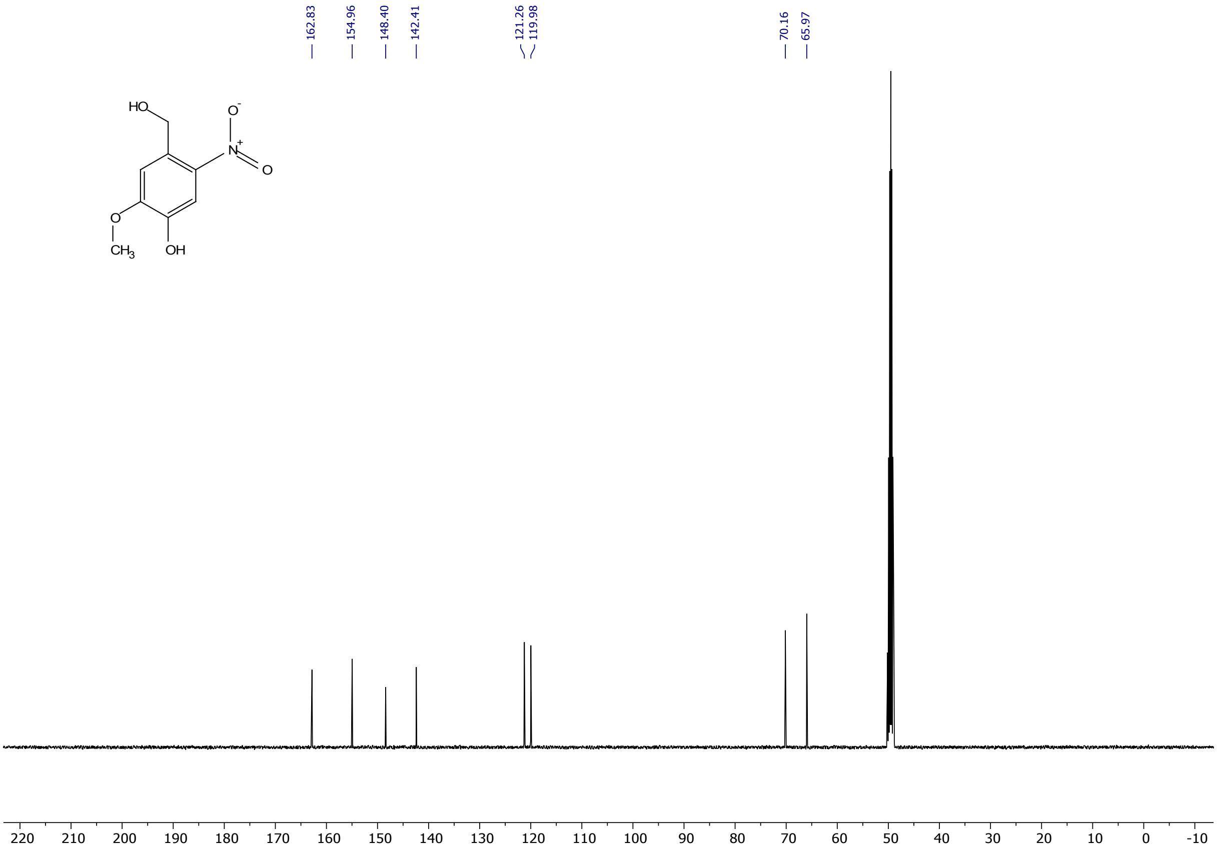

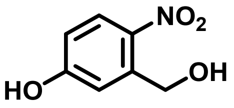

3-(Hydroxymethyl)-4-nitrophenol (SI32)

$NaBH_4$ (1.26 g, 35.9 mmol, 1.2 eq.) was added in portions to 5-hydroxy-2-nitro-benzaldehyd (5 g, 29.9 mmol) under nitrogen in MeOH (0.5 M) at 0 °C. The reaction was slowly warmed to 25 °C and kept at that temperature for another 16 h. The reaction was diluted with CH_2Cl_2 cooled to 0 °C and stopped by the addition sat. aq. NH_4Cl. The phases were separated and aqueous phase was extracted with CH_2Cl_2 twice. The combined organic phases were dried over Na_2SO_4 and the solvent was removed under reduced pressure. **SI32** was isolated by FCC (4:5:1 hexane/EtOAc/CH_2Cl_2; Rf = 0.33) in 84% yield as a yellow solid (4.23 g, 25.0 µmol).

^{1}H NMR (400 MHz, DMSO-d6) *δ* 10.90 (br. s, 1H), 8.05 (d, *J* = 8.9 Hz, 1H), 7.26 (dd, *J* = 2.7, 1.2 Hz, 1H), 6.80 (dd, *J* = 9.0, 2.8 Hz, 1H), 5.51 (t, *J* = 5.5 Hz, 1H), 4.82 (d, *J* = 4.8 Hz, 2H).

^{13}C NMR (101 MHz, DMSO-d6) *δ* 163.1, 143.0, 138.0, 127.9, 113.9, 113.8, 60.4.

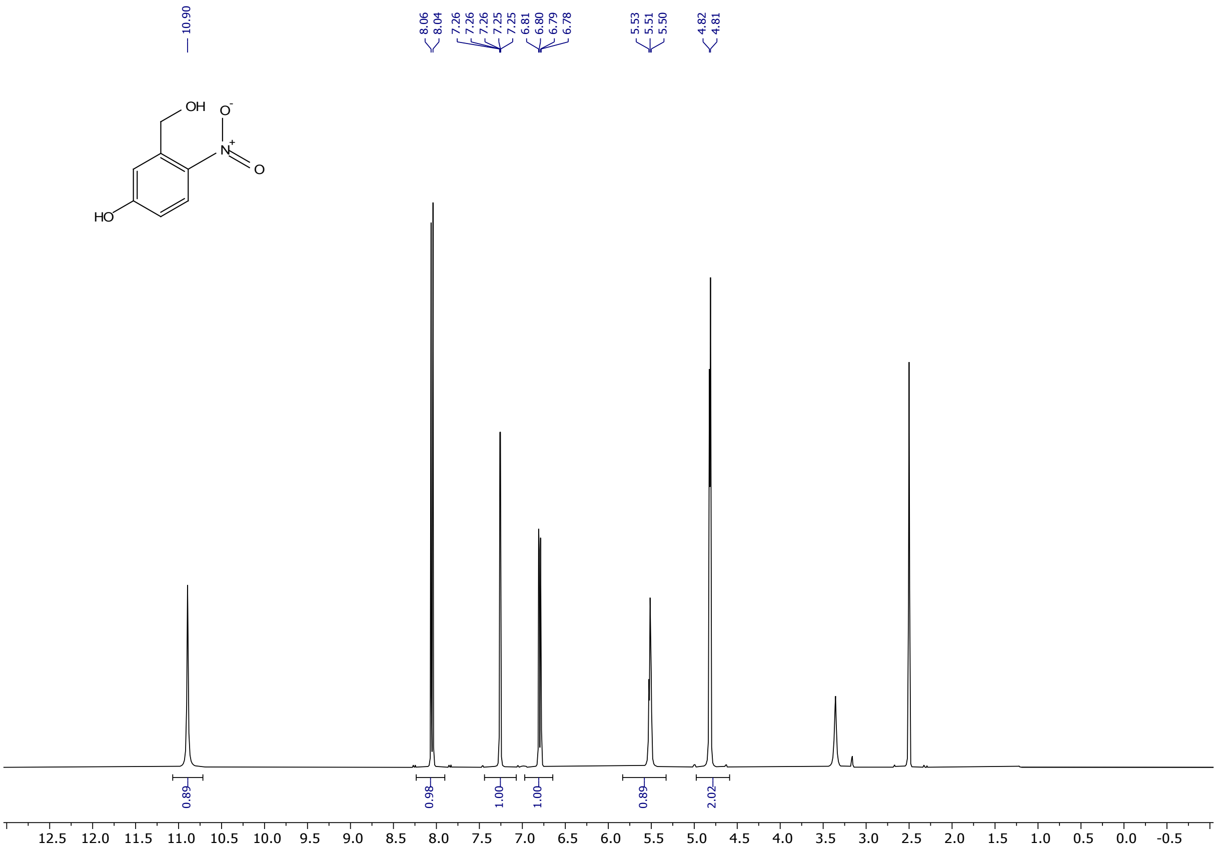
10.90
8.06
8.04
7.26
7.26
7.26
7.25
7.25
6.81
6.80
6.79
6.78
5.53
5.51
5.50
4.82
4.81
OH
O
N
O
HO
0.89
0.98
1.00
1.00
0.89
2.02
12.5 12.0 11.5 11.0 10.5 10.0 9.5 9.0 8.5 8.0 7.5 7.0 6.5 6.0 5.5 5.0 4.5 4.0 3.5 3.0 2.5 2.0 1.5 1.0 0.5 0.0 -0.5

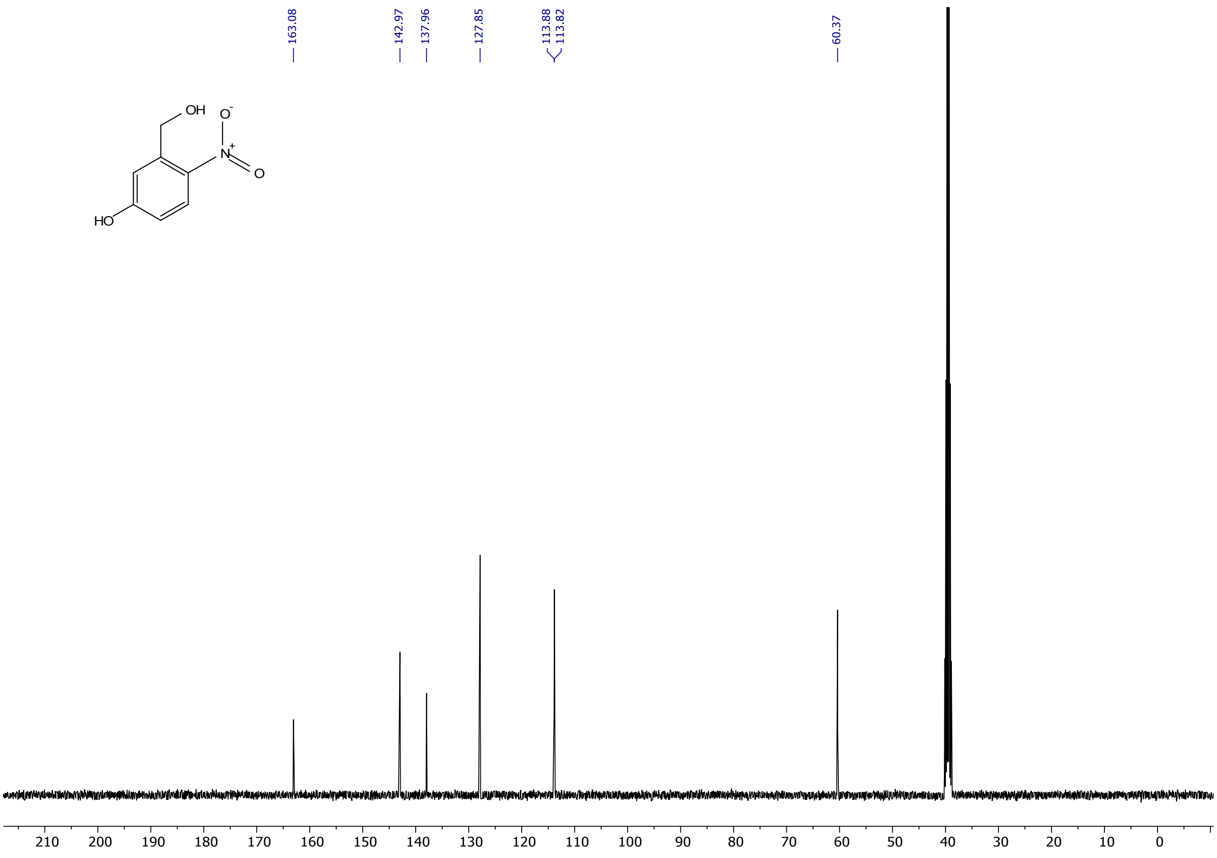
163.08
142.97
137.96
127.85
113.88
113.82
60.37
OH
O
N
O
HO
210 200 190 180 170 160 150 140 130 120 110 100 90 80 70 60 50 40 30 20 10 0

3 Automated Glycan Assembly

3.1 Standard solutions

Building block: Building blocks were dissolved in anhydrous CH_2Cl_2 (1 mL / coupling cycle).

TMSOTf: 80 mL anhydrous CH_2Cl_2, TMSOTf (0.9 mL, 4.9 mmol, 0.06 M).

NIS/TfOH activator: 30 mL anhydrous CH_2Cl_2, 15 mL anhydrous dioxane and NIS (1.57 g, 7 mmol, 0.015 M) was added triflic acid (67 µL, 2.6 µmol, 0.006 M) at 0°C. The solution was kept at 0 °C during the synthesis.

Fmoc deprotection: 80 mL anhydrous DMF, 20 mL piperidine (or 20 mL triethylamine for selective carbonate over carbamate deprotection).

Lev deprotection: N_2H_4·AcOH (725 mg, 7.87 mmol) in 50 mL of pyridine/acetic acid/water (20:16:1).

Capping: 50 mL anhydrous CH_2Cl_2, acetic anhydride (6 mL, 55 mmol) and methanesulfunic acid (1.2 mL, 18.5 mmol).

Pyridine wash: 90 mL anhydrous DMF and 10 mL pyridine.

TES quench: 5 mL anhydrous CH_2Cl_2, 550 µL triethylsilane.

3.2 Automation programs

A) First coupling

The building block delivery line to the reaction vessel was primed. The building block solution (6 equiv. of BB in 1 mL of CH_2Cl_2) was delivered to the reaction vessel. After the set temperature (T_1) was reached, the reaction was started by adding the TMSOTf solution (2 equiv. from standard solution). The reaction was performed in two thermal steps. Starting at T_1, the reagents incubate for a time t_1, then the temperature is linearly increased by a rate of 4 °C/min to a T_2, incubating for a t_2 (times and temperature are building block dependent). The temperature is adjusted to T_3 and 1 mL of triethylsilane solution (30 equiv. in 1 mL CH_2Cl_2) was delivered from an independent line via an auxiliary delivery system. The reagents were incubated for a t_3, then, the excess BB solution was recovered by the fraction collector system. The fraction collection line was wash with 2 mL of CH_2Cl_2:dioxane, and the resin was rinsed with CH_2Cl_2 (3 mL for 30 s). The resin is finally washed with CH_2Cl_2 (two times 3 mL for 10 s).

Table 9: Automation protocol for the attachment of alcohols to the TCAI-traceless acceptor resin.

Action	Cycles	Solution	Amount	T [°C]	Incubation
Cooling	-	-	-	T_1	-
BB Delivery	1	BB solution	1 mL	T_1	3 min
AW Delivery	1	TMSOTf solution	1 mL	T_1	3 min
Coupling reaction [a]	1	-	-	T_1 to T_2	t_1<br>t_2
Delivery	1	Capping solution	1 mL	T_3	12s
Quench reaction	1	-	1 mL	T_3	t_3
Fraction collection	-	-	-	T_3	15 s
Washing fr. col.	1	CH_2Cl_2:dioxane	2 mL	T_3	2 min
Wash	1	CH_2Cl_2	3 mL	T_3	30 s
Wash	2	CH_2Cl_2	3 mL	T_3	10 s

[a] There is a five-minute minimum offset time between T1 and T2 for temperature adjustment.

Auxiliary top delivery

An independent inlet delivered pneumatically reagents to the reaction vessel controlled by a rotary valve for pathway selection and one-way solenoid valve for dosing the reagents by time aperture, filling a reservoir loop and pushing with argon the solution towards the reactor.

B) Reactor vessel wash

Reactor vessel and resin were washed with DMF (three times 3 mL) and CH_2Cl_2 (three times 3 mL) incubating for 5 min at each step.

Table 10: Automation protocol for washing the reactor vessel from the top.

Action	Cycles	Solution	Amount	T [°C]	Incubation
Cooling	-	-	-	25	-
Delivery DMF	3	CH_2Cl_2	3 mL	25	5 min
Delivery CH_2Cl_2	3	TMSOTf solution	3 mL	25	5 min

C) Acidic wash

The resin was suspended in CH_2Cl_2 (2 mL) and the temperature of the reaction vessel was adjusted to - 20 °C. Upon reaching the low temperature, TMSOTf solution (1 mL, 0.06 mmol) was added dropwise to the reaction vessel. After bubbling for 3 min, the solution was drained and the resin was washed with DCM (2 mL) for 25 s.

Table 11: Automation protocol for acidic washing.

Action	Cycles	Solution	Amount	T (°C)	Incubation
Cooling	-	-	-	- 20	-
Delivery CH_2Cl_2	1	CH_2Cl_2	2 mL	- 20	-
Delivery AW	1	TMSOTf solution	1 mL	- 20	3 min
Wash	1	CH_2Cl_2	1 mL	- 20	25 s

D) Capping

The resin was washed with DMF (two times 2 mL) and the temperature of the reaction vessel was adjusted to 25 °C. Pyridine washing solution (2 mL) was delivered and incubated for 1 min. The reaction solution was drained and the resin was washed with CH_2Cl_2 (three times 3 mL for incubating 25 s). Capping solution (4 mL) was delivered and incubated for 20 min. The reaction solution was drained and the resin was washed

with CH_2Cl_2 (three times 3 mL incubating 25 s). The temperature of the reaction vessel was adjusted to - 20 °C for the next module.

Table 12: Automation protocol for capping via acetylation under acidic conditions.

Action	Cycles	Solution	Amount	T [°C]	Incubation
Cooling	-	-	-	25	-
Wash	2	DMF	2 mL	25	25 s
Pyridine wash	1	Pyridine wash	2 mL	25	1 min
Wash	3	CH_2Cl_2	2 mL	25	25 s
Deliver capping	1	Capping solution	4 mL	25	20 min
Wash	3	CH_2Cl_2	2 mL	25	25 s

E) Fmoc deprotection

The resin was washed with DMF (three times 2 mL incubating 25 s) and the temperature of the reaction vessel was adjusted to 50 °C. Fmoc deprotection solution (2 mL) was delivered to the reaction vessel. After 5 min of incubation, the reaction solution was drained

and the resin was washed with DMF (three times 2 mL incubating 25 s) and then CH_2Cl_2 (five times 2 mL incubating 25 s) at 25 °C.

Table 13: Automation protocol for Fmoc deprotection.

Action	Cycles	Solution	Amount	T [°C]	Incubation
Cooling	-	-	-	50	-
Wash	3	DMF	2 mL	50	25 s
Deliver Fmoc depr.	3	Fmoc deprotect.	2 mL	50	5 min
Wash		DMF	2 mL	50	25 s
Wash	5	CH_2Cl_2	2 mL	25	25 s

E2) Lev deprotection

The temperature of the reaction vessel was adjusted to 25 °C and the resin was washed with CH_2Cl_2 (three times 2 mL for 15 s) and the. Lev deprotection solution (2 mL) was delivered to the reaction vessel and the reaction was incubated for five minutes. The reaction solution was drained from the reactor vessel. These two steps were repeated three times. Finally, the resin was washed with DMF, THF, and CH_2Cl_2 (three times 2 mL for 15 s, respectively).

Table 14: Automation protocol for Lev deprotection.

Action	Cycles	Solution	Amount	T [°C]	Incubation
Cooling	-	-	-	25	-
Wash	3	CH_2Cl_2	2 mL	25	15 s
Deliver Fmoc depr.		Lev deprot.	2 mL	25	5 min
Wash	3	DMF	3 mL	25	15 s
Wash	3	THF	3 mL	25	15 s
Wash	5	CH_2Cl_2	2 mL	25	15 s

F) Phosphate coupling

After the set temperature (T_1) was reached, the resin was washed with CH_2Cl_2 (five times 5 mL incubating for 10 s). TMSOTf solution was delivered, bubbling for 3 min before draining. The resin was washed with CH_2Cl_2 (3 mL incubating for 10 s), the reactor vessel was drained and building block solution (4 equiv. of BB in 1 mL of CH_2Cl_2) was delivered. After the set temperature (T_1) was reached, the reaction was started by adding TMSOTf solution (1 mL over 3 min). The reaction was performed in two thermal steps. Starting at T_1, the reagents incubate for a time t_1, then the temperature is linearly increased by a rate of 4 °C/min to a T_2, incubating for a t_2 (times and temperature are building block dependent). The reactor vessel was drained and the resin was rinsed with CH_2Cl_2 (3 mL for 30 s). The resin is finally washed with CH_2Cl_2 (two times 3 mL for 10 s).

Table 15: Automation protocol for coupling a phosphate building block.

Action	Cycles	Solution	Amount	T [°C]	Incubation
Cooling	-	-	-	T_1	-
Wash	5	CH_2Cl_2	5 mL	T_1	10 s
Delivery AW	1	TMSOTf solution	1 mL	T_1	3 min
Wash	1	CH_2Cl_2	3 mL	T_1	10 s
Delivery BB	1	BB solution	1 mL	T_1	3 min
Delivery activator	1	NIS/TfOH	1 mL	T_1	3 min
Coupling reaction [a]	1	-	-	T_1 to T_2	t_1 t_2
Wash	1	CH_2Cl_2	3 mL	T_3	30 s
Wash	2	CH_2Cl_2	3 mL	T_3	10 s

[a] There is a five-minute minimum offset time between T1 and T2 for temperature adjustment.

G) Thioglycoside coupling

After the set temperature (T_1) was reached, the resin was washed with CH_2Cl_2 (five times 5 mL incubating for 10 s). TMSOTf solution was delivered, bubbling for 3 min before draining. The resin was washed with CH_2Cl_2 (3 mL incubating for 10 s), the reactor vessel was drained and building block solution (6 equiv of BB in 1 mL of CH_2Cl_2) was delivered. After the set temperature (T_1) was reached, the reaction was started by adding NIS/TfOH solution (1 mL over 3 min). The reaction was performed in two thermal steps. Starting at T_1, the reagents incubate for a time t_1, then the temperature is linearly increased by a rate of 4 °C/min to a T_2, incubating for a t_2 (times and temperature are building block dependent). The reactor vessel was drained and the resin was rinsed with CH_2Cl_2 (3 mL for 30 s) and CH_2Cl_2:dioxane (2 mL for 30 s). The resin is finally washed with CH_2Cl_2 (2x 3 mL for 10 s).

Table 16: Automation protocol for coupling a thioglycoside building block.

Action	Cycles	Solution	Amount	T [°C]	Incubation
Cooling	-	-	-	T_1	-
Wash	5	CH_2Cl_2	5 mL	T_1	10 s
Delivery AW	1	TMSOTf solution	1 mL	T_1	3 min
Wash	1	CH_2Cl_2	3 mL	T_1	10 s
Delivery BB	1	BB solution	1 mL	T_1	3 min
Delivery activator	1	NIS/TfOH	1 mL	T_1	3 min
Coupling reaction [a]	1	-	-	T_1 to T_2	t_1<br>t_2
Wash	1	CH_2Cl_2	3 mL	T_3	30 s
Wash	1	CH_2Cl_2:dioxane	2 mL	T_3	30 s
Wash	2	CH_2Cl_2	3 mL	T_3	10 s

[a] There is a five-minute minimum offset time between T1 and T2 for temperature adjustment.

H) Amide Coupling

Carboxylic acid (10 equiv.) and PyBOP (10 equiv.) were dissolved in DMF (4 mL), then NMM (16 equiv.) was added. This pre-activation was performed for 4 min before the mixture was added to the resin in a 5 mL fritted syringe and shaken at room temperature for 90 min at 400 rpm. The resin is washed with CH_2Cl_2 (three times 1 min) and DMF (three times 1 min).

Fmoc deprotection was performed in three cycles subjecting the resin in a fritted syringe to 5 mL a 20% v/v solution of piperidine in DMF and shaking at room temperature for 90 min at 400 rpm. The resin was washed with CH_2Cl_2 (three times 1 min) and DMF (three times 1 min).

Every step was performed under the exclusion of light via aluminum foil.

H) Methanolysis

On resin: The resin in a 5 mL fritted syringe was washed with anhydrous THF (three times 3 mL), then 5 mL of a 1:9 v/v solution of NaOMe (0.5 M in MeOH) and anhydrous THF were added and the reaction was shaken at room temperature for 16 h at 400 rpm. The solvent was removed and the resin was washed with 1% acetic acid in THF (three times 5 mL), CH_2Cl_2 (two times 5 mL) and dried under vacuum.

In solution: The crude or purified glycans from photocleavage were dissolved in THF/MeOH/H2O (3:1:1, 5 mL) and 20 equiv. of NaOH solution (1 M in H_2O) were added dropwise. The reaction was stirred at RT for 16 h and neutralized by dropwise addition of acetic acid. The solvent was removed under reduced pressure and the crude was used directly for hydrogenolysis.

I) Photocleavage

The resin was placed in a 5 mL fritted syringe with an 8 mm egg-shaped stirring bar. DMF (5 mL) was added and the reaction was placed in 5 cm distance of a Kessil PR160L at 3rd intensity and stirred at 600 rpm for 16 h. Then, a PTFE filter (0.45 µm pore size) was fitted to the syringe and the solvent was collected. The resin was washed with DMF, DMF/DCM (1:1), CH_2Cl_2 (3 mL each). The combined solvent was evaporated under reduced pressure.

J) Hydrogenolysis

The crude, semi-deprotected glycans were dissolved in a mixture of *t*BuOH/H_2O /EtOAc (3:1:1, minimum 5 mL) and added a drop of acetic acid. Palladium on charcoal (5%, 100 mg) were added before the flask was sealed and the air exchanged for H_2 by bubbling for 15 min. The reaction was then stirred for 16 h at room temperature and filtered through a plug of celite. The filter was washed with *t*BuOH/H_2O (2:1, 10 mL) and MeCN/H_2O (1:1, 10 mL). If the product was prone to aggregation, sonification performed for 10 min before every filtering step. The combined solvent fractions were evaporated under reduced pressure.

K) Preparative HPLC Purification

Analytical and preparative HPLC was performed following the standardized protocol shown in Table 17. The sample was loaded in solvent ratio “A” onto the column followed by linearly ramping to solvent ratio “B”. Ratios and column-type is indicated for each compound.

Table 17: General protocol for HPLC purifications

Time	Solvent ratio eluent [%]
0	A
5	A
45	B
50	100
55	100
60	A
65	A

All final products were lyophilized on a Christ Alpha 2-4 LD plus freeze dryer before characterization.

NP-analytival: (YMC, diol-300-np column, 150 x 4.5 mm, 5µm), flow rate of 1.0 mL /min with EtOAc/hexanes as eluents. (RP-HPLC Agilent 1200 Series)
NP-analytival: (YMC, diol-300-np column, 150 x 20 mm, 5µm), flow rate of 15.0 mL /min with EtOAc/hexanes as eluents. (RP-HPLC Agilent 1200 Series)

C5-analytical: (Phenomenex, luna C5 column, 250 x 4.6 mm, 5 µm), flow rate of 1.0 mL /min with MeCN/H_2O (0.1% formic acid) as eluents. (RP-HPLC Agilent 1200 Series)
C18-analytical: (Phenomenex, Synergi column, 250 x 4.6 mm, 4 µm), flow rate of 1.0 mL /min with MeCN/H_2O (0.1% formic acid) as eluents. (RP-HPLC Agilent 1200 Series)
Hypercarb: (Hypercarb column, 150 x 4.6 mm, 3 µm) flow rate of 0.7 mL/min with MeCN/H_2O (0.1% formic acid) as eluents. (RP-HPLC Agilent 1200 Series)
C5-preparative: (Phenomenex, Luna C5 column, 250 x 10 mm, 5 µm), flow rate of 4.0 mL /min with MeCN/H_2O (0.1% formic acid) as eluents. (RP-HPLC Agilent 1260 Infinity II Series)
C18-preparative: (Phenomenex, Synergi column, 250 x 10 mm, 4 µm), flow rate of 4.0 mL /min with MeCN/H_2O (0.1% formic acid) as eluents. (RP-HPLC Agilent 1260 Infinity II Series)
Hypercarb-preparative: Hypercarb column, 150 x 10 mm, 5 µm), flow rate of 3 mL /min with MeCN/H_2O (0.1% formic acid) as eluents. (RP-HPLC Agilent 1260 Infinity II Series)

3.3 Products from automated glycan assembly

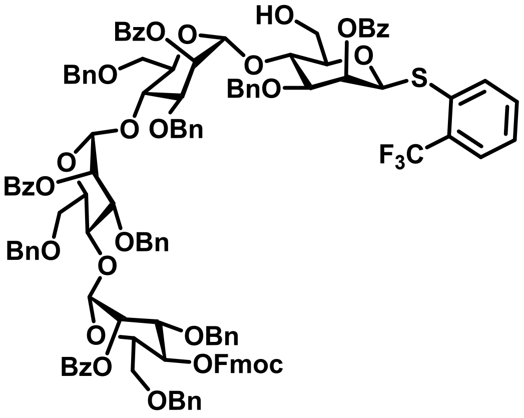

(Trifluoromethyl)phenyl 2-*O*-benzoyl-3,6-di-*O*-benzyl-4-O-(9-fluorenylmethoxycarbonyl)-*α*-D-mannopyranosyl-(1→4)-2-*O*-benzoyl-3,6-di-*O*-benzyl-*α*-D-mannopyranosyl-(1→4)-2-*O*-benzoyl-3,6-di-*O*-benzyl-*α*-D-mannopyranosyl-(1→4)-2-*O*-benzoyl-3-*O*-benzyl-1-thio-*β*-D-mannopyranoside (11)

Step	Building Block	Modules	Notes
AGA	BB **9**, 6 eq.	**A** – first coupling	-40°C (T1) 5 min<br>-20°C (T2) 35 min
		**B** – RV Wash	
		**C** – Acidic Wash	
		**D** – Capping	
	**3x**	**E** – Fmoc Deprotection	
		**C** – Acidic Wash	
	BB **10**, 4 eq.	**F** – Phosphate coupling	-30°C (T1) 10 min<br>0°C (T2) 20 min
**Post AGA**		**J** – Batch Photocleavage	16 h
**Purification**		**L** – NP - (10 – 100%)	R_t = 20.1 min

After a procedure including automated glycan assembly, photo-cleavage, purification and lyophilization **11** was obtained as a translucent resin (8.64 mg, 4.13 µmol, 20%).

Rt (NP – 10 - 100%) = 20.1 min.

^{1}H NMR (600 MHz, $CDCl_3$) *δ* 8.17 – 8.08 (m, 2H), 7.92 (d, *J* = 7.8 Hz, 4H), 7.89 (dd, *J* = 8.2, 1.3 Hz, 2H), 7.84 (d, *J* = 7.8 Hz, 1H), 7.76 (d, *J* = 7.6 Hz, 2H), 7.72 (d, *J* = 7.9 Hz, 1H), 7.61 – 7.50 (m, 4H), 7.50 – 7.42 (m, 6H), 7.41 – 7.27 (m, 15H), 7.25 – 7.00 (m, 30H), 6.10 (d, *J* = 3.1 Hz, 1H, H2'''), 5.75 (q, *J* = 2.5 Hz, 2H, H2'', H2'), 5.71 (t, *J* = 2.4 Hz, 1H, H2), 5.57 (d, *J* = 1.9 Hz, 1H, *α*-H1'), 5.52 (d, *J* = 2.0 Hz, 1H, *α*-H1''), 5.46 (d, *J* = 1.9 Hz, 1H, *α*-H1'''), 5.38 (t, *J* = 9.9 Hz, 1H, H4'''), 4.99 (s, 1H, *β*-H1), 4.89 (d, *J* = 10.6 Hz, 1H), 4.84 – 4.74 (m, 3H), 4.61 (dt, *J* = 22.8, 11.4 Hz, 3H), 4.54 – 4.44 (m, 5H), 4.40 – 4.32 (m, 3H), 4.30 – 4.23 (m, 3H), 4.19 (d, *J* = 9.4 Hz, 1H), 4.12 (td, *J* = 7.3, 2.1 Hz, 1H, CH1-Fmoc), 4.02 (td, *J* = 8.8, 3.0 Hz, 4H), 3.92 (ddd, *J* = 20.3, 9.4, 2.7 Hz, 3H), 3.87 – 3.79 (m, 3H), 3.76 – 3.70 (m, 1H), 3.63 (dd, *J* = 11.3, 3.5 Hz, 1H), 3.57 – 3.37 (m, 4H), 2.73 (t, *J* = 6.7 Hz, 1H, C6OH).

^{13}C NMR (101 MHz, $CDCl_3$) δ 165.6, 165.3, 165.3, 165.3, 154.6, 143.5, 143.5, 141.4, 138.7, 138.3, 138.2, 138.0, 137.1, 136.9, 136.6, 134.9, 133.5, 133.3, 133.2, 132.4, 131.5, 130.3, 130.1, 130.0, 129.9, 129.6, 129.6, 129.6, 129.4, 129.2, 128.9, 128.7, 128.6, 128.6, 128.6, 128.5, 128.4, 128.4, 128.4, 128.3, 128.3, 128.2, 128.1, 128.0, 127.9, 127.8, 127.8, 127.7, 127.6, 127.6, 127.5, 127.3, 127.3, 125.2, 120.2, 99.4 (*α*-C1'''), 99.2 (*α*-C1''), 99.0 (*α*-C1'), 85.6 (*β*-C1), 80.9, 79.5, 78.3, 77.7, 75.4, 73.7, 73.6, 73.5, 72.5, 72.4, 72.3, 71.6, 71.5, 71.4, 71.3, 71.2, 70.7, 70.0, 69.9, 69.2, 69.0, 68.4, 68.1, 68.0, 62.4, 46.9 (CH1-Fmoc).

HRMS (ESI): $C_{123}H_{113}F_3NaO_{26}S$ $[M+Na]^+$; calculated: 2117.7091, found: 2117.7507.

Optical rotation: $[\alpha]_D^{25}$ = -50.6° (c = 0.5, $CHCl_3$)

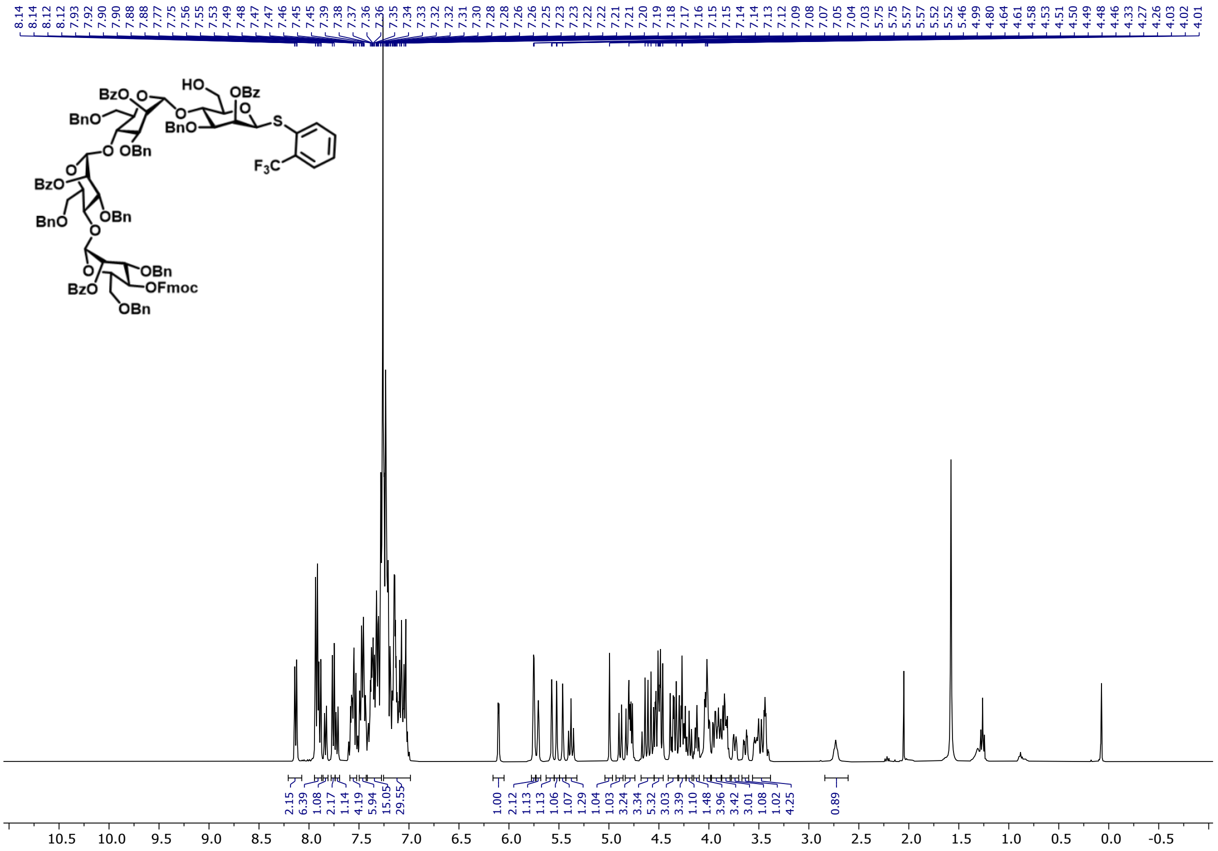
8.14 8.14 8.12 8.12 7.93 7.92 7.90 7.90 7.88 7.88 7.77 7.75 7.56 7.55 7.53 7.49 7.48 7.47 7.46 7.45 7.45 7.39 7.38 7.37 7.36 7.36 7.35 7.34 7.33 7.32 7.32 7.31 7.30 7.28 7.28 7.26 7.25 7.23 7.23 7.22 7.22 7.21 7.21 7.20 7.19 7.18 7.17 7.16 7.15 7.15 7.14 7.14 7.13 7.12 7.09 7.08 7.07 7.05 7.04 7.03 5.75 5.75 5.57 5.57 5.52 5.52 5.46 4.99 4.80 4.64 4.61 4.58 4.53 4.51 4.50 4.49 4.48 4.46 4.33 4.27 4.26 4.03 4.02 4.01
HO
OBz
BzO
BnO
S
F3C
OBn
OFmoc
2.15 6.39 1.08 2.17 1.14 4.19 5.94 15.05 29.55 1.00 2.12 1.13 1.13 1.06 1.07 1.29 1.04 1.03 3.24 3.34 5.32 3.03 3.39 1.10 1.48 3.96 3.42 3.01 1.08 1.02 4.25 0.89
10.5 10.0 9.5 9.0 8.5 8.0 7.5 7.0 6.5 6.0 5.5 5.0 4.5 4.0 3.5 3.0 2.5 2.0 1.5 1.0 0.5 0.0 -0.5

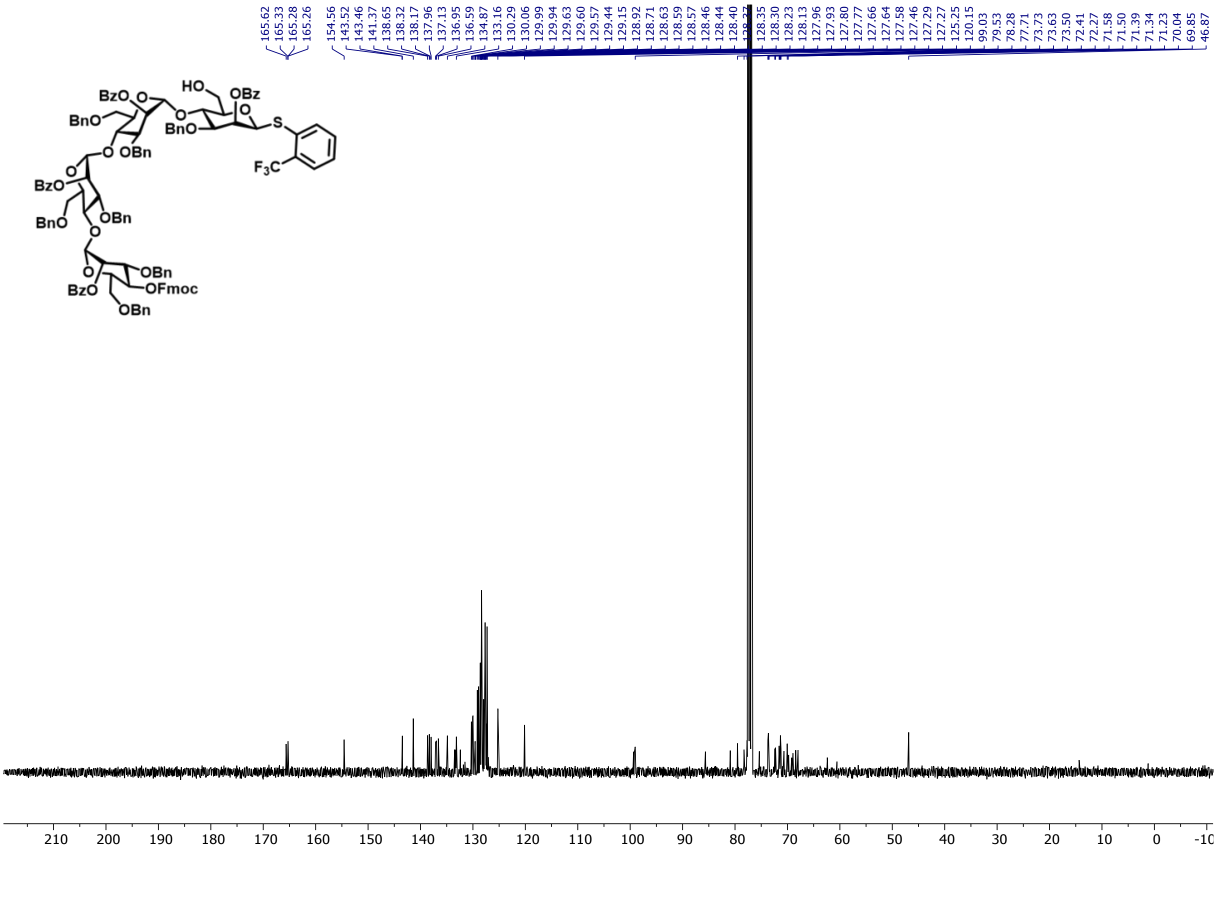
165.62 165.33 165.28 165.26 154.56 143.52 143.46 141.37 138.65 138.32 138.17 137.96 137.13 136.95 136.59 134.87 133.16 130.29 130.06 129.99 129.94 129.63 129.60 129.57 129.44 129.15 128.92 128.71 128.63 128.59 128.57 128.46 128.44 128.40 128.35 128.30 128.23 128.13 127.96 127.93 127.80 127.77 127.66 127.64 127.58 127.46 127.29 127.27 125.25 120.15 99.03 79.53 78.28 77.71 73.73 73.63 73.50 72.41 72.27 71.58 71.50 71.39 71.34 71.23 70.04 69.85 46.87
HO
OBz
BzO
BnO
S
F3C
OBn
OFmoc
210 200 190 180 170 160 150 140 130 120 110 100 90 80 70 60 50 40 30 20 10 0 -10

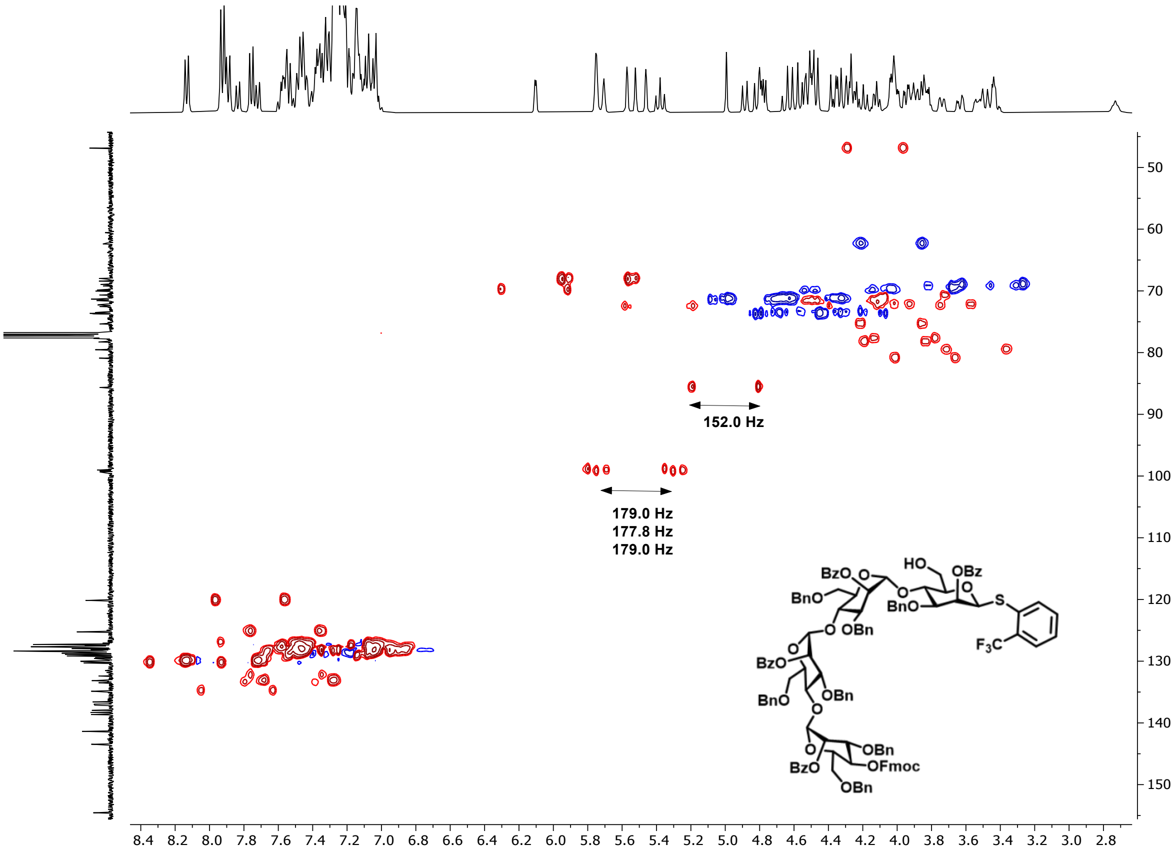

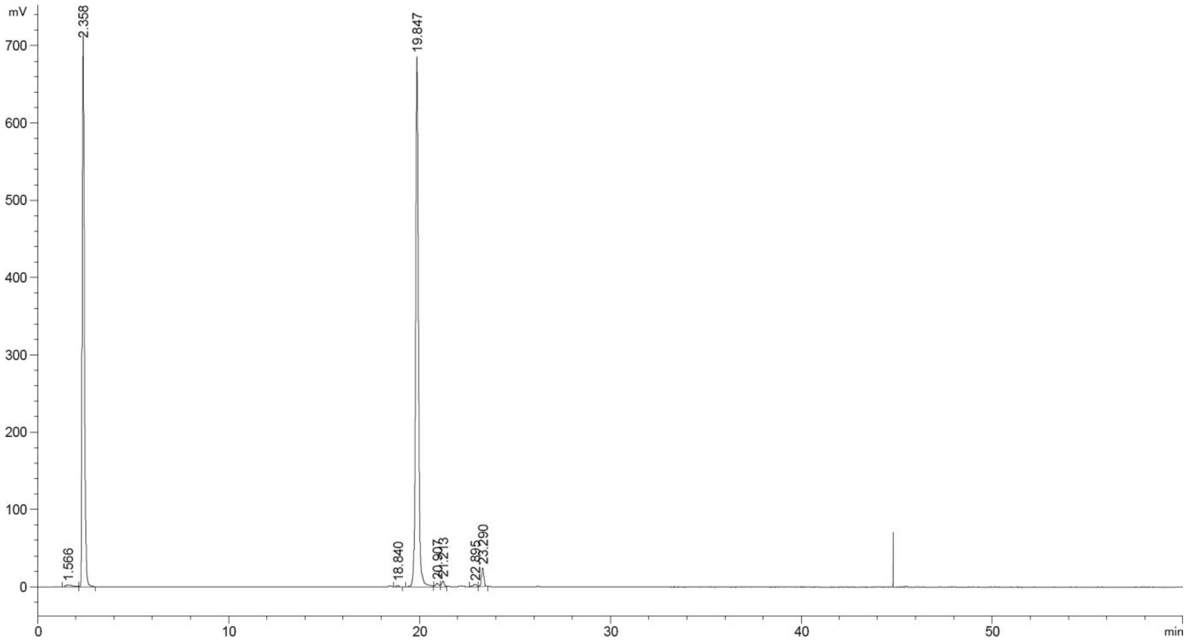

Figure 5: NP-HPLC trace of crude **11** (10-100%)

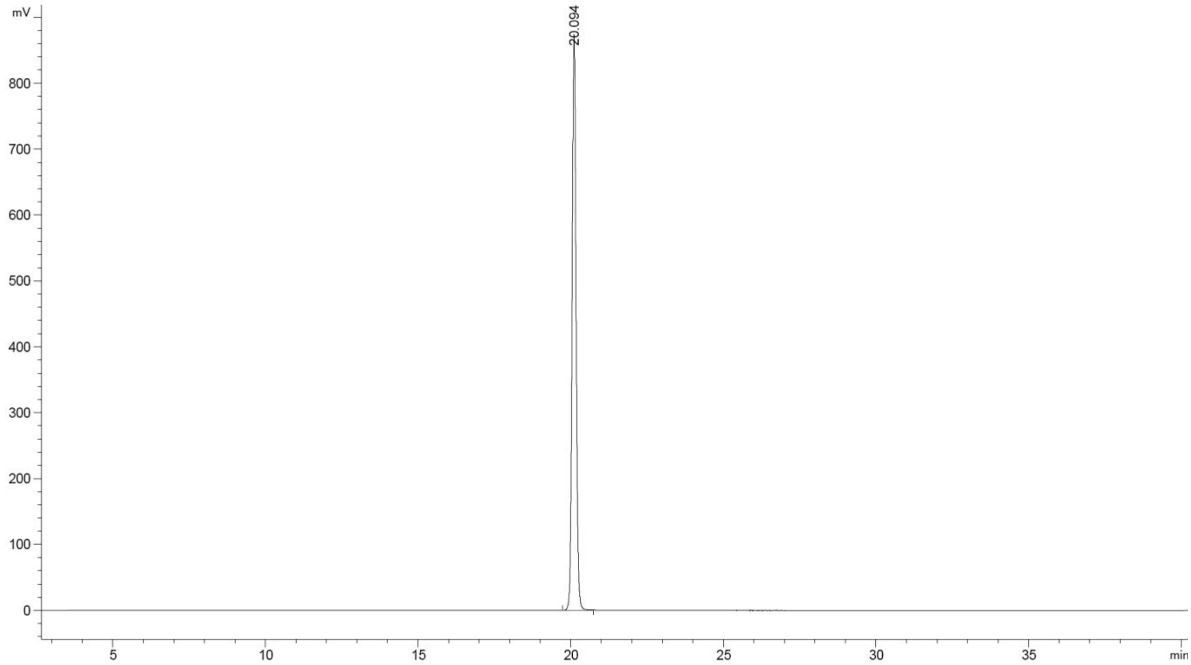

Figure 6: NP-HPLC trace of pure **11** (10-100%)

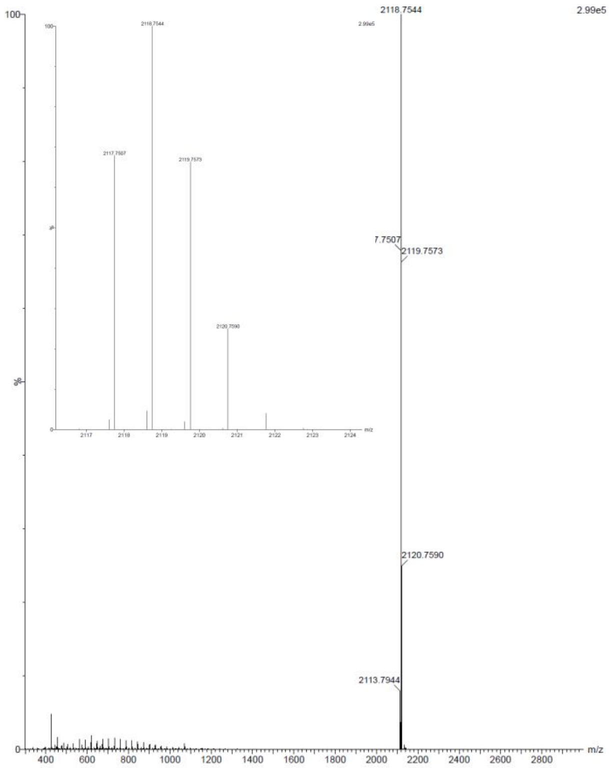

Figure 7: Q-TOF MS-spectrum of **11**.

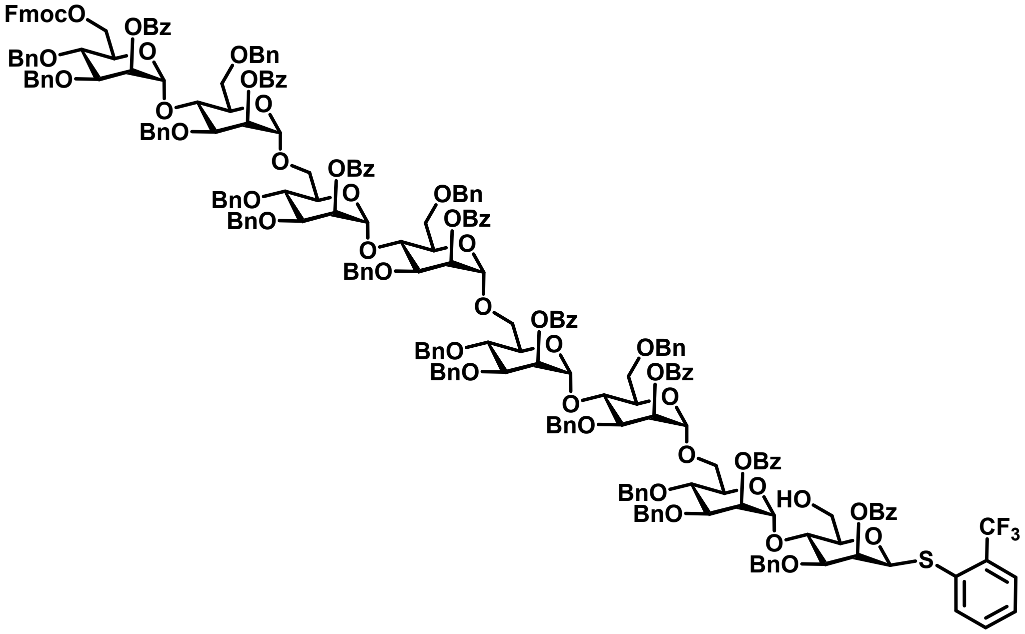

(Trifluoromethyl)phenyl 2-*O*-benzoyl-3,6-di-*O*-benzyl-4-O-(9-fluorenylmethoxycarbonyl)-*α*-D-mannopyranosyl-tri-[(1→4)-2-*O*-benzoyl-3,6-di-*O*-benzyl-*α*-D-mannopyranosyl-(1→6)-2-*O*-benzoyl-3,4-di-*O*-benzyl-*α*-D-mannopyranosyl]-(1→4)-2-*O*-benzoyl-3-*O*-benzyl-1-thio-*β*-D-mannopyranoside (13)

Step	Building Block	Modules	Notes
AGA	BB **9**, 6 eq.	**A** – first coupling	-40°C (T1) 5 min<br>-20°C (T2) 35 min
		**B** – RV Wash<br>**C** – Acidic Wash<br>**D** – Capping	
	alternating<br>**4x**<br>BB **14**, 4 eq.	**E** – Fmoc Deprotection<br>**C** – Acidic Wash<br>**F** – Phosphate coupling	-30°C (T1) 20 min<br>0°C (T2) 20 min
	**3x**<br>BB **10**, 4 eq.	**E** – Fmoc Deprotection<br>**C** – Acidic Wash<br>**F** – Phosphate coupling	-30°C (T1) 10 min<br>0°C (T2) 20 min
**Post AGA**		**J** – Batch Photocleavage	16 h

Purification	L – NP - (10-100)	R_t = 23.8 min

After a procedure including automated glycan assembly, photo-cleavage, purification and lyophilization, **13** was obtained as a translucent resin (23.1 mg, 5.95, μmol 30%).

Rt (NP - 10-100) = 23.8 min.

^{1}H NMR (400 MHz, $CDCl_3$) *δ* 8.15 – 7.97 (m, 17H), 7.77 (dd, *J* = 15.0, 7.7 Hz, 3H), 7.68 (dd, *J* = 7.9, 1.5 Hz, 1H), 7.59 (d, *J* = 7.0 Hz, 2H), 7.51 (dtdd, *J* = 15.9, 8.1, 5.9, 2.1 Hz, 15H), 7.46 – 7.38 (m, 5H), 7.37 – 7.27 (m, 32H), 7.25 – 7.06 (m, 43H), 6.95 (ddd, *J* = 10.7, 8.7, 7.1 Hz, 6H), 6.88 – 6.74 (m, 3H), 6.10 – 6.04 (m, 1H, H2), 5.84 – 5.75 (m, 5H, 5x H2), 5.72 (t, *J* = 2.4 Hz, 1H, H2), 5.65 (d, *J* = 2.4 Hz, 1H, H2), 5.55 (d, *J* = 1.8 Hz, 1H, *α*-1→4-H1), 5.51 – 5.43 (m, 3H, 3x *α*-1→4-H1), 5.13 (d, *J* = 1.8 Hz, 1H, *α*-1→6-H1), 4.95 (dd, *J* = 8.9, 1.4 Hz, 2H), 4.93 – 4.87 (m, 2H), 4.86 – 4.72 (m, 7H), 4.72 – 4.61 (m, 6H), 4.59 – 4.45 (m, 8H), 4.43 – 4.35 (m, 2H), 4.35 – 4.26 (m, 10H), 4.25 – 4.17 (m, 5H), 4.12 (dd, *J* = 9.4, 3.0 Hz, 2H), 3.97 (dddd, *J* = 24.2, 10.8, 8.2, 5.4 Hz, 10H), 3.83 (dddd, *J* = 16.7, 13.8, 8.4, 3.8 Hz, 10H), 3.76 – 3.70 (m, 3H), 3.69 – 3.61 (m, 5H), 3.54 (dd, *J* = 10.1, 5.1 Hz, 1H), 3.48 (ddd, *J* = 9.7, 4.6, 2.3 Hz, 1H), 3.38 (dd, *J* = 19.4, 11.1 Hz, 2H), 2.36 (t, *J* = 6.8 Hz, 1H, C6OH).

^{13}C NMR (101 MHz, $CDCl_3$) δ 165.6, 165.5, 165.4, 165.4, 165.2, 155.2, 143.6, 143.4, 141.3, 138.9, 138.9, 138.5, 138.5, 138.4, 138.4, 138.2, 138.0, 138.0, 138.0, 137.9, 137.0, 136.6, 136.6, 134.7, 133.5, 133.3, 133.1, 132.6, 132.4, 131.7, 131.4, 131.0, 130.2, 130.1, 130.1, 130.0, 130.0, 129.9, 129.7, 129.7, 129.4, 129.0, 128.9, 128.9, 128.9, 128.7, 128.7, 128.6, 128.6, 128.6, 128.6, 128.5, 128.5, 128.5, 128.5, 128.4, 128.3, 128.3, 128.2, 128.1, 128.1, 128.0, 127.9, 127.9, 127.8, 127.7, 127.6, 127.6, 127.6, 127.4, 127.4, 127.3, 127.3, 127.0, 125.4, 125.3, 120.1, 100.1 (*α*-1→4-C1), 99.9 (*α*-1→4-C1), 99.8 (*α*-1→4-C1), 99.6 (*α*-1→4-C1), 98.3 (3x *α*-1→6-C1), 85.5 (*β*-C1-thioacetal), 80.8, 79.4, 79.0, 78.7, 78.5, 75.4, 75.3, 75.1, 75.1, 73.9, 73.7, 73.6, 73.3, 72.5, 72.3, 72.2, 72.1, 71.9, 71.7, 71.6, 71.5, 70.8, 70.7, 70.6, 70.1, 69.8, 69.3, 69.1, 68.8, 68.5, 68.3, 67.8, 67.5, 67.0, 66.6, 66.1, 62.3, 46.8 (CH-Fmoc).

HRMS (ESI): $C_{231}H_{217}F_3NaO_{50}S$ $[M+Na]^+$; calculated: 3902.4008, found: 3902.3899 (main peak belongs to $[M+2Na]^{2+}$).

Optical rotation: $[\alpha]_D^{25}$ = -20.8° (c = 0.5, $CHCl_3$)

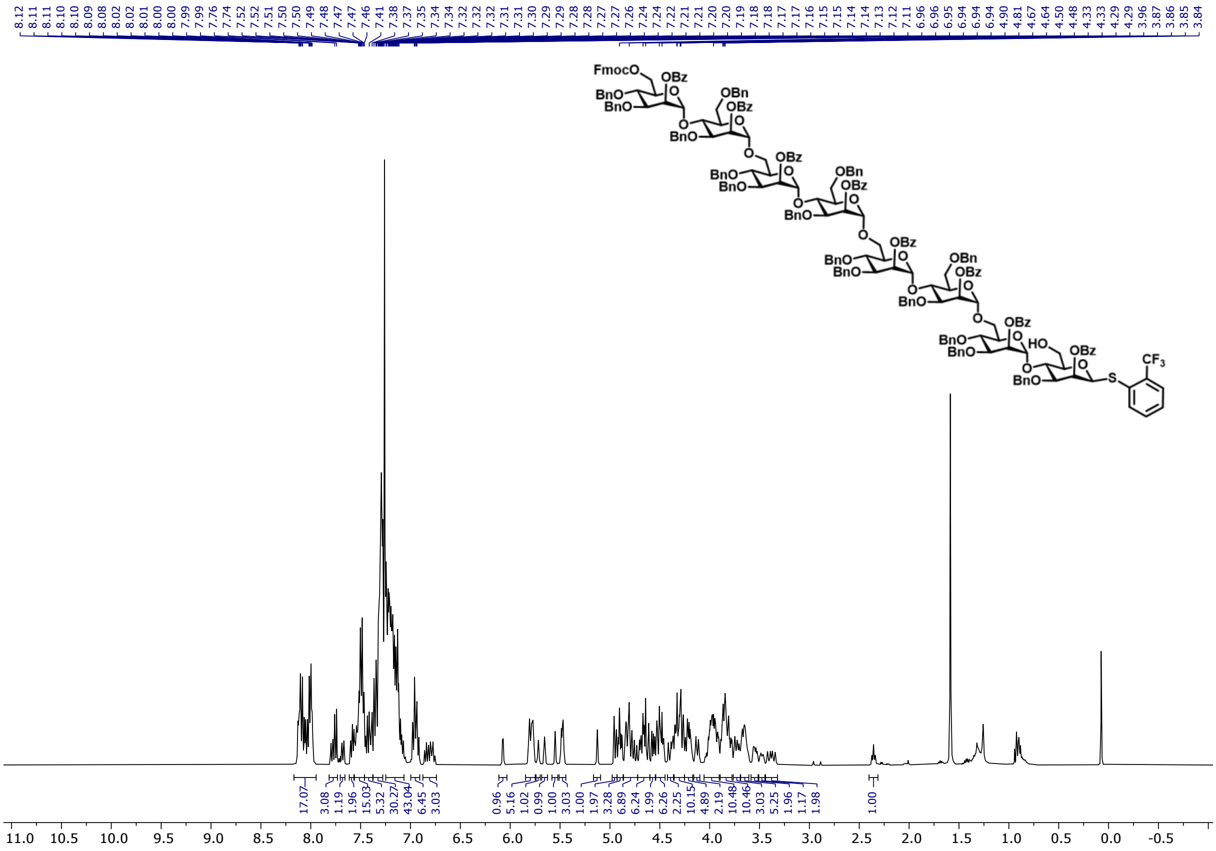
8.12 8.11 8.11 8.10 8.10 8.09 8.08 8.02 8.02 8.01 8.00 8.00 7.99 7.99 7.76 7.74 7.52 7.52 7.51 7.50 7.49 7.48 7.47 7.47 7.46 7.41 7.38 7.37 7.35 7.34 7.34 7.32 7.32 7.32 7.31 7.31 7.30 7.29 7.29 7.28 7.28 7.27 7.26 7.24 7.24 7.22 7.21 7.21 7.20 7.20 7.19 7.18 7.18 7.17 7.17 7.16 7.15 7.15 7.14 7.14 7.13 7.12 7.11 6.96 6.96 6.95 6.94 6.94 6.94 4.90 4.81 4.67 4.64 4.50 4.48 4.33 4.33 4.29 4.29 3.96 3.87 3.86 3.85 3.84
FmocO
OBz
BnO
OBn
CF3
S
HO
17.07 3.08 1.19 1.96 15.03 5.32 30.27 43.04 6.45 3.03 0.96 5.16 1.02 0.99 1.00 3.03 1.00 1.97 3.28 6.89 6.24 1.99 6.26 2.25 10.15 4.89 2.19 10.48 10.46 3.03 5.25 1.96 1.17 1.98 1.00
11.0 10.5 10.0 9.5 9.0 8.5 8.0 7.5 7.0 6.5 6.0 5.5 5.0 4.5 4.0 3.5 3.0 2.5 2.0 1.5 1.0 0.5 0.0 -0.5

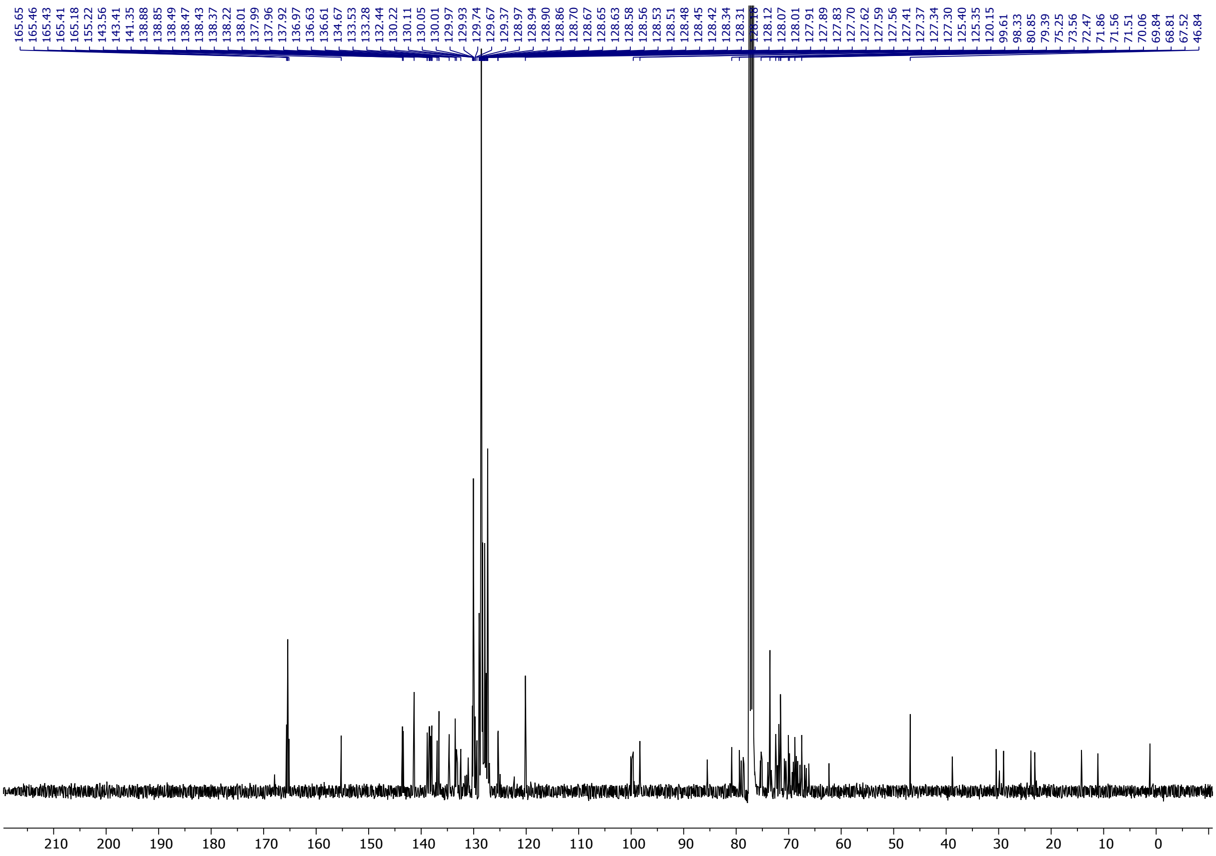
165.65 165.46 165.43 165.41 165.18 155.22 143.56 143.41 141.35 138.88 138.85 138.49 138.47 138.43 138.37 138.22 138.01 137.99 137.96 137.92 136.97 136.63 136.61 134.67 133.53 133.28 132.44 130.22 130.11 130.05 130.01 129.97 129.93 129.74 129.67 129.67 129.37 128.97 128.94 128.90 128.86 128.70 128.67 128.65 128.63 128.58 128.56 128.53 128.51 128.48 128.45 128.42 128.34 128.31 128.12 128.07 128.01 127.91 127.89 127.83 127.70 127.62 127.59 127.56 127.41 127.37 127.34 127.30 125.40 125.35 120.15 99.61 98.33 80.85 79.39 75.25 73.56 72.47 71.86 71.56 71.51 70.06 69.84 68.81 67.52 46.84
210 200 190 180 170 160 150 140 130 120 110 100 90 80 70 60 50 40 30 20 10 0

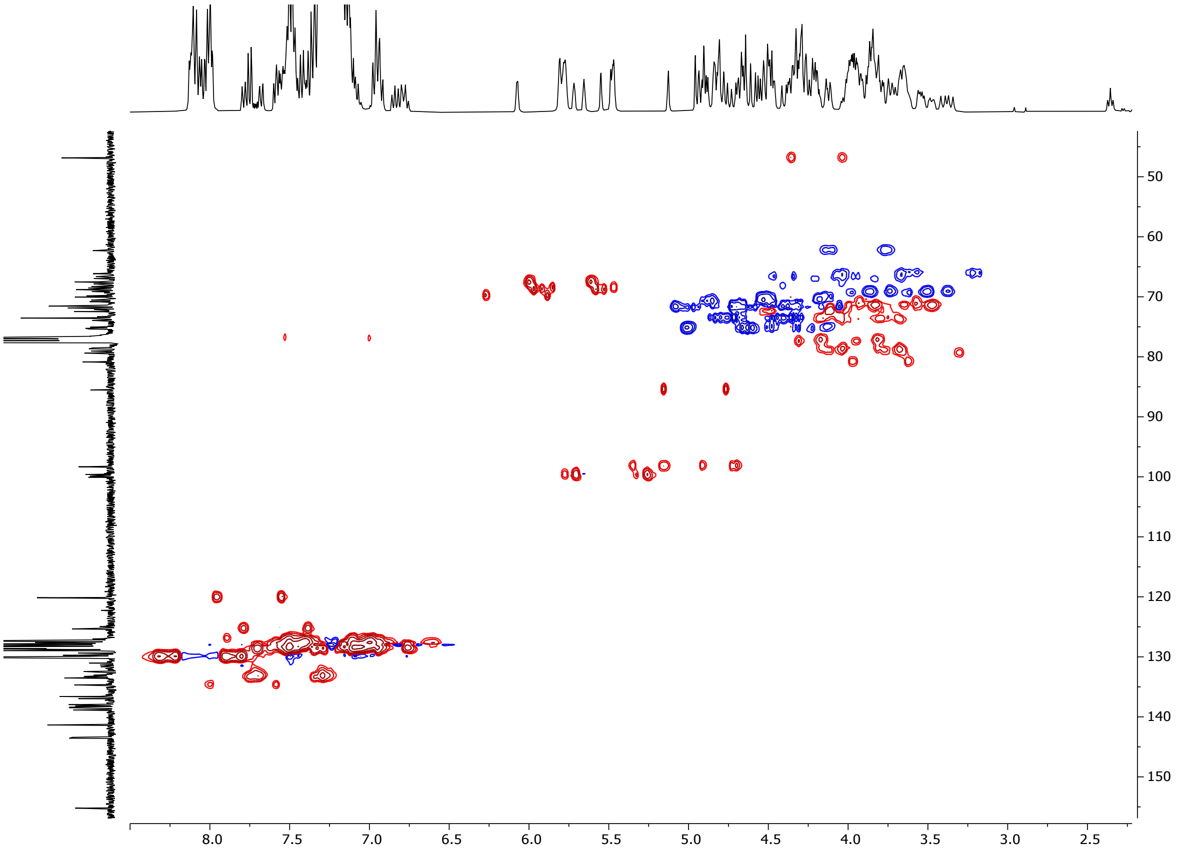
50
60
70
80
90
100
110
120
130
140
150
8.0
7.5
7.0
6.5
6.0
5.5
5.0
4.5
4.0
3.5
3.0
2.5

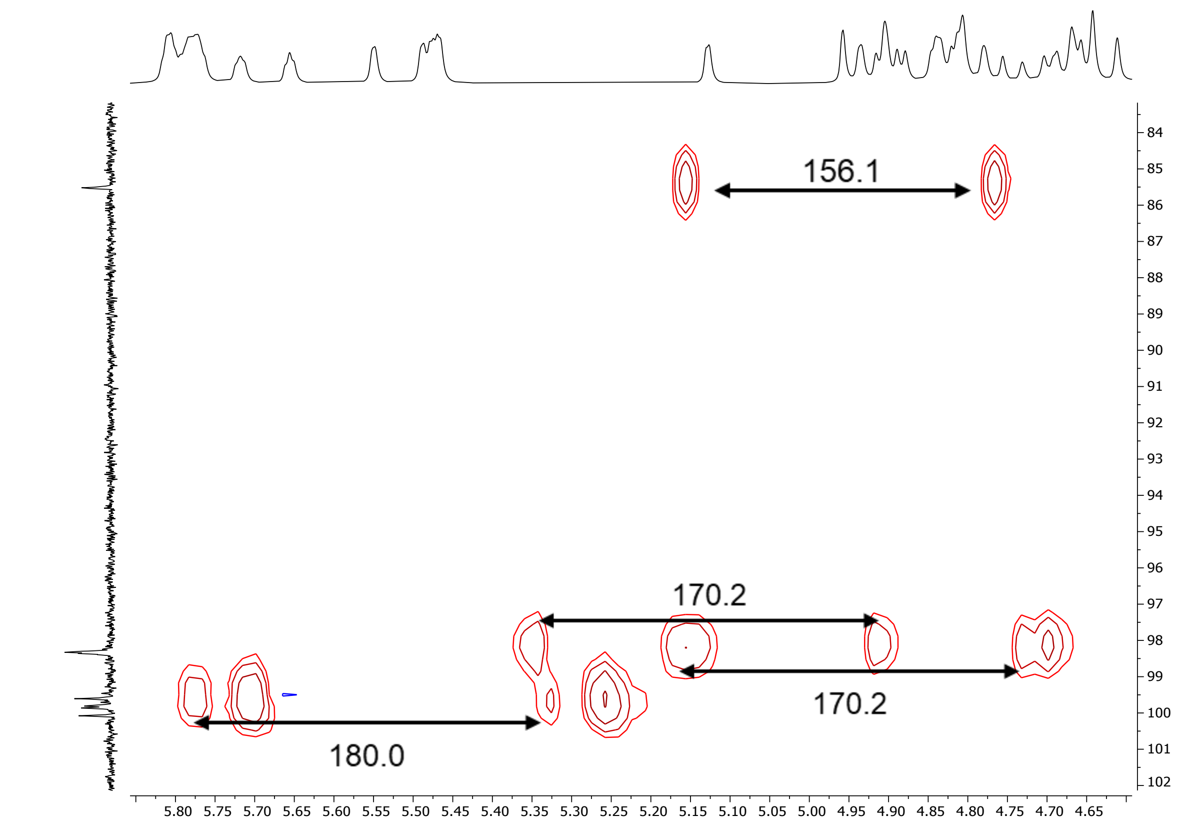
156.1
170.2
170.2
180.0
84
85
86
87
88
89
90
91
92
93
94
95
96
97
98
99
100
101
102
5.80 5.75 5.70 5.65 5.60 5.55 5.50 5.45 5.40 5.35 5.30 5.25 5.20 5.15 5.10 5.05 5.00 4.95 4.90 4.85 4.80 4.75 4.70 4.65

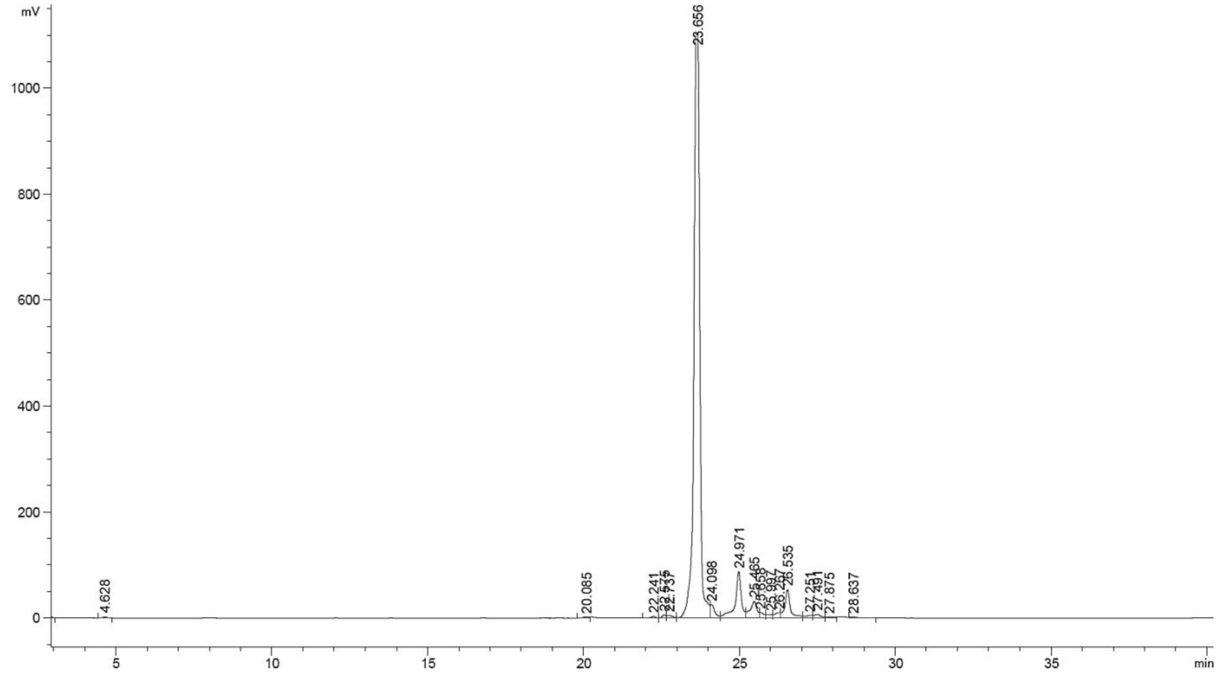

Figure 8: NP-HPLC trace of crude **13** (10-100%).

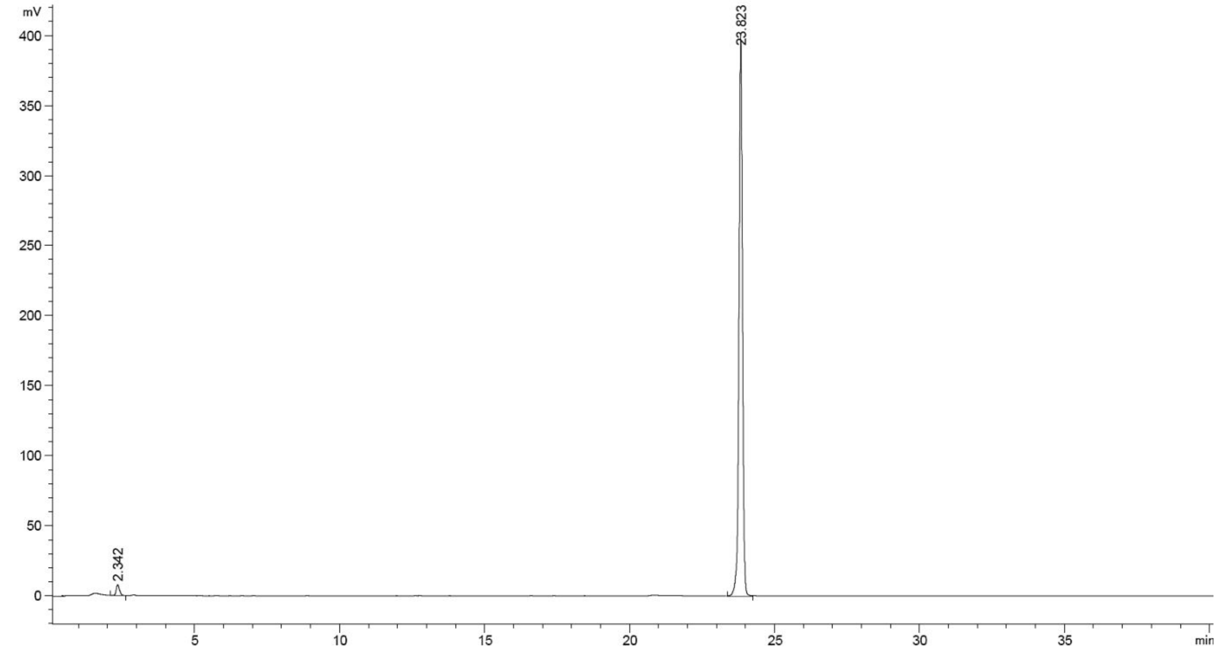

Figure 9: NP-HPLC trace of pure **13** (10-100%).

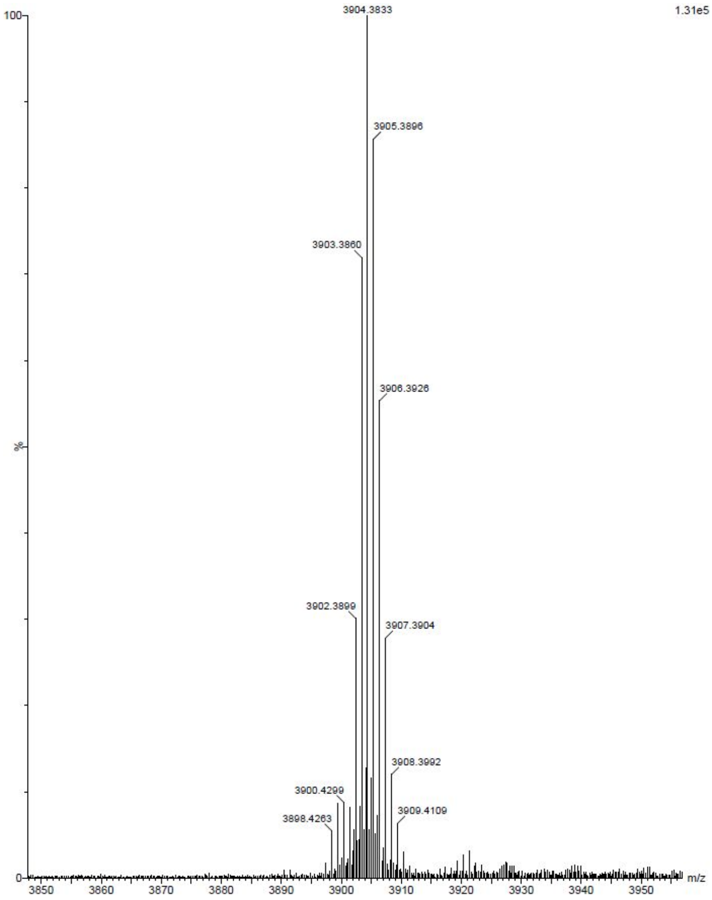

Figure 10: Q-TOF MS-spectrum of **13**.

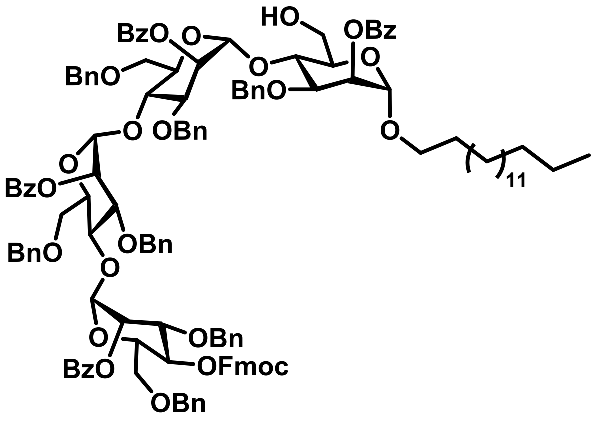

Hexadecyl 2-O-benzoyl-3,6-di-O-benzyl-4-O-(9-fluorenylmethoxycarbonyl)-*α*-D-mannopyranosyl-(1→4)-2-O-benzoyl-3,6-di-O-benzyl-*α*-D-mannopyranosyl-(1→4)-2-O-benzoyl-3,6-di-O-benzyl-*α*-D-mannopyranosyl-(1→4)-2-O-benzoyl-3-O-benzyl-*α*-D-mannopyranoside (15)

Step	Building Block	Modules	Notes
AGA	BB **9**, 6 eq.	**A** – first coupling	-40°C (T1) 5 min<br>-20°C (T2) 35 min
		**B** – RV Wash	
		**C** – Acidic Wash	
		**D** – Capping	
	**3x**	**E** – Fmoc Deprotection	
		**C** – Acidic Wash	
	BB **10** 3.5 eq.	**F** – Phosphate coupling	-30°C (T1) 10 min<br>0°C (T2) 20 min
		**E** – Fmoc Deprotection	
		**D** – Capping	
		**C** – Acidic Wash	
	Hexadecanol, 18 eq.	**G** – Thioglycoside Coupling	20°C (T1) 10 min<br>30°C (T2) 35 min
Post AGA		**J** – Batch Photocleavage	16 h
Purification		**L** – NP - (10-100)	R_t = 17.6 min

After a procedure including automated glycan assembly, photo-cleavage, purification and lyophilization, **15** was obtained as a translucent resin (4.3 mg, 1.99 µmol, 10%).

R_t (NP - 10-100) = 17.6 min.

^{1}H NMR (600 MHz, $CDCl_3$) *δ* 8.06 (dd, *J* = 8.3, 1.4 Hz, 2H), 7.97 – 7.86 (m, 6H), 7.75 (dd, *J* = 7.6, 1.0 Hz, 2H), 7.58 – 7.34 (m, 13H), 7.30 (ddt, *J* = 9.4, 6.4, 1.9 Hz, 12H), 7.25 – 7.18 (m, 15H), 7.16 – 7.01 (m, 13H), 5.79 (t, *J* = 2.5 Hz, 1H, H2'''), 5.75 (dd, *J* = 3.1, 2.0 Hz, 1H, H2''), 5.71 (t, *J* = 2.4 Hz, 1H, H2'), 5.60 (dd, *J* = 3.2, 1.9 Hz, 1H, H2), 5.57 (t, *J* = 2.4 Hz, 2H, *α*-C1'' and *α*-C1'''), 5.47 (d, *J* = 1.9 Hz, 1H, *α*-C1'), 5.37 (t, *J* = 10.0 Hz, 1H, H4'''), 4.97 (d, *J* = 1.8 Hz, 1H, *α*-H1), 4.84 – 4.77 (m, 3H), 4.72 (d, *J* = 11.0 Hz, 1H), 4.67 – 4.56 (m, 3H), 4.52 (ddd, *J* = 11.9, 9.5, 2.8 Hz, 4H), 4.46 (s, 1H), 4.39 (d, *J* = 11.9 Hz, 1H), 4.37 – 4.23 (m, 5H), 4.22 – 4.09 (m, 3H), 4.05 – 3.93 (m, 6H), 3.90 (dd, *J* = 9.8, 3.0 Hz, 2H), 3.86 – 3.70 (m, 4H), 3.65 (dd, *J* = 11.2, 3.7 Hz, 1H), 3.54 – 3.40 (m, 4H), 2.72 (t, *J* = 6.6 Hz, 1H, C6OH), 1.65 (p, *J* = 6.6 Hz, 2H), 1.45 – 1.27 (m, 26H), 0.90 – 0.85 (m, 3H, CH_3-hexadecanyl).

^{13}C NMR (151 MHz, $CDCl_3$) δ 166.0, 165.3, 154.6, 143.6, 141.4, 138.7, 138.4, 138.2, 138.0, 137.3, 137.1, 133.4, 133.1, 130.1, 130.0, 130.0, 128.9, 128.8, 128.7, 128.6, 128.5, 128.4, 128.4, 128.3, 128.3, 128.0, 127.9, 127.7, 127.7, 127.5, 127.4, 127.3, 125.3, 99.5 (*α*-C1'''), 99.3 (*α*-C1''), 99.1 (*α*-C1'), 97.9 (*α*-C1), 77.9, 77.7, 73.8, 73.7, 73.6, 72.6, 72.3, 71.5, 71.4, 70.1, 68.7, 68.5, 68.2, 46.9 (CH-Fmoc), 32.1, 29.9, 29.9, 29.8, 29.7, 29.6, 29.5, 26.3, 22.8, 14.3 (CH_3-hexadecanyl).

HRMS (ESI): $C_{132}H_{142}NaO_{27}$ $[M+Na]^+$; calculated: 2181.9636, found: 2181.9878.

Optical rotation: $[\alpha]_D^{25}$ = -16.4° (c = 0.5, $CHCl_3$)

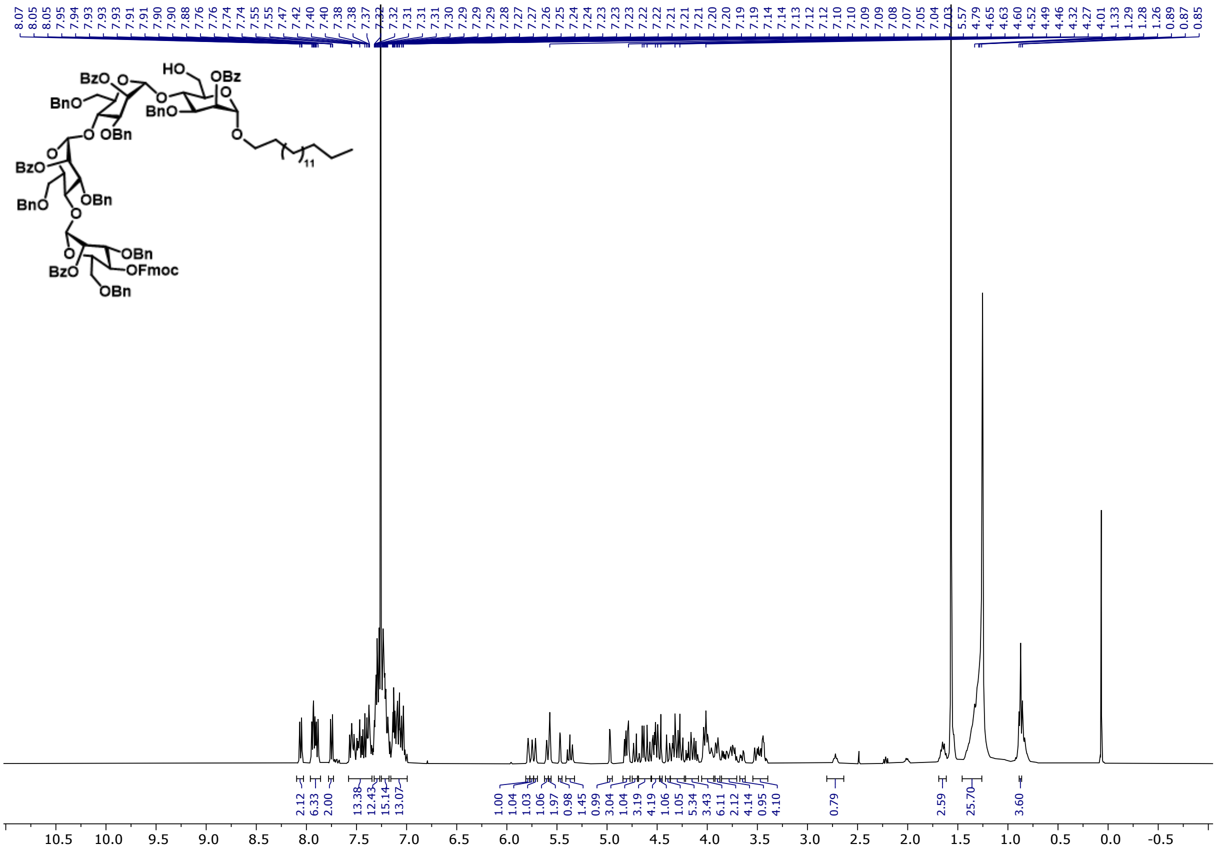
8.07 8.05 8.05 7.95 7.94 7.93 7.93 7.93 7.91 7.91 7.90 7.88 7.76 7.76 7.74 7.74 7.55 7.55 7.47 7.42 7.40 7.40 7.38 7.38 7.37 7.32 7.32 7.31 7.31 7.31 7.30 7.29 7.29 7.29 7.28 7.27 7.27 7.26 7.25 7.24 7.24 7.23 7.23 7.22 7.22 7.21 7.21 7.21 7.20 7.19 7.19 7.14 7.13 7.12 7.12 7.10 7.10 7.09 7.09 7.08 7.07 7.05 7.04 7.03 5.57 4.79 4.65 4.63 4.60 4.52 4.49 4.46 4.32 4.27 4.01 1.33 1.29 1.28 1.26 0.89 0.87 0.85
BzO
BnO
OBn
HO
OBz
BnO
11
BzO
BnO
OBn
OBn
OFmoc
BzO
OBn
2.12 6.33 2.00 13.38 12.43 15.14 13.07 1.00 1.04 1.03 1.06 1.97 0.98 1.45 0.99 3.04 1.04 3.19 4.19 1.06 1.05 5.34 3.43 6.11 2.12 4.14 0.95 4.10 0.79 2.59 25.70 3.60
10.5 10.0 9.5 9.0 8.5 8.0 7.5 7.0 6.5 6.0 5.5 5.0 4.5 4.0 3.5 3.0 2.5 2.0 1.5 1.0 0.5 0.0 -0.5

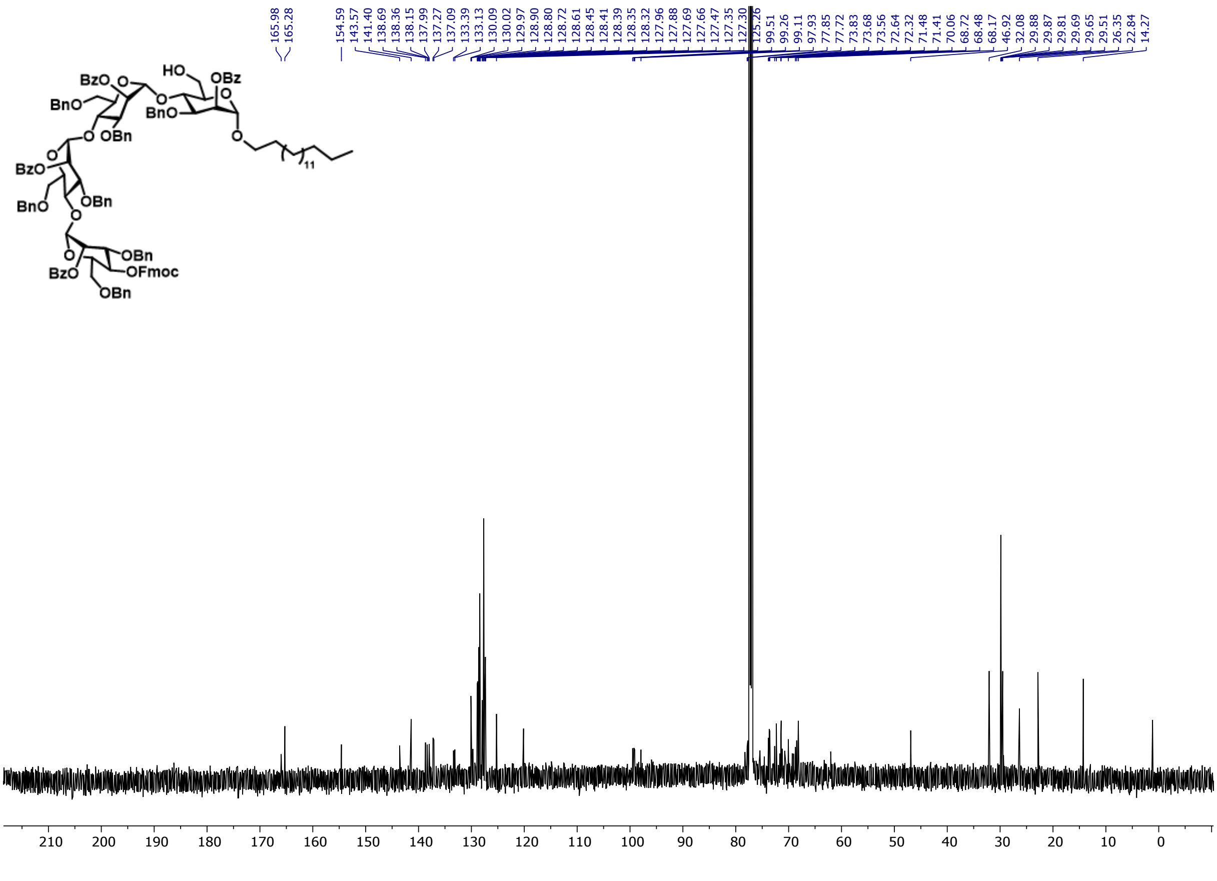
165.98 165.28 154.59 143.57 141.40 138.69 138.36 138.15 137.99 137.27 137.09 133.39 133.13 130.09 130.02 129.97 128.90 128.80 128.72 128.61 128.45 128.41 128.39 128.35 128.32 127.96 127.88 127.69 127.66 127.47 127.35 127.30 125.26 99.51 99.26 99.11 97.93 77.85 77.72 73.83 73.68 73.56 72.64 72.32 71.48 71.41 70.06 68.72 68.48 68.17 46.92 32.08 29.88 29.87 29.81 29.69 29.65 29.51 26.35 22.84 14.27
BzO
BnO
OBn
HO
OBz
BnO
11
BzO
BnO
OBn
OBn
OFmoc
BzO
OBn
210 200 190 180 170 160 150 140 130 120 110 100 90 80 70 60 50 40 30 20 10 0

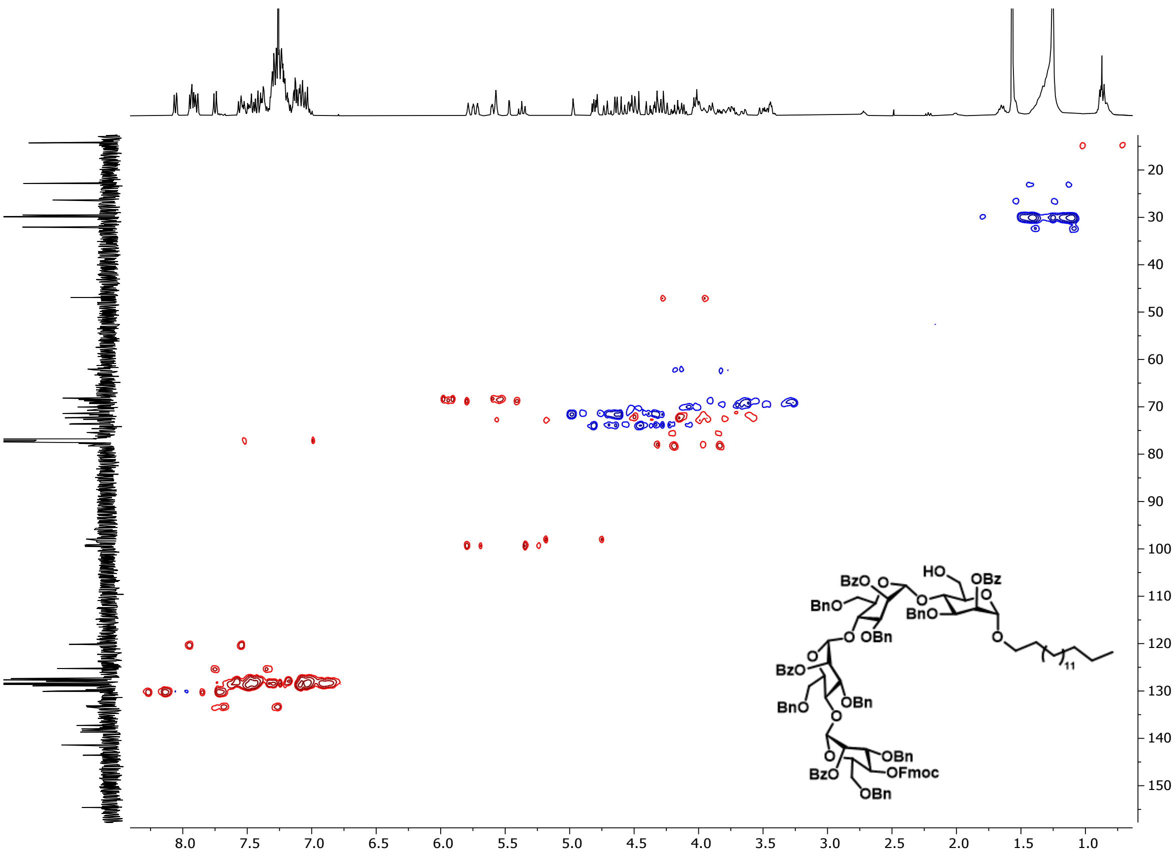
BzO
HO
OBz
BnO
BnO
OBn
BzO
BnO
OBn
OBn
OFmoc
BzO
OBn
11

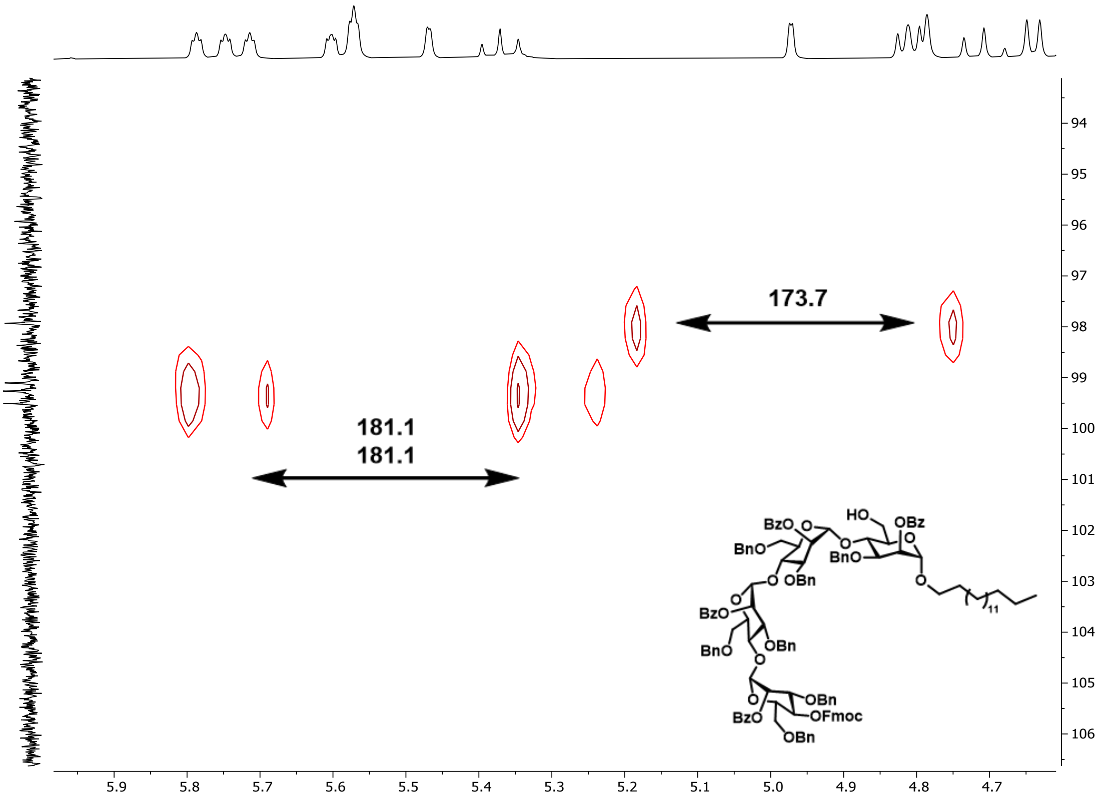
173.7
181.1
181.1
BzO
HO
OBz
BnO
BnO
OBn
BzO
BnO
OBn
OBn
OFmoc
BzO
OBn
11

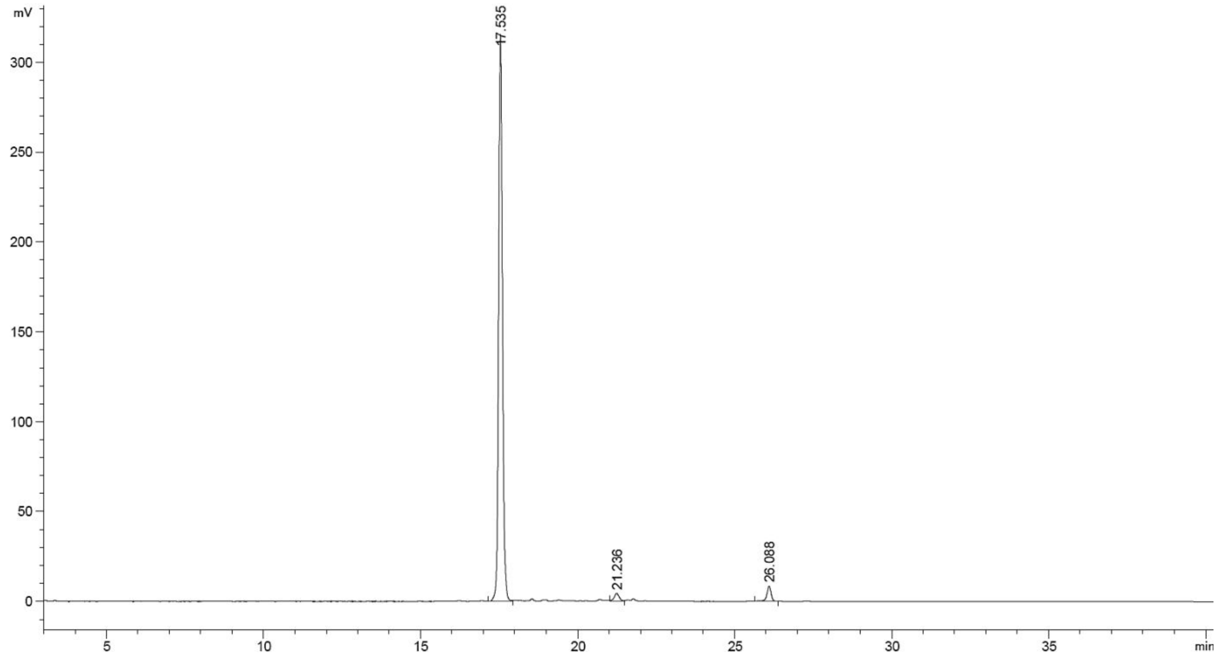

Figure 11: NP-HPLC trace of crude **15** (10-100%).

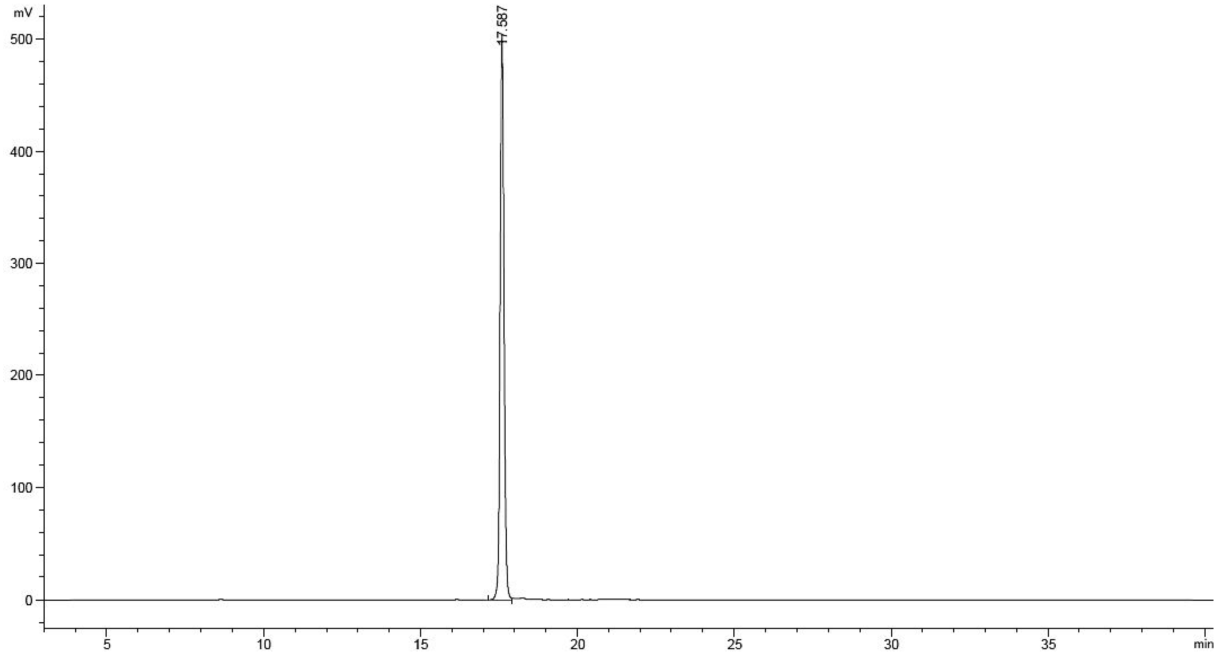

Figure 12: NP-HPLC trace of pure **15** (10-100%).

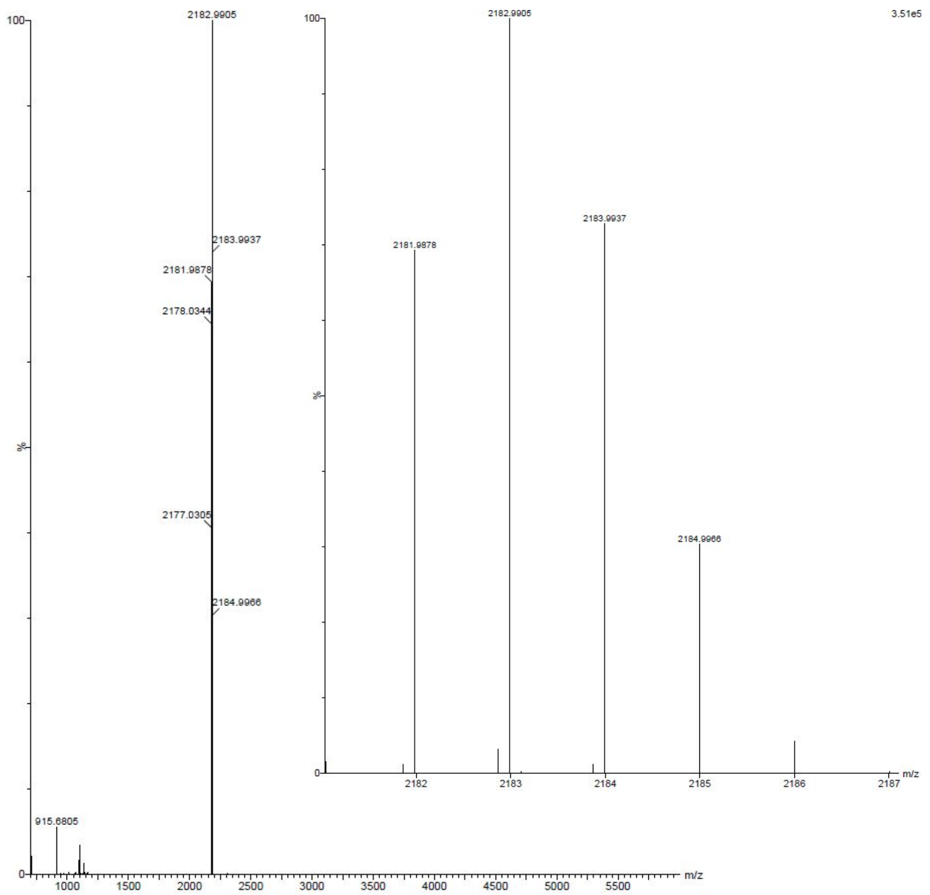

Figure 13: Q-TOF MS-spectrum of **15**.

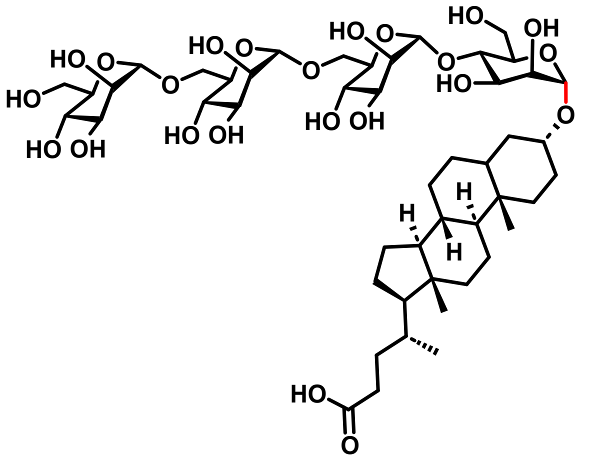

3-Tri-(1→6)-*α*-D-mannopyranosyl-(1→4)-*α*-D-mannopyranosyl-lithocholic acid (16)

Step	Building Block	Modules	Notes
AGA	BB **9**, 6 eq.	**A** – first coupling	-40°C (T1) 5 min<br>-20°C (T2) 35 min
		**B** – RV Wash<br>**C** – Acidic Wash<br>**D** – Capping	
	**3x**<br>BB **14**, 4 eq.	**E** – Fmoc Deprotection<br>**C** – Acidic Wash<br>**F** – Phosphate coupling	-30°C (T1) 20 min<br>0 °C (T2) 20 min
	BB **17**, 8 eq.	**E** – Fmoc Deprotection<br>**C** – Acidic Wash<br>**G** – Thioglycoside Coupling 2	15°C (T1) 10 min<br>35 °C (T2) 35 min
Post AGA		**I** – Methanolysis	16 h
		**J** – Batch Photocleavage	16 h
		**K** – Hydrogenolysis	16 h
Purification		**L** – C5-RP-(20-80% in 40 min)	R_t = 26.9 min

After a procedure including automated glycan assembly, hydrolysis, photo-cleavage, hydrogenolysis, purification and lyophilization, **16** was obtained as a white powder (3.9 mg, 3.72 μmol, 19%). Yield before deprotection was (14.8 mg, 35%).

R_t (C_{18} - 10 – 100%) = 25.7 min.

^{1}H NMR (400 MHz, D_2O/MeCN 1:1) *δ* 5.69 (d, *J* = 1.7 Hz, 1H, *α*-1→4-H1'), 5.40 (d, *J* = 1.6 Hz, 1H, *α*-H1), 5.33 (d, *J* = 1.7 Hz, 1H, *α*-H1''), 5.30 (d, *J* = 1.6 Hz, 1H, *α*-H1'''), 4.47 – 4.42 (m, 1H), 4.40 – 4.32 (m, 4H), 4.31 – 4.18 (m, 12H), 4.18 – 4.07 (m, 8H), 2.83 – 2.72 (m, 1H), 2.69 – 2.57 (m, 1H), 2.48 – 2.43 (m, 1H), 2.31 (dd, *J* = 23.5, 12.7 Hz, 2H), 2.23 – 1.99 (m, 4H), 1.96 – 1.82 (m, 7H), 1.82 – 1.67 (m, 7H), 1.65 – 1.49 (m, 4H), 1.40 (d, *J* = 6.7 Hz, 6H, 2x CH_3), 1.15 (s, 3H, CH_3).

^{13}C NMR (151 MHz, D_2O/MeCN 1:1) δ 177.0 (carboxylic acid), 102.5 (C1'), 100.8 (C1'''), 100.5 (C1''), 99.1 (C1), 78.4, 75.1, 74.0, 73.3, 72.7, 72.6, 72.5, 72.2, 72.0, 71.9, 71.7, 71.4, 71.3, 68.2, 68.0, 67.4, 67.0, 62.3, 57.5, 57.1, 43.7, 43.0, 41.6, 41.2, 37.0, 36.4, 36.2, 35.6, 33.4, 29.2, 29.0, 28.2, 27.4, 25.1, 24.0 (CH_3), 21.8, 19.0 (CH_3), 12.7 (CH_3).

HRMS (ESI): $C_{48}H_{80}NaO_{23}$ $[M+Na]^+$; calculated: 1047.4988, found: 1047.5059.

Optical rotation: $[\alpha]_D^{25}$ = +243.0° (c = 0.1, H_2O/MeCN 1:1)

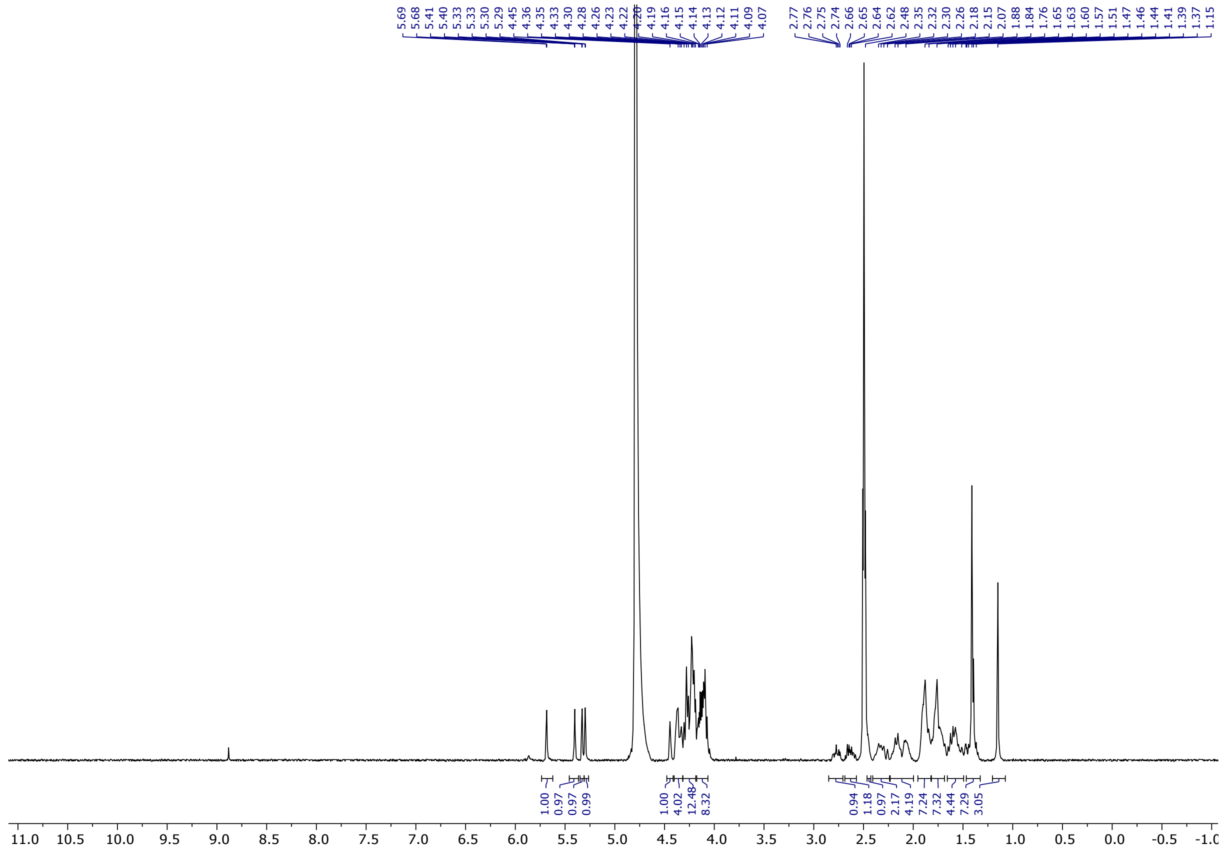
5.69 5.68 5.41 5.40 5.33 5.33 5.30 5.29 4.45 4.36 4.35 4.33 4.30 4.28 4.26 4.23 4.22 4.20 4.19 4.16 4.15 4.14 4.13 4.12 4.11 4.09 4.07
2.77 2.76 2.75 2.74 2.66 2.65 2.64 2.62 2.48 2.35 2.32 2.30 2.26 2.18 2.15 2.07 1.88 1.84 1.76 1.65 1.63 1.60 1.57 1.51 1.47 1.46 1.44 1.41 1.39 1.37 1.15
1.00 0.97 0.97 0.99 1.00 4.02 12.48 8.32 0.94 1.18 0.97 2.17 4.19 7.24 7.32 4.44 7.29 3.05
11.0 10.5 10.0 9.5 9.0 8.5 8.0 7.5 7.0 6.5 6.0 5.5 5.0 4.5 4.0 3.5 3.0 2.5 2.0 1.5 1.0 0.5 0.0 -0.5 -1.0

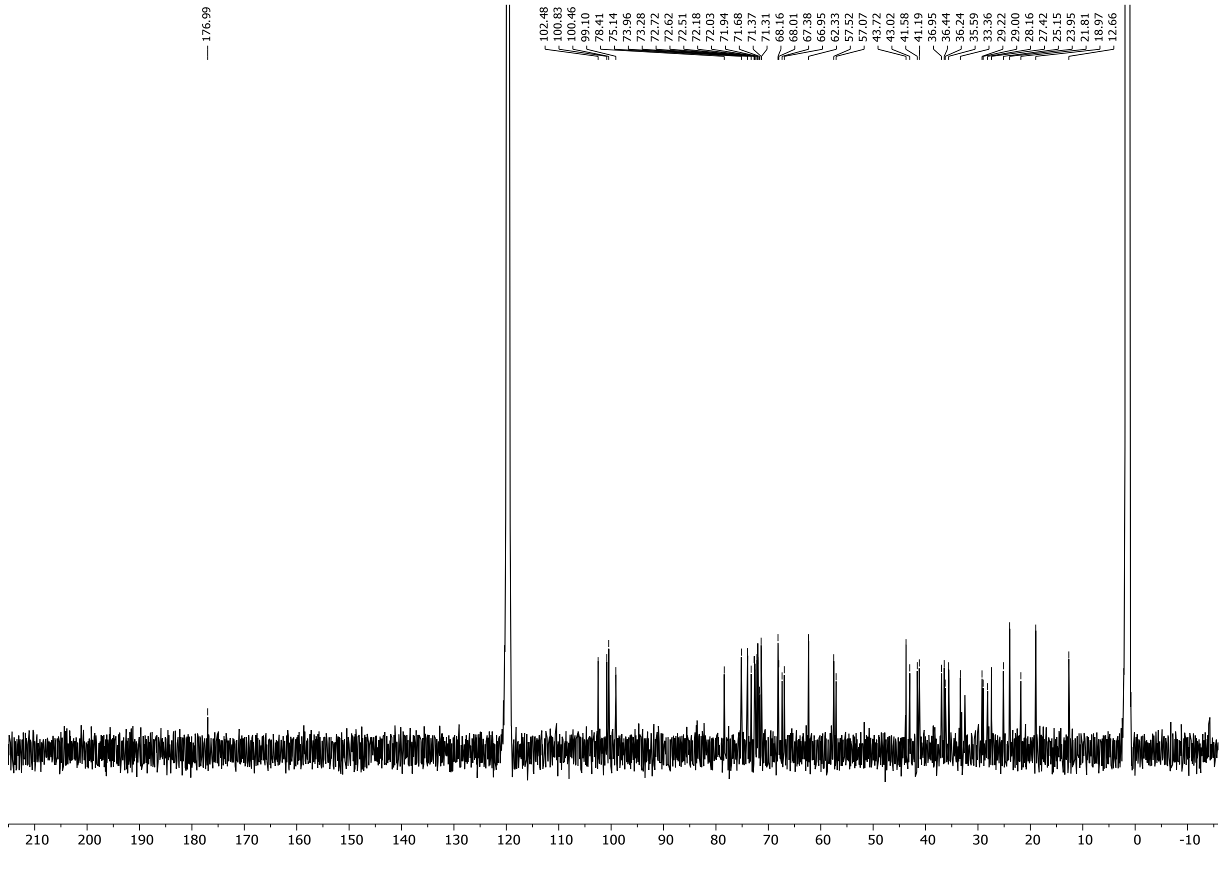
176.99
102.48 100.83 100.46 99.10 78.41 75.14 73.96 73.28 72.72 72.62 72.51 72.18 72.03 71.94 71.68 71.37 71.31 68.16 68.01 67.38 66.95 62.33 57.52 57.07 43.72 43.02 41.58 41.19 36.95 36.44 36.24 35.59 33.36 29.22 29.00 28.16 27.42 25.15 23.95 21.81 18.97 12.66
210 200 190 180 170 160 150 140 130 120 110 100 90 80 70 60 50 40 30 20 10 0 -10

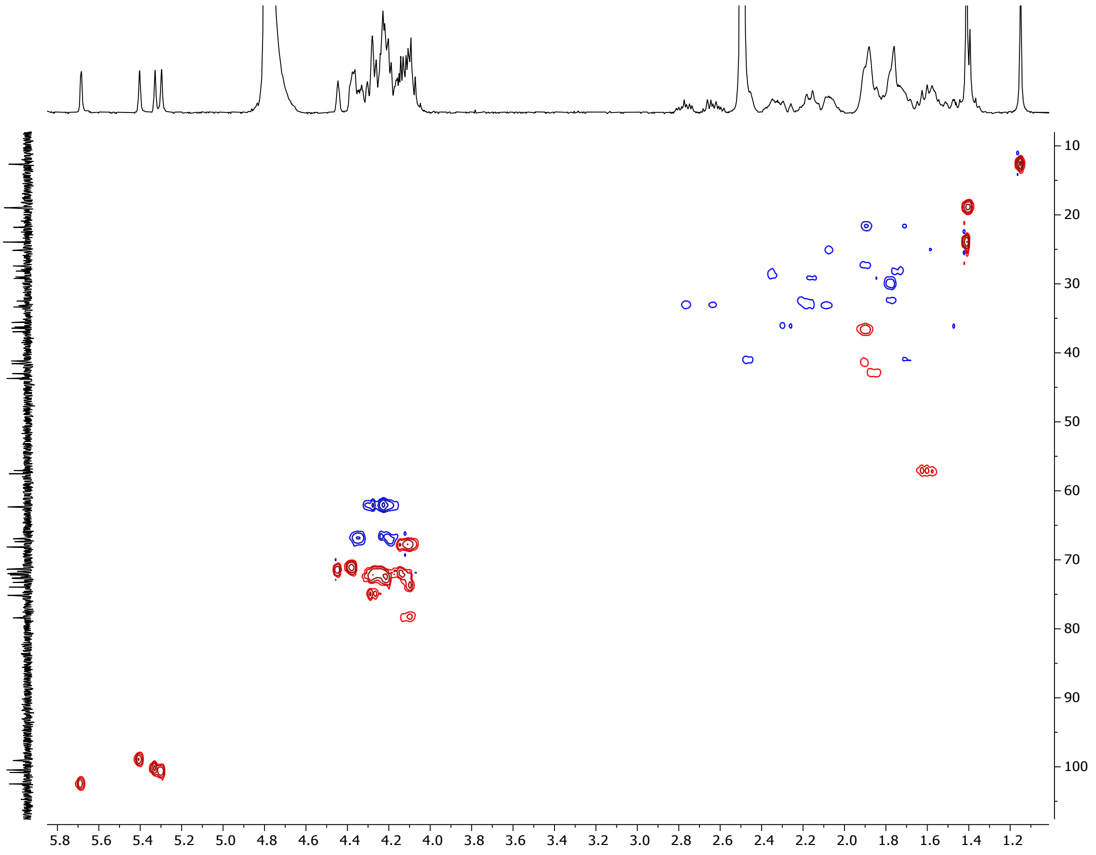

10
20
30
40
50
60
70
80
90
100
5.8 5.6 5.4 5.2 5.0 4.8 4.6 4.4 4.2 4.0 3.8 3.6 3.4 3.2 3.0 2.8 2.6 2.4 2.2 2.0 1.8 1.6 1.4 1.2

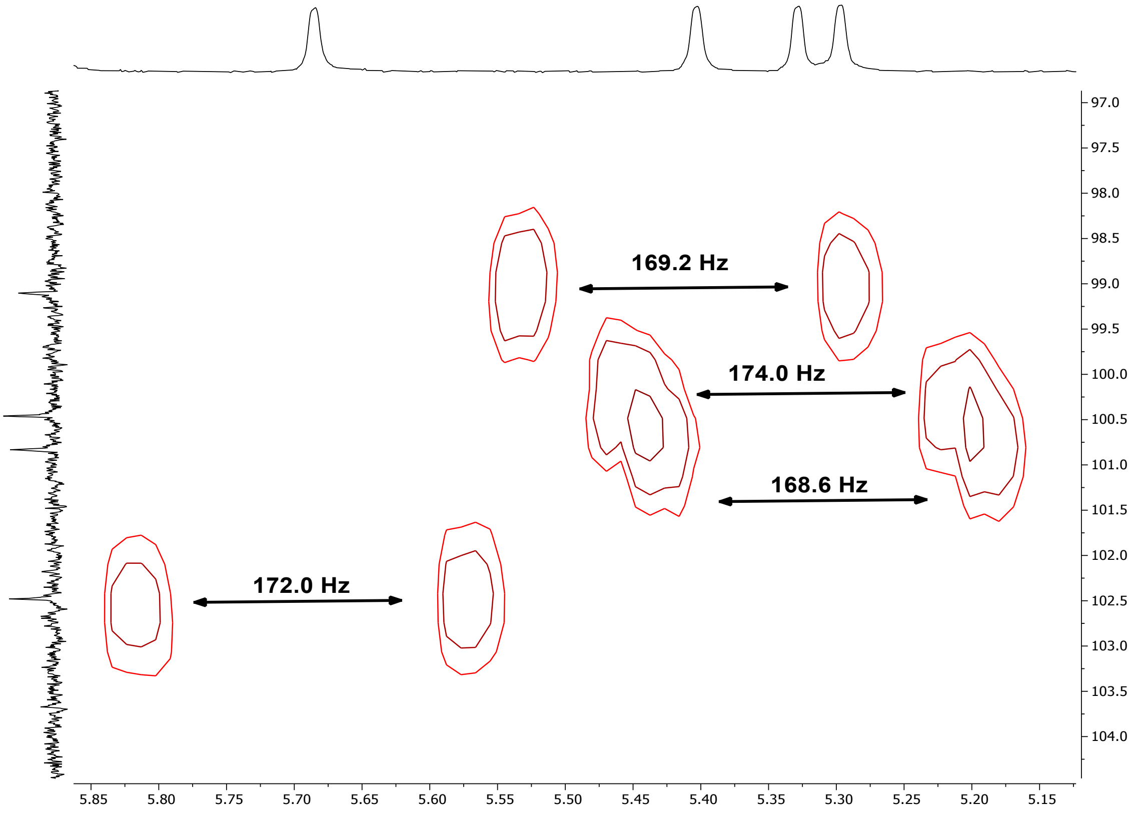

169.2 Hz
174.0 Hz
168.6 Hz
172.0 Hz
97.0
97.5
98.0
98.5
99.0
99.5
100.0
100.5
101.0
101.5
102.0
102.5
103.0
103.5
104.0
5.85 5.80 5.75 5.70 5.65 5.60 5.55 5.50 5.45 5.40 5.35 5.30 5.25 5.20 5.15

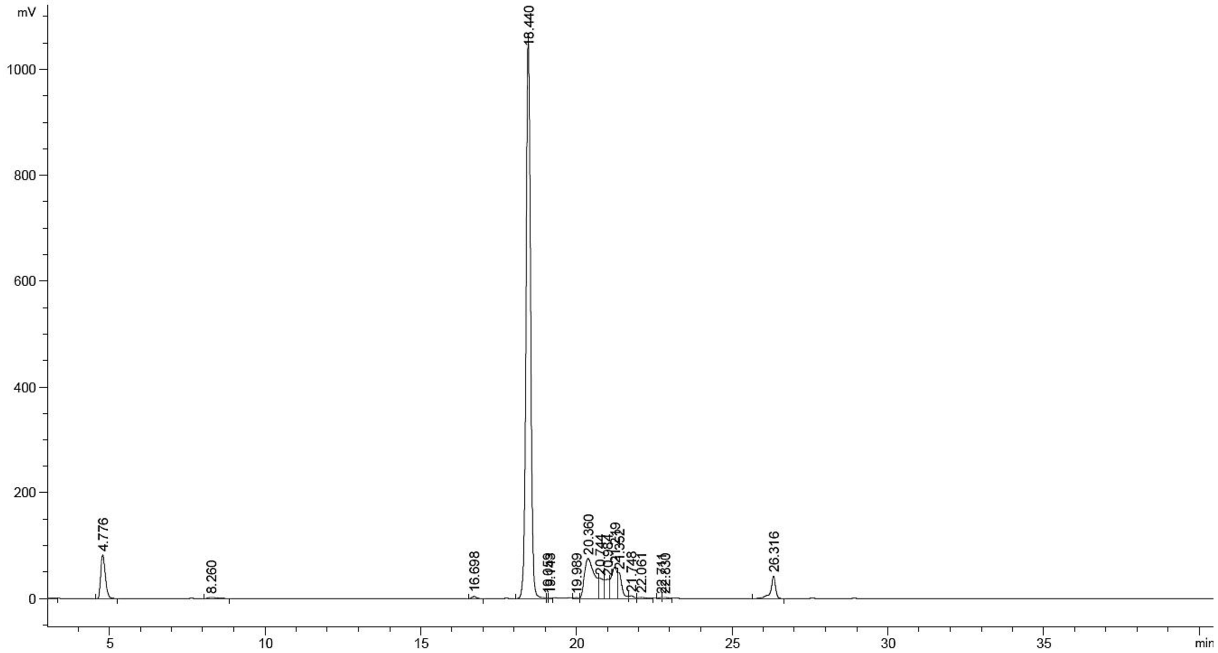

Figure 14: NP-HPLC trace of crude **16** (10-100%).

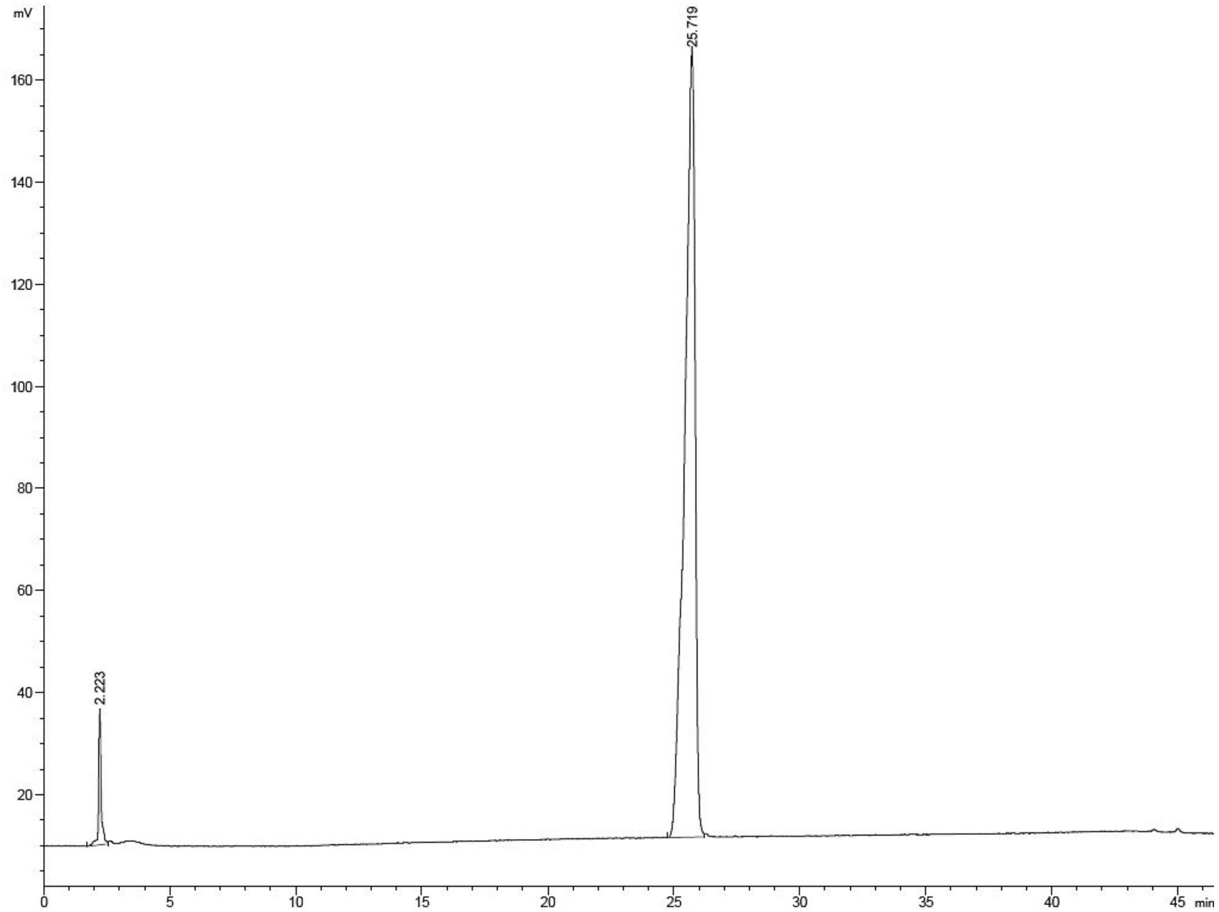

Figure 15: RP-HPLC trace of pure **16** (10-100%).

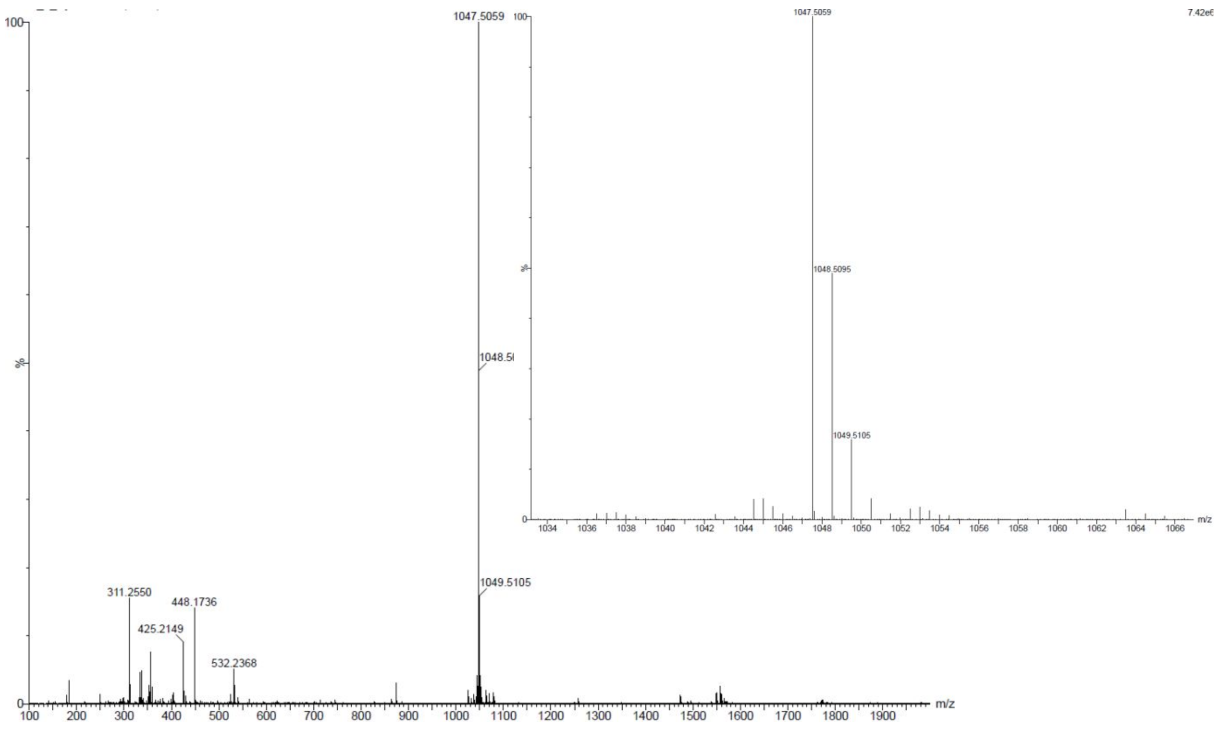

Figure 16: Q-TOF MS-spectrum of **16**.

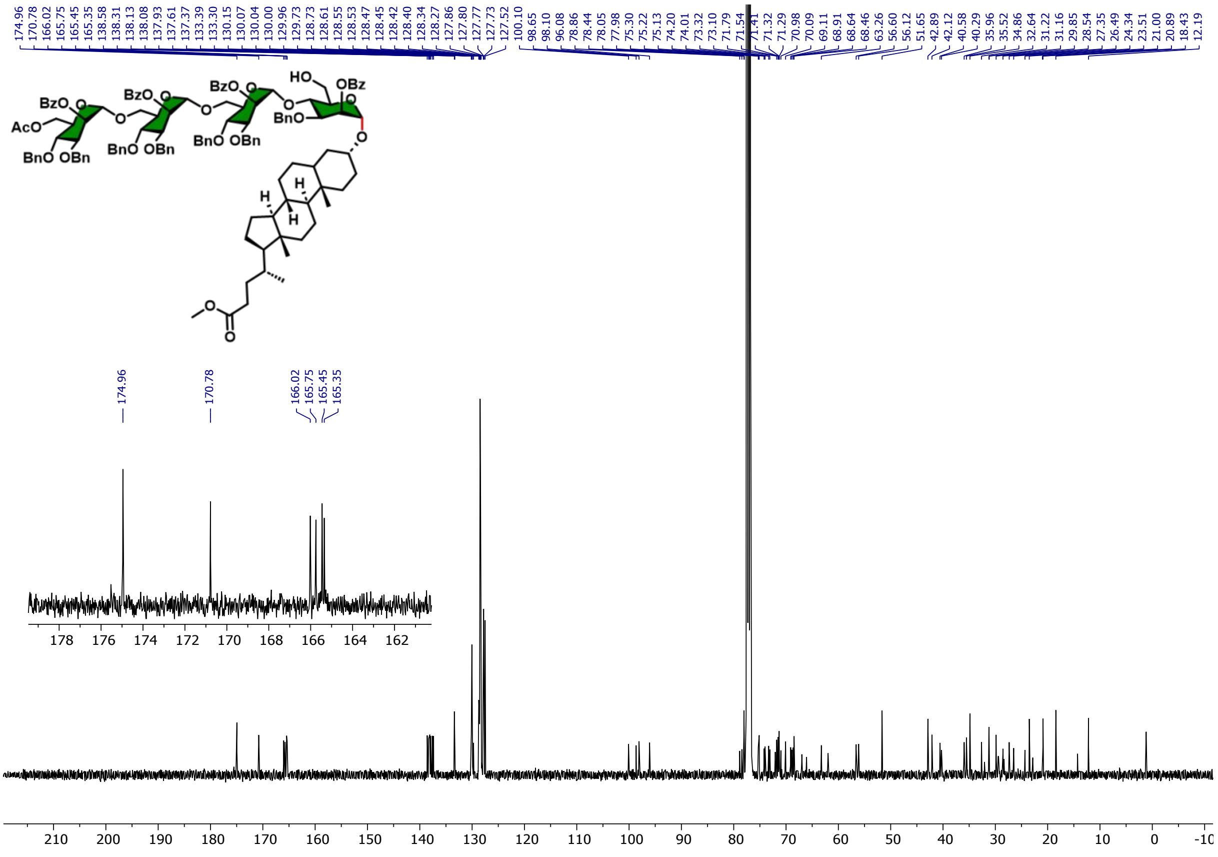

Figure 17: ^{13}C NMR spectrum of fully protected **16**.

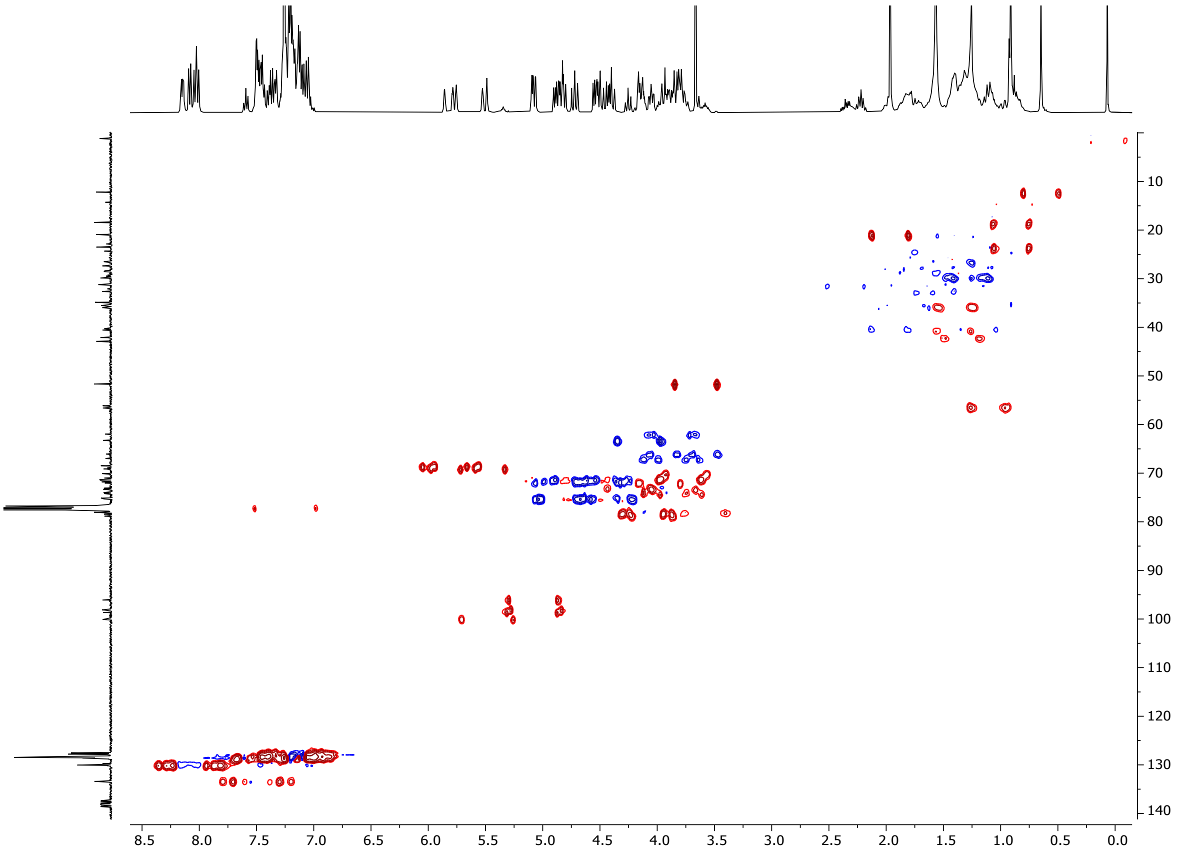

Figure 18: Coupled HSQC NMR spectrum of fully protected **16**.

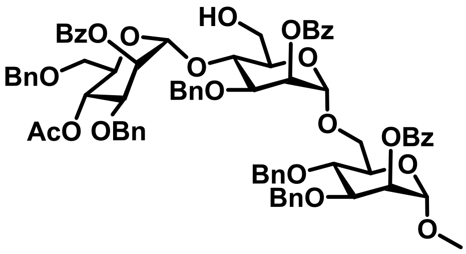

Methyl 4-O-acetyl-2-O-benzoyl-3,6-di-O-benzyl-*α*-D-mannopyranosyl-(1→4)-2-O-benzoyl-3,6-di-O-benzyl-*α*-D-mannopyranosyl-(1→6)-2-O-benzoyl-3,4-di-O-benzyl-*α*-D-mannopyranoside (18)

Step	Building Block	Modules	Notes
AGA	BB **9**, 6 eq.	**A** – first coupling	-40°C (T1) 5 min<br>-20°C (T2) 35 min
		**B** – RV Wash	
		**C** – Acidic Wash	
		**D** – Capping	
		**E** – Fmoc Deprotection	
		**C** – Acidic Wash	
	BB **10**, 3.5 eq.	**F** – Phosphate coupling	-30°C (T1) 10 min<br>0°C (T2) 20 min
		**E** – Fmoc Deprotection	
		**D** – Capping	
		**C** – Acidic Wash	
	BB **19**, 10 eq.	**G** – Thioglycoside Coupling	20°C (T1) 10 min<br>30°C (T2) 35 min
Post AGA		**J** – Batch Photocleavage	16 h
Purification		**L** – NP - (10-100)	R_t = 20.0 min

After a procedure including automated glycan assembly, photo-cleavage, purification and lyophilization, **18** was obtained as a translucent resin (5.4 mg, 4.01 µmol, 19%).

R_t (NP - 10-100) = 20.0 min.

^{1}H NMR (600 MHz, $CDCl_3$) *δ* 8.21 – 8.13 (m, 2H), 8.07 – 8.02 (m, 2H), 7.97 – 7.92 (m, 2H), 7.61 – 7.54 (m, 1H), 7.55 – 7.47 (m, 4H), 7.42 – 7.26 (m, 16H), 7.25 – 7.12 (m, 10H), 7.11 – 7.03 (m, 2H), 7.01 – 6.93 (m, 1H), 5.74 (dd, *J* = 3.1, 1.9 Hz, 1H, H2''), 5.68 (ddd, *J* = 4.8, 3.2, 1.9 Hz, 2H, H2 and H2'), 5.47 (d, *J* = 1.9 Hz, 1H, α-1→4-H1''), 5.17 (t, *J* = 9.8 Hz, 1H, H4''), 5.10 (d, *J* = 1.8 Hz, 1H, α-1→6-H1'), 4.95 (d, *J* = 11.3 Hz, 1H), 4.89 – 4.82 (m, 2H), 4.67 (d, *J* = 11.6 Hz, 2H), 4.63 – 4.53 (m, 3H), 4.46 (d, *J* = 11.1 Hz, 2H), 4.38 – 4.29 (m, 2H), 4.13 (dd, *J* = 8.9, 3.4 Hz, 1H), 4.05 (dd, *J* = 9.4, 3.1 Hz, 1H), 4.02 – 3.95 (m, 1H), 3.92 (d, *J* = 9.0 Hz, 1H), 3.87 (ddd, *J* = 9.8, 4.6, 1.8 Hz, 2H), 3.81 – 3.71 (m, 3H), 3.69 – 3.56 (m, 3H), 3.51 (dd, *J* = 10.3, 2.1 Hz, 1H), 3.38 (s, 3H, OAc), 2.92 (t, *J* = 6.4 Hz, 1H), 1.93 (s, 3H, OMe).

^{13}C NMR (151 MHz, $CDCl_3$) δ 169.9 (CO-Ac), 166.0 (CO-Bz), 165.7 (CO-Bz), 165.3 (CO-Bz), 138.5, 138.0, 137.9, 137.4, 136.9, 133.6, 133.4, 133.3, 130.1, 130.1, 130.0, 129.6, 128.8, 128.7, 128.6, 128.6, 128.5, 128.5, 128.5, 128.4, 128.4, 128.2, 128.0, 127.9, 127.9, 127.7, 127.7, 127.7, 127.5, 99.9, 99.1, 98.1, 78.7, 75.1, 74.9, 74.3, 73.9, 72.0, 71.6, 71.4, 71.2, 70.7, 70.6, 69.3, 68.9, 68.5, 68.1, 68.0, 66.6, 61.0, 55.2 (CH_3-Ac), 21.0 (OMe).

HRMS (ESI): $C_{77}H_{78}NaO_{20}$ $[M+Na]^+$; calculated: 1345.4984, found: 1345.5159.

Optical rotation: $[\alpha]_D^{25}$ = -10.0° (c = 0.1, $CHCl_3$)

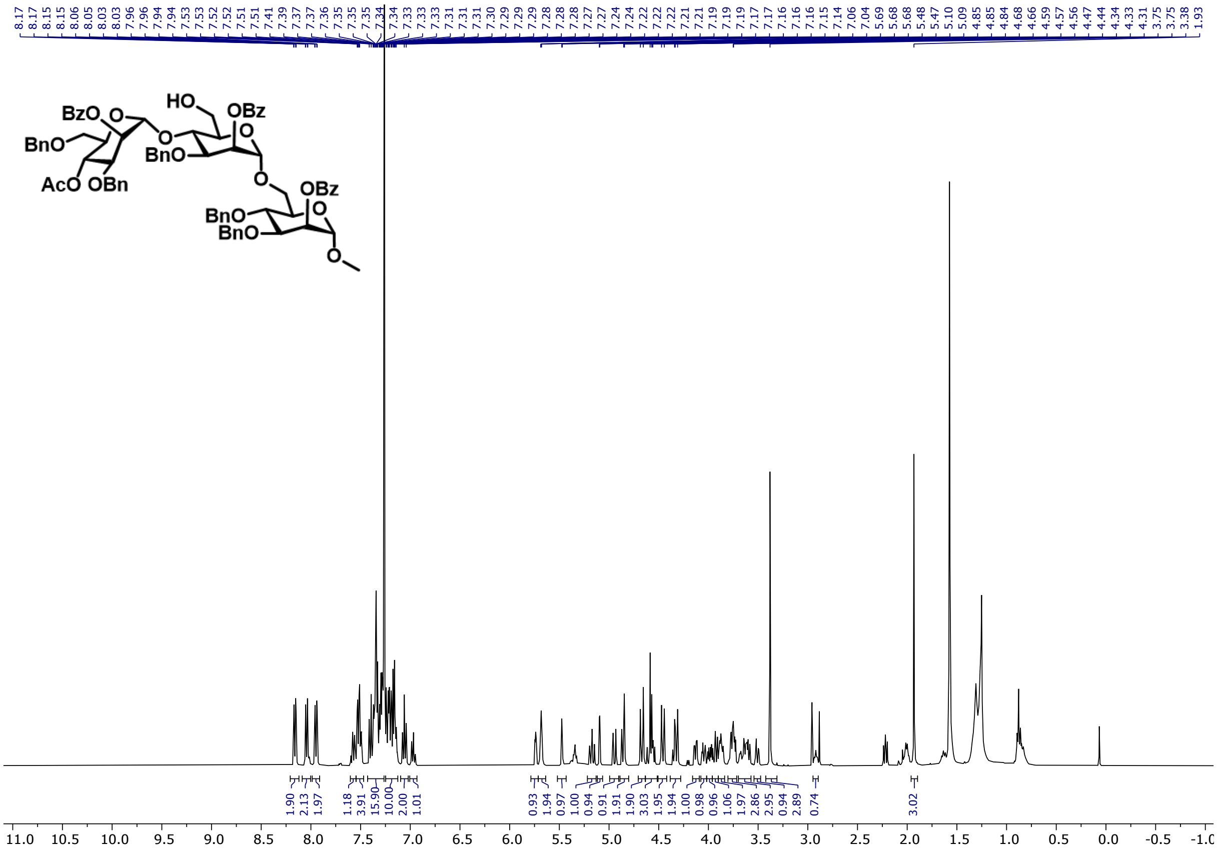
8.17 8.17 8.15 8.15 8.06 8.05 8.03 8.03 7.96 7.96 7.94 7.94 7.53 7.53 7.53 7.52 7.52 7.51 7.51 7.41 7.39 7.37 7.37 7.36 7.35 7.35 7.34 7.34 7.33 7.33 7.31 7.31 7.30 7.29 7.29 7.29 7.28 7.28 7.27 7.27 7.24 7.24 7.22 7.22 7.21 7.21 7.19 7.19 7.17 7.17 7.16 7.16 7.15 7.14 7.06 7.04 5.69 5.68 5.68 5.48 5.47 5.10 5.09 4.85 4.84 4.68 4.66 4.59 4.57 4.56 4.47 4.44 4.34 4.33 4.31 3.75 3.75 3.38 1.93
BzO
HO
OBz
BnO
BnO
AcO
OBn
OBz
BnO
BnO
1.90 2.13 1.97 1.18 3.91 15.90 10.00 2.00 1.01 0.93 1.94 0.97 1.00 0.94 0.91 1.91 1.90 3.03 1.95 1.94 1.00 0.98 0.96 1.06 1.97 2.86 2.95 0.94 2.89 0.74 3.02
11.0 10.5 10.0 9.5 9.0 8.5 8.0 7.5 7.0 6.5 6.0 5.5 5.0 4.5 4.0 3.5 3.0 2.5 2.0 1.5 1.0 0.5 0.0 -0.5 -1.0

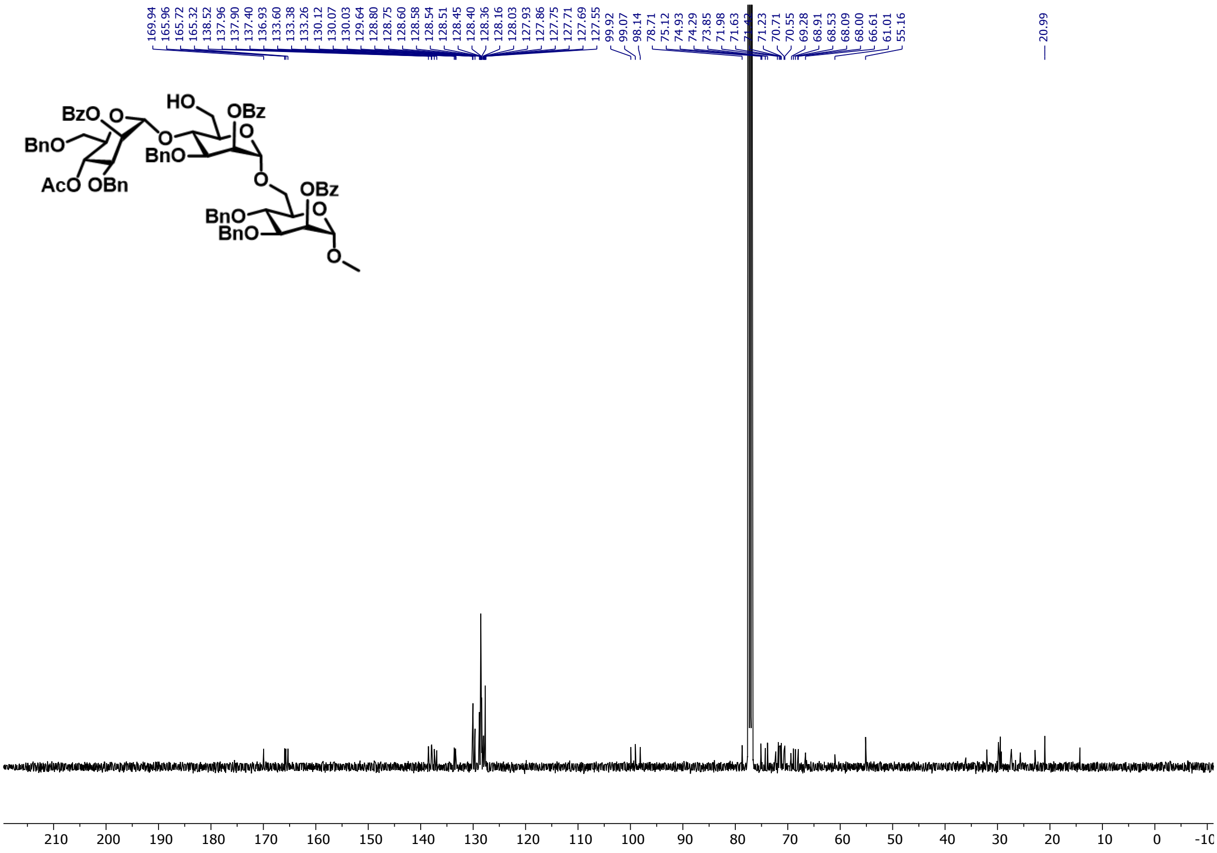
169.94 165.96 165.72 165.32 138.52 137.96 137.90 137.40 136.93 133.60 133.38 133.26 130.12 130.07 130.03 129.64 128.80 128.75 128.60 128.58 128.54 128.51 128.45 128.40 128.36 128.16 128.03 127.93 127.86 127.75 127.71 127.69 127.55 99.92 99.07 98.14 78.71 75.12 74.93 74.29 73.85 71.98 71.63 71.23 70.71 70.55 69.28 68.91 68.53 68.09 68.00 66.61 61.01 55.16 20.99
BzO
HO
OBz
BnO
BnO
AcO
OBn
OBz
BnO
BnO
210 200 190 180 170 160 150 140 130 120 110 100 90 80 70 60 50 40 30 20 10 0 -10

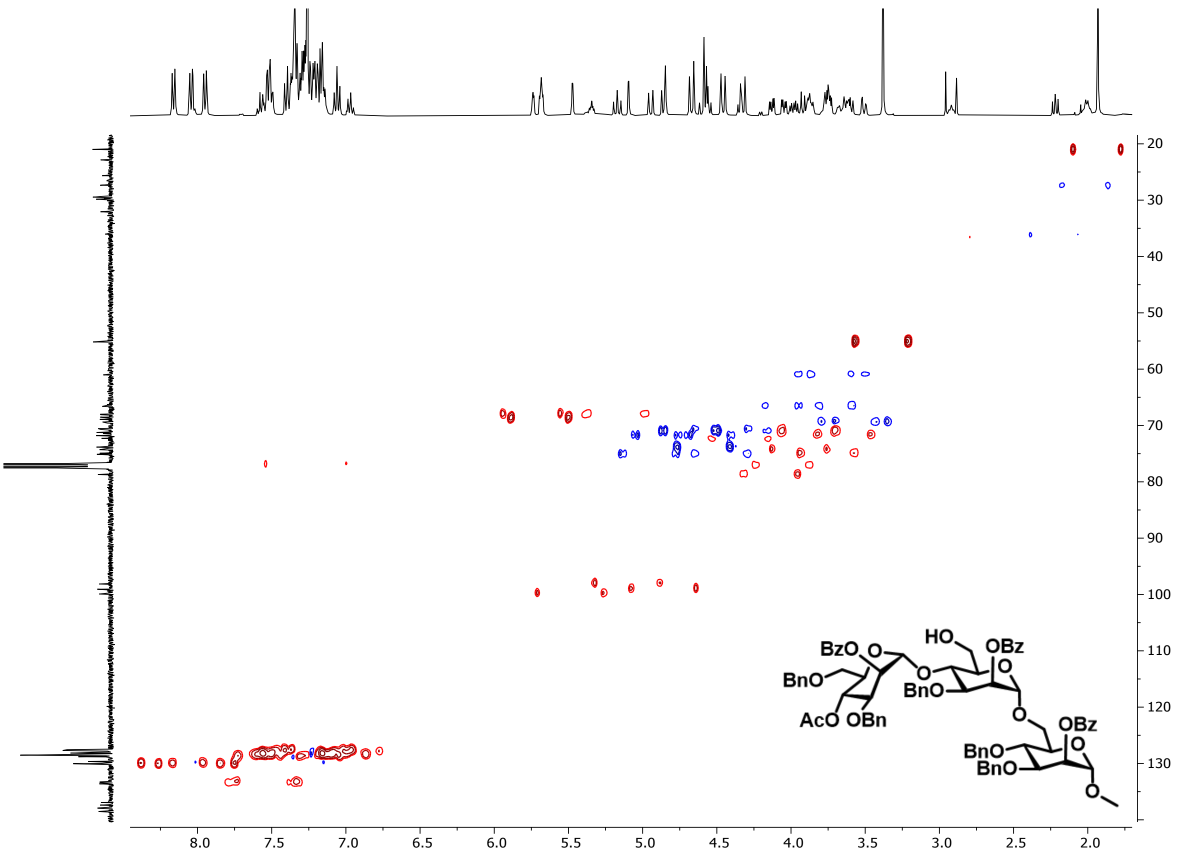
BzO
BnO
AcO OBn
HO
OBz
BnO
OBz
BnO
BnO

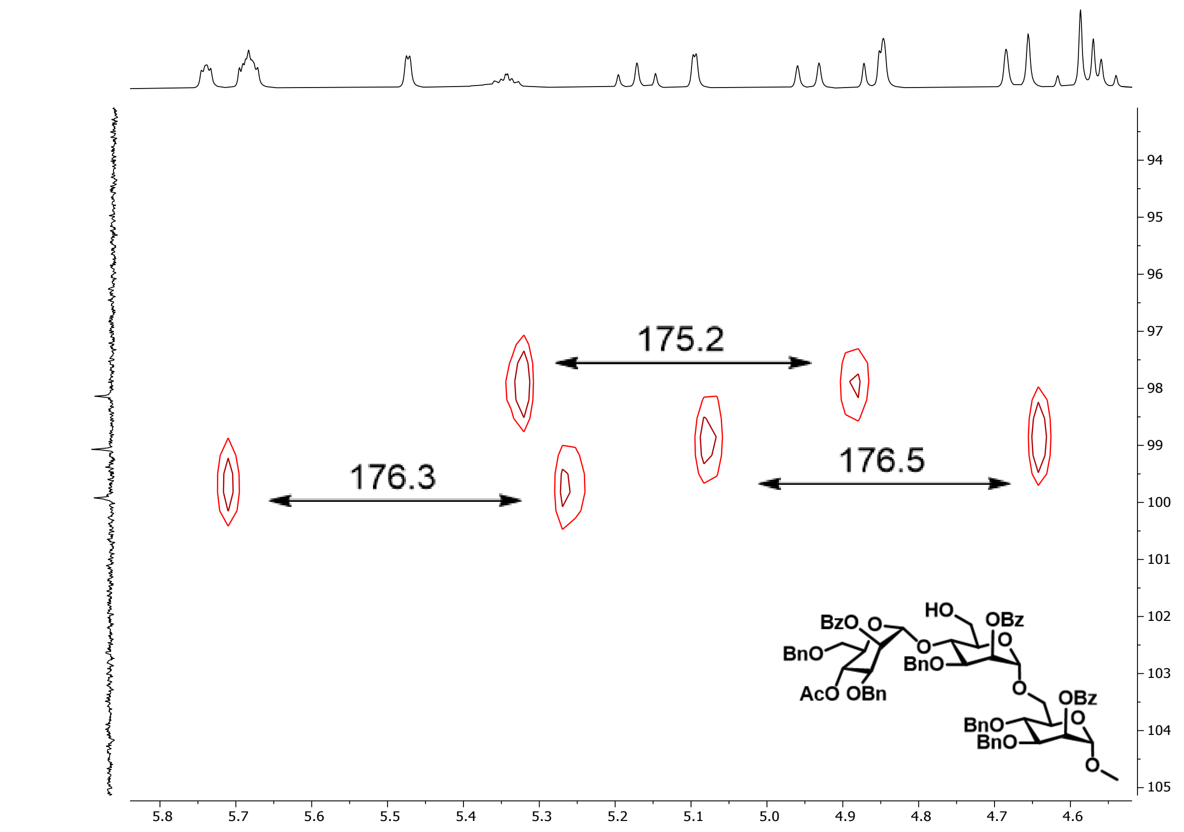
175.2
176.3
176.5
BzO
BnO
AcO OBn
HO
OBz
BnO
OBz
BnO
BnO

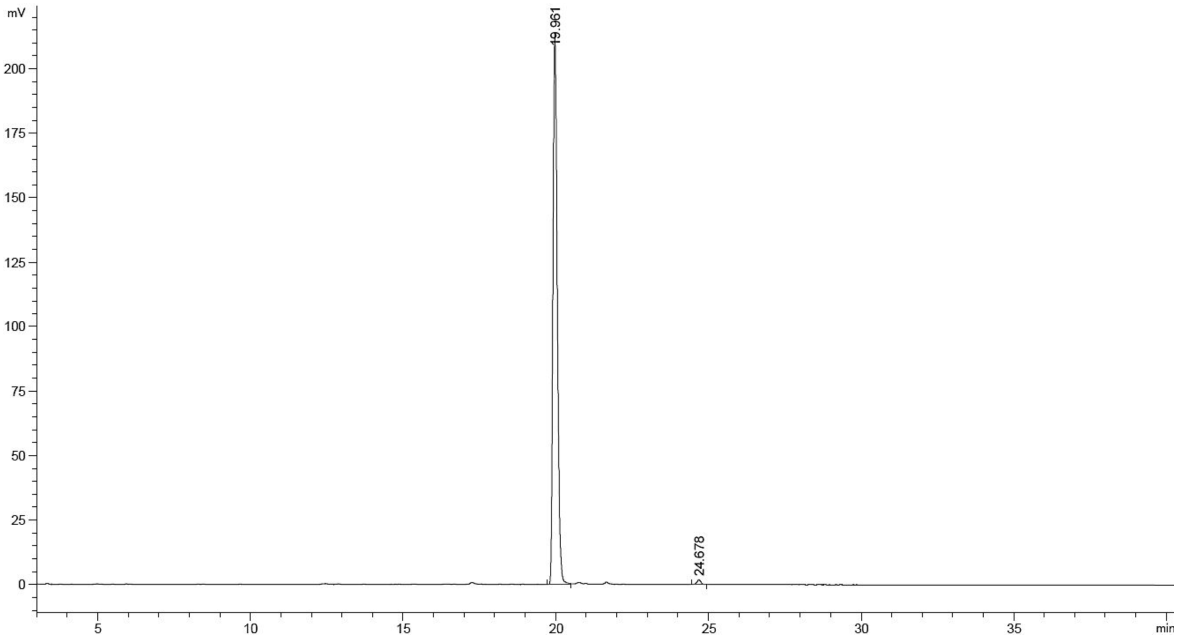

Figure 19: NP-HPLC trace of crude **18** (10-100%).

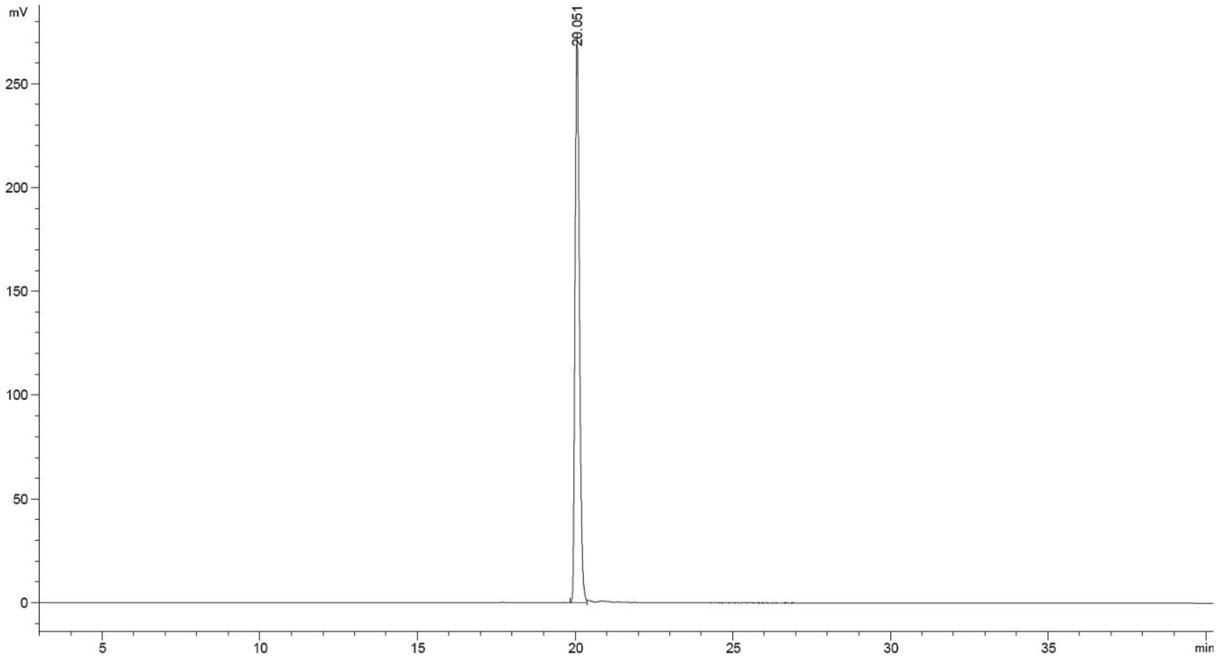

Figure 20: NP-HPLC trace of pure **18** (10-100%).

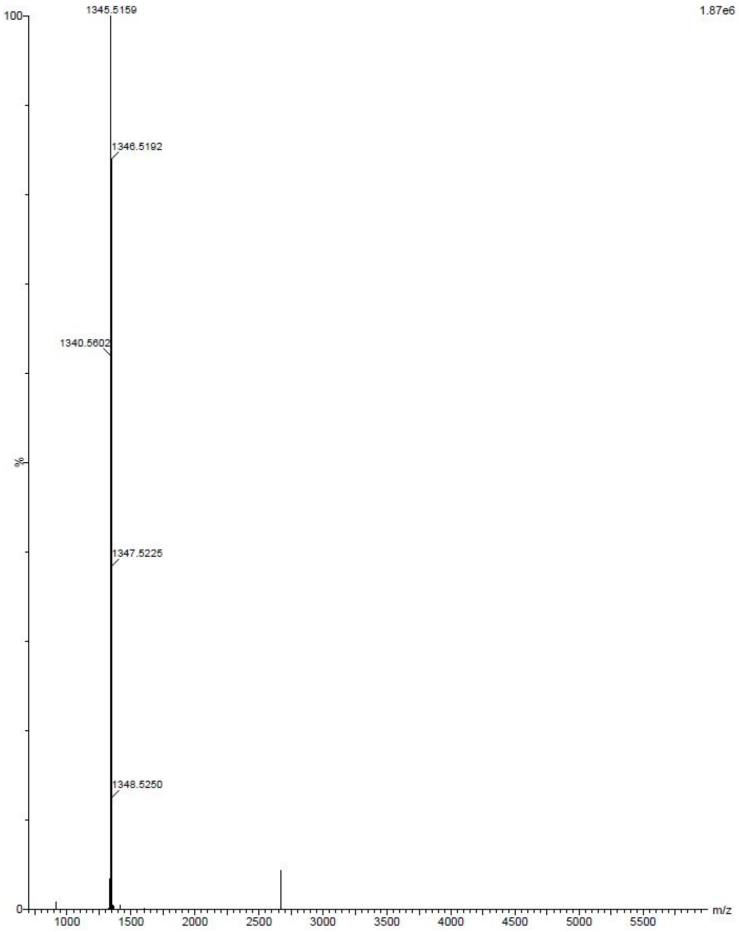

Figure 21: Q-TOF MS-spectrum of **18**.

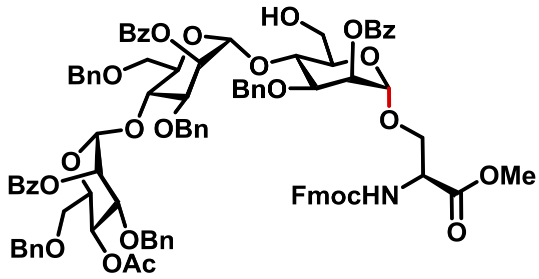

Methyl (((9H-fluoren-9-yl)methoxy)carbonyl)-*L*-serine 4-O-acetyl-2-O-benzoyl-3,6-di-O-*α*-D-mannopyranosyl-(1→4)-2-O-benzoyl-3,6-di-O-benzyl-*α*-D-mannopyranosyl-(1→4)-2-O-benzoyl-3-O-benzyl-*α*-D-mannopyranoside (20)

Step	Building Block	Modules	Notes
**AGA**	BB **9**, 6 eq.	**A** – first coupling	-40°C (T1) 5 min<br>-20°C (T2) 35 min
		**B** – RV Wash	
		**C** – Acidic Wash	
		**D** – Capping	
		**E** – Fmoc Deprotection	
		**C** – Acidic Wash	
	**2x**<br>BB **10**, 4 eq.	**F** – Phosphate coupling	-30°C (T1) 20 min<br>0°C (T2) 20 min
		**E** – Fmoc Deprotection	
		**D** – Capping	
		**C** – Acidic Wash	
	BB **21**, 10 eq.	**G** – Thioglycoside Coupling	20°C (T1) 5 min<br>35°C (T2) 35 min
**Post AGA**		**J** – Batch Photocleavage	16 h
**Purification**		**L** – NP - (10 – 100%)	R_t =23.6 min

After a procedure including automated glycan assembly, photo-cleavage, purification and lyophilization, **20** was obtained as a colorless resin (8.2 mg, 4.96 µmol, 25%).

R_t (NP - 10 – 100%) = 23.6 min.

^{1}H NMR (600 MHz, $CDCl_3$) δ 8.06 – 8.00 (m, 2H), 7.92 (ddd, J = 7.4, 6.2, 1.3 Hz, 4H), 7.76 (d, J = 7.6 Hz, 2H), 7.66 – 7.63 (m, 1H), 7.57 – 7.52 (m, 1H), 7.49 (dddt, J = 7.2, 5.8, 4.6, 1.3 Hz, 2H), 7.45 – 7.38 (m, 4H), 7.37 – 7.26 (m, 16H), 7.25 – 7.07 (m, 12H), 7.06 – 6.99 (m, 3H), 5.80 (d, J = 8.4 Hz, 1H, NH), 5.76 (t, J = 2.5 Hz, 1H, H2''), 5.70 – 5.65 (m, 1H, H2'), 5.53 – 5.51 (m, 1H, α-H1''), 5.49 (d, J = 1.9 Hz, 1H, α-H1'), 5.48 – 5.47 (m, 1H, C2'), 5.34 (t, J = 9.8 Hz, 1H, H4''), 4.93 (d, J = 1.8 Hz, 1H, α-H1), 4.79 (d, J = 10.5 Hz, 1H), 4.70 (d, J = 12.1 Hz, 1H), 4.67 (d, J = 11.1 Hz, 1H), 4.63 – 4.54 (m, 5H), 4.52 (d, J = 14.6 Hz, 1H), 4.49 – 4.39 (m, 4H), 4.37 (d, J = 12.1 Hz, 1H), 4.34 (t, J = 9.6 Hz, 1H), 4.28 (t, J = 6.9 Hz, 1H, CH-Fmoc), 4.10 (t, J = 9.4 Hz, 1H), 4.04 (td, J = 11.1, 3.4 Hz, 2H), 4.01 – 4.00 (m, 1H), 3.98 (dd, J = 6.8, 3.0 Hz, 1H), 3.97 – 3.93 (m, 2H), 3.89 (dd, J = 10.5, 1.8 Hz, 1H), 3.85 (s, 3H), 3.85 – 3.78 (m, 3H), 3.78 – 3.74 (m, 1H), 3.72 (d, J = 9.8 Hz, 1H), 3.44 (d, J = 3.8 Hz, 2H), 2.90 (s, 1H), 1.93 (s, 3H, OMe).

^{13}C NMR (151 MHz, $CDCl_3$) δ 170.6, 169.8, 165.9, 165.3, 156.1, 143.9, 143.8, 141.5, 138.2, 137.9, 137.1, 133.5, 133.2, 130.1, 130.1, 130.0, 129.7, 129.5, 128.9, 128.6, 128.5, 128.5, 128.5, 128.5, 128.4, 128.4, 128.4, 128.3, 128.0, 127.9, 127.8, 127.8, 127.7, 127.6, 127.3, 125.2, 120.2, 99.77 (C1''), 99.5 (C1'), 99.2 (C1), 78.2, 75.2, 73.7, 73.7, 72.3, 71.4, 71.3, 71.2, 69.9, 69.4, 68.6, 68.2, 68.1, 67.3, 61.4, 54.6, 53.1 (CH_3-Ac), 47.3 (CH-Fmoc), 21.1 (OMe).

HRMS (ESI): $C_{95}H_{93}NNaO_{24}$ $[M+Na]^+$; calculated: 1654.5985, found: 1654.6022.

Optical rotation: $[\alpha]_D^{25}$ = -4.0° (c = 0.1, $CHCl_3$)

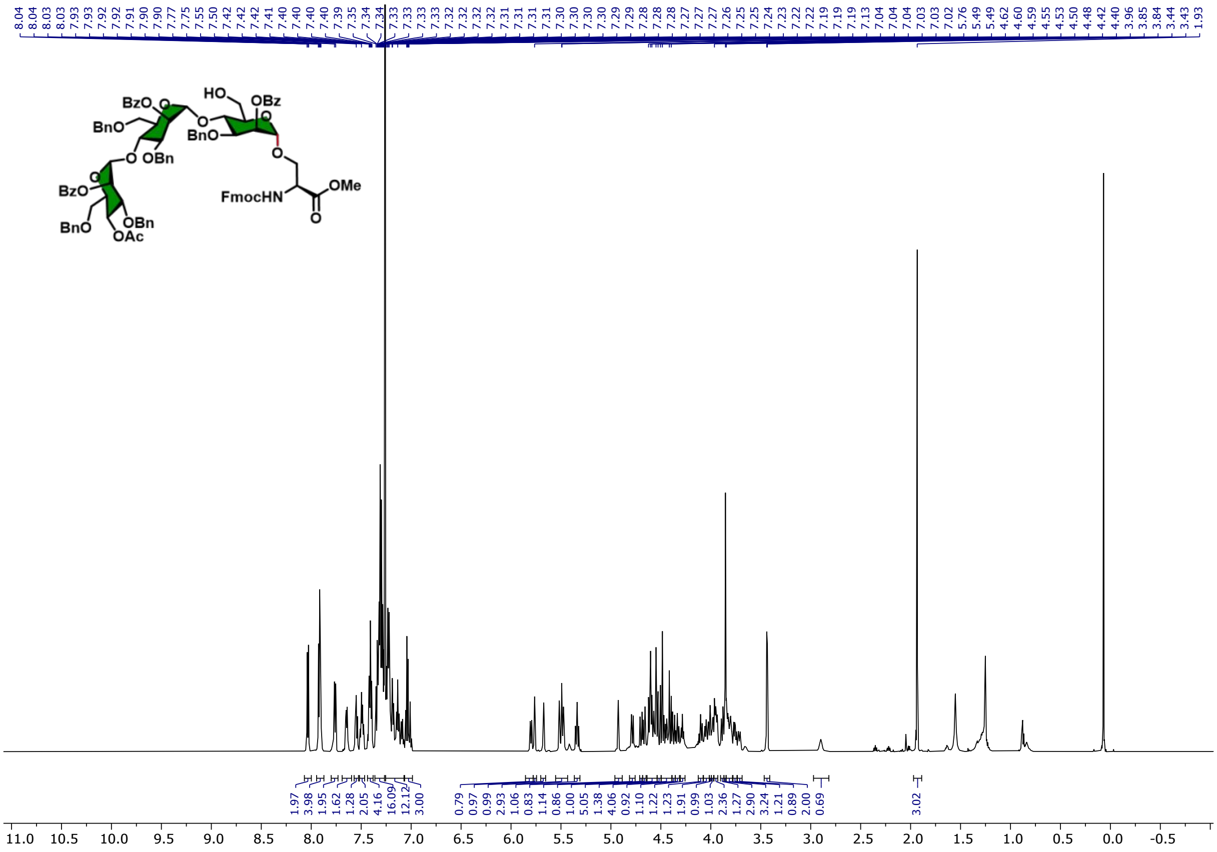
8.04 8.04 8.03 7.93 7.93 7.92 7.92 7.91 7.90 7.90 7.77 7.75 7.55 7.50 7.42 7.42 7.41 7.40 7.40 7.40 7.39 7.35 7.34 7.34 7.33 7.33 7.33 7.32 7.32 7.32 7.31 7.31 7.31 7.30 7.30 7.30 7.29 7.29 7.28 7.28 7.28 7.27 7.27 7.26 7.25 7.25 7.24 7.23 7.22 7.22 7.19 7.19 7.19 7.13 7.04 7.04 7.04 7.03 7.03 7.02 5.76 5.49 5.49 4.62 4.60 4.59 4.55 4.53 4.50 4.48 4.42 4.40 3.96 3.85 3.84 3.44 3.43 1.93
BzO
BnO
OBn
BzO
BnO
OBn
OAc
HO
OBz
BnO
FmocHN
OMe
1.97 3.98 1.95 1.62 1.28 2.05 4.16 16.09 12.12 3.00 0.79 0.97 0.99 2.93 1.06 0.83 1.14 0.86 1.00 5.05 1.38 4.06 0.92 1.10 1.22 1.23 1.91 0.99 1.03 2.36 1.27 2.90 3.24 1.21 0.89 2.00 0.69 3.02
11.0 10.5 10.0 9.5 9.0 8.5 8.0 7.5 7.0 6.5 6.0 5.5 5.0 4.5 4.0 3.5 3.0 2.5 2.0 1.5 1.0 0.5 0.0 -0.5

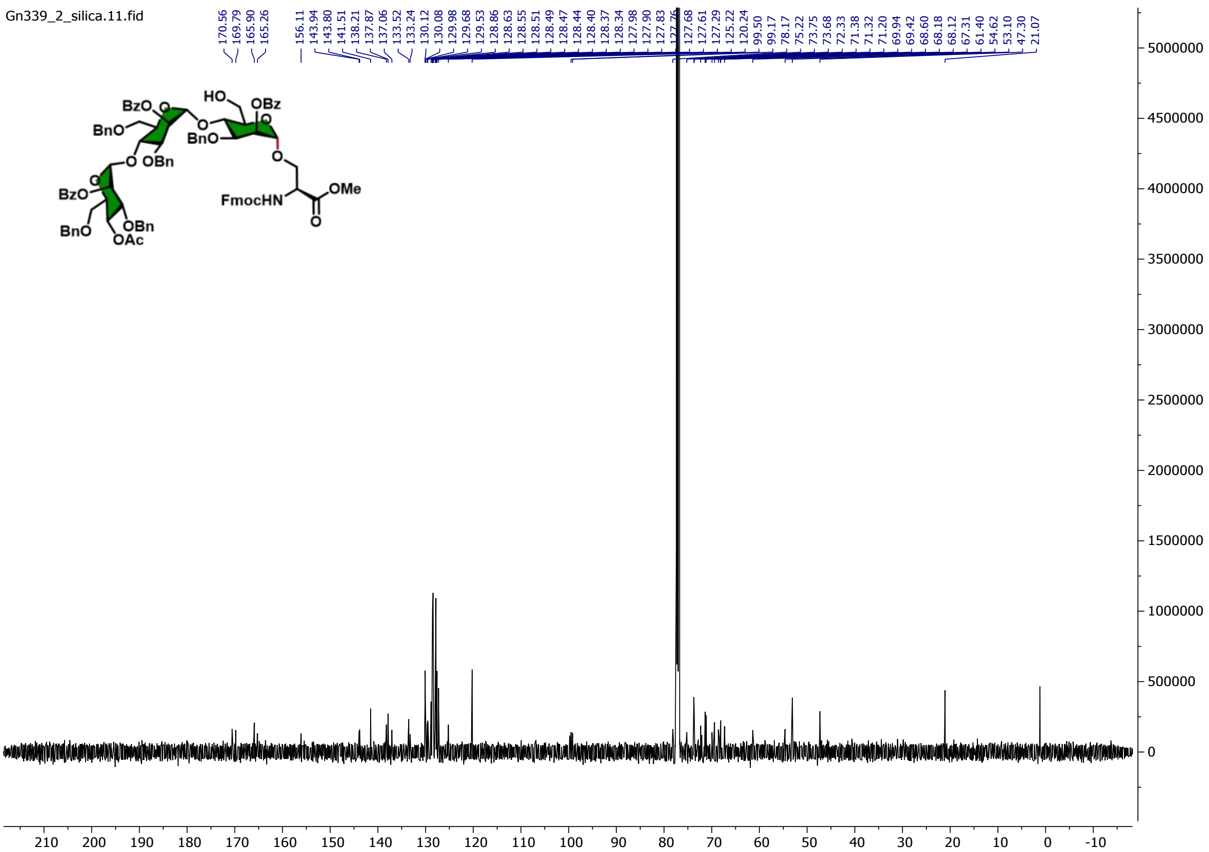
Gn339_2_silica.11.fid
170.56 169.79 165.90 165.26 156.11 143.94 143.80 141.51 138.21 137.87 137.06 133.52 133.24 130.12 130.08 129.98 129.68 129.53 128.86 128.63 128.55 128.51 128.49 128.47 128.44 128.40 128.37 128.34 127.98 127.90 127.83 127.76 127.68 127.61 127.29 125.22 120.24 99.50 99.17 78.17 75.22 73.75 73.68 72.33 71.38 71.32 71.20 69.94 69.42 68.60 68.12 67.31 61.40 54.62 53.10 47.30 21.07
BzO
BnO
OBn
BzO
BnO
OBn
OAc
HO
OBz
BnO
FmocHN
OMe
5000000 4500000 4000000 3500000 3000000 2500000 2000000 1500000 1000000 500000 0
210 200 190 180 170 160 150 140 130 120 110 100 90 80 70 60 50 40 30 20 10 0 -10

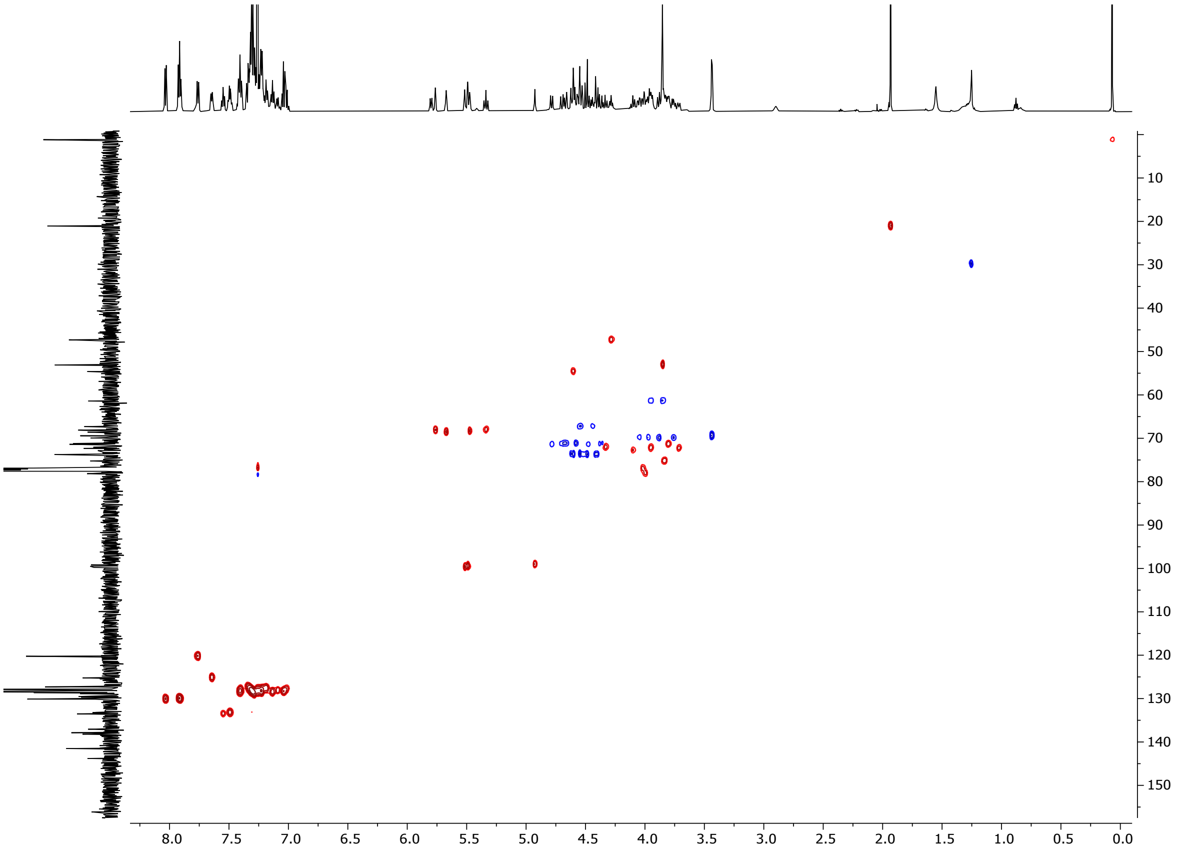
8.0
7.5
7.0
6.5
6.0
5.5
5.0
4.5
4.0
3.5
3.0
2.5
2.0
1.5
1.0
0.5
0.0
10
20
30
40
50
60
70
80
90
100
110
120
130
140
150

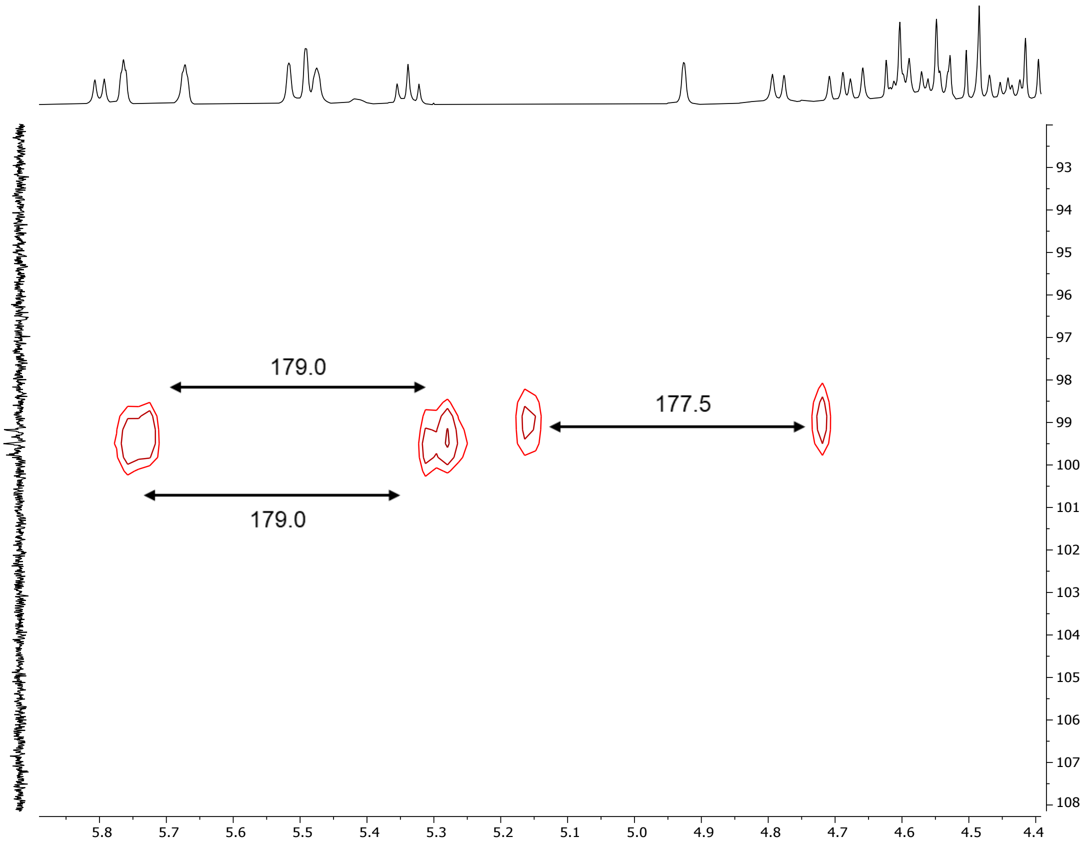
179.0
177.5
179.0
5.8
5.7
5.6
5.5
5.4
5.3
5.2
5.1
5.0
4.9
4.8
4.7
4.6
4.5
4.4
93
94
95
96
97
98
99
100
101
102
103
104
105
106
107
108

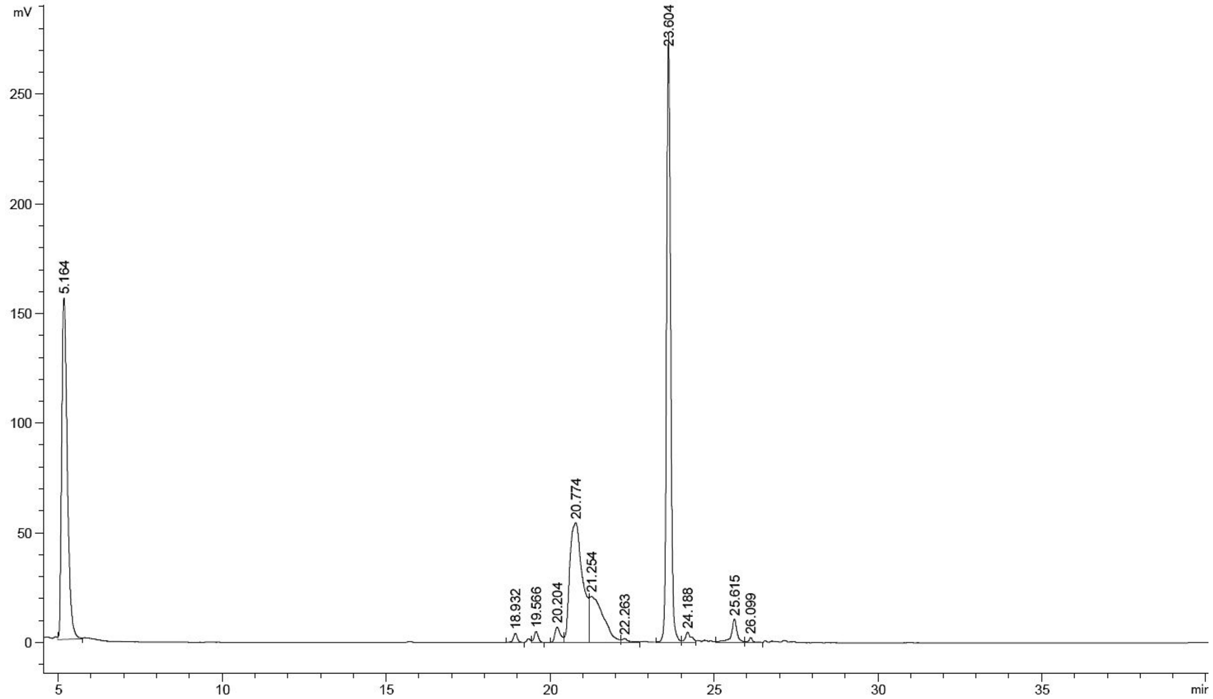

Figure 22: NP-HPLC trace of crude **20** (10-100%).

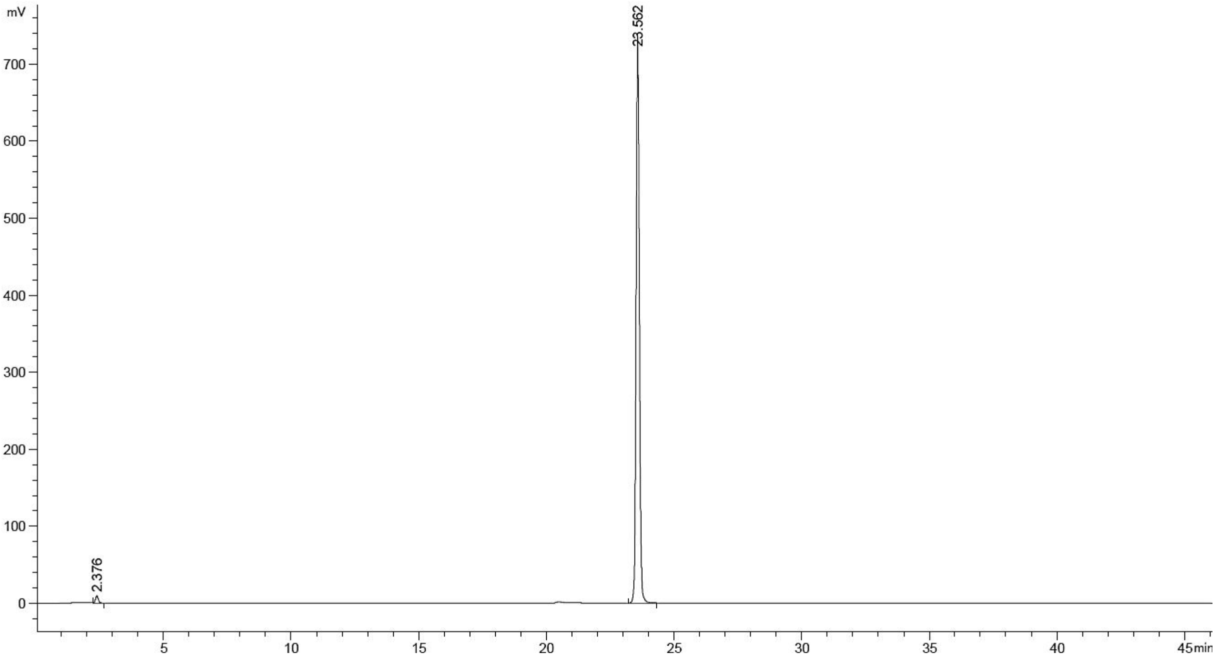

Figure 23: NP-HPLC trace of pure **20** (10-100%).

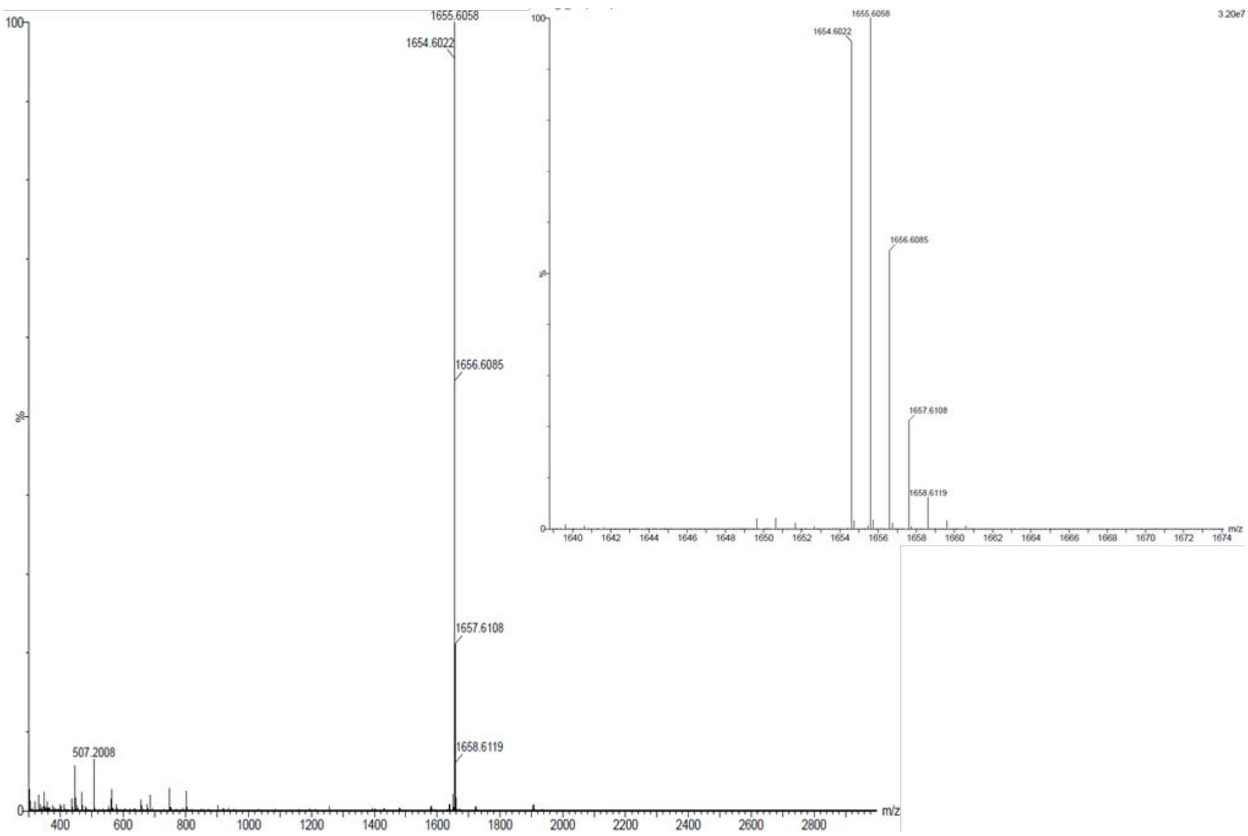

Figure 24: Q-TOF MS-spectrum of **20**.

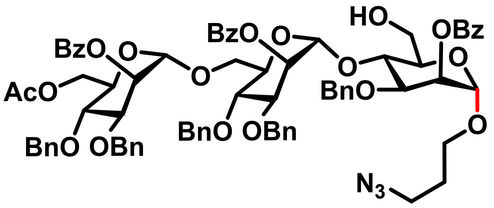

3-azidopropan 6-O-acetyl-2-O-benzoyl-3,4-di-O-*α*-D-mannopyranosyl-(1→6)-2-O-benzoyl-3,4-di-O-benzyl-*α*-D-mannopyranosyl-(1→4)-2-O-benzoyl-3-O-benzyl-*α*-D-mannopyranoside (22)

Step	Building Block	Modules	Notes
AGA	BB **9**, 6 eq.	**A** – first coupling	-40°C (T1) 5 min<br>-20°C (T2) 35 min
		**B** – RV Wash<br>**C** – Acidic Wash<br>**D** – Capping	
	**2x**<br>BB **14**, 4 eq.	**E** – Fmoc Deprotection<br>**C** – Acidic Wash<br>**F** – Phosphate coupling	-30°C (T1) 20 min<br>0°C (T2) 20 min
	1-azidopropanol,<br>20 eq.	**E** – Fmoc Deprotection<br>**D** – Capping<br>**C** – Acidic Wash<br>**G** – Thioglycoside Coupling	20°C (T1) 5 min<br>35°C (T2) 35 min
Post AGA		**J** – Batch Photocleavage	16 h
Purification		**L** – NP - (10 – 100%)	R_t = 19.6 min

After a procedure including automated glycan assembly, photo-cleavage and purification, **22** was obtained as a translucent resin (9.5 mg, 6.72 µmol, 34%).

R_t (NP - 10 – 100%) = 19.6 min.

^{1}H NMR (400 MHz, $CDCl_3$) *δ* 8.11 – 8.07 (m, 2H), 8.07 – 7.99 (m, 4H), 7.60 (ddt, *J* = 8.7, 7.1, 1.3 Hz, 1H), 7.55 – 7.50 (m, 1H), 7.49 – 7.42 (m, 5H), 7.42 – 7.36 (m, 2H), 7.35 – 7.26 (m, 11H), 7.26 – 7.18 (m, 8H), 7.17 – 7.04 (m, 6H), 5.79 (q, *J* = 2.4 Hz, 2H), 5.54 (dd, *J* = 3.2, 1.9 Hz, 1H), 5.45 (d, *J* = 1.9 Hz, 1H), 5.08 (d, *J* = 1.9 Hz, 1H),

4.93 (d, *J* = 1.8 Hz, 1H), 4.89 (d, *J* = 3.4 Hz, 1H), 4.87 – 4.82 (m, 2H), 4.77 (d, *J* = 11.2 Hz, 1H), 4.71 (d, *J* = 11.0 Hz, 1H), 4.59 (s, 1H), 4.56 (s, 1H), 4.52 (d, *J* = 5.2 Hz, 1H), 4.49 (d, *J* = 5.5 Hz, 1H), 4.43 (d, *J* = 11.1 Hz, 1H), 4.33 – 4.26 (m, 2H), 4.23 (t, *J* = 9.5 Hz, 1H), 4.19 – 4.14 (m, 1H), 4.04 (dd, *J* = 9.5, 3.1 Hz, 2H), 4.00 – 3.78 (m, 8H), 3.75 (t, *J* = 9.3 Hz, 1H), 3.68 (ddd, *J* = 9.8, 4.0, 2.2 Hz, 1H), 3.53 (ddd, *J* = 9.9, 6.5, 5.4 Hz, 1H), 3.42 (t, *J* = 6.7 Hz, 2H), 2.00 (s, 3H), 1.95 – 1.87 (m, 2H), 1.67 – 1.60 (m, 1H).

^{13}C NMR (101 MHz, $CDCl_3$) δ 170.8 (CO-Ac), 165.9 (CO-Bz), 165.6 (CO-Bz), 165.4 (CO-Bz), 138.2, 138.1, 138.1, 137.9, 137.1, 133.5, 133.4, 133.3, 130.1, 130.0, 130.0, 129.6, 128.7, 128.6, 128.6, 128.5, 128.5, 128.5, 128.5, 128.4, 128.3, 128.3, 127.9, 127.9, 127.8, 127.7, 100.3 (*α*-C1), 98.2 (*α*-C1), 97.9 (*α*-C1), 78.7, 78.2, 77.4, 75.3, 75.1, 74.1, 73.4, 73.4, 72.1, 71.8, 71.7, 71.4, 71.3, 70.1, 68.9, 68.6, 68.4, 67.2, 64.8, 63.3, 61.9, 48.5 (linker-CH_2), 28.9 (linker-CH_2), 20.9 (Acetate-CH_3).

HRMS (ESI): $C_{79}H_{81}N_3NaO_{20}$ [M+Na]$^+$; calculated: 1414.5413, found: 1414.5322

Optical rotation: $[\alpha]_D^{25}$ = +12.6° (c = 0.5, $CHCl_3$)

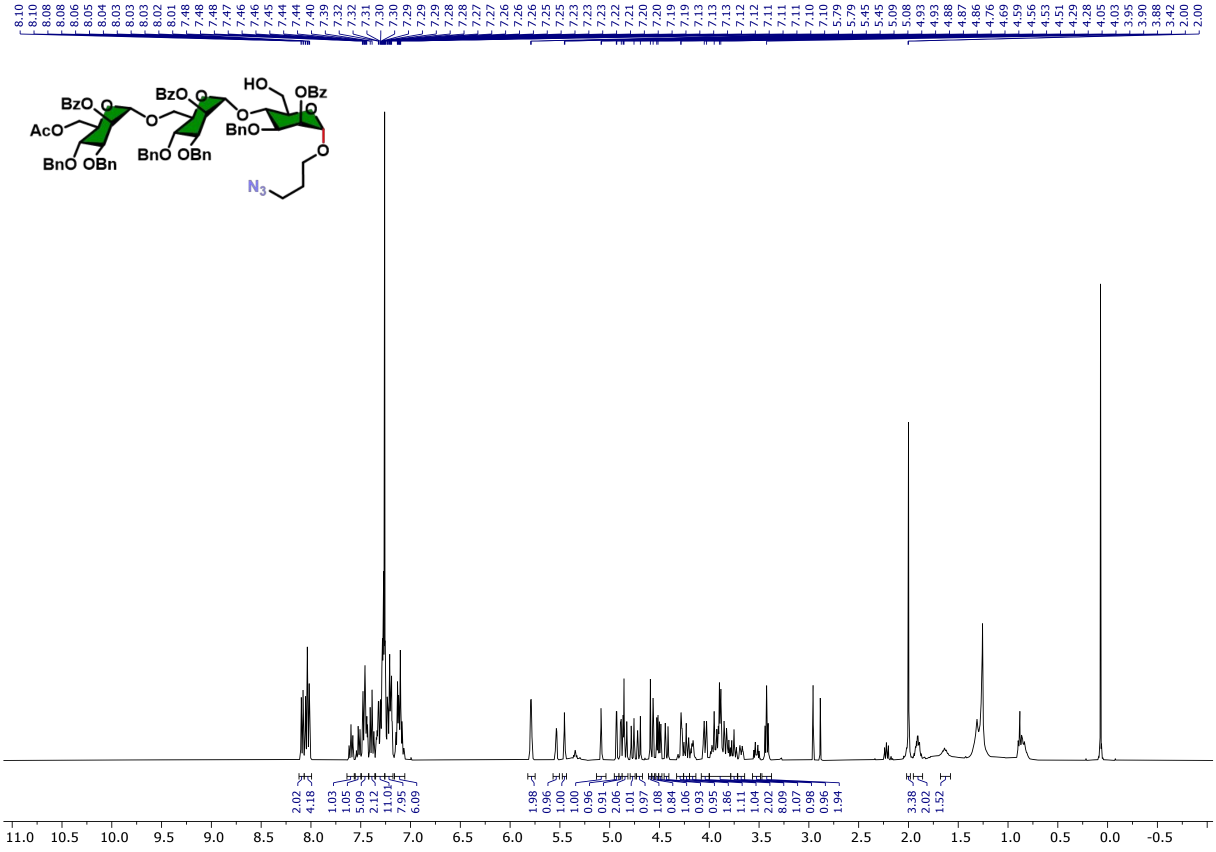

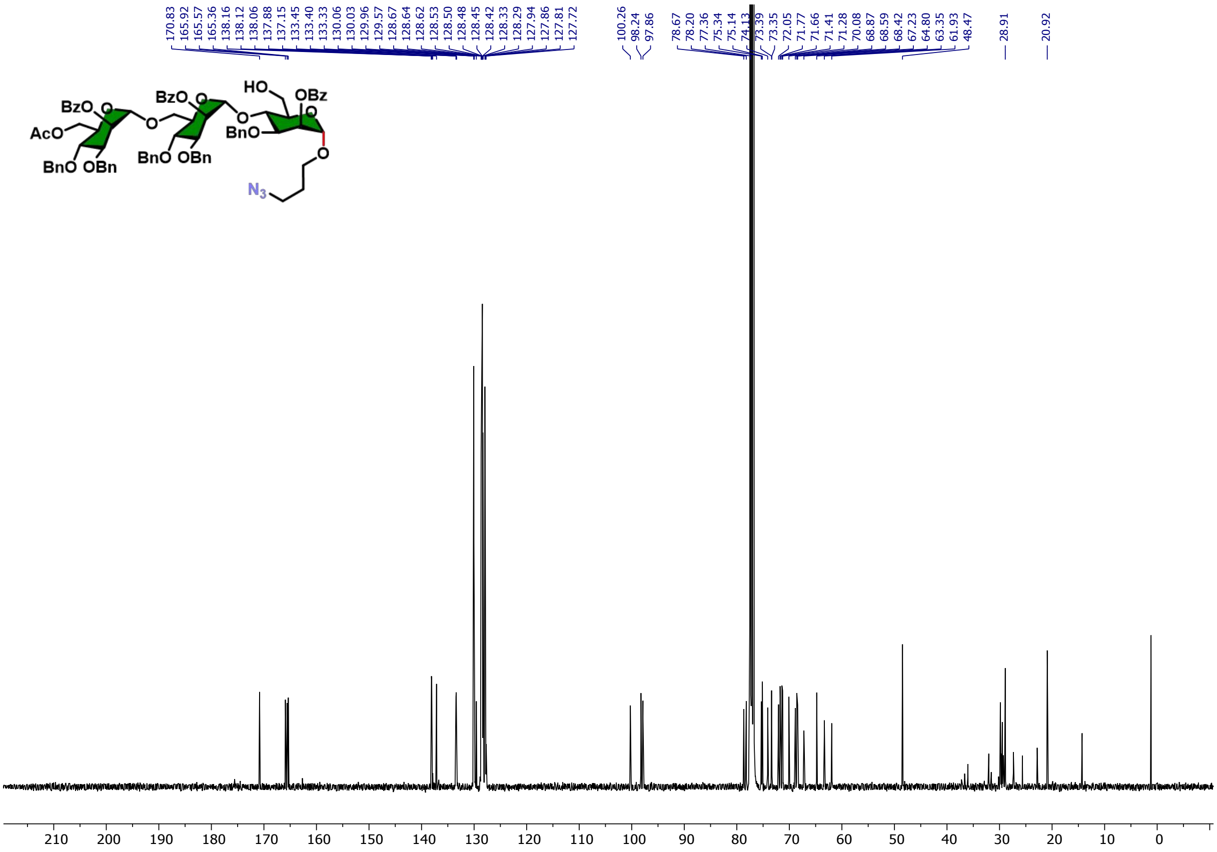
170.83
165.92
165.57
165.36
138.16
138.12
138.06
137.88
137.15
133.45
133.40
133.33
130.06
130.03
129.96
129.57
128.67
128.64
128.62
128.53
128.50
128.48
128.45
128.42
128.33
128.29
127.94
127.86
127.81
127.72
100.26
98.24
97.86
78.67
78.20
77.36
75.34
75.14
74.13
73.39
73.35
72.05
71.77
71.66
71.41
71.28
70.08
68.87
68.59
68.42
67.23
64.80
63.35
61.93
48.47
28.91
20.92
BzO
AcO
BnO OBn
BzO
BnO OBn
HO
OBz
BnO
N_3

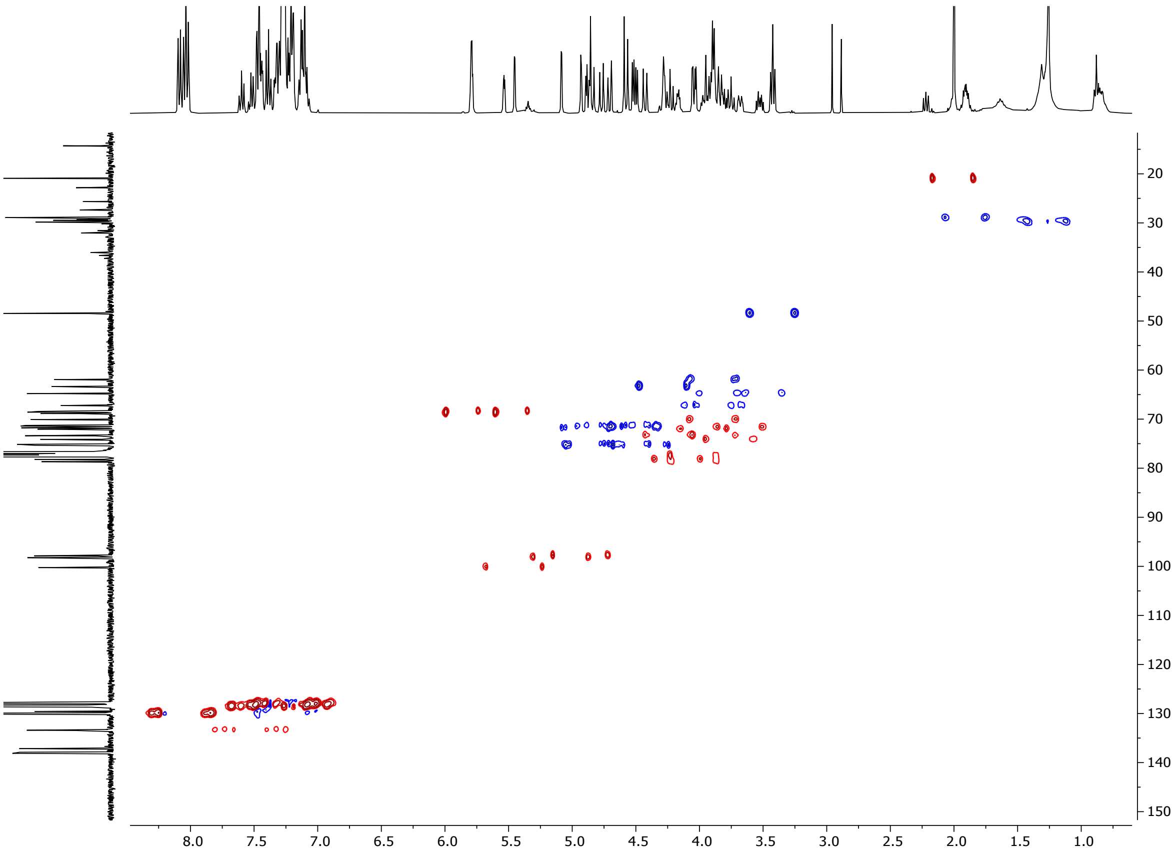

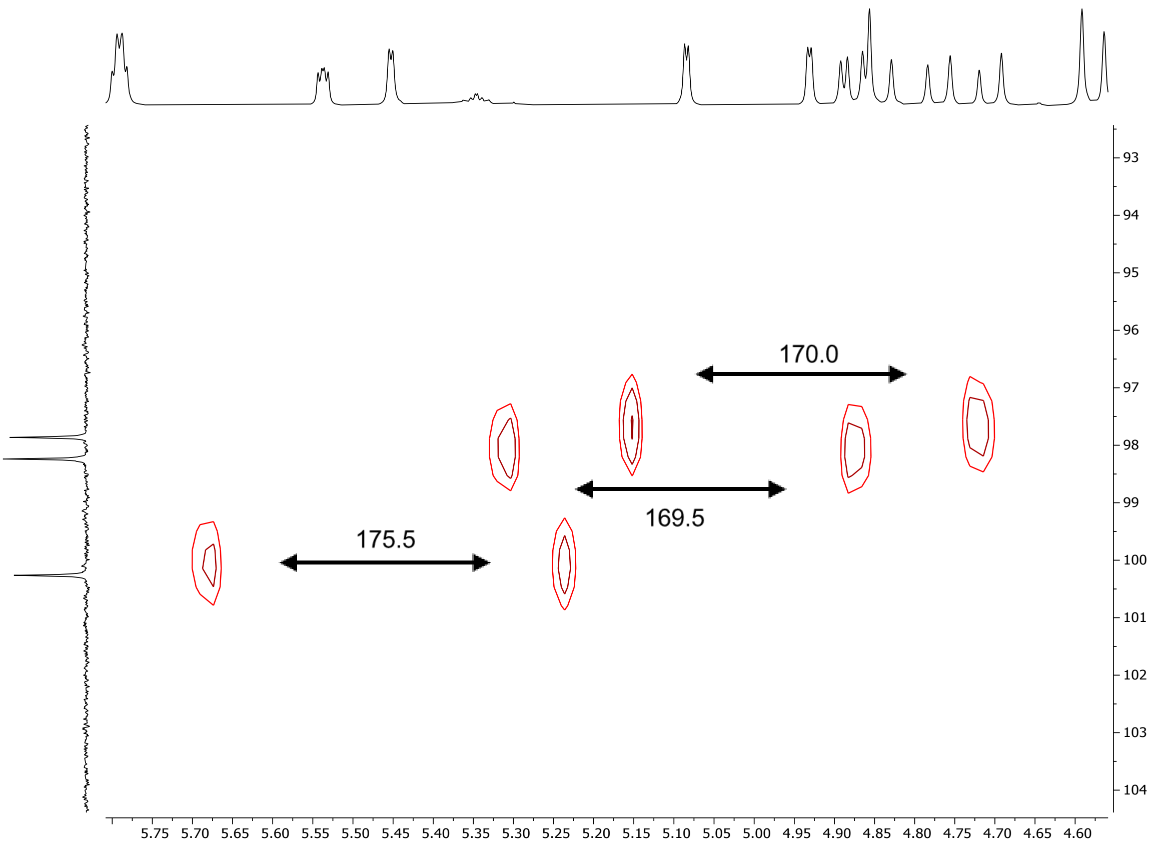

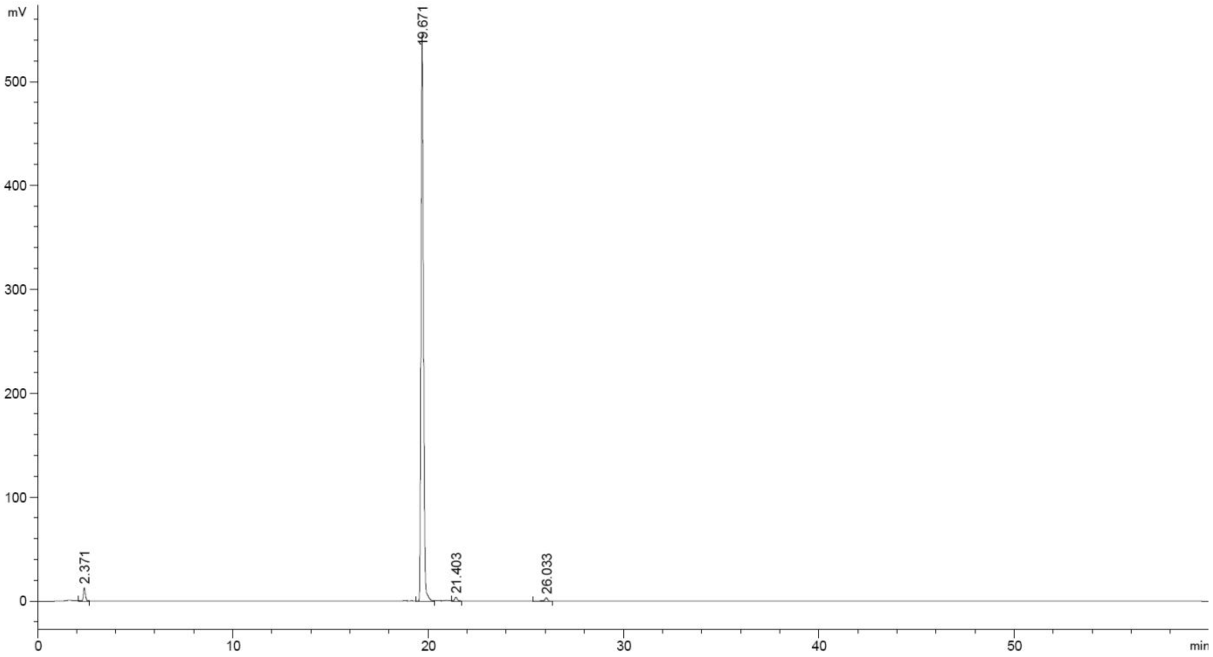

Figure 25: NP-HPLC trace of crude **22** (10-100%).

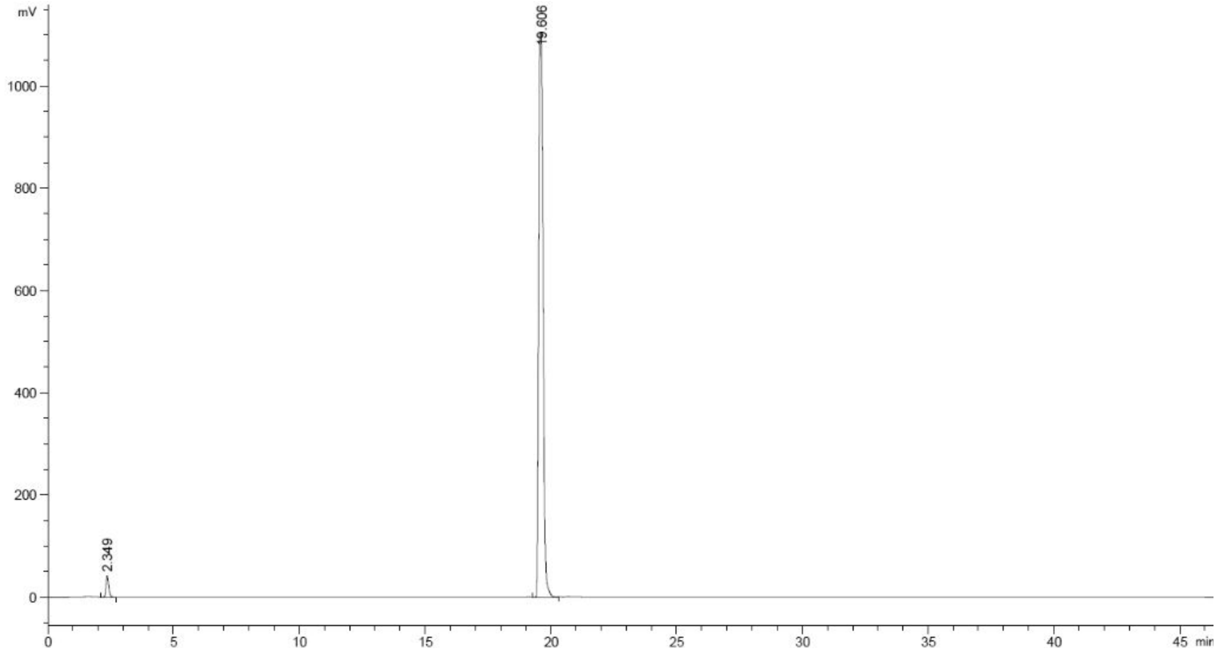

Figure 26: NP-HPLC trace of pure **22** (10-100%).

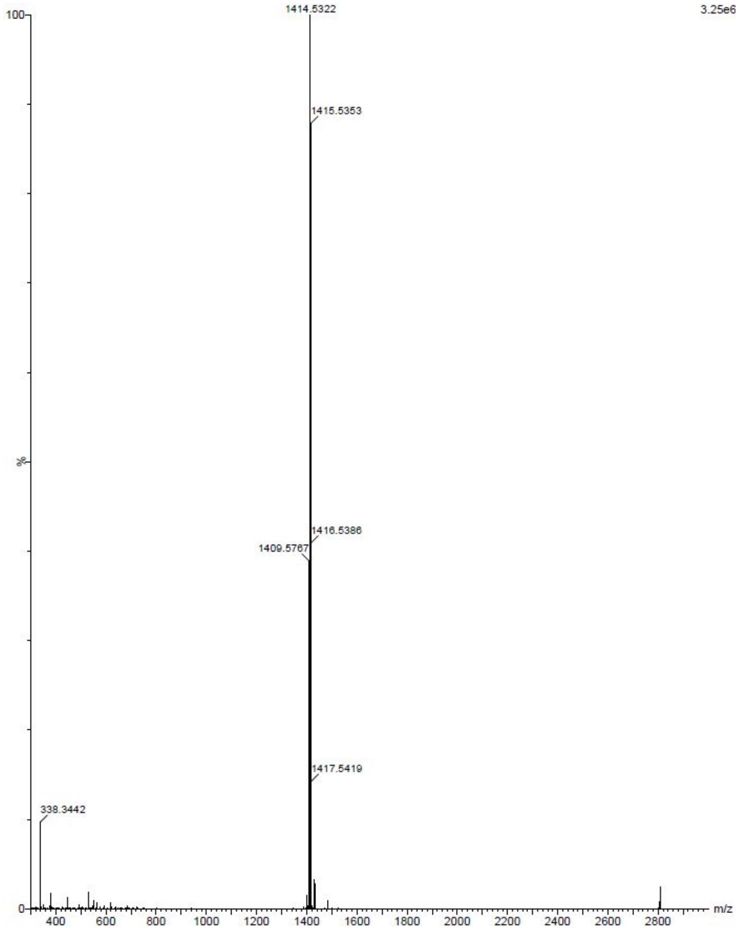

Figure 27: Q-TOF-MS-spectrum of **22**.

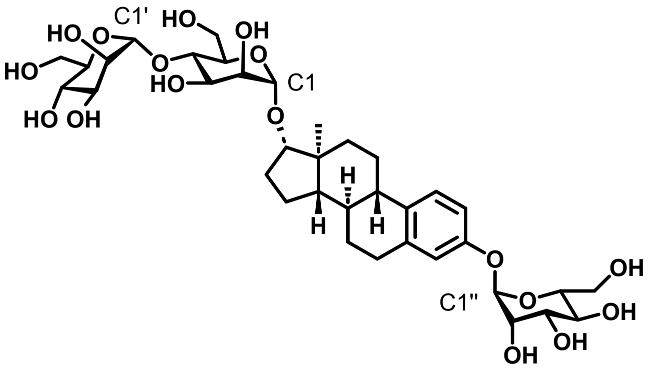

17-(α-D-Mannopyranosyl-(1→4)-α-D-mannopyranosyl)-3-(α-D-mannopyranosyl)-estradiol (23)

Step	Building Block	Modules	Notes
AGA	BB **9**, 6 eq.	**A** – first coupling	-40°C (T1) 5 min<br>-20°C (T2) 35 min
		**B** – RV Wash<br>**C** – Acidic Wash<br>**D** – Capping	
	BB **14**, 4 eq.	**E** – Fmoc Deprotection<br>**C** – Acidic Wash<br>**F** – Phosphate coupling	-30°C (T1) 10 min<br>0°C (T2) 20 min
	BB **24**, 8 eq.	**E** – Fmoc Deprotection<br>**D** – Capping<br>**C** – Acidic Wash<br>**G** – Thioglycoside Coupling	15°C (T1) 10 min<br>30°C (T2) 35 min
	BB **14**, 4 eq.	**E** – Fmoc Deprotection<br>**C** – Acidic Wash<br>**F** – Phosphate coupling	-30°C (T1) 10 min<br>0°C (T2) 20 min
Post AGA		**I** – Methanolysis	16 h
		**J** – Batch Photocleavage	16 h
		**K** – Hydrogenolysis	16 h
Purification		**L** – C18 - (0 – 40%)	R_t = 38.0 min

After a procedure including automated glycan assembly, on-resin methanolysis, photo-cleavage, hydrogenolysis, purification and lyophilization, **23** was obtained as a white powder (4.6 mg, 5.89 μmol, 28%).

R_t (C18 - 0 – 40%) = 38.0 min.

^{1}H NMR (600 MHz, D_2O/MeCN 4:1) *δ* 7.56 (d, *J* = 8.7 Hz, 1H, Ar), 7.19 (dd, *J* = 8.6, 2.7 Hz, 1H, Ar), 7.14 (d, *J* = 2.7 Hz, 1H, Ar), 5.78 (d, *J* = 1.8 Hz, 1H, *α*-H1''), 5.51 (d, *J* = 1.8 Hz, 1H, *α*-H1'), 5.10 (d, *J* = 1.8 Hz, 1H, *α*-H1), 4.34 (dd, *J* = 3.4, 1.8 Hz, 1H), 4.27 (dd, *J* = 3.3, 1.9 Hz, 1H), 4.22 (dd, *J* = 9.6, 3.4 Hz, 1H), 4.18 (dd, *J* = 9.3, 3.3 Hz, 1H), 4.14 – 4.05 (m, 5H), 4.04 – 3.95 (m, 7H), 3.93 – 3.89 (m, 3H), 3.16 – 3.07 (m, 2H), 2.60 (dd, *J* = 13.6, 3.6 Hz, 1H), 2.52 – 2.45 (m, 1H), 2.45 – 2.36 (m, 1H), 2.25 – 2.19 (m, 1H), 2.19 – 2.13 (m, 1H), 2.02 (dt, *J* = 10.6, 6.1 Hz, 1H), 1.83 – 1.75 (m, 1H), 1.70 (ddd, *J* = 17.7, 8.8, 3.1 Hz, 2H), 1.67 – 1.56 (m, 3H), 1.55 – 1.49 (m, 1H), 1.08 (s, 3H, CH_3).

^{13}C NMR (151 MHz, D_2O/MeCN 4:1) *δ* 153.8 (ArO), 138.8, 135.3, 126.9, 117.4, 114.6, 101.6 (C1'), 98.6 (C1''), 98.4 (C1), 85.2, 73.9, 73.5, 71.7, 71.5, 71.4, 70.8, 70.8, 70.6, 70.3, 66.8, 66.7, 61.2, 61.1, 60.9, 49.7, 43.9, 42.8, 38.5, 37.1, 29.4, 27.0, 26.7, 26.2, 23.0, 11.5 (CH_3).

HRMS (ESI): $C_{36}H_{54}NaO_{17}$ $[M+Na]^+$; calculated: 781.3259, found: 781.3282.

Optical rotation: $[\alpha]_D^{25}$ = +162.0° (c = 0.05, H_2O/MeCN 1:1)

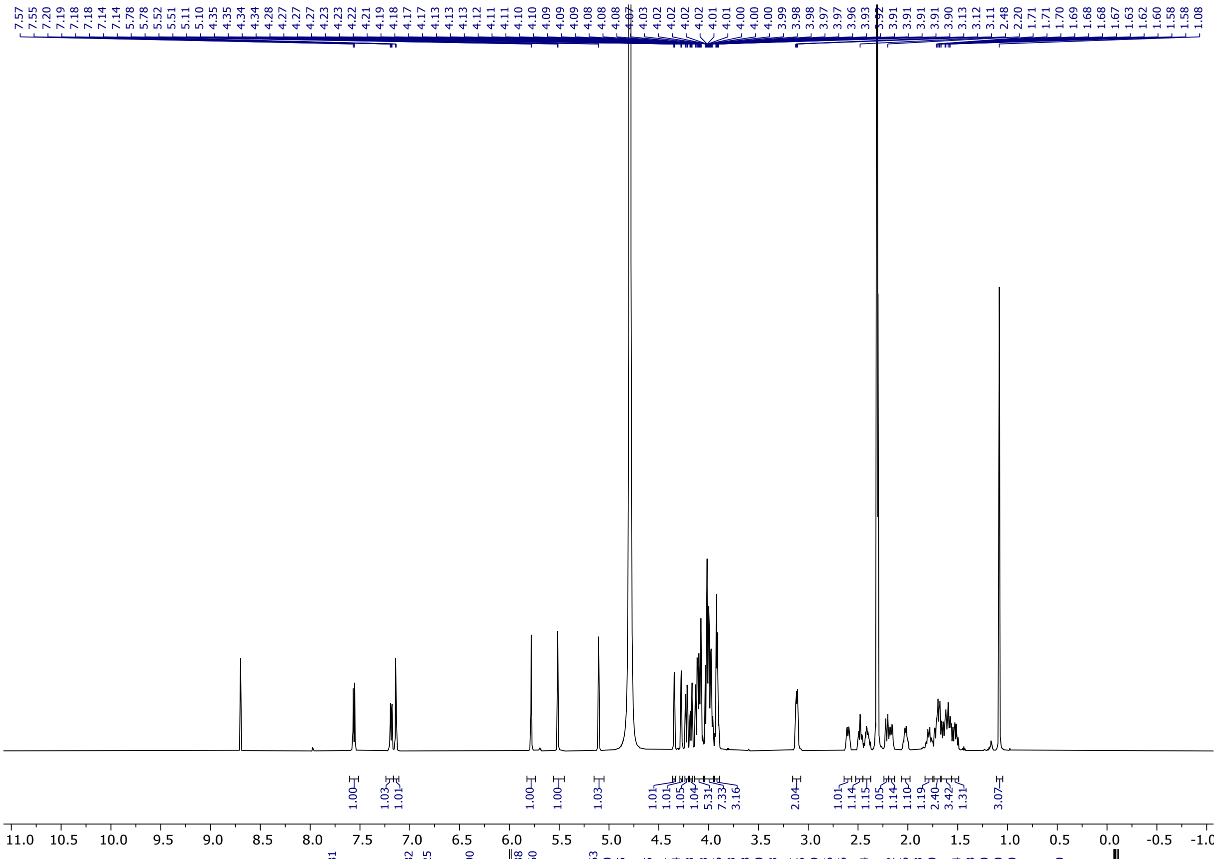
7.57 7.55 7.20 7.19 7.18 7.18 7.14 5.78 5.78 5.52 5.51 5.11 5.10 4.35 4.34 4.28 4.27 4.27 4.27 4.23 4.22 4.21 4.19 4.18 4.17 4.13 4.13 4.13 4.12 4.11 4.11 4.10 4.10 4.09 4.09 4.09 4.08 4.08 4.08 4.07 4.03 4.02 4.02 4.02 4.02 4.01 4.01 4.00 4.00 3.99 3.98 3.98 3.97 3.97 3.96 3.93 3.92 3.91 3.91 3.91 3.91 3.90 3.13 3.12 3.11 2.48 2.20 1.71 1.71 1.70 1.69 1.68 1.68 1.67 1.63 1.62 1.60 1.58 1.58 1.08
1.00 1.03 1.01 1.00 1.00 1.03 1.01 1.01 1.05 1.04 5.31 7.33 3.16 2.04 1.01 1.14 1.15 1.05 1.14 1.10 1.19 2.40 3.42 1.31 3.07
11.0 10.5 10.0 9.5 9.0 8.5 8.0 7.5 7.0 6.5 6.0 5.5 5.0 4.5 4.0 3.5 3.0 2.5 2.0 1.5 1.0 0.5 0.0 -0.5 -1.0

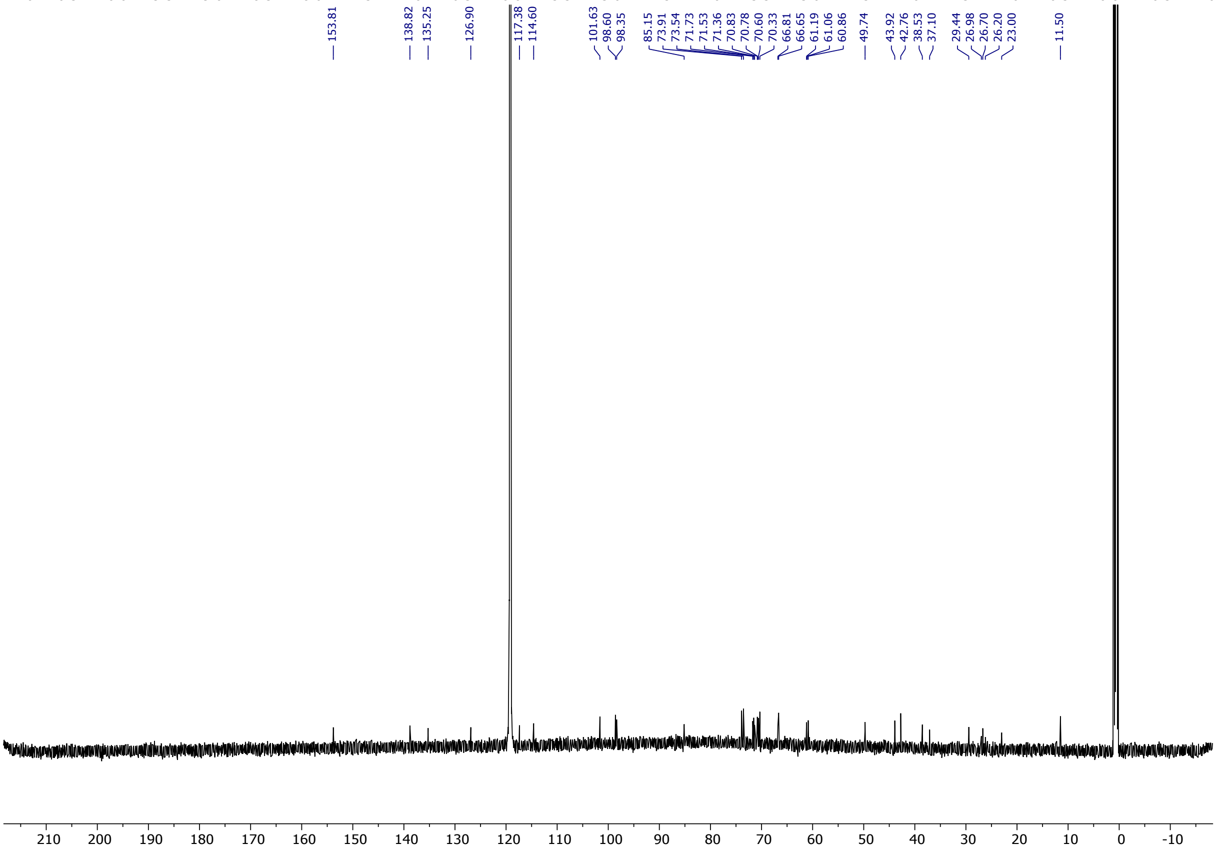
153.81 138.82 135.25 126.90 117.38 114.60 101.63 98.60 98.35 85.15 73.91 73.54 71.73 71.53 71.36 70.83 70.78 70.60 70.33 66.81 66.65 61.19 61.06 60.86 49.74 43.92 42.76 38.53 37.10 29.44 26.98 26.70 26.20 23.00 11.50
210 200 190 180 170 160 150 140 130 120 110 100 90 80 70 60 50 40 30 20 10 0 -10

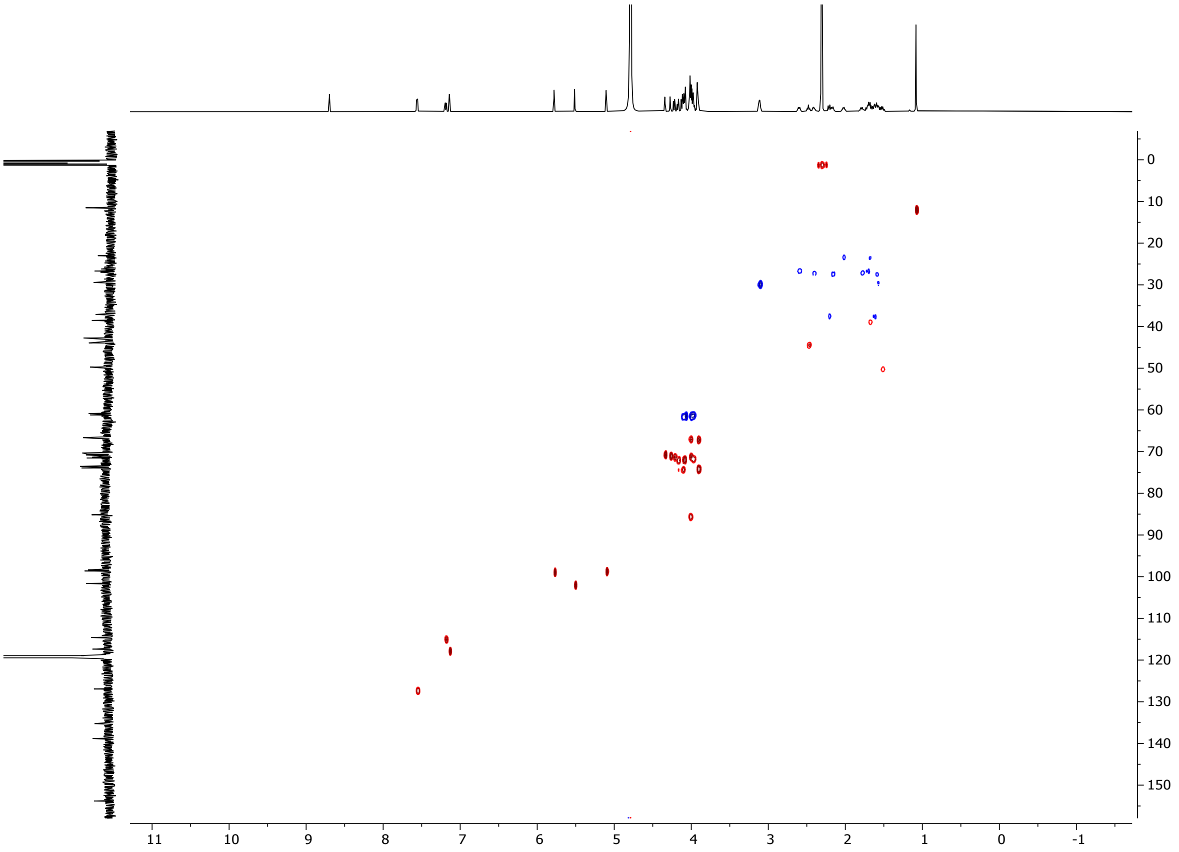

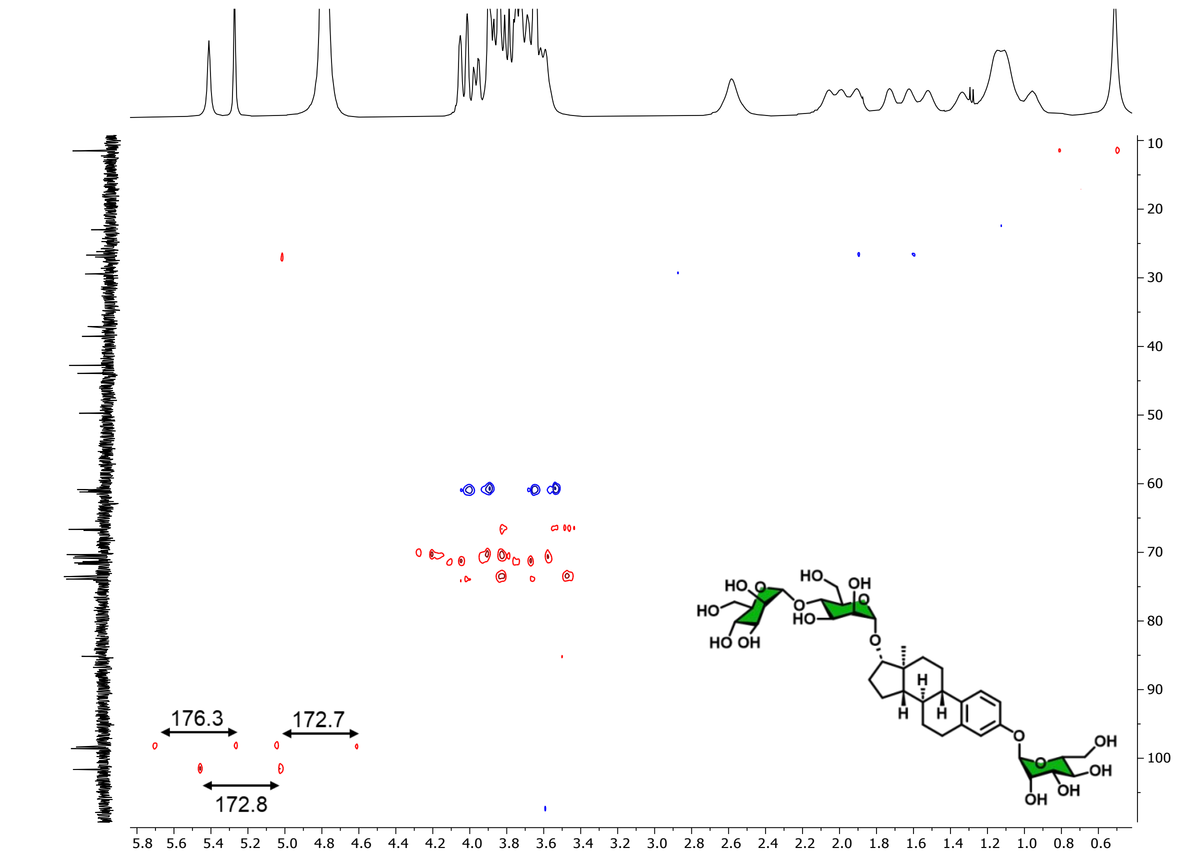
176.3
172.7
172.8
HO
OH
O

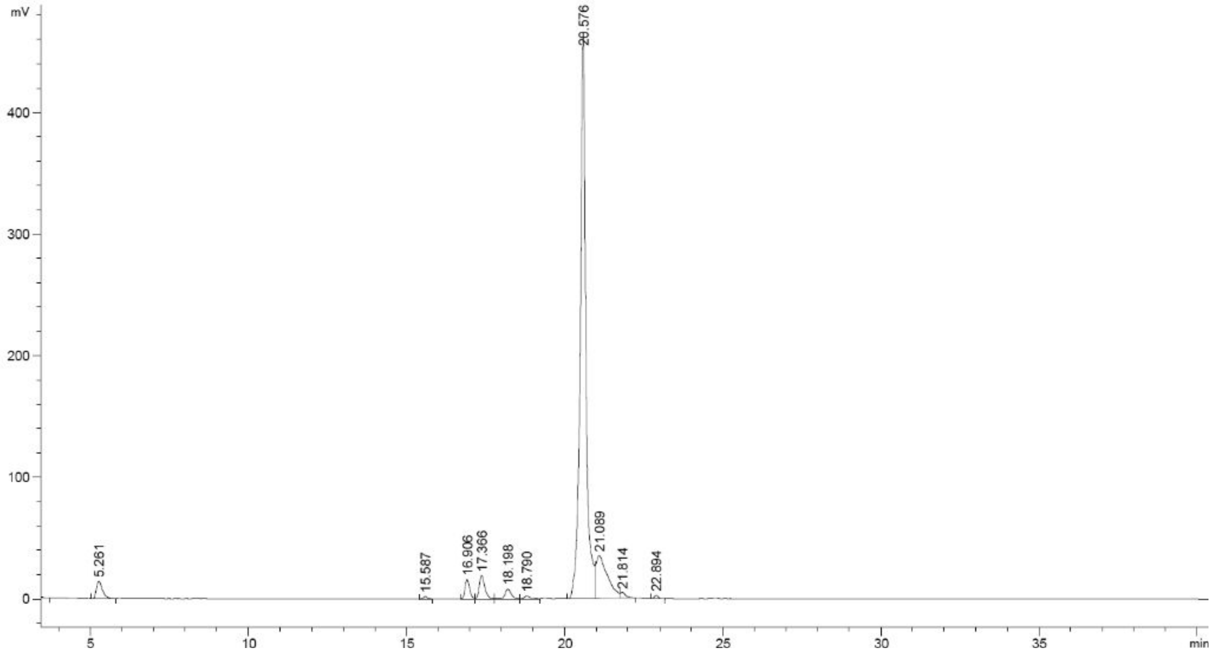

Figure 28: NP-HPLC trace of crude fully protected **24** (10-100%).

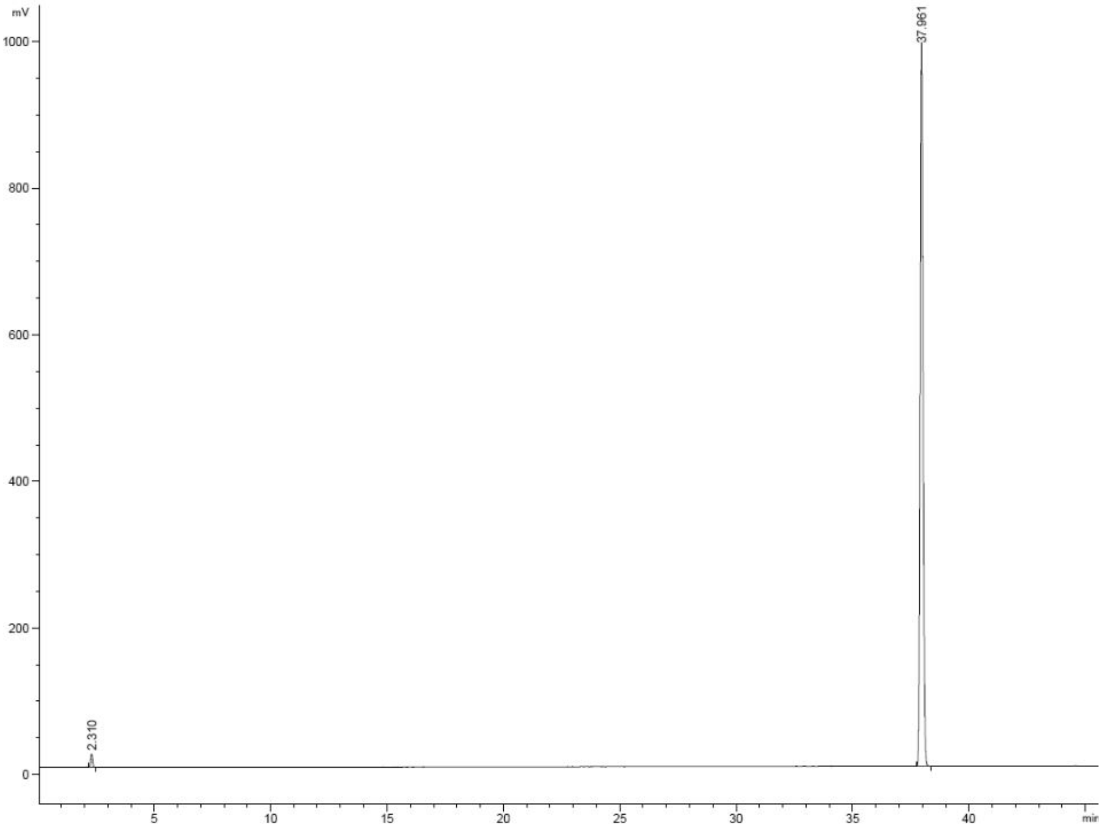

Figure 29: RP-HPLC trace of pure **23** (C_{18}, 0-40%).

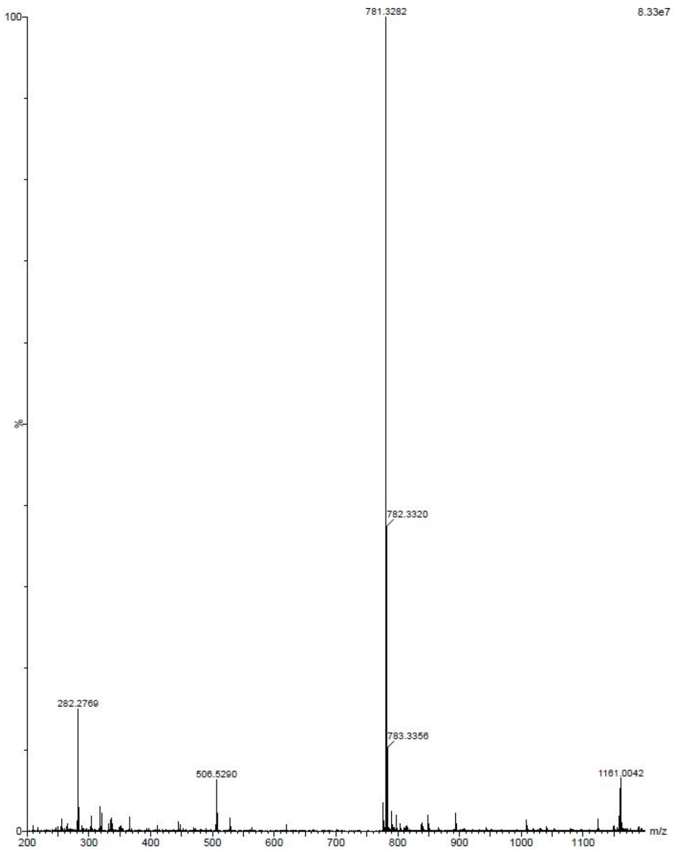

Figure 30: Q-TOF MS-spectrum of **23**.

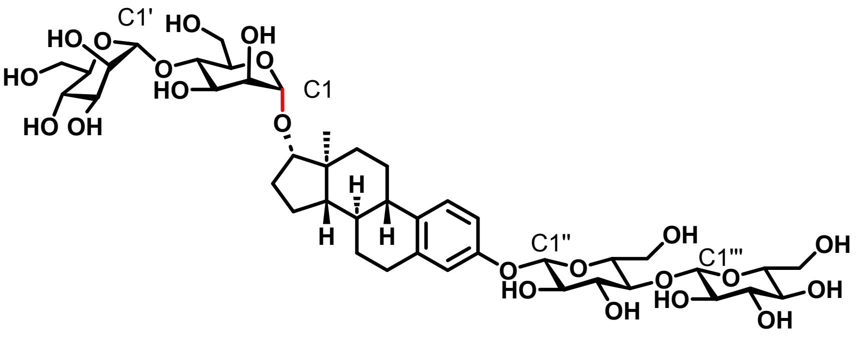

17-(*α*-D-Mannopyranosyl-(1→4)-*α*-D-mannopyranosyl)-3-(*β*-D-Glucopyranosyl-(1→4)-*β*-D-Glucopyranosyl)-estradiol (25)

Step	Building Block	Modules	Notes
AGA	BB **9**, 6 eq.	**A** – First coupling	-40°C (T1) 5 min<br>-20°C (T2) 35 min
		**B** – RV Wash	
		**C** – Acidic Wash	
		**D** – Capping	
		**E** – Fmoc Deprotection	
		**C** – Acidic Wash	
	BB **14**, 4 eq.	**F** – Phosphate coupling	-30°C (T1) 10 min<br>0°C (T2) 20 min
		**E** – Fmoc Deprotection	
		**D** – Capping	
		**C** – Acidic Wash	
	BB **24**, 8 eq.	**G** – Thioglycoside Coupling 2	15°C (T1) 10 min<br>30°C (T2) 35 min
		**E** – Fmoc Deprotection	
	**2x**	**C** – Acidic Wash	
	BB **26**, 6 eq.	**G** – Thioglycoside Coupling	-20°C (T1) 20 min<br>0°C (T2) 20 min
Post AGA		**I** – Methanolysis	16 h
		**J** – Batch Photocleavage	16 h
		**K** – Hydrogenolysis	16 h
Purification		**L** – C5-RP-(10-50%)	R_t = 24.7 min

After a procedure including automated glycan assembly, on-resin methanolysis, photo-cleavage, hydrogenolysis, purification and lyophilization, **25** was obtained as a white powder (2.3 mg, 2.4 µmol, 13%).

R_t (RP-C5 - 10 – 60%) = 22.6 min.

^{1}H NMR (400 MHz, D_2O/MeCN 1:1) *δ* 7.45 (d, *J* = 8.7 Hz, 1H, Ar), 7.05 (dd, *J* = 8.5, 2.8 Hz, 1H, Ar), 6.99 (d, *J* = 2.7 Hz, 1H, Ar), 5.38 (d, *J* = 1.8 Hz, 1H, *α*-H1'), 5.16 (d, *J* = 7.9 Hz, 1H, *β*-H1''), 4.99 (d, *J* = 1.8 Hz, 1H, *α*-H1), 4.64 (d, *J* = 7.9 Hz, 1H, *β*-H1'''), 4.16 (dd, *J* = 3.3, 1.8 Hz, 1H), 4.10 – 3.75 (m, 19H), 3.68 (t, *J* = 8.0 Hz, 1H), 3.65 – 3.58 (m, 2H), 3.56 – 3.49 (m, 1H), 3.43 (dd, *J* = 9.4, 7.9 Hz, 1H), 3.06 – 2.93 (m, 2H), 2.48 (d, *J* = 12.7 Hz, 1H), 2.40 – 2.32 (m, 1H), 2.32 – 2.23 (m, 1H), 2.12 – 2.02 (m, 1H), 1.90 (d, *J* = 8.6 Hz, 1H), 1.73 – 1.33 (m, 8H), 0.96 (s, 3H, CH_3).

^{13}C NMR (taken from HSQC, D_2O/MeCN 1:1) *δ* 127.0 (Ar-CH), 117.0 (Ar-CH), 114.4 (Ar-CH), 103.0 (C1'), 101.6 (C1''), 100.6 (C1), 98.4 (C1'''), 98.4, 85.2, 79.1, 76.2, 74.9, 74.1, 73.9, 73.5, 73.1, 71.9, 71.6, 71.5, 70.9, 70.7, 69.8, 68.8, 61.4, 61.0, 60.6, 60.2, 49.9, 44.1, 42.7, 38.7, 37.2, 29.5, 29.2, 27.2, 26.9, 26.7, 29.2, 23.1, 11.6 (CH_3).

HRMS (ESI): $C_{42}H_{64}NaO_{22}$ $[M+Na]^+$; calculated: 943.3787, found: 943.3864.

Optical rotation: $[\alpha]_D^{25}$ = +93.0° (c = 0.1, H_2O/MeCN 1:1)

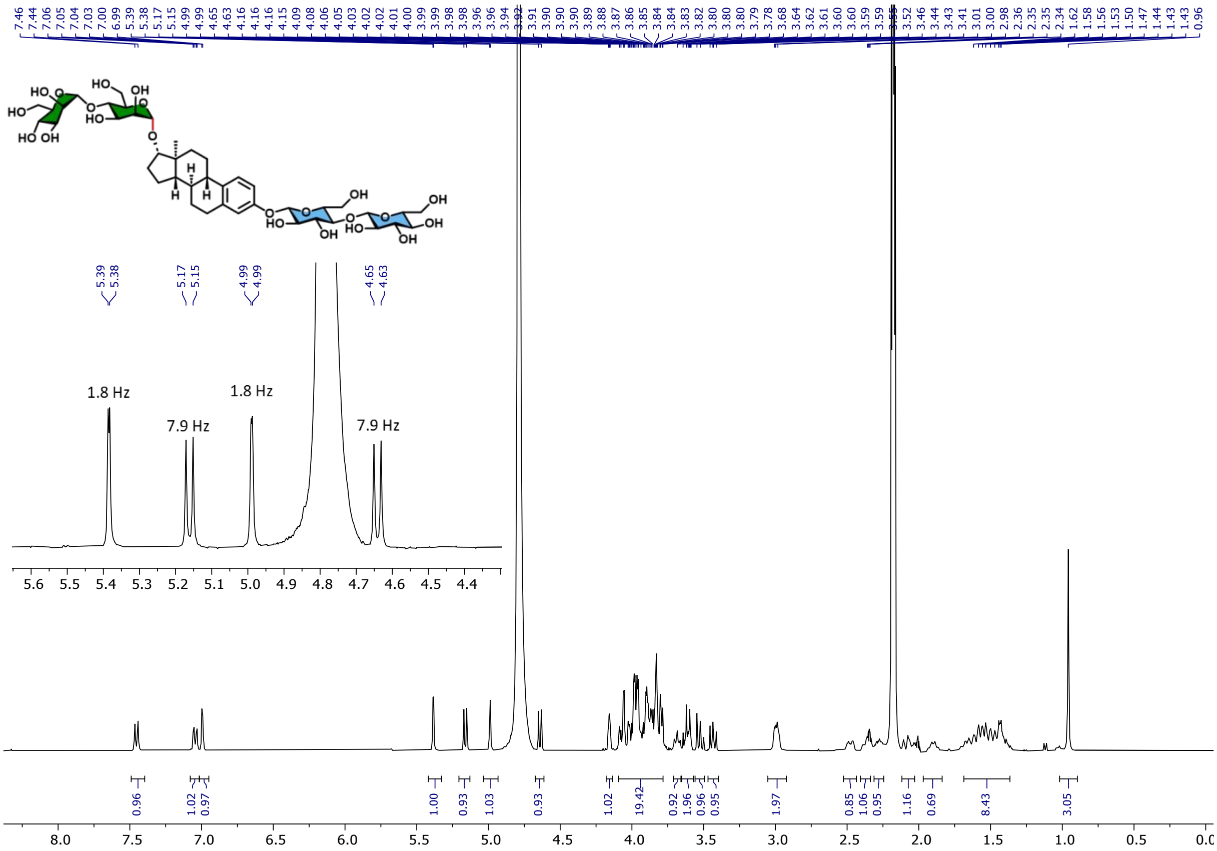

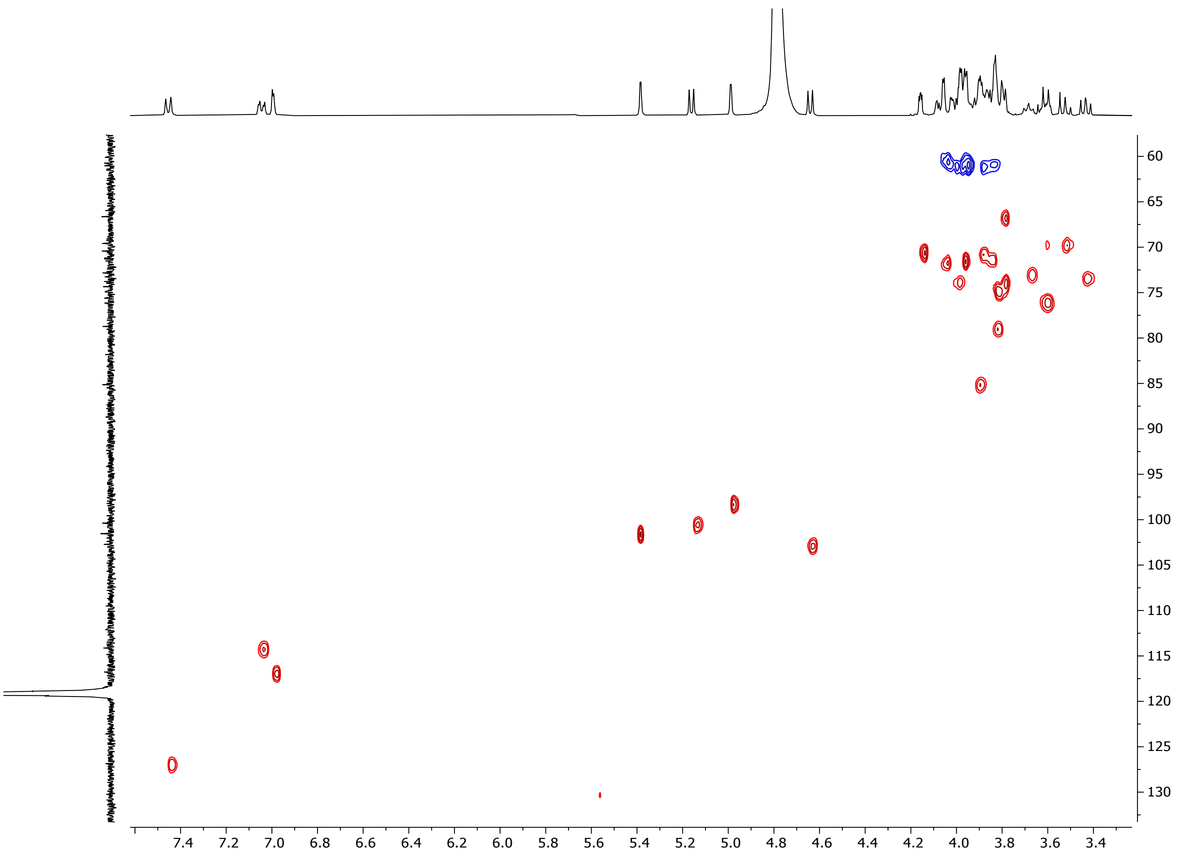

7.4 7.2 7.0 6.8 6.6 6.4 6.2 6.0 5.8 5.6 5.4 5.2 5.0 4.8 4.6 4.4 4.2 4.0 3.8 3.6 3.4
60 65 70 75 80 85 90 95 100 105 110 115 120 125 130

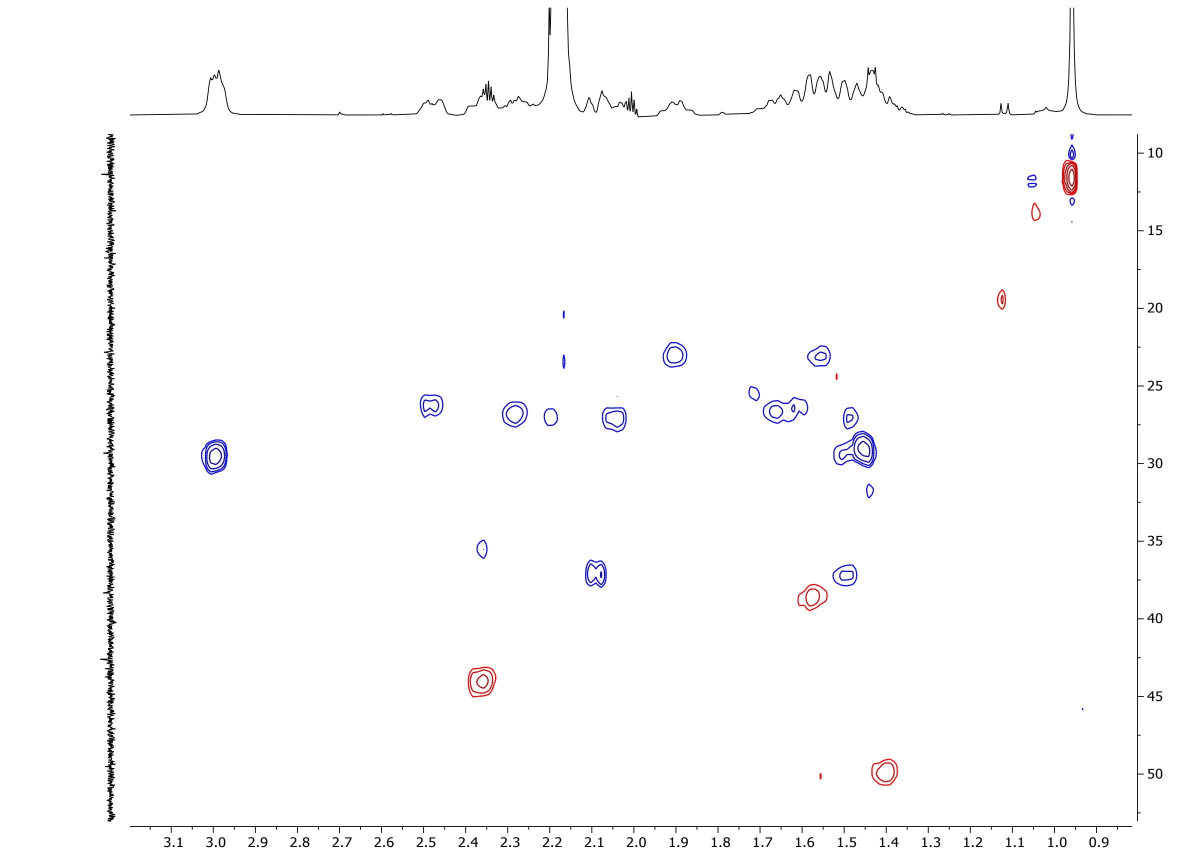

3.1 3.0 2.9 2.8 2.7 2.6 2.5 2.4 2.3 2.2 2.1 2.0 1.9 1.8 1.7 1.6 1.5 1.4 1.3 1.2 1.1 1.0 0.9
10 15 20 25 30 35 40 45 50

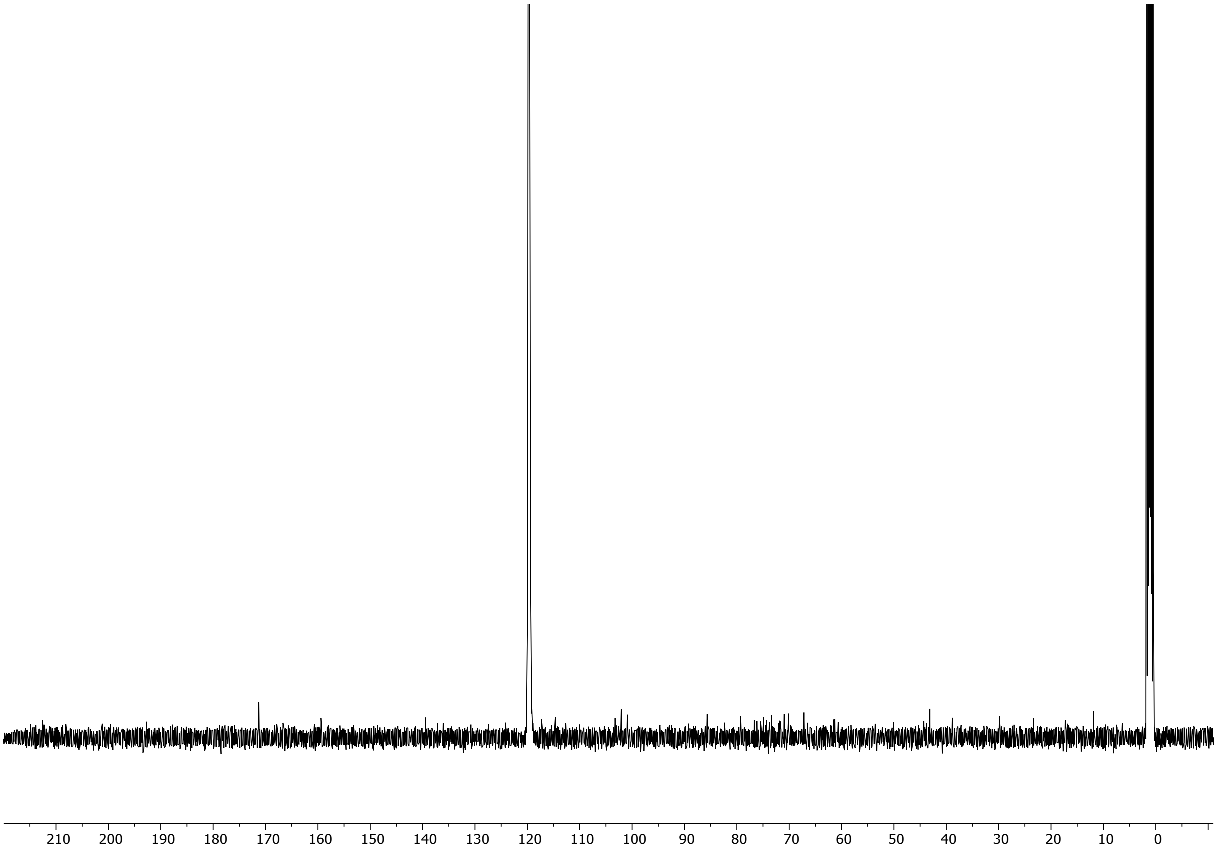

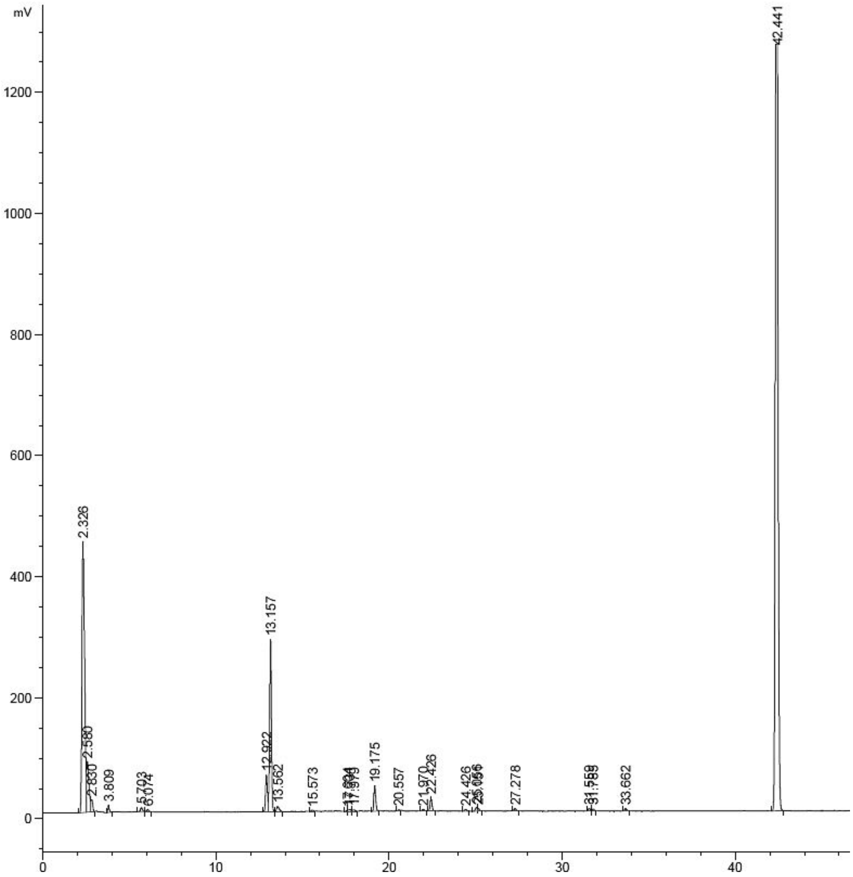

Figure 31: RP-HPLC trace of crude **25** (C_{18}; 20-90%).

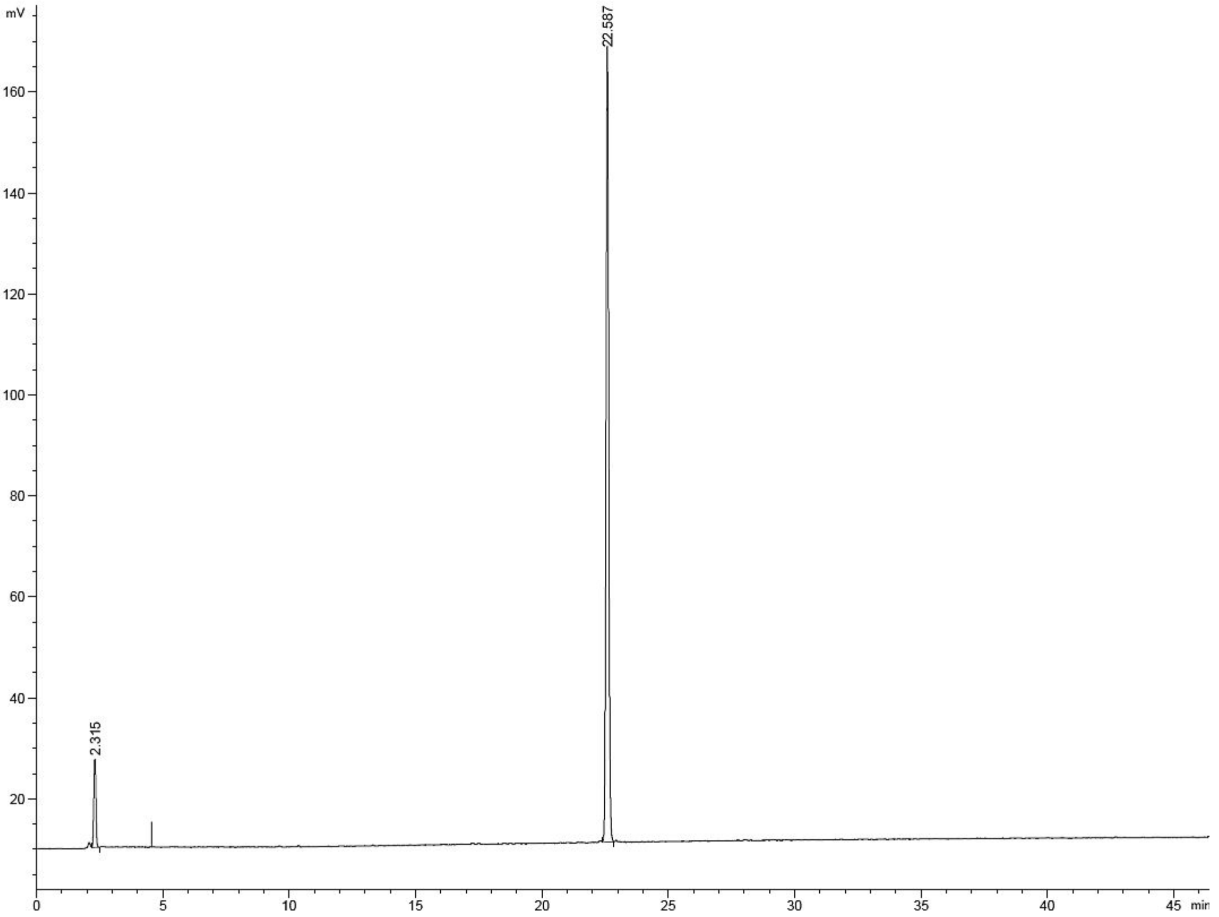

Figure 32: RP-HPLC trace of pure **25** (C_5; 10-60%).

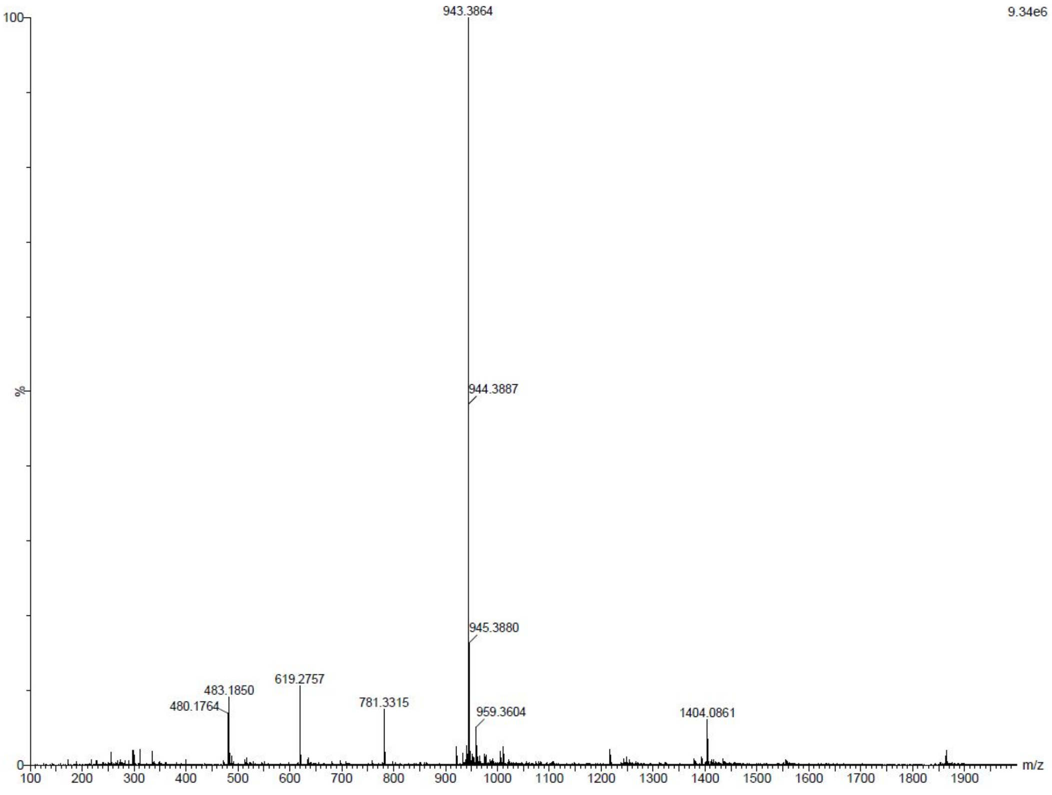

Figure 33: Q-TOF MS-spectrum of **25**.

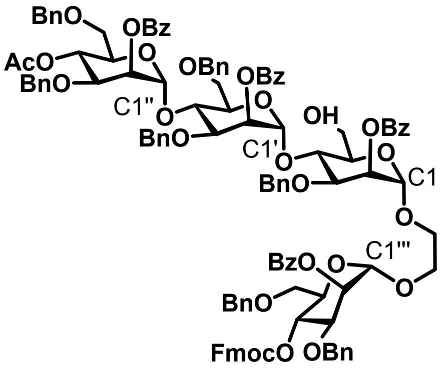

1-(4-O-Acetyl-2-O-benzoyl-3,6-di-O-benzyl-*α*-D-mannopyranosyl-(1→4)-2-O-benzoyl-3,6-di-O-benzyl-*α*-D-mannopyranosyl-(1→4)-2-O-benzoyl-3,6-di-O-benzyl-*α*-D-mannopyranosyl)-2-(2-O-benzoyl-3,6-di-O-benzyl-4-O-(9-fluorenylmethoxycarbonyl)-*α*-D-mannopyranosyl)-ethane diol (27)

Step	Building Block	Modules	Notes
AGA	BB **9**, 6 eq.	**A** – first coupling	-40°C (T1) 5 min<br>-20°C (T2) 35 min
		**B** – RV Wash<br>**C** – Acidic Wash<br>**D** – Capping	
		**E** – Fmoc Deprotection<br>**C** – Acidic Wash	
	BB **10**, 3.5 eq.	**F** – Phosphate coupling	-30°C (T1) 10 min<br>0°C (T2) 20 min
		**E** – Fmoc Deprotection<br>**D** – Capping<br>**C** – Acidic Wash	
	BB **8**, 10 eq.	**G** – Thioglycoside Coupling	20°C (T1) 10 min<br>35°C (T2) 35 min
		**E** – Fmoc Deprotection<br>**C** – Acidic Wash	
	BB **10**, 3.5 eq.	**F** – Phosphate coupling	-30°C (T1) 10 min<br>0°C (T2) 20 min
**Post AGA**		**J** – Batch Photocleavage	16 h
**Purification**		**L** – NP - (10-100)	R_t = 22.3 min

After a procedure including automated glycan assembly, photo-cleavage, purification and lyophilization, **27** was obtained as a translucent resin (6.4 mg, 3.13 µmol, 15%).

R_t (NP - 10-100) = 22.3 min.

^{1}H NMR (600 MHz, $CDCl_3$) *δ* 8.06 (dd, *J* = 6.7, 1.5 Hz, 4H), 7.97 – 7.89 (m, 4H), 7.76 (d, *J* = 7.6 Hz, 2H), 7.57 (dd, *J* = 7.6, 3.8 Hz, 2H), 7.55 – 7.46 (m, 4H), 7.42 – 7.36 (m, 6H), 7.35 – 7.19 (m, 33H), 7.18 – 7.11 (m, 3H), 7.10 – 6.98 (m, 6H), 5.81 – 5.76 (m, 1H), 5.72 (ddd, *J* = 5.5, 3.3, 1.9 Hz, 2H), 5.67 – 5.62 (m, 1H), 5.56 (d, *J* = 1.9 Hz, 1H, *α*-H1''), 5.49 (d, *J* = 2.0 Hz, 1H, *α*-H1'), 5.42 (t, *J* = 9.9 Hz, 1H, H4'''), 5.35 (t, *J* = 9.8 Hz, 1H, H4''), 5.15 (d, *J* = 1.9 Hz, 1H), 5.08 (d, *J* = 1.9 Hz, 1H, *α*-H1'''), 4.79 (d, *J* = 5.9 Hz, 1H), 4.78 – 4.75 (m, 2H), 4.72 (d, *J* = 12.1 Hz, 1H), 4.64 – 4.55 (m, 5H), 4.50 (d, *J* = 11.7 Hz, 1H), 4.46 (d, *J* = 10.4 Hz, 1H), 4.40 (dd, *J* = 12.1, 3.4 Hz, 2H), 4.38 – 4.31 (m, 3H), 4.21 – 4.17 (m, 1H), 4.17 – 4.10 (m, 3H), 4.08 (dd, *J* = 10.0, 3.9 Hz, 1H), 3.99 (ddd, *J* = 25.2, 9.4, 2.5 Hz, 3H), 3.94 – 3.69 (m, 12H), 3.44 (d, *J* = 3.4 Hz, 2H, CH_2-Fmoc), 2.87 (t, *J* = 6.6 Hz, 1H, OH), 1.94 (s, 3H, CH_3-Ac).

^{13}C NMR (151 MHz, $CDCl_3$) *δ* 169.8 (CO-Ac), 166.0 (CO-Bz), 165.9 (CO-Bz), 165.3 (CO-Bz), 165.2 (CO-Bz), 154.7 (CO_3-Fmoc), 143.5, 143.4, 141.4, 138.2, 138.2, 138.0, 137.8, 137.4, 137.2, 133.4, 133.4, 133.2, 130.1, 130.1, 130.0, 129.8, 129.7, 129.7, 129.6, 128.6, 128.6, 128.6, 128.5, 128.5, 128.5, 128.4, 128.4, 128.4, 128.3, 128.0, 127.89, 127.8, 127.8, 127.7, 127.7, 127.7, 127.6, 127.3, 127.3, 125.3, 125.3, 120.2, 120.2, 99.6 (C1''), 99.4 (C1'), 98.4 (C1), 98.4 (C1'''), 78.2, 77.7, 75.2, 75.1, 73.9, 73.7, 73.7, 72.8, 72.8, 72.4, 72.2, 71.8, 71.7, 71.4, 71.4, 71.3, 71.3, 70.2, 70.2, 70.0, 69.5, 69.45, 69.0, 68.7, 68.5, 68.2, 67.1, 67.0, 61.7, 46.9 (CH-Fmoc), 21.1 (CH_3-Ac).

HRMS (ESI): $C_{120}H_{116}NaO_{29}$ $[M+Na]^+$; calculated: 2043.7500, found: 2043.7753.

Optical rotation: $[\alpha]_D^{25}$ = -18.2° (c = 0.5, $CHCl_3$)

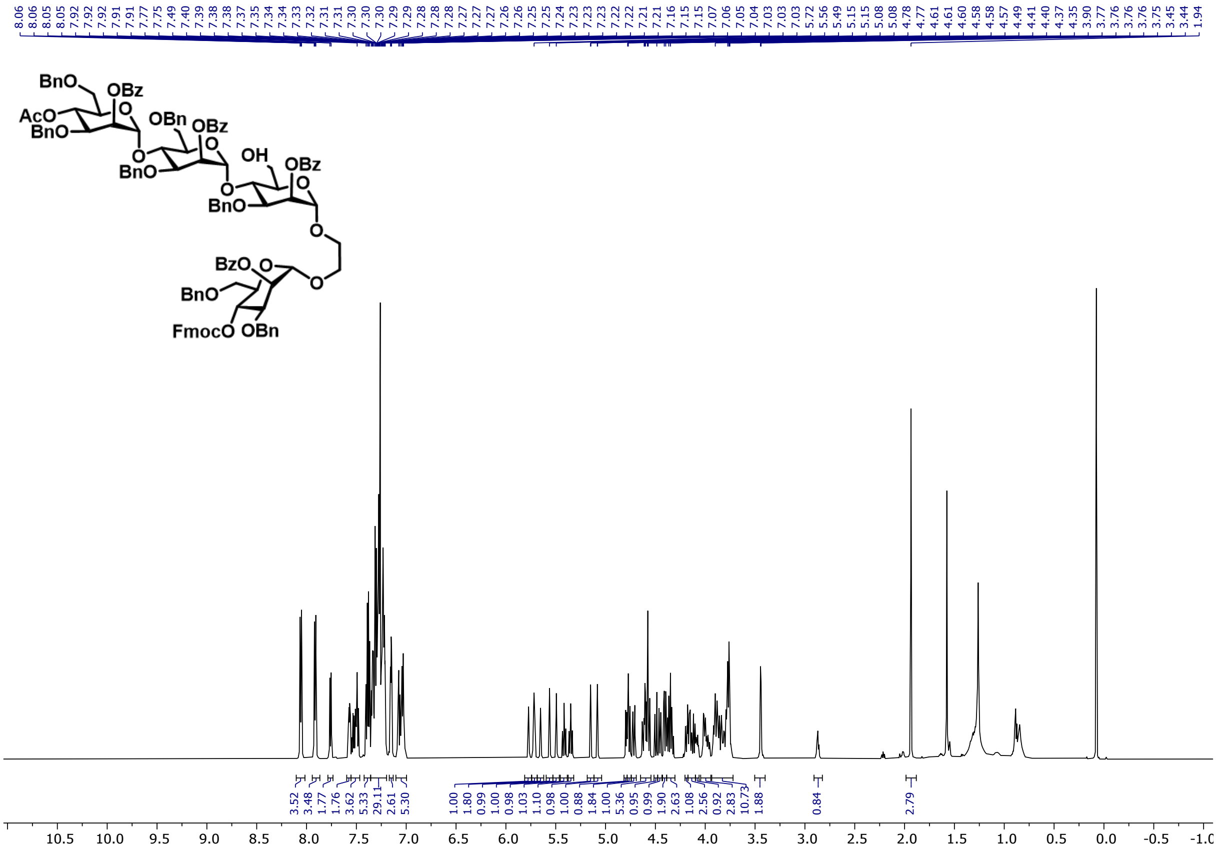
8.06 8.06 8.05 8.05 7.92 7.92 7.92 7.91 7.91 7.77 7.75 7.49 7.40 7.39 7.38 7.38 7.37 7.35 7.34 7.34 7.33 7.32 7.31 7.31 7.30 7.30 7.30 7.29 7.29 7.28 7.28 7.28 7.27 7.27 7.27 7.26 7.26 7.25 7.25 7.24 7.23 7.23 7.22 7.22 7.21 7.21 7.16 7.15 7.15 7.07 7.06 7.05 7.04 7.03 7.03 7.03 5.72 5.56 5.49 5.15 5.15 5.08 4.78 4.77 4.61 4.61 4.60 4.58 4.58 4.57 4.49 4.41 4.40 4.37 4.35 3.90 3.77 3.76 3.76 3.76 3.75 3.45 3.44 1.94
BnO
OBz
AcO
BnO
O
OBn
OBz
O
BnO
O
OH
OBz
O
BnO
O
O
BzO
O
BnO
FmocO
OBn
3.52 3.48 1.77 1.76 3.62 5.33 29.11 2.61 5.30 1.00 1.80 0.99 1.00 0.98 1.03 1.10 0.98 1.00 0.88 1.84 1.00 5.36 0.95 0.99 1.90 2.63 1.08 2.56 0.92 2.83 10.73 1.88 0.84 2.79
10.5 10.0 9.5 9.0 8.5 8.0 7.5 7.0 6.5 6.0 5.5 5.0 4.5 4.0 3.5 3.0 2.5 2.0 1.5 1.0 0.5 0.0 -0.5 -1.0

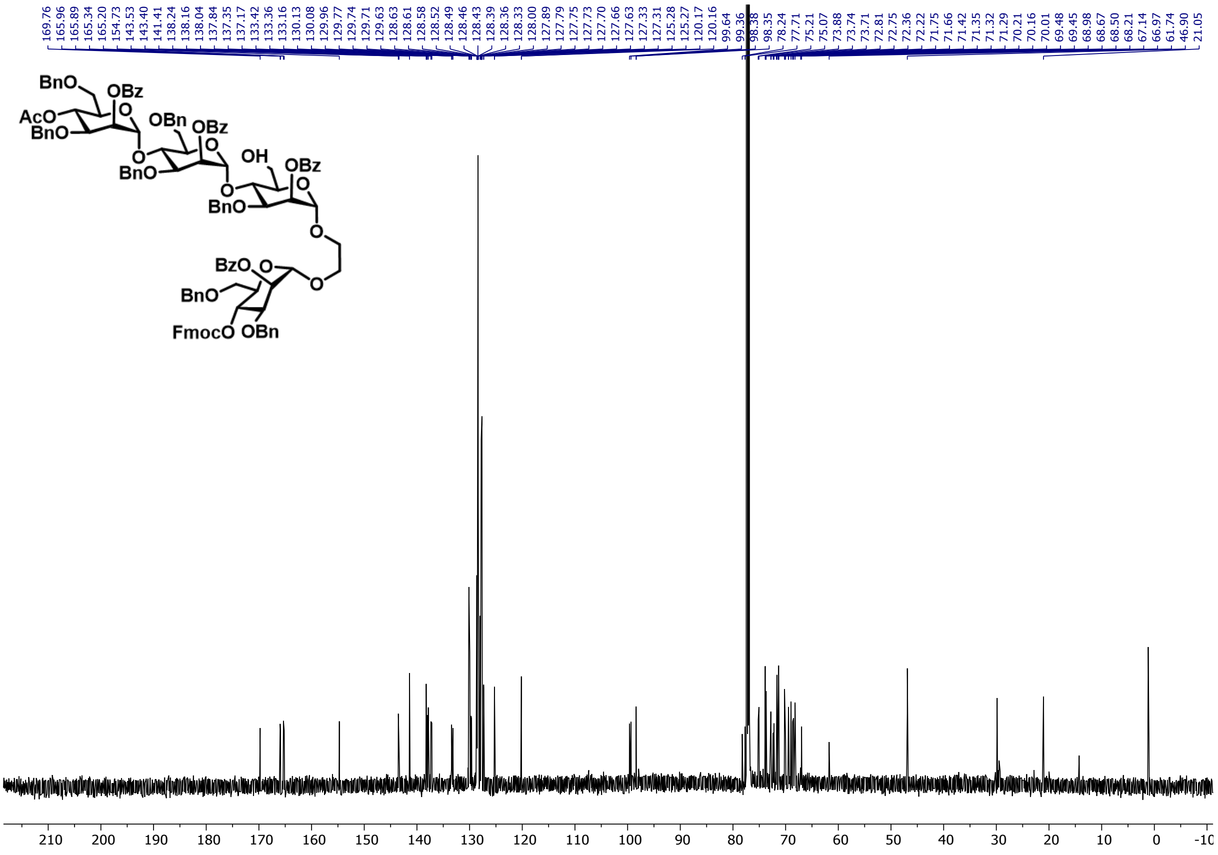
169.76 165.96 165.89 165.34 165.20 154.73 143.53 143.40 141.41 138.24 138.16 138.04 137.84 137.35 137.17 133.42 133.36 133.16 130.13 130.08 129.96 129.77 129.74 129.71 129.63 128.63 128.61 128.58 128.52 128.49 128.46 128.43 128.39 128.36 128.33 128.00 127.89 127.79 127.75 127.73 127.70 127.66 127.63 127.33 127.31 125.28 125.27 120.17 120.16 99.64 99.36 98.38 98.35 78.24 77.71 75.21 75.07 73.88 73.74 73.71 72.81 72.75 72.36 72.22 71.75 71.66 71.42 71.35 71.32 71.29 70.21 70.16 70.01 69.48 69.45 68.98 68.67 68.50 68.21 67.14 66.97 61.74 46.90 21.05
BnO
OBz
AcO
BnO
O
OBn
OBz
O
BnO
O
OH
OBz
O
BnO
O
O
BzO
O
BnO
FmocO
OBn
210 200 190 180 170 160 150 140 130 120 110 100 90 80 70 60 50 40 30 20 10 0 -10

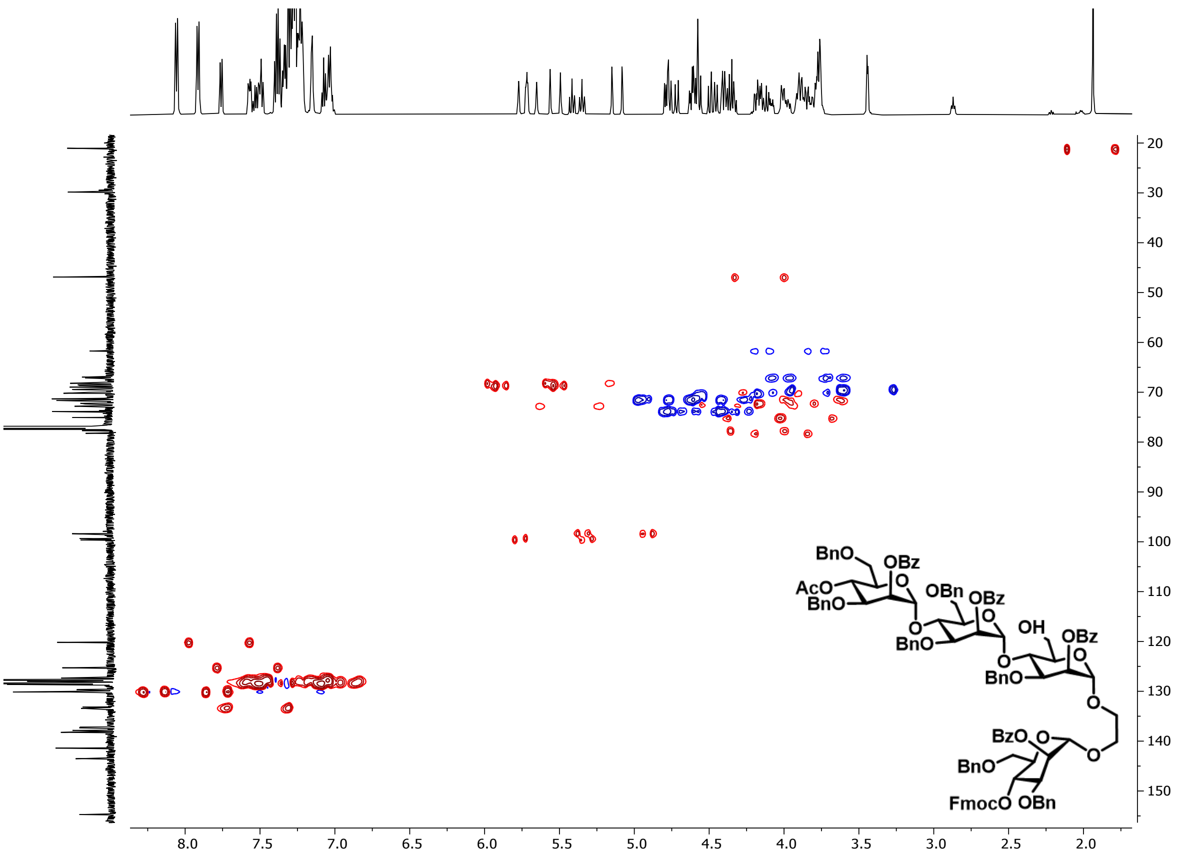
BnO
OBz
AcO
BnO
OBn
OBz
BnO
OH
OBz
BnO
BzO
BnO
FmocO
OBn

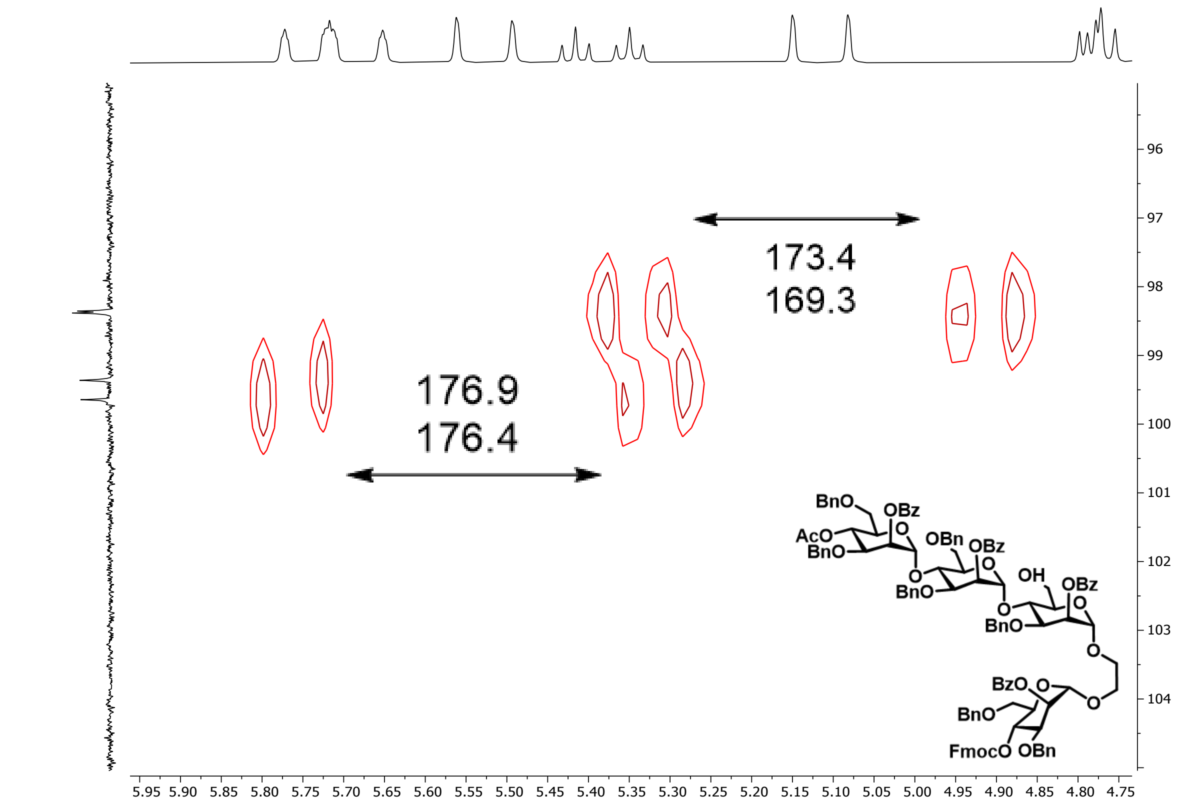
173.4
169.3
176.9
176.4
BnO
OBz
AcO
BnO
OBn
OBz
BnO
OH
OBz
BnO
BzO
BnO
FmocO
OBn

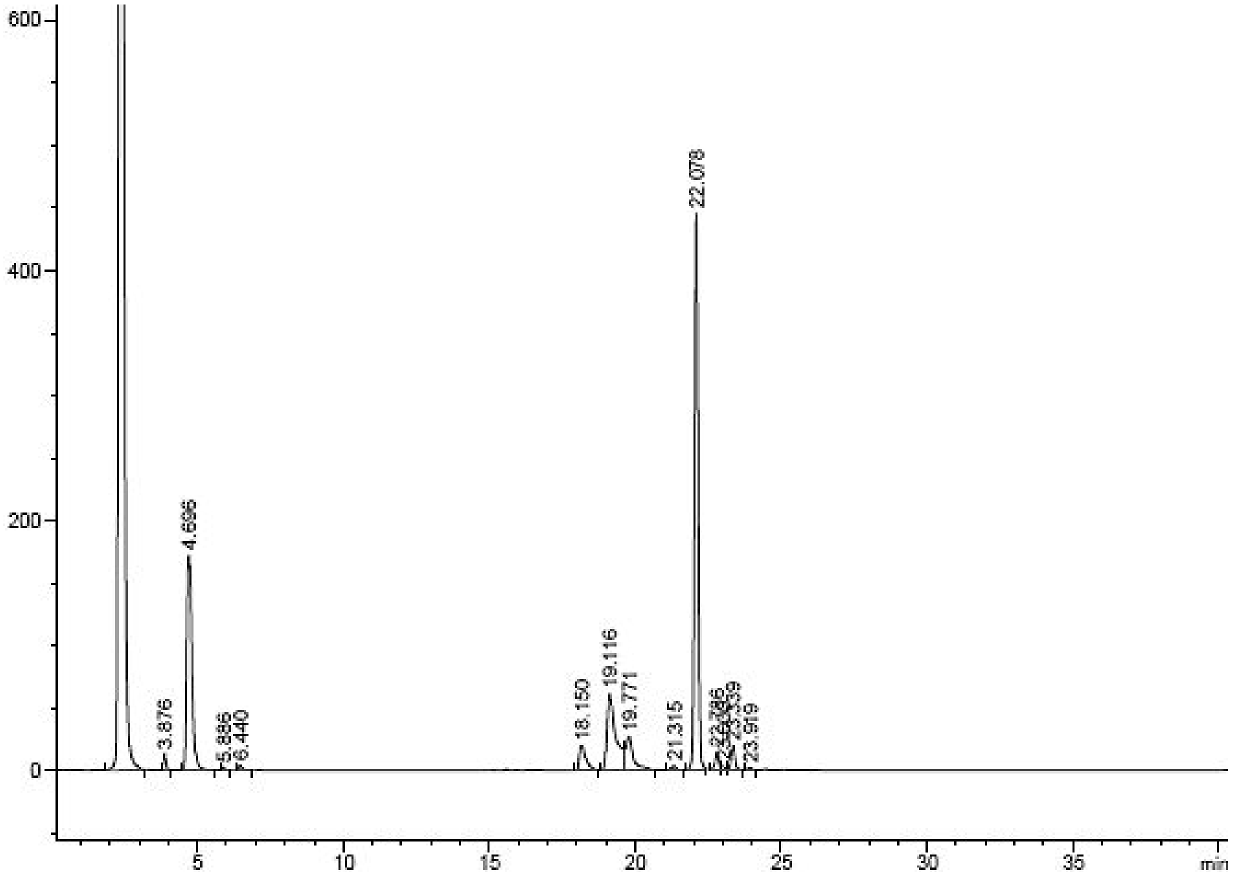

Figure 34: NP-HPLC trace of crude **27** (10-100%).

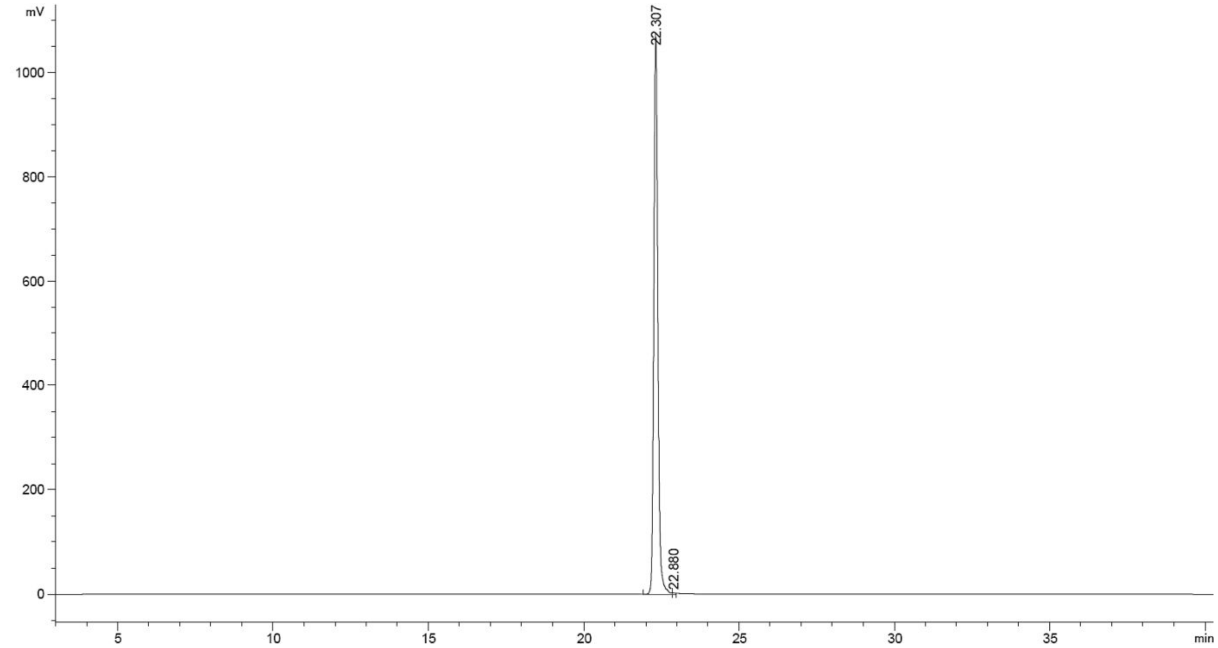

Figure 35: NP-HPLC trace of pure **27** (10-100%).

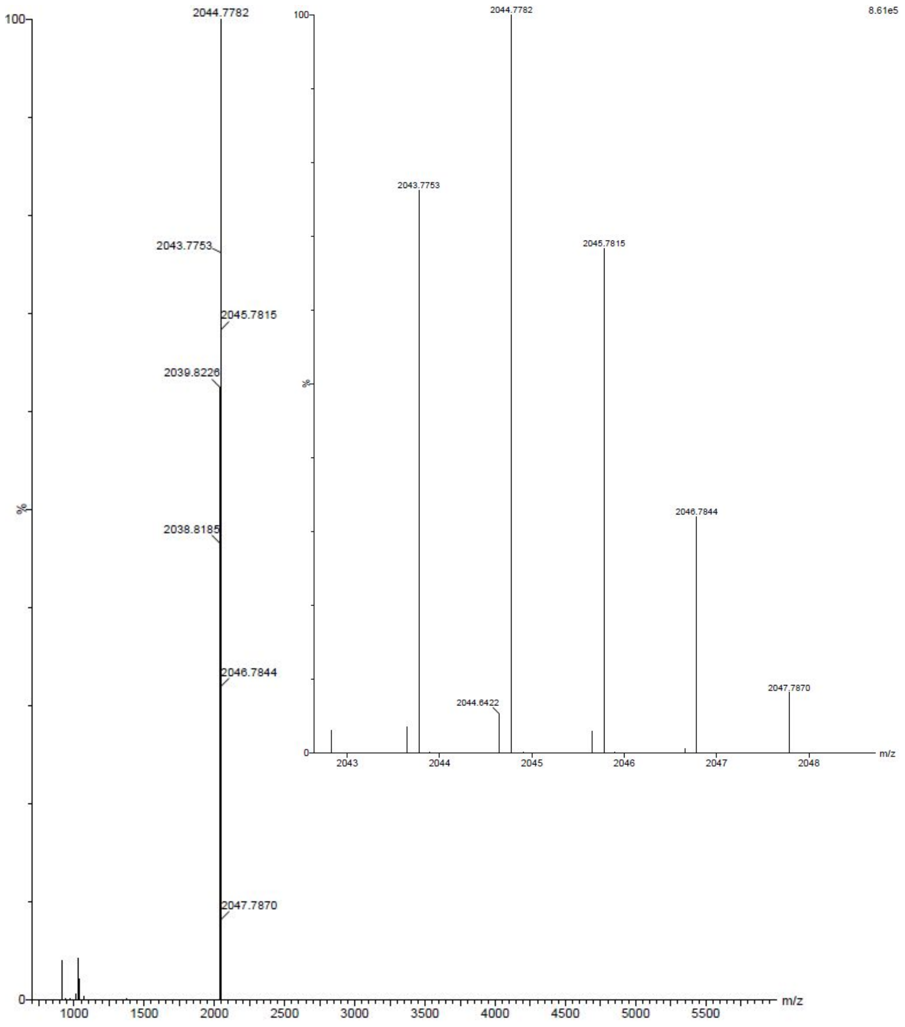

Figure 36: Q-TOF MS-spectrum of **27**.

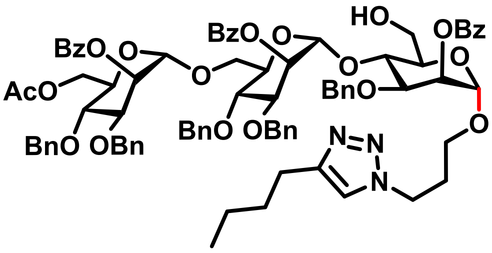

3-(4-Butyl-1H-1,2,3-triazol-1-yl)propan 6-O-acetyl-2-O-benzoyl-3,4-di-O-*α*-D-mannopyranosyl-(1→6)-2-O-benzoyl-3,4-di-O-benzyl-*α*-D-mannopyranosyl-(1→4)-2-O-benzoyl-3-O-benzyl-*α*-D-mannopyranoside (28)

Step	Building Block	Modules	Notes
AGA	BB **9**, 6 eq.	**A** – first coupling	-40°C (T1) 5 min<br>-20°C (T2) 35 min
		**B** – RV Wash<br>**C** – Acidic Wash<br>**D** – Capping	
	**2x**<br>BB **14**, 4 eq.	**E** – Fmoc Deprotection<br>**C** – Acidic Wash<br>**F** – Phosphate coupling	-30°C (T1) 20 min<br>0°C (T2) 20 min
	1-azidopropanol, 20 eq.	**E** – Fmoc Deprotection<br>**D** – Capping<br>**C** – Acidic Wash<br>**G** – Thioglycoside Coupling	20°C (T1) 5 min<br>35°C (T2) 35 min
Post AGA	1-hexyne, 20 eq.	**M** – CuAAC	16 h
		**J** – Batch Photocleavage	16 h
Purification		**L** – NP – (10 – 100%)	R_t = 26.5 min

Performed on 0.016 mmol scale. After a procedure including automated glycan assembly, on-resin CuAAC, photo-cleavage and purification, **28** was obtained as a translucent resin (7.7 mg, 5.2 µmol, 33%).

R_t (NP - 10 – 100%) = 26.5 min.

^{1}H NMR (400 MHz, $CDCl_3$) δ 8.13 – 8.02 (m, 6H), 7.66 – 7.60 (m, 1H), 7.58 – 7.52 (m, 1H), 7.51 – 7.45 (m, 5H), 7.44 – 7.38 (m, 2H), 7.37 – 7.29 (m, 10H), 7.28 – 7.20 (m, 10H), 7.18 – 7.08 (m, 6H), 5.83 (dd, J = 3.1, 1.9 Hz, 1H, H2''), 5.81 (dd, J = 3.2, 1.9 Hz, 1H, H2'), 5.52 (dd, J = 3.3, 1.8 Hz, 1H, H2), 5.49 (d, J = 1.9 Hz, 1H, α-1→4-H1'), 5.11 (d, J = 1.9 Hz, 1H, α-1→6-H1''), 4.93 (s, 1H, α-1→4-H1), 4.92 – 4.88 (m, 2H), 4.87 (d, J = 8.4 Hz, 1H), 4.79 (d, J = 11.2 Hz, 1H), 4.73 (d, J = 11.1 Hz, 1H), 4.63 (d, J = 11.1 Hz, 1H), 4.60 (d, J = 11.1 Hz, 1H), 4.55 (d, J = 11.0 Hz, 1H), 4.52 (d, J = 11.4 Hz, 1H), 4.50 – 4.40 (m, 3H), 4.35 – 4.20 (m, 3H), 4.20 – 4.11 (m, 1H), 4.09 (t, J = 3.1 Hz, 1H), 4.07 (t, J = 3.1 Hz, 1H), 4.03 – 3.76 (m, 9H), 3.73 (ddd, J = 9.8, 4.0, 2.3 Hz, 1H), 3.44 (ddd, J = 10.1, 7.0, 4.8 Hz, 1H), 2.82 – 2.72 (m, 2H), 2.24 (dddd, J = 17.3, 15.9, 8.0, 5.7 Hz, 2H), 2.02 (s, 3H, CH_3-Ac), 1.69 (tt, J = 7.7, 6.5 Hz, 2H), 1.41 (h, J = 7.3 Hz, 2H), 1.34 (s, 1H), 0.95 (t, J = 7.3 Hz, 3H, CH_3-butyl).

^{13}C NMR (101 MHz, $CDCl_3$) δ 170.7 (CO-Ac), 165.9 (CO-Bz), 165.46 (CO-Bz), 165.3 (CO-Bz), 148.5, 138.0, 138.0, 138.0, 137.8, 137.1, 133.4, 133.3, 133.2, 129.9, 129.9, 129.4, 128.5, 128.5, 128.4, 128.4, 128.3, 128.3, 128.2, 127.8, 127.8, 127.7, 127.7, 127.6, 120.9 (CH-triazole), 100.2 (C1'), 98.1 (C1''), 97.9 (C1), 78.6, 78.1, 75.2, 75.0, 74.0, 73.3, 72.0, 71.7, 71.3, 70.0, 68.7, 68.5, 68.4, 67.1, 64.5, 63.2, 61.9, 47.0, 31.6, 30.2, 25.3, 22.4, 20.8 (CH_3-Ac), 13.9 (CH_3-butyl).

HRMS (ESI): $C_{85}H_{92}N_3NaO_{20}$ $[M+H]^+$; calculated: 1474.6274, found: 1474.6295

Optical rotation: $[\alpha]_D^{25}$ = +3.0° (c = 0.5, $CHCl_3$)

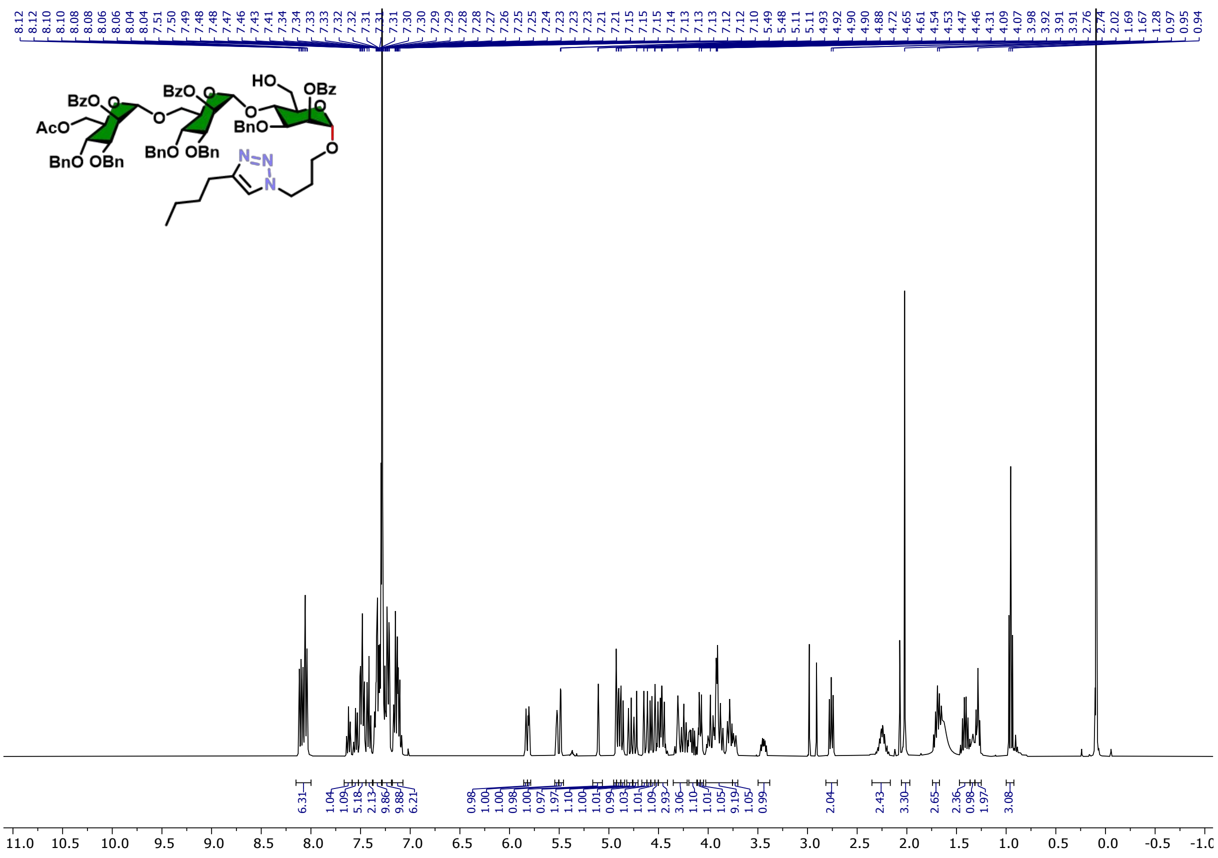

8.12 8.12 8.10 8.10 8.08 8.08 8.06 8.06 8.04 8.04 7.51 7.50 7.49 7.48 7.48 7.47 7.46 7.43 7.41 7.34 7.34 7.33 7.33 7.32 7.32 7.31 7.31 7.31 7.30 7.30 7.29 7.29 7.28 7.28 7.27 7.26 7.25 7.25 7.24 7.23 7.23 7.23 7.21 7.21 7.15 7.15 7.15 7.14 7.13 7.13 7.13 7.12 7.12 7.10 5.49 5.48 5.11 5.11 4.93 4.92 4.90 4.90 4.88 4.72 4.65 4.61 4.54 4.53 4.47 4.46 4.31 4.09 4.07 3.98 3.92 3.91 3.91 2.76 2.74 2.02 1.69 1.67 1.28 0.97 0.95 0.94
BzO
AcO
BnO OBn
BzO
BnO OBn
HO
OBz
BnO
N=N
N
O
6.31 1.04 1.09 5.18 2.13 9.86 9.88 6.21 0.98 1.00 1.00 0.98 1.00 0.97 1.97 1.10 1.00 1.01 0.99 1.03 1.01 1.09 2.93 3.06 1.10 1.01 1.05 9.19 1.05 0.99 2.04 2.43 3.30 2.65 2.36 0.98 1.97 3.08
11.0 10.5 10.0 9.5 9.0 8.5 8.0 7.5 7.0 6.5 6.0 5.5 5.0 4.5 4.0 3.5 3.0 2.5 2.0 1.5 1.0 0.5 0.0 -0.5 -1.0

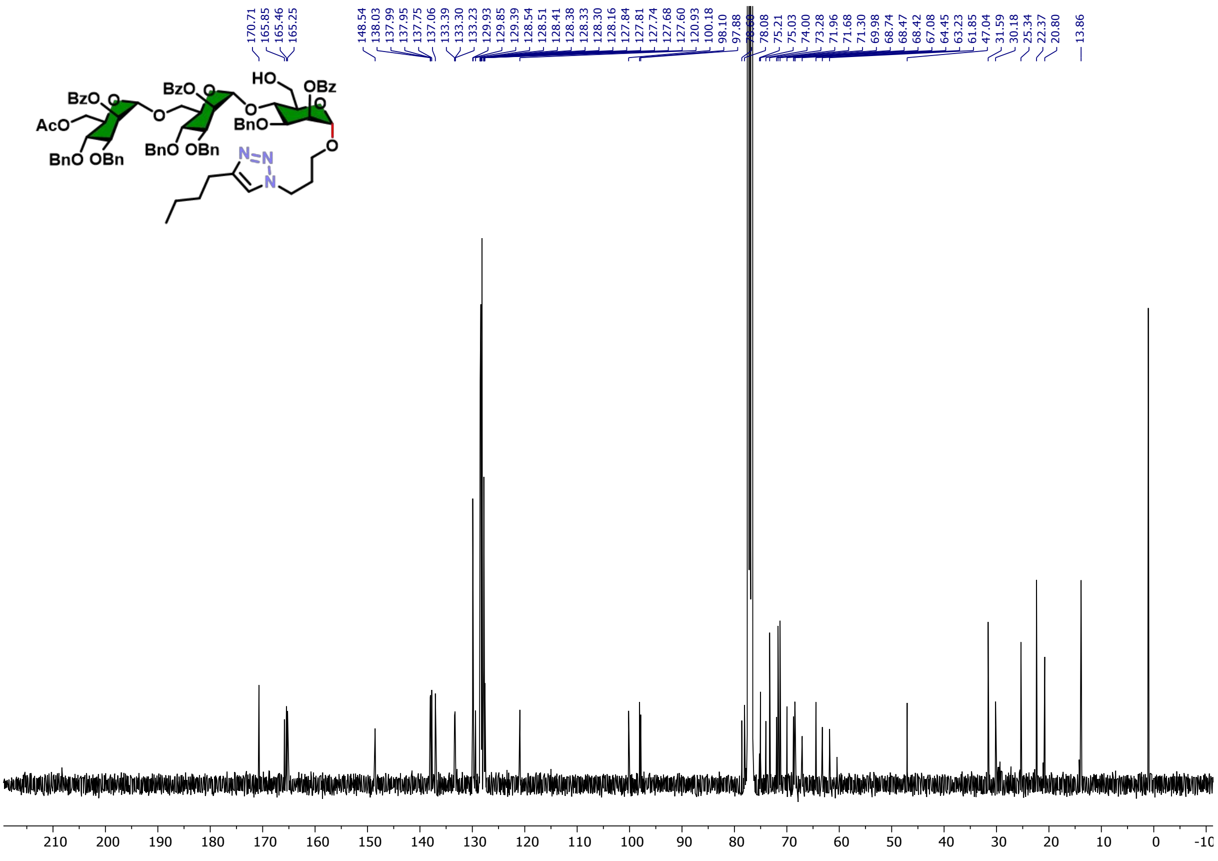

170.71 165.85 165.46 165.25 148.54 138.03 137.99 137.95 137.75 137.06 133.39 133.30 133.23 129.93 129.85 129.39 128.54 128.51 128.41 128.38 128.33 128.30 128.16 127.84 127.81 127.74 127.68 127.60 120.93 100.18 98.10 97.88 78.08 75.21 75.03 74.00 73.28 71.96 71.68 71.30 69.98 68.74 68.47 68.42 67.08 64.45 63.23 61.85 47.04 31.59 30.18 25.34 22.37 20.80 13.86
BzO
AcO
BnO OBn
BzO
BnO OBn
HO
OBz
BnO
N=N
N
O
210 200 190 180 170 160 150 140 130 120 110 100 90 80 70 60 50 40 30 20 10 0 -10

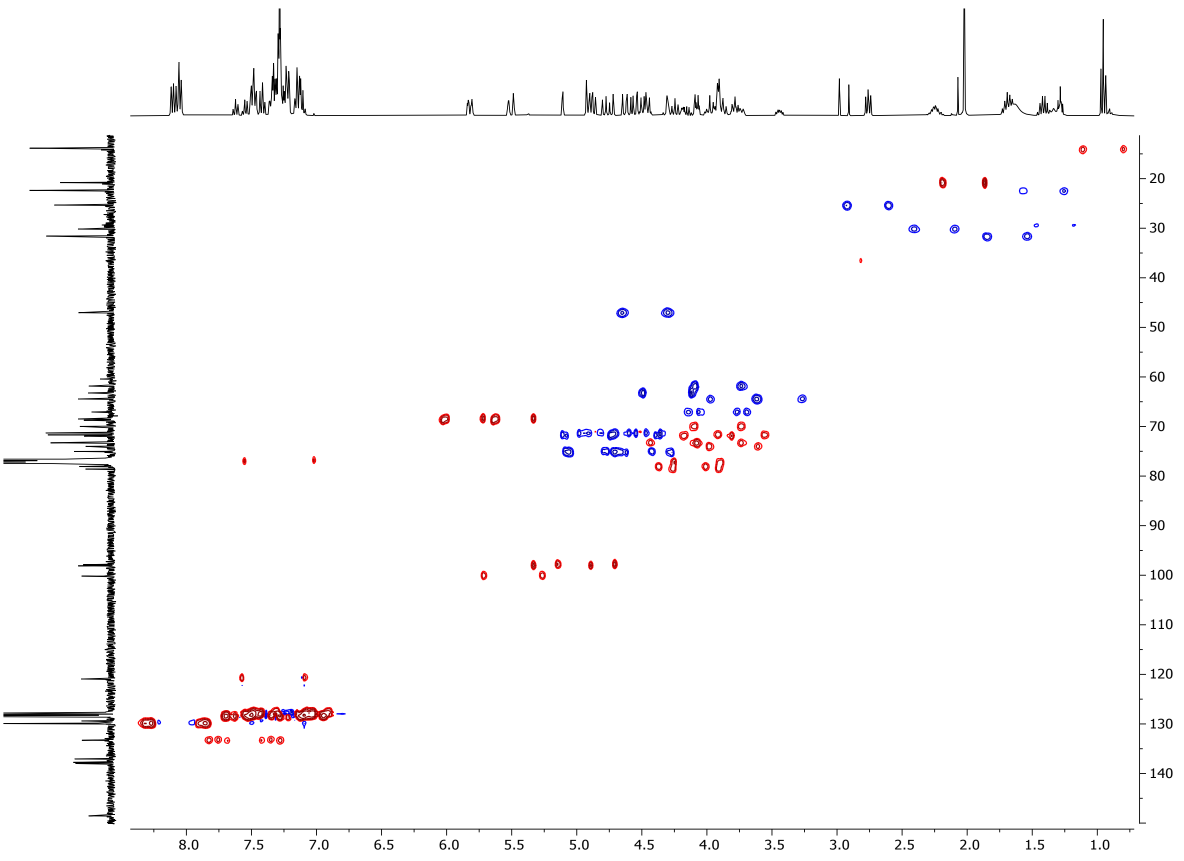
20
30
40
50
60
70
80
90
100
110
120
130
140
8.0 7.5 7.0 6.5 6.0 5.5 5.0 4.5 4.0 3.5 3.0 2.5 2.0 1.5 1.0

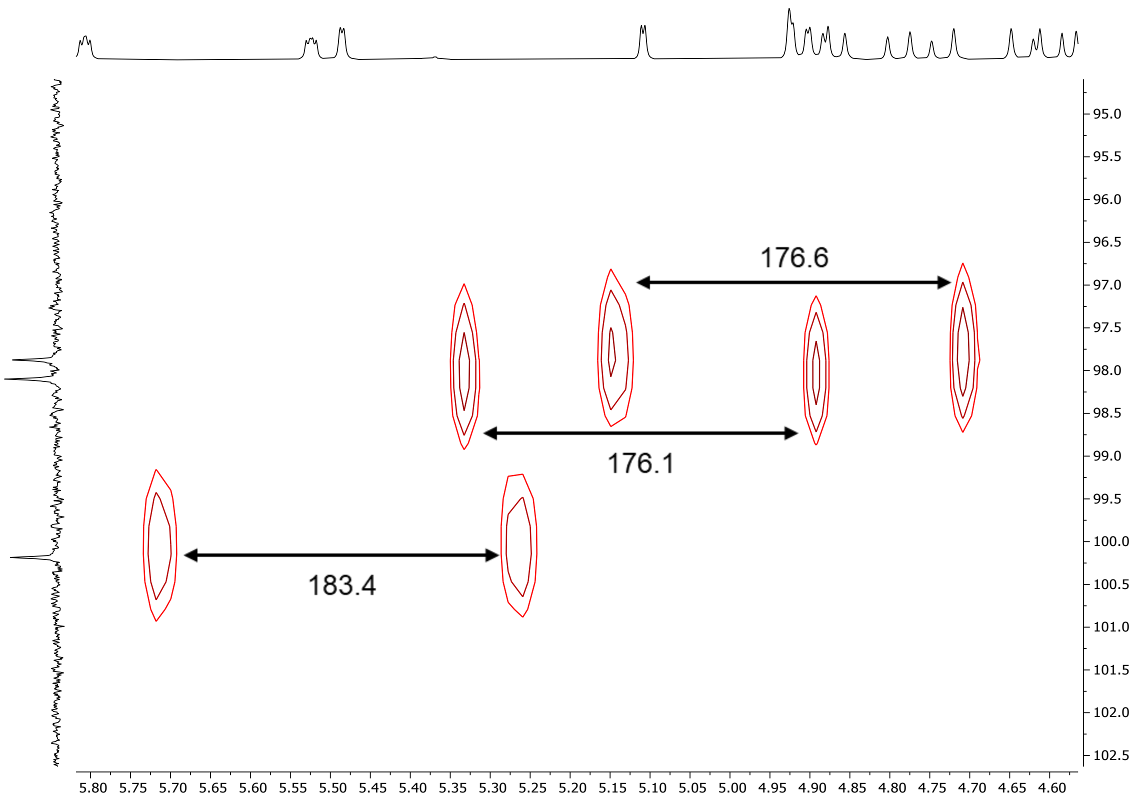
176.6
176.1
183.4
95.0 95.5 96.0 96.5 97.0 97.5 98.0 98.5 99.0 99.5 100.0 100.5 101.0 101.5 102.0 102.5
5.80 5.75 5.70 5.65 5.60 5.55 5.50 5.45 5.40 5.35 5.30 5.25 5.20 5.15 5.10 5.05 5.00 4.95 4.90 4.85 4.80 4.75 4.70 4.65 4.60

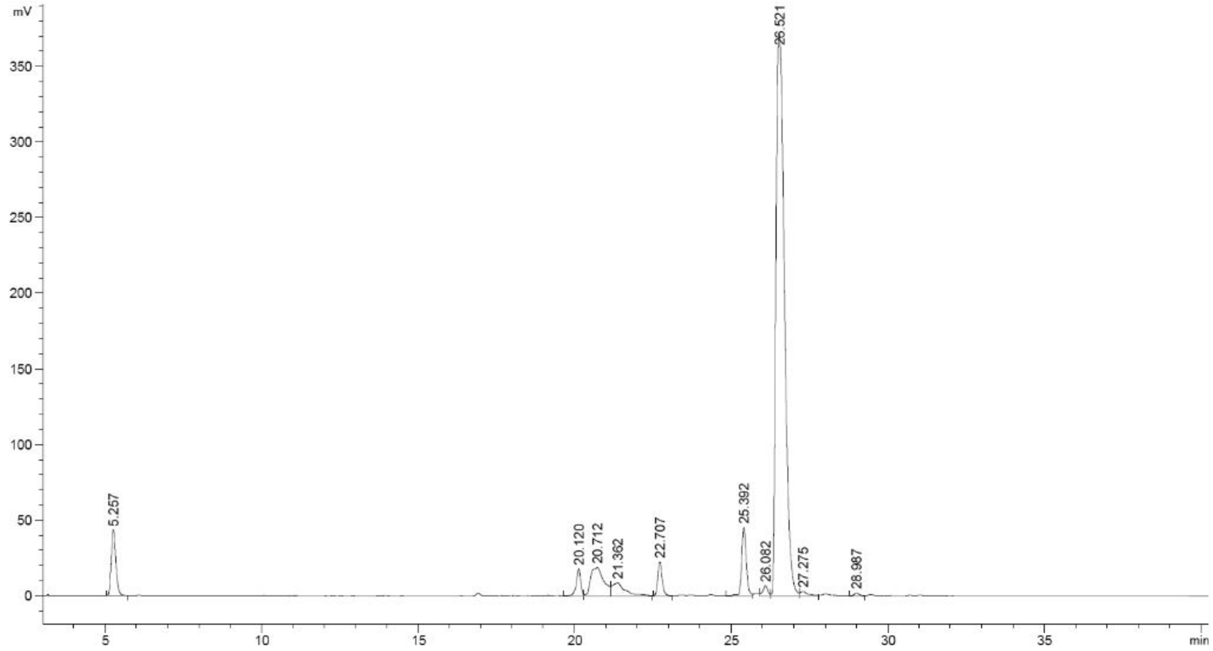

Figure 37: NP-HPLC trace of crude **28** (10-100%).

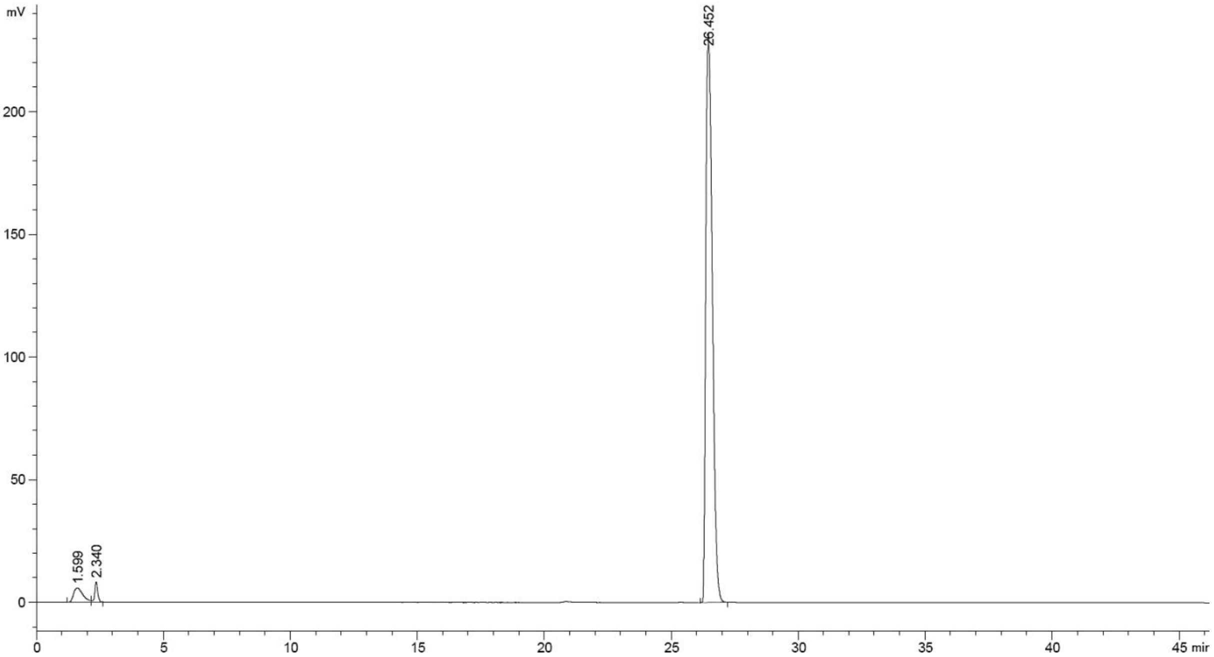

Figure 38: NP-HPLC trace of pure **28** (10-100%).

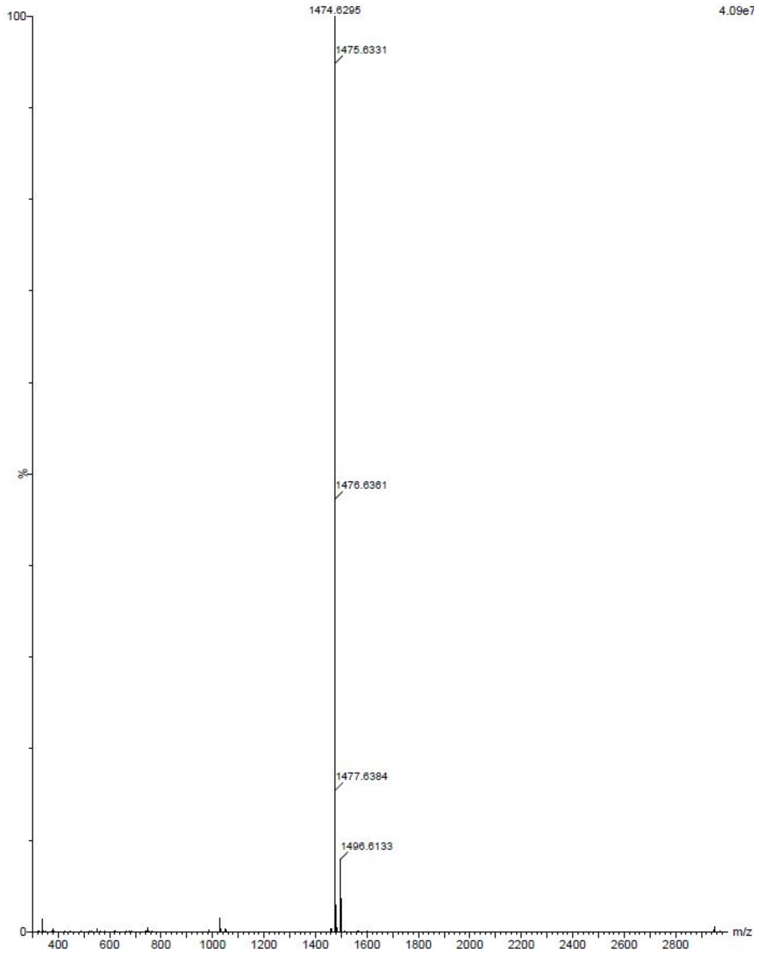

Figure 39: Q-TOF MS-spectrum of **28**.

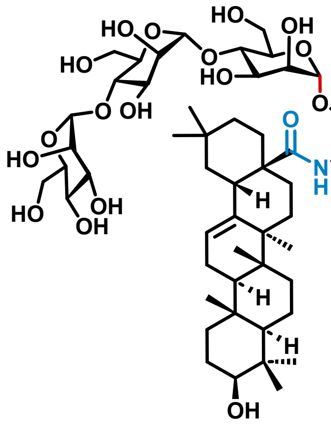

2-Ethan *α*-D-mannopyranosyl-(1→4)-*α*-D-mannopyranosyl)-(1→4)-(*α*-D-mannopyranosyl)- (28-oleanolic)-1-carboxamide (30)

Step	Building Block	Modules	Notes
AA	BB **9**, 6 eq.	**A** – first coupling	-40°C (T1) 5 min<br>-20°C (T2) 35 min
		**B** – RV Wash<br>**C** – Acidic Wash<br>**D** – Capping	
	**2x**<br>BB **10**, 4 eq.	**E** – Fmoc Deprotection<br>**C** – Acidic Wash<br>**F** – Phosphate Coupling	-30°C (T1) 10 min<br>0°C (T2) 20 min
	BB **29**, 10 eq. in<br>1:1 CH_2Cl_2/dioxane	**E** – Fmoc Deprotection<br>**D** – Capping<br>**C** – Acidic Wash<br>**G** – Thioglycoside Coupling	18°C (T1) 10 min<br>35°C (T2) 35 min
Peptide Coupling	Oleanolic acid 12 eq.	**E** – Fmoc Deprotection<br>**H** – Peptide Coupling	3x 20% piperidine<br>3 h
Post AGA		**I** – Methanolysis<br>**J** – Batch Photocleavage<br>**K** – Hydrogenolysis	16 h<br>16 h<br>16 h
Purification		**L** – C18 - (30 – 80%)	R_t =21.3 min

After a procedure including automated glycan assembly, SPPS, on-resin methanolysis, photo-cleavage, hydrogenolysis, purification and lyophilization, **30** was obtained as a white powder (2.0 mg, 2.03 µmol, 10%).

R_t (C18 - 30 – 80%) = 21.3 min.

^{1}H NMR (600 MHz, D_2O/MeCN 1:1) *δ* 8.87 (s, 1H, NH), 5.89 (m, 1H, alkene), 5.68 (d, *J* = 2.1 Hz, 1H, *α*-H1'), 5.64 (d, *J* = 2.3 Hz, 1H, *α*-H1''), 5.25 (s, 1H, *α*-H1), 4.40 (dt, *J* = 8.4, 2.5 Hz, 2H), 4.31 – 4.24 (m, 7H), 4.23 – 3.89 (m, 12H), 3.65 (t, *J* = 12.1 Hz, 2H), 3.11 – 3.03 (m, 1H), 2.40 – 2.35 (m, 2H), 2.32 – 2.21 (m, 1H), 2.11 – 1.57 (m, 19H), 1.48 (t, *J* = 13.5 Hz, 2H), 1.42 – 1.35 (m, 12H), 1.21 (d, *J* = 3.3 Hz, 7H).

^{13}C NMR (151 MHz, D_2O/MeCN 1:1) *δ* 179.2 (CO-amide), 144.0 (alkene), 122.7 (alkene), 101.3 (C1''), 100.9 (C1'), 99.7 (C1), 78.2, 74.0, 73.4, 73.2, 72.0, 71.2, 71.0, 70.7, 70.5, 70.5, 70.2, 66.6, 66.1, 60.8, 60.7, 60.5, 54.7, 47.1, 46.2, 46.1, 41.4, 41.3, 38.9, 38.5, 38.0, 38.0, 36.4, 33.3, 32.2, 32.0, 31.9 (CH_3), 29.9, 27.3 (CH_3), 26.8, 26.1, 25.0 (CH_3), 22.9, 22. 9, 22.7 (CH_3), 17.8, 16.3 (CH_3), 14.9 (CH_3), 14.5 (CH_3).

HRMS (ESI): $C_{50}H_{83}NNaO_{18}$ $[M+Na]^+$; calculated: 1008.5508, found: 1008.5552.

Optical rotation: $[\alpha]_D^{25}$ = +74.0° (c = 0.05, H_2O/MeCN 1:1)

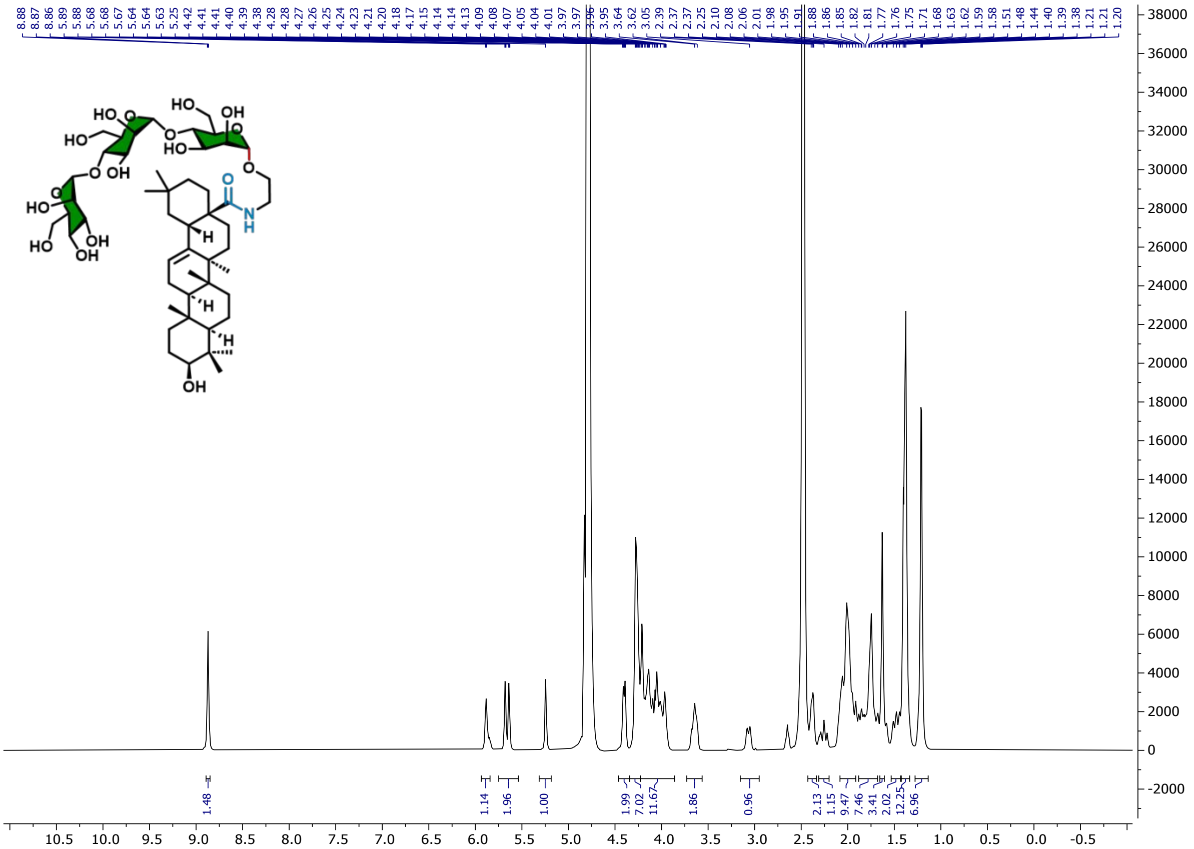

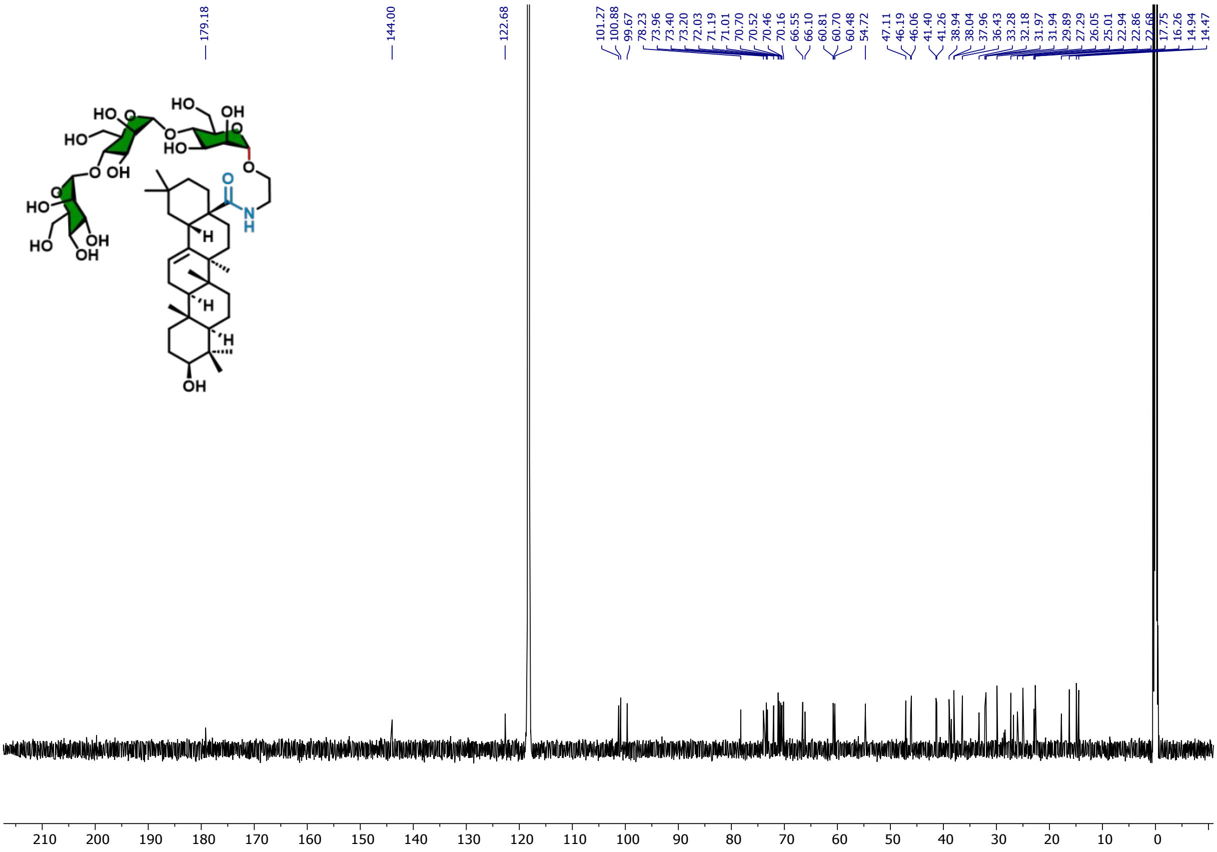
179.18
144.00
122.68
101.27
100.88
99.67
78.23
73.96
73.40
73.20
72.03
71.19
71.01
70.70
70.52
70.46
70.16
66.55
66.10
60.81
60.70
60.48
54.72
47.11
46.19
46.06
41.40
41.26
38.94
38.04
37.96
36.43
33.28
32.18
31.97
31.94
29.89
27.29
26.05
25.01
22.94
22.86
22.68
17.75
16.26
14.94
14.47

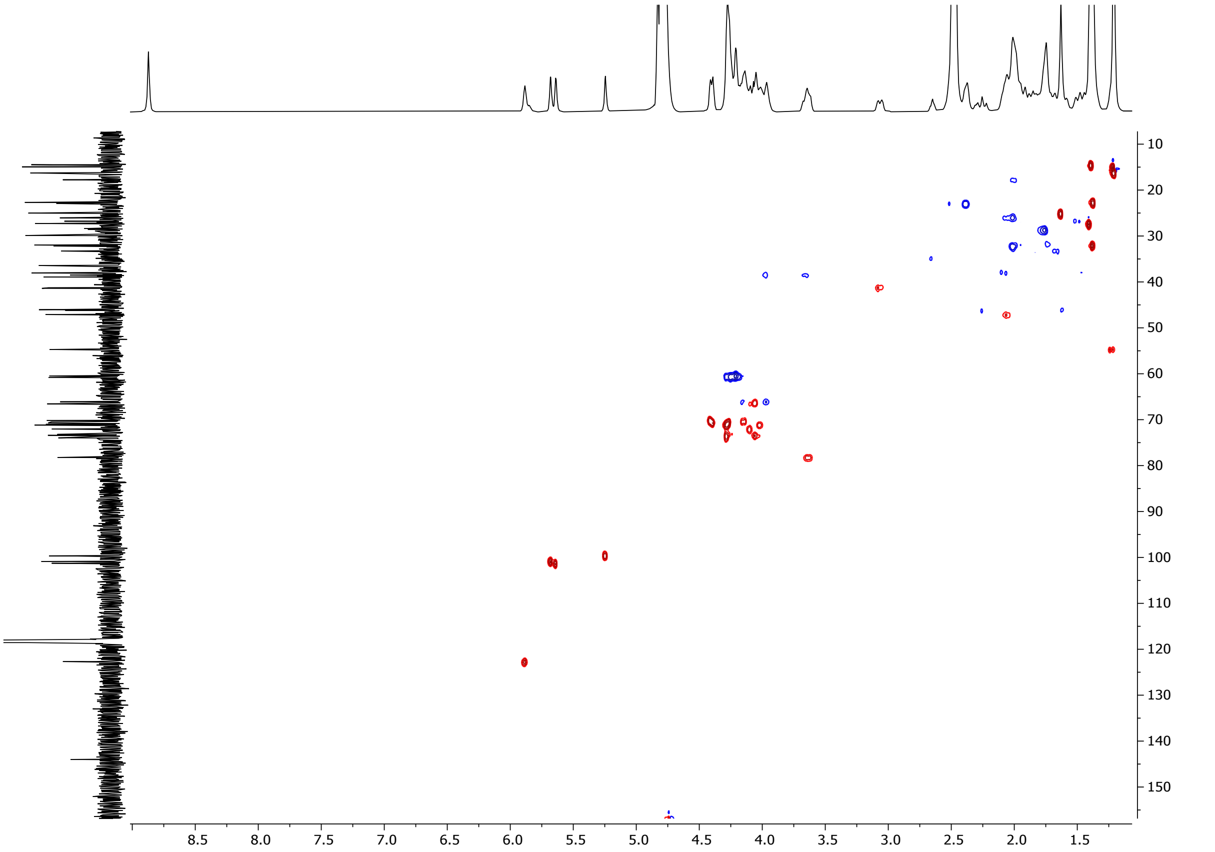

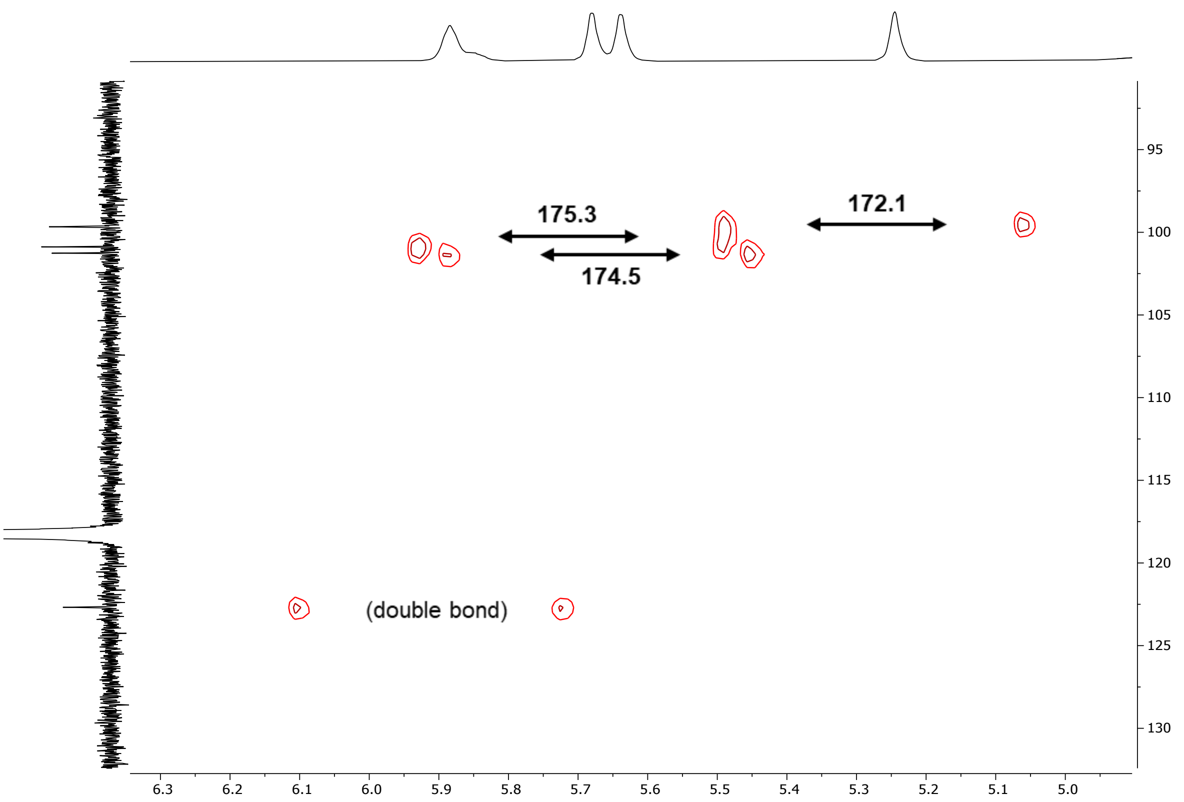

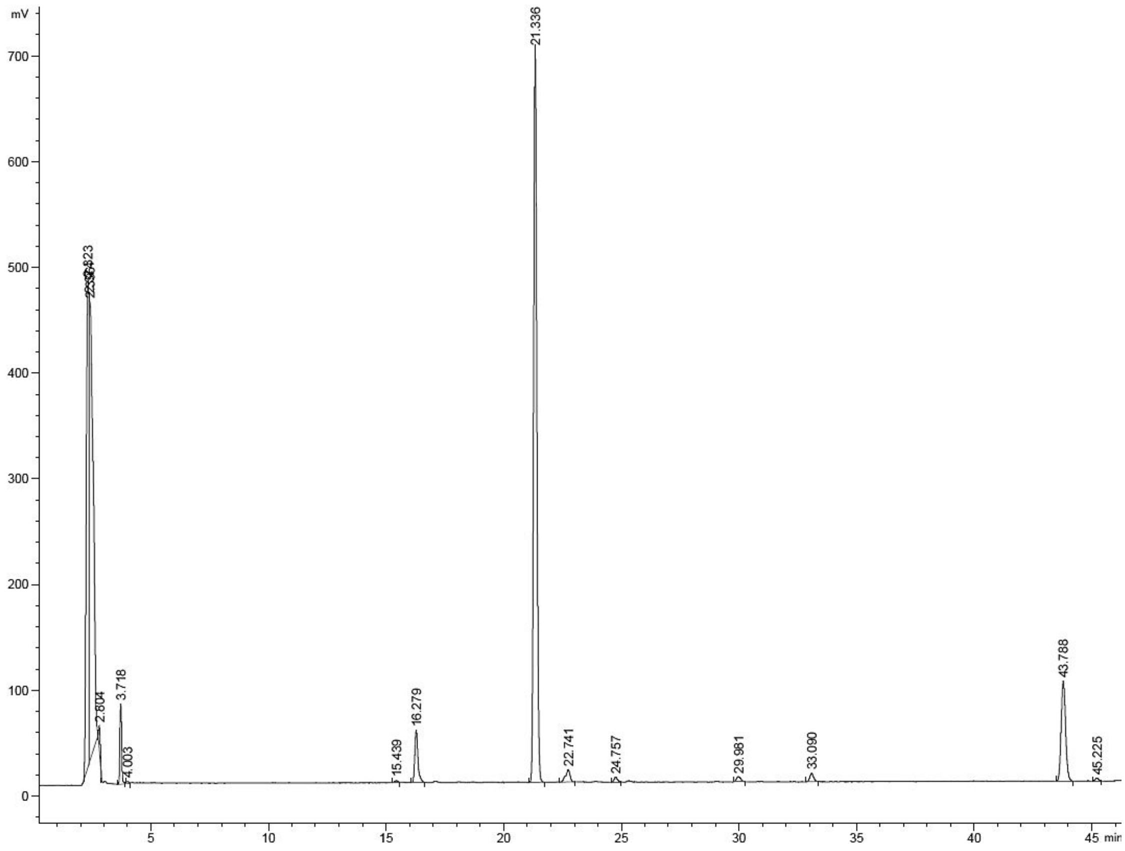

Figure 40: RP-HPLC trace of crude **30** (C_{18}; 30-80%).

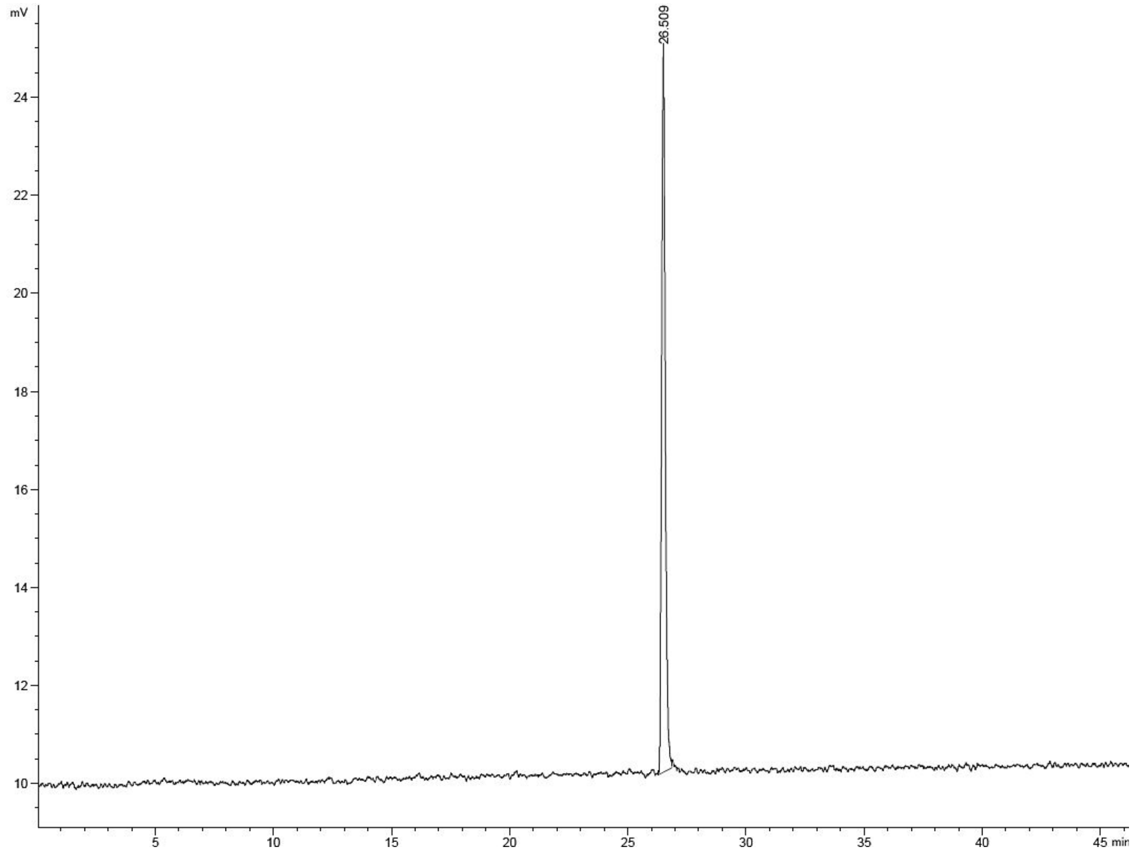

Figure 41: RP-HPLC trace of pure **30** (C_{18}; 10-100%).

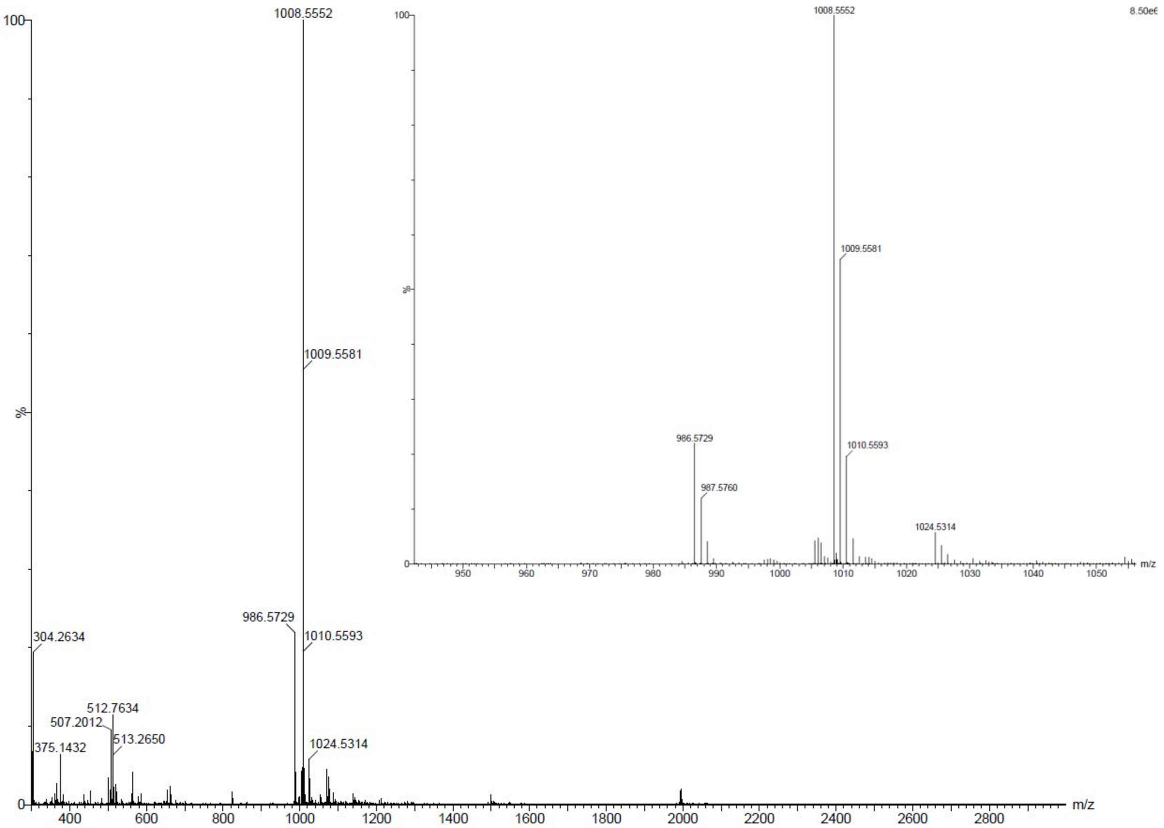

Figure 42: Q-TOF MS-spectrum of **30**.

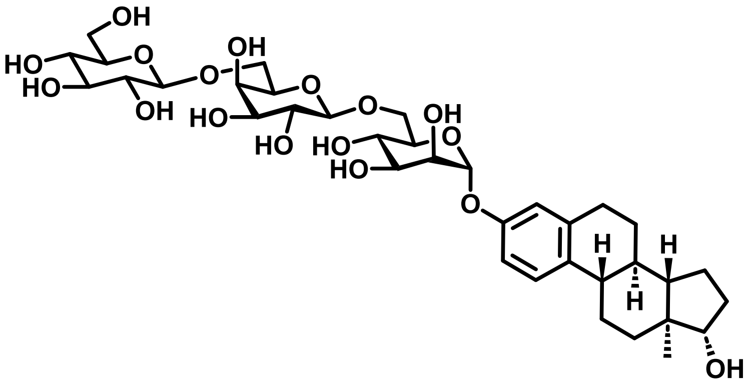

17-*β*-D-Glucopyranosyl-(1→6)-*β*-D-galactopyranosyl-(1→6)-*α*-D-mannopyranosyl-estradiol (31)

Step	Building Block	Modules	Notes
AGA	BB **24**, 8 eq.	**A** – first coupling	-40°C (T1) 5 min<br>-20°C (T2) 35 min
		**B** – RV Wash	
		**C** – Acidic Wash	
		**D** – Capping	
		**E** – Fmoc Deprotection	
		**C** – Acidic Wash	
	BB **7**, 6 eq.	**G** – Thioglycoside Coupling	-30°C (T1) 10 min<br>0°C (T2) 20 min
		**E** – Fmoc Deprotection	
		**C** – Acidic Wash	
	BB **32**, 6 eq.	**G** – Thioglycoside Coupling	-30°C (T1) 10 min<br>0°C (T2) 20 min
		**E** – Fmoc Deprotection	
		**C** – Acidic Wash	
	BB **33**, 6 eq.	**G** – Thioglycoside Coupling	-30°C (T1) 10 min<br>0°C (T2) 20 min
Post AGA		**I** – Methanolysis	16 h
		**J** – Batch Photocleavage	16 h
		**K** – Hydrogenolysis	16 h
Purification		**L** – C18 - (10 – 60%)	R_t = 25.2 min

After a procedure including automated glycan assembly, on-resin methanolysis, photo-cleavage, hydrogenolysis, purification and lyophilization, **31** obtained as a white powder (3.2 mg, 4.22 µmol, 21%).

R_t (RP-C18 - 10 – 60%) = 25.2 min.

^{1}H NMR (400 MHz, D_2O/MeCN 1:1) *δ* 7.80 (d, *J* = 8.7 Hz, 1H, Ar), 7.42 (dd, *J* = 8.6, 2.6 Hz, 1H, Ar), 7.35 (d, *J* = 2.6 Hz, 1H, Ar), 5.99 (d, *J* = 2.0 Hz, 1H, *α*-H1-Man), 4.92 (d, *J* = 8.0 Hz, 1H, *β*-H1-Gal), 4.83 (d, *J* = 7.7 Hz, 1H, *β*-H1-Glu), 4.59 – 4.54 (m, 1H), 4.51 – 4.44 (m, 2H), 4.43 – 4.23 (m, 8H), 4.22 – 4.13 (m, 2H), 4.05 (dd, *J* = 9.9, 3.2 Hz, 1H), 4.02 – 3.98 (m, 1H), 3.97 – 3.78 (m, 3H), 3.72 (t, *J* = 8.6 Hz, 1H), 3.36 (d, *J* = 7.9 Hz, 2H, benzylic estradiol), 2.91 – 2.80 (m, 1H), 2.75 – 2.64 (m, 1H), 2.47 – 2.38 (m, 2H), 2.27 – 2.17 (m, 1H), 2.06 – 1.77 (m, 7H), 1.77 – 1.66 (m, 1H), 1.26 (s, 3H, CH_3).

^{13}C NMR (101 MHz, D_2O/MeCN 1:1) *δ* 154.8 (CO-Ar), 139.7, 136.2, 127.8, 118.3, 115.4, 104.4 (C1-Glu), 103.8 (C1-Gal), 99.7 (C1-Man), 82.2, 77.1, 76.9, 74.5, 74.2, 73.7, 73.4, 71.8, 71.6, 71.2, 70.8, 69.5, 69.4, 67.2, 62.0, 50.8, 44.9, 44.0, 39.7, 37.5, 30.4, 30.3, 28.0, 27.2, 23.8, 11.9 (CH_3).

HRMS (ESI): $C_{36}H_{54}NaO_{17}$ $[M+Na]^+$; calculated: 781.3259, found: 781.3318.

Optical rotation: $[\alpha]_D^{25}$ = +70.0° (c = 0.05, H_2O/MeCN 1:1)

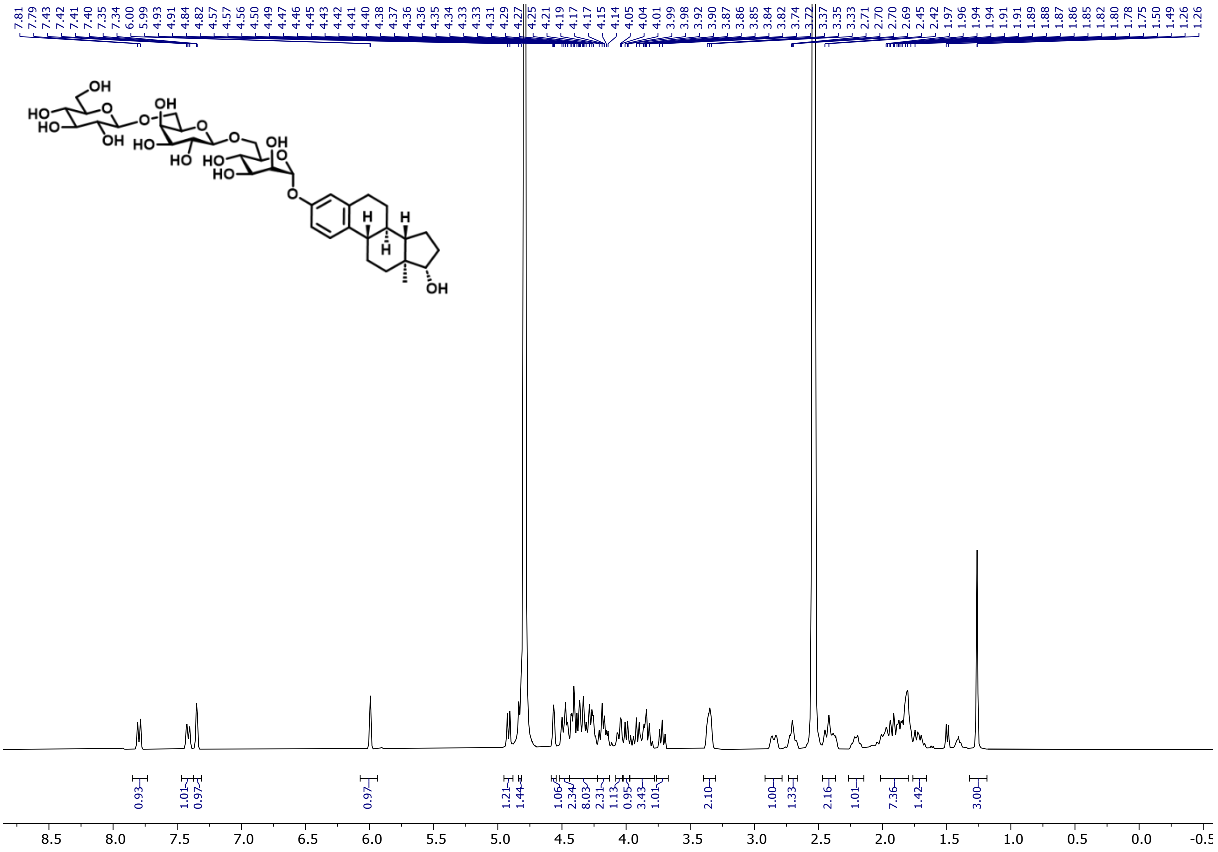

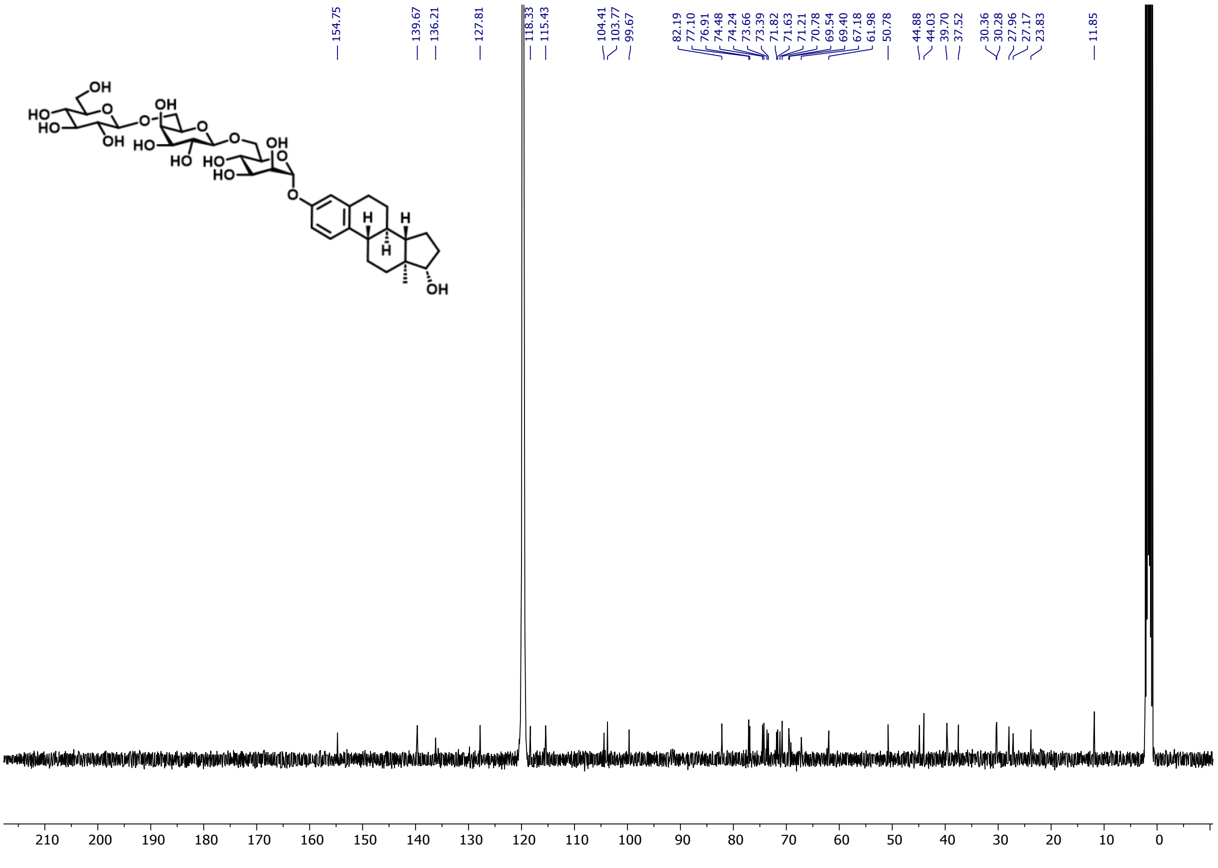

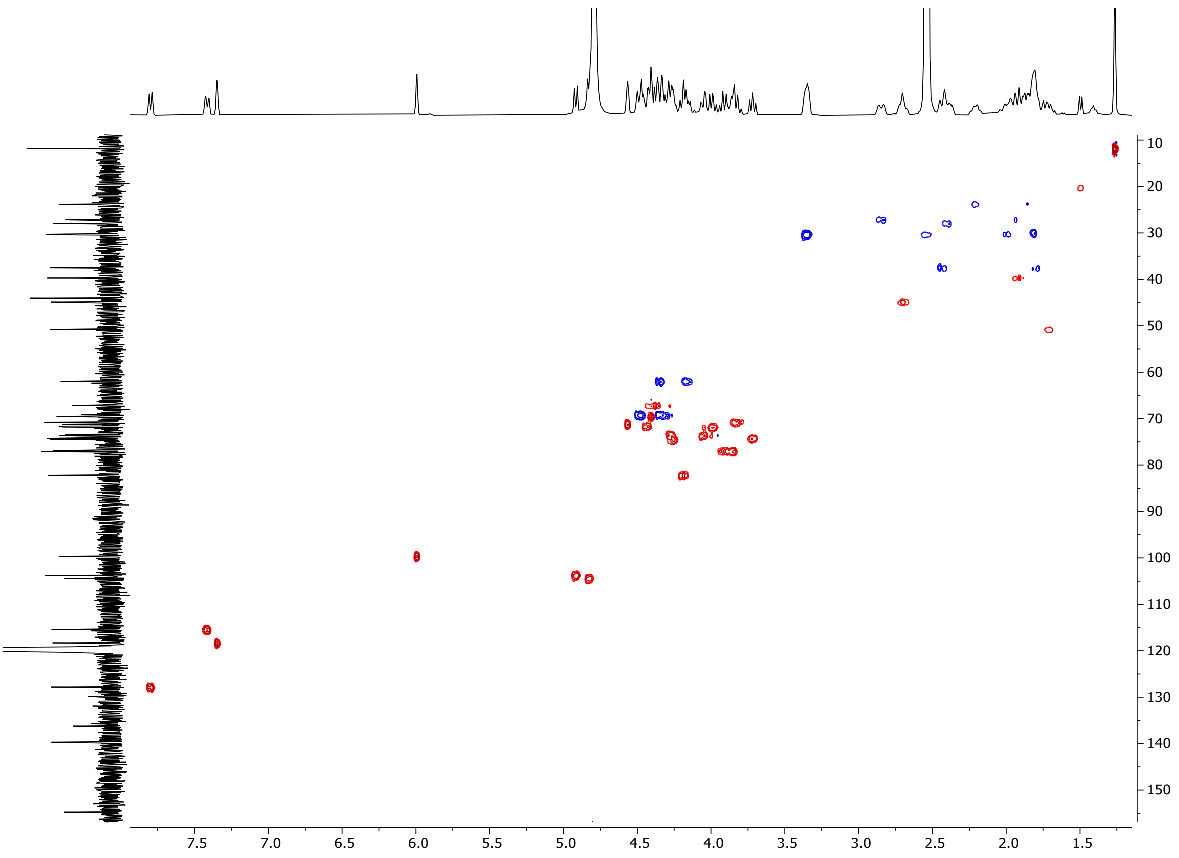

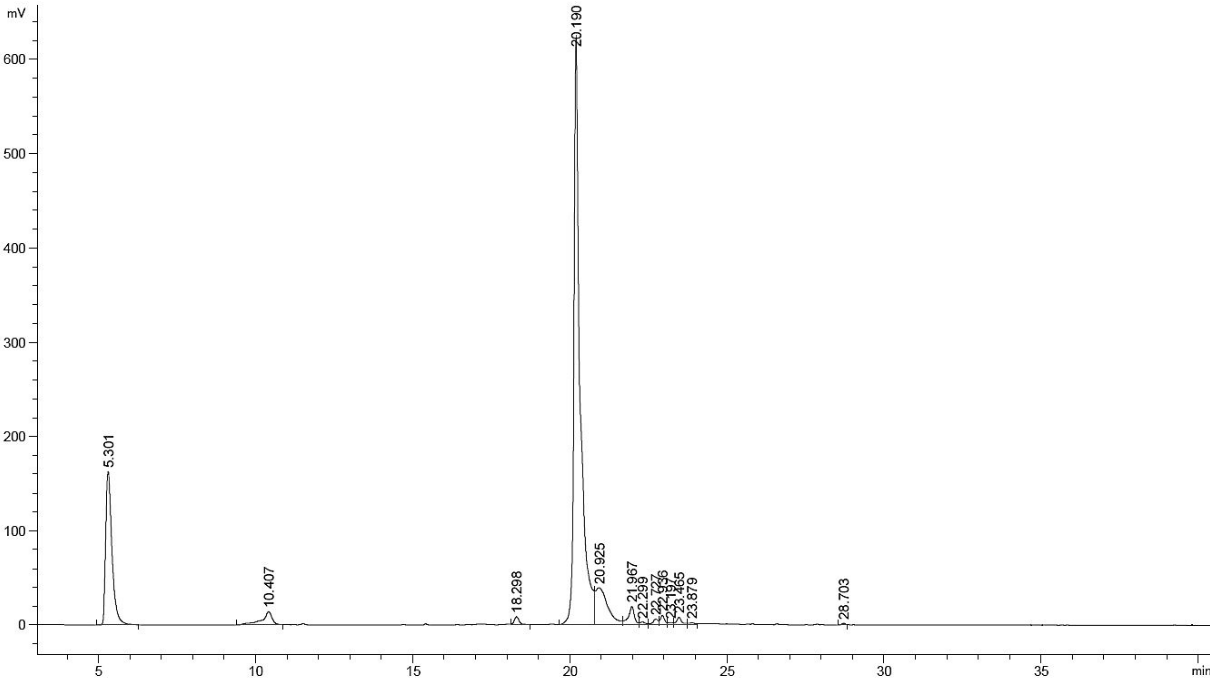

Figure 43: NP-HPLC trace of crude protected **31** (10-100%).

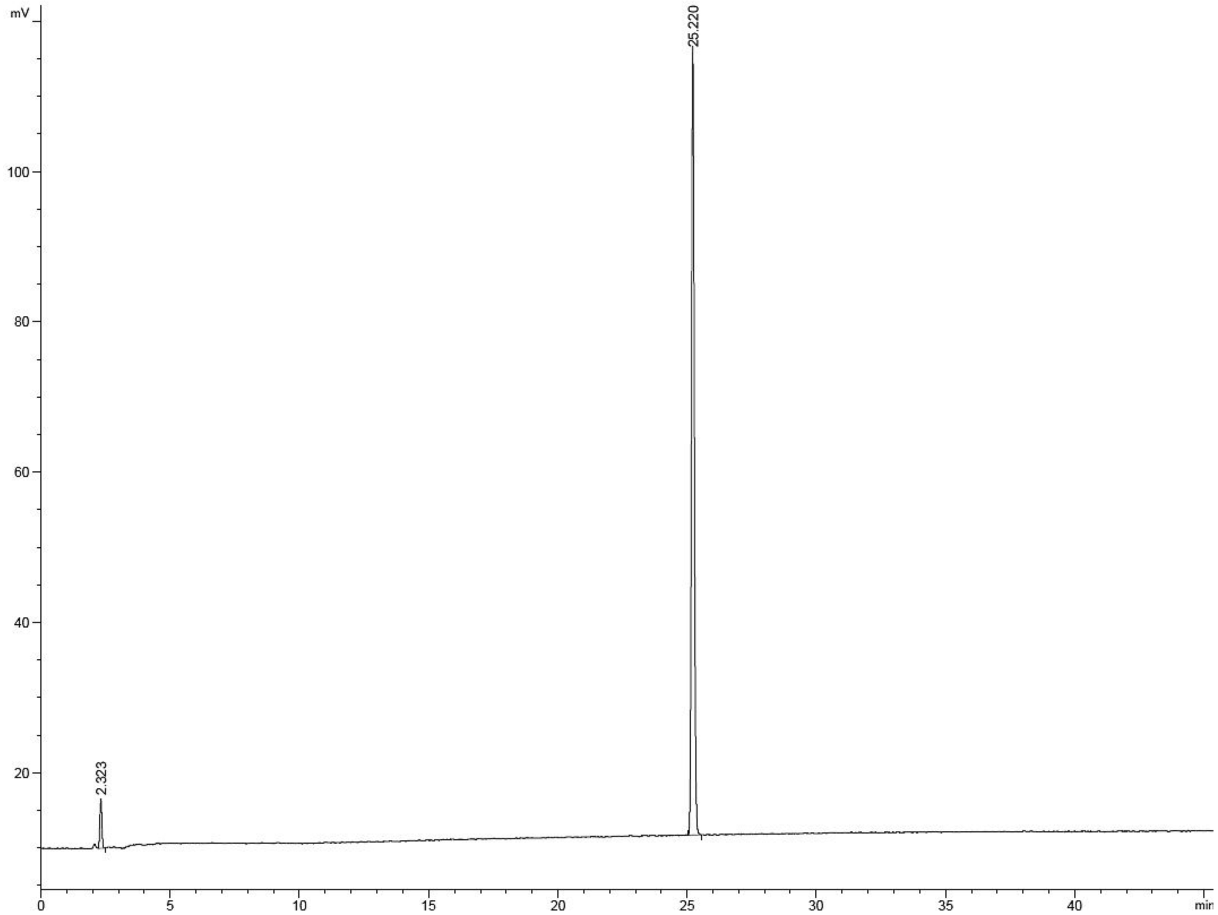

Figure 44: RP-HPLC trace of pure **31** (C_{18}; 10-60%).

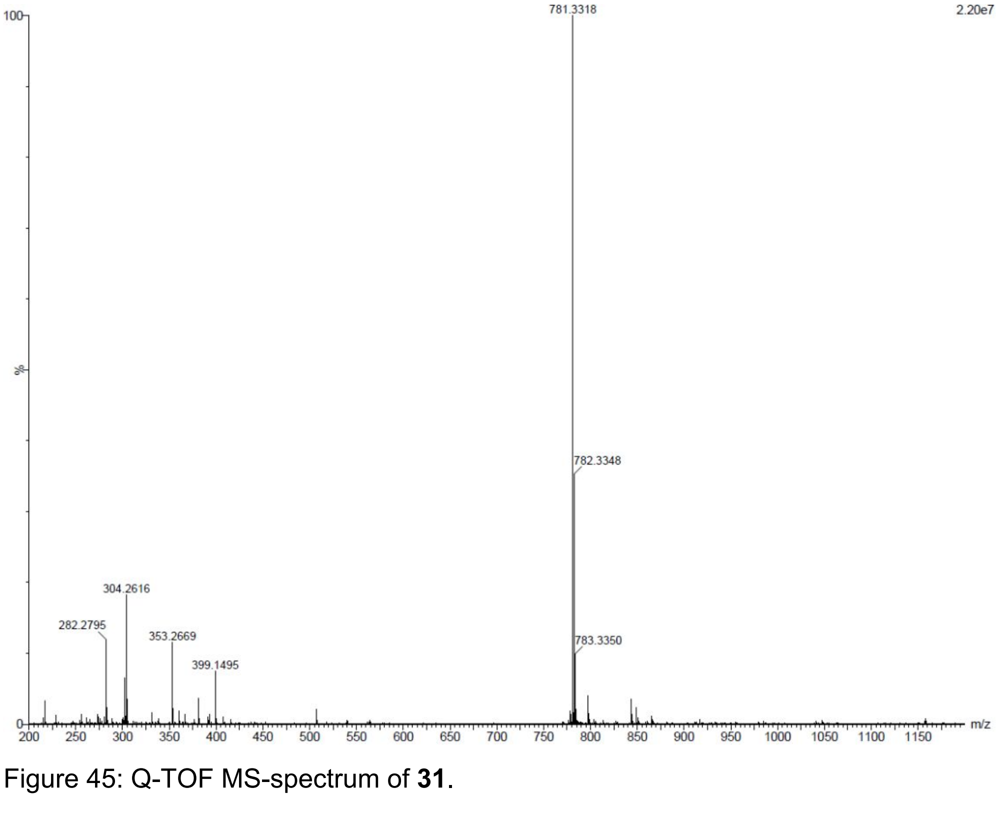

Figure 45: Q-TOF MS-spectrum of **31**.

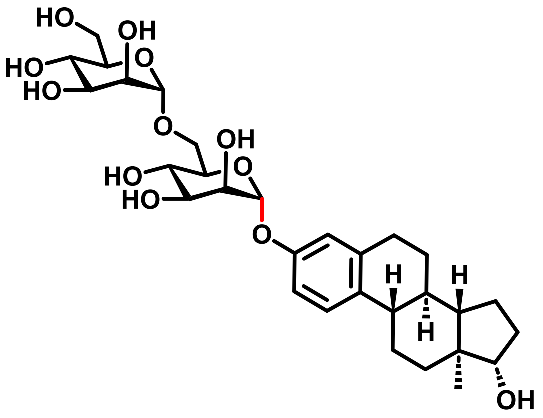

17-(*α*-D-mannopyranosyl-(1→6)-*α*-D-mannopyranosyl)-estradiol (34)

Step	Building Block	Modules	Notes
AGA	BB **24,** 8 eq.	**A** – first coupling	-40°C (T1) 5 min<br>-20°C (T2) 35 min
		**B** – RV Wash	
		**C** – Acidic Wash	
		**D** – Capping	
	**2x**<br>BB **14**, 4 eq.	**E** – Fmoc Deprotection	
		**C** – Acidic Wash	
		**F** – Phosphate coupling	-30°C (T1) 10 min<br>0°C (T2) 20 min
Post AGA		**I** – Methanolysis	16 h
		**J** – Batch Photocleavage	16 h
		**K** – Hydrogenolysis	16 h
Purification		**L** – C18 – (0 – 40%)	R_t = 40.9 min

After a procedure including automated glycan assembly, on-resin methanolysis, photo-cleavage, hydrogenolysis, purification and lyophilization, **34** obtained as a white powder (4.8 mg, 8.05 µmol, 37%).

R_t (C18 - 0 – 40%) = 40.9 min.

^{1}H NMR (600 MHz, D_2O/MeCN 4:1) δ 7.47 – 7.40 (m, 1H, Ar), 7.05 (dd, J = 8.6, 2.7 Hz, 1H, Ar), 6.98 (d, J = 2.7 Hz, 1H, Ar), 5.62 (d, J = 1.8 Hz, 1H, *α*-H1), 4.90 (d, J = 1.7 Hz, 1H, *α*-H1'), 4.22 (dd, J = 3.4, 1.8 Hz, 1H), 4.09 – 4.00 (m, 2H), 3.97 – 3.90 (m, 4H), 3.89 – 3.80 (m, 3H), 3.78 – 3.69 (m, 3H), 2.97 (dd, J = 9.0, 4.2 Hz, 2H, benzylic), 2.53

– 2.41 (m, 1H), 2.32 (ddd, J = 14.5, 7.9, 4.1 Hz, 1H), 2.09 – 1.98 (m, 2H), 1.88 – 1.78 (m, 1H), 1.69 – 1.27 (m, 6H), 0.88 (s, 3H, CH_3).

^{13}C NMR (151 MHz, D_2O/MeCN 4:1) δ 154.3 (CO-Ar), 139.5, 136.1, 127.5, 118.1, 115.2, 100.1, 99.3, 82.1, 73.4, 72.3, 71.7, 71.6, 70.9, 70.9, 67.6, 67.4, 66.2, 61.8, 50.4, 44.5, 43.7, 39.4, 37.2, 30.1, 29.9, 27.6, 26.8, 23.5, 11.6 (CH_3).

HRMS (ESI): $C_{33}H_{44}NaO_{12}$ [M+Na]$^+$; calculated: 619.2731, found: 619.2731.

Optical rotation: $[\alpha]_D^{25}$ = +128.0° (c = 0.05, H_2O/MeCN 1:1)

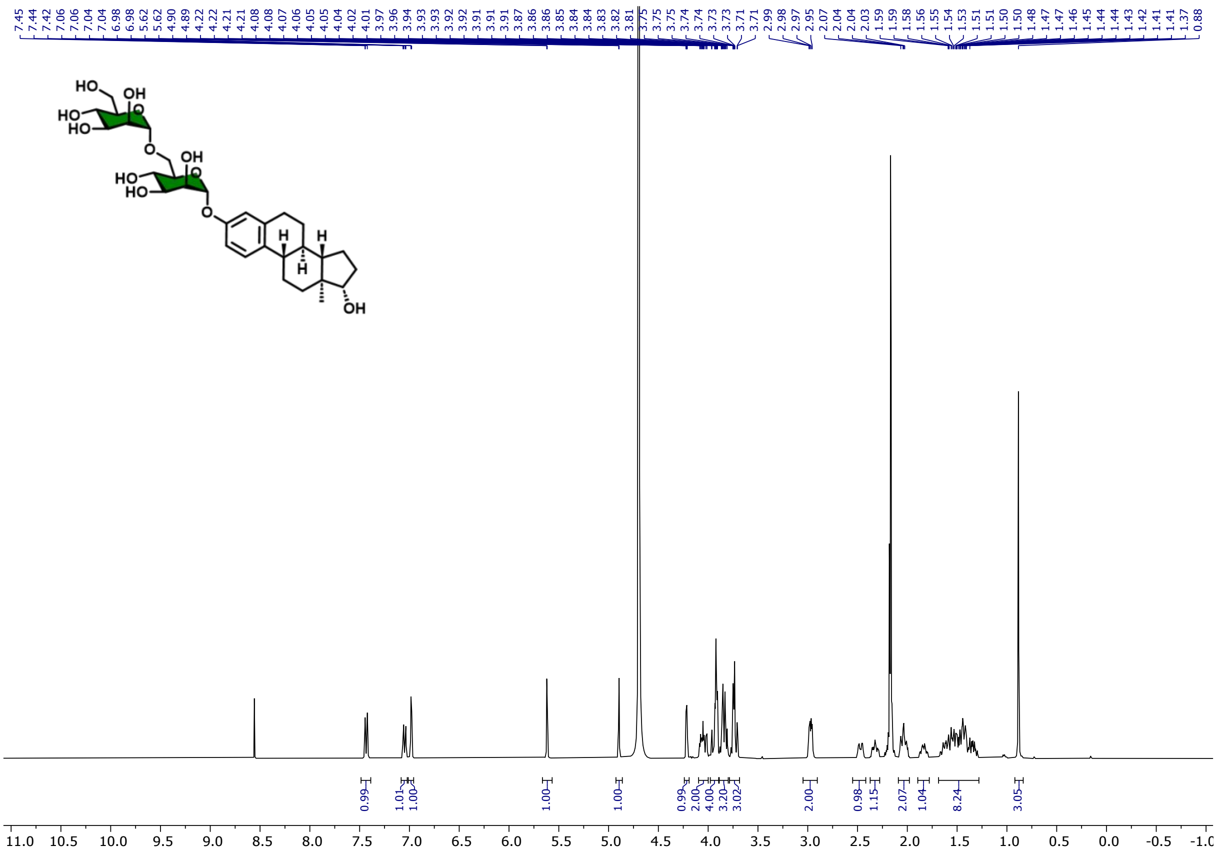

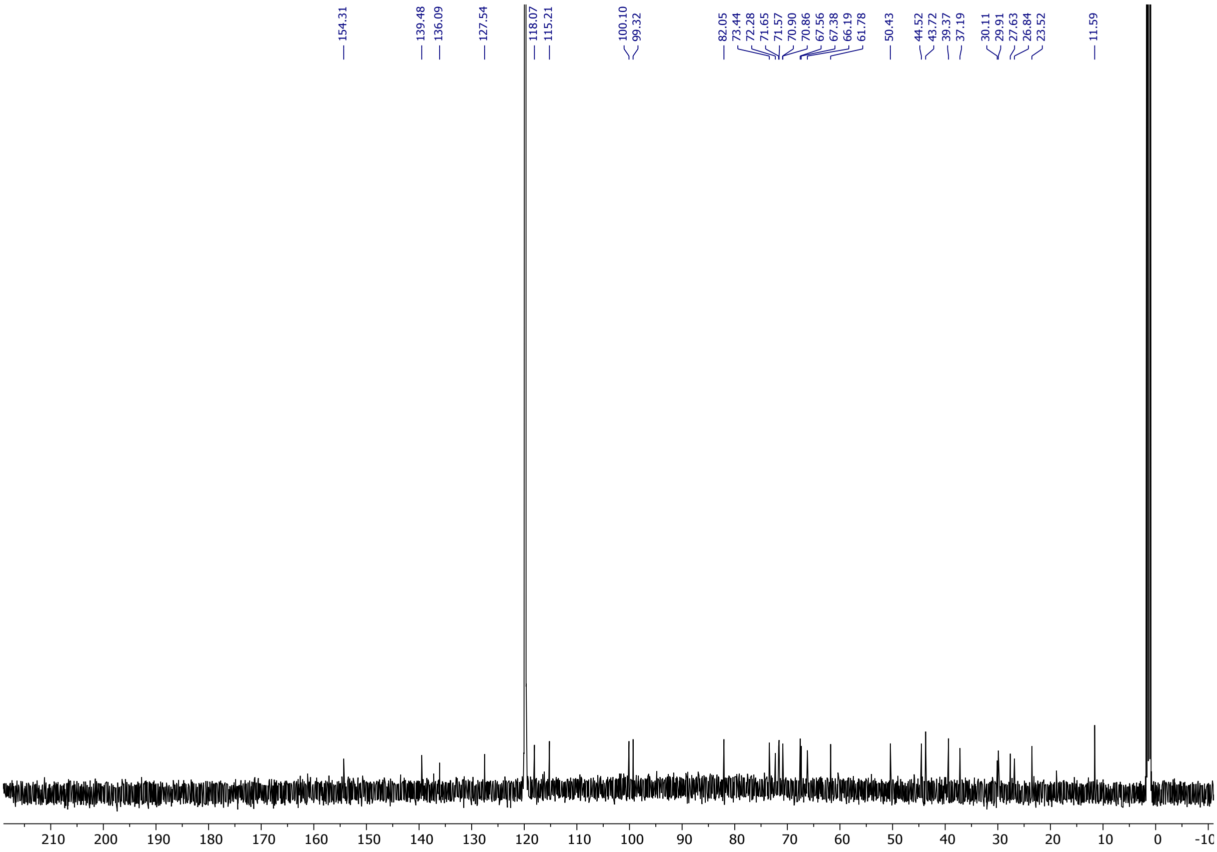
154.31
139.48
136.09
127.54
118.07
115.21
100.10
99.32
82.05
73.44
72.28
71.65
71.57
70.90
70.86
67.56
67.38
66.19
61.78
50.43
44.52
43.72
39.37
37.19
30.11
29.91
27.63
26.84
23.52
11.59

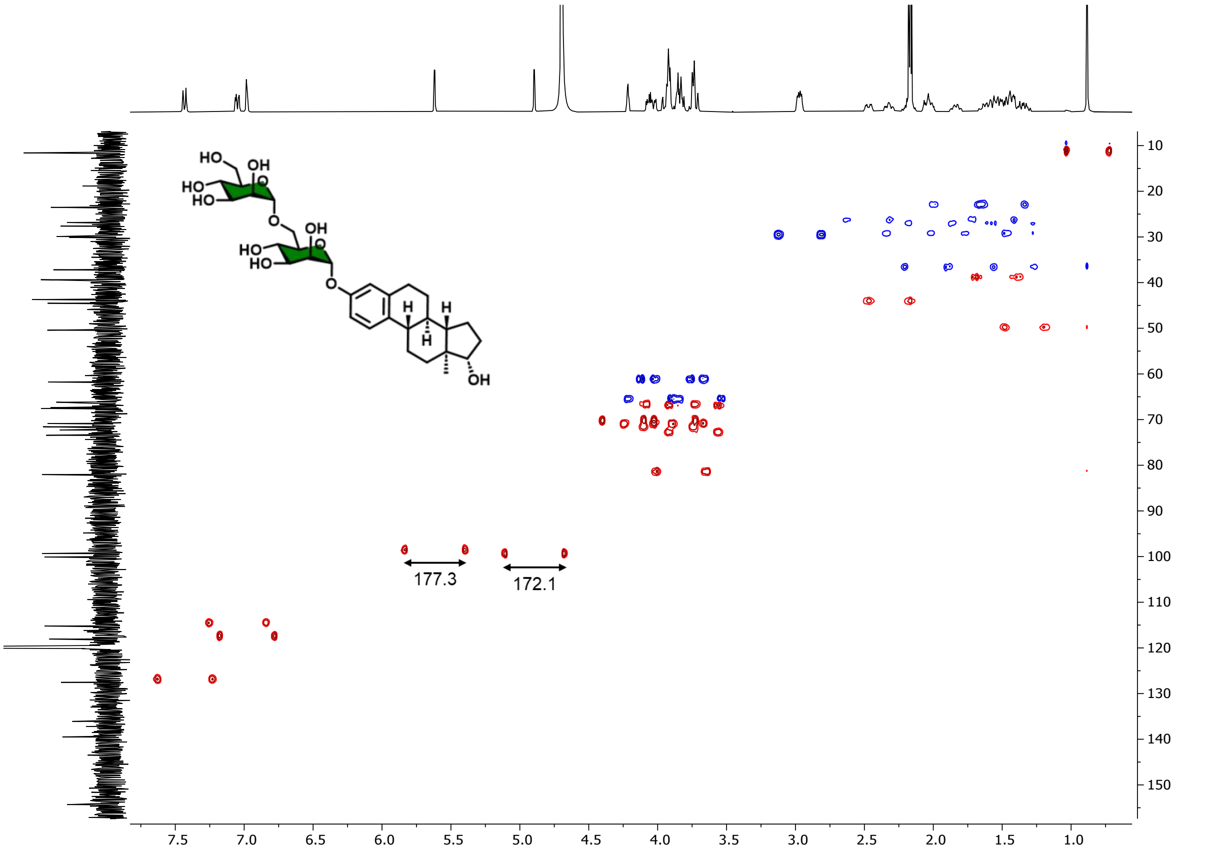
HO
OH
HO
HO
O
OH
HO
HO
O
H
H
H
OH
177.3
172.1

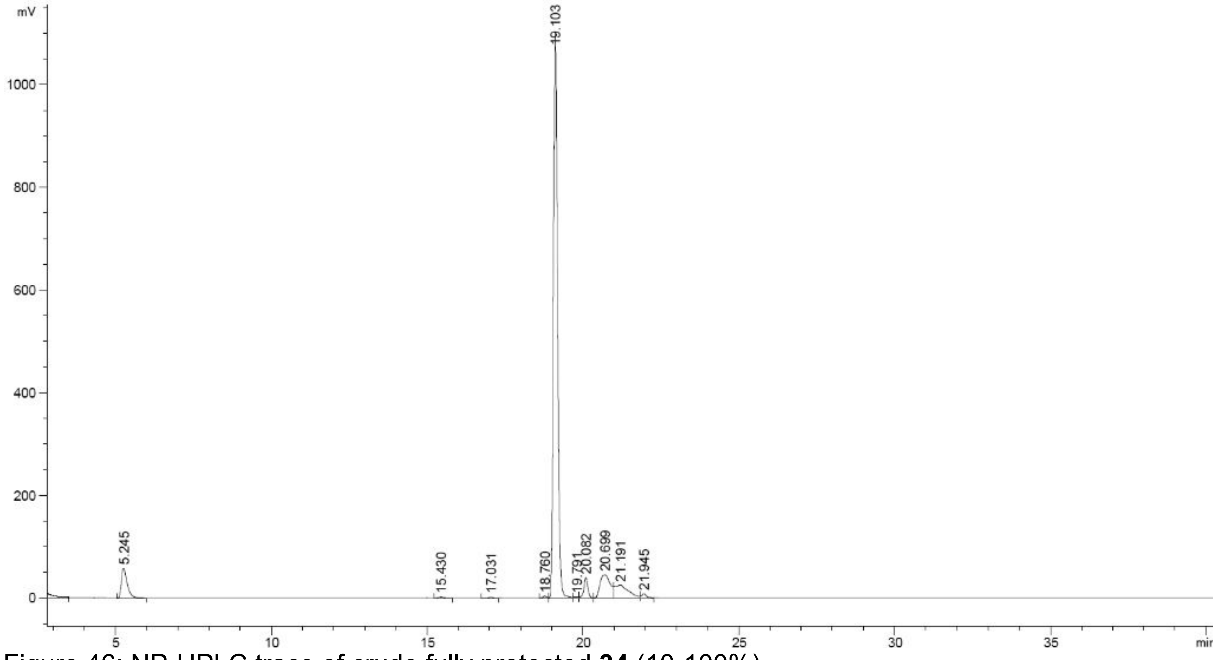

Figure 46: NP-HPLC trace of crude fully protected **34** (10-100%).

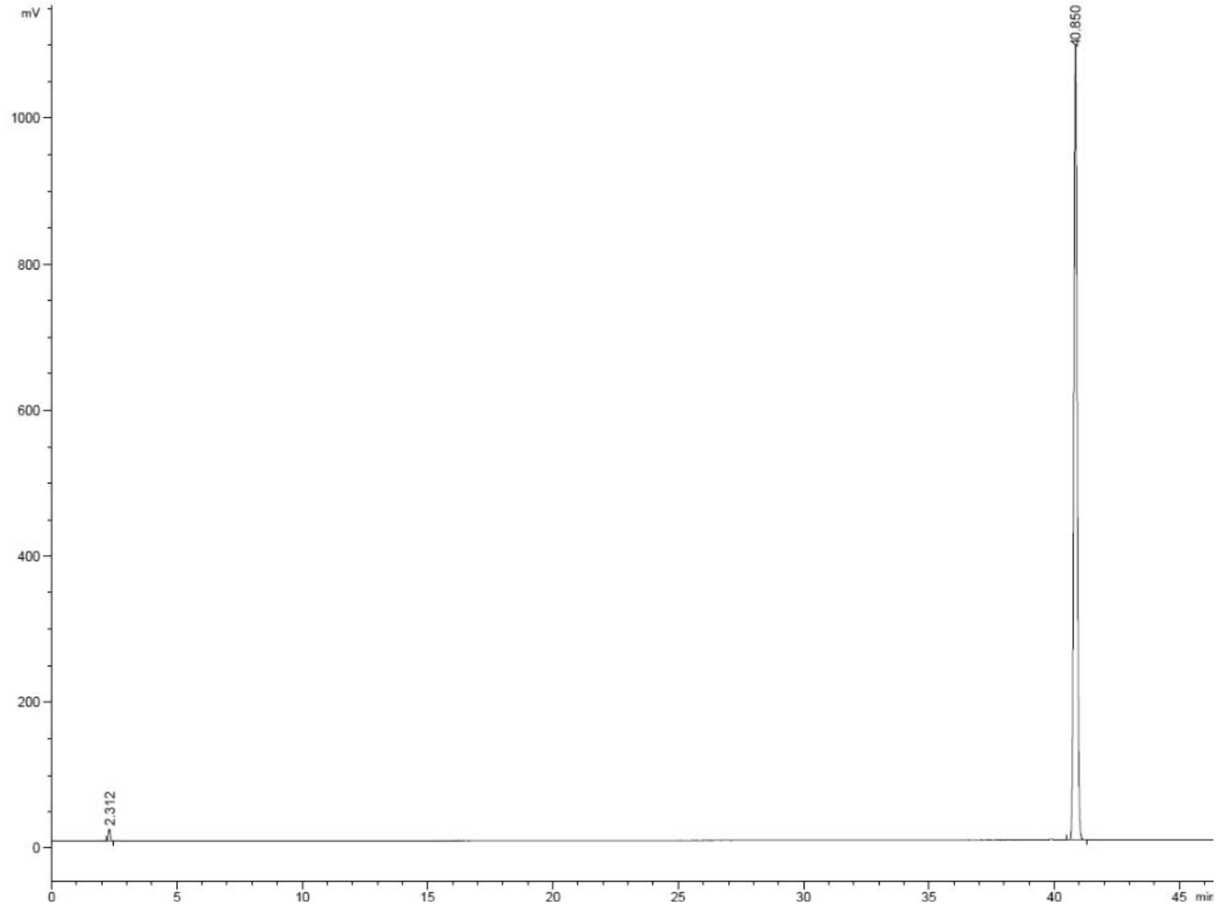

Figure 47: RP-HPLC trace of pure **34** (C_{18}; 0-40%).

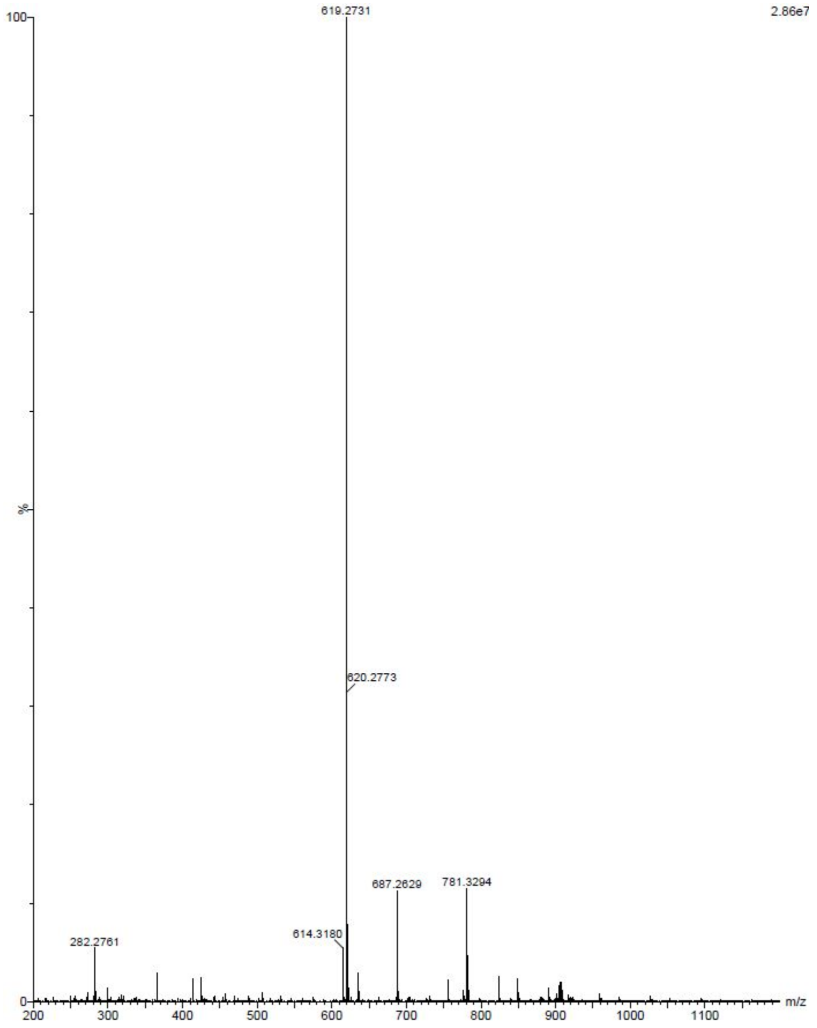

Figure 48: Q-TOF MS-spectrum of **34**.

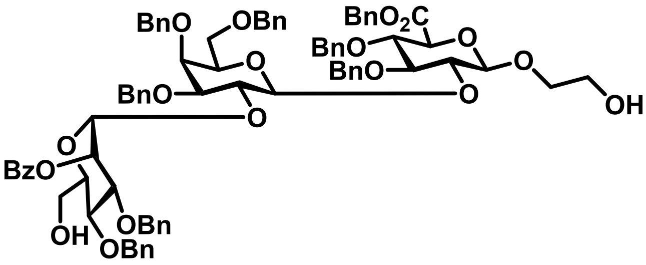

3,4-di-O-benzyl-2-benzoyl-6-OH-*α*-D-mannopyranosyl-(1→2)-3,4,6-tri-O-benzyl--*β*-D-galactopyranosyl-(1→2)-3,4,6-tri-O-benzoyl-*β*-D-glucuronosyl ethylene glycol (35)

Step	Building Block	Modules	Notes
AGA	BB **8**, 10 eq.	**A** – first coupling	-40°C (T1) 5 min<br>-20°C (T2) 35 min
		**B** – RV Wash<br>**C** – Acidic Wash<br>**D** – Capping	
	double cycle<br>BB **36**, 6 eq.	**E** – Fmoc Deprotection<br>**C** – Acidic Wash<br>**G** – Thioglycoside Coupling	-40°C (T1) 20 min<br>0 °C (T2) 20 min
	BB **37**, 6 eq.	**E2** – Lev Deprotection<br>**C** – Acidic Wash<br>**G** – Thioglycoside Coupling	-40°C (T1) 20 min<br>-10°C (T2) 30 min
	BB **7**, 6 eq.	**E** – Fmoc Deprotection **Et_3N**<br>**C** – Acidic Wash<br>**G** – Thioglycoside Coupling	-20°C (T1) 10 min<br>0°C (T2) 20 min
**Post AGA**		**J** – Batch Photocleavage	16 h
**Purification**		**L** – NP-(10-100% in 40 min)	R_t = 22.8 min

After a procedure including automated glycan assembly, photo-cleavage, purification, and lyophilization, **35** obtained as a translucent resin (11.9 mg, 8.6 µmol, 43%).

R_t (NP - 10 – 100%) = 22.8 min.

¹H NMR (400 MHz, $CDCl_3$) *δ* 8.07 – 8.01 (m, 2H), 7.63 – 7.54 (m, 1H), 7.44 (t, *J* = 7.7 Hz, 2H), 7.40 – 7.05 (m, 40H), 5.61 – 5.56 (m, 1H, H2''), 5.55 (d, *J* = 1.7 Hz, 1H, *α*-H1-Man), 5.23 (d, *J* = 12.2 Hz, 1H, CH_2-benzyl ester), 5.15 (d, *J* = 12.2 Hz, 1H, CH_2-benzyl ester), 4.98 – 4.81 (m, 5H), 4.61 (dd, *J* = 20.9, 9.8 Hz, 4H), 4.52 (d, *J* = 11.9 Hz, 1H), 4.49 – 4.40 (m, 5H), 4.37 (d, *J* = 11.8 Hz, 1H), 4.16 (dt, *J* = 9.4, 3.0 Hz, 1H), 4.09 (dd, *J* = 10.0, 7.8 Hz, 1H), 4.04 – 3.94 (m, 3H), 3.93 – 3.83 (m, 5H), 3.66 – 3.49 (m, 8H), 3.38 (dd, *J* = 10.0, 2.7 Hz, 1H), 2.27 (s, 2H, 2x OH).

¹³C NMR (101 MHz, $CDCl_3$) δ 168.5 (CO-GlcA), 165.7 (CO-Bz), 138.8, 138.6, 138.3, 138.1, 137.8, 137.7, 137.2, 135.1, 133.3, 130.1, 129.9, 128.8, 128.7, 128.6, 128.6, 128.5, 128.5, 128.4, 128.3, 128.3, 128.2, 128.2, 128.1, 128.0, 128.0, 127.8, 127.7, 127.7, 127.6, 127.5, 103.1 (*β*-C1), 102.7 (*β*-C1), 97.3 (*α*-C1), 84.2, 80.7, 79.7, 77.9, 75.9, 75.2, 74.8, 74.6, 74.6, 74.4, 74.1, 73.8, 73.7, 72.9, 72.8, 72.6, 71.9, 71.2, 69.2, 68.8, 67.5, 61.7, 61.7.

HRMS (ESI): $C_{83}H_{86}NaO_{19}$ $[M+Na]^+$; calculated: 1409.5661, found: 1409.5601.

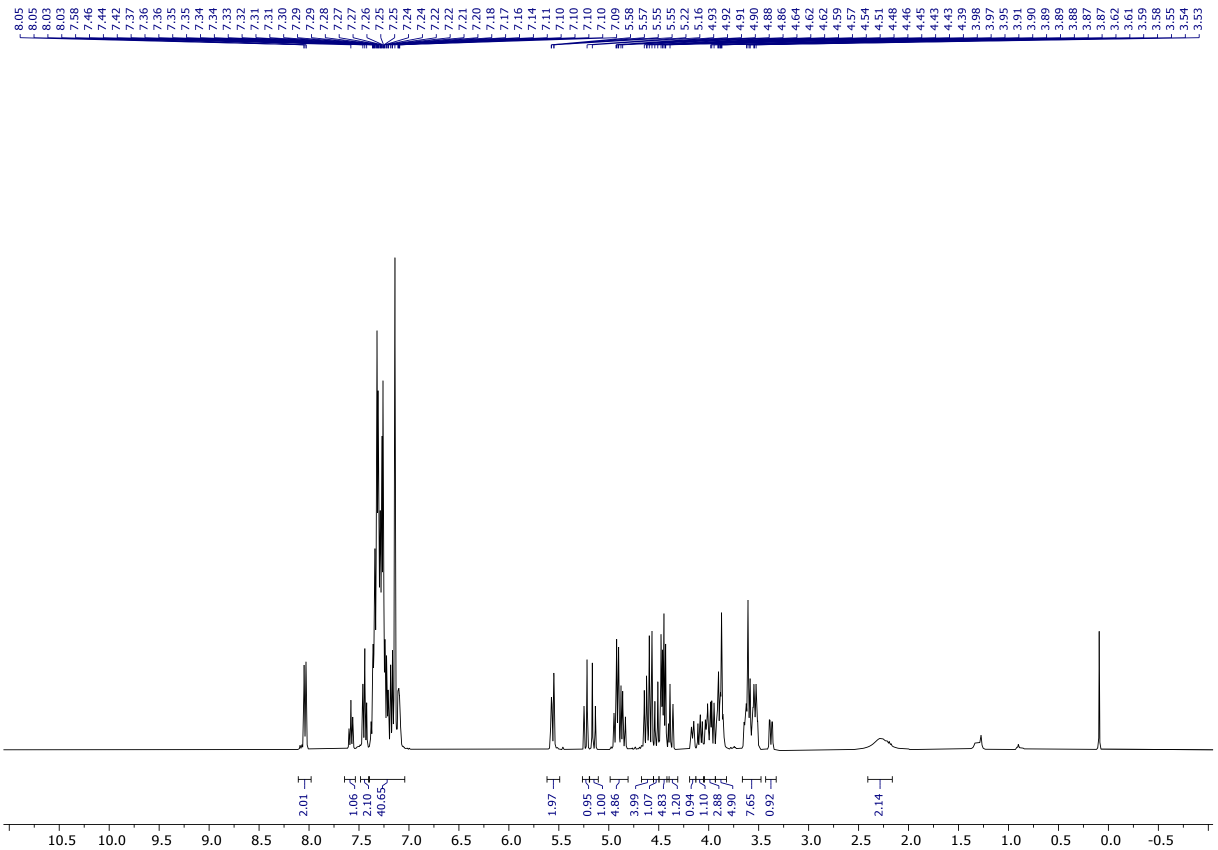

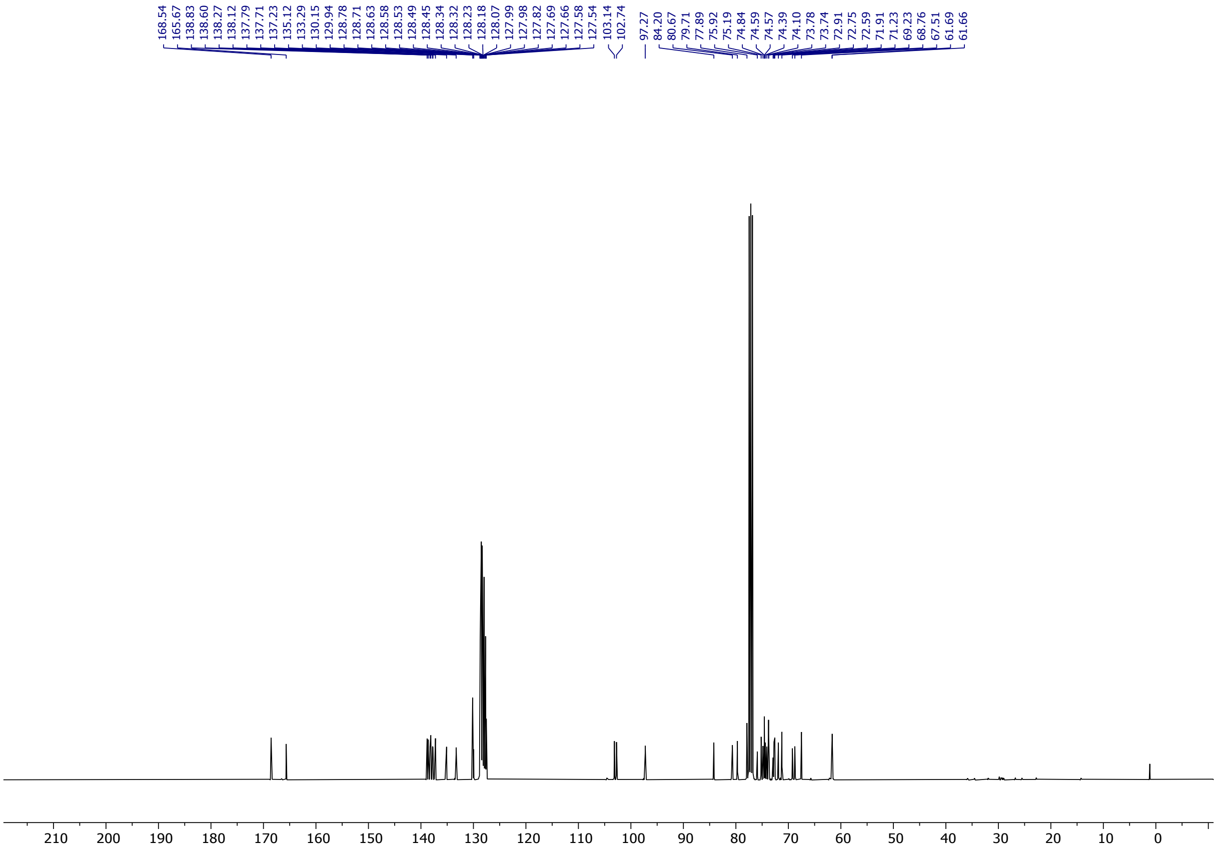
168.54
165.67
138.83
138.60
138.27
138.12
137.79
137.71
137.23
135.12
133.29
130.15
129.94
128.78
128.71
128.63
128.58
128.53
128.49
128.45
128.34
128.32
128.23
128.18
128.07
127.99
127.98
127.82
127.69
127.66
127.58
127.54
103.14
102.74
97.27
84.20
80.67
79.71
77.89
75.92
75.19
74.84
74.59
74.57
74.39
74.10
73.78
73.74
72.91
72.75
72.59
71.91
71.23
69.23
68.76
67.51
61.69
61.66

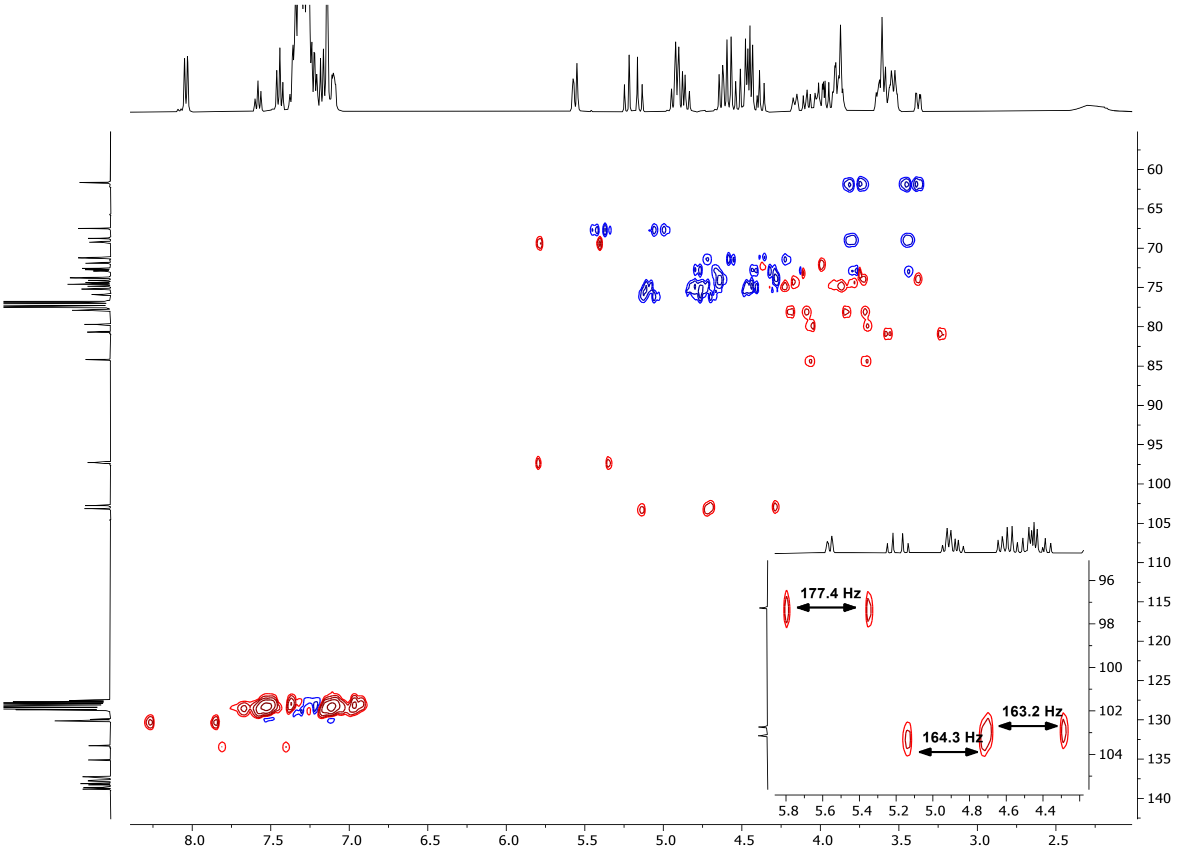
177.4 Hz
164.3 Hz
163.2 Hz

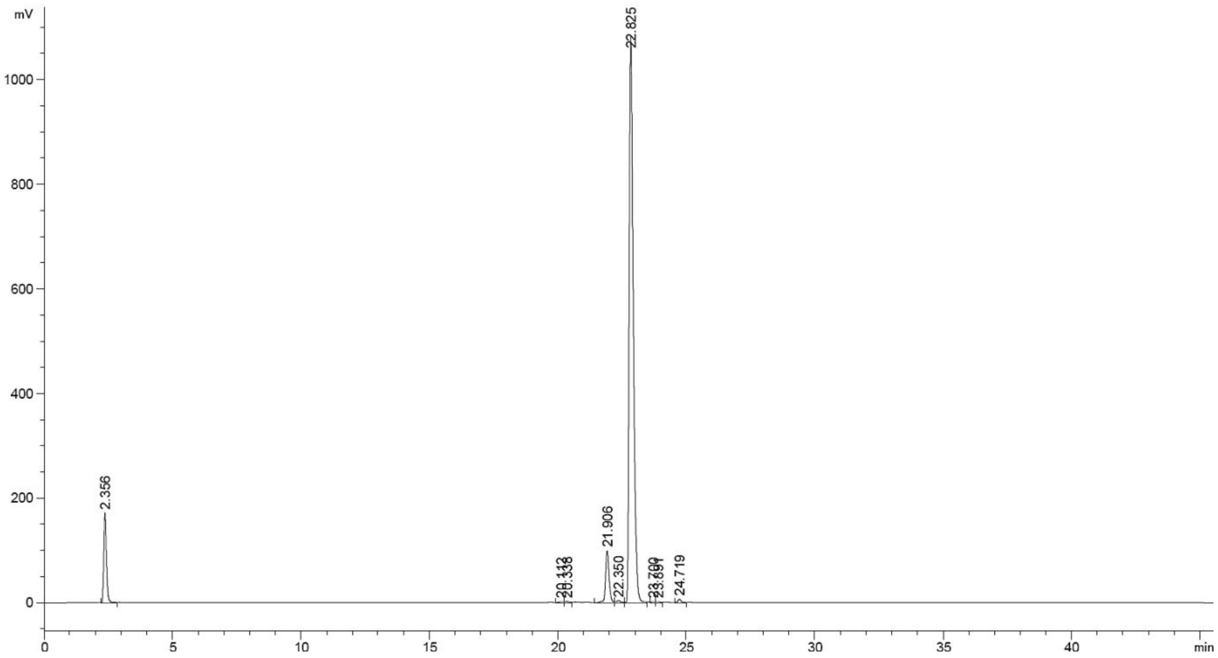

Figure 49: NP-HPLC trace of crude **35** (10-100%).

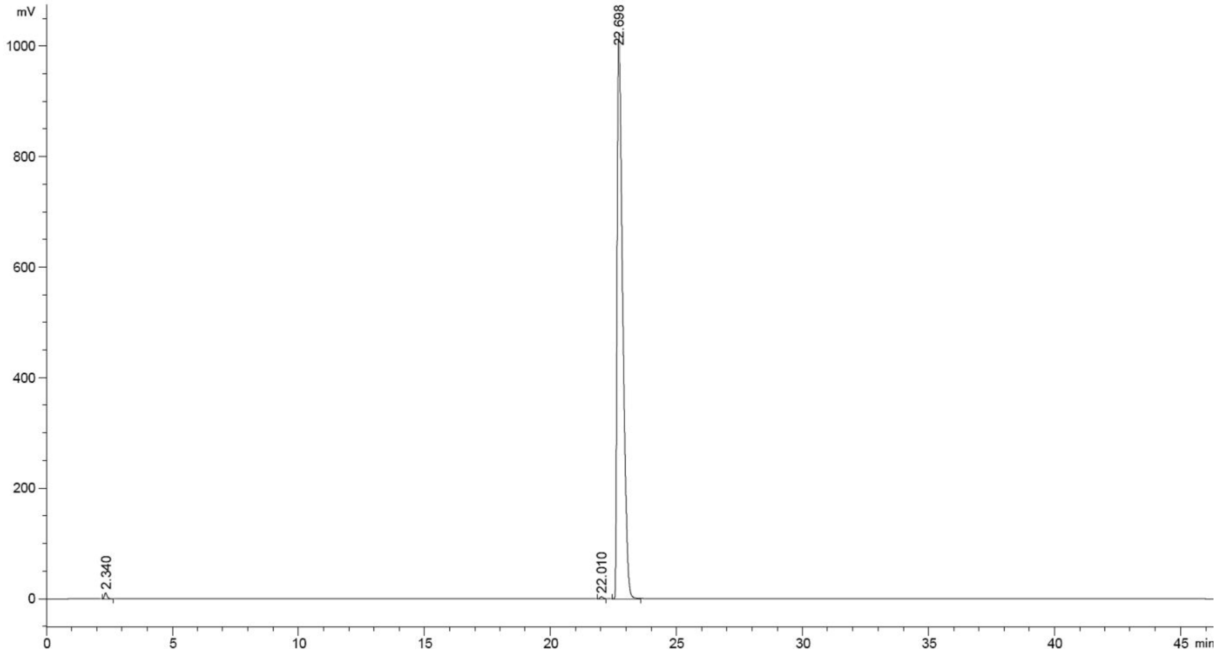

Figure 50: NP-HPLC trace of pure **35** (10-100%).

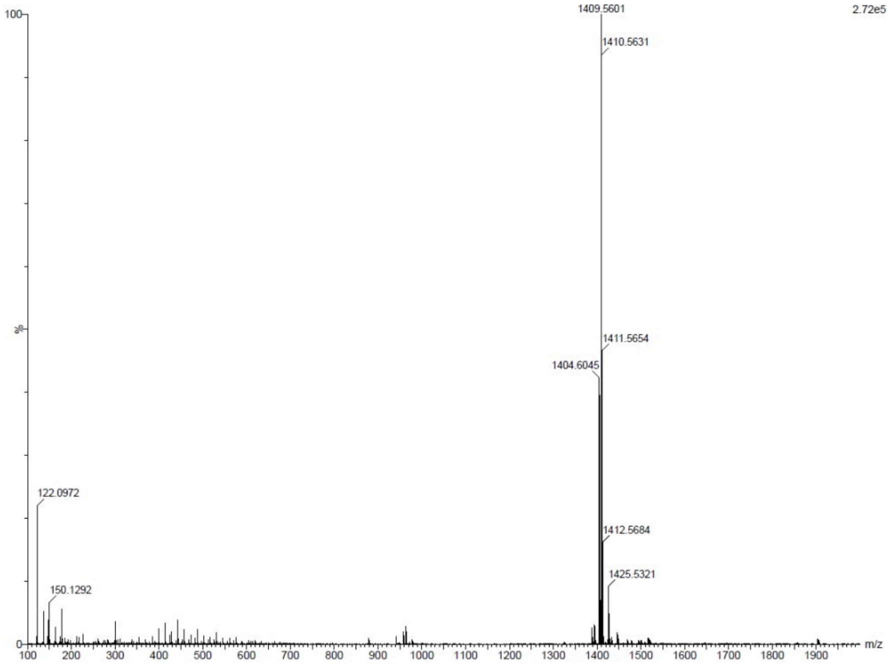

Figure 51: Q-TOF MS-spectrum of **35**.

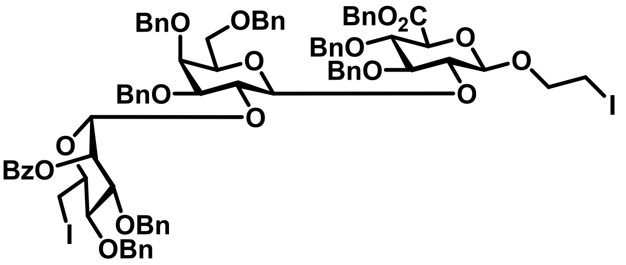

2-O 3,4-Di-O-benzyl-2-benzoyl-6-iodo-*α*-D-mannopyranosyl-(1→2)-3,4,6-tri-O-benzyl-*β*-D-galactopyranosyl-(1→2)-3,4,6-tri-O-benzoyl-*β*-D-glucuronosyl 1-iodo ethane (SI33)

PPh_3 (19.4 mg, 74.8 µmol, 4 equiv.) and iodine (19 mg, 74.8 µmol, 4 equiv.) were added to Di-hydroxyl starting material **35** (26 mg, 18.7 µmol) in 2 mL anhydrous THF under argon. The reaction was heated to 50 °C before imidazole (7.6 mg, 112 µmol, 6 equiv.) were added and the reaction was heated to 66°C. After 4 h the reaction was still incomplete (with roughly 20% remaining as the mono-iodide). The reaction was diluted with EtOAc and washed with citric acid solution (10% w/v). the aqueous phase was extracted with EtOAc two times and the combined organic phases were dried over Na_2SO_4. The solvent was removed under reduced pressure. Preparative normal phase HPLC gave the title compound in 67% yield (20.0 mg, 12.4 µmol) as a translucent resin.

R_t (NP - 10 – 100%) = 13.0 min.

^{1}H NMR (400 MHz, $CDCl_3$) *δ* 8.13 – 8.06 (m, 2H), 7.60 – 7.48 (m, 1H), 7.40 (t, *J* = 7.8 Hz, 2H), 7.36 – 7.21 (m, 30H), 7.19 – 7.05 (m, 10H), 5.63 (dd, *J* = 3.2, 1.8 Hz, 1H, H2''), 5.55 (d, *J* = 1.8 Hz, 1H, *α*-H1''), 5.19 (d, *J* = 12.2 Hz, 1H, CH_2-benzyl ester), 5.13 (d, *J* = 12.2 Hz, 1H, CH_2-benzyl ester), 4.99 – 4.85 (m, 4H), 4.82 (d, *J* = 10.9 Hz, 1H), 4.66 (dd, *J* = 14.8, 11.0 Hz, 2H), 4.61 – 4.54 (m, 3H), 4.53 – 4.36 (m, 6H), 4.12 – 3.99 (m, 3H), 3.99 – 3.91 (m, 2H), 3.85 (tdd, *J* = 8.8, 7.2, 2.3 Hz, 4H), 3.80 – 3.71 (m, 2H), 3.64 (dd, *J* = 9.0, 7.6 Hz, 1H), 3.57 (dd, *J* = 9.1, 5.4 Hz, 1H), 3.50 (dd, *J* = 7.4, 5.7 Hz, 1H), 3.35 (dd, *J* = 10.0, 2.7 Hz, 1H), 3.20 – 3.09 (m, 4H, iodoethanyl).

^{13}C NMR (101 MHz, $CDCl_3$) δ 168.8 (CO-GlcA), 165.7 (CO-Bz), 138.9, 138.2, 138.0, 137.9, 137.9, 137.2, 135.2, 133.2, 130.3, 129.9, 128.7, 128.7, 128.6, 128.6, 128.6, 128.6, 128.5, 128.4, 128.4, 128.4, 128.2, 128.0, 128.0, 128.0, 127.9, 127.8, 127.8, 127.7, 127.7, 127.6, 127.6, 102.8 (*β*-C1), 102.1 (*β*-C1), 97.4 (*α*-C1), 84.3, 81.1, 79.3, 78.6, 77.7, 77.4, 75.9, 75.6, 74.9, 74.7, 74.7, 73.8, 73.5, 73.0, 72.5, 71.2, 70.3, 69.1, 68.8, 68.6, 67.4, 10.5 (mannose-C6H_2-I), 2.5 (inker-CH_2-I).

HRMS (ESI): $C_{83}H_{84}I_2NaO_{17}$ $[M+Na]^+$; calculated: 1629.3696, found: 1629.3672.

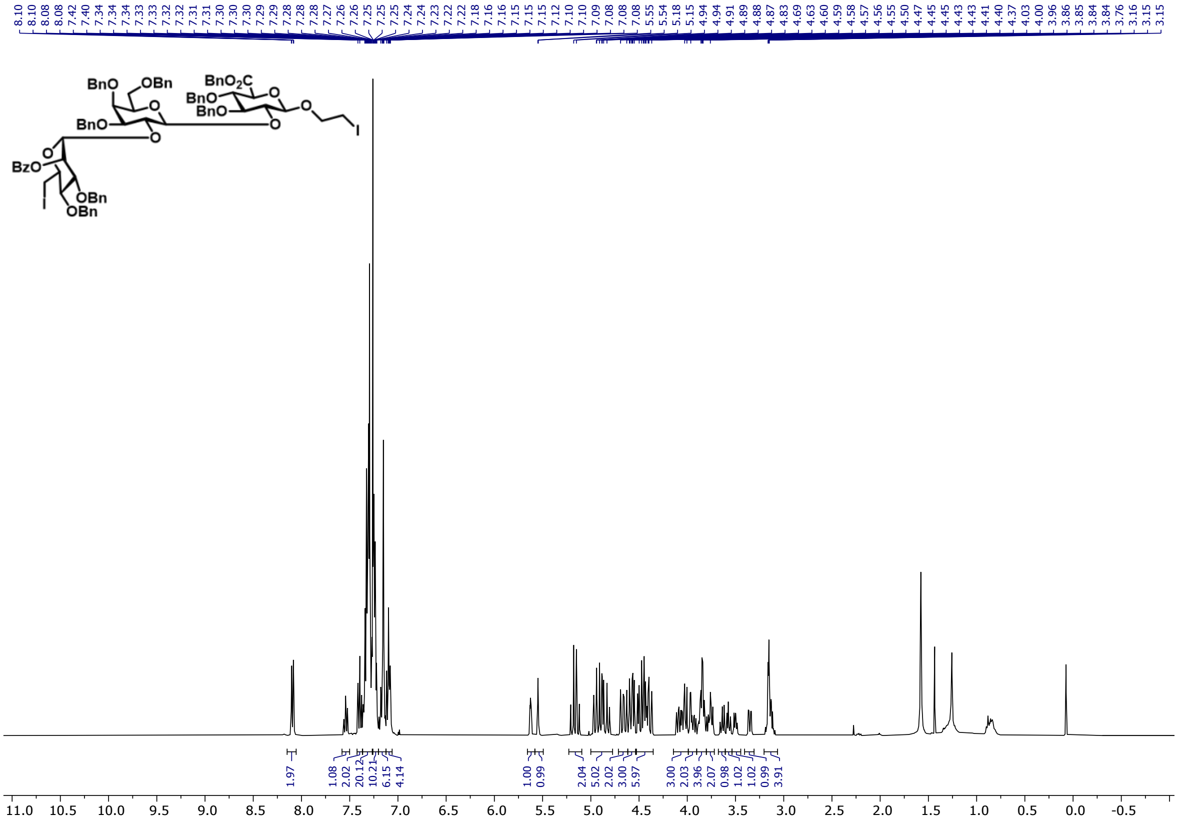

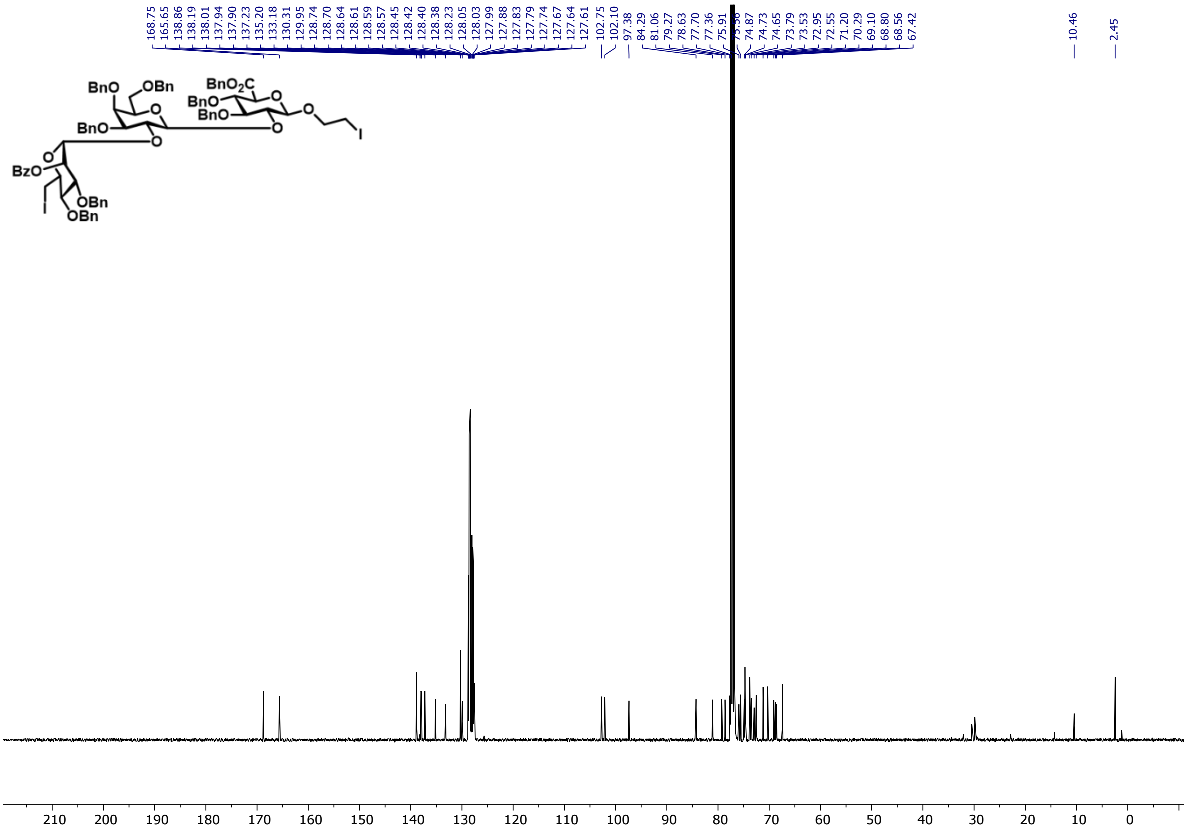

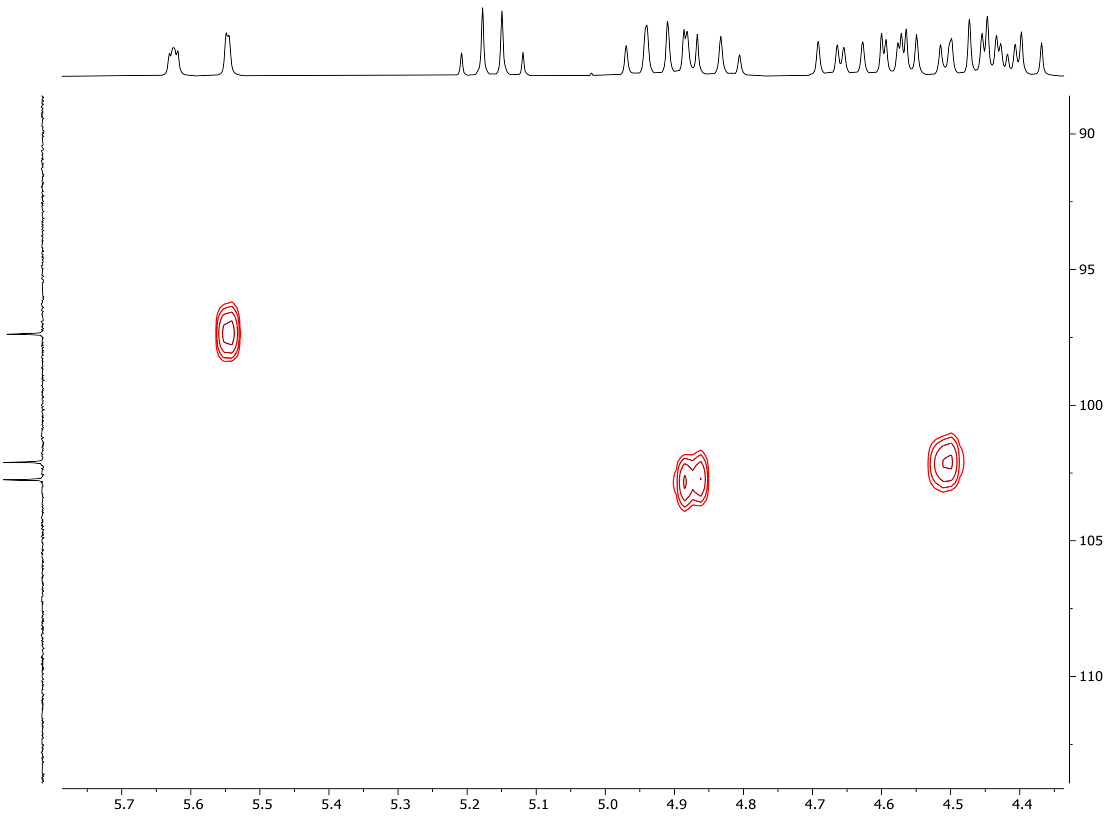

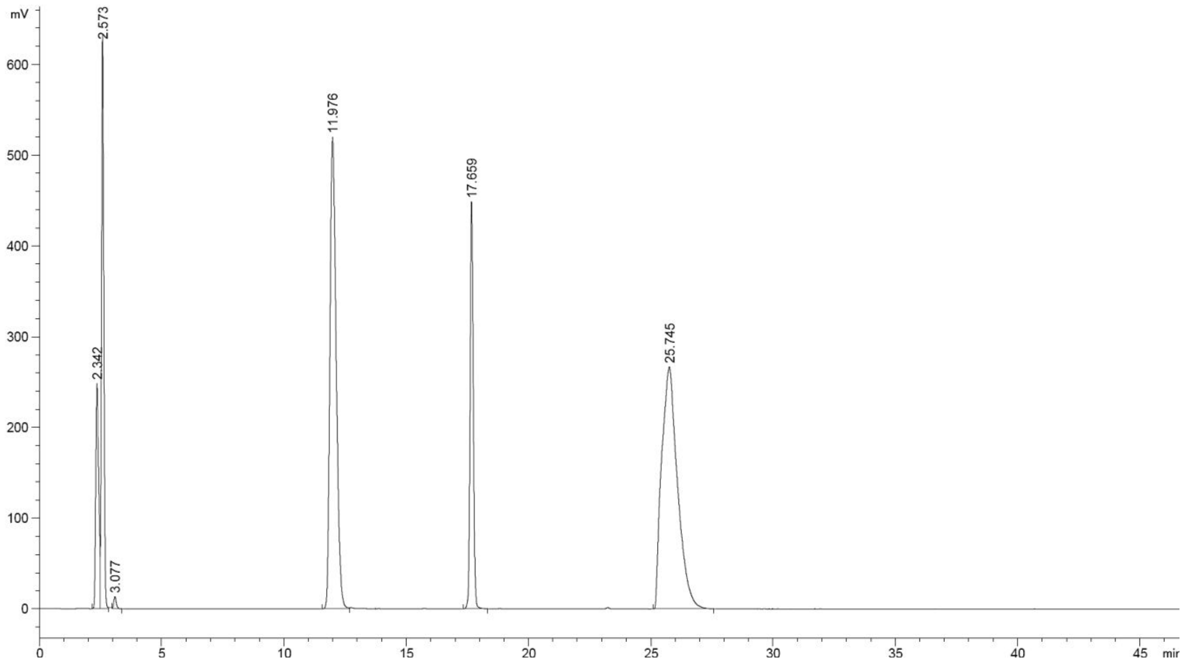

Figure 52: NP-HPLC trace of crude **SI33** (10-100%).

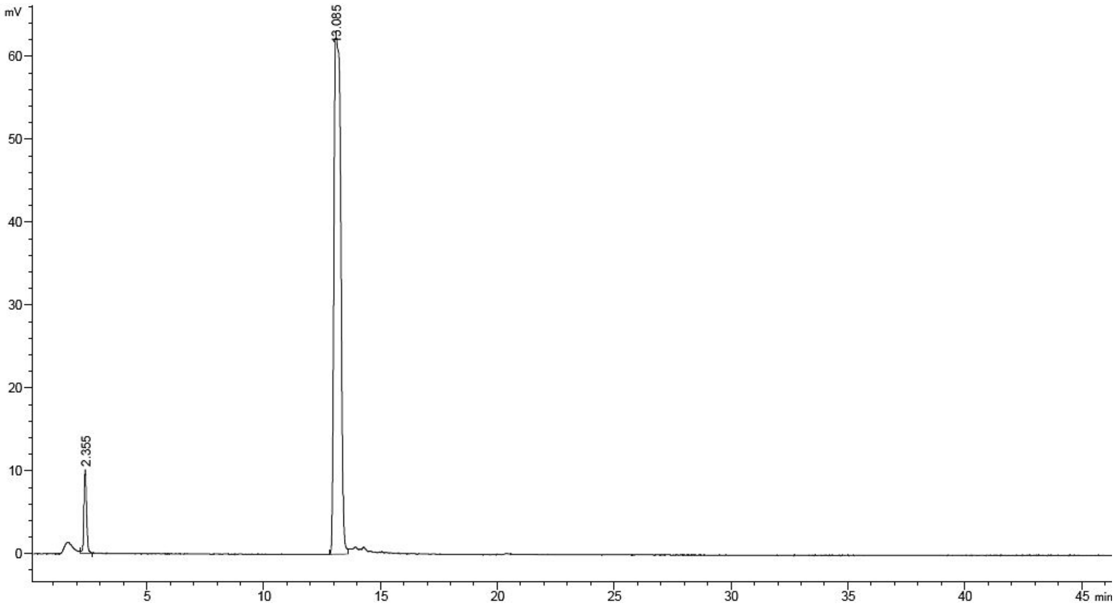

Figure 53: NP-HPLC trace of pure **SI33** (10-100%).

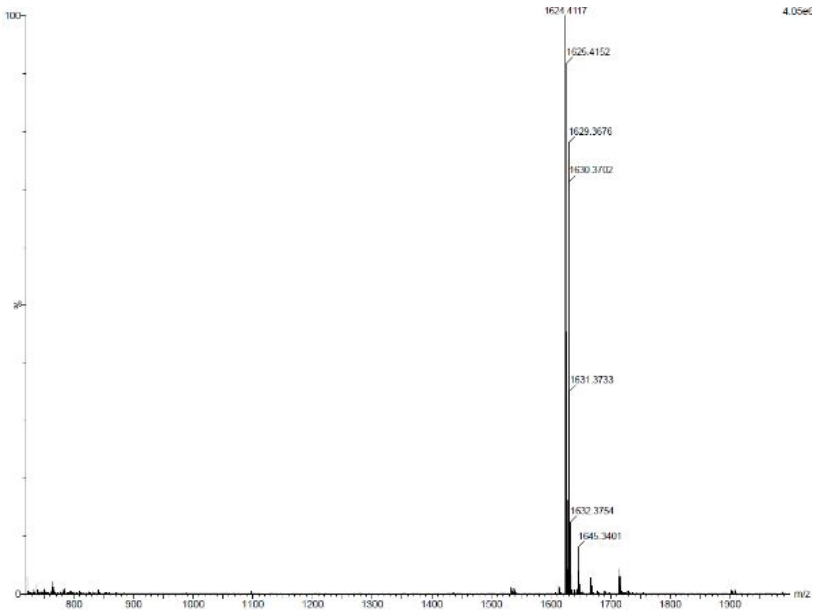

Figure 54: Q-TOF MS-spectrum of **SI33**.

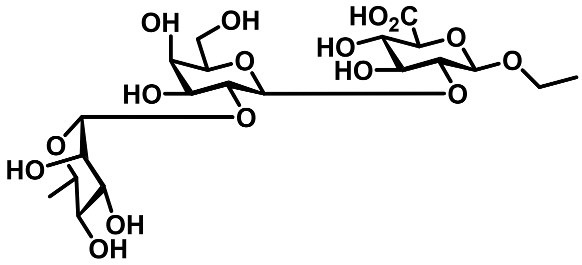

Ethyl *α*-D-rhamnopyranosyl-(1→2)-*β*-D-galactopyranosyl-(1→2)-*β*-D-glucuronopyranosyl (38)

Di-iodide **SI33** (20 mg, 12.4 μmol) was dissolved in *t*BuOH/EtOAc/H_2O (2:1:1) before 50 mg Pd(OH)$_2$/C (10%) was added and the suspension was stirred under an atmosphere of 8 bars of H_2. The reaction was filtered over a pad of celite and the solvent was removed under vacuum.

The crude was dissolved in THF/H_2O (1:2, 4 mL), cooled to 0 °C and added 1 M LiOH solution (124 μL, 10 equiv.) and H_2O_2 solution (30% in H_2O, 190 μL, 150 equiv.). The reaction was allowed to warm to 23 °C over 30 min and stirred at that temperature for another 90 min before it was diluted with 5 mL of H_2O and neutralized by the addition of Amberlite IR-120 (H).

The solvent was removed under reduced pressure after filtration. Preparative reverse phase HPLC gave **38** in 69% yield (4.51 mg, 8.5 μmol) as a white powder.

R_t (Hypercarb - 0 – 30%) = 28.9 min.

^{1}H NMR (600 MHz, D_2O) *δ* 5.25 (d, *J* = 1.8 Hz, 1H, *α*-H1''), 4.91 (d, *J* = 7.7 Hz, 1H, *β*-H1-GlcA), 4.62 (d, *J* = 7.8 Hz, 1H, *β*-H1'-Gal), 4.07 (dd, *J* = 3.5, 1.8 Hz, 1H), 4.06 – 4.02 (m, 1H), 4.01 – 3.96 (m, 1H), 3.94 (dd, *J* = 3.5, 1.0 Hz, 1H), 3.83 – 3.79 (m, 2H), 3.77 – 3.71 (m, 3H), 3.71 – 3.68 (m, 2H), 3.67 – 3.60 (m, 3H), 3.53 (t, *J* = 9.5 Hz, 1H), 3.46 (t, *J* = 9.7 Hz, 1H), 1.29 (d, *J* = 6.3 Hz, 3H, H6''-CH_3), 1.25 (t, *J* = 7.1 Hz, 3H, CH_3-ethyl).

^{13}C NMR (151 MHz, D_2O) *δ* 175.6 (carboxylic acid), 102.6 (*β*-C1-GlcA), 100.2 (*β*-C1'-Gal), 100.2 (*α*-C1''), 78.3, 76.7, 76.4, 75.9, 75.0, 72.1, 72.0, 71.5, 70.1, 70.1, 69.0, 7.78, 65.8, 60.84, 16.5 (C6''-CH_3), 14.1 (CH_3-ethyl).

HRMS (ESI): $C_{20}H_{33}O_{16}$ [M-H]$^-$; calculated: 529.1774, found: 529.1774.

Optical rotation: $[\alpha]_D^{25}$ = +52.0° (c = 0.1, H_2O/MeCN 1:1)

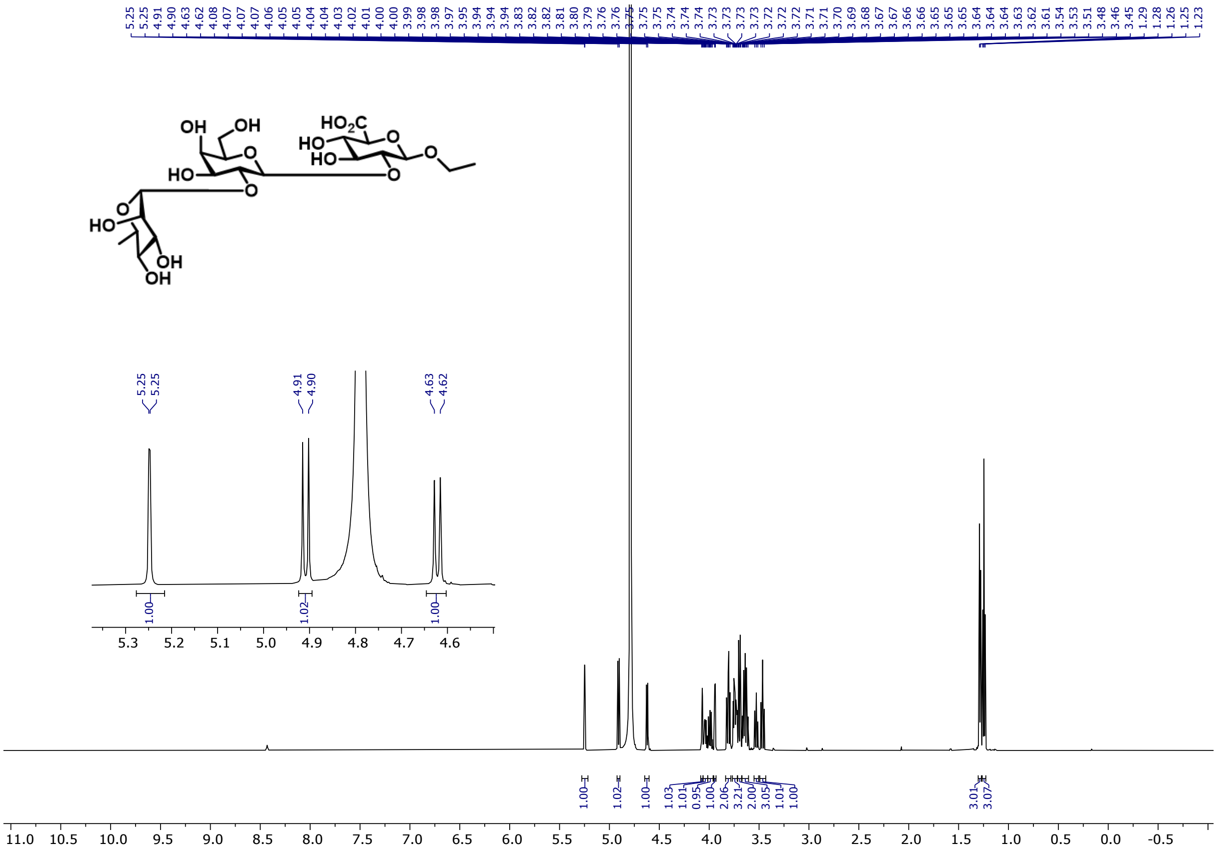

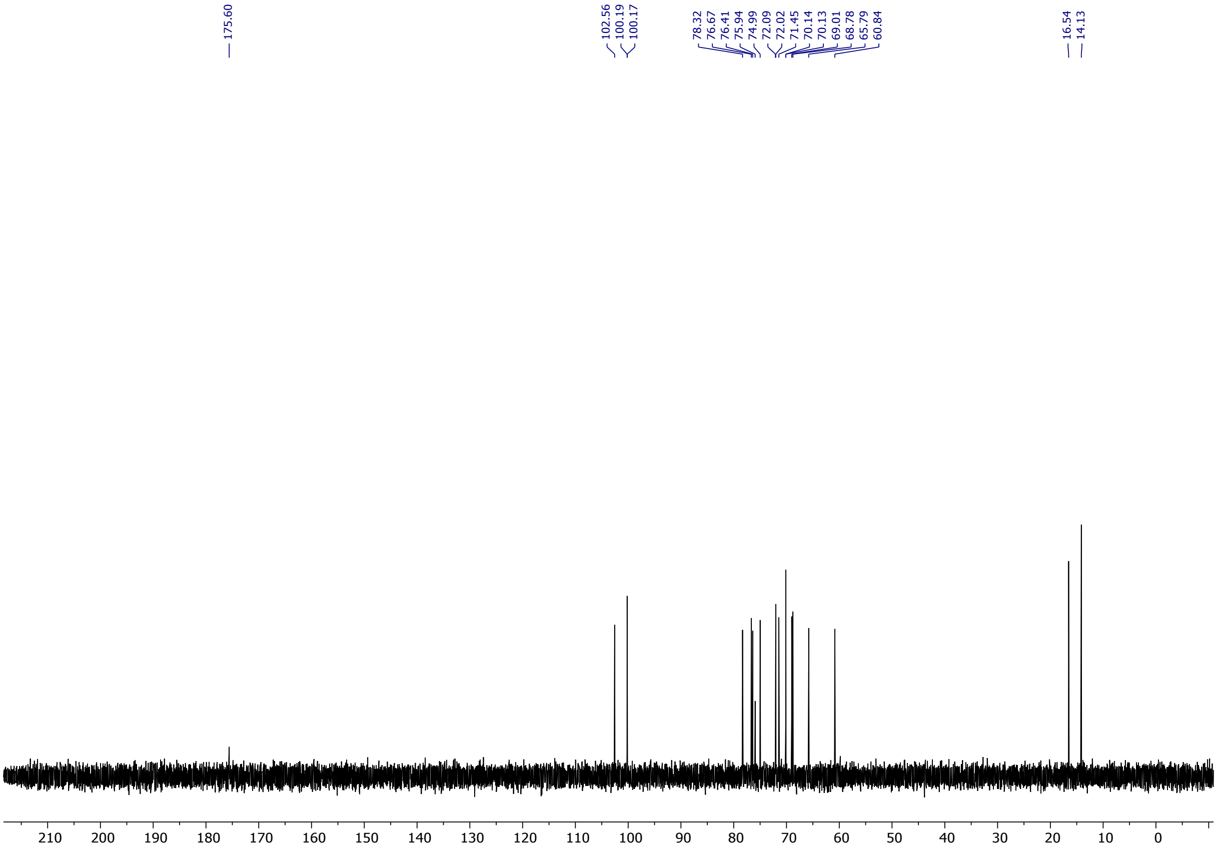

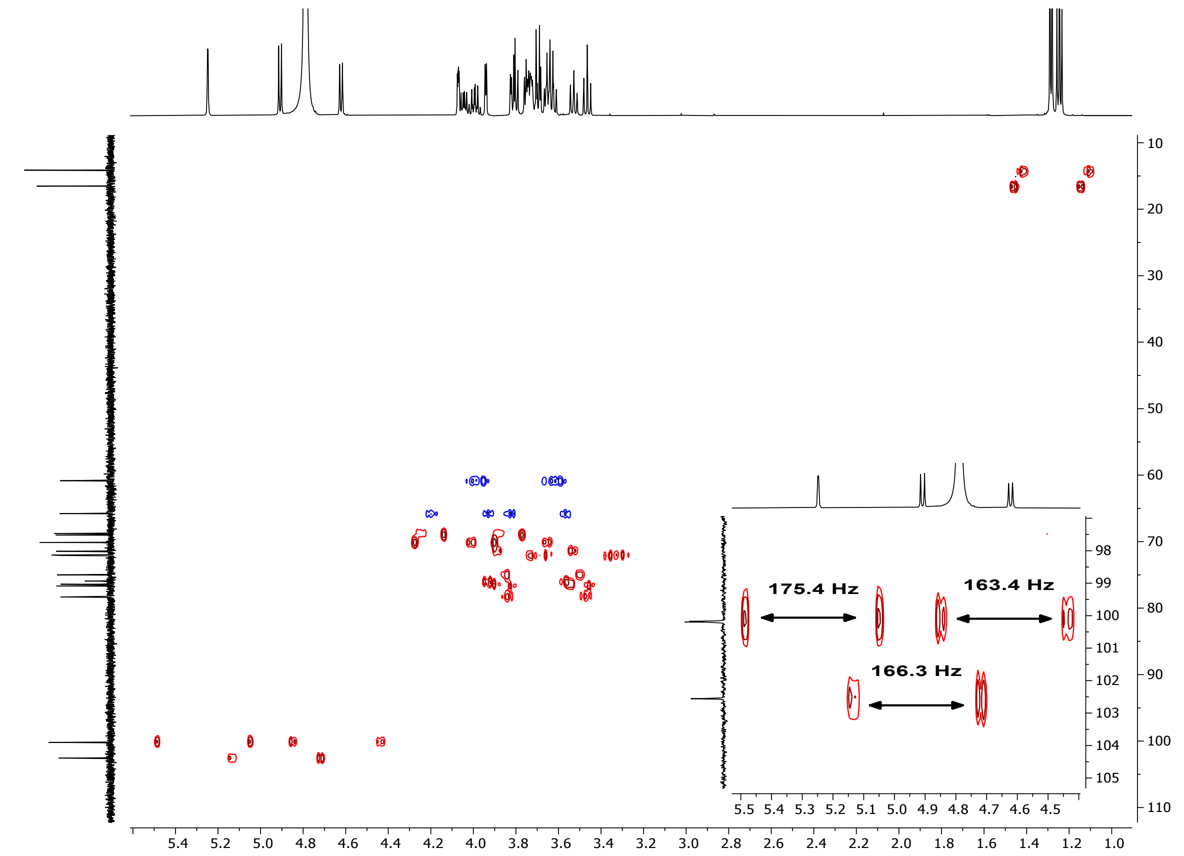
175.4 Hz
163.4 Hz
166.3 Hz
5.5 5.4 5.3 5.2 5.1 5.0 4.9 4.8 4.7 4.6 4.5
98 99 100 101 102 103 104 105
10 20 30 40 50 60 70 80 90 100 110
5.4 5.2 5.0 4.8 4.6 4.4 4.2 4.0 3.8 3.6 3.4 3.2 3.0 2.8 2.6 2.4 2.2 2.0 1.8 1.6 1.4 1.2 1.0

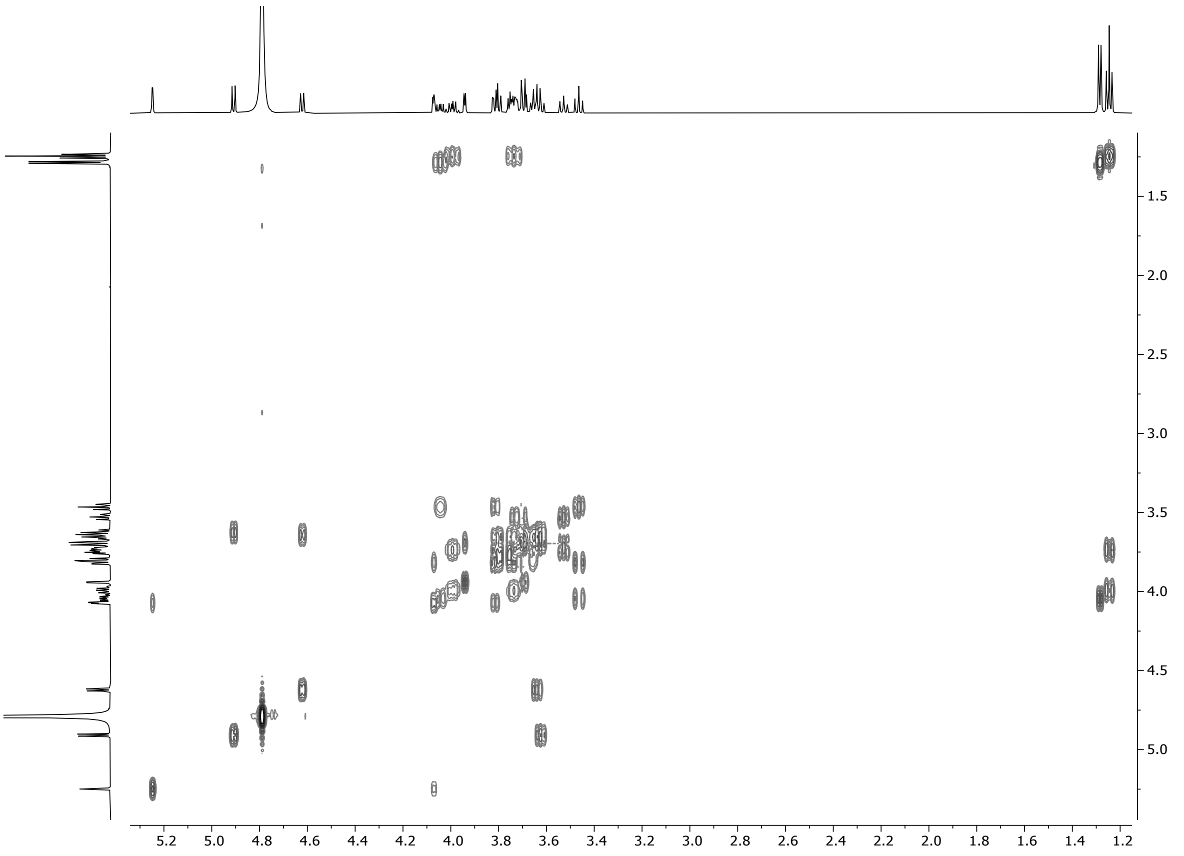
1.5 2.0 2.5 3.0 3.5 4.0 4.5 5.0
5.2 5.0 4.8 4.6 4.4 4.2 4.0 3.8 3.6 3.4 3.2 3.0 2.8 2.6 2.4 2.2 2.0 1.8 1.6 1.4 1.2

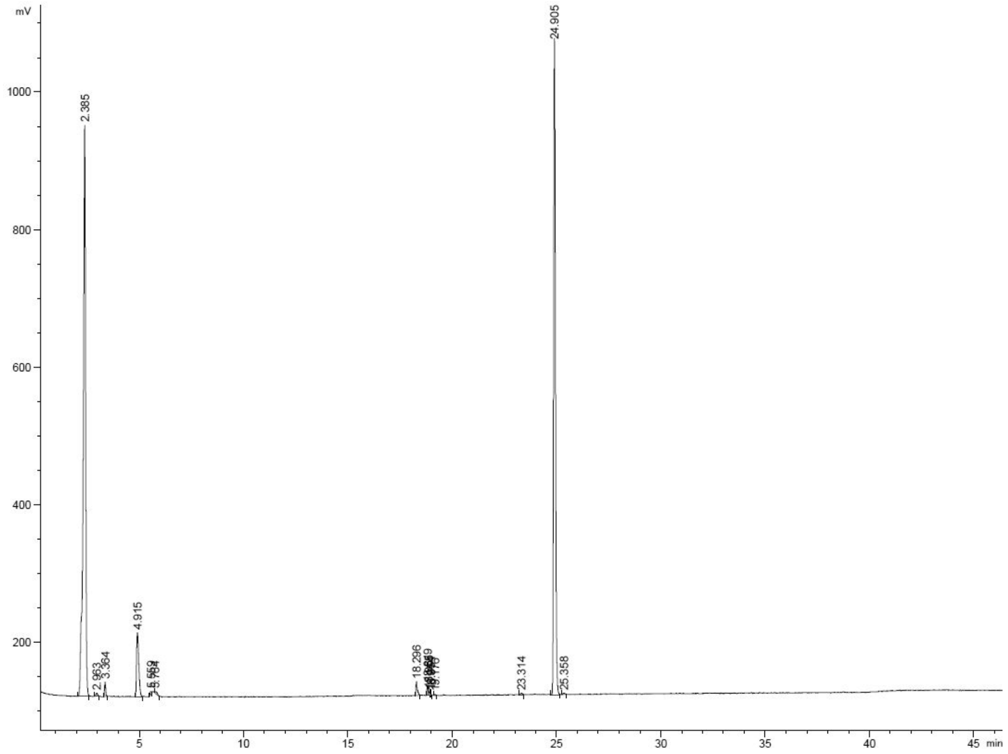

Figure 55: RP-HPLC trace of crude **38** (C_{18}, 0-30%).

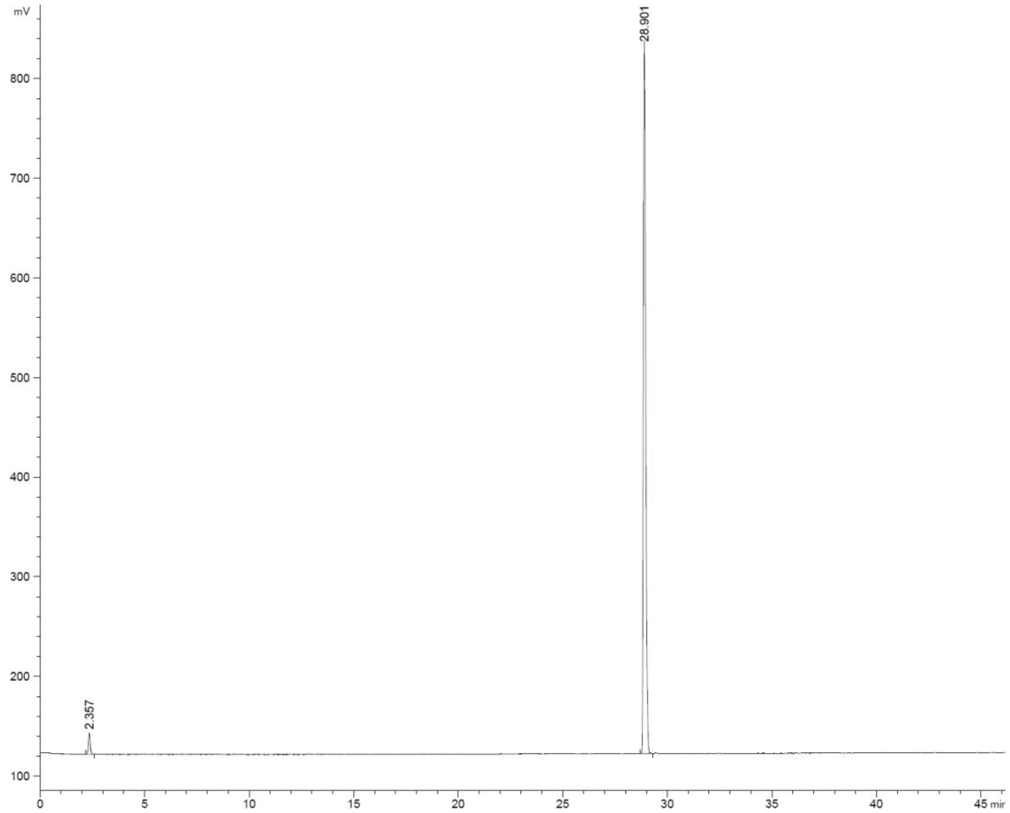

Figure 56: RP-HPLC trace of pure **38** (Hypercarb, 0-30%).

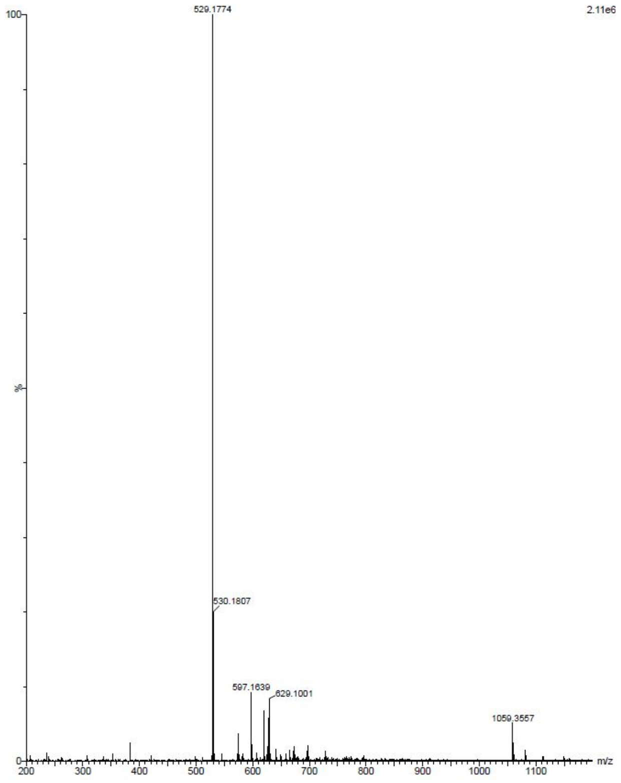

Figure 57: Q-TOF MS-spectrum of **38**.

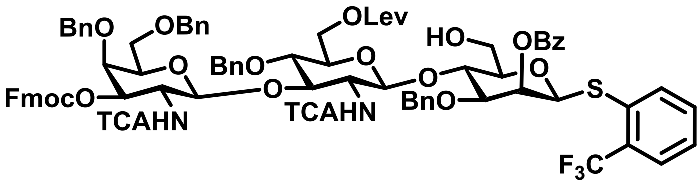

(Trifluoromethyl)phenyl 4,6-di-O-benzyl-3-O-(9-fluorenylmethoxycarbonyl)-2-[(2,2,2,-trichloroacetyl)amino]-*β*-D-galactopyranosyl-(1→3)-4-O-benzyl-6-O-levulinoyl-3-O-(9-fluorenylmethoxycarbonyl)-2-[(2,2,2,-trichloroacetyl)amino]-*β*-D-glucopyranosyl-(1→4)-2-O-benzoyl-3-O-benzyl-1-thio-*β*-D-mannopyranoside (41)

Step	Building Block	Modules	Notes
AGA	BB **9**, 6 eq.	**A** – first coupling	-40°C (T1) 5 min<br>-20°C (T2) 35 min
		**B** – RV Wash	
		**C** – Acidic Wash	
		**D** – Capping	
		**E** – Fmoc Deprotection **Et_3N**	
		**C** – Acidic Wash	
	double cycle<br>BB **39**, 3 eq.	**F** – Phosphate coupling<br>(half the amount of TMSOTf)	-40°C (T1) 15 min<br>-10°C (T2) 20 min
		**E** – Fmoc Deprotection **Et_3N**	
		**C** – Acidic Wash	
	double cycle<br>BB **40**(α), 3 eq.	**F** – Phosphate coupling<br>(half the amount of TMSOTf)	-40°C (T1) 15 min<br>-10°C (T2) 20 min
Post AGA		**J** – Batch Photocleavage	16 h
Purification		**L** – NP-Method (10-100%)	R_t = 24.2 min

The coupling of the first BB to the resin is performed under standard conditions. All further couplings (procedure F) and acidic washes (procedure C) were performed with a TMSOTf concentration half of the standard solution (0.45 mL in 80 mL CH_2Cl_2). Fmoc deprotection was performed according to protocol E using a 20% Et_3N solution and heating to 25°C instead of 50°C.

After a procedure including automated glycan assembly, photo-cleavage, purification and lyophilization, **41** was obtained as a translucent resin (8.78 mg, 5.06 μmol, 25%).

Rt (NP- 10-100%) = 24.2 min.

^{1}H NMR (700 MHz, $CDCl_3$) *δ* 8.09 – 8.06 (m, 2H), 7.78 (d, *J* = 7.8 Hz, 1H), 7.73 (t, *J* = 8.2 Hz, 2H), 7.66 (dd, *J* = 8.0, 1.4 Hz, 1H), 7.60 – 7.55 (m, 3H), 7.52 – 7.49 (m, 1H), 7.46 (t, *J* = 7.7 Hz, 3H), 7.37 (dq, *J* = 18.5, 8.1 Hz, 4H), 7.32 – 7.27 (m, 11H), 7.25 – 7.22 (m, 6H), 7.19 (dd, *J* = 9.2, 6.6 Hz, 5H), 6.07 (d, *J* = 3.5 Hz, 1H, H2-Man), 5.17 (dd, *J* = 11.3, 2.9 Hz, 1H), 5.08 (s, 1H, *β*-H1-Man), 4.97 (d, *J* = 5.4 Hz, 1H, *β*-C1-GlcNTCA), 4.89 (s, 1H), 4.87 (d, *J* = 7.6 Hz, 1H, *β*-H1-GalNTCA), 4.77 (d, *J* = 11.5 Hz, 1H), 4.62 (t, *J* = 11.3 Hz, 2H), 4.47 (d, *J* = 11.5 Hz, 1H), 4.43 (d, *J* = 10.8 Hz, 1H), 4.41 – 4.35 (m, 4H), 4.35 – 4.33 (m, 1H), 4.22 (t, *J* = 7.4 Hz, 1H, CH-Fmoc), 4.19 (dd, *J* = 11.8, 3.2 Hz, 1H), 4.17 – 4.13 (m, 1H), 4.08 – 4.04 (m, 2H), 4.03 – 3.99 (m, 2H), 3.96 (dd, *J* = 11.6, 4.3 Hz, 1H), 3.86 (dd, *J* = 12.2, 4.2 Hz, 1H), 3.79 (dt, *J* = 8.4, 5.7 Hz, 1H), 3.74 (td, *J* = 6.6, 3.3 Hz, 1H), 3.67 (dt, *J* = 9.4, 6.3 Hz, 2H), 3.55 (dq, *J* = 9.6, 4.8 Hz, 1H), 3.50 (dd, *J* = 9.2, 7.1 Hz, 1H), 3.45 (dd, *J* = 9.1, 5.9 Hz, 1H), 2.68 – 2.63 (m, 1H), 2.61 (t, *J* = 6.2 Hz, 2H), 2.44 (dt, *J* = 17.2, 6.3 Hz, 1H, CH_2-Lev), 2.36 (dt, *J* = 17.3, 6.0 Hz, 1H, CH_2-Lev), 2.07 (s, 3H, CH_3-Lev).

^{13}C NMR (176 MHz, $CDCl_3$) δ 206.8 (carbonyl-Lev), 172.5 (carboxyl-Lev), 165.7 (CO-Bz), 162.6 (CO-TCA), 161.5 (CO-TCA), 154.9, 143.3, 143.1, 141.4, 138.0, 137.8, 137.7, 137.2, 134.3, 133.5, 133.4, 132.4, 131.3, 131.1, 130.2, 129.7, 128.7, 128.7, 128.6, 128.6, 128.5, 128.4, 128.3, 128.2, 128.2, 128.2, 128.1, 128.1, 128.0, 128.0, 127.9, 127.8, 127.7, 127.4, 127.3, 126.8, 125.3, 125.3, 124.4, 122.8, 120.3, 120.2, 100.4 (*β*-C1-GalNTCA), 98.1 (*β*-C1-GlcNTCA), 92.6 (CCl_3), 92.3 (CCl_3), 85.7 (*β*-C1-Man), 79.0, 78.7, 75.8, 75.7, 75.2, 74.3, 73.7, 73.7, 73.6, 73.3, 72.6, 71.4, 70.5, 70.2, 68.1, 64.4, 63.6, 55.6, 54.0, 46.7 (CH-Fmoc), 37.8, 29.8 (CH_3-Lev), 27.9 (CH_2-Lev).

HRMS (ESI): $C_{84}H_{79}Cl_6F_3N_2NaO_{20}S$ $[M+Na]^+$; calculated: 1759.2898, found: 1759.2882.

Optical rotation: $[\alpha]_D^{25}$ = -17.2° (c = 0.5, $CHCl_3$)

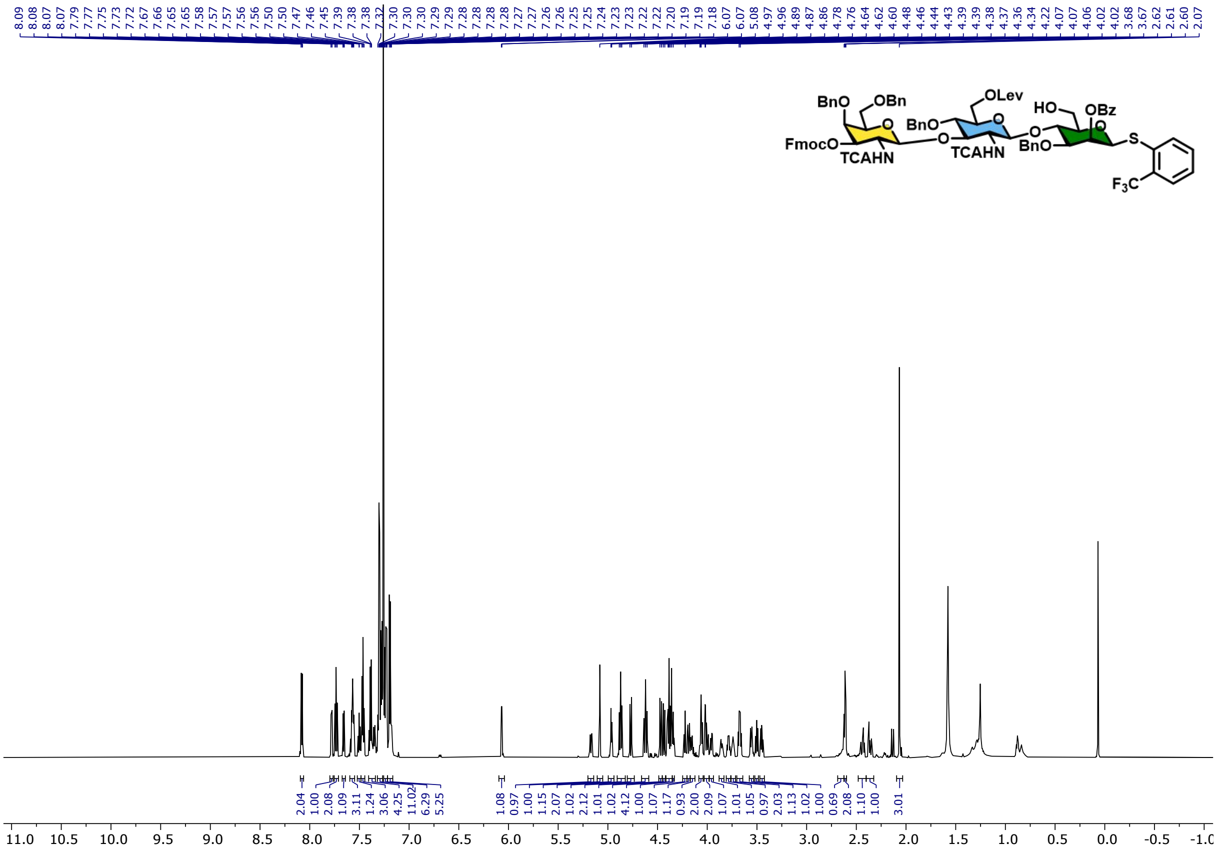

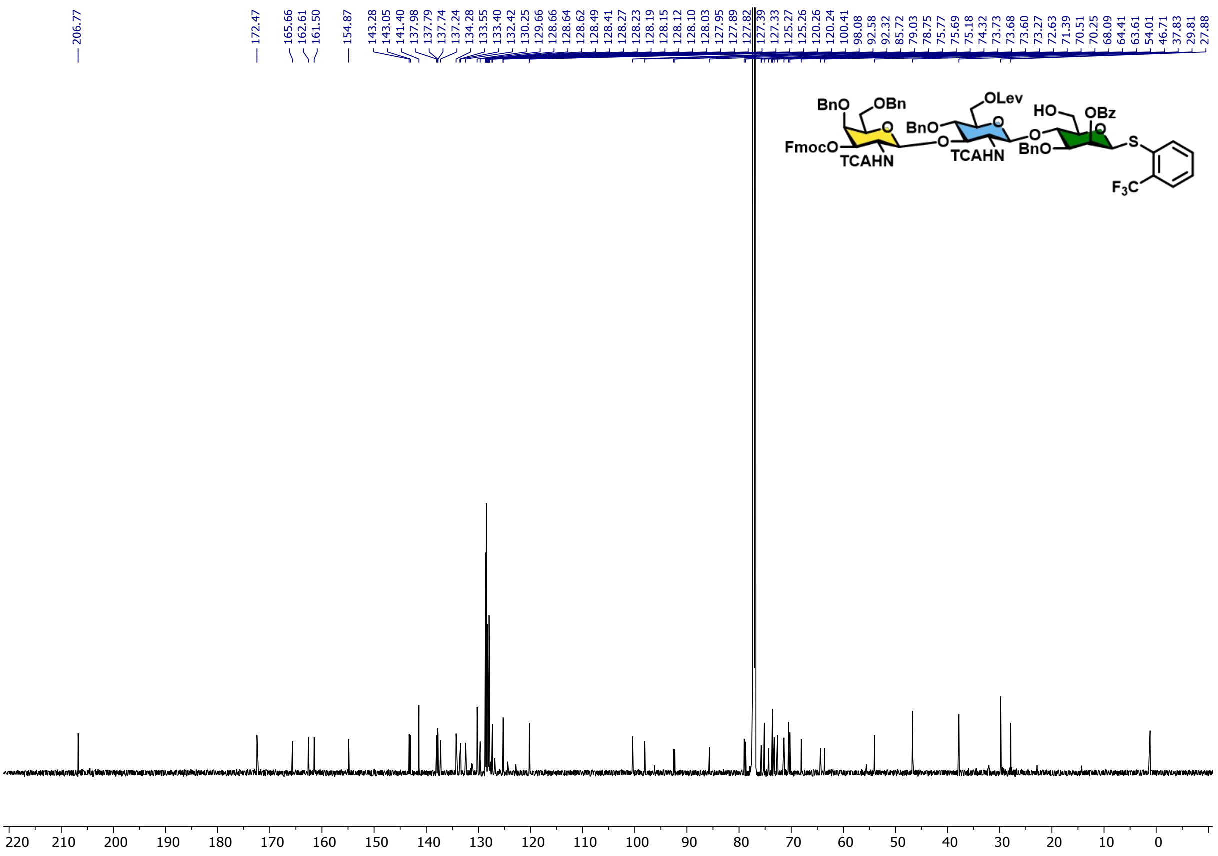

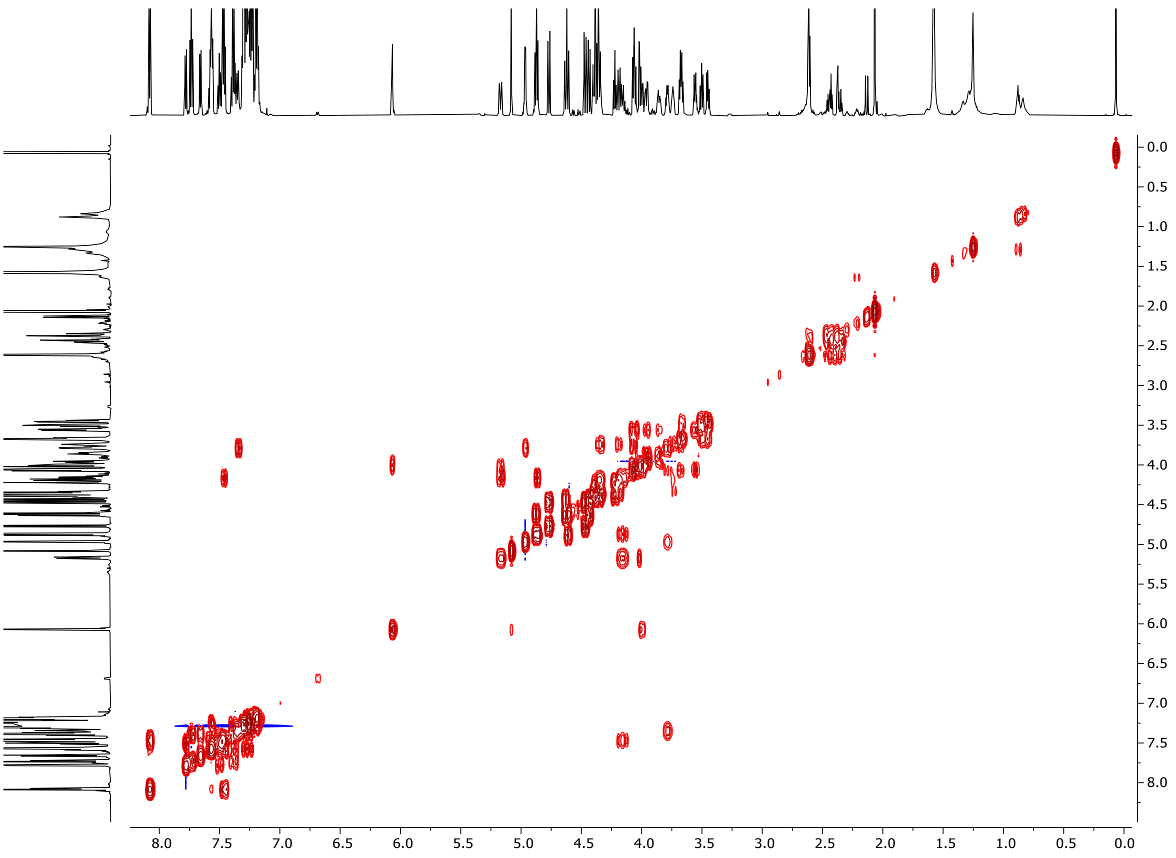

8.0
7.5
7.0
6.5
6.0
5.5
5.0
4.5
4.0
3.5
3.0
2.5
2.0
1.5
1.0
0.5
0.0

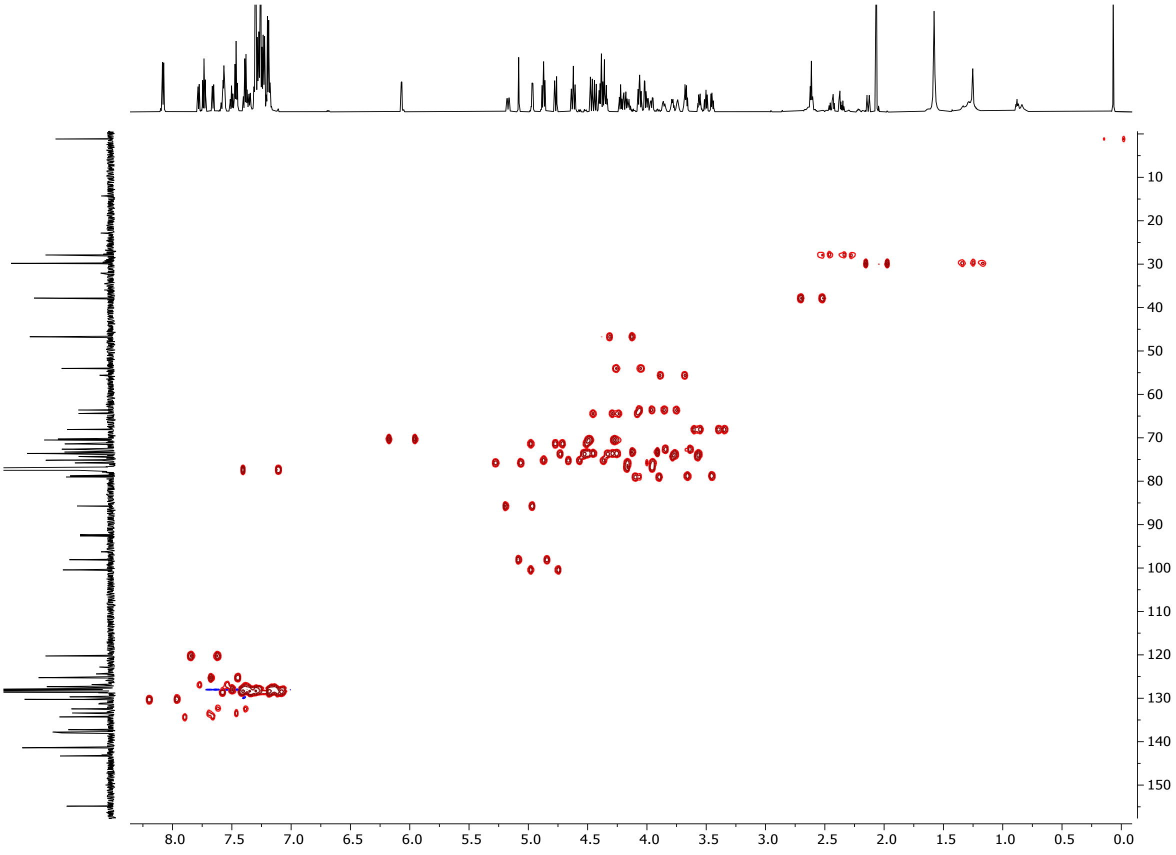

8.0
7.5
7.0
6.5
6.0
5.5
5.0
4.5
4.0
3.5
3.0
2.5
2.0
1.5
1.0
0.5
0.0
10
20
30
40
50
60
70
80
90
100
110
120
130
140
150

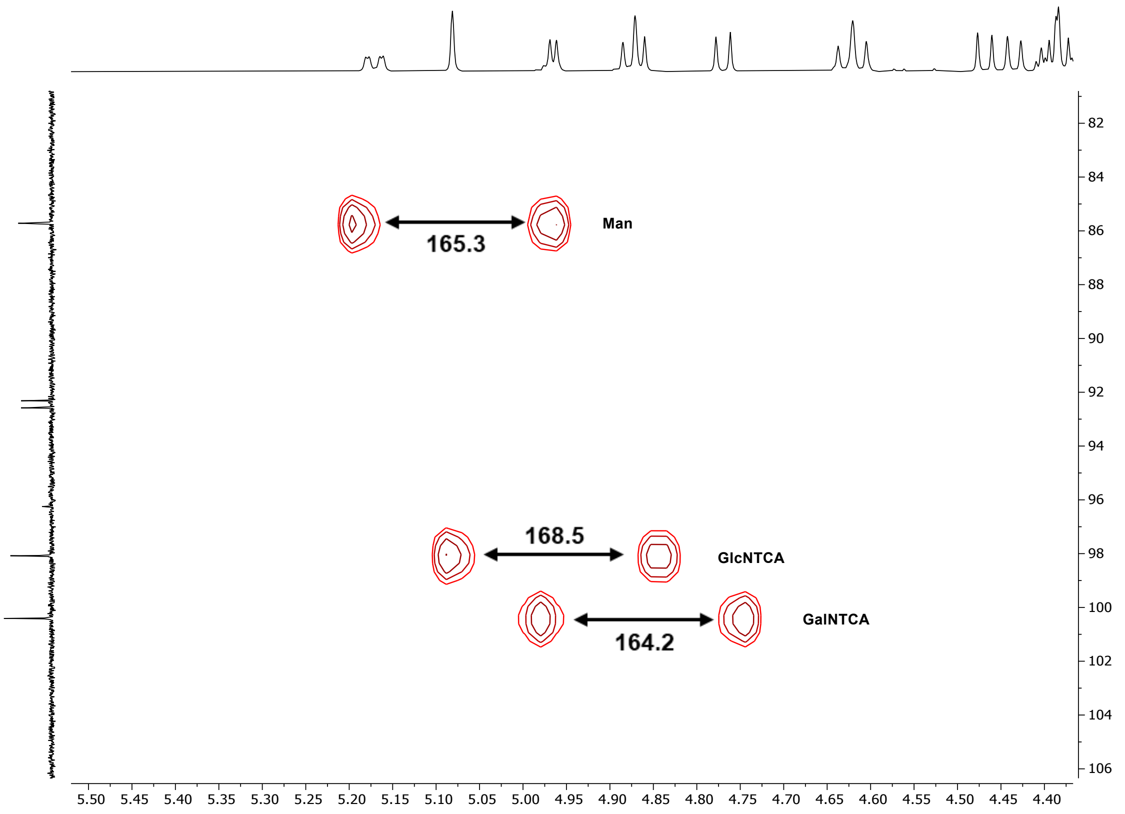

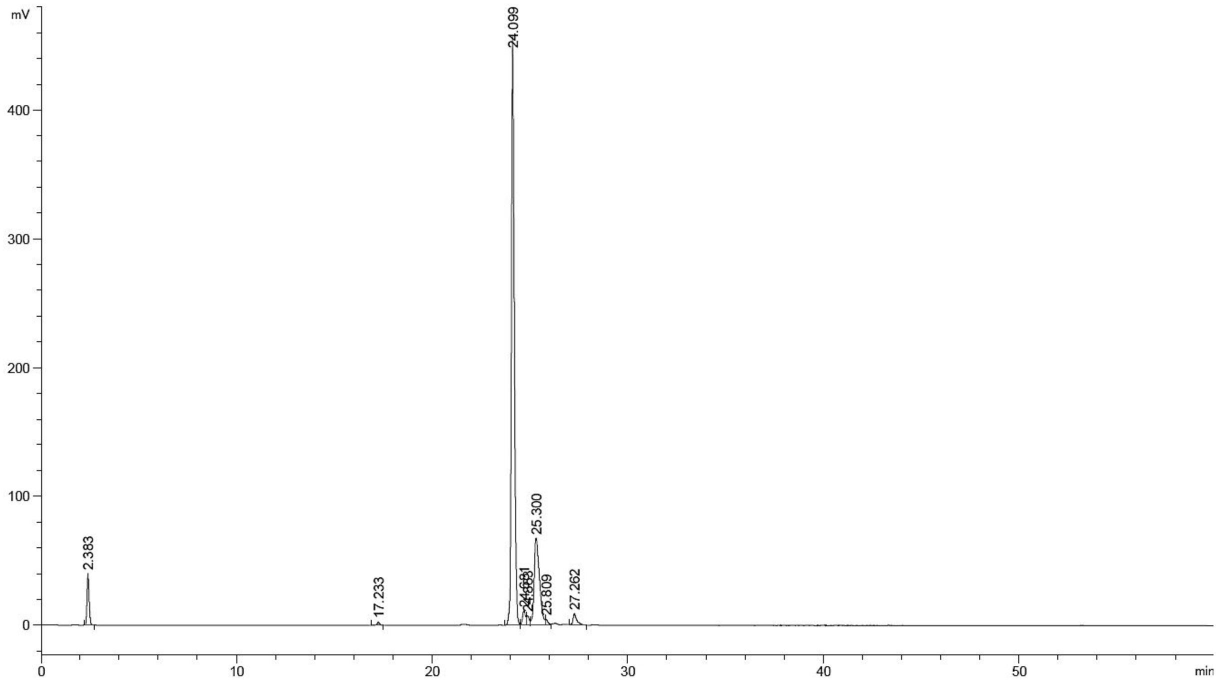

Figure 58: NP-HPLC trace of crude **41** (10-100%).

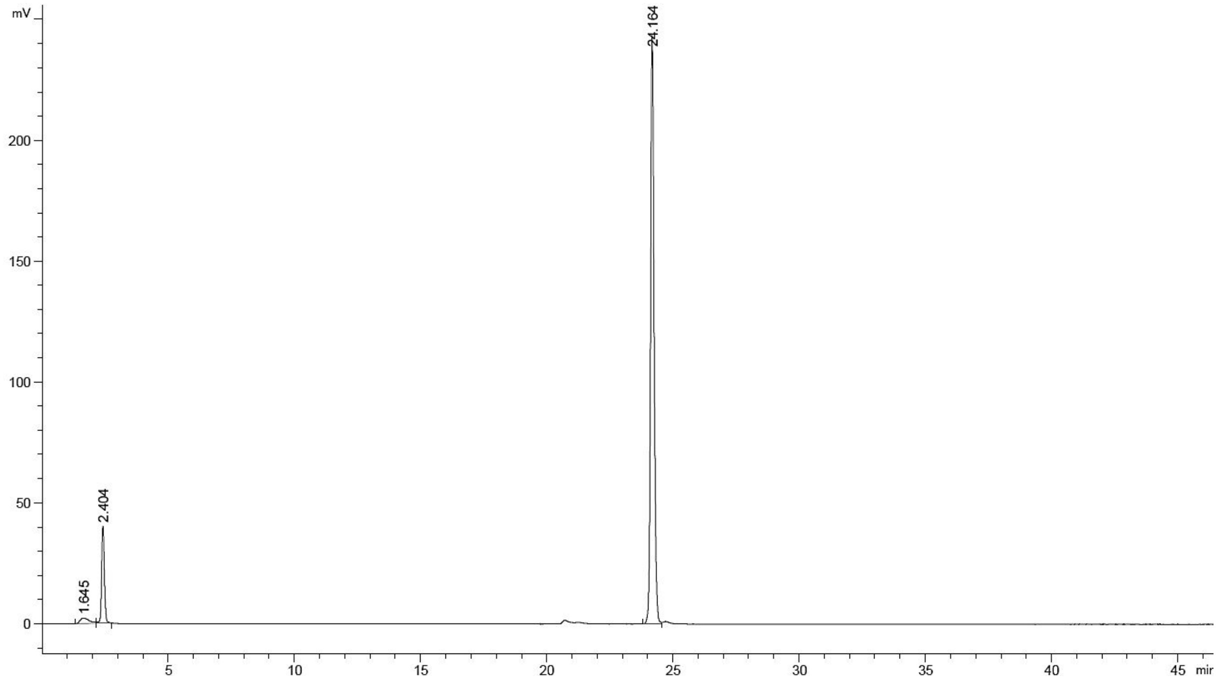

Figure 59: NP-HPLC trace of pure **41** (10-100%).

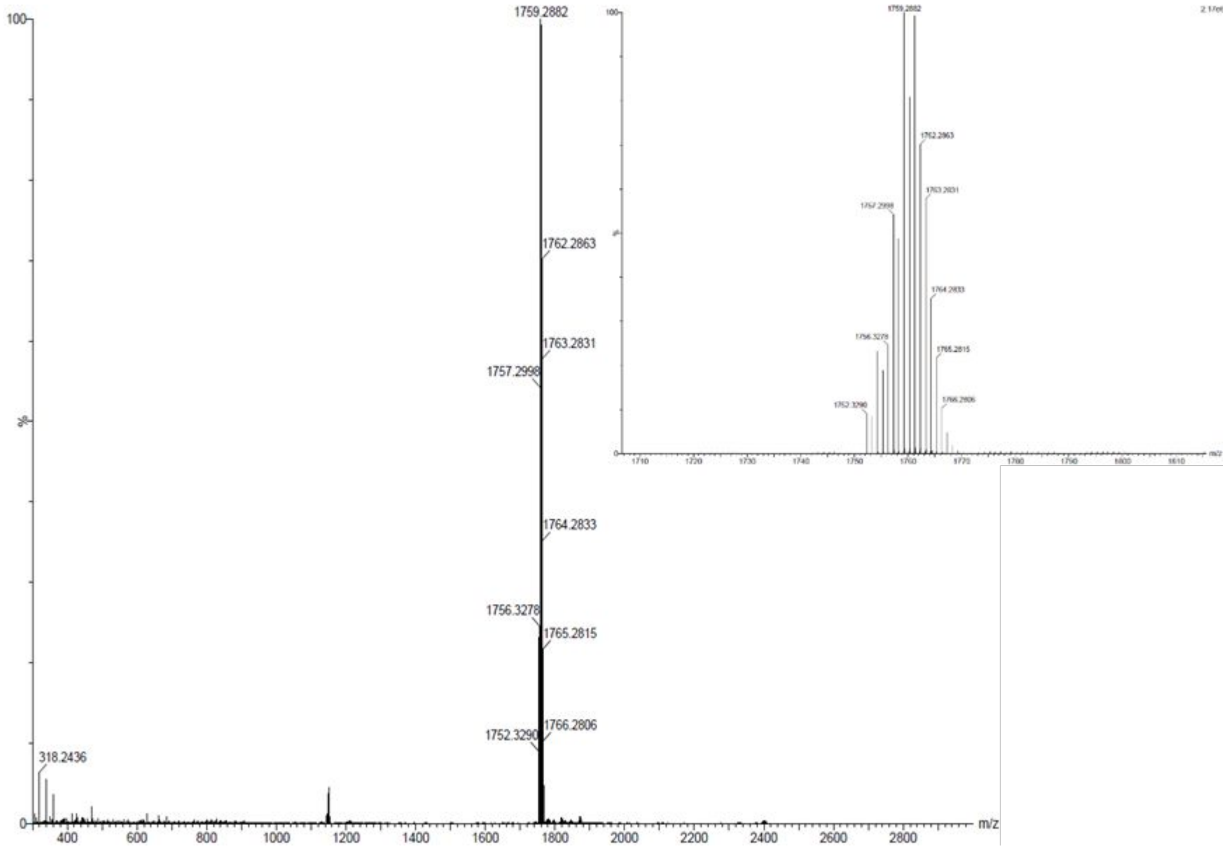

Figure 60: Q-TOF MS-spectrum of 4**1**.

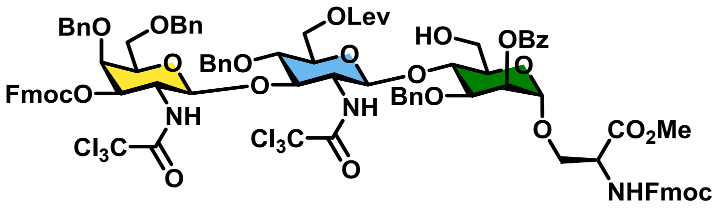

Methyl (((9H-fluoren-9-yl)methoxy)carbonyl)-*L*-serine 4,6-di-O-benzyl-3-O-(9-fluorenylmethoxycarbonyl)-2-[(2,2,2,-trichloroacetyl)amino]-*β*-D-galactopyranosyl-(1→3)-4-O-benzyl-6-O-levulinoyl-3-O-(9-fluorenylmethoxycarbonyl)-2-[(2,2,2,-trichloroacetyl)amino]-*β*-D-glucopyranosyl-(1→4)-2-O-benzoyl-3-O-benzyl-1-O-*α*-D-mannopyranosyl (42)

Step	Building Block	Modules	Notes
AGA	BB **9**, 6 eq.	**A** – first coupling	-40°C (T1) 5 min<br>-20°C (T2) 35 min
		**B** – RV Wash<br>**C** – Acidic Wash<br>**D** – Capping	
	BB **21**, 8 eq.	**E** – Fmoc Deprotection **Et$_3$N**<br>**C** – Acidic Wash<br>**G** – Thioglycoside coupling 2	18°C (T1) 10 min<br>35 °C (T2) 35 min
	double cycle<br>BB **39**, 3 eq.	**E** – Fmoc Deprotection **Et$_3$N**<br>**C** – Acidic Wash<br>**F** – Phosphate coupling<br>(half the amount of TMSOTf)	-40°C (T1) 15 min<br>-10°C (T2) 20 min
	double cycle<br>BB **40**(α), 3 eq.	**E** – Fmoc Deprotection **Et$_3$N**<br>**C** – Acidic Wash<br>**F** – Phosphate coupling<br>(half the amount of TMSOTf)	-40°C (T1) 15 min<br>-10°C (T2) 20 min
**Post AGA**		**J** – Batch Photocleavage	16 h
**Purification**		**L** – NP-(10-100% in 40 min)	R_t = 27.8 min

The coupling of the first BB to the resin is performed under standard conditions. All further couplings (procedure F) and acidic washes (procedure C) were performed with

a TMSOTf concentration half of the standard solution (0.45 mL in 80 mL CH_2Cl_2). Fmoc deprotection was performed according to protocol E using a 20% Et_3N solution and heating to 25°C instead of 50°C.

After a procedure including automated glycan assembly, photo-cleavage, purification, and lyophilization, **42** was obtained as a translucent resin (4.16 mg, 2.19 µmol, 11%).

R_t (NP - 10 – 100%) = 27.8 min.

^{1}H NMR (600 MHz, $CDCl_3$) δ 8.01 (dd, J = 8.2, 1.4 Hz, 2H), 7.76 – 7.71 (m, 4H), 7.63 (d, J = 7.5 Hz, 2H), 7.60 – 7.57 (m, 1H), 7.55 (d, J = 7.5 Hz, 2H), 7.45 (t, J = 7.8 Hz, 2H), 7.41 – 7.36 (m, 4H), 7.35 – 7.27 (m, 12H), 7.26 – 7.22 (m, 7H), 7.21 – 7.13 (m, 7H), 6.00 (d, J = 8.7 Hz, 1H, NH-Fmoc), 5.41 (t, J = 2.8 Hz, 1H, H2), 5.09 (d, J = 6.4 Hz, 1H, β-H1-GlcNTCA), 5.04 (dd, J = 11.4, 3.0 Hz, 1H, H3''), 4.88 (d, J = 2.2 Hz, 1H, *α*-H1-Man), 4.85 (d, J = 8.2 Hz, 1H, β-H1-GalNTCA), 4.75 (dd, J = 10.9, 7.1 Hz, 2H), 4.69 (d, J = 11.5 Hz, 1H), 4.62 (d, J = 11.4 Hz, 1H), 4.56 (m, 1H), 4.47 (d, J = 11.3 Hz, 1H), 4.46 – 4.42 (m, 2H), 4.41 (d, J = 7.4 Hz, 2H), 4.38 (d, J = 7.4 Hz, 2H), 4.34 (d, J = 11.8 Hz, 1H), 4.32 – 4.19 (m, 6H), 4.12 (t, J = 9.0 Hz, 1H), 4.07 – 4.01 (m, 3H), 3.92 (dd, J = 10.8, 3.1 Hz, 1H), 3.87 – 3.82 (m, 1H), 3.79 – 3.76 (m, 1H), 3.75 (s, 4H, CH_3-methylester), 3.70 – 3.63 (m, 2H), 3.61 (t, J = 7.0 Hz, 1H), 3.58 (d, J = 4.1 Hz, 1H), 3.54 (dd, J = 9.1, 7.6 Hz, 1H), 3.47 (dd, J = 9.1, 5.7 Hz, 1H), 2.58 (t, J = 6.5 Hz, 2H, CH_2-Lev), 2.43 (t, J = 6.5 Hz, 2H, CH_2-Lev), 2.09 (s, 3H, CH_3-Lev).

^{13}C NMR (151 MHz, $CDCl_3$) δ 206.6 (carbonyl-Lev), 172.6 (carboxyl-Lev), 170.5 (carboxyl-serine), 165.8, 162.4, 161.8, 156.2, 155.1, 143.9, 143.3, 143.0, 141.4, 141.4, 138.0, 137.9, 137.9, 137.6, 133.6, 130.0, 129.7, 128.7, 128.6, 128.5, 128.5, 128.4, 128.3, 128.1, 128.0, 128.0, 127.9, 127.7, 127.5, 127.4, 127.4, 127.3, 127.3, 125.4, 125.3, 125.3, 120.3, 120.2, 120.1, 100.3 (*β*-C1), 99.2 (*α*-C1), 98.9 (*β*-C1), 92.6 (CCl_3), 92.5 (CCl_3), 76.2, 75.4, 74.9, 74.2, 73.8, 73.6, 73.4, 72.9, 72.0, 71.5, 70.6, 70.1, 69.3, 67.9, 67.4, 63.6, 62.6, 57.9, 54.6, 54.0, 53.0 (CH_3-methylester), 47.3 (CH-Fmoc), 46.7 (CH-Fmoc), 37.9, 29.9, 27.9.

HRMS (ESI): $C_{96}H_{93}Cl_6F_3N_3NaO_{25}$ $[M+Na]^+$; calculated: 1922.4098, found: 1922.4110.

Optical rotation: $[\alpha]_D^{25}$ = +15° (c = 0.1, $CHCl_3$)

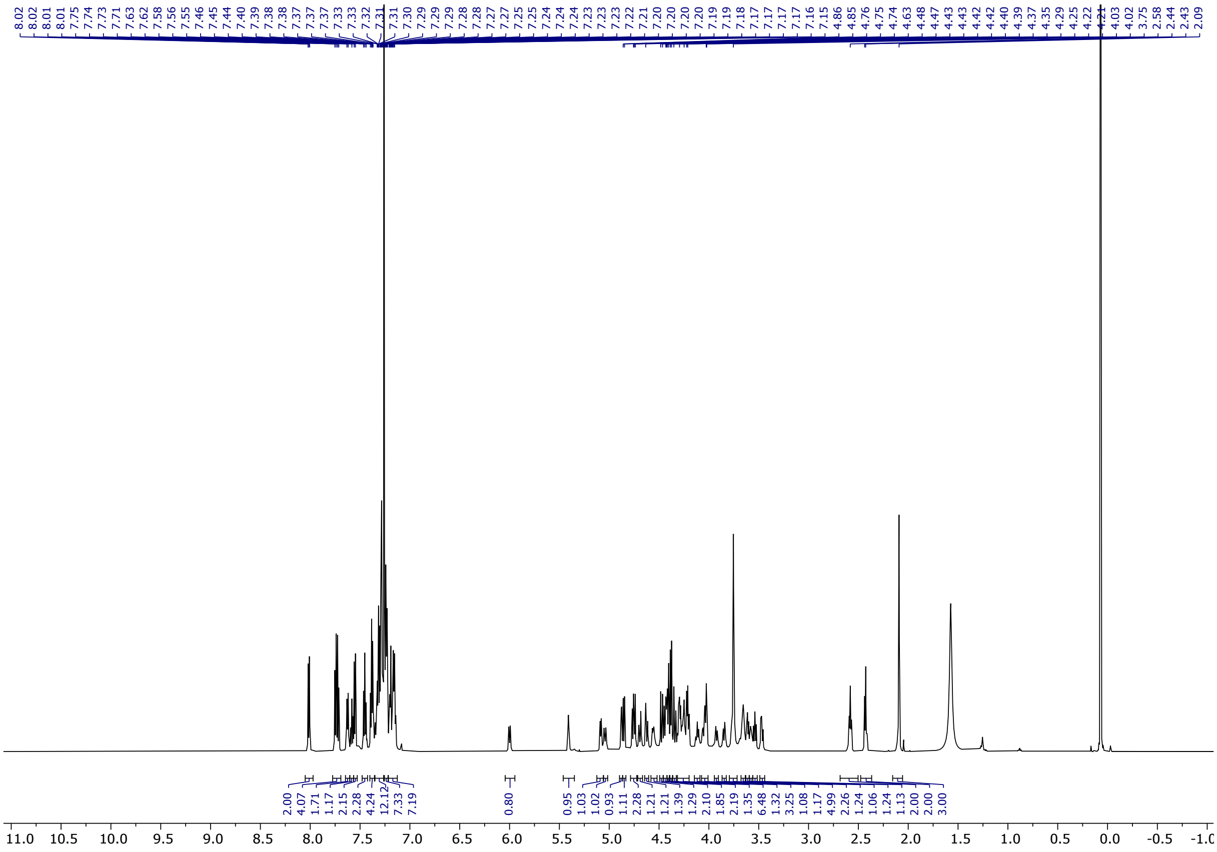

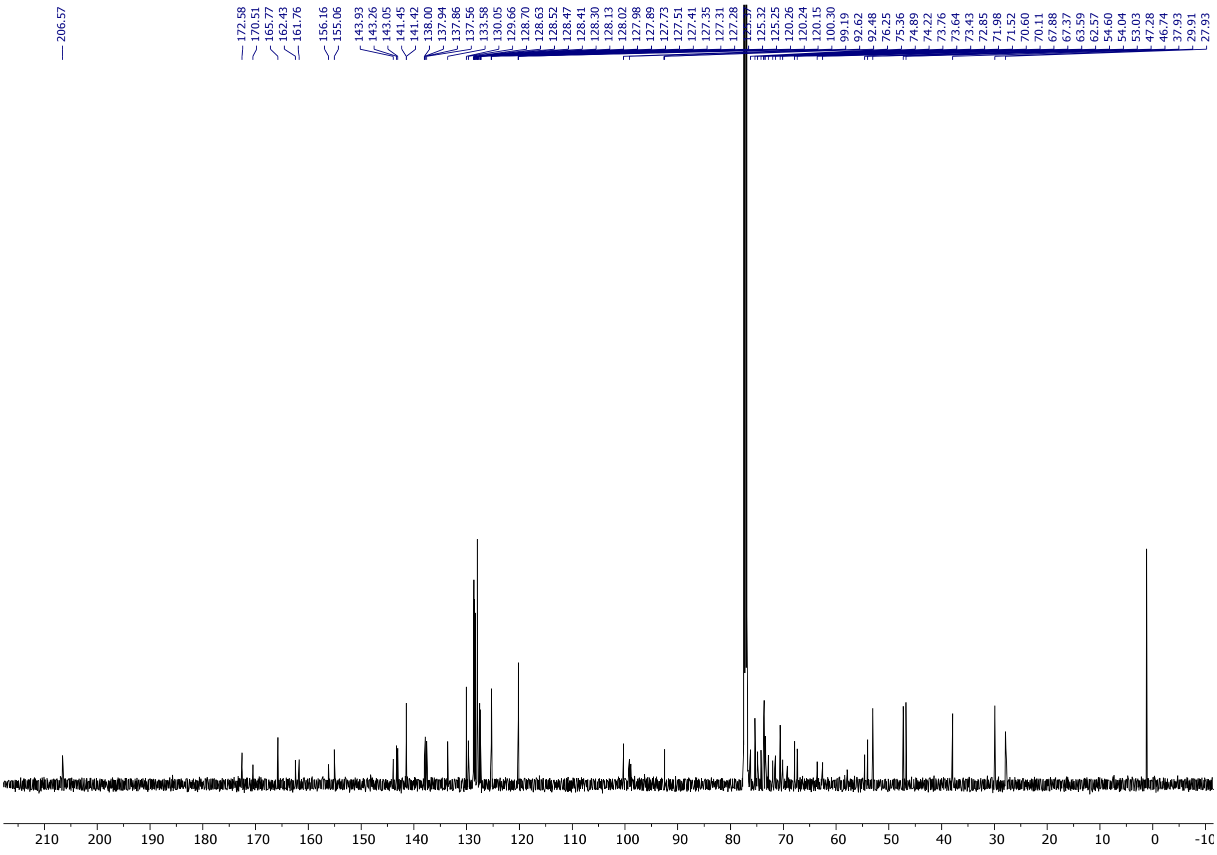

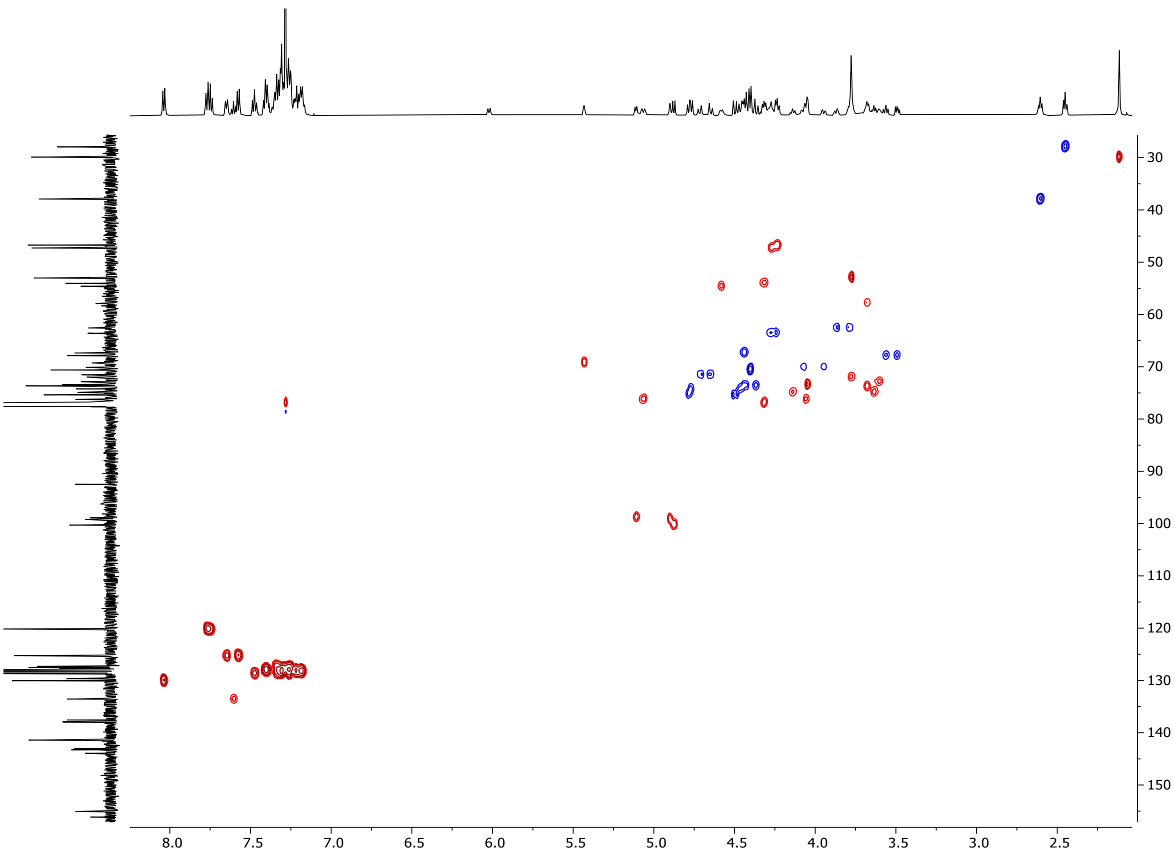
8.0
7.5
7.0
6.5
6.0
5.5
5.0
4.5
4.0
3.5
3.0
2.5
30
40
50
60
70
80
90
100
110
120
130
140
150

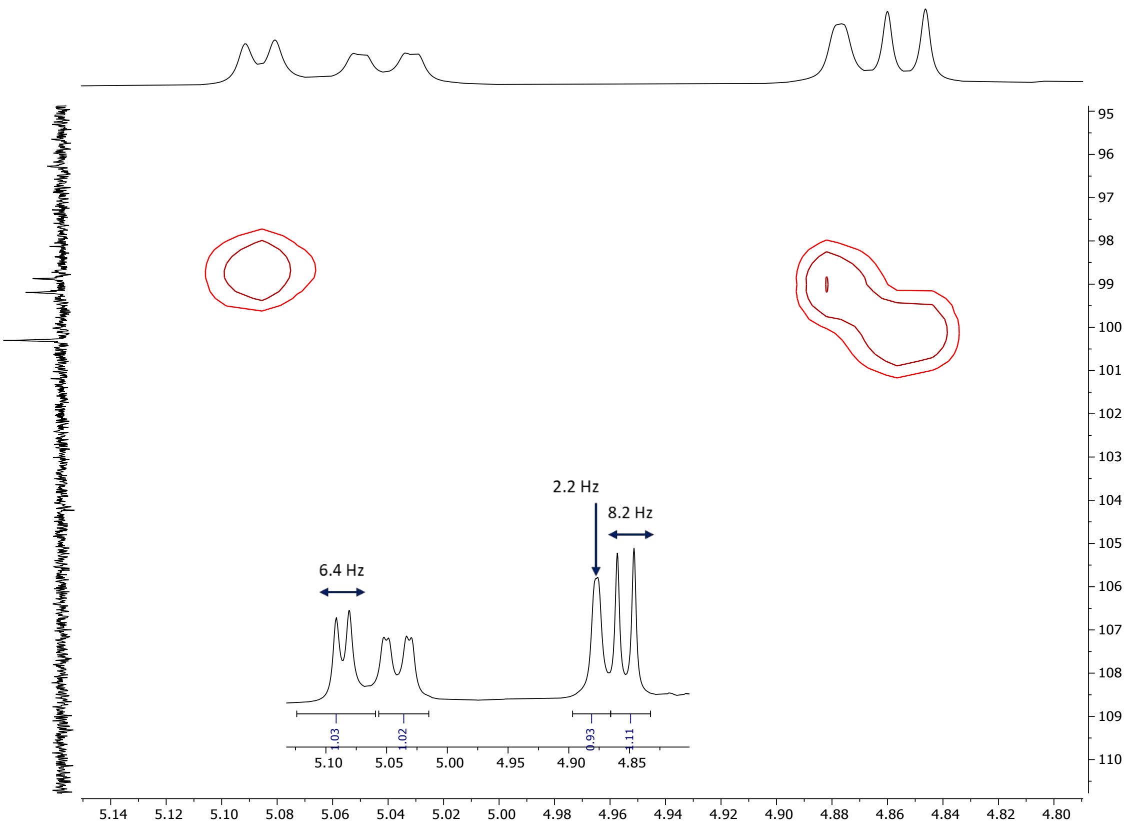
6.4 Hz
2.2 Hz
8.2 Hz
1.03
1.02
0.93
1.11
5.10
5.05
5.00
4.95
4.90
4.85
5.14
5.12
5.10
5.08
5.06
5.04
5.02
5.00
4.98
4.96
4.94
4.92
4.90
4.88
4.86
4.84
4.82
4.80
95
96
97
98
99
100
101
102
103
104
105
106
107
108
109
110

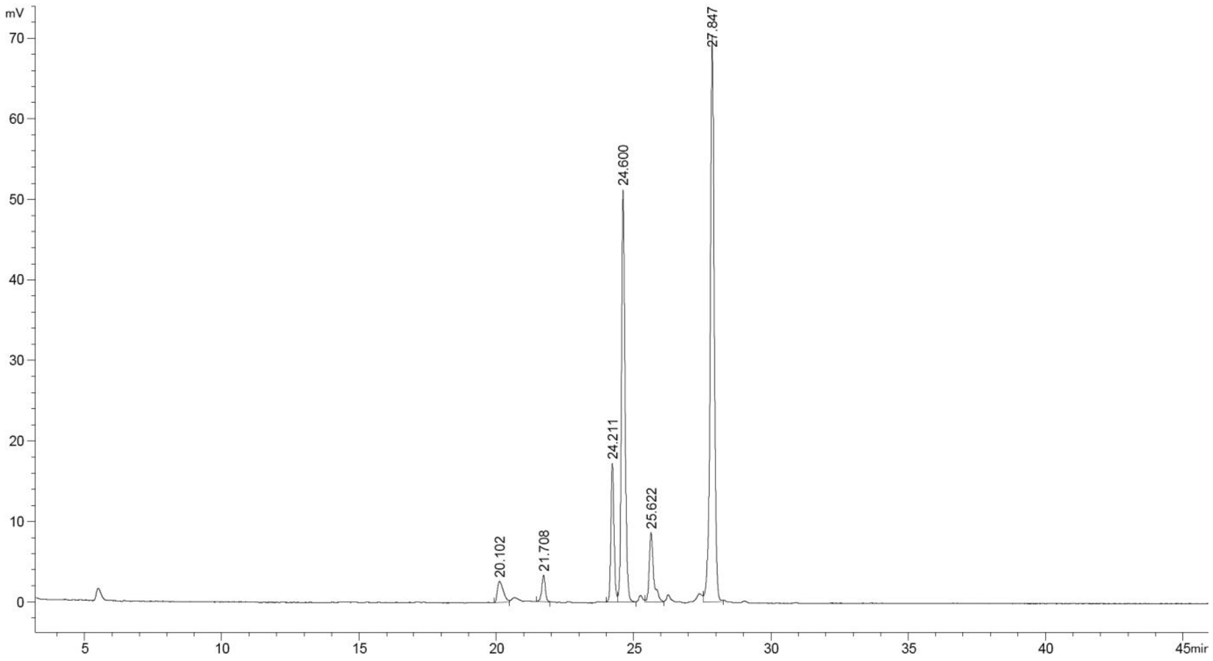

Figure 61: NP-HPLC trace of crude **42** (10-100%).

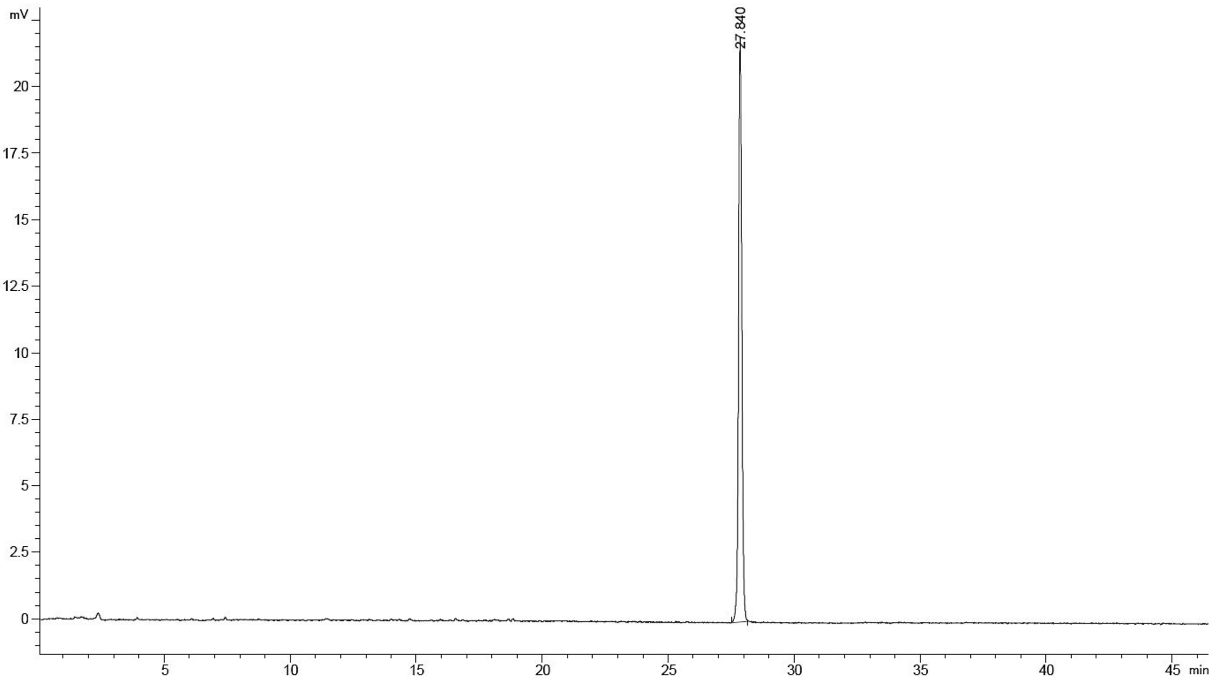

Figure 62: NP-HPLC trace of pure **42** (10-100%).

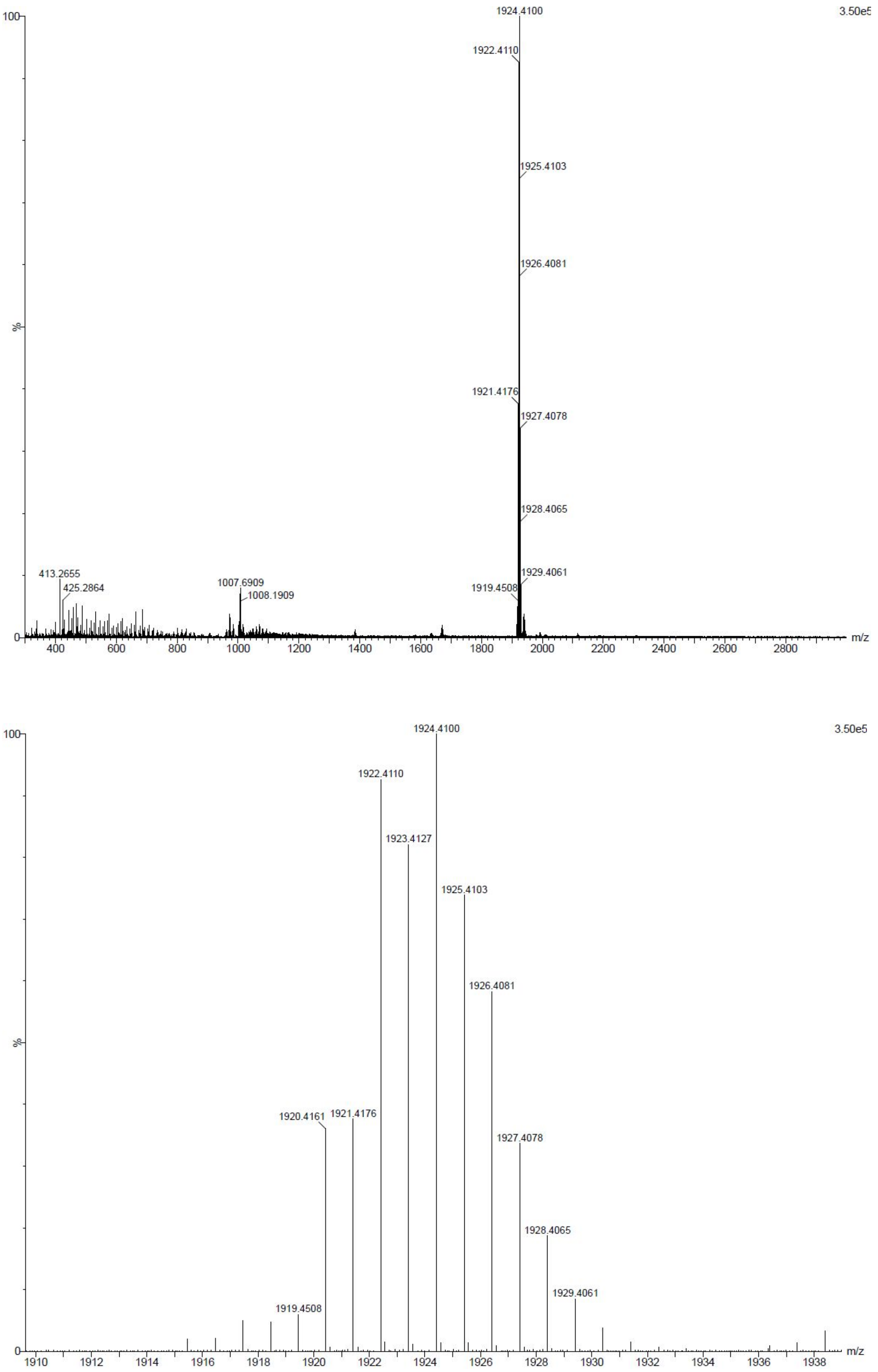

Figure 63: Q-TOF MS-spectrum of **42**.

References

(1) Danglad-Flores, J.; Sletten, E. T.; Reuber, E. E.; Bienert, K.; Riegler, H.; Seeberger, P. H. Optimized Platform for Automated Glycan Assembly. *Device* **2024**. DOI: 10.1016/j.device.2024.100499.

(2) Forsythe, N.; Liu, L.; Kashiwagi, G. A.; Demchenko, A. V. Activation of Thioglycosides under Mild Alkylation Conditions. *Carbohydr. Res.* **2023**, *531* (June), 108872. DOI: 10.1016/j.carres.2023.108872.

(3) Teschers, C. S.; Gilmour, R. Flow Photocleavage for Automated Glycan Assembly (AGA). *Org. Process Res. Dev.* **2020**, *24*, 2234–2239. DOI: 10.1021/acs.oprd.0c00286.

(4) Le Mai Hoang, K.; Pardo-Vargas, A.; Zhu, Y.; Yu, Y.; Loria, M.; Delbianco, M.; Seeberger, P. H. Traceless Photolabile Linker Expedites the Chemical Synthesis of Complex Oligosaccharides by Automated Glycan Assembly. *J. Am. Chem. Soc.* **2019**, *141*, 9079–9086. DOI: 10.1021/jacs.9b03769.

(5) Klán, P.; Šolomek, T.; Bochet, C. G.; Blanc, A.; Givens, R.; Rubina, M.; Popik, V.; Kostikov, A.; Wirz, J. Photoremovable Protecting Groups in Chemistry and Biology: Reaction Mechanisms and Efficacy. *Chem. Rev.* **2013**, *113*, 119–191. DOI: 10.1021/cr300177k.

(6) Bakhatan, Y.; Alshanski, I.; Grunhaus, D.; Hurevich, M. The Breaking Beads Approach for Photocleavage from Solid Support. *Org. Biomol. Chem.* **2020**, *18*, 4183–4188. DOI: 10.1039/d0ob00821d.
